# Extensive Copy-Number Variation of Young Genes across Stickleback Populations

**DOI:** 10.1371/journal.pgen.1004830

**Published:** 2014-12-04

**Authors:** Frédéric J. J. Chain, Philine G. D. Feulner, Mahesh Panchal, Christophe Eizaguirre, Irene E. Samonte, Martin Kalbe, Tobias L. Lenz, Monika Stoll, Erich Bornberg-Bauer, Manfred Milinski, Thorsten B. H. Reusch

**Affiliations:** 1 Department of Evolutionary Ecology, Max Planck Institute for Evolutionary Biology, Plön, Germany; 2 Institute for Evolution and Biodiversity, Evolutionary Bioinformatics, Westfälische Wilhelms University, Münster, Germany; 3 School of Biological and Chemical Sciences, Queen Mary University of London, London, United Kingdom; 4 Genetic Epidemiology, Westfälische Wilhelms University, Münster, Germany; 5 Evolutionary Ecology of Marine Fishes, GEOMAR Helmholtz Centre for Ocean Research, Kiel, Germany; University of Michigan, United States of America

## Abstract

Duplicate genes emerge as copy-number variations (CNVs) at the population level, and remain copy-number polymorphic until they are fixed or lost. The successful establishment of such structural polymorphisms in the genome plays an important role in evolution by promoting genetic diversity, complexity and innovation. To characterize the early evolutionary stages of duplicate genes and their potential adaptive benefits, we combine comparative genomics with population genomics analyses to evaluate the distribution and impact of CNVs across natural populations of an eco-genomic model, the three-spined stickleback. With whole genome sequences of 66 individuals from populations inhabiting three distinct habitats, we find that CNVs generally occur at low frequencies and are often only found in one of the 11 populations surveyed. A subset of CNVs, however, displays copy-number differentiation between populations, showing elevated within-population frequencies consistent with local adaptation. By comparing teleost genomes to identify lineage-specific genes and duplications in sticklebacks, we highlight rampant gene content differences among individuals in which over 30% of young duplicate genes are CNVs. These CNV genes are evolving rapidly at the molecular level and are enriched with functional categories associated with environmental interactions, depicting the dynamic early copy-number polymorphic stage of genes during population differentiation.

## Author Summary

After a locus is duplicated in a genome, individuals from a population instantaneously differ in the number of copies of this locus producing a copy-number variation (CNV). Over time, the joint effects of selection and other evolutionary forces will act to either eliminate the extra genetic copy or retain it. Depending on this evolutionary interplay, young duplications, including newly duplicated genes, can persist for millions of years as CNVs. CNVs may especially be prevalent between populations that have colonized and adapted to disparate environments in which selective pressures differ. Using whole genome sequences from several populations of three-spined sticklebacks that inhabit different environments, we find that a third of young duplicated genes are CNVs. These young CNV genes are enriched with environmental response functions and evolving rapidly at the molecular level, making them promising candidates for a role in the rapid ecological adaptation to novel environments.

## Introduction

Structural polymorphisms such as copy-number variations (CNVs) epitomize the dynamic nature of genomes. Inter- and intra-specific comparisons of whole genomes have revealed large genomic portions deleted and duplicated between individuals [1]–[7]. The substantial contribution of these CNVs to genetic diversity is fuelled by their high mutation rates, which have been estimated to be orders of magnitude greater than that of single nucleotide polymorphisms in mutation accumulation lines [8]–[12]. Although most deletions and duplications are thought to be under purifying selection and eventually eliminated from genomes [11], [12], high gene duplication rates provide ample opportunities for functional diversification and adaptation given the right ecological circumstances [13]–[16]. In this study, we report genomic CNVs across natural populations inhabiting distinct ecological niches, their evolutionary dynamics, and their putative role in local adaptation.

When a gene is initially duplicated, it appears as a CNV at the population level. That is, the duplication event occurs in one individual genome within the population, producing a locus that varies in quantity (copy-number) amongst individuals. Under neutrality, this early copy-number polymorphic stage of a new duplicate gene can persist for millions of years before fixation or loss in a population [17]. But the ultimate probability of (and time to) fixation depends on numerous factors including mutation rates, effective population size, and natural selection. As a small subset of CNVs may eventually give rise to new genes [18]–[20], their evolutionary dynamics can give insights into the earliest life stages of genes. New genes provide a platform for the evolution of novel functions [21]–[24], and their persistence may be associated with environmental adaptations [25]–[27]. Exposure to distinct environments thus sets the stage for differential gene loss and fixation, potentially reflecting local adaptation, and culminating in varying gene content between populations. Here we use population genomics to access the proportion of young genes that are CNVs across populations from different habitats.

The three-spined stickleback (*Gasterosteus aculeatus*) provides an excellent opportunity to evaluate the frequency, distribution and functional impact of CNVs and young genes as a response to local environmental conditions. Sticklebacks have repeatedly colonized and adapted to various freshwater habitats since the last glaciation period (approximately 12,000 years ago), making this fish an important evolutionary and ecological vertebrate model [28]–[30]. The ability of sticklebacks to thrive in several distinct environments and the associated recurrence of ecotypes and rapid adaptations may to some extent rely on ancestral variation and the differential sorting of CNVs and young genes among populations. Using 66 whole genomes, we have extended earlier scans of polymorphisms in sticklebacks [7], [30]–[33] to evaluate CNVs across populations. Our sampling design of replicated lake-river population pairs that have diversified post-glacially allows us to estimate CNV frequencies as well as gene gain and loss across individuals from the same population, from neighboring populations in distinct habitats, and from populations that have diverged at various time scales. The combination of population-level approaches with comparative genomics enables us to evaluate the dynamic evolution of young lineage-specific genes in stickleback genomes.

## Results

### Genome-wide copy-number variation in sticklebacks

**Figure 1:**
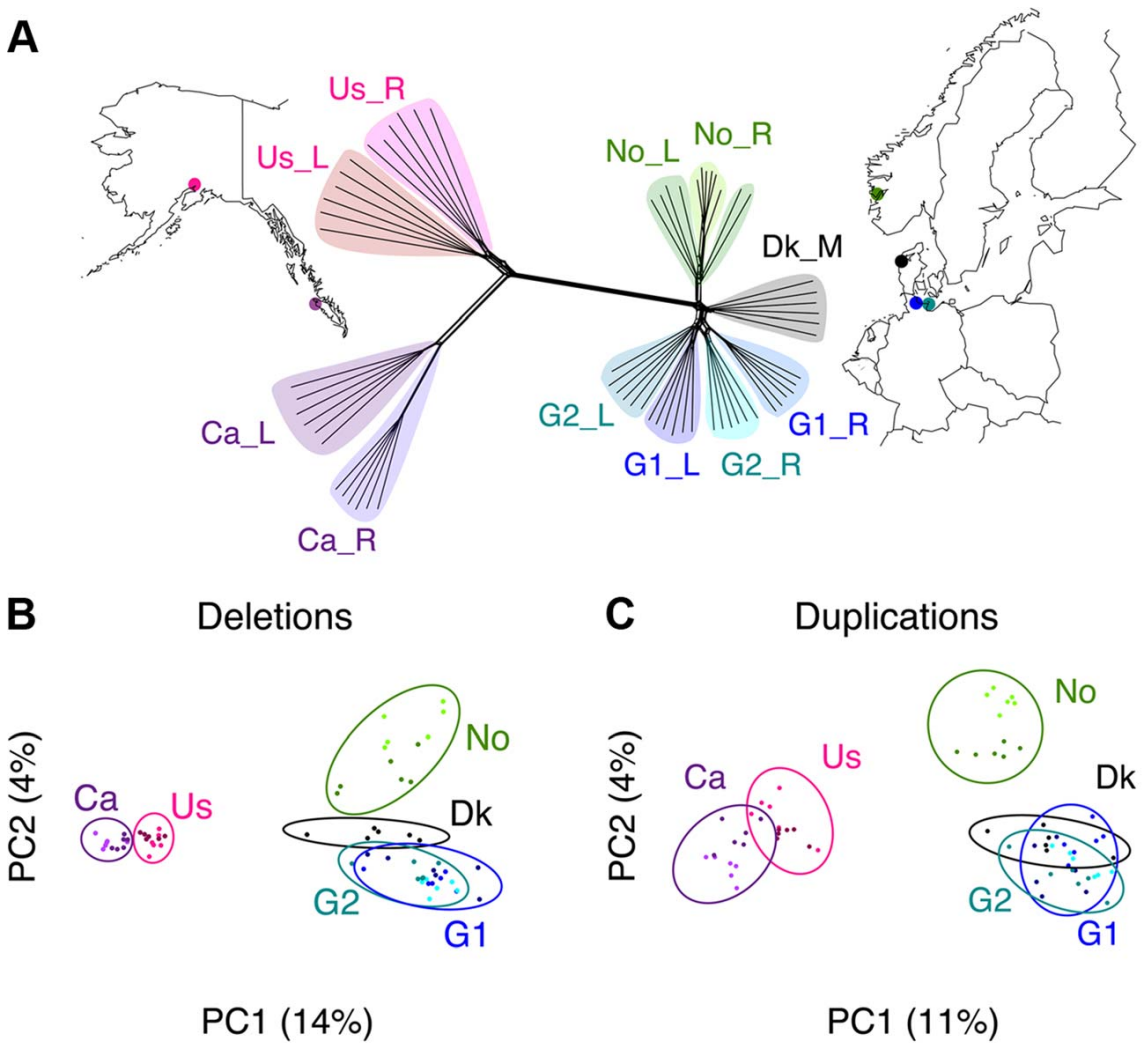
Phylogenomic relationships among samples. (**A**) Phylogenomic network of the 66 genomes was constructed using the Neighbor-net method based on 50,000 randomly selected high-quality SNPs. Parapatric population pairs were sampled from the United States (Us), Canada (Ca), Norway (No), and two sites in Germany (G1 and G2), and a marine population from Denmark (Dk). Ecotypes include rivers (_R), lakes (_L) and marine (_M) samples. (**B–C**) Principal components analysis plots of CNV deletions and duplications. Clustering of individuals is based on shared CNVs, which follow patterns of geographic distributions (highlighted with ellipses).

Three-spined sticklebacks were sampled from eleven populations across Europe and North America (herein referred to as “Atlantic” and “Pacific”) including lake, river/stream and marine populations (**Fig. 1A**). This sampling design allows for both an assessment of large-scale distribution of CNVs as a function of geographic proximity, and of the putative adaptive role of CNVs in two recently derived ecotypes from lakes and rivers. Whole genomes of 66 fish, six individuals from each population, were sequenced using two paired-end libraries (100 bp reads with 140 bp and 300 bp insert sizes) and a mate-pair library (50 bp reads with a 3 kb insert size), achieving an average depth of 26 fold and covering over 99% of the reference genome (**Table 1** and **S1 Table**, study accession number ERP004574).

**Table 1:** Summary of sequencing and variation statistics across sampled populations.

Population	Raw data (Gbp)	Average raw data depth (x fold) per individual	Clean data (Gbp)	Average clean data depth (x fold) per individual	Average % mapped read pairs	Final average depth of coverage	CNV regions (Mbp)
**Overall**	**1235.3**	**40.7**	**964.9**	**31.8**	**83.1**	**26.4**	3.6
Dk_M	97.3	35.3	84.4	30.6	77.7	**23.8**	1.9
G1_R	110.2	39.9	94.1	34.1	80.7	**27.5**	1.9
G1_L	132.9	48.2	106.3	38.5	78.8	**30.3**	2.0
G2_R	111.0	40.2	86.7	31.4	78.4	**24.6**	1.9
G2_L	92.8	33.6	73.0	26.5	77.5	**20.5**	1.9
No_R	98.4	35.7	85.6	31.0	85.0	**26.4**	1.6
No_L	96.2	34.8	83.3	30.2	86.2	**26.0**	1.7
Us_R	154.2	55.9	102.6	37.2	87.4	**32.5**	1.7
Us_L	127.8	46.3	95.1	34.4	87.4	**30.1**	1.7
Ca_R	108.0	39.1	71.2	25.8	87.0	**22.5**	1.6
Ca_L	106.6	38.6	82.5	29.9	87.7	**26.2**	1.6

More detailed information including population abbreviations and individual statistics can be found in **Table S1**.

CNVs were inferred based on three complementary approaches: depth of coverage, discordant paired-end mapping, and split-reads in comparison with the reference genome (see **Methods**). We found that the combined 66 stickleback genomes contained 758 duplications (mean length of 20,373 bp) and 3,550 deletions (mean length of 7,189 bp). After merging overlapping duplications and deletions we delineated a total of 3,898 CNV regions covering 36.3 Mbp (∼8% of the genome, mean length of 9,310 bp). The pattern of CNV sharing across individuals follows the geographic and phylogenomic distributions (**Fig. 1B-C** and **S1 Figure**). Based on independent PCR validation of CNVs, we confirmed the presence of 96% of the tested CNV loci (7/7 deletions and 15/16 duplications) and recovered 88% (284/321) concordant genotypes among these CNV loci (**S13 Table** and **S2 Figure**).

### Most CNVs are at low frequencies

**Figure 2:**
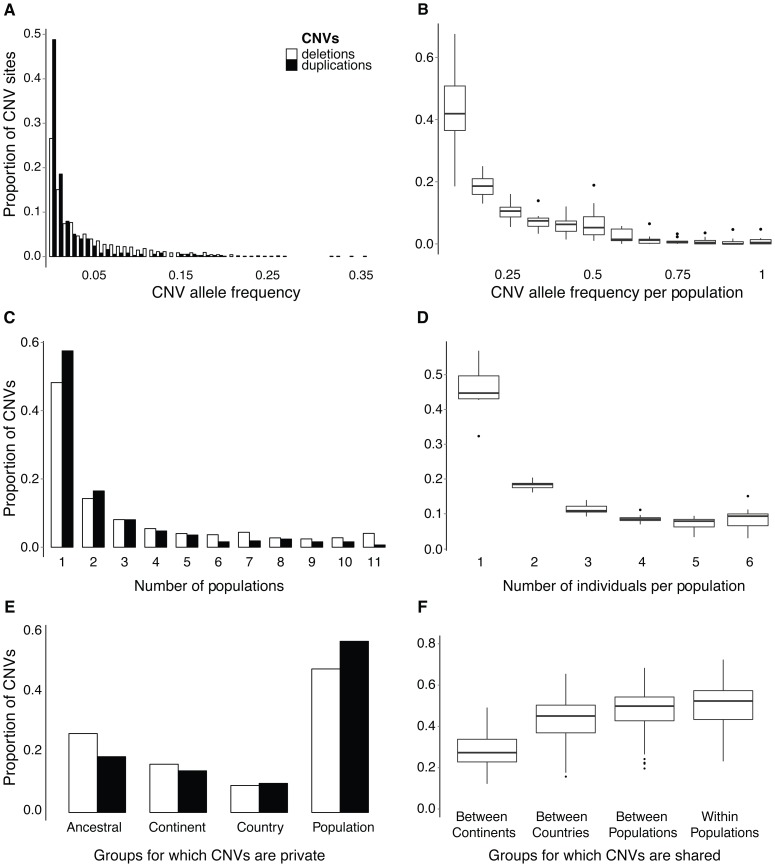
Frequency and occurrence of CNVs across individuals and populations. **(A)** Allele frequency spectrum of bi-allelic CNVs across all 66 individuals, showing most deletions (white) and duplications (black) occurring at very low frequencies. See **S3B Figure** for a comparison with intergenic SNPs. (**B**) Allele frequency spectrum of non-reference alleles from bi-allelic CNVs across 12 individuals from each population represented as boxplots. (**C**) The occurrence of CNVs across populations. (**D**) The proportion of CNVs shared across individuals within populations. (**E**) The proportion of CNVs specific (private) to groups of individuals depending on the scale of aggregation. Mutually exclusive groups for which CNVs are private include: those occurring across continents (Ancestral), those specific to a continent but shared across populations from different countries (Continent), those specific to a country but shared across populations within a country (Country), and those only found in one population (Population). (**F**) The average proportion of shared CNVs between individuals across mutually exclusive groups. The proportion of CNV sharing across individuals was calculated for four groups: “Between Continents” is sharing from different continents, “Between Countries” is sharing from different countries within the same continent, “Between Populations” is sharing from different populations from the same country, and “Within Populations” is sharing from the same population.

We first address the question of how CNVs are distributed among individuals and across populations using both the allele frequency spectrum and CNV presence/absence per individual. Allelic frequencies were analyzed using genotypes inferred at bi-allelic sites, constituting 38% of all CNVs (**S2 Table)**. CNVs generally occur at very low frequencies across all individuals (**Fig. 2A**) and are rarely “fixed” (all homozygous) within populations (**Fig. 2B**); 72% of bi-allelic CNVs are found at overall frequencies below 0.05, wherein about half of all bi-allelic duplications are singletons found in a single individual (**S3A Figure**). Overall, CNVs are maintained at lower frequencies than intergenic SNPs (**S3B Figure**) and although some CNVs are shared among multiple populations (**Fig. 2C**), they are often heterozygous with a high abundance of singleton alleles. We also found that CNVs fully overlapping genes have higher allele frequencies and are found in more individuals than other CNVs (**S4** and **S5 Figures**). An excess of low frequency variants can be caused by selection, but demographic processes (bottlenecks and population expansions) and population structure can also elicit similar patterns [34]. Given our limited sample size per population, we used our whole dataset to get more reliable variant frequency estimates, accepting the existence of an underlying population structure. However, since we compare CNVs with intergenic SNPs called form the same dataset, both types of variants have experienced the same demographic history. To evaluate the potential influence of selection on the frequencies of CNVs versus SNPs, we estimated the scaled coefficient of natural selection γ and 95% confidence intervals (CI) using the Poisson Random Field approach implemented in the program prfreq [35]. Whereas intergenic SNPs appear to be near neutral (γ = 0.2, CI 0.1 to 0.3), purifying selection may be shaping the distribution of both deletions (γ = −2.2, CI −2.0 to −2.5) and duplications (γ = −5.4 CI −4.6 to −6.8). Taken together, CNVs appear to be for the most part deleterious compared to intergenic SNPs based on the allele frequency spectrum.

Although most CNVs are at low frequencies, a few bi-allelic CNVs (1%) are fixed within populations (**Fig. 2B**), and a total of 370 CNVs (9%) are present at least in a heterozygous state in all six individuals from at least one population (**Fig. 2D**). Most of these CNVs are shared across multiple populations suggesting they are of ancestral origin, although five are specific to a population and ten are specific to a group of neighboring populations. The aforementioned private CNVs intersect ten genes in total, including a G-protein signaling modulator (*SGSM3*), calmegin (*CLGN*) - a gene associated with spermatogenesis in mammals, an enzymatic gene (*B4GALNT2*) that is duplicated in marine individuals, and transposable element (*POGK*) members that are deleted in Atlantic individuals (**S21 Figure**). Other private CNVs intersect non-coding regions such as a fixed (homozygous) deletion upstream of an opsin gene (*TMTOPSA*) in a German river (G1_R) population (**S3 Table**). In general though, CNVs are copy-number polymorphic within populations **(** **Fig. 2D** **)**.

Given our sampling design, we were able to evaluate the extent of CNV sharing between populations with different divergence times. This revealed that 50% of CNVs are population specific in that we only detect them in a single population, and another 25% are shared across continents (**Fig. 2E**). Despite generally low frequency estimates, many CNVs are shared across populations either due to gene flow, incomplete lineage sorting from ancestral polymorphisms pre-dating population divergence, or recurrent mutations. The extent of CNV sharing between individuals follows patterns of common ancestry that decreases with geographic distance (**Fig. 2F** **and S6 Figure**).

### Substantial differences in gene content between individuals

**Figure 3:**
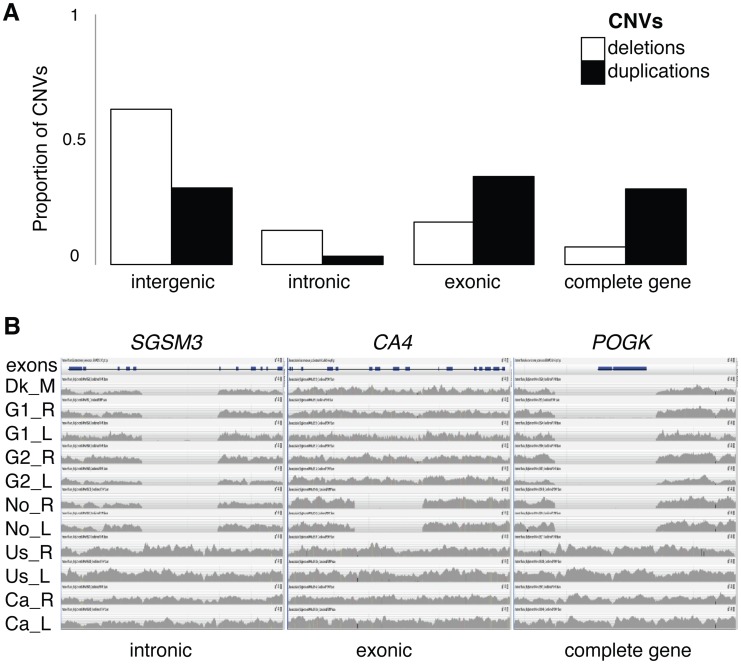
CNV proportions across genomic regions and homozygous deletions. (**A**) Proportion of deletions (white) and duplications (black) overlapping four mutually exclusive genomic categories; entirely within intergenic regions, entirely within intronic regions, partially overlapping a gene including an exonic region, or completely overlapping at least one gene. (**B**) Read depth coverage (grey histograms) along a gene (blocks are exons) for one representative individual from each population, showing CNV deletions. Examples include an intronic loss from a small G protein signaling modulator 3 gene (*SGSM3*) in Atlantic individuals, an exonic partial gene loss of a carbonic anhydrase gene (*CA4*) in Norwegian individuals, and the complete gene loss of a pogo transposable element with *KRAB* domain gene (*POGK*) in Atlantic individuals.

To evaluate the effect of CNVs on the emergence, duplication and loss of genes, we focus the rest of our analyses on the genes encompassed in CNV regions, herein referred to as “CNV genes”. Both deletion CNVs and duplication CNVs harbor genes, but despite being more abundant and covering more nucleotides, deletion CNVs were preferentially found in non-coding regions compared to duplications (χ^2^ with Yates correction, *p*<0.0001) even after correcting for CNV length (**S1 Text**); only 7% of deletion CNVs overlap entire genes compared with 33% of duplication CNVs (**Fig. 3A**). In total, we found 1,016 protein-coding genes (5%) and 174 RNA genes (11%) that are autosomal CNV genes (**S4 Table** and **S7 Figure**). CNV genes mirror the distribution patterns of total CNVs; CNV genes are found at low frequencies, are generally population specific, and are more often shared between individuals from adjacent populations **(S8–S12 Figures)**.

A subset of CNV genes (86 protein-coding, 7 RNA genes and 2 pseudogenes) was identified as “gene losses”, genes that were present in some genomes but completely missing from others due to homozygous deletions (**Fig. 3B** **and S5 Table**). A detailed analysis of unmapped reads suggests that the identification of CNV genes, including gene losses, is minimally affected by a reference genome bias (**S1 Text**). Based on the assembly of unmapped reads we detected over 100 putative genes that are not currently assembled in the reference genome [30], however these are generally short contigs and could represent partial genes or pseudogenes (**S6 Table**). Based on mapped data, each individual was found to have on average 142 CNV genes (ranging from 68 to 236) and 22 gene losses (ranging from 5 to 39, **S7 Table**), leading to two individuals differing on average by a combined 242 CNV genes (1.1% of genes).

### CNV genes are predominantly young genes

**Figure 4:**
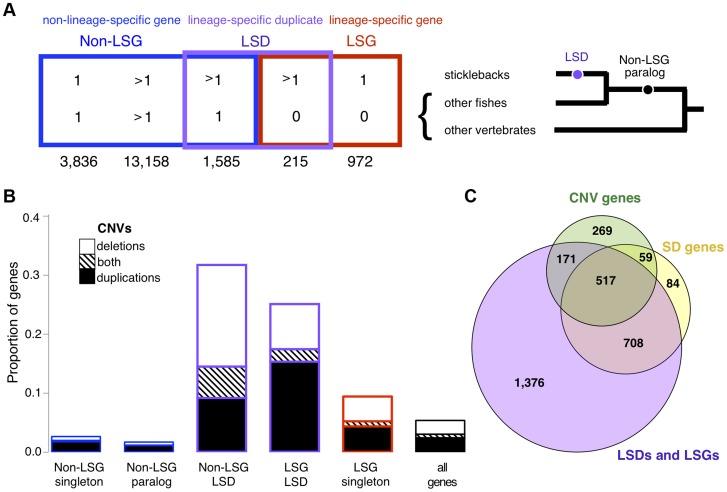
Relationship between young genes and CNVs. (**A**) Five mutually-exclusive gene categories based on orthology and paralogy: Non LSGs (Non-Lineage Specific Gene singletons), Non-LSG paralogs (Non-Lineage Specific Gene paralogs), Non-LSG LSD (Non-Lineage Specific Genes that are Lineage-Specific Duplicates), LSG LSD (Lineage-Specific Genes that are Lineage-Specific Duplicates) and LSG singletons (Lineage-Specific Gene singletons). Young genes are determined from broader overlapping categories, inferred as having no detectable orthologs (LSGs: lineage-specific genes) or recent paralogs (LSDs: lineage-specific duplications). LSGs are normally singletons but can also be duplicated, in which case they are both LSGs and LSDs (LSG LSD). LSGs and LSDs were found to have characteristic properties of young genes such as short gene lengths, narrow gene expression and rapid molecular evolution (**S1 Text**). The relationship of corresponding genes across and within species (orthologs and paralogs) is represented for each category, wherein “>1” represents paralogs and “0” represents no detectable ortholog. For example, non-LSG singletons have a one-to-one relationship between sticklebacks and at least one other species, non-LSG paralogs have a many-to-many relationship due to an old duplication event (black dot in tree), and non-LSG LSD have a one-to-many relationship due to a recent duplication event along the stickleback lineage (purple dot in tree). The number of autosomal protein-coding genes belonging to each category is reported below each category name. (**B**) Proportion of genes (both protein-coding and RNA) completely encompassed within CNV regions (deletions in white, duplications in black, both deletion and duplication in stripes). (**C**) Overlap between protein-coding genes in segmental duplication (SD genes), CNV genes, and LSGs and LSDs. The majority of SD genes are LSGs and LSDs, many of which are also CNV genes.

As newly duplicated genes undergo an early evolutionary stage that consists of copy-number polymorphism at the population level, we rationalized that young genes that have not reached copy-number fixation would be well represented among CNV genes. To evaluate the proportion of young genes that are copy-number fixed versus copy-number polymorphic, we first identified young genes using a comparative genomics approach based on orthology and paralogy relationships from Ensembl (**Fig. 4A**). Young genes included lineage-specific genes (LSGs) that are only found in sticklebacks (putative orphan genes) and lineage-specific duplications (LSDs) that have recently duplicated in sticklebacks, both of which show hallmarks of new genes (**S1 Text**). Concordant with our expectations, a substantial overrepresentation of young stickleback genes overlaps with CNVs (χ^2^ with Yates correction, *p*<0.0001, **Fig. 4B**) including gene losses (homozygous deletions; χ^2^ with Yates correction, *p*<0.0001). About a third of LSDs and 10% of LSG singletons are CNV genes (**S4 Table**), indicating that many young genes have not been fixed across populations since they emerged, or that recurrent mutations causing CNVs preferentially involve young genes. The CNVs in LSGs and LSDs have fewer singletons and higher allele frequencies than those in non-LSGs (Mann-Whitney test W = 4631, *p* = 4.672e-06), demonstrating that CNVs in young genes are not simply single events.

LSGs and LSDs are on average shorter in length than other genes (**S1 Text**). After correcting for gene length (see **Methods**), we found that protein-coding LSGs and LSDs remain overrepresented among both deletions and duplications (*p*<0.001) whereas non-LSGs are underrepresented (*p*<0.001). Similar results are also found for RNA genes (**S1 Text**). A comparative molecular analysis (see **Methods**) revealed that, like young genes, CNV genes are evolving rapidly at the sequence level, with higher dN (nonsynonymous substitutions per nonsynonymous site) compared to non-CNV genes (Mann-Whitney test W = 505097.5, *p* = 1.482e-06) and higher dN/dS (the ratio of nonsynonymous substitutions per nonsynonymous site versus synonymous substitutions per synonymous site; Mann-Whitney test W = 477084.5, *p* = 0.0004). Elevated dN and dN/dS are found for both deletion CNV genes (*p* = 2.742e-06 and *p* = 0.0003 respectively) and duplication CNV genes (*p* = 0.0011 and *p* = 0.0172 respectively) when analyzed separately. These results suggest that the early evolution of young genes involves a period of relaxed purifying selection or positive selection, which may promote their genomic persistence. Surprisingly, almost half of the autosomal genes (9/20) found to be under positive selection from the Selectome database [36], [37] are CNV genes, and all are LSDs (**Table 2**).

**Table 2:** Genes under positive selection in the stickleback lineage from the Selectome database, indicating whether they are deletions, duplications, or both.

Chromosome	Start	Gene ID	Orthology	Gene Name	CNV
groupIV	25707274	ENSGACG00000019515	Non-LSG LSD	NXPE3	del
groupIX	18937351	ENSGACG00000019717	Non-LSG LSD	TBCE	
groupXI	4320058	ENSGACG00000007447	Non-LSG LSD	GVIN1	both
groupXI	4500951	ENSGACG00000007454	Non-LSG LSD	GVIN1	del
groupXIII	17421767	ENSGACG00000013874	Non-LSG LSD	UGT2A2	del
groupXIII	17427540	ENSGACG00000013879	Non-LSG LSD	UGT2A2	del
groupXX	2326982	ENSGACG00000004471	Non-LSG LSD	Sult3a1	
scaffold_1180	355	ENSGACG00000000056	Non-LSG LSD	Sult3a1	
scaffold_1239	2045	ENSGACG00000000553	LSG LSD	NLRC3-like	
scaffold_148	130613	ENSGACG00000001242	Non-LSG LSD	ASZ1	
scaffold_171	135483	ENSGACG00000001045	Non-LSG LSD	TBCE	dup
scaffold_215	39094	ENSGACG00000001200	LSG LSD	NLRC3-like	
scaffold_255	27530	ENSGACG00000001313	LSG LSD	NLRC3-like	
scaffold_294	52843	ENSGACG00000001453	LSG LSD	NLRC3-like	
scaffold_461	10414	ENSGACG00000000292	LSG LSD	NLRC3-like	
scaffold_56	1117520	ENSGACG00000002171	Non-LSG LSD	NXPE3	both
scaffold_590	3989	ENSGACG00000000389	Non-LSG LSD	CLEC-like	dup
scaffold_678	985	ENSGACG00000000408	Non-LSG LSD	CLEC-like	del
scaffold_774	8092	ENSGACG00000015668	LSG LSD	NLRC3-like	
scaffold_94	69678	ENSGACG00000011205	LSG LSD	NLRC3-like	

Segmental duplications, stretches greater than 1kb that are similar in sequence (>90%), are known hotspots for structural variations such as CNVs in humans [3], [4], [38], [39]. Not surprisingly, the great majority of genes in stickleback segmental duplications are young genes including 64% of all protein-coding LSDs, therefore most CNV genes in segmental duplications are also young genes **(** **Fig. 4C** **and S13 Figure)**. As for annotated RNA genes, most (59%) reside in segmental duplications, including almost all (88%) of the 174 RNA CNV genes. CNV regions that are outside of segmental duplications are generally gene-poor and are found in fewer individuals (Mann-Whitney test W = 201332, *p* = 7.592e-06, **S14 Figure**). However, CNVs outside of segmental duplications remain enriched with LSGs and LSDs (χ^2^ with Yates correction, *p*<0.0001; **S15 Figure** and **S4 Table**). In other words, the relationship between young genes and CNVs is not solely determined by segmental duplications.

**Figure 5:**
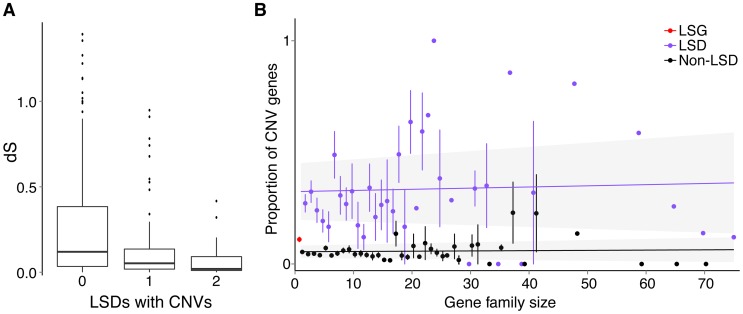
Relationship between gene duplications and CNVs. (**A**) Association between dS of LSD pairs and CNV regions, where dS is a proxy for age since duplication. Boxplots representing the distributions of pairwise dS (synonymous rates of substitution) between LSD pairs where neither (0, n = 129), one (1, n = 59) or both (2, n = 39) genes overlap CNVs. The whiskers represent values beyond the interquartile range (IQR) reaching the highest/lowest value within 1.5 * IQR as implemented in R (ggplot2). (**B**) Gene families and CNVs. Proportion of protein-coding CNV gene members (genes entirely overlapping CNV regions) by gene family size (mean with standard error, plotted with jittering). In red is the mean proportion of LSG singletons (single-gene families – no paralogs), in purple are LSD members and black are non-LSD members. Linear regressions are drawn with the 95% confidence intervals shaded grey.

Lineage-specific genes and duplications identified by our methods may vary in age and could be as old as the most recent common ancestor of sticklebacks and other fishes. To evaluate whether CNV genes are primarily younger versus older lineage-specific genes, we calculated the number of synonymous substitutions per synonymous sites (dS) between LSD gene pairs as a proxy for their age (see **Methods**). We found that LSD pairs in which both are CNV genes have significantly smaller dS than LSD pairs in which only one is a CNV gene (Mann-Whitney test W = 832, *p* = 0.007), which in turn have significantly smaller dS than LSD pairs in which neither is a CNV gene (Mann-Whitney test W = 5155, *p* = 0.004). From these results we infer that younger paralogs are more likely to be CNV genes than older paralogs (**Fig. 5A**). Alternatively, gene conversion between paralogs (see **Methods**) would reduce dS making them appear younger than they are [40], [41]. However, we did not find a positive correlation between CNV genes and gene conversion, but rather a non-significant negative relationship (Fisher's exact test, *p* = 0.070).

Since CNV genes are enriched with young duplicated genes, we tested for an association between the proportion of CNV genes in a gene family and family size. Here the idea was to examine whether larger gene families with many paralogs might be more volatile in terms of copy-number changes through duplications and deletions. We found that CNV genes are not significantly associated with gene family size for both LSDs (ANCOVA, *p* = 0.225) and non-LSDs (ANCOVA, *p* = 0.835), whether or not we include low-frequency (<0.05) CNVs. However, LSDs consistently make up a large proportion of CNV genes across families of all sizes (**Fig. 5B** **)**. We also found that LSG singletons are significantly more often CNV genes than non-LSG singletons (Fisher's exact test, *p*<0.0001), emphasizing the dynamic evolution of very young duplicate genes and potentially novel genes that have arisen by mechanisms other than duplication such as *de novo* gene birth [20].

### Ecological response of CNVs and young genes

**Figure 6:**
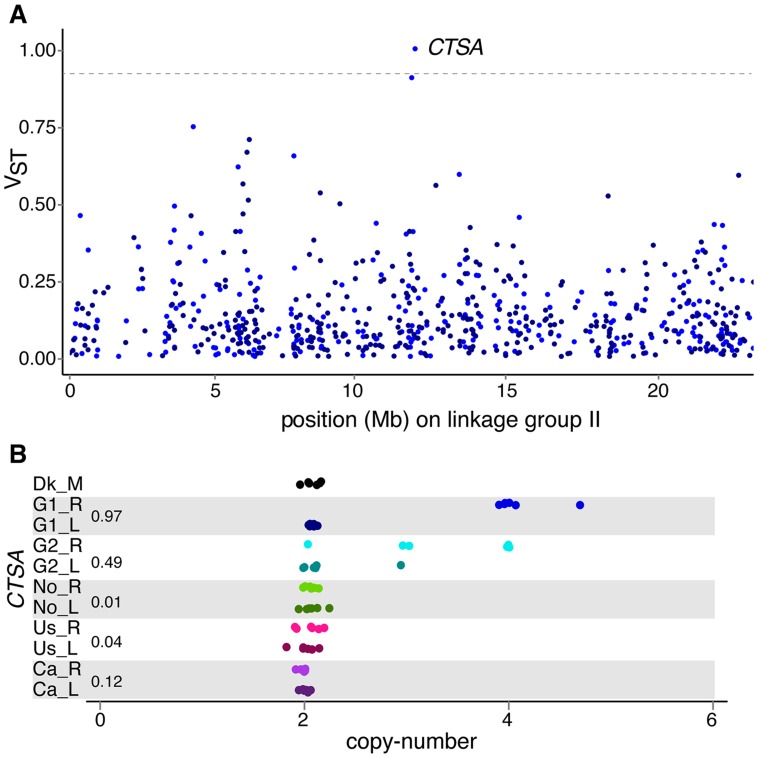
Population differentiation in gene copy-number. (**A**) Population differentiation of genes along linkage group II, measured by the V_ST_ statistic between a German lake-river pair (G1_R vs G1_L). Color shades of dots (that are jittered for ease of visualization) refer to higher average copy-number in river (light) or in lake (dark). The dashed line is drawn at V_ST_ of 0.89. (**B**) The gene with the highest V_ST_ is a lysosome protective protein (*CTSA*) paralog, which has higher copy-numbers in both German river populations compared to their German lake counterparts. Normalized read depth approximating copy-number is plotted across 66 individuals grouped and colored by population, with an expected copy-number of two for a diploid locus. V_ST_ values are reported beside the population pairs.

The comparison of gene copy-numbers between populations can help identify candidate genes under positive selection. We investigated the effects of population differentiation between lake and river ecotypes using the V_ST_ statistic [4], a measure analogous to Wright's F_ST_ on allele frequency differentiation, to identify the genes and exons that have the most differentiated copy-numbers between parapatric lake-river populations. The use of V_ST_ allowed us to incorporate copy-number information from each individual and each locus, including multi-allelic sites, in the evaluation of copy-number variance between populations inhabiting different environments. High V_ST_ indicates larger variance between populations relative to the variance within each population, a pattern consistent with positive selection on copy-number in one or both populations. Although high differentiation can reveal loci under selection, this signal can also be caused by drift (especially from founder effects). Young duplicated genes (LSDs) make up half of the 14 genes with V_ST_ values above the 99.9% percentile of 0.89 (**Table 3**), and have higher average V_ST_ compared to older genes (0.17 versus 0.12, Mann-Whitney test *p*<0.0001). Similar results were found when V_ST_ was calculated across genic exons to evaluate the impact of CNVs on partially duplicated and deleted genes (**S10 Table**). The gene with the highest V_ST_ is a multi-allelic gene similar to the lysosomal protective protein cathepsin A (*CTSA*) that is duplicated in almost every German river individual compared to only one heterozygous German lake individual (**Fig. 6**). Cathepsins are proteases with both stabilizing and activating properties, and are involved in immune response within the *MHC* class II antigen presentation pathway [42], [43]. Other genes with immune functions also have extremely high V_ST_ including *CMKLR1* between the G1 populations, and *PYCARD* between the Ca populations.

**Table 3:** Genes with the most extreme V_ST_ values between paraptric populations, where positive values represent higher copy-numbers in rivers and negative values represent higher copy-numbers in lakes.

Chromosome	Start	Gene ID	Gene Name	Orthology	G1	G2	No	Us	Ca
groupI	19754525	ENSGACG00000013689	PIPOX	Non-LSG Sing	0.01	0.93	0.01	-0.02	0.00
groupII	11731640	ENSGACG00000015895	SLC38A7	Non-LSG Para	0.93	0.53	−0.03	0.50	−0.04
groupII	11736098	ENSGACG00000015897	CTSA	Non-LSG Para	0.97	0.49	−0.01	0.04	−0.12
groupVIII	7921433	ENSGACG00000007122	RAVER2	Non-LSG Para	−0.01	0.00	0.00	−0.93	0.07
groupIX	17101775	ENSGACG00000019395	-	LSG singleton	0.90	0.01	0.30	−0.28	0.00
groupXI	2519758	ENSGACG00000005975	Ferritin M	Non-LSG LSD	0.92	0.19	0.03	−0.01	−0.18
groupXIII	18573306	ENSGACG00000014283	NLRC3-like	Non-LSG LSD	−0.91	0.68	0.12	−0.04	0.34
groupXX	8675984	ENSGACG00000007491	CMKLR1	Non-LSG LSD	−0.93	−0.49	0.29	−0.54	0.04
groupXX	13513401	ENSGACG00000012348	PYCARD1	Non-LSG LSD	0.24	−0.16	−0.28	0.00	0.92
groupXX	13525609	ENSGACG00000012351	PYCARD2	Non-LSG LSD	−0.29	0.04	0.00	0.10	0.90
groupXXI	7994018	ENSGACG00000003408	-	Non-LSG LSD	−0.93	−0.04	0.00	−0.02	0.67
scaffold_54	671409	ENSGACG00000002279	-	LSG singleton	0.89	0.00	−0.02	0.05	−0.79
scaffold_54	1189469	ENSGACG00000002392	HS3ST3L	Non-LSG Para	0.93	−0.01	−0.11	−0.10	−0.25
scaffold_320	2284	ENSGACG00000001341	TRIM35	Non-LSG LSD	−0.08	−0.50	−0.16	0.00	−0.90

Over half of the genes in certain ecologically relevant families are CNVs, including Major Histocompatibility Complex (*MHC*) immune genes (18 out of 32), and groups of G-protein coupled receptor genes including olfactory receptor (*OR*) genes (121 out of 216) and trace amine associated receptors (*TAAR* – 49 out of 64). The average V_ST_ values of immune response genes (0.17) and G-protein coupled receptor genes (0.16) were both significantly higher than the average of all genes (0.14, Mann-Whitney test *p* = 0.0301 and *p* = 0.0037, respectively). *TAARs* had particularly high average V_ST_ (0.24). Consistent with the low amount of CNV sharing across populations, V_ST_ values across the genome were mostly not correlated across the different pairs of lake-river populations (**S16 Figure**), with the highest correlation being between the German groups G1 and G2 with a Pearson's *r* of 0.18.

## Discussion

Like other mutations, most duplications and deletions are likely neutral or detrimental to fitness and the majority of duplicate genes are expected to be lost over time by purifying selection or by drift alone [17], [21]. Assuming that the limited sample sizes per population and the underlying population structure does not adversely skew our allele frequency estimates, most CNVs are present at low frequencies across several diverged populations and habitats, even lower than intergenic SNPs, suggesting that CNVs are generally experiencing purifying selection in nature. Although the majority of CNVs occur at low frequencies, a subset of CNVs was found to reach high frequencies within specific populations, some of which are highly differentiated in copy-numbers between parapatric lake-river populations. CNV genes may reach different frequencies between populations due to selection, but also drift (for example from bottlenecks and founder effects). The observation that CNVs have higher average dN and dN/dS compared to non-CNV genes, coupled with the high incidence of CNV genes in the Selectome database, suggests that a subset of CNV genes may be preserved by positive selection. The wealth of genomic CNVs as standing genetic variation [7] possibly facilitates the rapid adaptation of sticklebacks to various distinct habitats and changing ecology, e.g. infectious diseases. Examples of CNVs associated with environmental change include an expansion of an amylase gene associated with high-starch diets in humans [13], and a recurrent deletion of a gene enhancer associated with an adaptive trait in sticklebacks [44]. It is therefore possible that some genes remain copy-number polymorphic across populations due to selection after habitat or lifestyle diversification.

A quarter of CNVs are shared among distantly related populations, either due to the presence of ancient polymorphisms spanning across continents or to more recent gene flow, or due to recurrent mutations affecting the same genetic region. Recurrent CNVs appear to be common [4] and can be caused by non-allelic homologous recombination hotspots and maintained by gene conversion [45], leading to high turnover rates and the same genes appearing as CNVs across species [46], [47]. This implies that the underlying genomic architecture will provide differential opportunities across the genome for the formation and subsequent preservation of CNVs; CNVs can have higher frequencies due to recurrent mutations in recombination hotspots even if they are under strong purifying selection. However, here we report that CNV sharing is much greater between individuals from the same population or adjacent populations, suggesting that shared ancestry may explain much of the distribution patterns observed, including ancestral CNVs that are shared across continents. Given that Atlantic and Pacific individuals separated at most a few million years ago [48], some ancestral CNV genes may have been maintained even when they are slightly deleterious; genes originating via duplication can take several million years before becoming fixed or pseudogenized [17]. Taken together, we surmise that CNVs occurring across continents are probably a combination of ancestral and recurrent CNVs. In either case, CNVs may have greater opportunities for further persistence when encountering different environments, especially those affecting genes involved in environmental response, e.g. olfactory genes and *MHC* immune genes associated with adaptation to infectious diseases.

Focusing on the impact of CNVs on genes, we found that around 5% of protein-coding genes were completely encompassed in CNVs. There were proportionally twice as many RNA genes as protein-coding genes among CNVs, which may reflect rapid RNA gene turnover through duplications and deletions. We estimate that two individuals differ by over 200 CNV genes, double that which has been predicted in humans [49]. Whereas many of these CNV genes may eventually be lost, this finding is in line with the differing genic content observed between species due to new genes [20]. The substantial amount of genetic structural variation affecting genes reported here has consequences on the interpretations from comparative approaches that use only one reference genome from a species rather than population-wide data, for example to calculate the number of orphan genes in a species. Our results demonstrate the highly dynamic landscape of stickleback genomes and the impressive contribution of genic CNVs to biological diversity across environments in addition to sequence divergence (see companion paper submitted by Feulner *et al.*), supporting the idea that structural changes play an important role during population differentiation.

The earliest stage of gene evolution involves a period of presence-absence polymorphism that can be detected as a CNV. Consistent with this notion, young genes in sticklebacks were enriched with CNVs. This was also independent of gene family size, in which younger gene family members were often CNVs across a range of family sizes. Within LSD pairs, more recently duplicated genes were also more frequently associated with CNVs, similar to recent findings in humans using different methods [19]. These are strong indications that CNVs offer a way of capturing young genes during an early evolutionary stage; as much as 30% of lineage-specific paralogs in genomes may be copy-number polymorphic since their emergence. Alternatively, recurrent duplication and deletion events of genomic regions containing young genes may predispose them to be CNVs. Consistent with this latter scenario, we found that LSGs and LSDs are also enriched in segmental duplications, genomic regions that have strong relationships with structural variations in other animals [1], [3], [39], [46], [50], and that can produce raw material potentiating new genes [51], [52]. Segmental duplications may especially affect the dynamics of RNA genes, since we found a strikingly large overlap between RNA CNV genes and segmental duplications. Together these observations point to a strong association between segmental duplications, CNVs and young genes, confirmed here in natural populations spanning a range of divergence times. Although segmental duplications have been conceptualized as fixed CNVs [53], [54], these are normally annotated based on a single genome and thus a subset of segmental duplications may alternatively represent duplicated regions that are actually segregating CNVs [39]. While there is a noticeable impact of segmental duplications on CNVs, we also found a significant enrichment of CNVs among young genes outside of segmental duplications, suggesting a more complex interplay between young genes and CNVs.

Gene ontology enrichment analysis of CNV genes returned several overrepresented categories with functions involved in environmental response, similar to results in other species [50], [55], [56]. These functions include protein ubiquitination and glycosylation, G-protein coupled receptor signaling pathway and immune activity **(S8 Table)**. The same is true after removing low frequency CNVs occurring in only one individual, as well as for genes that show large copy-number differences among individuals (genes with both deletions and duplications in different individuals). This overrepresentation is driven by LSDs, which make up a significant portion of the gene families in these categories **(S9 Table)**. Although the overrepresentation of these functional categories may hint at an ecological role of many CNVs, it could also indicate that these gene categories are simply under relaxed purifying selection and can better tolerate copy-number fluctuations compared to other functions.

Over half of the genes in some immune and olfaction gene families are CNV genes, and on average they show high population differentiation. The differentiation and population specificity of some of these genes (**S19–S22 Figures** and **S1 Text**) may reflect important differences across habitats such as parasite resistance [57]. In sticklebacks, both *MHC* and olfactory genes are involved in mate choice and ecological diversification among lake and river ecotypes [58], [59]. Immune genes were among the most differentiated CNV genes between parapatric populations, including a gene resembling a lysosomal protease in the two German population pairs (**Fig. 6**). These same populations are known to experience different parasite communities and parasite loads [60], perhaps contributing to differentiating lysosomal proteases that play critical roles in antigen presentation by *MHC* genes [43]. As for olfactory receptor gene families, *TAARs* show particularly high population differentiation on average, and have recently expanded and diversified in teleosts while displaying evolutionary signatures of strong positive selection [61]. It is thus tempting to speculate that some *TAARs* have perhaps emerged and persisted through adaptations to lineage-specific odours [62], [63]. Although we initially hypothesized similar ecological selection gradients among the habitat pairs, we found that differentiated CNV genes were mostly unique to populations rather than ecotypes. Interestingly then, recurrent CNVs originating due to biased mutational mechanisms and the underlying genome architecture generally have different evolutionary trajectories in different populations, possibly due to influences such as local adaptation. However, further experimental and functional studies would be required to formally test the role of candidate CNVs, especially considering that gene ontology assignment, which may be particularly biased against young genes [64], is based on sequence similarity to genes in other vertebrates that may not reflect functions in sticklebacks.

We have combined comparative and population genomics approaches to characterize the evolution of CNVs and of young genes across several distinct environments. Our results demonstrate that most CNV genes in the genome constitute recently emerged genes – as much as a third of all lineage-specific duplications – that are not fixed after millions of years. These young genes often have annotated functions associated with environmental response, some of which potentially play a role in adaptation soon after the colonization of a new habitat. The high prevalence of genes with copy-number differences across populations highlights their contribution in shaping the diversity of stickleback genomes.

## Methods

### Sampling and data processing

Three-spined stickleback fish were caught from five pairs of lakes and rivers in North America and Northern Europe (more details can be found in companion paper submitted by Feulner *et al.*), as well as from a marine population in the North Sea [7] (**S1 Table** and **Fig. 1A**). Muscle tissue from six sampled individuals from each location (aiming for an equal sex ratio) was used for DNA extraction (using a Qiagen DNA Midi Kit following the manufacturer's protocol for high molecular weight DNA) and Illumina sequencing following our previous methods [7]. To capture natural variation present in the wild, we randomly picked individual fish for sequencing, thus without pre-selection of any particular morphological or parasitological characteristics. For each individual, two paired-end libraries (100 bp reads, average insert size of 140 bp and 300 bp) and a mate-pair library (50 bp reads, average insert gap of 3 kb) was produced, achieving an average depth of coverage of 26x (**Table 1** and **S1 Table**). Raw sequence data was processed and filtered following previous procedures [7] and mapped against the three-spined stickleback reference genome [30] from Ensembl version 68 [65]. The use of six individuals per population was chosen to have a balance between a wide geographical range of samples, an adequate number of individuals represented per population, and sufficient depth of sequence coverage per genome to reliably call SNPs and CNVs. Raw sequence reads from the 66 genomes are accessible under the European Nucleotide Archive study accession ERP004574.

We called CNVs for each individual separately based on the combination of signals from a read depth approach (CNVnator), paired-end approach (Breakdancer and delly) and split-reads approach (Pindel). Deletions and duplications were defined as the decrease or increase of copy-number relative to the reference genome, wherein duplications in sequenced genomes could actually be deletions in the reference genome, but this occurrence may be low [6], [49]. This also means that we considered large insertions and deletions to be CNVs, even though the mechanism of formation is different from duplications. The read depth software CNVnator [66] was used to evaluate CNVs along the genome in 500 bp windows. The paired-end mapping approach implemented in Breakdancer [67] made use of paired-end data (with the longer insert size of ∼300 bp) and mate-pair libraries that were processed separately, and only using uniquely mapped reads. We kept the default cut-off for the insert size deviation, but increased the required read pairs to establish a connection to 4. Haploid sequence coverage was adjusted when calling variants on all 66 libraries combined. We also used delly and duppy [68] to infer CNVs with the default parameters. The split-reads approach implemented in Pindel [69] was used with the default settings on all 66 paired-end libraries together.

For each individual, deletions and duplications that overlapped (more than 50% of their length) within the same CNV calling approach were merged together and then compared across approaches; we only kept deletion and duplication calls from CNVnator that overlapped (more than 50% of their length) with calls from at least one other approach. In an effort to reduce the impact of the reference genome when calling CNVs, deletions and duplications found in all individuals were excluded as well as calls that overlapped (more than 50% of their length) with repeat-masked regions from the Ensembl annotations (version 68). We also removed short CNVs (<500 bp) and those found on the sex chromosome that are influenced by male hemizygosity. Deletions and duplications were then compared across individuals to evaluate CNV regions; contiguous chromosomal stretches encompassed by CNVs. All copy-number variations are reported in **S11 Table**. The visualization of CNV clustering based on shared variants was performed using principal components analysis as implemented by prcomp() in R [70].

Complete overlap of genes and genic regions was given a 5% leeway due to imperfect breakpoint calling in CNVnator (>95% of the gene length covered by a CNV was considered a CNV gene). Gene information was acquired using EnsemblCompara version 68 [65], [71]. Read depth averages were used to infer CNV genotypes for each gene and were retrieved via CNVnator using the default genotyping output. For each individual, gene read depth was normalized by the median and then centered around two (to represent diploids). Differences between individuals were evaluated based on this normalized read depth. Gene loss was inferred when the normalized read depth average fell below 0.25, effectively 8-fold lower coverage than expected. For each gene (or exon) loss, at least one individual had a normalized read depth average above 2 to curtail regional mapping biases. These inferences are made using the average read depth across a region, and thus ignore heterogeneity within the region. Shell and perl scripts were used with BEDTools [72] to evaluate the distribution of CNVs, overlap of gene regions, and variance in read depth between populations. We evaluated V_ST_ between parapatric lake-river population pairs for each gene following the definition in [4].

To identify CNV sharing across individuals, we required a reciprocal overlap of CNVs (more than 50% of their lengths) between individuals. For the evaluation of CNV occurrence across individuals, we tabulated presence and absence of each CNV for each individual. In addition, CNV allele frequencies (determined based on the copy-number on each chromosome) were calculated (and polarized) compared to the reference genome, such that each reference locus was considered to be in a diploid state with two single-copy alleles. Copy-number was inferred for each CNV and gene based on normalized read depth using CNVnator, in which read depth at each locus was normalized by the median across all individuals, centered around two (diploid), and rounded to the closest integer as a proxy for copy-number. We then retained all putative bi-allelic CNV loci – those loci with either two or three different genotypes that could be explained by a combination of zero deletion alleles, one deletion allele and two deletion alleles, or by a combination of zero duplication alleles, one duplication allele and two duplication alleles. In other words, genotypes were assumed to only differ by one copy per allele. Allele frequencies and genotypes were inferred from 1492 putative bi-allelic CNVs (38% of CNVs) and used to determine allele frequency spectra.

A phylogeographic analysis on the genomic data was performed using the Neighbor-net method [73] as implemented in SPLITSTREE4 [74] and using default settings, except that ambiguous states were averaged over all possible resolutions. A network is preferable over a tree because of the potential gene flow and shared ancestral polymorphisms across populations. The phylogenomic network (**Fig. 1A**) was created using 50,000 randomly selected high-quality single nucleotide polymorphisms (SNPs) that were outside of repeat-masked and CNV regions, as well as at least 10 bp from any indels. These same SNPs were also used to compare with the allele frequency spectrum of CNVs. After the initial processing and filtering of the raw sequencing data following the procedures stated above in [7], SNPs and indels were called with GATKv1.6 [75] using concordant SNP calls from SAMtools v0.1.18 [76] for variant recalibration. Phasing and imputation was performed with BEAGLE v3.1 [77]. VCFtools v0.1.11 [78] was utilized for processing genotypes and allele frequency spectra.

To test for the influence of selection on the allele frequency spectrum of CNVs, we used a Poisson Random Field approach implemented in prfreq [35], which evaluates the expected allele frequency distributions given different demographic and selection models. We compared the estimates of the scaled selection coefficient γ of CNVs (both deletions and duplications) with intergenic SNPs, by fitting the “single point mass” model over a range of γ (between −20 and 10). Estimates were taken modeling a stationary population, under the assumption that demographics affect intergenic SNPs and CNVs in a similar fashion.

### Assembly of unmapped reads

We extracted all unmapped reads for each individual and performed a *de novo* assembly with Velvet [79], using a k-mer of 21 bp, a minimum contig length of 99 bp and coverage cutoff of 4 reads. The resulting contigs for each individual were then clustered and assembled using CAP3 [80] with a 97 percent identity. This returned 161,780 contigs that were made up of sequences from at least two individuals, and had an average length of 456 bp (max  = 12,780 bp). BLASTX [81] was then performed on the assembly output versus the nr protein database, and hits below 1e-05 were kept as putative orthologs. BLASTX was also run on the three-spined stickleback protein database (Ensembl version 68) and BLAT was run on the three-spined stickleback reference genome (**S17 Figure**). Blast2GO [82] was used to annotate the hits. In total we found over 100 putative genes that were not found in the reference genome (**S6 Table** and **S1 Text**), in addition to another 100 genes absent from the Blast2GO results but annotated through BLASTX.

### Gene annotations and lineage-specific genes and duplications

Gene annotations were taken from Ensembl, including protein-coding genes and RNA genes: ribosomal RNA (rRNA), micro RNA (miRNA), small nucleolar RNA (snoRNA) and small nuclear RNA (snRNA). The annotations of olfactory receptor (*OR*) genes were taken from multiple sources, including 122 general odorant receptors [63], six *V1R* genes [83], 24 *V2R* genes [63] and 64 *TAAR* genes [62]. Gene ontology (GO) terms were inferred using the Ensembl annotations (version 68), and significant enrichment of GO terms was acquired using the topGO weight algorithm and determined by FDR adjusted p-values to help correct for multiple testing [84].

We categorized genes as LSGs and LSDs using orthology and paralogy relationships from EnsemblCompara version 68 [65], [71]. BLASTX searches versus the nr database returns some hits between some of our identified LSG singletons (34%) and most LSG LSDs (74%), suggesting that these may not have strictly originated in sticklebacks but alternatively have substantially diverged from orthologs while retaining enough similarities to be recognized as potential homologs (**S12 Table**). Nevertheless, LSGs and LSDs have properties associated with young genes (**S1 Text**). It is also possible that some of these genes are on their way to pseudogenization, although we found that 48% have expression information in EST databases. Expression data was collected from the EST database on NCBI, mapped against the stickleback genome, and expression profiles were examined across gene categories. Segmental duplications were downloaded from http://humanparalogy.gs.washington.edu/stickleback/data/.

We used permutations to test for overrepresentation or underrepresentation of genes among CNVs. We permuted (n = 1000) the CNV data to randomly select across each chromosome the same number (and length) of regions as deletions and duplications. We then evaluated the overlap of genes based on our observed data versus the distribution of this random sampling. See **S1 Text** for detailed results.

### Validation of CNV calls

We validated some CNV calls by PCR and quantitative PCR. We designed primers in and surrounding CNV calls to (1) genotype genic deletions in individuals using PCR and gel assays, and to (2) measure relative copy-numbers between individuals using qPCR. This allowed us to evaluate concordance for CNV loci, genotypes and read depth. Overall, we validated 96% (22/23) of the CNV loci, in terms of CNV presence, with lower concordance (88%) for genotypes, most of which were called heterozygotes by PCR. For the same individuals for which genome sequencing was performed, DNA was re-extracted using the DNeasy 96 Qiagen extraction kit (Hilden Germany) and adjusted the concentration to 10 ng/uL. For six deletions affecting between 3 to 42 individuals and showing clear genotype distinctions between a homozygote deletion, heterozygote deletion and no deletion in our sequencing data, we performed a gel assay in all 66 individuals scoring presence or absence based on different primer pairs; one spanning the complete CNV deletion and the other falling within the deletion. In this way, primer pairs were designed such that a positive signal in both pairs would return a heterozygote (primer sequences can be found in **S13 Table**). Some combinations in certain individuals gave inconclusive signals (in case one of the primer pairs failed), but in total we confirmed the 6 deletions with 88% (284/321) concordant presence/absence genotypes across individuals. Secondly, a qPCR assay was performed on 17 loci, selected to have a range of low to high CNV allele frequencies, and a range of low to high copy-numbers, to evaluate concordance of relative read depth. Using a standard housekeeping gene (ribosomal protein *L13*) as an internal control reference, we followed a modified version of the comparative C_T_ method [85]–[87], in which the internal control was also used as the calibrator and the ΔC_T_ values were used to directly compare relative copy-number between individuals. We estimated absolute copy-number assuming that the reference gene is diploid in all individuals (the CNVnator read depth across the 66 individuals ranged from 1.78 to 2.35). Concordance of read depth signal was evaluated using the Pearson correlation statistic (as implemented in R) on the relative copy-number between individuals evaluated from the read depth analysis in CNVnator versus the qPCR analysis. This returned 94% (16/17) concordant relative read depth calls after Bonferroni correction (100% with FDR q-values <0.05) and an average correlation of 0.78, including a high concordance for the gene with the highest V_ST_ (**S2 Figure**).

### Molecular evolution

We evaluated the relationship between the age of paralog pairs and CNVs. A proxy for relative age of paralogs was calculated by first aligning LSD pairs (n = 350) using MACSE [88], and then by estimating pairwise synonymous substitutions per synonymous site (dS) using a maximum likelihood approach implemented in PAML 4.5 [89]. Genes with dS greater than 1.5 (n = 123) were excluded from analysis due to poor alignment or substitution saturation. 89% of these pairs (dS >1.5) encountered no CNVs. We performed comparative genomic analyses using sequences from nine-spined sticklebacks to evaluate molecular rates of evolution (dN, dS and dN/dS) among genes. Raw nine-spined stickleback 454 sequences from Guo *et al.* [90] were assembled with iAssembler v1.3.0 [91] using a strict 100% identity threshold and resulting contigs were mapped to three-spined stickleback gene transcripts from Ensembl version 68 using BLAST. Top hits with an e-value threshold of 1e-4, score above 90 and >90% percent identity were used in a sequence alignment with MACSE. The longest transcript for each gene was used as the representative gene alignment. Pairwise rates of molecular evolution were conducted using PAML 4.5, and threshold values for dN were set to a maximum of 10 and dS to a maximum of 1.0.

Evidence for gene conversion was identified using default settings in GENECONV v.1.81 [92], with the exception of listing pairwise p-values (-ListPair) and to include monomorphic sites (-Include_monosites). After excluding gene pairs with dS <0.05 due to difficulties of detecting gene conversion events with highly similar sequences [93], LSD pairs with CNVs still did not show a significant association with gene conversion (two-sided Fisher's exact test, *p* = 1), and still have significantly smaller dS than LSD pairs without CNV regions (Mann-Whitney, W = 2388, *p* = 0.0105).

### Ethics statement

This study was performed according to the requirements of the German Protection of Animals Act (Tierschutzgesetz) and was approved by the 'Ministry of Energy, Agriculture, the Environment and Rural Areas' of the state of Schleswig-Holstein, Germany (reference number: V 312-72241.123-34). Wild sticklebacks were caught using minnow traps or hand nets. Before dissection the fish were anesthetized with MS222 and sacrificed by an incision into the brain followed by immediate decapitation, and every effort was made to minimize suffering. No further animal ethics committee approval was needed. The species used in this study are not endangered or protected.

## Supporting Information

Figure S1 Matrix of CNV similarity between the 66 genomes. Matrix of similarity between the 66 genomes for presence of (**A**) deletions and (**B**) duplications. Individuals are clustered by population, in which colors at the top match colors in **Fig. 1**. Heat map shows more similar comparisons (in terms of presence/absence of CNVs) with darker blue. Individuals share more similarity within populations, and within continent.
(TIF)

Figure S2 CNV validation. CNV validation. Concordance of read depth calculated using CNVnator and qPCR of the gene showing the highest V_ST_ (*CTSA* in **Fig. 6**). Each dot represents an individual, the red line shows a 1∶1 ratio, and the blue line is the regression line (Pearson correlation  = 0.88, p = 7.55e-15).
(TIF)

Figure S3 Frequency distribution of CNVs and sharing among individuals. **A)** Presence of CNVs among individuals. The proportion of CNVs (deletions in white, duplications in black) that are shared between individuals. **B)** Allele frequency spectrum of bi-allelic CNVs across all 66 individuals, showing deletions (white) and duplications (black) occurring at lower frequencies than intergenic SNPs (grey).
(TIF)

Figure S4 Proportion of CNVs shared across individuals depending on gene overlap. The proportion of CNVs shared across individuals depending on gene overlap. For both **(A)** deletions and **(B)** duplications, the proportion of CNVs are shown for CNVs overlapping intergenic regions, partial genes, one full gene, or multiple full genes. CNVs overlapping full genes and multiple genes are generally found in more individuals.
(TIF)

Figure S5 Frequency distribution of CNVs depending on gene overlap. The allele frequency spectrum of bi-allelic CNVs across 66 individuals depending on genic overlap for both **(A)** deletions and **(B)** duplications. CNVs overlapping no genes (white), partially overlapping a gene (black) and fully overlapping a gene (grey). CNVs fully overlapping a gene are found at higher frequencies.
(TIF)

Figure S6 Average proportion of shared CNVs. The average proportion of shared CNVs between individuals across mutually exclusive groups for (**A**) deletions and (**B**) duplications. These figures are analogous to **Fig. 2F** in the main text. The proportion of CNV sharing was calculated for four groups: “Between Continents” is sharing across individuals from different continents, “Between Countries” is sharing across individuals from different countries within the same continent, “Between Populations” is sharing across individuals from different populations from the same country, and “Within Populations” is sharing across individuals from the same population.
(TIF)

Figure S7 Proportion of genes fully encompassed in CNVs. Proportion of autosomal genes by biotype that are fully encompassed in CNVs.
(TIF)

Figure S8 Frequency and sharing of CNV genes across all individuals. **(A)** Presence of CNV genes among individuals. The proportion of CNV genes (deletions in white, duplications in black) that are shared between individuals **(B)** Allele frequency spectrum of bi-allelic CNV genes across all 66 individuals, showing most deletions (white) and duplications (black) occurring at very low frequencies. (analogous to **S3 Figure**).
(TIF)

Figure S9 CNV genes shared across populations. Occurrence of CNV genes across populations. The proportion of CNV genes (deletions in white, duplications in black) that are shared between individuals. This figure is analogous to **Fig. 2C** in the main text.
(TIF)

Figure S10 Proportion of CNV genes that are specific to groups of individuals. Proportion of CNV genes that are specific (private) to groups of individuals spanning different scales of divergence. Mutually exclusive groups for which CNV genes are private include: those occurring across continents (Ancestral/Recurrent), those specific to a continent but shared across populations from different countries (Continent), those specific to a country but shared across populations within a country (Country), and those only found in one population (Population). This figure is analogous to **Fig. 2D** in the main text.
(TIF)

Figure S11 Frequency and sharing of CNV genes across individuals within populations. **(A)** Allele frequency spectrum of non-reference alleles from bi-allelic CNV genes across 12 individuals from each population represented as boxplots (analogous to **Fig. 2B** in the main text). **(B)** Boxplots showing the proportion of CNV genes shared across individuals within populations (from a single individual up to all 6 individuals, analogous to **Fig. 2E** in the main text)
(TIF)

Figure S12 Average proportion of shared CNV genes. Average proportion of shared CNV genes between individuals across mutually exclusive groups. The proportion of CNV gene sharing was calculated for four groups: “Between Continents” is sharing across individuals from different continents, “Between Countries” is sharing across individuals from different countries within the same continent, “Between Populations” is sharing across individuals from different populations from the same country, and “Within Populations” is sharing across individuals from the same population. This figure is analogous to **Fig. 2F** in the main text.
(TIF)

Figure S13 Distribution and density of CNVs across the genome. Circos plot showing the distribution and density of CNVs across the genome, including **(A)** the 20 autosomes (total of 380.5 Mbp) and **(B)** unplaced scaffolds (total of 60.7 Mbp). Circular tracks from the exterior represent the following features: **A** - LSG singletons (in red) and LSDs (in purple), **B** – density of segmental duplications (in black), **C** – CNV density (in green), and **D** - divided into duplications (in blue facing outwards) and deletions (in red facing inwards), **E** – lost genes.
(TIF)

Figure S14 CNV genes shared across individuals and segmental duplication overlap. CNV genes shared across individuals depending on segmental duplication (SD) overlap. For both (**A**) deletions and (**B**) duplications, the proportion of CNV genes shared in segmental duplications (black) or outside of segmental duplications (white) is shown. Genes outside of segmental duplications are generally found in fewer individuals.
(TIF)

Figure S15 Proportion of genes overlapping CNVs and segmental duplication overlap. Proportion of genes overlapping CNVs (**A**) in segmental duplications (SDs) and (**B**) outside of SDs. Analogous to **Fig. 4B** in the main text. The proportion of genes (both protein-coding and RNA) from each gene category (non-LSG singletons, non-LSG paralogs, non-LSG LSD, LSG LSD, LSG singletons and all genes in total) that is completely encompassed within CNVs (deletions in white, duplications in black, both deletion and duplication in stripes).
(TIF)

Figure S16 V_ST_ scan across the genome in five parapatric populations. V_ST_ scan across the genome performed on every gene for each of 5 river-lake population pairs. The five pairs from top to bottom are G1, G2, No, Us and Ca, and the horizontal line represents the 99^th^ quantile. Autosomes are alternately shaded black or grey, starting from chromosome one to 21 (skipping the sex chromosome 19), with the last section in black representing the unplaced scaffolds.
(TIF)

Figure S17 Overlap of BLAST hits of unmapped contigs. Overlap of BLAST hits of the unmapped contigs versus the nr database (nr), stickleback genome (*G.aculeatus* DNA) and stickleback proteins (*G.aculeatus* proteins). Only a very small proportion of contigs hit non-stickleback sequences (803).
(TIF)

Figure S18 Structural properties of genes.Structural properties of genes across categories. Lineage-specific gene categories (LSG LSD and LSG singleton) have (**A**) shorter gene lengths and (**B**) fewer exons.
(TIF)

Figure S19 CNV of immune genes. Copy-number variation of immune related genes across populations. Normalized read depth approximating gene copy-number is plotted across 66 individuals grouped and colored by population following the format of **Fig. 6**.
(TIF)

Figure S20 CNV of olfaction genes. Copy-number variation of olfactory related genes across populations. Normalized read depth approximating gene copy-number is plotted across 66 individuals grouped and colored by population following the format of **Fig. 6**.
(TIF)

Figure S21 CNV of group-specific gene expansions and losses. Copy-number variation of group-specific gene expansions and losses. Normalized read depth approximating gene copy-number is plotted across 66 individuals grouped and colored by population following the format of **Fig. 6**.
(TIF)

Figure S22 High CNV differences between groups. High copy-number variation of group-specific gene expansions and losses. Normalized read depth approximating gene copy-number is plotted across 66 individuals grouped and colored by population following the format of **Fig. 6**.
(TIF)

Table S1 Summary of sequencing statistics and sample information.
(PDF)

Table S2 CNV genotypes for bi-allelic CNVs. Genotype codes are 0 for homozygous deletion, 1 for heterozygous deletion, 2 for wild type diploid, 3 for heterozygous duplication and 4 for homozygous duplication. Frequency represents the respective frequencies of each genotype. The number of CNV alleles, homozygous genotypes with the CNV allele, and heterozygous genotypes with the CNV allele are shown for all 66 individuals (Total), and for each population.
(PDF)

Table S3 CNVs that are private to a population or region and found in each individual from that population or region. Asterisk indicates fixed CNVs in the population or region.
(PDF)

Table S4 Lineage-specific nuclear genes and other biotypes in the three-spined stickleback genome based on annotations from Ensembl v68. Columns represent total numbers of genes in each category across the autosomal genome, and the number and proportions of genes in each category that are found partially or completely in copy-number variation regions (CNVRs), including deletions (DEL) and duplications (DUP). The numbers and proportion of genes fully overlapping CNVRs that do not overlap segmental duplications (SDs) are reported. The number of gene losses are also reported.
(PDF)

Table S5 Putatively lost genes (with an average normalized read depth below 0.25), and number of individuals from each population with the putative loss. Information includes linkage group (LG), start and end positions, Ensembl gene ID, biotype, category based on orthology and paralogy (see **Fig. 4**), the number of individuals (Ind) with the gene loss polymorphism, and the number of individuals from each population (see **Fig. 1**) with the gene loss polymorphism.
(PDF)

Table S6 Annotated gene names from the top nr BLASTx hits of unmapped reads using Blast2GO, and also from BLASTx for genes without blast2GO annotations.
(PDF)

Table S7 Number of CNVs, CNV genes and gene losses for each population and individual within the population.
(PDF)

Table S8 Gene ontology (GO) enrichment analysis among CNV genes (genes that are entirely overlapping CNVRs). The number of genes from each GO category and the number of genes completely overlapping CNVRs, including deletions and duplications reported separately, with significantly overrepresented categories in bold.
(PDF)

Table S9 Gene ontology (GO) enrichment analysis among stickleback LSDs.
(PDF)

Table S10 Exons with extreme V_ST_ values (>0.89) between lake-river population pairs, where positive values represent higher copy-numbers in rivers and negative values represent higher copy-numbers in lakes.
(PDF)

Table S11 CNVs and CNV genes separated into (a) deletion calls and duplication calls, (b) CNV gene deletions and CNV gene duplications.
(PDF)

Table S12 Number and proportion of autosomal genes across different categories with no significant BLAST protein hits in other fish from Ensembl v68.
(PDF)

Table S13 Primers used for validating CNVs.
(PDF)

Table S14 Median dN, dS and dN/dS of genes using pairwise statistics with nine-spined stickleback orthologs.
(PDF)

Table S15 Genes consistent with positive selection with pairwise dN/dS >1 between three-spined stickleback and nine-spined stickleback. CDS represents protein-coding length, Length is the total gene length, and Cat is one of 5 gene categories: Non-LSG singletons, Non-LSG paralogs, Non-LSG LSD, LSG LSD and LSG singletons.
(PDF)

Text S1 Supporting text including methods and analyses.
(PDF)
