# Supplementary figures and images for: Extensive Copy-Number Variation of Young Genes across Stickleback Populations
Source: PLoS Genet. 2014 Dec 4;10(12):e1004830. doi: 10.1371/journal.pgen.1004830 (PMC4256280; doi:10.1371/journal.pgen.1004830)

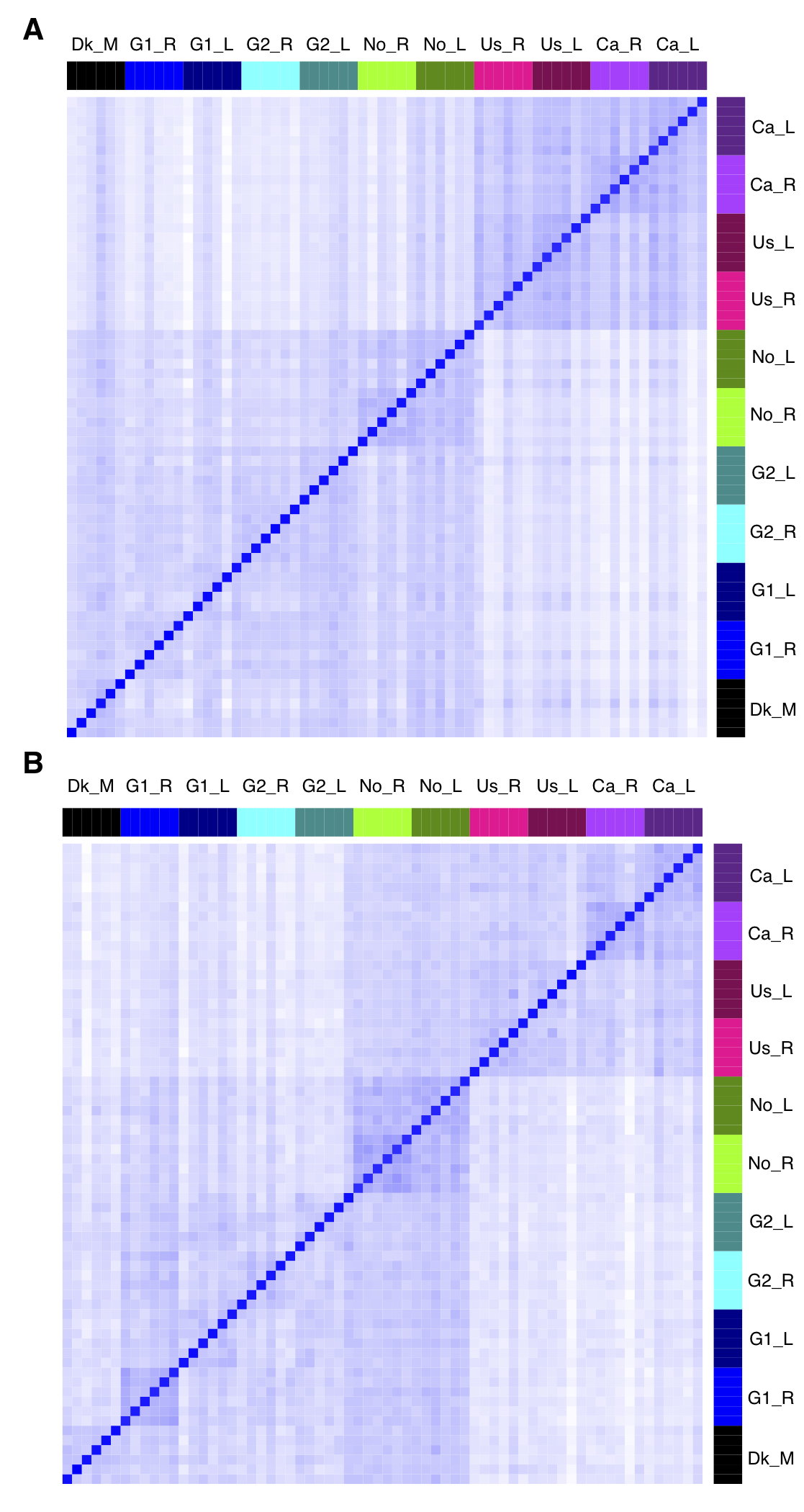

Supplement: Figure S1 — Matrix of CNV similarity between the 66 genomes. Matrix of similarity between the 66 genomes for presence of (A) deletions and (B) duplications. Individuals are clustered by population, in which colors at the top match colors in Fig. 1 . Heat map shows more similar comparisons (in terms of presence/absence of CNVs) with darker blue. Individuals share more similarity within populations, and within continent. (TIF) [file pgen.1004830.s001.tif]

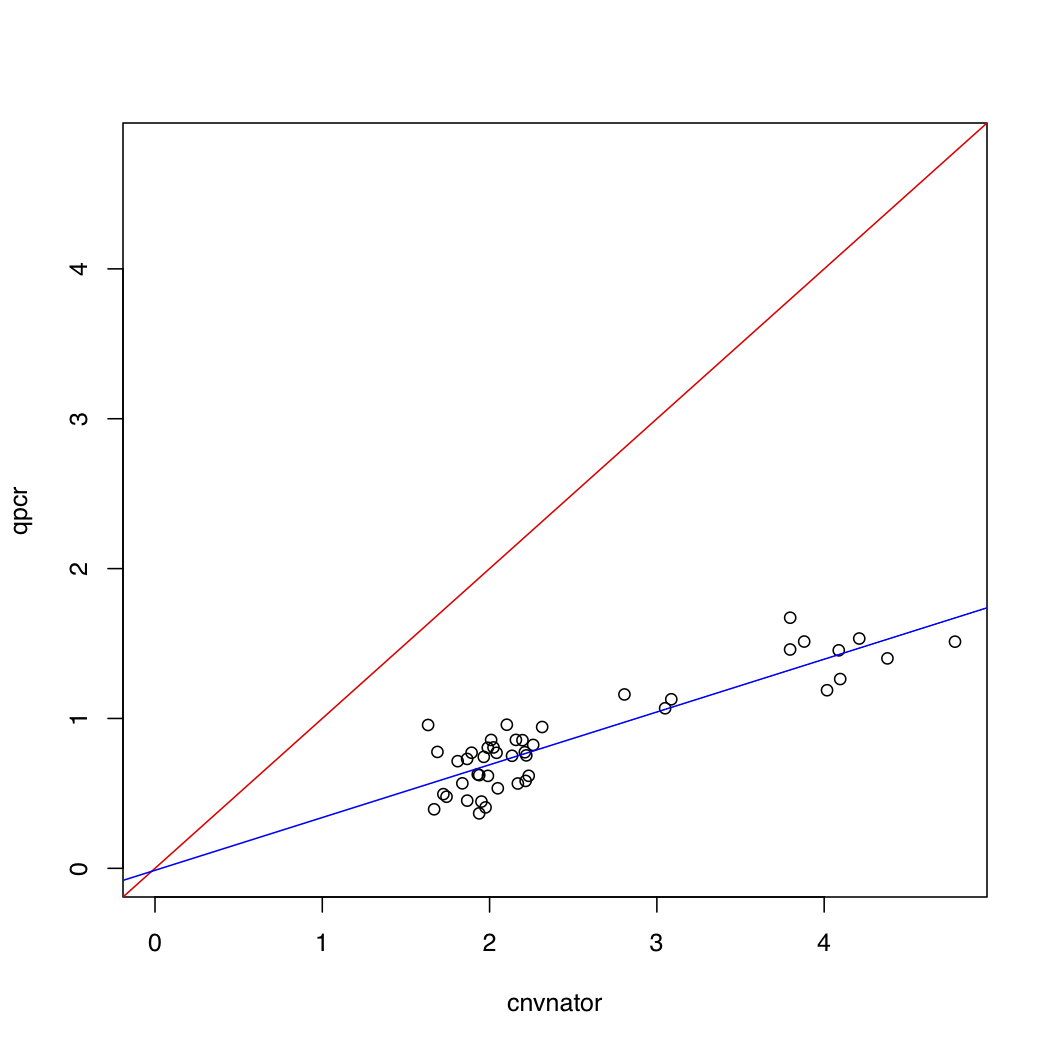

Supplement: Figure S2 — CNV validation. CNV validation. Concordance of read depth calculated using CNVnator and qPCR of the gene showing the highest VST (CTSA in Fig. 6 ). Each dot represents an individual, the red line shows a 1∶1 ratio, and the blue line is the regression line (Pearson correlation = 0.88, p = 7.55e-15). (TIF) [file pgen.1004830.s002.tif]

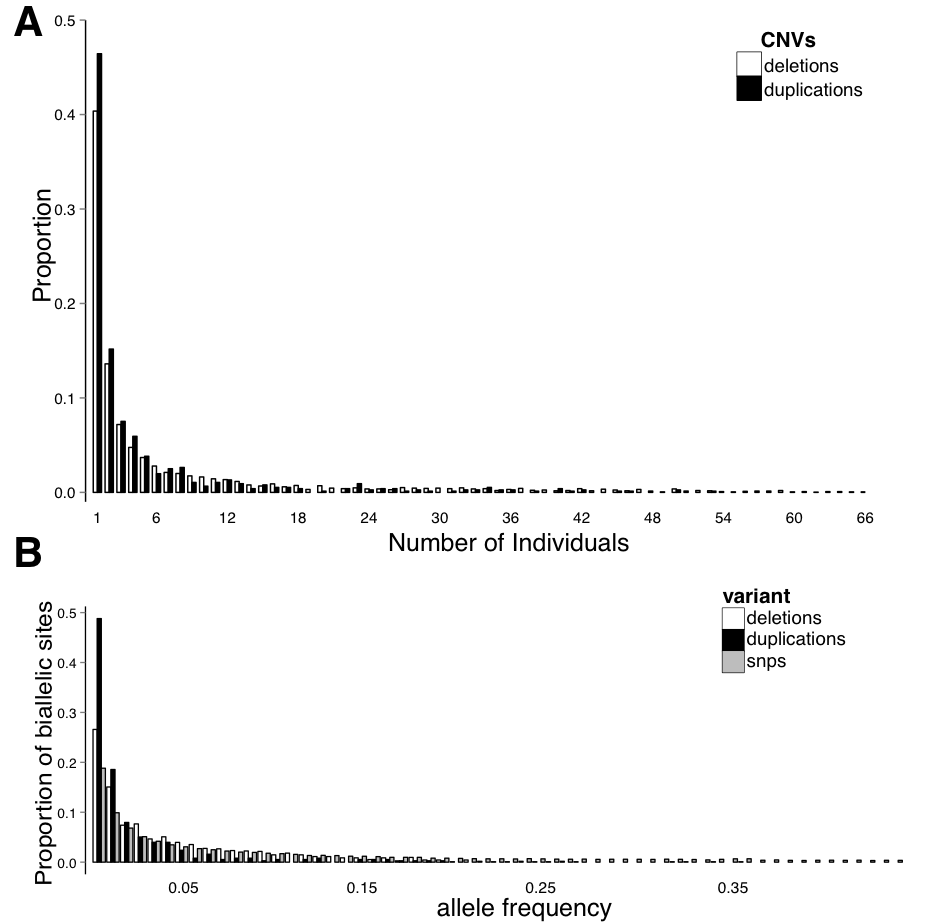

Supplement: Figure S3 — Frequency distribution of CNVs and sharing among individuals. A) Presence of CNVs among individuals. The proportion of CNVs (deletions in white, duplications in black) that are shared between individuals. B) Allele frequency spectrum of bi-allelic CNVs across all 66 individuals, showing deletions (white) and duplications (black) occurring at lower frequencies than intergenic SNPs (grey). (TIF) [file pgen.1004830.s003.tif]

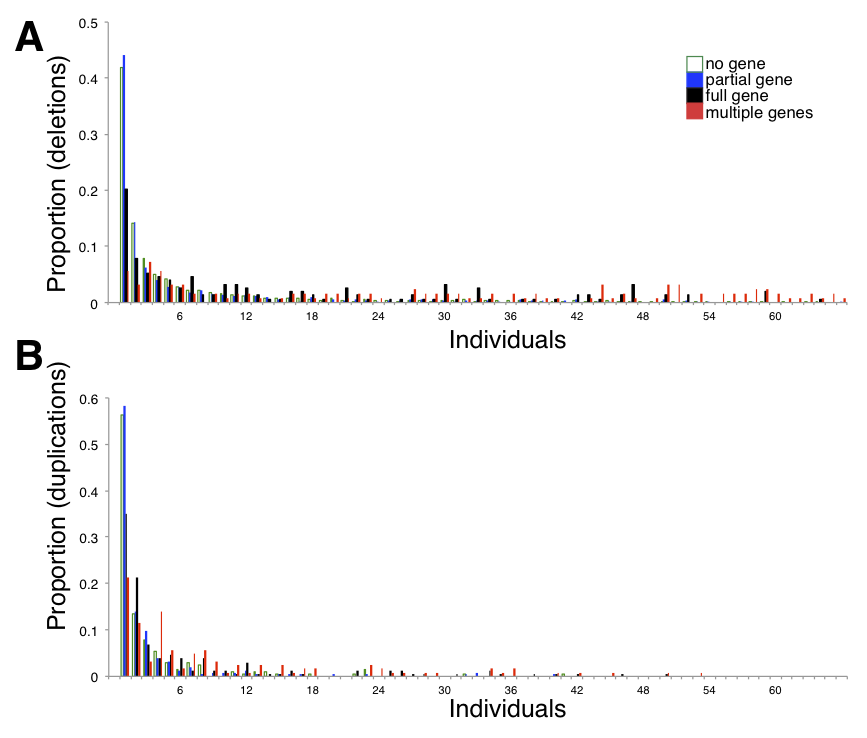

Supplement: Figure S4 — Proportion of CNVs shared across individuals depending on gene overlap. The proportion of CNVs shared across individuals depending on gene overlap. For both (A) deletions and (B) duplications, the proportion of CNVs are shown for CNVs overlapping intergenic regions, partial genes, one full gene, or multiple full genes. CNVs overlapping full genes and multiple genes are generally found in more individuals. (TIF) [file pgen.1004830.s004.tif]

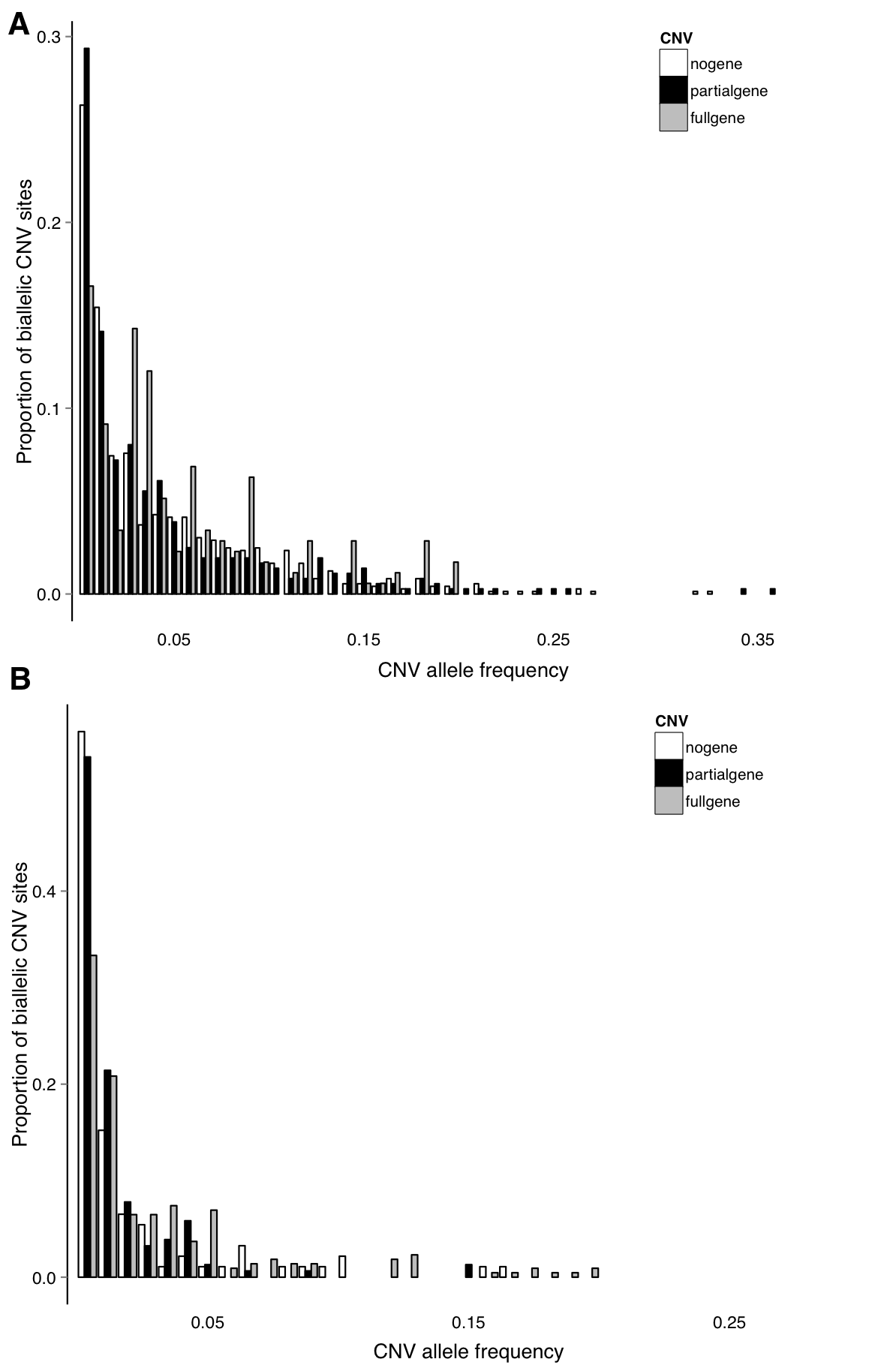

Supplement: Figure S5 — Frequency distribution of CNVs depending on gene overlap. The allele frequency spectrum of bi-allelic CNVs across 66 individuals depending on genic overlap for both (A) deletions and (B) duplications. CNVs overlapping no genes (white), partially overlapping a gene (black) and fully overlapping a gene (grey). CNVs fully overlapping a gene are found at higher frequencies. (TIF) [file pgen.1004830.s005.tif]

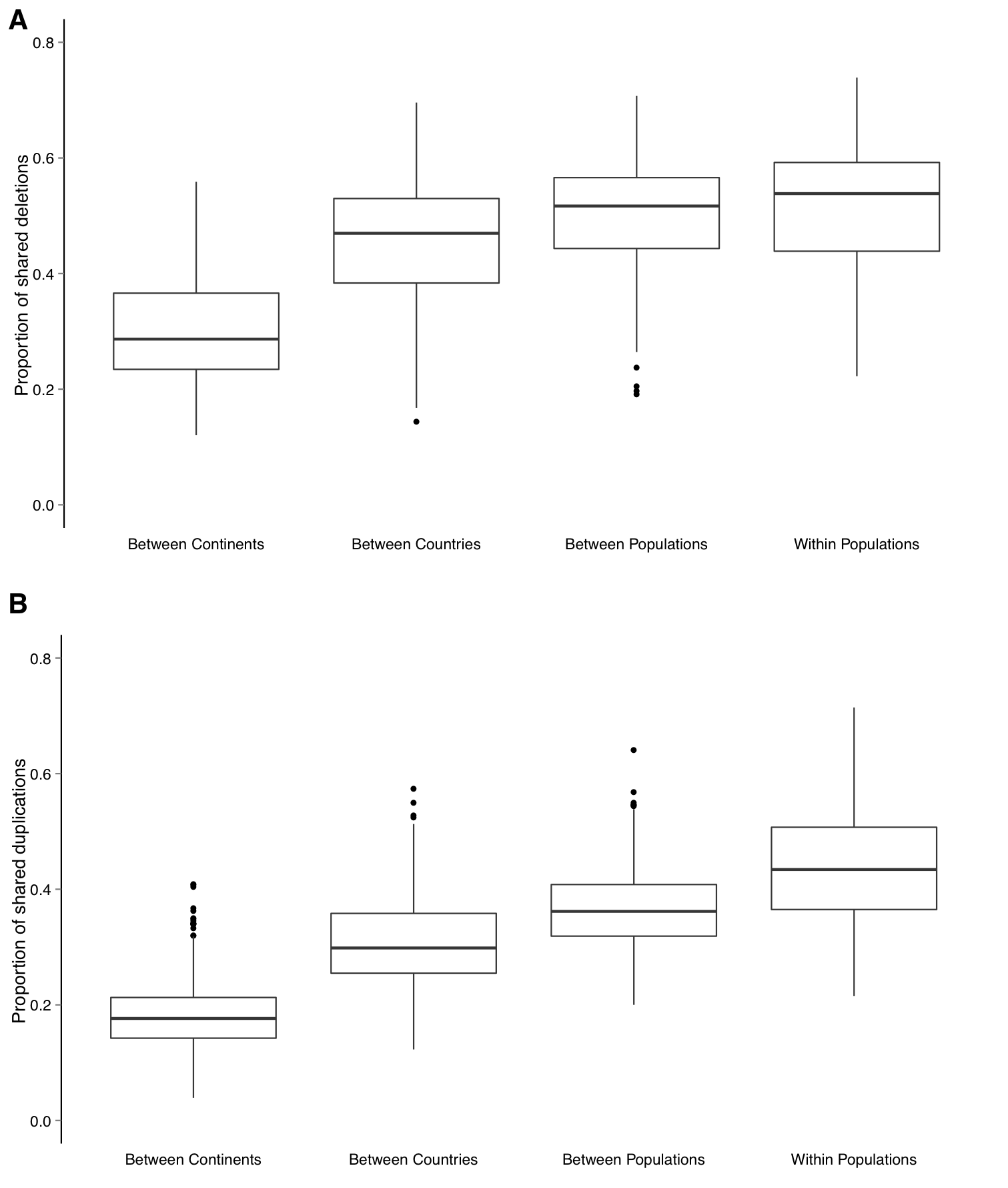

Supplement: Figure S6 — Average proportion of shared CNVs. The average proportion of shared CNVs between individuals across mutually exclusive groups for (A) deletions and (B) duplications. These figures are analogous to Fig. 2F in the main text. The proportion of CNV sharing was calculated for four groups: “Between Continents” is sharing across individuals from different continents, “Between Countries” is sharing across individuals from different countries within the same continent, “Between Populations” is sharing across individuals from different populations from the same country, and “Within Populations” is sharing across individuals from the same population. (TIF) [file pgen.1004830.s006.tif]

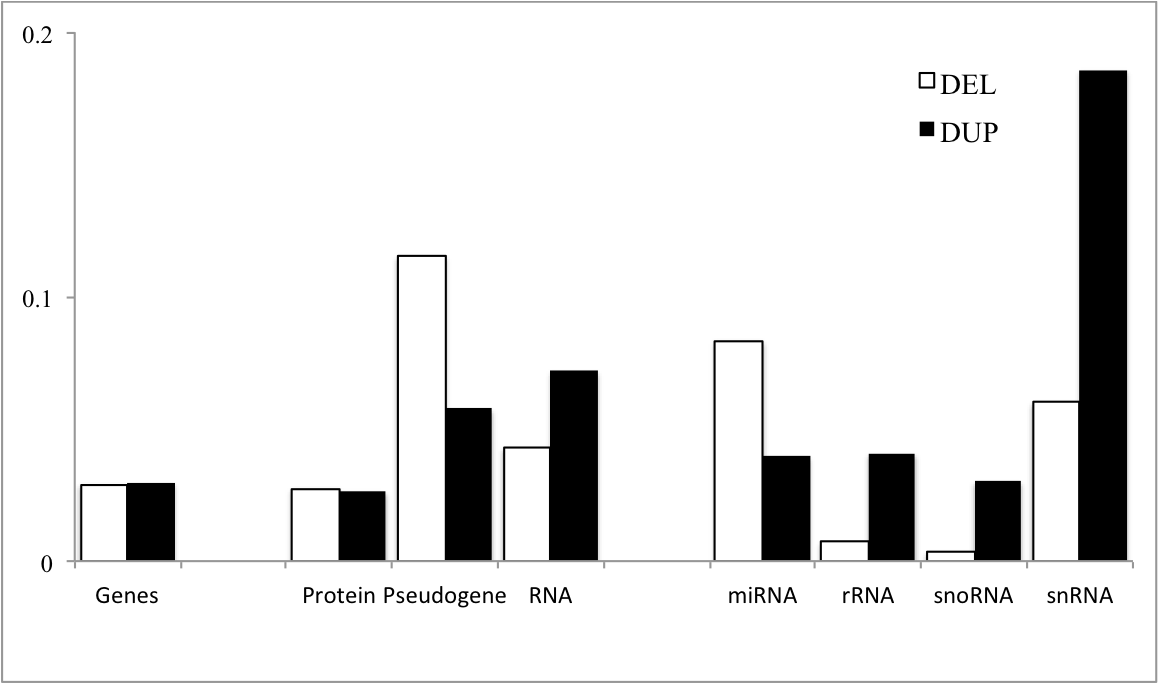

Supplement: Figure S7 — Proportion of genes fully encompassed in CNVs. Proportion of autosomal genes by biotype that are fully encompassed in CNVs. (TIF) [file pgen.1004830.s007.tif]

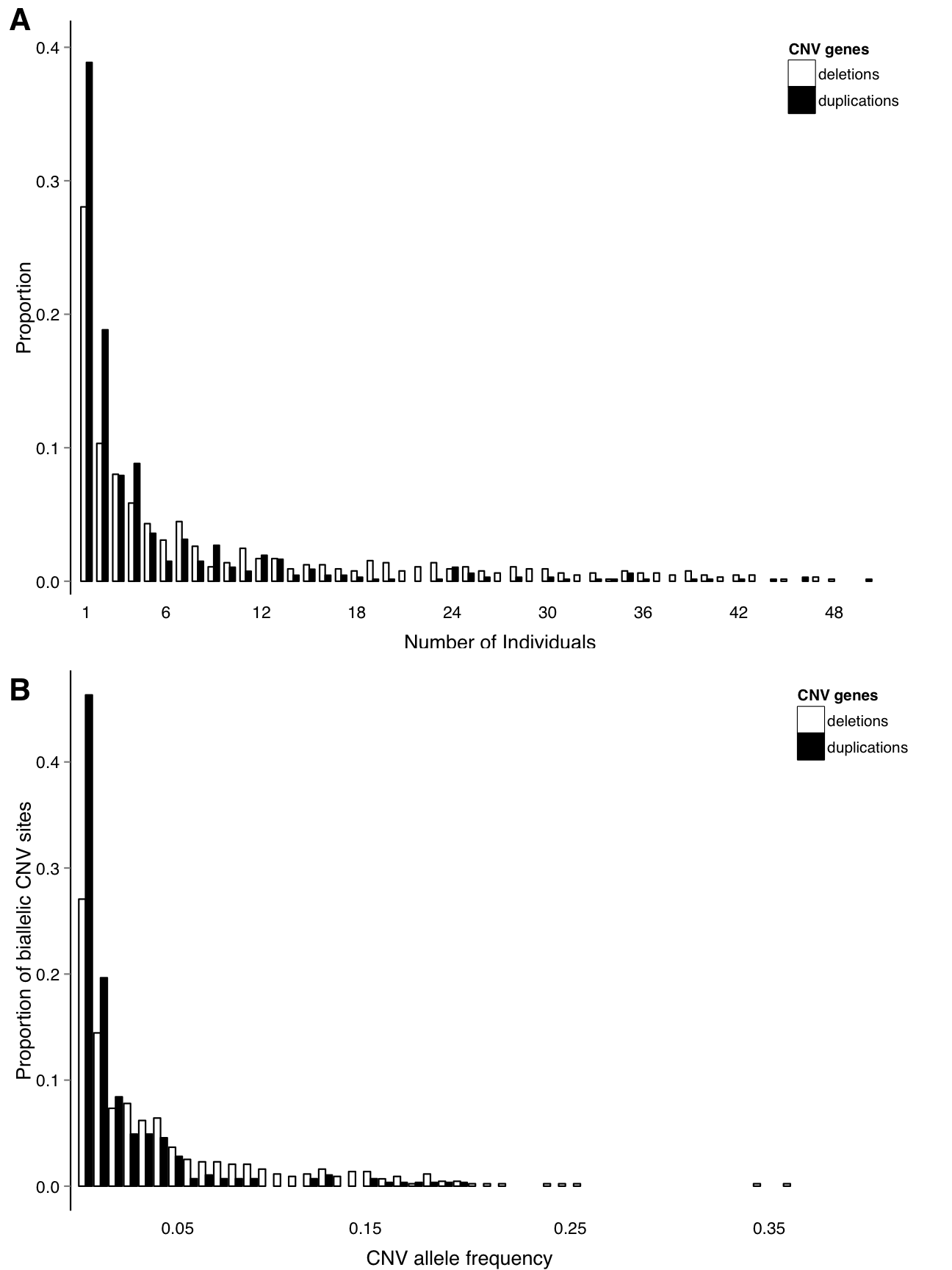

Supplement: Figure S8 — Frequency and sharing of CNV genes across all individuals. (A) Presence of CNV genes among individuals. The proportion of CNV genes (deletions in white, duplications in black) that are shared between individuals (B) Allele frequency spectrum of bi-allelic CNV genes across all 66 individuals, showing most deletions (white) and duplications (black) occurring at very low frequencies. (analogous to S3 Figure). (TIF) [file pgen.1004830.s008.tif]

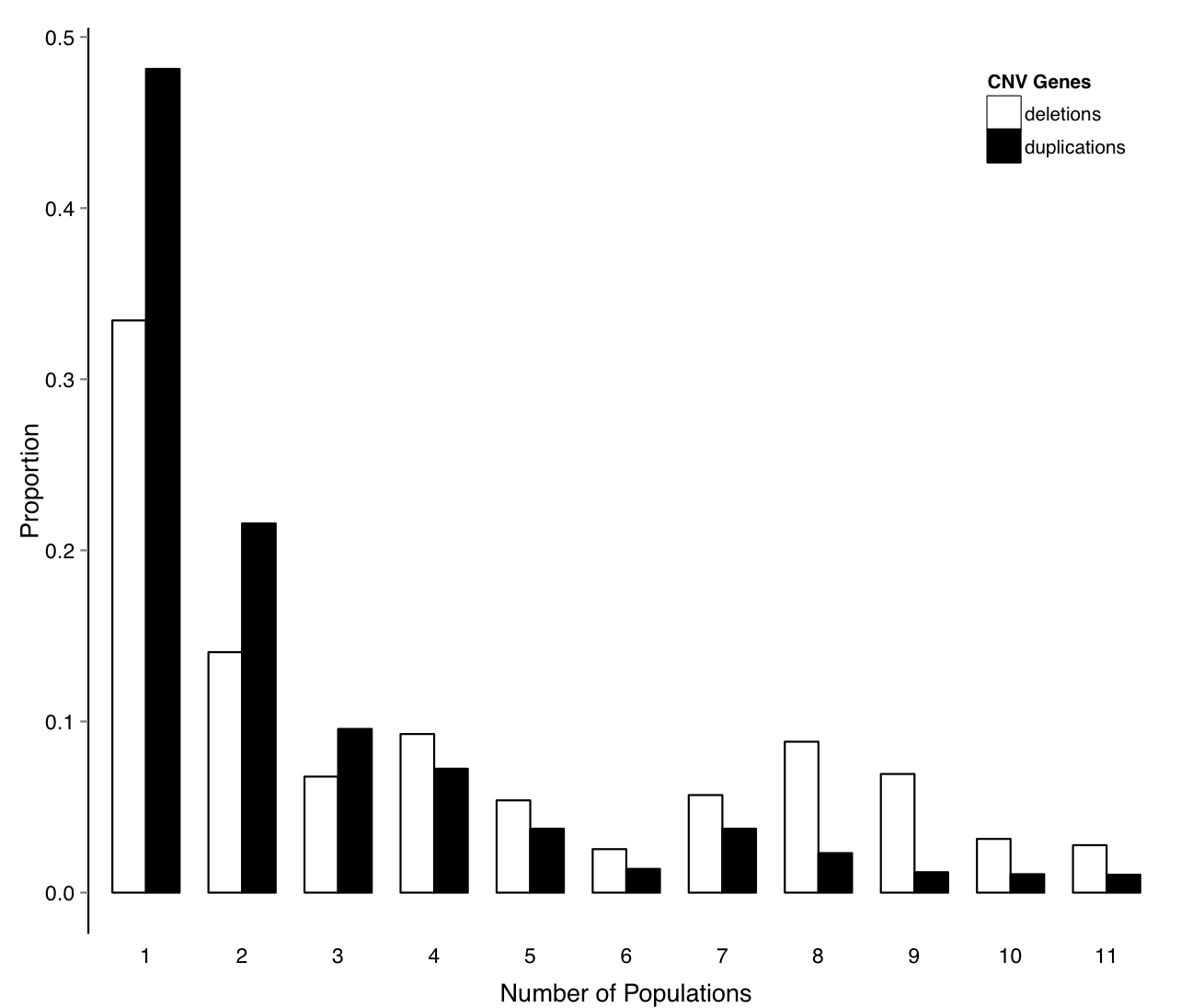

Supplement: Figure S9 — CNV genes shared across populations. Occurrence of CNV genes across populations. The proportion of CNV genes (deletions in white, duplications in black) that are shared between individuals. This figure is analogous to Fig. 2C in the main text. (TIF) [file pgen.1004830.s009.tif]

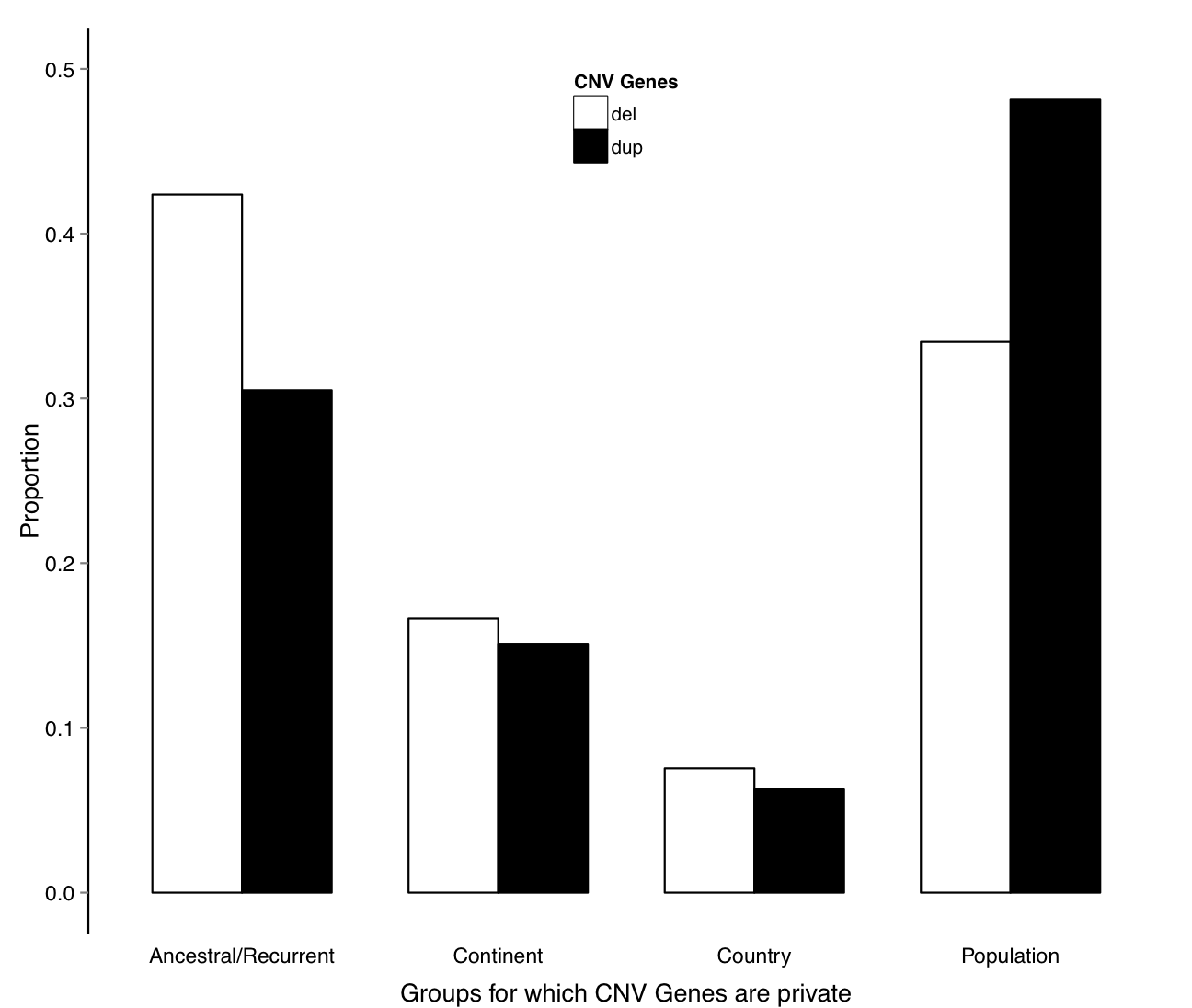

Supplement: Figure S10 — Proportion of CNV genes that are specific to groups of individuals. Proportion of CNV genes that are specific (private) to groups of individuals spanning different scales of divergence. Mutually exclusive groups for which CNV genes are private include: those occurring across continents (Ancestral/Recurrent), those specific to a continent but shared across populations from different countries (Continent), those specific to a country but shared across populations within a country (Country), and those only found in one population (Population). This figure is analogous to Fig. 2D in the main text. (TIF) [file pgen.1004830.s010.tif]

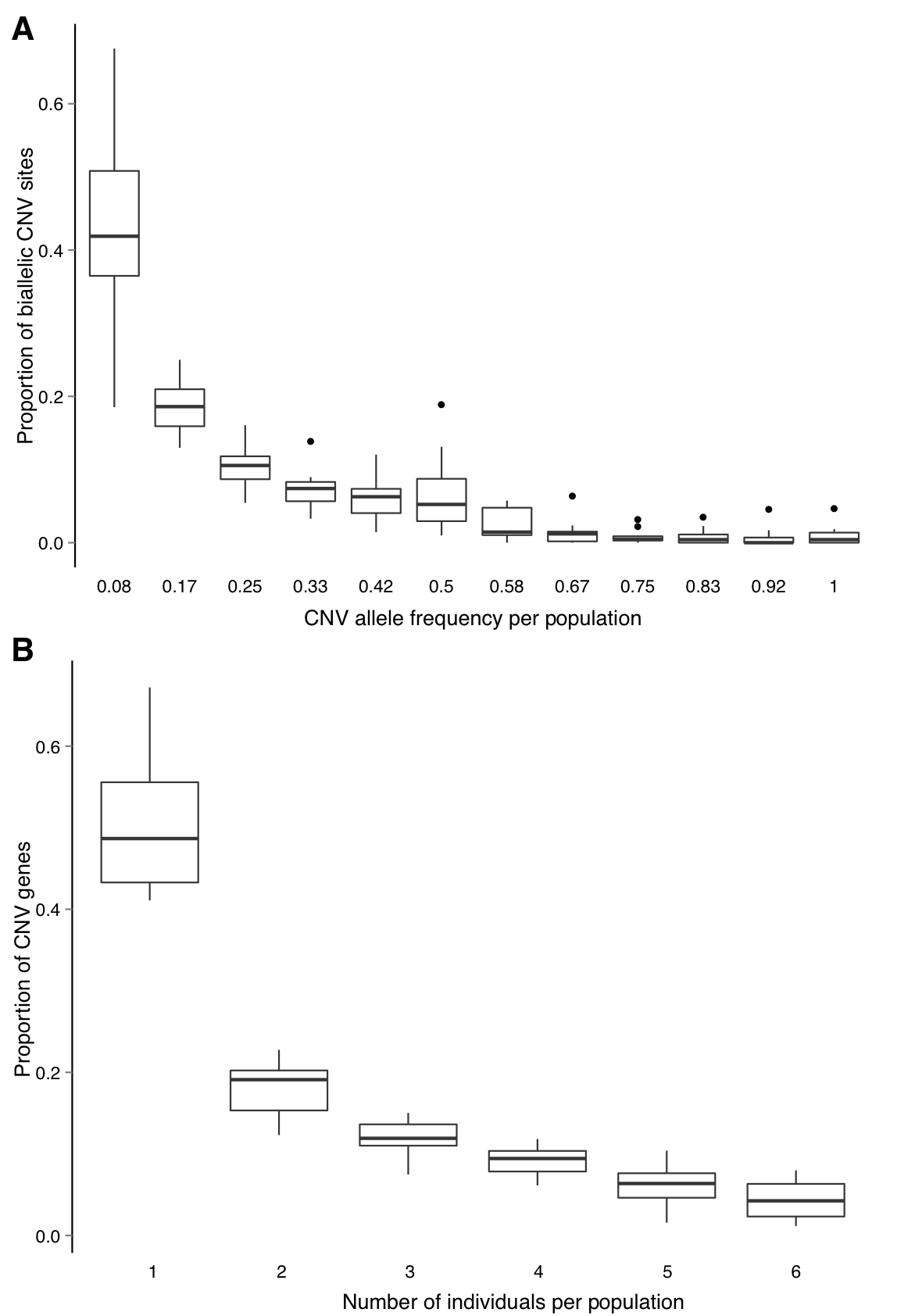

Supplement: Figure S11 — Frequency and sharing of CNV genes across individuals within populations. (A) Allele frequency spectrum of non-reference alleles from bi-allelic CNV genes across 12 individuals from each population represented as boxplots (analogous to Fig. 2B in the main text). (B) Boxplots showing the proportion of CNV genes shared across individuals within populations (from a single individual up to all 6 individuals, analogous to Fig. 2E in the main text) (TIF) [file pgen.1004830.s011.tif]

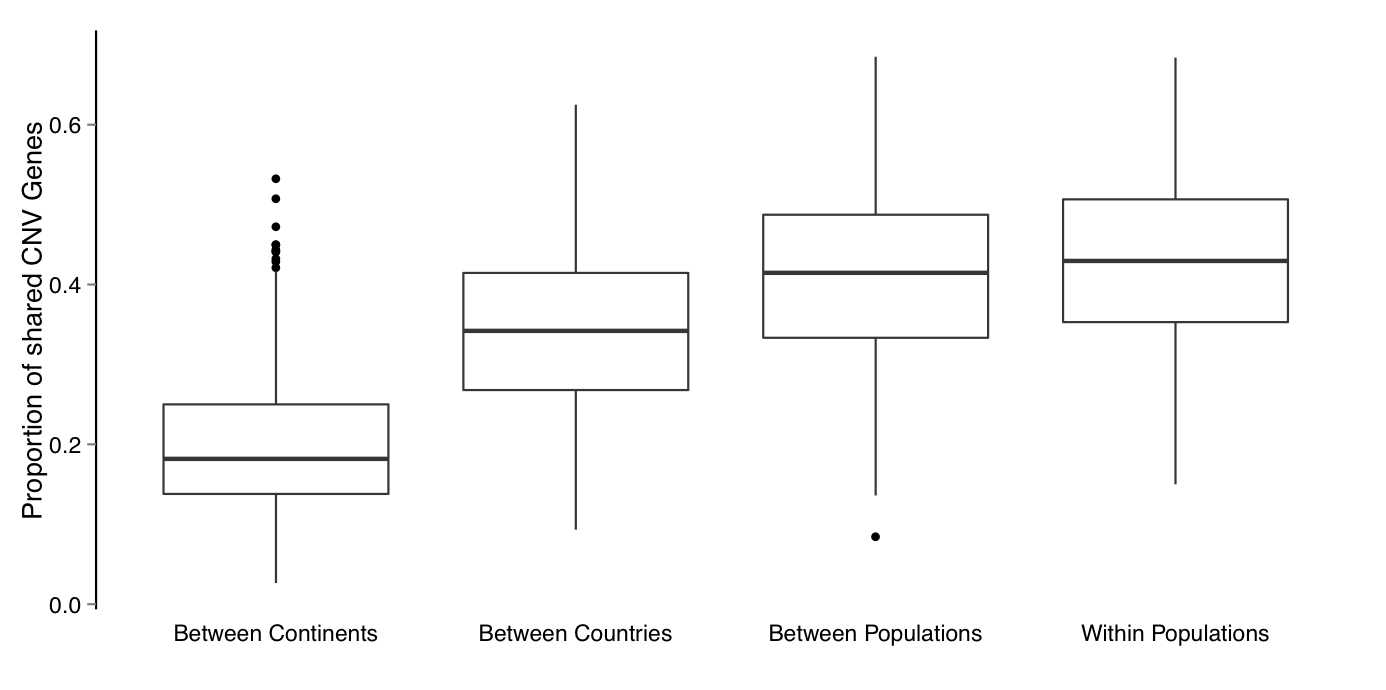

Supplement: Figure S12 — Average proportion of shared CNV genes. Average proportion of shared CNV genes between individuals across mutually exclusive groups. The proportion of CNV gene sharing was calculated for four groups: “Between Continents” is sharing across individuals from different continents, “Between Countries” is sharing across individuals from different countries within the same continent, “Between Populations” is sharing across individuals from different populations from the same country, and “Within Populations” is sharing across individuals from the same population. This figure is analogous to Fig. 2F in the main text. (TIF) [file pgen.1004830.s012.tif]

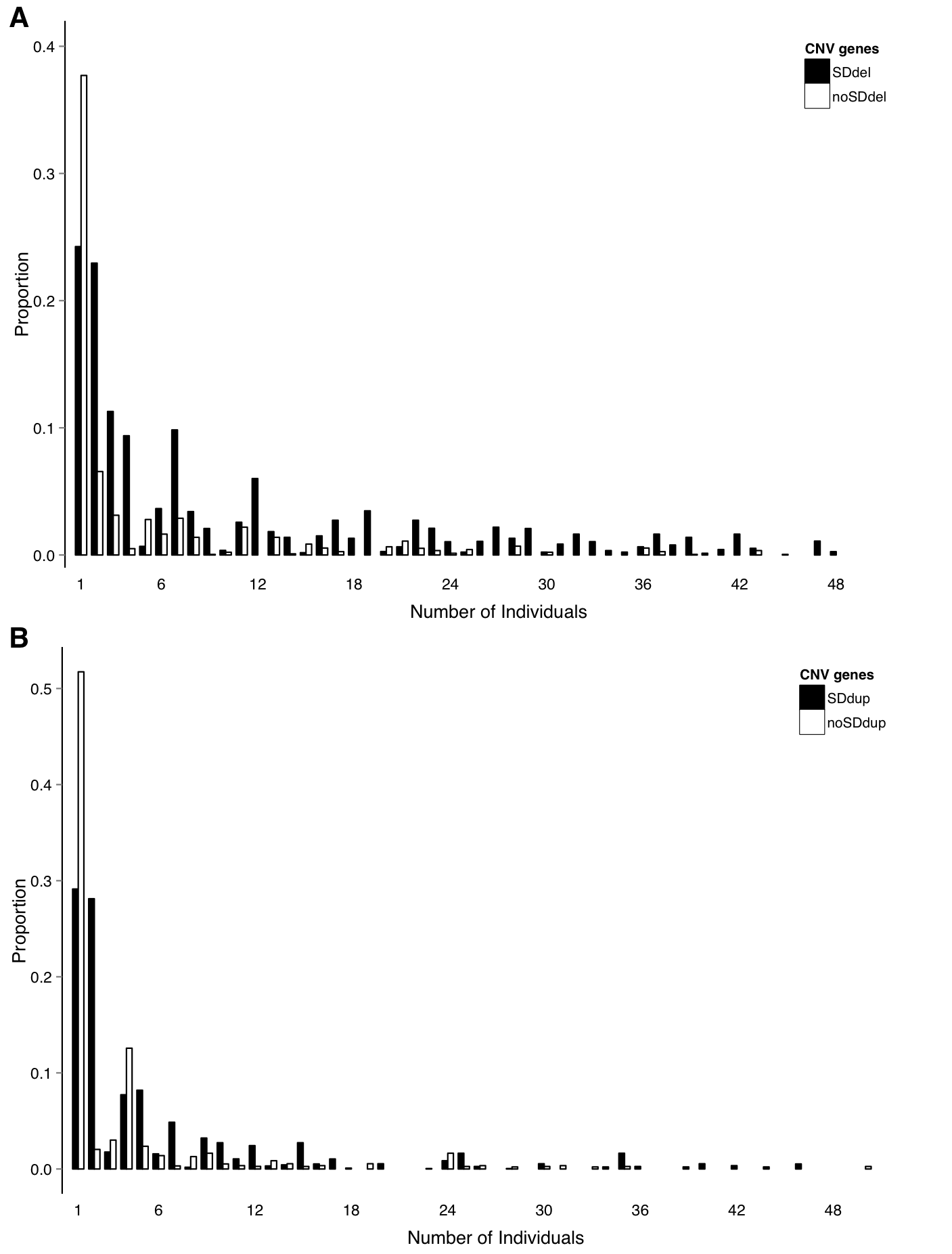

Supplement: Figure S14 — CNV genes shared across individuals and segmental duplication overlap. CNV genes shared across individuals depending on segmental duplication (SD) overlap. For both (A) deletions and (B) duplications, the proportion of CNV genes shared in segmental duplications (black) or outside of segmental duplications (white) is shown. Genes outside of segmental duplications are generally found in fewer individuals. (TIF) [file pgen.1004830.s014.tif]

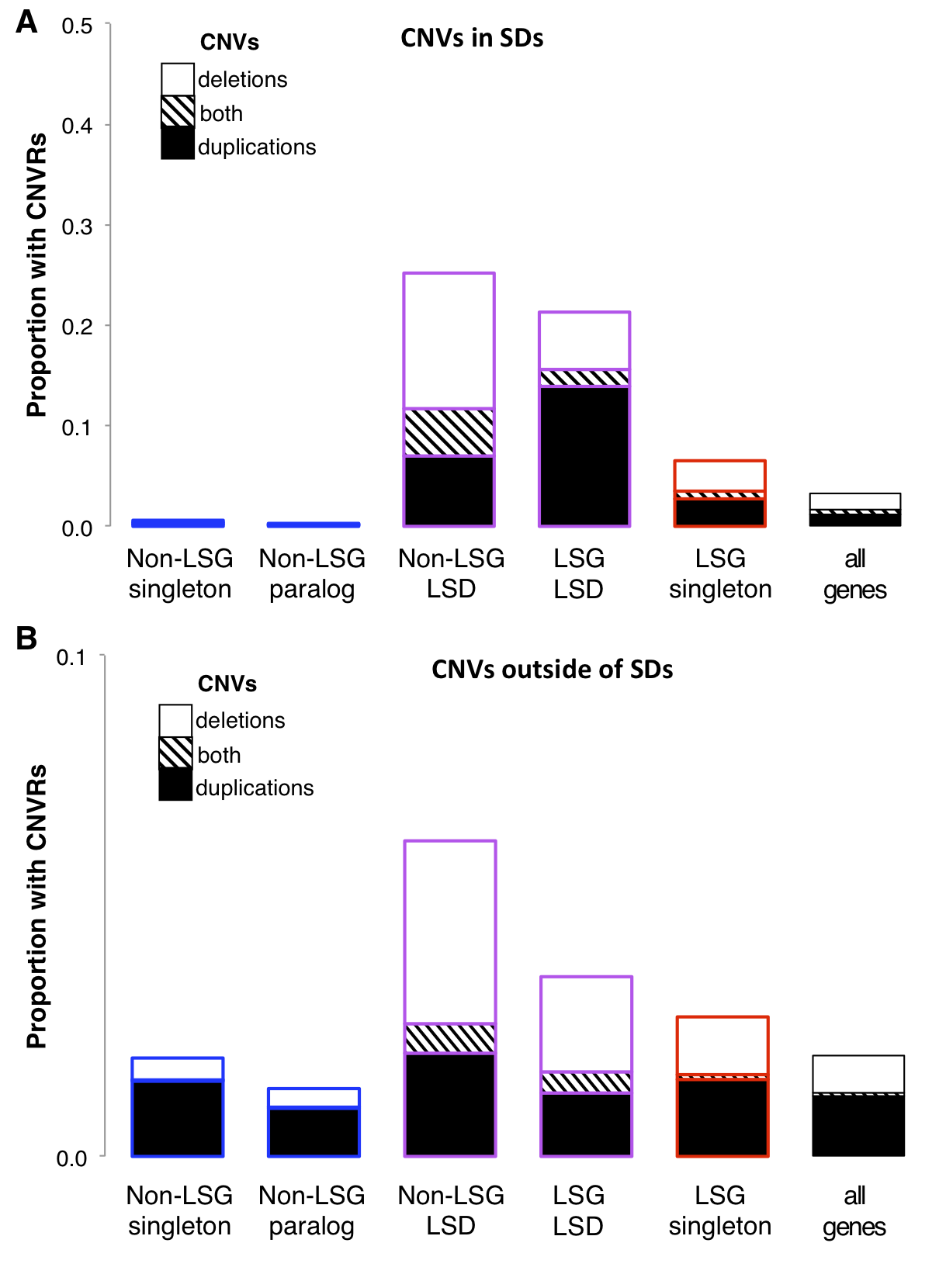

Supplement: Figure S15 — Proportion of genes overlapping CNVs and segmental duplication overlap. Proportion of genes overlapping CNVs (A) in segmental duplications (SDs) and (B) outside of SDs. Analogous to Fig. 4B in the main text. The proportion of genes (both protein-coding and RNA) from each gene category (non-LSG singletons, non-LSG paralogs, non-LSG LSD, LSG LSD, LSG singletons and all genes in total) that is completely encompassed within CNVs (deletions in white, duplications in black, both deletion and duplication in stripes). (TIF) [file pgen.1004830.s015.tif]

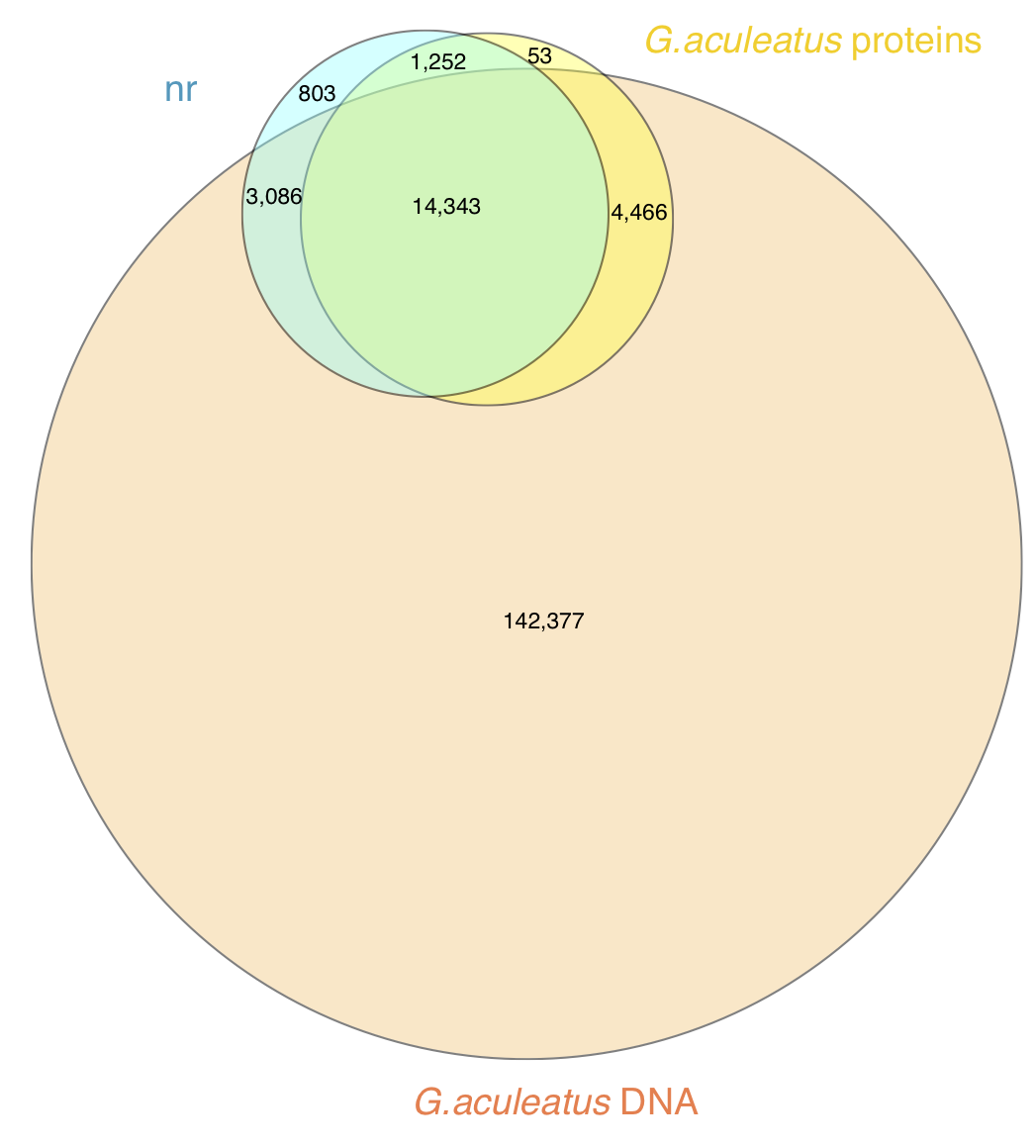

Supplement: Figure S17 — Overlap of BLAST hits of unmapped contigs. Overlap of BLAST hits of the unmapped contigs versus the nr database (nr), stickleback genome (G.aculeatus DNA) and stickleback proteins (G.aculeatus proteins). Only a very small proportion of contigs hit non-stickleback sequences (803). (TIF) [file pgen.1004830.s017.tif]

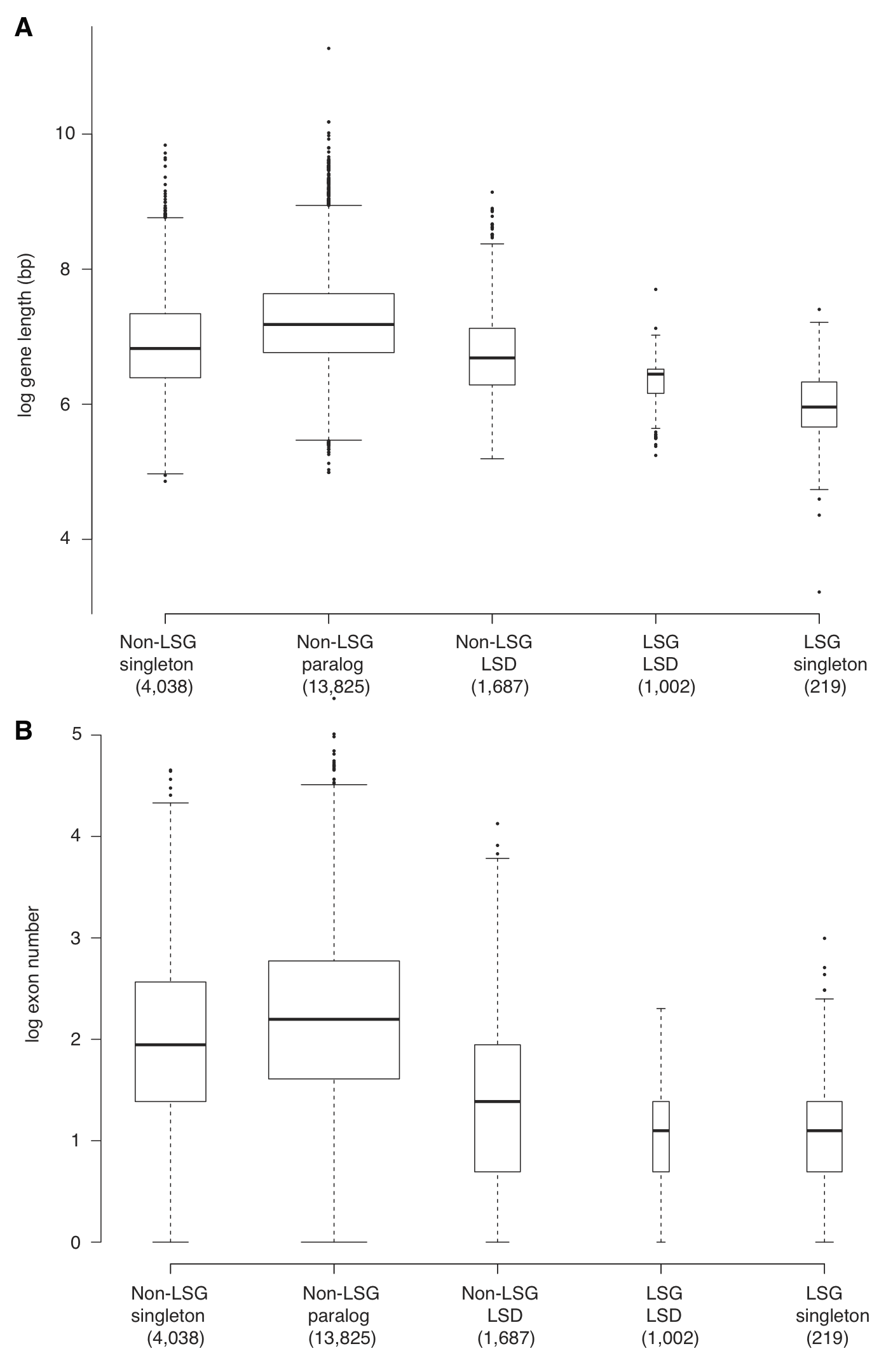

Supplement: Figure S18 — Structural properties of genes.Structural properties of genes across categories. Lineage-specific gene categories (LSG LSD and LSG singleton) have (A) shorter gene lengths and (B) fewer exons. (TIF) [file pgen.1004830.s018.tif]

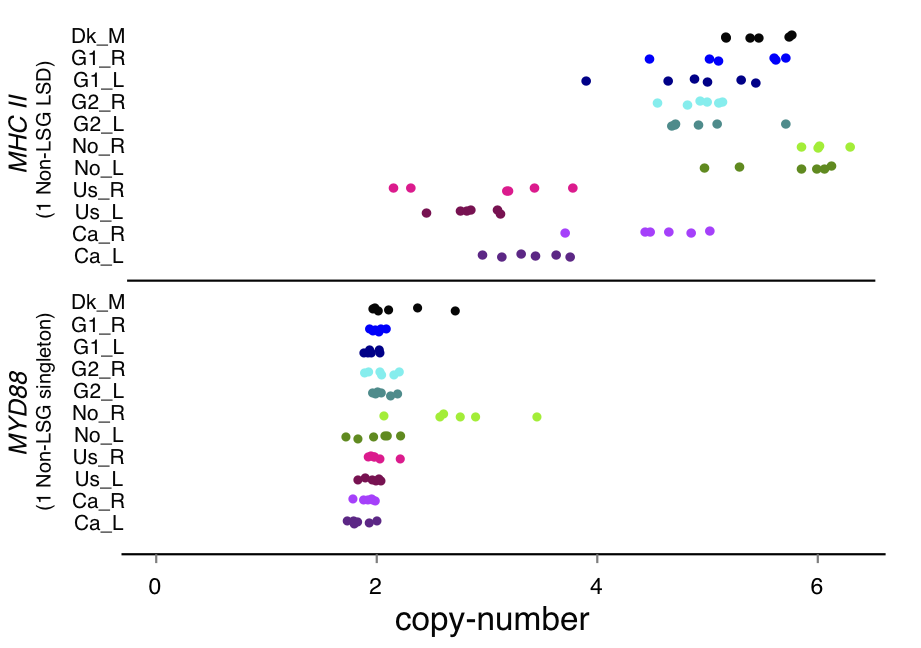

Supplement: Figure S19 — CNV of immune genes. Copy-number variation of immune related genes across populations. Normalized read depth approximating gene copy-number is plotted across 66 individuals grouped and colored by population following the format of Fig. 6 . (TIF) [file pgen.1004830.s019.tif]

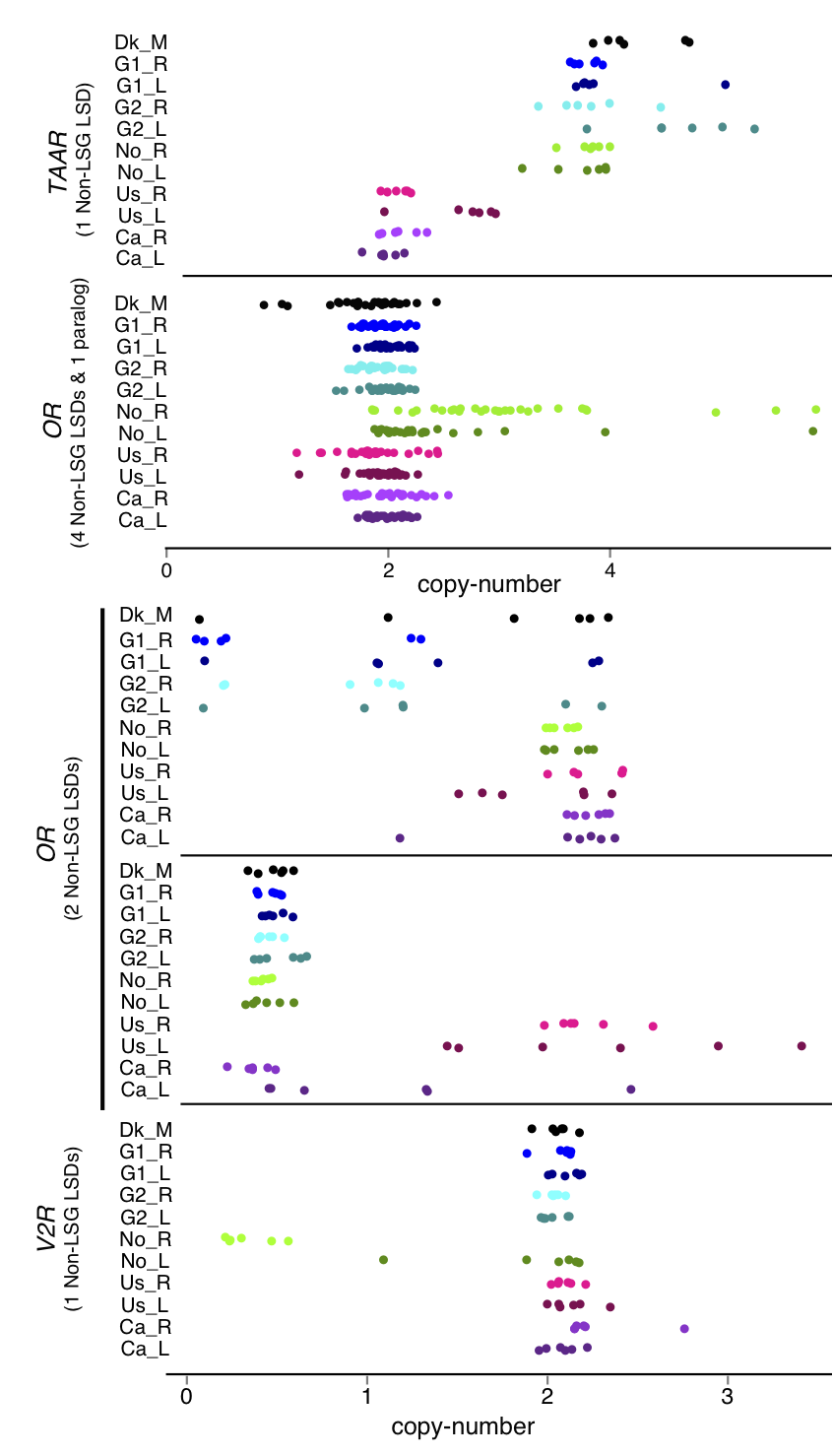

Supplement: Figure S20 — CNV of olfaction genes. Copy-number variation of olfactory related genes across populations. Normalized read depth approximating gene copy-number is plotted across 66 individuals grouped and colored by population following the format of Fig. 6 . (TIF) [file pgen.1004830.s020.tif]

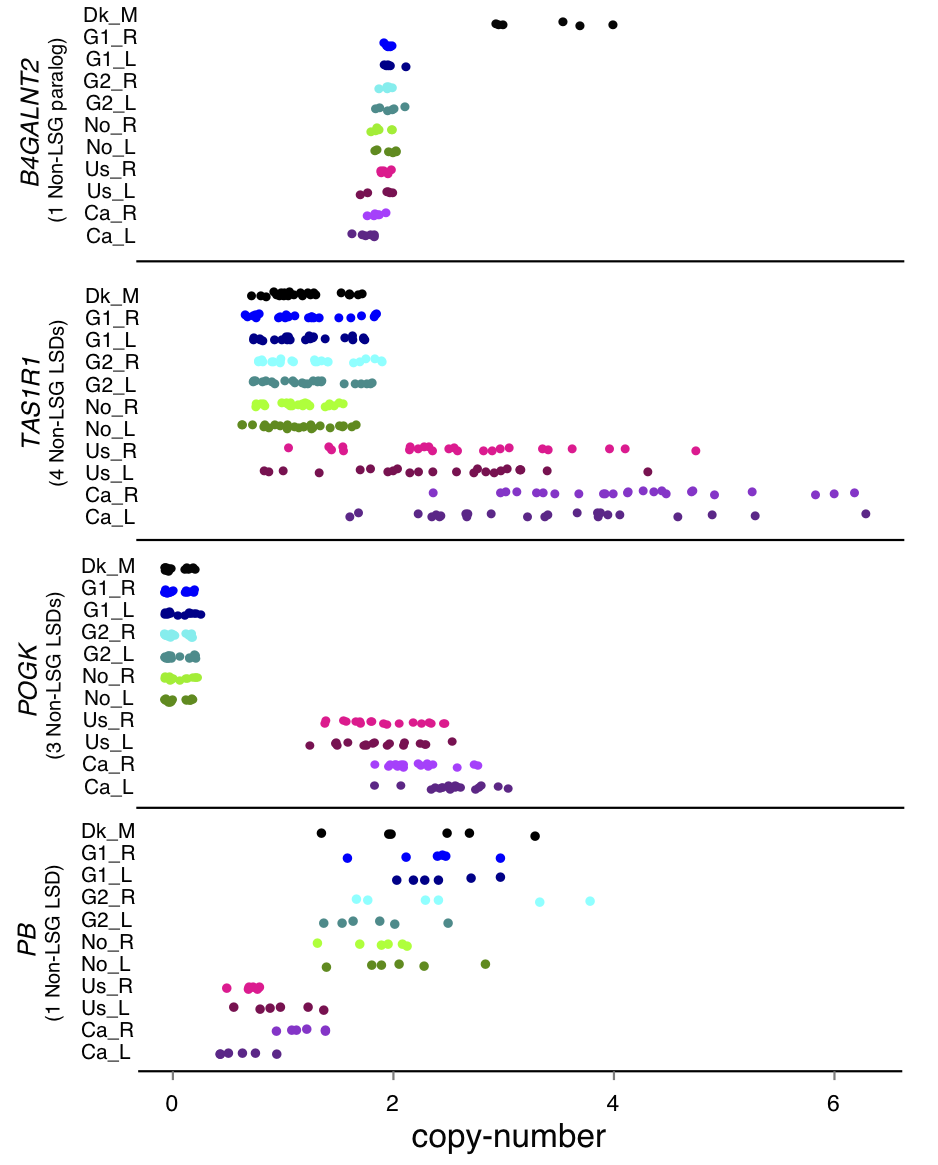

Supplement: Figure S21 — CNV of group-specific gene expansions and losses. Copy-number variation of group-specific gene expansions and losses. Normalized read depth approximating gene copy-number is plotted across 66 individuals grouped and colored by population following the format of Fig. 6 . (TIF) [file pgen.1004830.s021.tif]

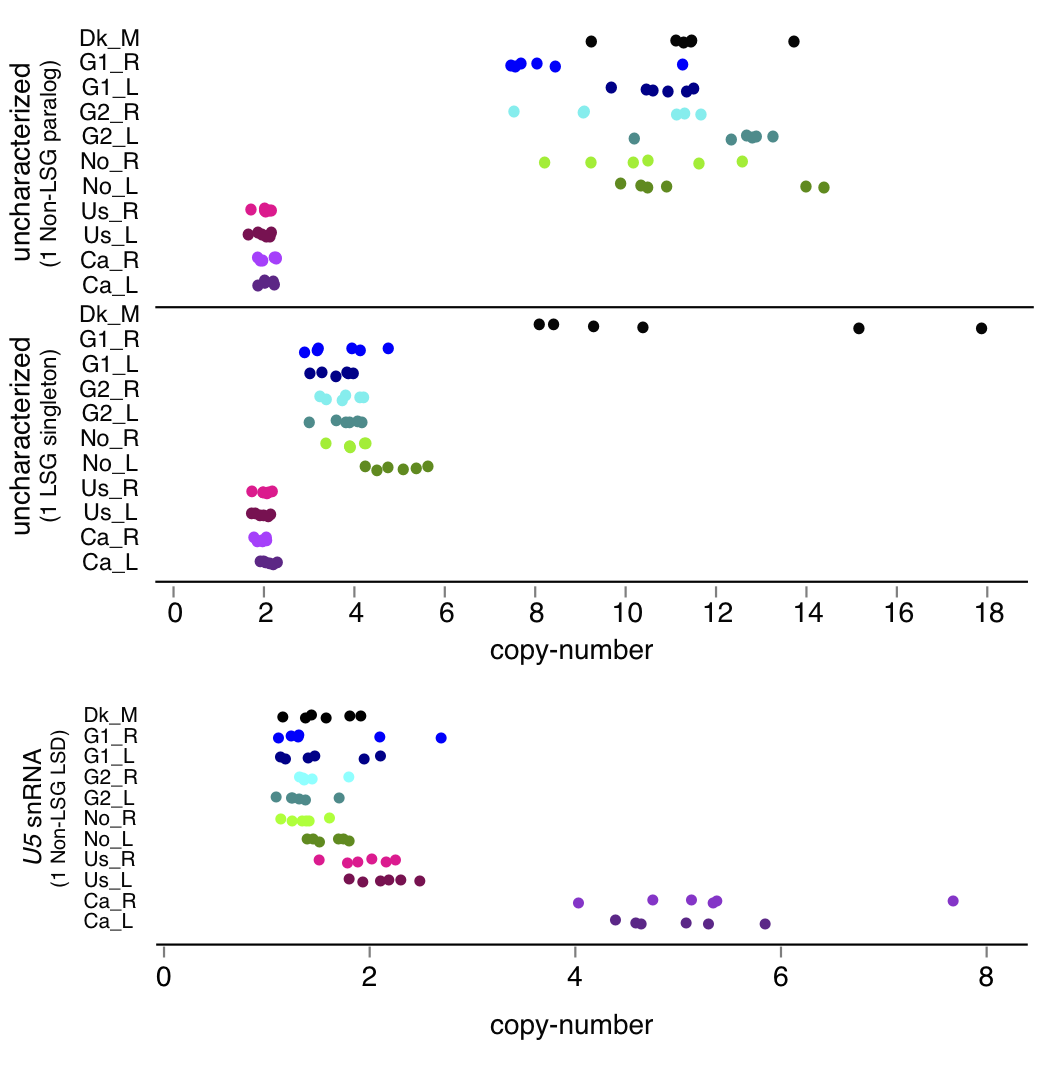

Supplement: Figure S22 — High CNV differences between groups. High copy-number variation of group-specific gene expansions and losses. Normalized read depth approximating gene copy-number is plotted across 66 individuals grouped and colored by population following the format of Fig. 6 . (TIF) [file pgen.1004830.s022.tif]
