# Supplementary material for: Extensive Copy-Number Variation of Young Genes across Stickleback Populations
Source: PLoS Genet. 2014 Dec 4;10(12):e1004830. doi: 10.1371/journal.pgen.1004830 (PMC4256280; doi:10.1371/journal.pgen.1004830)
Supplement: Table S1 — Summary of sequencing statistics and sample information. (PDF) [file pgen.1004830.s023.pdf]

Supplementary Table 1 - Summary of sequencing statistics and sample information

Individual ID	Raw data (Gbp)	Raw data depth (x fold)	Clean data (Gbp)	Mapping %	Final depth of coverage	Population ID	Population	Location	Coordinates	Latitude	Longitude	Ecotype	Sex
**Total**	**1235.3**	**2685.5**	**964.9**	**-**	**-**								
**Average**	**18.7**	**40.7**	**14.6**	**83.2**	**26.4**								
BS1	16.1	35.0	14.1	82.8	25.5	G1_R	Malenter Au	Schleswig-Holstein, Germany	54°12'15.08"N, 10°33'41.90"E	54.204189	10.561639	River	Female
BS2	27.2	59.0	19.7	81.2	34.8	G1_L	Grosser Ploener See	Schleswig-Holstein, Germany	54° 9'21.61"N , 10°25'48.52"E	54.156003	10.430144	Lake	Female
BS3	20.4	44.4	18.3	79.4	31.7	G1_R	Malenter Au	Schleswig-Holstein, Germany	54°12'15.08"N, 10°33'41.90"E	54.204189	10.561639	River	Male
BS4	27.6	60.0	23.5	76.5	39.0	G1_L	Grosser Ploener See	Schleswig-Holstein, Germany	54° 9'21.61"N , 10°25'48.52"E	54.156003	10.430144	Lake	Male
BS5	18.9	41.2	15.5	79.9	26.9	G1_R	Malenter Au	Schleswig-Holstein, Germany	54°12'15.08"N, 10°33'41.90"E	54.204189	10.561639	River	Male
BS6	24.9	54.1	17.7	77.0	29.6	G1_L	Grosser Ploener See	Schleswig-Holstein, Germany	54° 9'21.61"N , 10°25'48.52"E	54.156003	10.430144	Lake	Male
BS7	22.7	49.4	19.1	80.9	33.5	G1_R	Malenter Au	Schleswig-Holstein, Germany	54°12'15.08"N, 10°33'41.90"E	54.204189	10.561639	River	Female
BS8	16.4	35.6	14.1	82.2	25.2	G1_L	Grosser Ploener See	Schleswig-Holstein, Germany	54° 9'21.61"N , 10°25'48.52"E	54.156003	10.430144	Lake	Female
BS9	16.4	35.7	14.2	81.4	25.2	G1_R	Malenter Au	Schleswig-Holstein, Germany	54°12'15.08"N, 10°33'41.90"E	54.204189	10.561639	River	Male
BS10	21.2	46.0	18.1	80.9	31.8	G1_L	Grosser Ploener See	Schleswig-Holstein, Germany	54° 9'21.61"N , 10°25'48.52"E	54.156003	10.430144	Lake	Female
BS11	15.5	33.8	12.9	79.4	22.2	G1_R	Malenter Au	Schleswig-Holstein, Germany	54°12'15.08"N, 10°33'41.90"E	54.204189	10.561639	River	Female
BS12	15.7	34.1	13.2	77.8	22.4	G1_L	Grosser Ploener See	Schleswig-Holstein, Germany	54° 9'21.61"N , 10°25'48.52"E	54.156003	10.430144	Lake	Male
BS13	19.7	42.9	15.6	70.7	24.0	G2_R	Eider	Schleswig-Holstein, Germany	54° 09' 58.07'' N, 10° 04' 31.05'' E	54.167638	10.075206	River	Male
BS14	13.7	29.7	11.1	66.4	16.0	G2_L	Westensee	Schleswig-Holstein, Germany	54°16'39.70"N, 9°55'41.04"E	54.277694	9.928067	Lake	Female
BS15	16.4	35.7	13.3	74.0	21.4	G2_R	Eider	Schleswig-Holstein, Germany	54° 09' 58.07'' N, 10° 04' 31.05'' E	54.167638	10.075206	River	Female
BS16	15.4	33.4	13.0	85.8	24.3	G2_L	Westensee	Schleswig-Holstein, Germany	54°16'39.70"N, 9°55'41.04"E	54.277694	9.928067	Lake	Male
BS17	24.1	52.4	17.5	81.2	30.8	G2_R	Eider	Schleswig-Holstein, Germany	54° 09' 58.07'' N, 10° 04' 31.05'' E	54.167638	10.075206	River	Male
BS18	17.2	37.4	14.3	73.5	22.9	G2_L	Westensee	Schleswig-Holstein, Germany	54°16'39.70"N, 9°55'41.04"E	54.277694	9.928067	Lake	Male
BS19	19.1	41.6	12.5	80.6	21.9	G2_R	Eider	Schleswig-Holstein, Germany	54° 09' 58.07'' N, 10° 04' 31.05'' E	54.167638	10.075206	River	Female
BS20	17.5	38.0	9.5	85.0	17.7	G2_L	Westensee	Schleswig-Holstein, Germany	54°16'39.70"N, 9°55'41.04"E	54.277694	9.928067	Lake	Female
BS21	14.5	31.5	12.5	78.8	21.5	G2_R	Eider	Schleswig-Holstein, Germany	54° 09' 58.07'' N, 10° 04' 31.05'' E	54.167638	10.075206	River	Male
BS22	13.1	28.5	11.2	78.1	19.1	G2_L	Westensee	Schleswig-Holstein, Germany	54°16'39.70"N, 9°55'41.04"E	54.277694	9.928067	Lake	Male
BS23	17.1	37.1	15.3	84.1	28.0	G2_R	Eider	Schleswig-Holstein, Germany	54° 09' 58.07'' N, 10° 04' 31.05'' E	54.167638	10.075206	River	Female
BS24	16.0	34.8	13.8	78.2	23.5	G2_L	Westensee	Schleswig-Holstein, Germany	54°16'39.70"N, 9°55'41.04"E	54.277694	9.928067	Lake	Female
BS25	15.8	34.2	13.3	74.0	21.4	Dk_M	North Sea	Lemvig, Denmark	56°34' 14.63"N, 8°17' 32.64"E	56.570731	8.292400	Marine	Male
BS26	17.2	37.3	14.6	78.8	25.0	Dk_M	North Sea	Lemvig, Denmark	56°34' 14.63"N, 8°17' 32.64"E	56.570731	8.292400	Marine	Female
BS27	15.6	33.9	13.3	75.8	21.9	Dk_M	North Sea	Lemvig, Denmark	56°34' 14.63"N, 8°17' 32.64"E	56.570731	8.292400	Marine	Male
BS28	13.7	29.8	12.0	79.1	20.7	Dk_M	North Sea	Lemvig, Denmark	56°34' 14.63"N, 8°17' 32.64"E	56.570731	8.292400	Marine	Male
BS29	14.4	31.3	12.5	78.2	21.2	Dk_M	North Sea	Lemvig, Denmark	56°34' 14.63"N, 8°17' 32.64"E	56.570731	8.292400	Marine	Female
BS30	20.6	44.9	18.7	80.2	32.5	Dk_M	North Sea	Lemvig, Denmark	56°34' 14.63"N, 8°17' 32.64"E	56.570731	8.292400	Marine	Female
BS31b	15.5	33.6	13.9	88.3	26.7	Us_R	Meadow Creek	Alaska, USA	61° 34' 8.76"N, 149° 45' 36.00"W	61.569100	-149.760000	River	Female
BS32b	17.9	38.8	15.9	87.5	30.2	Us_L	Long Lake	Alaska, USA	61° 34' 33.96"N, 149° 46' 25.50"W	61.576100	-149.773750	Lake	Female
BS33	29.0	63.1	22.0	88.2	42.2	Us_R	Meadow Creek	Alaska, USA	61° 34' 8.76"N, 149° 45' 36.00"W	61.569100	-149.760000	River	Male
BS34b	13.2	28.8	12.1	87.4	23.1	Us_L	Long Lake	Alaska, USA	61° 34' 33.96"N, 149° 46' 25.50"W	61.576100	-149.773750	Lake	Male
BS35	33.1	72.0	18.1	88.6	34.8	Us_R	Meadow Creek	Alaska, USA	61° 34' 8.76"N, 149° 45' 36.00"W	61.569100	-149.760000	River	Female
BS36b	18.1	39.4	16.2	87.1	30.6	Us_L	Long Lake	Alaska, USA	61° 34' 33.96"N, 149° 46' 25.50"W	61.576100	-149.773750	Lake	Female
BS37	25.4	55.2	10.8	88.3	20.7	Us_R	Meadow Creek	Alaska, USA	61° 34' 8.76"N, 149° 45' 36.00"W	61.569100	-149.760000	River	Male
BS38b	28.1	61.1	7.0	89.0	13.5	Us_L	Long Lake	Alaska, USA	61° 34' 33.96"N, 149° 46' 25.50"W	61.576100	-149.773750	Lake	Male
BS39	22.6	49.2	12.4	84.8	22.9	Us_R	Meadow Creek	Alaska, USA	61° 34' 8.76"N, 149° 45' 36.00"W	61.569100	-149.760000	River	Female
BS40b	26.3	57.2	24.8	86.0	46.3	Us_L	Long Lake	Alaska, USA	61° 34' 33.96"N, 149° 46' 25.50"W	61.576100	-149.773750	Lake	Female

BS41	28.6	62.2	25.4	86.8	47.9	Us_R	Meadow Creek	Alaska, USA	61° 34' 8.76"N, 149° 45' 36.00"W	61.569100	-149.760000	River	Male
BS42b	24.2	52.6	19.2	89.1	37.1	Us_L	Long Lake	Alaska, USA	61° 34' 33.96"N, 149° 46' 25.50"W	61.576100	-149.773750	Lake	Female
BS43	15.7	34.1	13.8	86.9	26.1	Ca_R	Misty Stream Inlet	Vancouver Island, Canada	50° 36' 10.40"N, 127° 15' 8.30"W	50.602889	-127.252306	River	Male
BS44	15.6	33.9	6.3	88.2	12.1	Ca_L	Misty Lake	Vancouver Island, Canada	50° 36' 16.40"N, 127° 15' 42.30"W	50.604556	-127.261750	Lake	Male
BS45	16.4	35.7	15.2	88.2	29.1	Ca_R	Misty Stream Inlet	Vancouver Island, Canada	50° 36' 10.40"N, 127° 15' 8.30"W	50.602889	-127.252306	River	Female
BS46	14.7	32.0	11.2	86.7	21.2	Ca_L	Misty Lake	Vancouver Island, Canada	50° 36' 16.40"N, 127° 15' 42.30"W	50.604556	-127.261750	Lake	Female
BS47	16.0	34.8	6.6	88.8	12.7	Ca_R	Misty Stream Inlet	Vancouver Island, Canada	50° 36' 10.40"N, 127° 15' 8.30"W	50.602889	-127.252306	River	Male
BS48	15.4	33.5	11.9	87.6	22.6	Ca_L	Misty Lake	Vancouver Island, Canada	50° 36' 16.40"N, 127° 15' 42.30"W	50.604556	-127.261750	Lake	Male
BS49	21.3	46.3	11.1	84.9	20.6	Ca_R	Misty Stream Inlet	Vancouver Island, Canada	50° 36' 10.40"N, 127° 15' 8.30"W	50.602889	-127.252306	River	Female
BS50b	19.7	42.7	15.6	88.0	29.8	Ca_L	Misty Lake	Vancouver Island, Canada	50° 36' 16.40"N, 127° 15' 42.30"W	50.604556	-127.261750	Lake	Female
BS51	19.5	42.3	7.3	86.8	13.7	Ca_R	Misty Stream Inlet	Vancouver Island, Canada	50° 36' 10.40"N, 127° 15' 8.30"W	50.602889	-127.252306	River	Male
BS52b	23.6	51.3	21.4	86.9	40.5	Ca_L	Misty Lake	Vancouver Island, Canada	50° 36' 16.40"N, 127° 15' 42.30"W	50.604556	-127.261750	Lake	Male
BS53	19.1	41.5	17.2	87.8	32.9	Ca_R	Misty Stream Inlet	Vancouver Island, Canada	50° 36' 10.40"N, 127° 15' 8.30"W	50.602889	-127.252306	River	Female
BS54	17.7	38.4	16.2	88.4	31.1	Ca_L	Misty Lake	Vancouver Island, Canada	50° 36' 16.40"N, 127° 15' 42.30"W	50.604556	-127.261750	Lake	Female
BS55	16.7	36.3	14.0	85.4	26.0	No_R	Skogseidvatn River	Fusa, Norway	60° 15' 19.33"N, 5° 55' 35.68"E	60.255369	5.926577	River	Male
BS56	13.2	28.8	11.4	86.2	21.3	No_L	Skogseidvatn Lake	Fusa, Norway	60° 14' 41.57"N, 5° 54' 55.39"E	60.244881	5.915387	Lake	Male
BS57	15.5	33.7	14.3	86.3	26.7	No_R	Skogseidvatn River	Fusa, Norway	60° 15' 19.33"N, 5° 55' 35.68"E	60.255369	5.926577	River	Female
BS58b	16.6	36.2	15.3	86.4	28.7	No_L	Skogseidvatn Lake	Fusa, Norway	60° 14' 41.57"N, 5° 54' 55.39"E	60.244881	5.915387	Lake	Female
BS59	17.1	37.2	13.0	83.1	23.4	No_R	Skogseidvatn River	Fusa, Norway	60° 15' 19.33"N, 5° 55' 35.68"E	60.255369	5.926577	River	Male
BS60	13.5	29.4	8.4	83.5	15.3	No_L	Skogseidvatn Lake	Fusa, Norway	60° 14' 41.57"N, 5° 54' 55.39"E	60.244881	5.915387	Lake	Male
BS61	16.6	36.1	14.6	84.7	26.9	No_R	Skogseidvatn River	Fusa, Norway	60° 15' 19.33"N, 5° 55' 35.68"E	60.255369	5.926577	River	Female
BS62	16.0	34.8	15.6	86.7	29.4	No_L	Skogseidvatn Lake	Fusa, Norway	60° 14' 41.57"N, 5° 54' 55.39"E	60.244881	5.915387	Lake	Male
BS63b	19.0	41.4	17.9	86.8	33.7	No_R	Skogseidvatn River	Fusa, Norway	60° 15' 19.33"N, 5° 55' 35.68"E	60.255369	5.926577	River	Male
BS64b	19.9	43.3	17.7	85.9	33.0	No_L	Skogseidvatn Lake	Fusa, Norway	60° 14' 41.57"N, 5° 54' 55.39"E	60.244881	5.915387	Lake	Male
BS65	13.5	29.3	11.9	84.2	21.8	No_R	Skogseidvatn River	Fusa, Norway	60° 15' 19.33"N, 5° 55' 35.68"E	60.255369	5.926577	River	Female
BS66	16.8	36.6	14.9	87.3	28.3	No_L	Skogseidvatn Lake	Fusa, Norway	60° 14' 41.57"N, 5° 54' 55.39"E	60.244881	5.915387	Lake	Female
