# Supplementary material for: Extensive Copy-Number Variation of Young Genes across Stickleback Populations
Source: PLoS Genet. 2014 Dec 4;10(12):e1004830. doi: 10.1371/journal.pgen.1004830 (PMC4256280; doi:10.1371/journal.pgen.1004830)
Supplement: Table S2 — CNV genotypes for bi-allelic CNVs. Genotype codes are 0 for homozygous deletion, 1 for heterozygous deletion, 2 for wild type diploid, 3 for heterozygous duplication and 4 for homozygous duplication. Frequency represents the respective frequencies of each genotype. The number of CNV alleles, homozygous genotypes with the CNV allele, and heterozygous genotypes with the CNV allele are shown for all 66 individuals (Total), and for each population. (PDF) [file pgen.1004830.s024.pdf]

Supplementary Table 2 - CNV genotypes for bi-allelic CNVs. Genotype codes are 0 for homozygous deletion, 1 for heterozygous deletion, 2 for wild type diploid, 3 for heterozygous duplication and 4 for homozygous duplication. Frequency represents the respective frequencies of each genotype. The number of CNV alleles, homozygous genotypes with the CNV allele, and heterozygous genotypes with the CNV allele are shown for all 66 individuals (Total), and for each population.

Linkage Group	Start	End	Genotype	Frequency	Total	NS	MA	GPS	EI	WS	SKR	SKL	MC	LL	MSV	MLV
groupI	286500	318000	2;3;	56;10;	10;0;10	0;0;0	0;0;0	0;0;0	0;0;0	0;0;0	0;0;0	0;0;0	5;0;5	4;0;4	0;0;0	1;0;1
groupI	371500	375000	0;2;	1;65;	2;1;0	0;0;0	0;0;0	2;1;0	0;0;0	0;0;0	0;0;0	0;0;0	0;0;0	0;0;0	0;0;0	0;0;0
groupI	457500	463000	2;3;	65;1;	1;0;1	0;0;0	0;0;0	0;0;0	0;0;0	0;0;0	0;0;0	0;0;0	0;0;0	1;0;1	0;0;0	0;0;0
groupI	852000	892000	2;3;	61;5;	5;0;5	0;0;0	0;0;0	0;0;0	0;0;0	0;0;0	0;0;0	0;0;0	1;0;1	1;0;1	0;0;0	3;0;3
groupI	1213500	1215500	2;3;	65;1;	1;0;1	0;0;0	0;0;0	1;0;1	0;0;0	0;0;0	0;0;0	0;0;0	0;0;0	0;0;0	0;0;0	0;0;0
groupI	1374000	1376000	1;2;	2;64;	2;0;2	0;0;0	0;0;0	0;0;0	0;0;0	0;0;0	0;0;0	0;0;0	1;0;1	0;0;0	0;0;0	1;0;1
groupI	1460000	1462500	1;2;	4;62;	4;0;4	1;0;1	0;0;0	0;0;0	0;0;0	0;0;0	0;0;0	0;0;0	1;0;1	1;0;1	0;0;0	1;0;1
groupI	1487500	1491000	1;2;	1;65;	1;0;1	0;0;0	0;0;0	0;0;0	0;0;0	0;0;0	0;0;0	0;0;0	1;0;1	0;0;0	0;0;0	0;0;0
groupI	1747000	1754000	1;2;	1;65;	1;0;1	0;0;0	0;0;0	0;0;0	0;0;0	0;0;0	0;0;0	1;0;1	0;0;0	0;0;0	0;0;0	0;0;0
groupI	2381500	2383500	1;2;	3;63;	3;0;3	0;0;0	0;0;0	2;0;2	0;0;0	0;0;0	0;0;0	0;0;0	1;0;1	0;0;0	0;0;0	0;0;0
groupI	2433500	2437000	1;2;	1;65;	1;0;1	0;0;0	0;0;0	0;0;0	0;0;0	1;0;1	0;0;0	0;0;0	0;0;0	0;0;0	0;0;0	0;0;0
groupI	2462500	2466000	1;2;	1;65;	1;0;1	0;0;0	0;0;0	0;0;0	0;0;0	0;0;0	0;0;0	1;0;1	0;0;0	0;0;0	0;0;0	0;0;0
groupI	2478500	2482500	1;2;	1;65;	1;0;1	0;0;0	0;0;0	0;0;0	0;0;0	1;0;1	0;0;0	0;0;0	0;0;0	0;0;0	0;0;0	0;0;0
groupI	2596000	2601500	2;3;	65;1;	1;0;1	0;0;0	0;0;0	0;0;0	0;0;0	0;0;0	0;0;0	0;0;0	0;0;0	0;0;0	0;0;0	1;0;1
groupI	2839000	2844000	1;2;	1;65;	1;0;1	0;0;0	0;0;0	0;0;0	1;0;1	0;0;0	0;0;0	0;0;0	0;0;0	0;0;0	0;0;0	0;0;0
groupI	2995500	3028500	2;3;	60;6;	6;0;6	0;0;0	0;0;0	1;0;1	0;0;0	0;0;0	3;0;3	2;0;2	0;0;0	0;0;0	0;0;0	0;0;0
groupI	3087000	3095000	2;3;	65;1;	1;0;1	0;0;0	0;0;0	0;0;0	0;0;0	0;0;0	0;0;0	1;0;1	0;0;0	0;0;0	0;0;0	0;0;0
groupI	3644500	3645500	1;2;	1;65;	1;0;1	0;0;0	0;0;0	1;0;1	0;0;0	0;0;0	0;0;0	0;0;0	0;0;0	0;0;0	0;0;0	0;0;0
groupI	3724000	3726000	1;2;	2;64;	2;0;2	0;0;0	0;0;0	0;0;0	2;0;2	0;0;0	0;0;0	0;0;0	0;0;0	0;0;0	0;0;0	0;0;0
groupI	4072000	4077000	2;3;	64;2;	2;0;2	0;0;0	0;0;0	1;0;1	1;0;1	0;0;0	0;0;0	0;0;0	0;0;0	0;0;0	0;0;0	0;0;0
groupI	4236500	4241500	0;1;2;	1;5;60;	7;1;5	0;0;0	0;0;0	3;1;1	2;0;2	2;0;2	0;0;0	0;0;0	0;0;0	0;0;0	0;0;0	0;0;0
groupI	4307000	4308000	0;1;2;	1;6;59;	8;1;6	0;0;0	7;1;5	1;0;1	0;0;0	0;0;0	0;0;0	0;0;0	0;0;0	0;0;0	0;0;0	0;0;0
groupI	5017500	5018500	0;1;2;	1;3;62;	5;1;3	0;0;0	0;0;0	0;0;0	5;1;3	0;0;0	0;0;0	0;0;0	0;0;0	0;0;0	0;0;0	0;0;0
groupI	5160000	5162000	1;2;	2;64;	2;0;2	0;0;0	0;0;0	0;0;0	0;0;0	0;0;0	0;0;0	0;0;0	0;0;0	0;0;0	0;0;0	2;0;2
groupI	5179500	5181500	1;2;	1;65;	1;0;1	0;0;0	0;0;0	0;0;0	0;0;0	0;0;0	0;0;0	0;0;0	0;0;0	1;0;1	0;0;0	0;0;0
groupI	5241500	5251500	2;3;	62;4;	4;0;4	0;0;0	0;0;0	4;0;4	0;0;0	0;0;0	0;0;0	0;0;0	0;0;0	0;0;0	0;0;0	0;0;0
groupI	5429000	5432000	1;2;	2;64;	2;0;2	0;0;0	0;0;0	0;0;0	0;0;0	0;0;0	0;0;0	0;0;0	0;0;0	0;0;0	2;0;2	0;0;0
groupI	5600500	5602500	0;1;2;	1;5;60;	7;1;5	0;0;0	0;0;0	0;0;0	0;0;0	0;0;0	5;1;3	2;0;2	0;0;0	0;0;0	0;0;0	0;0;0

groupI	5737000	5760500	2;3;	64;2;	2;0;2	0;0;0	0;0;0	0;0;0	0;0;0	0;0;0	0;0;0	0;0;0	0;0;0	0;0;0	0;0;0	2;0;2
groupI	5969500	5972500	1;2;	1;65;	1;0;1	0;0;0	0;0;0	0;0;0	0;0;0	0;0;0	0;0;0	0;0;0	0;0;0	0;0;0	1;0;1	0;0;0
groupI	6210000	6220500	2;3;	64;2;	2;0;2	2;0;2	0;0;0	0;0;0	0;0;0	0;0;0	0;0;0	0;0;0	0;0;0	0;0;0	0;0;0	0;0;0
groupI	6550000	6552000	1;2;	3;63;	3;0;3	0;0;0	0;0;0	0;0;0	0;0;0	0;0;0	0;0;0	1;0;1	1;0;1	0;0;0	1;0;1	0;0;0
groupI	6741500	6747000	1;2;	1;65;	1;0;1	1;0;1	0;0;0	0;0;0	0;0;0	0;0;0	0;0;0	0;0;0	0;0;0	0;0;0	0;0;0	0;0;0
groupI	7051500	7064500	2;3;	65;1;	1;0;1	0;0;0	0;0;0	0;0;0	0;0;0	1;0;1	0;0;0	0;0;0	0;0;0	0;0;0	0;0;0	0;0;0
groupI	7113000	7207500	2;3;	65;1;	1;0;1	0;0;0	0;0;0	0;0;0	0;0;0	0;0;0	0;0;0	0;0;0	0;0;0	0;0;0	1;0;1	0;0;0
groupI	7263000	7265000	1;2;	2;64;	2;0;2	1;0;1	0;0;0	1;0;1	0;0;0	0;0;0	0;0;0	0;0;0	0;0;0	0;0;0	0;0;0	0;0;0
groupI	7314000	7318500	1;2;	1;65;	1;0;1	0;0;0	0;0;0	0;0;0	0;0;0	0;0;0	0;0;0	0;0;0	1;0;1	0;0;0	0;0;0	0;0;0
groupI	7759500	7761000	0;1;2;	3;4;59;	10;3;4	0;0;0	0;0;0	0;0;0	0;0;0	0;0;0	9;3;3	1;0;1	0;0;0	0;0;0	0;0;0	0;0;0
groupI	7890500	7894000	2;3;	44;22;	22;0;22	0;0;0	0;0;0	0;0;0	0;0;0	0;0;0	0;0;0	0;0;0	5;0;5	5;0;5	6;0;6	6;0;6
groupI	8157000	8159000	2;3;	65;1;	1;0;1	0;0;0	0;0;0	0;0;0	1;0;1	0;0;0	0;0;0	0;0;0	0;0;0	0;0;0	0;0;0	0;0;0
groupI	8170000	8172000	0;1;2;	1;3;62;	5;1;3	3;1;1	0;0;0	0;0;0	1;0;1	0;0;0	0;0;0	0;0;0	0;0;0	1;0;1	0;0;0	0;0;0
groupI	8183000	8185500	1;2;	14;52;	14;0;14	0;0;0	0;0;0	0;0;0	0;0;0	0;0;0	0;0;0	1;0;1	1;0;1	0;0;0	6;0;6	6;0;6
groupI	9350500	9352500	1;2;	2;64;	2;0;2	0;0;0	0;0;0	1;0;1	0;0;0	1;0;1	0;0;0	0;0;0	0;0;0	0;0;0	0;0;0	0;0;0
groupI	9736000	9739500	1;2;	3;63;	3;0;3	0;0;0	0;0;0	0;0;0	3;0;3	0;0;0	0;0;0	0;0;0	0;0;0	0;0;0	0;0;0	0;0;0
groupI	10048500	10070000	2;3;	65;1;	1;0;1	0;0;0	0;0;0	0;0;0	0;0;0	0;0;0	0;0;0	1;0;1	0;0;0	0;0;0	0;0;0	0;0;0
groupI	10667000	10669000	0;1;2;	5;6;55;	16;5;6	0;0;0	0;0;0	0;0;0	0;0;0	0;0;0	0;0;0	0;0;0	0;0;0	0;0;0	10;4;2	6;1;4
groupI	10687500	10737500	1;2;	2;64;	2;0;2	1;0;1	0;0;0	0;0;0	0;0;0	0;0;0	0;0;0	0;0;0	0;0;0	1;0;1	0;0;0	0;0;0
groupI	11300000	11309500	2;3;	65;1;	1;0;1	0;0;0	0;0;0	0;0;0	0;0;0	0;0;0	0;0;0	0;0;0	1;0;1	0;0;0	0;0;0	0;0;0
groupI	11341000	11343000	1;2;	12;54;	12;0;12	0;0;0	0;0;0	0;0;0	0;0;0	0;0;0	0;0;0	0;0;0	0;0;0	0;0;0	6;0;6	6;0;6
groupI	11422500	11427000	1;2;	2;64;	2;0;2	1;0;1	0;0;0	0;0;0	0;0;0	0;0;0	0;0;0	0;0;0	0;0;0	0;0;0	0;0;0	1;0;1
groupI	11429000	11437500	2;3;	64;2;	2;0;2	0;0;0	0;0;0	2;0;2	0;0;0	0;0;0	0;0;0	0;0;0	0;0;0	0;0;0	0;0;0	0;0;0
groupI	11996000	11997000	0;1;2;	1;2;63;	4;1;2	0;0;0	0;0;0	0;0;0	0;0;0	0;0;0	0;0;0	3;1;1	1;0;1	0;0;0	0;0;0	0;0;0
groupI	12065000	12069000	1;2;	1;65;	1;0;1	1;0;1	0;0;0	0;0;0	0;0;0	0;0;0	0;0;0	0;0;0	0;0;0	0;0;0	0;0;0	0;0;0
groupI	12070000	12074000	1;2;	1;65;	1;0;1	1;0;1	0;0;0	0;0;0	0;0;0	0;0;0	0;0;0	0;0;0	0;0;0	0;0;0	0;0;0	0;0;0
groupI	12430000	12457500	2;3;	65;1;	1;0;1	0;0;0	1;0;1	0;0;0	0;0;0	0;0;0	0;0;0	0;0;0	0;0;0	0;0;0	0;0;0	0;0;0
groupI	12459000	12463500	2;3;4;	48;11;7;	25;7;11	0;0;0	0;0;0	0;0;0	0;0;0	0;0;0	0;0;0	0;0;0	5;0;5	1;0;1	9;3;3	10;4;2
groupI	13179000	13185000	0;1;2;	1;3;62;	5;1;3	1;0;1	0;0;0	0;0;0	0;0;0	0;0;0	0;0;0	4;1;2	0;0;0	0;0;0	0;0;0	0;0;0
groupI	13302500	13305000	1;2;	1;65;	1;0;1	0;0;0	0;0;0	1;0;1	0;0;0	0;0;0	0;0;0	0;0;0	0;0;0	0;0;0	0;0;0	0;0;0
groupI	13643500	13647000	1;2;	5;61;	5;0;5	0;0;0	0;0;0	0;0;0	0;0;0	1;0;1	0;0;0	0;0;0	2;0;2	0;0;0	1;0;1	1;0;1
groupI	14085000	14092000	2;3;	65;1;	1;0;1	0;0;0	0;0;0	0;0;0	0;0;0	1;0;1	0;0;0	0;0;0	0;0;0	0;0;0	0;0;0	0;0;0
groupI	14803500	14815000	2;3;	65;1;	1;0;1	0;0;0	0;0;0	0;0;0	0;0;0	1;0;1	0;0;0	0;0;0	0;0;0	0;0;0	0;0;0	0;0;0
groupI	14879000	14881500	0;1;2;	2;5;59;	9;2;5	0;0;0	0;0;0	0;0;0	0;0;0	0;0;0	0;0;0	0;0;0	0;0;0	0;0;0	8;2;4	1;0;1

groupI	15091500	15096000	1;2;	1;65;	1;0;1	0;0;0	0;0;0	0;0;0	0;0;0	1;0;1	0;0;0	0;0;0	0;0;0	0;0;0	0;0;0	0;0;0
groupI	15304500	15306500	1;2;	3;63;	3;0;3	0;0;0	0;0;0	0;0;0	0;0;0	0;0;0	0;0;0	0;0;0	3;0;3	0;0;0	0;0;0	0;0;0
groupI	15588000	15591500	0;1;2;	1;16;49;	18;1;16	3;1;1	4;0;4	5;0;5	0;0;0	2;0;2	3;0;3	1;0;1	0;0;0	0;0;0	0;0;0	0;0;0
groupI	15606500	15610000	1;2;	1;65;	1;0;1	0;0;0	0;0;0	0;0;0	0;0;0	0;0;0	0;0;0	0;0;0	0;0;0	0;0;0	1;0;1	0;0;0
groupI	15737000	15739000	1;2;	1;65;	1;0;1	0;0;0	0;0;0	1;0;1	0;0;0	0;0;0	0;0;0	0;0;0	0;0;0	0;0;0	0;0;0	0;0;0
groupI	15747500	15749500	1;2;	1;65;	1;0;1	0;0;0	0;0;0	0;0;0	0;0;0	1;0;1	0;0;0	0;0;0	0;0;0	0;0;0	0;0;0	0;0;0
groupI	16145000	16147500	1;2;	1;65;	1;0;1	0;0;0	0;0;0	0;0;0	0;0;0	0;0;0	0;0;0	0;0;0	0;0;0	0;0;0	0;0;0	1;0;1
groupI	16212500	16215500	1;2;	6;60;	6;0;6	0;0;0	0;0;0	0;0;0	0;0;0	0;0;0	0;0;0	0;0;0	4;0;4	1;0;1	1;0;1	0;0;0
groupI	16410000	16412000	0;1;2;	2;8;56;	12;2;8	4;0;4	3;1;1	2;1;0	0;0;0	2;0;2	0;0;0	1;0;1	0;0;0	0;0;0	0;0;0	0;0;0
groupI	16438500	16466500	2;3;	62;4;	4;0;4	0;0;0	0;0;0	3;0;3	0;0;0	1;0;1	0;0;0	0;0;0	0;0;0	0;0;0	0;0;0	0;0;0
groupI	16493500	16502000	2;3;	65;1;	1;0;1	0;0;0	0;0;0	0;0;0	0;0;0	0;0;0	0;0;0	0;0;0	0;0;0	1;0;1	0;0;0	0;0;0
groupI	16795500	16810000	2;3;	65;1;	1;0;1	0;0;0	0;0;0	0;0;0	0;0;0	0;0;0	0;0;0	1;0;1	0;0;0	0;0;0	0;0;0	0;0;0
groupI	17204500	17210000	1;2;	1;65;	1;0;1	0;0;0	0;0;0	0;0;0	0;0;0	0;0;0	0;0;0	0;0;0	0;0;0	1;0;1	0;0;0	0;0;0
groupI	17379000	17420500	2;3;	54;12;	12;0;12	1;0;1	0;0;0	1;0;1	0;0;0	0;0;0	0;0;0	2;0;2	4;0;4	4;0;4	0;0;0	0;0;0
groupI	17585500	17591500	2;3;4;	59;5;2;	9;2;5	0;0;0	0;0;0	0;0;0	0;0;0	0;0;0	8;2;4	1;0;1	0;0;0	0;0;0	0;0;0	0;0;0
groupI	17630000	17633000	1;2;	7;59;	7;0;7	1;0;1	0;0;0	4;0;4	0;0;0	2;0;2	0;0;0	0;0;0	0;0;0	0;0;0	0;0;0	0;0;0
groupI	17743500	17766500	2;3;	64;2;	2;0;2	0;0;0	0;0;0	0;0;0	0;0;0	0;0;0	0;0;0	0;0;0	2;0;2	0;0;0	0;0;0	0;0;0
groupI	17802000	17811500	2;3;	63;3;	3;0;3	0;0;0	0;0;0	3;0;3	0;0;0	0;0;0	0;0;0	0;0;0	0;0;0	0;0;0	0;0;0	0;0;0
groupI	17967000	17970000	1;2;	3;63;	3;0;3	0;0;0	0;0;0	3;0;3	0;0;0	0;0;0	0;0;0	0;0;0	0;0;0	0;0;0	0;0;0	0;0;0
groupI	18218000	18226000	2;3;	65;1;	1;0;1	0;0;0	0;0;0	0;0;0	0;0;0	0;0;0	0;0;0	0;0;0	1;0;1	0;0;0	0;0;0	0;0;0
groupI	18991000	19000500	2;3;	65;1;	1;0;1	1;0;1	0;0;0	0;0;0	0;0;0	0;0;0	0;0;0	0;0;0	0;0;0	0;0;0	0;0;0	0;0;0
groupI	19023500	19027000	0;1;2;	1;6;59;	8;1;6	0;0;0	4;1;2	1;0;1	2;0;2	1;0;1	0;0;0	0;0;0	0;0;0	0;0;0	0;0;0	0;0;0
groupI	19110000	19113000	1;2;	2;64;	2;0;2	0;0;0	0;0;0	0;0;0	0;0;0	0;0;0	0;0;0	0;0;0	1;0;1	1;0;1	0;0;0	0;0;0
groupI	19136000	19137500	1;2;	8;58;	8;0;8	0;0;0	0;0;0	0;0;0	0;0;0	0;0;0	0;0;0	0;0;0	5;0;5	3;0;3	0;0;0	0;0;0
groupI	19171500	19175500	0;1;2;	1;7;58;	9;1;7	0;0;0	0;0;0	0;0;0	0;0;0	0;0;0	0;0;0	0;0;0	6;1;4	3;0;3	0;0;0	0;0;0
groupI	19275500	19298500	1;2;	3;63;	3;0;3	0;0;0	1;0;1	0;0;0	2;0;2	0;0;0	0;0;0	0;0;0	0;0;0	0;0;0	0;0;0	0;0;0
groupI	19434500	19438500	1;2;	1;65;	1;0;1	0;0;0	0;0;0	0;0;0	1;0;1	0;0;0	0;0;0	0;0;0	0;0;0	0;0;0	0;0;0	0;0;0
groupI	19645500	19647000	0;1;2;	6;8;52;	20;6;8	0;0;0	0;0;0	0;0;0	0;0;0	0;0;0	0;0;0	0;0;0	2;0;2	1;0;1	7;2;3	10;4;2
groupI	19903500	19920000	2;3;	65;1;	1;0;1	0;0;0	0;0;0	0;0;0	0;0;0	0;0;0	0;0;0	0;0;0	0;0;0	0;0;0	0;0;0	1;0;1
groupI	20002000	20005000	1;2;	1;65;	1;0;1	0;0;0	0;0;0	0;0;0	0;0;0	0;0;0	0;0;0	0;0;0	0;0;0	1;0;1	0;0;0	0;0;0
groupI	20009500	20021500	1;2;	5;61;	5;0;5	0;0;0	4;0;4	0;0;0	1;0;1	0;0;0	0;0;0	0;0;0	0;0;0	0;0;0	0;0;0	0;0;0
groupI	20110000	20112000	1;2;	5;61;	5;0;5	0;0;0	0;0;0	1;0;1	3;0;3	1;0;1	0;0;0	0;0;0	0;0;0	0;0;0	0;0;0	0;0;0
groupI	20212500	20221000	2;3;	65;1;	1;0;1	0;0;0	0;0;0	0;0;0	0;0;0	1;0;1	0;0;0	0;0;0	0;0;0	0;0;0	0;0;0	0;0;0
groupI	20402500	20405000	1;2;	2;64;	2;0;2	0;0;0	0;0;0	0;0;0	0;0;0	0;0;0	0;0;0	0;0;0	1;0;1	1;0;1	0;0;0	0;0;0

groupI	20969500	20971000	1;2;	2;64;	2;0;2	0;0;0	0;0;0	0;0;0	1;0;1	0;0;0	0;0;0	0;0;0	1;0;1	0;0;0	0;0;0	0;0;0
groupI	21201500	21203000	1;2;	8;58;	8;0;8	1;0;1	0;0;0	5;0;5	1;0;1	1;0;1	0;0;0	0;0;0	0;0;0	0;0;0	0;0;0	0;0;0
groupI	21309000	21314500	2;3;	65;1;	1;0;1	0;0;0	0;0;0	0;0;0	0;0;0	0;0;0	0;0;0	0;0;0	0;0;0	0;0;0	0;0;0	1;0;1
groupI	21343500	21348500	1;2;	1;65;	1;0;1	0;0;0	0;0;0	0;0;0	0;0;0	0;0;0	0;0;0	0;0;0	1;0;1	0;0;0	0;0;0	0;0;0
groupI	21477000	21479000	1;2;	14;52;	14;0;14	0;0;0	0;0;0	0;0;0	0;0;0	0;0;0	0;0;0	0;0;0	2;0;2	4;0;4	2;0;2	6;0;6
groupI	21480500	21490000	1;2;	1;65;	1;0;1	0;0;0	0;0;0	0;0;0	0;0;0	0;0;0	0;0;0	0;0;0	1;0;1	0;0;0	0;0;0	0;0;0
groupI	21693500	21698000	1;2;	4;62;	4;0;4	4;0;4	0;0;0	0;0;0	0;0;0	0;0;0	0;0;0	0;0;0	0;0;0	0;0;0	0;0;0	0;0;0
groupI	21714000	21718000	1;2;	2;64;	2;0;2	2;0;2	0;0;0	0;0;0	0;0;0	0;0;0	0;0;0	0;0;0	0;0;0	0;0;0	0;0;0	0;0;0
groupI	22157500	22164000	2;3;	64;2;	2;0;2	1;0;1	0;0;0	0;0;0	0;0;0	0;0;0	0;0;0	1;0;1	0;0;0	0;0;0	0;0;0	0;0;0
groupI	22295000	22353000	2;3;	55;11;	11;0;11	0;0;0	0;0;0	0;0;0	0;0;0	0;0;0	0;0;0	1;0;1	2;0;2	4;0;4	2;0;2	2;0;2
groupI	23091000	23093500	1;2;	1;65;	1;0;1	0;0;0	0;0;0	0;0;0	0;0;0	0;0;0	0;0;0	1;0;1	0;0;0	0;0;0	0;0;0	0;0;0
groupI	23349000	23359500	2;3;	65;1;	1;0;1	0;0;0	0;0;0	0;0;0	0;0;0	0;0;0	0;0;0	0;0;0	0;0;0	0;0;0	1;0;1	0;0;0
groupI	23665500	23678500	2;3;	65;1;	1;0;1	0;0;0	0;0;0	0;0;0	0;0;0	0;0;0	0;0;0	0;0;0	1;0;1	0;0;0	0;0;0	0;0;0
groupI	24160500	24162500	1;2;	2;64;	2;0;2	2;0;2	0;0;0	0;0;0	0;0;0	0;0;0	0;0;0	0;0;0	0;0;0	0;0;0	0;0;0	0;0;0
groupI	24176500	24178000	1;2;	3;63;	3;0;3	0;0;0	0;0;0	0;0;0	3;0;3	0;0;0	0;0;0	0;0;0	0;0;0	0;0;0	0;0;0	0;0;0
groupI	24741500	24750000	0;1;2;	2;2;62;	6;2;2	0;0;0	0;0;0	5;2;1	0;0;0	1;0;1	0;0;0	0;0;0	0;0;0	0;0;0	0;0;0	0;0;0
groupI	24832000	24838000	1;2;	1;65;	1;0;1	0;0;0	0;0;0	0;0;0	0;0;0	0;0;0	0;0;0	1;0;1	0;0;0	0;0;0	0;0;0	0;0;0
groupI	24885000	24886500	1;2;	16;50;	16;0;16	0;0;0	0;0;0	0;0;0	0;0;0	0;0;0	0;0;0	1;0;1	3;0;3	2;0;2	5;0;5	5;0;5
groupI	25097500	25098500	0;1;2;	4;5;57;	13;4;5	0;0;0	3;1;1	6;3;0	3;0;3	0;0;0	0;0;0	0;0;0	0;0;0	0;0;0	0;0;0	1;0;1
groupI	25207500	25209500	1;2;	1;65;	1;0;1	0;0;0	1;0;1	0;0;0	0;0;0	0;0;0	0;0;0	0;0;0	0;0;0	0;0;0	0;0;0	0;0;0
groupI	25233000	25236000	1;2;	2;64;	2;0;2	0;0;0	1;0;1	0;0;0	0;0;0	0;0;0	0;0;0	0;0;0	1;0;1	0;0;0	0;0;0	0;0;0
groupI	25951000	25953500	1;2;	1;65;	1;0;1	0;0;0	0;0;0	0;0;0	0;0;0	0;0;0	0;0;0	1;0;1	0;0;0	0;0;0	0;0;0	0;0;0
groupI	25964000	25968000	1;2;	1;65;	1;0;1	0;0;0	0;0;0	0;0;0	1;0;1	0;0;0	0;0;0	0;0;0	0;0;0	0;0;0	0;0;0	0;0;0
groupI	25979000	25983500	1;2;	1;65;	1;0;1	0;0;0	0;0;0	0;0;0	0;0;0	0;0;0	0;0;0	1;0;1	0;0;0	0;0;0	0;0;0	0;0;0
groupI	26007000	26010000	0;1;2;	3;12;51;	18;3;12	0;0;0	0;0;0	0;0;0	0;0;0	0;0;0	0;0;0	0;0;0	3;1;1	6;1;4	2;0;2	7;1;5
groupI	26478500	26485000	2;3;	65;1;	1;0;1	1;0;1	0;0;0	0;0;0	0;0;0	0;0;0	0;0;0	0;0;0	0;0;0	0;0;0	0;0;0	0;0;0
groupI	26536000	26553500	2;3;	58;8;	8;0;8	0;0;0	0;0;0	0;0;0	0;0;0	0;0;0	0;0;0	0;0;0	0;0;0	1;0;1	6;0;6	1;0;1
groupI	26661500	26665000	1;2;	1;65;	1;0;1	0;0;0	0;0;0	0;0;0	0;0;0	0;0;0	0;0;0	0;0;0	0;0;0	1;0;1	0;0;0	0;0;0
groupI	26734500	26741500	1;2;	19;47;	19;0;19	0;0;0	0;0;0	0;0;0	0;0;0	0;0;0	0;0;0	0;0;0	5;0;5	5;0;5	6;0;6	3;0;3
groupI	27201500	27204500	0;1;2;	1;3;62;	5;1;3	0;0;0	0;0;0	0;0;0	0;0;0	0;0;0	0;0;0	0;0;0	0;0;0	0;0;0	1;0;1	4;1;2
groupI	27570500	27585000	2;3;	63;3;	3;0;3	0;0;0	0;0;0	0;0;0	0;0;0	3;0;3	0;0;0	0;0;0	0;0;0	0;0;0	0;0;0	0;0;0
groupI	27646500	27662500	2;3;4;	45;18;3;	24;3;18	0;0;0	0;0;0	0;0;0	0;0;0	3;0;3	0;0;0	0;0;0	5;0;5	9;3;3	6;0;6	1;0;1
groupI	27753500	27784000	2;3;	45;21;	21;0;21	0;0;0	0;0;0	4;0;4	0;0;0	2;0;2	0;0;0	0;0;0	2;0;2	2;0;2	5;0;5	6;0;6
groupI	28103500	28120500	1;2;	1;65;	1;0;1	0;0;0	0;0;0	0;0;0	0;0;0	0;0;0	0;0;0	0;0;0	0;0;0	1;0;1	0;0;0	0;0;0

groupII	97500	98500	0;1;2;	3;16;47;	22;3;16	1;0;1	0;0;0	0;0;0	0;0;0	1;0;1	0;0;0	0;0;0	3;0;3	8;2;4	7;1;5	2;0;2
groupII	117500	120000	1;2;	3;63;	3;0;3	0;0;0	0;0;0	3;0;3	0;0;0	0;0;0	0;0;0	0;0;0	0;0;0	0;0;0	0;0;0	0;0;0
groupII	384500	386000	1;2;	5;61;	5;0;5	0;0;0	0;0;0	3;0;3	0;0;0	1;0;1	0;0;0	0;0;0	0;0;0	1;0;1	0;0;0	0;0;0
groupII	437000	440000	0;1;2;	2;10;54;	14;2;10	0;0;0	0;0;0	0;0;0	0;0;0	0;0;0	0;0;0	0;0;0	4;0;4	0;0;0	8;2;4	2;0;2
groupII	534500	537000	0;1;2;	1;3;62;	5;1;3	0;0;0	1;0;1	0;0;0	4;1;2	0;0;0	0;0;0	0;0;0	0;0;0	0;0;0	0;0;0	0;0;0
groupII	924000	937000	2;3;	65;1;	1;0;1	0;0;0	0;0;0	0;0;0	0;0;0	0;0;0	0;0;0	0;0;0	0;0;0	0;0;0	1;0;1	0;0;0
groupII	978500	982000	1;2;	3;63;	3;0;3	0;0;0	0;0;0	0;0;0	0;0;0	0;0;0	0;0;0	0;0;0	0;0;0	0;0;0	0;0;0	3;0;3
groupII	1055000	1057000	0;1;2;	3;6;57;	12;3;6	0;0;0	0;0;0	0;0;0	0;0;0	0;0;0	0;0;0	0;0;0	1;0;1	2;0;2	9;3;3	0;0;0
groupII	1506000	1508000	0;2;	1;65;	2;1;0	0;0;0	0;0;0	0;0;0	0;0;0	2;1;0	0;0;0	0;0;0	0;0;0	0;0;0	0;0;0	0;0;0
groupII	1854500	1855500	0;2;	1;65;	2;1;0	0;0;0	0;0;0	0;0;0	2;1;0	0;0;0	0;0;0	0;0;0	0;0;0	0;0;0	0;0;0	0;0;0
groupII	1947000	1951500	1;2;	1;65;	1;0;1	0;0;0	0;0;0	0;0;0	0;0;0	0;0;0	0;0;0	0;0;0	1;0;1	0;0;0	0;0;0	0;0;0
groupII	2082000	2084500	1;2;	8;58;	8;0;8	0;0;0	0;0;0	0;0;0	0;0;0	0;0;0	0;0;0	0;0;0	2;0;2	3;0;3	2;0;2	1;0;1
groupII	2117500	2119000	1;2;	1;65;	1;0;1	0;0;0	0;0;0	0;0;0	0;0;0	0;0;0	0;0;0	0;0;0	0;0;0	0;0;0	0;0;0	1;0;1
groupII	2511000	2521000	2;3;	65;1;	1;0;1	0;0;0	0;0;0	0;0;0	0;0;0	1;0;1	0;0;0	0;0;0	0;0;0	0;0;0	0;0;0	0;0;0
groupII	2558000	2565000	2;3;	65;1;	1;0;1	1;0;1	0;0;0	0;0;0	0;0;0	0;0;0	0;0;0	0;0;0	0;0;0	0;0;0	0;0;0	0;0;0
groupII	2865500	2867500	0;1;2;	2;8;56;	12;2;8	0;0;0	1;0;1	3;0;3	5;1;3	3;1;1	0;0;0	0;0;0	0;0;0	0;0;0	0;0;0	0;0;0
groupII	2969500	3001500	2;3;	65;1;	1;0;1	0;0;0	0;0;0	0;0;0	0;0;0	1;0;1	0;0;0	0;0;0	0;0;0	0;0;0	0;0;0	0;0;0
groupII	2985000	2989500	1;2;	5;61;	5;0;5	1;0;1	3;0;3	0;0;0	0;0;0	0;0;0	0;0;0	0;0;0	0;0;0	1;0;1	0;0;0	0;0;0
groupII	3303000	3304500	0;1;2;	6;1;59;	13;6;1	0;0;0	0;0;0	0;0;0	0;0;0	0;0;0	0;0;0	0;0;0	0;0;0	0;0;0	12;6;0	1;0;1
groupII	3305500	3308000	1;2;	3;63;	3;0;3	0;0;0	0;0;0	1;0;1	1;0;1	0;0;0	0;0;0	0;0;0	0;0;0	1;0;1	0;0;0	0;0;0
groupII	3371500	3379500	2;3;	64;2;	2;0;2	0;0;0	0;0;0	0;0;0	0;0;0	0;0;0	0;0;0	0;0;0	2;0;2	0;0;0	0;0;0	0;0;0
groupII	3590000	3601500	1;2;	2;64;	2;0;2	0;0;0	0;0;0	0;0;0	0;0;0	0;0;0	0;0;0	0;0;0	1;0;1	1;0;1	0;0;0	0;0;0
groupII	3999000	4011000	2;3;	65;1;	1;0;1	1;0;1	0;0;0	0;0;0	0;0;0	0;0;0	0;0;0	0;0;0	0;0;0	0;0;0	0;0;0	0;0;0
groupII	4110500	4112500	1;2;	1;65;	1;0;1	0;0;0	0;0;0	0;0;0	0;0;0	0;0;0	0;0;0	0;0;0	0;0;0	0;0;0	0;0;0	1;0;1
groupII	4127500	4132000	1;2;	7;59;	7;0;7	0;0;0	0;0;0	0;0;0	0;0;0	0;0;0	5;0;5	2;0;2	0;0;0	0;0;0	0;0;0	0;0;0
groupII	5307500	5311500	1;2;	1;65;	1;0;1	0;0;0	0;0;0	0;0;0	1;0;1	0;0;0	0;0;0	0;0;0	0;0;0	0;0;0	0;0;0	0;0;0
groupII	5515500	5516500	0;1;2;	1;5;60;	7;1;5	0;0;0	1;0;1	4;1;2	1;0;1	1;0;1	0;0;0	0;0;0	0;0;0	0;0;0	0;0;0	0;0;0
groupII	6072500	6081500	1;2;	1;65;	1;0;1	0;0;0	0;0;0	0;0;0	0;0;0	0;0;0	0;0;0	1;0;1	0;0;0	0;0;0	0;0;0	0;0;0
groupII	6198500	6204000	1;2;	3;63;	3;0;3	0;0;0	1;0;1	0;0;0	0;0;0	0;0;0	0;0;0	0;0;0	1;0;1	1;0;1	0;0;0	0;0;0
groupII	6497500	6507500	2;3;	62;4;	4;0;4	1;0;1	0;0;0	2;0;2	0;0;0	1;0;1	0;0;0	0;0;0	0;0;0	0;0;0	0;0;0	0;0;0
groupII	6861000	6872000	1;2;	4;62;	4;0;4	0;0;0	0;0;0	0;0;0	0;0;0	0;0;0	4;0;4	0;0;0	0;0;0	0;0;0	0;0;0	0;0;0
groupII	7028500	7029500	0;1;2;	1;2;63;	4;1;2	0;0;0	0;0;0	3;1;1	0;0;0	1;0;1	0;0;0	0;0;0	0;0;0	0;0;0	0;0;0	0;0;0
groupII	7237500	7243500	2;3;	65;1;	1;0;1	0;0;0	1;0;1	0;0;0	0;0;0	0;0;0	0;0;0	0;0;0	0;0;0	0;0;0	0;0;0	0;0;0
groupII	7457000	7460000	0;1;2;	3;22;41;	28;3;22	5;1;3	6;0;6	1;0;1	5;1;3	2;0;2	1;0;1	0;0;0	6;1;4	0;0;0	0;0;0	2;0;2

groupII	7581000	7582500	0;1;2;	2;7;57;	11;2;7	1;0;1	0;0;0	4;1;2	4;1;2	1;0;1	0;0;0	0;0;0	1;0;1	0;0;0	0;0;0	0;0;0
groupII	7587000	7590000	0;1;2;	5;5;56;	15;5;5	0;0;0	0;0;0	0;0;0	0;0;0	0;0;0	0;0;0	0;0;0	0;0;0	0;0;0	9;3;3	6;2;2
groupII	9358500	9362500	1;2;	11;55;	11;0;11	1;0;1	0;0;0	1;0;1	0;0;0	0;0;0	0;0;0	0;0;0	0;0;0	0;0;0	6;0;6	3;0;3
groupII	9465000	9468000	0;2;	1;65;	2;1;0	0;0;0	0;0;0	0;0;0	2;1;0	0;0;0	0;0;0	0;0;0	0;0;0	0;0;0	0;0;0	0;0;0
groupII	10197500	10206000	2;3;	65;1;	1;0;1	1;0;1	0;0;0	0;0;0	0;0;0	0;0;0	0;0;0	0;0;0	0;0;0	0;0;0	0;0;0	0;0;0
groupII	10448500	10458000	2;3;	65;1;	1;0;1	1;0;1	0;0;0	0;0;0	0;0;0	0;0;0	0;0;0	0;0;0	0;0;0	0;0;0	0;0;0	0;0;0
groupII	11310000	11315500	2;3;	65;1;	1;0;1	0;0;0	0;0;0	0;0;0	0;0;0	0;0;0	0;0;0	0;0;0	1;0;1	0;0;0	0;0;0	0;0;0
groupII	11364000	11371500	2;3;	65;1;	1;0;1	0;0;0	0;0;0	0;0;0	0;0;0	0;0;0	0;0;0	0;0;0	0;0;0	1;0;1	0;0;0	0;0;0
groupII	11733500	11744000	2;3;4;	54;3;9;	21;9;3	0;0;0	12;6;0	0;0;0	8;3;2	1;0;1	0;0;0	0;0;0	0;0;0	0;0;0	0;0;0	0;0;0
groupII	12294000	12295500	0;1;2;	5;7;54;	17;5;7	0;0;0	0;0;0	0;0;0	0;0;0	0;0;0	0;0;0	0;0;0	0;0;0	0;0;0	8;2;4	9;3;3
groupII	12941500	12944500	1;2;	1;65;	1;0;1	0;0;0	0;0;0	0;0;0	1;0;1	0;0;0	0;0;0	0;0;0	0;0;0	0;0;0	0;0;0	0;0;0
groupII	13408000	13422000	2;3;	61;5;	5;0;5	0;0;0	1;0;1	2;0;2	1;0;1	1;0;1	0;0;0	0;0;0	0;0;0	0;0;0	0;0;0	0;0;0
groupII	13487500	13494000	2;3;	64;2;	2;0;2	0;0;0	0;0;0	2;0;2	0;0;0	0;0;0	0;0;0	0;0;0	0;0;0	0;0;0	0;0;0	0;0;0
groupII	13532500	13535500	0;1;2;	4;2;60;	10;4;2	0;0;0	0;0;0	0;0;0	0;0;0	0;0;0	0;0;0	0;0;0	0;0;0	0;0;0	10;4;2	0;0;0
groupII	14001500	14008000	2;3;	65;1;	1;0;1	0;0;0	0;0;0	0;0;0	0;0;0	1;0;1	0;0;0	0;0;0	0;0;0	0;0;0	0;0;0	0;0;0
groupII	14544000	14575000	2;3;4;	44;18;4;	26;4;18	0;0;0	0;0;0	0;0;0	0;0;0	0;0;0	0;0;0	0;0;0	7;2;3	7;2;3	6;0;6	6;0;6
groupII	14828500	14832500	1;2;	1;65;	1;0;1	0;0;0	0;0;0	0;0;0	0;0;0	0;0;0	0;0;0	1;0;1	0;0;0	0;0;0	0;0;0	0;0;0
groupII	14840500	14843000	1;2;	2;64;	2;0;2	0;0;0	0;0;0	0;0;0	0;0;0	0;0;0	0;0;0	0;0;0	2;0;2	0;0;0	0;0;0	0;0;0
groupII	15776000	15792000	1;2;	8;58;	8;0;8	0;0;0	0;0;0	0;0;0	0;0;0	0;0;0	0;0;0	0;0;0	2;0;2	0;0;0	5;0;5	1;0;1
groupII	16955500	16958000	1;2;	3;63;	3;0;3	0;0;0	3;0;3	0;0;0	0;0;0	0;0;0	0;0;0	0;0;0	0;0;0	0;0;0	0;0;0	0;0;0
groupII	17058500	17060500	1;2;	2;64;	2;0;2	0;0;0	0;0;0	1;0;1	0;0;0	1;0;1	0;0;0	0;0;0	0;0;0	0;0;0	0;0;0	0;0;0
groupII	17407500	17409500	1;2;	2;64;	2;0;2	0;0;0	0;0;0	0;0;0	0;0;0	0;0;0	0;0;0	0;0;0	1;0;1	0;0;0	1;0;1	0;0;0
groupII	18004000	18013000	2;3;	65;1;	1;0;1	0;0;0	0;0;0	0;0;0	0;0;0	0;0;0	0;0;0	0;0;0	0;0;0	1;0;1	0;0;0	0;0;0
groupII	18511500	18523500	2;3;	65;1;	1;0;1	0;0;0	0;0;0	0;0;0	0;0;0	1;0;1	0;0;0	0;0;0	0;0;0	0;0;0	0;0;0	0;0;0
groupII	18948500	18961500	2;3;	65;1;	1;0;1	0;0;0	0;0;0	0;0;0	0;0;0	0;0;0	0;0;0	1;0;1	0;0;0	0;0;0	0;0;0	0;0;0
groupII	19354000	19356000	0;1;2;	1;9;56;	11;1;9	0;0;0	1;0;1	0;0;0	7;1;5	3;0;3	0;0;0	0;0;0	0;0;0	0;0;0	0;0;0	0;0;0
groupII	19395500	19408000	2;3;	64;2;	2;0;2	0;0;0	2;0;2	0;0;0	0;0;0	0;0;0	0;0;0	0;0;0	0;0;0	0;0;0	0;0;0	0;0;0
groupII	19414000	19423500	2;3;	62;4;	4;0;4	0;0;0	0;0;0	1;0;1	0;0;0	3;0;3	0;0;0	0;0;0	0;0;0	0;0;0	0;0;0	0;0;0
groupII	20130500	20135500	1;2;	1;65;	1;0;1	0;0;0	0;0;0	0;0;0	0;0;0	0;0;0	0;0;0	0;0;0	1;0;1	0;0;0	0;0;0	0;0;0
groupII	20348000	20350000	2;3;	64;2;	2;0;2	1;0;1	0;0;0	0;0;0	0;0;0	0;0;0	0;0;0	0;0;0	1;0;1	0;0;0	0;0;0	0;0;0
groupII	20485000	20491000	2;3;	64;2;	2;0;2	0;0;0	2;0;2	0;0;0	0;0;0	0;0;0	0;0;0	0;0;0	0;0;0	0;0;0	0;0;0	0;0;0
groupII	20519000	20521500	0;1;2;	1;2;63;	4;1;2	0;0;0	3;1;1	0;0;0	1;0;1	0;0;0	0;0;0	0;0;0	0;0;0	0;0;0	0;0;0	0;0;0
groupII	20545500	20548000	0;1;2;	6;3;57;	15;6;3	0;0;0	0;0;0	0;0;0	0;0;0	0;0;0	0;0;0	0;0;0	0;0;0	0;0;0	11;5;1	4;1;2
groupII	20921500	20927500	2;3;	65;1;	1;0;1	0;0;0	1;0;1	0;0;0	0;0;0	0;0;0	0;0;0	0;0;0	0;0;0	0;0;0	0;0;0	0;0;0

groupII	21296000	21301000	1;2;	2;64;	2;0;2	0;0;0	0;0;0	0;0;0	0;0;0	0;0;0	0;0;0	0;0;0	0;0;0	2;0;2	0;0;0	0;0;0
groupII	21478500	21480000	0;1;2;	1;16;49;	18;1;16	0;0;0	0;0;0	0;0;0	0;0;0	0;0;0	0;0;0	0;0;0	3;0;3	5;1;3	5;0;5	5;0;5
groupII	21504000	21529000	1;2;	13;53;	13;0;13	0;0;0	0;0;0	0;0;0	0;0;0	0;0;0	0;0;0	0;0;0	4;0;4	4;0;4	0;0;0	5;0;5
groupII	21805500	21814500	2;3;	65;1;	1;0;1	0;0;0	0;0;0	1;0;1	0;0;0	0;0;0	0;0;0	0;0;0	0;0;0	0;0;0	0;0;0	0;0;0
groupII	22103500	22116000	2;3;	63;3;	3;0;3	2;0;2	0;0;0	0;0;0	0;0;0	0;0;0	0;0;0	0;0;0	0;0;0	1;0;1	0;0;0	0;0;0
groupII	22220500	22225000	1;2;	3;63;	3;0;3	0;0;0	0;0;0	1;0;1	0;0;0	1;0;1	0;0;0	0;0;0	1;0;1	0;0;0	0;0;0	0;0;0
groupII	22320000	22322500	1;2;	1;65;	1;0;1	1;0;1	0;0;0	0;0;0	0;0;0	0;0;0	0;0;0	0;0;0	0;0;0	0;0;0	0;0;0	0;0;0
groupII	22604000	22735000	2;3;	65;1;	1;0;1	0;0;0	0;0;0	1;0;1	0;0;0	0;0;0	0;0;0	0;0;0	0;0;0	0;0;0	0;0;0	0;0;0
groupIII	509000	516000	1;2;	1;65;	1;0;1	0;0;0	0;0;0	0;0;0	0;0;0	0;0;0	0;0;0	0;0;0	1;0;1	0;0;0	0;0;0	0;0;0
groupIII	703500	710000	2;3;	65;1;	1;0;1	0;0;0	0;0;0	0;0;0	0;0;0	0;0;0	0;0;0	1;0;1	0;0;0	0;0;0	0;0;0	0;0;0
groupIII	713000	736000	2;3;	56;10;	10;0;10	0;0;0	0;0;0	0;0;0	0;0;0	0;0;0	0;0;0	0;0;0	0;0;0	0;0;0	4;0;4	6;0;6
groupIII	883500	887000	1;2;	1;65;	1;0;1	0;0;0	0;0;0	1;0;1	0;0;0	0;0;0	0;0;0	0;0;0	0;0;0	0;0;0	0;0;0	0;0;0
groupIII	1177000	1179500	1;2;	1;65;	1;0;1	0;0;0	0;0;0	0;0;0	0;0;0	0;0;0	0;0;0	0;0;0	0;0;0	0;0;0	0;0;0	1;0;1
groupIII	1300500	1310000	2;3;	65;1;	1;0;1	0;0;0	0;0;0	0;0;0	0;0;0	0;0;0	0;0;0	0;0;0	0;0;0	1;0;1	0;0;0	0;0;0
groupIII	1530000	1532000	1;2;	6;60;	6;0;6	0;0;0	0;0;0	0;0;0	3;0;3	0;0;0	0;0;0	0;0;0	1;0;1	2;0;2	0;0;0	0;0;0
groupIII	1753500	1756500	1;2;	2;64;	2;0;2	1;0;1	0;0;0	0;0;0	0;0;0	0;0;0	0;0;0	0;0;0	1;0;1	0;0;0	0;0;0	0;0;0
groupIII	1797000	1803500	2;3;	62;4;	4;0;4	0;0;0	0;0;0	1;0;1	0;0;0	3;0;3	0;0;0	0;0;0	0;0;0	0;0;0	0;0;0	0;0;0
groupIII	1889000	1891000	0;1;2;	1;3;62;	5;1;3	0;0;0	0;0;0	0;0;0	0;0;0	0;0;0	0;0;0	0;0;0	0;0;0	0;0;0	5;1;3	0;0;0
groupIII	2040000	2041500	1;2;	2;64;	2;0;2	0;0;0	0;0;0	0;0;0	0;0;0	0;0;0	0;0;0	0;0;0	0;0;0	1;0;1	1;0;1	0;0;0
groupIII	2155500	2167000	2;3;	65;1;	1;0;1	1;0;1	0;0;0	0;0;0	0;0;0	0;0;0	0;0;0	0;0;0	0;0;0	0;0;0	0;0;0	0;0;0
groupIII	2263000	2268000	1;2;	1;65;	1;0;1	0;0;0	1;0;1	0;0;0	0;0;0	0;0;0	0;0;0	0;0;0	0;0;0	0;0;0	0;0;0	0;0;0
groupIII	2378500	2381500	1;2;	2;64;	2;0;2	0;0;0	0;0;0	0;0;0	0;0;0	0;0;0	0;0;0	1;0;1	0;0;0	0;0;0	1;0;1	0;0;0
groupIII	2564500	2566500	0;1;2;	1;8;57;	10;1;8	0;0;0	1;0;1	1;0;1	5;1;3	3;0;3	0;0;0	0;0;0	0;0;0	0;0;0	0;0;0	0;0;0
groupIII	2601000	2605000	1;2;	8;58;	8;0;8	0;0;0	0;0;0	0;0;0	0;0;0	0;0;0	0;0;0	0;0;0	2;0;2	1;0;1	1;0;1	4;0;4
groupIII	2668000	2676500	2;3;	64;2;	2;0;2	0;0;0	0;0;0	0;0;0	2;0;2	0;0;0	0;0;0	0;0;0	0;0;0	0;0;0	0;0;0	0;0;0
groupIII	2779500	2782500	1;2;	1;65;	1;0;1	0;0;0	1;0;1	0;0;0	0;0;0	0;0;0	0;0;0	0;0;0	0;0;0	0;0;0	0;0;0	0;0;0
groupIII	2832500	2840000	2;3;	64;2;	2;0;2	0;0;0	0;0;0	0;0;0	0;0;0	2;0;2	0;0;0	0;0;0	0;0;0	0;0;0	0;0;0	0;0;0
groupIII	2915500	2924500	2;3;	62;4;	4;0;4	0;0;0	0;0;0	0;0;0	2;0;2	0;0;0	0;0;0	2;0;2	0;0;0	0;0;0	0;0;0	0;0;0
groupIII	3213000	3220500	2;3;	65;1;	1;0;1	0;0;0	0;0;0	0;0;0	0;0;0	1;0;1	0;0;0	0;0;0	0;0;0	0;0;0	0;0;0	0;0;0
groupIII	3310500	3316000	2;3;	65;1;	1;0;1	0;0;0	0;0;0	0;0;0	0;0;0	0;0;0	0;0;0	0;0;0	1;0;1	0;0;0	0;0;0	0;0;0
groupIII	3314500	3319000	1;2;	1;65;	1;0;1	1;0;1	0;0;0	0;0;0	0;0;0	0;0;0	0;0;0	0;0;0	0;0;0	0;0;0	0;0;0	0;0;0
groupIII	3531500	3534500	1;2;	1;65;	1;0;1	0;0;0	0;0;0	0;0;0	1;0;1	0;0;0	0;0;0	0;0;0	0;0;0	0;0;0	0;0;0	0;0;0
groupIII	3766000	3769000	1;2;	1;65;	1;0;1	0;0;0	0;0;0	0;0;0	1;0;1	0;0;0	0;0;0	0;0;0	0;0;0	0;0;0	0;0;0	0;0;0
groupIII	4157000	4158500	0;1;2;	1;12;53;	14;1;12	0;0;0	0;0;0	0;0;0	0;0;0	0;0;0	0;0;0	0;0;0	4;0;4	4;1;2	6;0;6	0;0;0

groupIII	4177500	4180000	0;1;2;	1;2;63;	4;1;2	4;1;2	0;0;0	0;0;0	0;0;0	0;0;0	0;0;0	0;0;0	0;0;0	0;0;0	0;0;0	0;0;0
groupIII	4317000	4318000	0;1;2;	2;8;56;	12;2;8	0;0;0	0;0;0	0;0;0	0;0;0	0;0;0	0;0;0	0;0;0	0;0;0	1;0;1	8;2;4	3;0;3
groupIII	4349000	4361000	2;3;	60;6;	6;0;6	0;0;0	0;0;0	0;0;0	0;0;0	0;0;0	0;0;0	0;0;0	2;0;2	2;0;2	2;0;2	0;0;0
groupIII	4368500	4373500	1;2;	2;64;	2;0;2	0;0;0	0;0;0	2;0;2	0;0;0	0;0;0	0;0;0	0;0;0	0;0;0	0;0;0	0;0;0	0;0;0
groupIII	4492500	4513000	1;2;	14;52;	14;0;14	0;0;0	0;0;0	0;0;0	0;0;0	0;0;0	0;0;0	0;0;0	1;0;1	1;0;1	6;0;6	6;0;6
groupIII	4706000	4718500	1;2;	1;65;	1;0;1	0;0;0	0;0;0	1;0;1	0;0;0	0;0;0	0;0;0	0;0;0	0;0;0	0;0;0	0;0;0	0;0;0
groupIII	4992500	4999000	2;3;	64;2;	2;0;2	0;0;0	0;0;0	1;0;1	1;0;1	0;0;0	0;0;0	0;0;0	0;0;0	0;0;0	0;0;0	0;0;0
groupIII	5086500	5101500	2;3;	63;3;	3;0;3	0;0;0	0;0;0	0;0;0	0;0;0	0;0;0	0;0;0	0;0;0	0;0;0	1;0;1	1;0;1	1;0;1
groupIII	5342000	5350000	2;3;	65;1;	1;0;1	0;0;0	1;0;1	0;0;0	0;0;0	0;0;0	0;0;0	0;0;0	0;0;0	0;0;0	0;0;0	0;0;0
groupIII	5549500	5553500	0;1;2;	1;9;56;	11;1;9	0;0;0	0;0;0	0;0;0	0;0;0	0;0;0	0;0;0	0;0;0	1;0;1	1;0;1	6;1;4	3;0;3
groupIII	5555500	5558500	1;2;	2;64;	2;0;2	0;0;0	0;0;0	0;0;0	2;0;2	0;0;0	0;0;0	0;0;0	0;0;0	0;0;0	0;0;0	0;0;0
groupIII	5606000	5607000	0;1;2;	1;6;59;	8;1;6	0;0;0	0;0;0	0;0;0	0;0;0	0;0;0	0;0;0	0;0;0	2;0;2	6;1;4	0;0;0	0;0;0
groupIII	5796000	5799500	1;2;	4;62;	4;0;4	0;0;0	1;0;1	0;0;0	2;0;2	0;0;0	0;0;0	1;0;1	0;0;0	0;0;0	0;0;0	0;0;0
groupIII	6040500	6042000	2;3;	56;10;	10;0;10	0;0;0	2;0;2	0;0;0	2;0;2	0;0;0	0;0;0	0;0;0	1;0;1	2;0;2	3;0;3	0;0;0
groupIII	6179000	6180500	1;2;	5;61;	5;0;5	0;0;0	0;0;0	0;0;0	0;0;0	0;0;0	0;0;0	0;0;0	3;0;3	2;0;2	0;0;0	0;0;0
groupIII	6380000	6381000	0;1;2;	4;5;57;	13;4;5	0;0;0	0;0;0	0;0;0	0;0;0	0;0;0	0;0;0	0;0;0	2;0;2	10;4;2	1;0;1	0;0;0
groupIII	6603000	6604500	0;1;2;	1;7;58;	9;1;7	0;0;0	0;0;0	0;0;0	0;0;0	0;0;0	0;0;0	0;0;0	3;0;3	0;0;0	0;0;0	6;1;4
groupIII	6726000	6728000	0;1;2;	4;4;58;	12;4;4	0;0;0	0;0;0	0;0;0	0;0;0	0;0;0	0;0;0	0;0;0	0;0;0	0;0;0	2;0;2	10;4;2
groupIII	7093500	7096000	2;3;	65;1;	1;0;1	0;0;0	0;0;0	0;0;0	0;0;0	0;0;0	0;0;0	0;0;0	0;0;0	1;0;1	0;0;0	0;0;0
groupIII	7576500	7587500	2;3;4;	48;16;2;	20;2;16	0;0;0	0;0;0	0;0;0	0;0;0	0;0;0	0;0;0	0;0;0	6;2;2	4;0;4	5;0;5	5;0;5
groupIII	7587000	7593500	2;3;4;	64;1;1;	3;1;1	0;0;0	0;0;0	0;0;0	0;0;0	0;0;0	0;0;0	0;0;0	0;0;0	0;0;0	0;0;0	3;1;1
groupIII	8511500	8515000	1;2;	1;65;	1;0;1	1;0;1	0;0;0	0;0;0	0;0;0	0;0;0	0;0;0	0;0;0	0;0;0	0;0;0	0;0;0	0;0;0
groupIII	8607000	8611500	0;2;	1;65;	2;1;0	0;0;0	0;0;0	0;0;0	0;0;0	0;0;0	0;0;0	0;0;0	2;1;0	0;0;0	0;0;0	0;0;0
groupIII	8817500	8836000	2;3;	65;1;	1;0;1	0;0;0	0;0;0	1;0;1	0;0;0	0;0;0	0;0;0	0;0;0	0;0;0	0;0;0	0;0;0	0;0;0
groupIII	9354500	9361000	1;2;	1;65;	1;0;1	0;0;0	0;0;0	1;0;1	0;0;0	0;0;0	0;0;0	0;0;0	0;0;0	0;0;0	0;0;0	0;0;0
groupIII	9901500	9907500	2;3;	65;1;	1;0;1	0;0;0	1;0;1	0;0;0	0;0;0	0;0;0	0;0;0	0;0;0	0;0;0	0;0;0	0;0;0	0;0;0
groupIII	10177500	10186000	2;3;	65;1;	1;0;1	0;0;0	0;0;0	0;0;0	1;0;1	0;0;0	0;0;0	0;0;0	0;0;0	0;0;0	0;0;0	0;0;0
groupIII	10194000	10196000	1;2;	4;62;	4;0;4	0;0;0	0;0;0	3;0;3	0;0;0	1;0;1	0;0;0	0;0;0	0;0;0	0;0;0	0;0;0	0;0;0
groupIII	10253500	10255500	1;2;	1;65;	1;0;1	1;0;1	0;0;0	0;0;0	0;0;0	0;0;0	0;0;0	0;0;0	0;0;0	0;0;0	0;0;0	0;0;0
groupIII	10259500	10261000	0;2;	6;60;	12;6;0	0;0;0	12;6;0	0;0;0	0;0;0	0;0;0	0;0;0	0;0;0	0;0;0	0;0;0	0;0;0	0;0;0
groupIII	10700500	10705500	2;3;	64;2;	2;0;2	0;0;0	0;0;0	0;0;0	0;0;0	0;0;0	0;0;0	2;0;2	0;0;0	0;0;0	0;0;0	0;0;0
groupIII	12023000	12025500	1;2;	2;64;	2;0;2	0;0;0	2;0;2	0;0;0	0;0;0	0;0;0	0;0;0	0;0;0	0;0;0	0;0;0	0;0;0	0;0;0
groupIII	12409000	12416000	2;3;	63;3;	3;0;3	0;0;0	3;0;3	0;0;0	0;0;0	0;0;0	0;0;0	0;0;0	0;0;0	0;0;0	0;0;0	0;0;0
groupIII	13447500	13451000	1;2;	1;65;	1;0;1	0;0;0	1;0;1	0;0;0	0;0;0	0;0;0	0;0;0	0;0;0	0;0;0	0;0;0	0;0;0	0;0;0

groupIII	13465000	13478000	1;2;	1;65;	1;0;1	0;0;0	0;0;0	0;0;0	0;0;0	0;0;0	0;0;0	1;0;1	0;0;0	0;0;0	0;0;0	0;0;0
groupIII	13765500	13767000	0;1;2;	1;1;64;	3;1;1	0;0;0	0;0;0	0;0;0	0;0;0	0;0;0	0;0;0	0;0;0	0;0;0	0;0;0	0;0;0	3;1;1
groupIII	14559500	14562500	1;2;	1;65;	1;0;1	1;0;1	0;0;0	0;0;0	0;0;0	0;0;0	0;0;0	0;0;0	0;0;0	0;0;0	0;0;0	0;0;0
groupIII	14630000	14632500	1;2;	1;65;	1;0;1	1;0;1	0;0;0	0;0;0	0;0;0	0;0;0	0;0;0	0;0;0	0;0;0	0;0;0	0;0;0	0;0;0
groupIII	14758000	14760000	1;2;	2;64;	2;0;2	0;0;0	0;0;0	0;0;0	2;0;2	0;0;0	0;0;0	0;0;0	0;0;0	0;0;0	0;0;0	0;0;0
groupIII	14780500	14781500	1;2;	8;58;	8;0;8	0;0;0	1;0;1	1;0;1	0;0;0	0;0;0	0;0;0	0;0;0	0;0;0	3;0;3	1;0;1	2;0;2
groupIII	14785000	14787000	1;2;	3;63;	3;0;3	0;0;0	0;0;0	0;0;0	0;0;0	0;0;0	0;0;0	1;0;1	2;0;2	0;0;0	0;0;0	0;0;0
groupIII	14802500	14809000	1;2;	1;65;	1;0;1	0;0;0	0;0;0	0;0;0	0;0;0	0;0;0	0;0;0	0;0;0	0;0;0	0;0;0	0;0;0	1;0;1
groupIII	14837500	14839500	1;2;	4;62;	4;0;4	0;0;0	0;0;0	3;0;3	0;0;0	0;0;0	0;0;0	0;0;0	1;0;1	0;0;0	0;0;0	0;0;0
groupIII	15892500	15912500	2;3;	65;1;	1;0;1	0;0;0	0;0;0	0;0;0	0;0;0	0;0;0	0;0;0	0;0;0	0;0;0	0;0;0	0;0;0	1;0;1
groupIII	15948000	15958500	2;3;	65;1;	1;0;1	1;0;1	0;0;0	0;0;0	0;0;0	0;0;0	0;0;0	0;0;0	0;0;0	0;0;0	0;0;0	0;0;0
groupIV	144500	161500	2;3;	64;2;	2;0;2	0;0;0	0;0;0	0;0;0	2;0;2	0;0;0	0;0;0	0;0;0	0;0;0	0;0;0	0;0;0	0;0;0
groupIV	862500	865000	1;2;	6;60;	6;0;6	0;0;0	0;0;0	0;0;0	0;0;0	0;0;0	0;0;0	1;0;1	0;0;0	1;0;1	1;0;1	3;0;3
groupIV	895500	898500	1;2;	2;64;	2;0;2	0;0;0	0;0;0	0;0;0	0;0;0	0;0;0	2;0;2	0;0;0	0;0;0	0;0;0	0;0;0	0;0;0
groupIV	1231500	1237500	1;2;	2;64;	2;0;2	0;0;0	0;0;0	0;0;0	0;0;0	0;0;0	0;0;0	0;0;0	0;0;0	1;0;1	1;0;1	0;0;0
groupIV	1536500	1537500	0;1;2;	2;14;50;	18;2;14	5;0;5	4;1;2	1;0;1	5;1;3	3;0;3	0;0;0	0;0;0	0;0;0	0;0;0	0;0;0	0;0;0
groupIV	1559000	1562500	0;1;2;	1;1;64;	3;1;1	0;0;0	0;0;0	3;1;1	0;0;0	0;0;0	0;0;0	0;0;0	0;0;0	0;0;0	0;0;0	0;0;0
groupIV	1577500	1580500	1;2;	6;60;	6;0;6	0;0;0	0;0;0	1;0;1	3;0;3	1;0;1	0;0;0	0;0;0	0;0;0	0;0;0	1;0;1	0;0;0
groupIV	1671000	1672000	0;1;2;	1;7;58;	9;1;7	0;0;0	0;0;0	1;0;1	6;1;4	2;0;2	0;0;0	0;0;0	0;0;0	0;0;0	0;0;0	0;0;0
groupIV	1770500	1780000	1;2;	1;65;	1;0;1	0;0;0	0;0;0	1;0;1	0;0;0	0;0;0	0;0;0	0;0;0	0;0;0	0;0;0	0;0;0	0;0;0
groupIV	1952500	1954000	0;1;2;	1;2;63;	4;1;2	0;0;0	0;0;0	4;1;2	0;0;0	0;0;0	0;0;0	0;0;0	0;0;0	0;0;0	0;0;0	0;0;0
groupIV	2210000	2212500	1;2;	2;64;	2;0;2	0;0;0	0;0;0	0;0;0	0;0;0	0;0;0	0;0;0	0;0;0	2;0;2	0;0;0	0;0;0	0;0;0
groupIV	2220000	2225500	1;2;	2;64;	2;0;2	0;0;0	0;0;0	0;0;0	0;0;0	0;0;0	0;0;0	0;0;0	1;0;1	1;0;1	0;0;0	0;0;0
groupIV	2383000	2400000	2;3;	65;1;	1;0;1	0;0;0	0;0;0	0;0;0	0;0;0	0;0;0	0;0;0	1;0;1	0;0;0	0;0;0	0;0;0	0;0;0
groupIV	2837000	2840000	1;2;	4;62;	4;0;4	0;0;0	0;0;0	0;0;0	0;0;0	1;0;1	3;0;3	0;0;0	0;0;0	0;0;0	0;0;0	0;0;0
groupIV	3282000	3284000	1;2;	6;60;	6;0;6	0;0;0	0;0;0	0;0;0	0;0;0	0;0;0	0;0;0	0;0;0	0;0;0	3;0;3	3;0;3	0;0;0
groupIV	3345500	3349500	1;2;	1;65;	1;0;1	0;0;0	0;0;0	0;0;0	0;0;0	0;0;0	0;0;0	0;0;0	1;0;1	0;0;0	0;0;0	0;0;0
groupIV	3558500	3566000	2;3;4;	60;5;1;	7;1;5	0;0;0	0;0;0	0;0;0	0;0;0	0;0;0	0;0;0	0;0;0	0;0;0	0;0;0	7;1;5	0;0;0
groupIV	3682000	3689000	1;2;	1;65;	1;0;1	0;0;0	0;0;0	0;0;0	0;0;0	0;0;0	0;0;0	0;0;0	1;0;1	0;0;0	0;0;0	0;0;0
groupIV	4021500	4028000	1;2;	3;63;	3;0;3	0;0;0	0;0;0	2;0;2	0;0;0	1;0;1	0;0;0	0;0;0	0;0;0	0;0;0	0;0;0	0;0;0
groupIV	4702000	4703000	0;1;2;	1;10;55;	12;1;10	0;0;0	0;0;0	0;0;0	0;0;0	0;0;0	0;0;0	0;0;0	5;1;3	4;0;4	2;0;2	1;0;1
groupIV	5221500	5237500	2;3;	55;11;	11;0;11	0;0;0	2;0;2	3;0;3	3;0;3	3;0;3	0;0;0	0;0;0	0;0;0	0;0;0	0;0;0	0;0;0
groupIV	5864500	5876500	2;3;	65;1;	1;0;1	1;0;1	0;0;0	0;0;0	0;0;0	0;0;0	0;0;0	0;0;0	0;0;0	0;0;0	0;0;0	0;0;0
groupIV	6795500	6796500	1;2;	4;62;	4;0;4	0;0;0	0;0;0	0;0;0	0;0;0	0;0;0	4;0;4	0;0;0	0;0;0	0;0;0	0;0;0	0;0;0

groupIV	7142500	7144500	0;1;2;	1;9;56;	11;1;9	0;0;0	0;0;0	0;0;0	0;0;0	0;0;0	7;1;5	4;0;4	0;0;0	0;0;0	0;0;0	0;0;0
groupIV	7590000	7593000	1;2;	1;65;	1;0;1	0;0;0	0;0;0	0;0;0	0;0;0	0;0;0	0;0;0	0;0;0	1;0;1	0;0;0	0;0;0	0;0;0
groupIV	7668000	7677500	1;2;	16;50;	16;0;16	0;0;0	0;0;0	0;0;0	0;0;0	0;0;0	0;0;0	0;0;0	6;0;6	0;0;0	6;0;6	4;0;4
groupIV	7845500	7848500	1;2;	2;64;	2;0;2	0;0;0	0;0;0	0;0;0	0;0;0	0;0;0	0;0;0	0;0;0	1;0;1	1;0;1	0;0;0	0;0;0
groupIV	7855500	7872000	2;3;	46;20;	20;0;20	0;0;0	0;0;0	0;0;0	0;0;0	0;0;0	0;0;0	0;0;0	4;0;4	5;0;5	6;0;6	5;0;5
groupIV	7888000	7893000	1;2;	1;65;	1;0;1	1;0;1	0;0;0	0;0;0	0;0;0	0;0;0	0;0;0	0;0;0	0;0;0	0;0;0	0;0;0	0;0;0
groupIV	8114500	8117500	0;1;2;	2;1;63;	5;2;1	5;2;1	0;0;0	0;0;0	0;0;0	0;0;0	0;0;0	0;0;0	0;0;0	0;0;0	0;0;0	0;0;0
groupIV	8178000	8188000	2;3;	64;2;	2;0;2	0;0;0	0;0;0	0;0;0	0;0;0	0;0;0	0;0;0	0;0;0	0;0;0	0;0;0	1;0;1	1;0;1
groupIV	8353500	8358500	2;4;	65;1;	2;1;0	0;0;0	0;0;0	0;0;0	0;0;0	2;1;0	0;0;0	0;0;0	0;0;0	0;0;0	0;0;0	0;0;0
groupIV	8598000	8603000	1;2;	1;65;	1;0;1	1;0;1	0;0;0	0;0;0	0;0;0	0;0;0	0;0;0	0;0;0	0;0;0	0;0;0	0;0;0	0;0;0
groupIV	8697500	8707000	2;3;	65;1;	1;0;1	0;0;0	0;0;0	0;0;0	0;0;0	1;0;1	0;0;0	0;0;0	0;0;0	0;0;0	0;0;0	0;0;0
groupIV	9438500	9461000	2;3;	64;2;	2;0;2	0;0;0	0;0;0	2;0;2	0;0;0	0;0;0	0;0;0	0;0;0	0;0;0	0;0;0	0;0;0	0;0;0
groupIV	9617000	9619500	1;2;	1;65;	1;0;1	1;0;1	0;0;0	0;0;0	0;0;0	0;0;0	0;0;0	0;0;0	0;0;0	0;0;0	0;0;0	0;0;0
groupIV	9731000	9734500	1;2;	1;65;	1;0;1	1;0;1	0;0;0	0;0;0	0;0;0	0;0;0	0;0;0	0;0;0	0;0;0	0;0;0	0;0;0	0;0;0
groupIV	9840500	9843500	1;2;	3;63;	3;0;3	0;0;0	0;0;0	0;0;0	0;0;0	3;0;3	0;0;0	0;0;0	0;0;0	0;0;0	0;0;0	0;0;0
groupIV	9954500	9957000	0;1;2;	1;5;60;	7;1;5	1;0;1	4;1;2	0;0;0	0;0;0	2;0;2	0;0;0	0;0;0	0;0;0	0;0;0	0;0;0	0;0;0
groupIV	9957500	9961500	1;2;	1;65;	1;0;1	1;0;1	0;0;0	0;0;0	0;0;0	0;0;0	0;0;0	0;0;0	0;0;0	0;0;0	0;0;0	0;0;0
groupIV	9975500	9980000	0;1;2;	1;2;63;	4;1;2	0;0;0	0;0;0	0;0;0	4;1;2	0;0;0	0;0;0	0;0;0	0;0;0	0;0;0	0;0;0	0;0;0
groupIV	10034000	10037500	0;1;2;	2;2;62;	6;2;2	0;0;0	0;0;0	0;0;0	0;0;0	0;0;0	0;0;0	0;0;0	0;0;0	2;0;2	2;1;0	2;1;0
groupIV	10093000	10095500	0;1;2;	2;5;59;	9;2;5	2;0;2	0;0;0	0;0;0	0;0;0	1;0;1	0;0;0	0;0;0	0;0;0	0;0;0	6;2;2	0;0;0
groupIV	10110500	10138500	1;2;	3;63;	3;0;3	1;0;1	0;0;0	0;0;0	0;0;0	0;0;0	2;0;2	0;0;0	0;0;0	0;0;0	0;0;0	0;0;0
groupIV	10167500	10172500	2;3;	62;4;	4;0;4	0;0;0	4;0;4	0;0;0	0;0;0	0;0;0	0;0;0	0;0;0	0;0;0	0;0;0	0;0;0	0;0;0
groupIV	10392000	10393500	0;1;2;	1;2;63;	4;1;2	0;0;0	0;0;0	0;0;0	4;1;2	0;0;0	0;0;0	0;0;0	0;0;0	0;0;0	0;0;0	0;0;0
groupIV	10428500	10431000	1;2;	2;64;	2;0;2	0;0;0	0;0;0	1;0;1	0;0;0	1;0;1	0;0;0	0;0;0	0;0;0	0;0;0	0;0;0	0;0;0
groupIV	10895000	10896500	0;1;2;	2;7;57;	11;2;7	2;1;0	3;1;1	2;0;2	1;0;1	2;0;2	0;0;0	0;0;0	0;0;0	0;0;0	0;0;0	1;0;1
groupIV	11259000	11269500	2;3;	41;25;	25;0;25	0;0;0	0;0;0	0;0;0	0;0;0	0;0;0	0;0;0	1;0;1	6;0;6	6;0;6	6;0;6	6;0;6
groupIV	11473500	11477000	2;3;	63;3;	3;0;3	2;0;2	0;0;0	0;0;0	0;0;0	0;0;0	0;0;0	1;0;1	0;0;0	0;0;0	0;0;0	0;0;0
groupIV	12436500	12442500	2;3;	64;2;	2;0;2	1;0;1	0;0;0	0;0;0	0;0;0	0;0;0	0;0;0	0;0;0	1;0;1	0;0;0	0;0;0	0;0;0
groupIV	12638000	12643000	0;1;2;	1;3;62;	5;1;3	0;0;0	5;1;3	0;0;0	0;0;0	0;0;0	0;0;0	0;0;0	0;0;0	0;0;0	0;0;0	0;0;0
groupIV	12743500	12746500	1;2;	2;64;	2;0;2	0;0;0	0;0;0	2;0;2	0;0;0	0;0;0	0;0;0	0;0;0	0;0;0	0;0;0	0;0;0	0;0;0
groupIV	13212500	13221000	2;3;4;	60;5;1;	7;1;5	0;0;0	0;0;0	0;0;0	0;0;0	0;0;0	7;1;5	0;0;0	0;0;0	0;0;0	0;0;0	0;0;0
groupIV	13707000	13709000	0;1;2;	1;8;57;	10;1;8	0;0;0	0;0;0	0;0;0	0;0;0	0;0;0	0;0;0	0;0;0	2;0;2	1;0;1	4;1;2	3;0;3
groupIV	13712500	13714000	0;1;2;	2;2;62;	6;2;2	0;0;0	0;0;0	0;0;0	0;0;0	0;0;0	0;0;0	0;0;0	0;0;0	0;0;0	6;2;2	0;0;0
groupIV	14171500	14173500	1;2;	2;64;	2;0;2	2;0;2	0;0;0	0;0;0	0;0;0	0;0;0	0;0;0	0;0;0	0;0;0	0;0;0	0;0;0	0;0;0

groupIV	14365500	14366500	0;1;2;	6;13;47;	25;6;13	0;0;0	0;0;0	0;0;0	0;0;0	0;0;0	0;0;0	0;0;0	6;1;4	3;0;3	11;5;1	5;0;5
groupIV	14497000	14499000	0;1;2;	1;6;59;	8;1;6	0;0;0	0;0;0	0;0;0	0;0;0	0;0;0	0;0;0	0;0;0	0;0;0	0;0;0	7;1;5	1;0;1
groupIV	14753000	14755000	0;1;2;	9;12;45;	30;9;12	2;0;2	0;0;0	0;0;0	0;0;0	0;0;0	0;0;0	0;0;0	6;1;4	2;0;2	12;6;0	8;2;4
groupIV	14760500	14762500	0;1;2;	1;16;49;	18;1;16	0;0;0	0;0;0	0;0;0	0;0;0	0;0;0	0;0;0	0;0;0	6;1;4	2;0;2	5;0;5	5;0;5
groupIV	14866000	14869000	1;2;	3;63;	3;0;3	0;0;0	0;0;0	0;0;0	0;0;0	0;0;0	0;0;0	0;0;0	3;0;3	0;0;0	0;0;0	0;0;0
groupIV	14987500	15010000	2;3;	65;1;	1;0;1	0;0;0	0;0;0	0;0;0	0;0;0	0;0;0	0;0;0	0;0;0	1;0;1	0;0;0	0;0;0	0;0;0
groupIV	15209000	15210500	0;1;2;	1;4;61;	6;1;4	0;0;0	0;0;0	0;0;0	0;0;0	0;0;0	5;1;3	1;0;1	0;0;0	0;0;0	0;0;0	0;0;0
groupIV	15729500	15742500	2;3;	54;12;	12;0;12	0;0;0	0;0;0	0;0;0	0;0;0	0;0;0	0;0;0	0;0;0	0;0;0	0;0;0	6;0;6	6;0;6
groupIV	15776000	15778500	0;1;2;	11;13;42;	35;11;1	0;0;0	0;0;0	0;0;0	0;0;0	0;0;0	0;0;0	0;0;0	6;0;6	6;0;6	12;6;0	11;5;1
groupIV	16092000	16102000	2;3;	61;5;	5;0;5	0;0;0	5;0;5	0;0;0	0;0;0	0;0;0	0;0;0	0;0;0	0;0;0	0;0;0	0;0;0	0;0;0
groupIV	16692000	16693500	0;1;2;	2;3;61;	7;2;3	0;0;0	0;0;0	0;0;0	0;0;0	0;0;0	0;0;0	0;0;0	0;0;0	0;0;0	0;0;0	7;2;3
groupIV	16755000	16758500	2;3;	46;20;	20;0;20	0;0;0	0;0;0	0;0;0	0;0;0	0;0;0	0;0;0	0;0;0	5;0;5	3;0;3	6;0;6	6;0;6
groupIV	16979000	16981500	1;2;	6;60;	6;0;6	0;0;0	0;0;0	0;0;0	0;0;0	0;0;0	0;0;0	0;0;0	2;0;2	4;0;4	0;0;0	0;0;0
groupIV	17357000	17360000	0;1;2;	9;3;54;	21;9;3	0;0;0	0;0;0	0;0;0	0;0;0	0;0;0	0;0;0	0;0;0	0;0;0	0;0;0	10;4;2	11;5;1
groupIV	17378000	17381000	0;1;2;	3;6;57;	12;3;6	0;0;0	3;0;3	3;1;1	3;1;1	3;1;1	0;0;0	0;0;0	0;0;0	0;0;0	0;0;0	0;0;0
groupIV	17434000	17437500	1;2;	5;61;	5;0;5	0;0;0	0;0;0	0;0;0	0;0;0	0;0;0	0;0;0	0;0;0	2;0;2	1;0;1	2;0;2	0;0;0
groupIV	17444000	17446000	0;1;2;	2;7;57;	11;2;7	0;0;0	0;0;0	0;0;0	0;0;0	0;0;0	0;0;0	0;0;0	2;0;2	2;0;2	0;0;0	7;2;3
groupIV	17447000	17449000	0;1;2;	4;6;56;	14;4;6	0;0;0	0;0;0	0;0;0	0;0;0	0;0;0	0;0;0	0;0;0	0;0;0	0;0;0	10;4;2	4;0;4
groupIV	17525500	17529000	1;2;	1;65;	1;0;1	0;0;0	0;0;0	0;0;0	0;0;0	0;0;0	0;0;0	0;0;0	0;0;0	0;0;0	0;0;0	1;0;1
groupIV	17539500	17545500	1;2;	2;64;	2;0;2	0;0;0	0;0;0	0;0;0	0;0;0	0;0;0	0;0;0	0;0;0	0;0;0	0;0;0	2;0;2	0;0;0
groupIV	17557000	17559500	0;1;2;	5;5;56;	15;5;5	0;0;0	0;0;0	0;0;0	0;0;0	0;0;0	0;0;0	0;0;0	0;0;0	0;0;0	10;4;2	5;1;3
groupIV	18105500	18107000	0;1;2;	1;5;60;	7;1;5	0;0;0	0;0;0	0;0;0	0;0;0	0;0;0	0;0;0	0;0;0	1;0;1	6;1;4	0;0;0	0;0;0
groupIV	19535000	19543500	1;2;	1;65;	1;0;1	0;0;0	0;0;0	0;0;0	0;0;0	1;0;1	0;0;0	0;0;0	0;0;0	0;0;0	0;0;0	0;0;0
groupIV	19591500	19592500	0;1;2;	1;1;64;	3;1;1	3;1;1	0;0;0	0;0;0	0;0;0	0;0;0	0;0;0	0;0;0	0;0;0	0;0;0	0;0;0	0;0;0
groupIV	19836000	19848500	2;3;	56;10;	10;0;10	0;0;0	0;0;0	0;0;0	0;0;0	0;0;0	0;0;0	0;0;0	6;0;6	3;0;3	1;0;1	0;0;0
groupIV	19936500	19940500	0;1;2;	1;1;64;	3;1;1	0;0;0	0;0;0	0;0;0	0;0;0	0;0;0	0;0;0	0;0;0	0;0;0	0;0;0	3;1;1	0;0;0
groupIV	19954500	19963500	0;1;2;	1;7;58;	9;1;7	0;0;0	0;0;0	0;0;0	0;0;0	0;0;0	0;0;0	0;0;0	6;1;4	3;0;3	0;0;0	0;0;0
groupIV	20385000	20387000	1;2;	10;56;	10;0;10	0;0;0	0;0;0	0;0;0	0;0;0	0;0;0	0;0;0	0;0;0	0;0;0	0;0;0	6;0;6	4;0;4
groupIV	20408500	20410500	1;2;	2;64;	2;0;2	0;0;0	0;0;0	0;0;0	0;0;0	0;0;0	0;0;0	0;0;0	1;0;1	1;0;1	0;0;0	0;0;0
groupIV	20496500	20498000	1;2;	22;44;	22;0;22	0;0;0	0;0;0	0;0;0	0;0;0	0;0;0	0;0;0	1;0;1	6;0;6	3;0;3	6;0;6	6;0;6
groupIV	20540500	20544500	1;2;	3;63;	3;0;3	0;0;0	0;0;0	0;0;0	0;0;0	1;0;1	0;0;0	0;0;0	0;0;0	0;0;0	1;0;1	1;0;1
groupIV	20552500	20557000	1;2;	2;64;	2;0;2	0;0;0	0;0;0	0;0;0	2;0;2	0;0;0	0;0;0	0;0;0	0;0;0	0;0;0	0;0;0	0;0;0
groupIV	20581500	20585000	1;2;	1;65;	1;0;1	1;0;1	0;0;0	0;0;0	0;0;0	0;0;0	0;0;0	0;0;0	0;0;0	0;0;0	0;0;0	0;0;0
groupIV	20697000	20698000	0;1;2;	1;8;57;	10;1;8	0;0;0	0;0;0	0;0;0	0;0;0	0;0;0	0;0;0	0;0;0	1;0;1	2;0;2	5;0;5	2;1;0

groupIV	21017000	21020500	2;4;	42;24;	48;24;0	0;0;0	0;0;0	0;0;0	0;0;0	0;0;0	0;0;0	0;0;0	12;6;0	12;6;0	12;6;0	12;6;0
groupIV	21034500	21047000	2;3;	44;22;	22;0;22	0;0;0	0;0;0	0;0;0	0;0;0	0;0;0	0;0;0	0;0;0	5;0;5	5;0;5	6;0;6	6;0;6
groupIV	21047500	21192000	2;3;	42;24;	24;0;24	0;0;0	0;0;0	0;0;0	0;0;0	0;0;0	0;0;0	0;0;0	6;0;6	6;0;6	6;0;6	6;0;6
groupIV	21424500	21426000	0;2;	1;65;	2;1;0	0;0;0	0;0;0	0;0;0	0;0;0	0;0;0	0;0;0	2;1;0	0;0;0	0;0;0	0;0;0	0;0;0
groupIV	21459500	21463000	1;2;	4;62;	4;0;4	0;0;0	4;0;4	0;0;0	0;0;0	0;0;0	0;0;0	0;0;0	0;0;0	0;0;0	0;0;0	0;0;0
groupIV	21494500	21497000	1;2;	1;65;	1;0;1	0;0;0	0;0;0	0;0;0	1;0;1	0;0;0	0;0;0	0;0;0	0;0;0	0;0;0	0;0;0	0;0;0
groupIV	22014500	22020000	1;2;	13;53;	13;0;13	0;0;0	0;0;0	0;0;0	0;0;0	0;0;0	0;0;0	0;0;0	0;0;0	1;0;1	6;0;6	6;0;6
groupIV	22045500	22053500	1;2;	20;46;	20;0;20	0;0;0	0;0;0	0;0;0	0;0;0	0;0;0	0;0;0	0;0;0	6;0;6	2;0;2	6;0;6	6;0;6
groupIV	22045500	22047500	1;2;	6;60;	6;0;6	0;0;0	0;0;0	0;0;0	0;0;0	0;0;0	0;0;0	0;0;0	4;0;4	0;0;0	2;0;2	0;0;0
groupIV	22160500	22165000	1;2;	23;43;	23;0;23	0;0;0	0;0;0	0;0;0	0;0;0	0;0;0	0;0;0	0;0;0	6;0;6	5;0;5	6;0;6	6;0;6
groupIV	22230500	22234500	1;2;	15;51;	15;0;15	0;0;0	0;0;0	1;0;1	0;0;0	3;0;3	0;0;0	0;0;0	6;0;6	5;0;5	0;0;0	0;0;0
groupIV	22265000	22267500	0;1;2;	20;4;42;	44;20;4	0;0;0	0;0;0	0;0;0	0;0;0	0;0;0	0;0;0	0;0;0	12;6;0	8;2;4	12;6;0	12;6;0
groupIV	22703000	22705500	0;1;2;	2;11;53;	15;2;11	5;1;3	0;0;0	5;1;3	2;0;2	3;0;3	0;0;0	0;0;0	0;0;0	0;0;0	0;0;0	0;0;0
groupIV	22930500	22934500	1;2;	1;65;	1;0;1	0;0;0	0;0;0	0;0;0	0;0;0	0;0;0	0;0;0	0;0;0	1;0;1	0;0;0	0;0;0	0;0;0
groupIV	23022000	23031500	2;3;	65;1;	1;0;1	0;0;0	0;0;0	1;0;1	0;0;0	0;0;0	0;0;0	0;0;0	0;0;0	0;0;0	0;0;0	0;0;0
groupIV	23651000	23656500	1;2;	10;56;	10;0;10	0;0;0	0;0;0	0;0;0	0;0;0	0;0;0	0;0;0	0;0;0	0;0;0	0;0;0	6;0;6	4;0;4
groupIV	23767000	23768500	0;1;2;	10;2;54;	22;10;2	0;0;0	0;0;0	0;0;0	0;0;0	0;0;0	0;0;0	0;0;0	0;0;0	0;0;0	11;5;1	11;5;1
groupIV	23816500	23833500	2;3;	65;1;	1;0;1	0;0;0	0;0;0	0;0;0	0;0;0	0;0;0	0;0;0	0;0;0	0;0;0	0;0;0	1;0;1	0;0;0
groupIV	23875000	23900000	2;3;	46;20;	20;0;20	0;0;0	0;0;0	0;0;0	0;0;0	0;0;0	0;0;0	0;0;0	4;0;4	4;0;4	6;0;6	6;0;6
groupIV	23949500	23956500	1;2;	15;51;	15;0;15	1;0;1	0;0;0	0;0;0	0;0;0	0;0;0	0;0;0	1;0;1	0;0;0	1;0;1	6;0;6	6;0;6
groupIV	24150500	24152500	0;1;2;	10;14;42;	34;10;1[illegible]	0;0;0	0;0;0	0;0;0	0;0;0	0;0;0	0;0;0	0;0;0	11;5;1	10;4;2	7;1;5	6;0;6
groupIV	24188000	24191500	1;2;	19;47;	19;0;19	0;0;0	0;0;0	0;0;0	0;0;0	0;0;0	0;0;0	0;0;0	6;0;6	5;0;5	5;0;5	3;0;3
groupIV	24248500	24257000	2;3;4;	42;5;19;	43;19;5	0;0;0	0;0;0	0;0;0	0;0;0	0;0;0	0;0;0	0;0;0	12;6;0	11;5;1	10;4;2	10;4;2
groupIV	24316000	24319000	0;1;2;	1;16;49;	18;1;16	0;0;0	0;0;0	0;0;0	0;0;0	0;0;0	0;0;0	0;0;0	4;0;4	1;0;1	6;0;6	7;1;5
groupIV	24539500	24547000	2;3;	64;2;	2;0;2	0;0;0	0;0;0	0;0;0	0;0;0	0;0;0	0;0;0	0;0;0	0;0;0	2;0;2	0;0;0	0;0;0
groupIV	24614000	24627000	2;3;4;	63;1;2;	5;2;1	0;0;0	1;0;1	0;0;0	2;1;0	2;1;0	0;0;0	0;0;0	0;0;0	0;0;0	0;0;0	0;0;0
groupIV	24730500	24734500	0;1;2;	8;20;38;	36;8;20	3;0;3	5;2;1	5;0;5	5;0;5	3;0;3	11;5;1	4;1;2	0;0;0	0;0;0	0;0;0	0;0;0
groupIV	24839000	24841500	1;2;	24;42;	24;0;24	0;0;0	0;0;0	0;0;0	0;0;0	0;0;0	0;0;0	0;0;0	6;0;6	6;0;6	6;0;6	6;0;6
groupIV	24859500	24860500	0;1;2;	12;4;50;	28;12;4	0;0;0	0;0;0	0;0;0	0;0;0	0;0;0	0;0;0	0;0;0	5;1;3	0;0;0	12;6;0	11;5;1
groupIV	24893500	24910500	1;2;	14;52;	14;0;14	0;0;0	0;0;0	0;0;0	0;0;0	0;0;0	0;0;0	0;0;0	3;0;3	0;0;0	6;0;6	5;0;5
groupIV	24915000	24934000	1;2;	6;60;	6;0;6	0;0;0	0;0;0	0;0;0	0;0;0	0;0;0	0;0;0	0;0;0	0;0;0	0;0;0	5;0;5	1;0;1
groupIV	24989500	24991500	2;3;	60;6;	6;0;6	0;0;0	0;0;0	0;0;0	0;0;0	0;0;0	0;0;0	0;0;0	3;0;3	2;0;2	1;0;1	0;0;0
groupIV	24997500	24999500	1;2;	10;56;	10;0;10	0;0;0	0;0;0	0;0;0	0;0;0	0;0;0	0;0;0	0;0;0	1;0;1	0;0;0	4;0;4	5;0;5
groupIV	25130000	25132500	1;2;	9;57;	9;0;9	0;0;0	0;0;0	0;0;0	0;0;0	0;0;0	0;0;0	0;0;0	3;0;3	1;0;1	5;0;5	0;0;0

groupIV	25276500	25278000	1;2;	1;65;	1;0;1	0;0;0	0;0;0	0;0;0	0;0;0	0;0;0	0;0;0	1;0;1	0;0;0	0;0;0	0;0;0	0;0;0
groupIV	25425000	25428000	2;3;	48;18;	18;0;18	0;0;0	0;0;0	0;0;0	0;0;0	0;0;0	0;0;0	0;0;0	6;0;6	1;0;1	6;0;6	5;0;5
groupIV	25512000	25519000	2;3;	65;1;	1;0;1	0;0;0	0;0;0	0;0;0	0;0;0	0;0;0	0;0;0	0;0;0	0;0;0	1;0;1	0;0;0	0;0;0
groupIV	25687000	25730500	1;2;	6;60;	6;0;6	2;0;2	0;0;0	0;0;0	0;0;0	2;0;2	0;0;0	1;0;1	0;0;0	1;0;1	0;0;0	0;0;0
groupIV	25743000	25751000	0;1;2;	2;10;54;	14;2;10	0;0;0	1;0;1	1;0;1	6;2;2	3;0;3	0;0;0	0;0;0	0;0;0	0;0;0	2;0;2	1;0;1
groupIV	25820500	25829000	2;3;	65;1;	1;0;1	0;0;0	0;0;0	0;0;0	0;0;0	0;0;0	0;0;0	0;0;0	0;0;0	1;0;1	0;0;0	0;0;0
groupIV	25965000	25967500	0;1;2;	1;4;61;	6;1;4	0;0;0	0;0;0	4;1;2	0;0;0	2;0;2	0;0;0	0;0;0	0;0;0	0;0;0	0;0;0	0;0;0
groupIV	26001000	26009500	2;3;	63;3;	3;0;3	0;0;0	0;0;0	0;0;0	0;0;0	0;0;0	0;0;0	0;0;0	0;0;0	0;0;0	2;0;2	1;0;1
groupIV	26052000	26067500	2;3;	65;1;	1;0;1	0;0;0	0;0;0	0;0;0	0;0;0	0;0;0	1;0;1	0;0;0	0;0;0	0;0;0	0;0;0	0;0;0
groupIV	26367500	26377000	2;3;	65;1;	1;0;1	0;0;0	0;0;0	0;0;0	0;0;0	0;0;0	0;0;0	0;0;0	1;0;1	0;0;0	0;0;0	0;0;0
groupIV	26419500	26441500	2;3;	47;19;	19;0;19	0;0;0	0;0;0	0;0;0	0;0;0	0;0;0	0;0;0	0;0;0	5;0;5	5;0;5	4;0;4	5;0;5
groupIV	26503500	26530500	0;1;2;	3;2;61;	8;3;2	0;0;0	0;0;0	0;0;0	0;0;0	0;0;0	0;0;0	0;0;0	0;0;0	0;0;0	7;3;1	1;0;1
groupIV	26619500	26621500	0;1;2;	1;2;63;	4;1;2	0;0;0	2;1;0	0;0;0	1;0;1	1;0;1	0;0;0	0;0;0	0;0;0	0;0;0	0;0;0	0;0;0
groupIV	26676000	26678000	1;2;	1;65;	1;0;1	1;0;1	0;0;0	0;0;0	0;0;0	0;0;0	0;0;0	0;0;0	0;0;0	0;0;0	0;0;0	0;0;0
groupIV	26696000	26699500	1;2;	12;54;	12;0;12	3;0;3	0;0;0	0;0;0	1;0;1	1;0;1	0;0;0	0;0;0	0;0;0	0;0;0	2;0;2	5;0;5
groupIV	26808500	26810500	0;1;2;	1;3;62;	5;1;3	0;0;0	0;0;0	0;0;0	0;0;0	0;0;0	4;1;2	1;0;1	0;0;0	0;0;0	0;0;0	0;0;0
groupIV	26852500	26857000	2;3;4;	61;4;1;	6;1;4	0;0;0	3;1;1	2;0;2	1;0;1	0;0;0	0;0;0	0;0;0	0;0;0	0;0;0	0;0;0	0;0;0
groupIV	26894500	26899000	0;1;2;	2;5;59;	9;2;5	1;0;1	1;0;1	3;1;1	0;0;0	4;1;2	0;0;0	0;0;0	0;0;0	0;0;0	0;0;0	0;0;0
groupIV	26998000	26999500	0;2;	1;65;	2;1;0	2;1;0	0;0;0	0;0;0	0;0;0	0;0;0	0;0;0	0;0;0	0;0;0	0;0;0	0;0;0	0;0;0
groupIV	27125000	27126500	1;2;	1;65;	1;0;1	0;0;0	0;0;0	0;0;0	0;0;0	0;0;0	0;0;0	0;0;0	1;0;1	0;0;0	0;0;0	0;0;0
groupIV	27133500	27140000	1;2;	4;62;	4;0;4	0;0;0	0;0;0	0;0;0	0;0;0	0;0;0	0;0;0	0;0;0	4;0;4	0;0;0	0;0;0	0;0;0
groupIV	27167000	27178000	1;2;	15;51;	15;0;15	0;0;0	1;0;1	0;0;0	0;0;0	0;0;0	0;0;0	0;0;0	3;0;3	6;0;6	1;0;1	4;0;4
groupIV	27389500	27393000	1;2;	2;64;	2;0;2	0;0;0	0;0;0	0;0;0	0;0;0	0;0;0	0;0;0	0;0;0	2;0;2	0;0;0	0;0;0	0;0;0
groupIV	27400500	27404000	1;2;	8;58;	8;0;8	0;0;0	0;0;0	0;0;0	0;0;0	0;0;0	0;0;0	0;0;0	0;0;0	1;0;1	3;0;3	4;0;4
groupIV	27851000	27852500	0;1;2;	1;4;61;	6;1;4	0;0;0	0;0;0	0;0;0	0;0;0	0;0;0	0;0;0	0;0;0	1;0;1	4;1;2	0;0;0	1;0;1
groupIV	28013000	28017000	1;2;	1;65;	1;0;1	1;0;1	0;0;0	0;0;0	0;0;0	0;0;0	0;0;0	0;0;0	0;0;0	0;0;0	0;0;0	0;0;0
groupIV	28228000	28232000	1;2;	3;63;	3;0;3	2;0;2	0;0;0	0;0;0	0;0;0	0;0;0	0;0;0	1;0;1	0;0;0	0;0;0	0;0;0	0;0;0
groupIV	28663500	28665500	1;2;	1;65;	1;0;1	0;0;0	0;0;0	0;0;0	0;0;0	0;0;0	0;0;0	1;0;1	0;0;0	0;0;0	0;0;0	0;0;0
groupIV	28927000	28930000	1;2;	1;65;	1;0;1	0;0;0	0;0;0	0;0;0	1;0;1	0;0;0	0;0;0	0;0;0	0;0;0	0;0;0	0;0;0	0;0;0
groupIV	29354000	29356500	1;2;	1;65;	1;0;1	0;0;0	1;0;1	0;0;0	0;0;0	0;0;0	0;0;0	0;0;0	0;0;0	0;0;0	0;0;0	0;0;0
groupIV	30482000	30484500	1;2;	4;62;	4;0;4	0;0;0	0;0;0	0;0;0	0;0;0	0;0;0	0;0;0	0;0;0	0;0;0	1;0;1	2;0;2	1;0;1
groupIV	31745000	31749000	1;2;	4;62;	4;0;4	0;0;0	0;0;0	0;0;0	0;0;0	0;0;0	0;0;0	0;0;0	0;0;0	0;0;0	2;0;2	2;0;2
groupIV	31992000	31995500	1;2;	1;65;	1;0;1	0;0;0	0;0;0	0;0;0	0;0;0	0;0;0	0;0;0	0;0;0	0;0;0	0;0;0	1;0;1	0;0;0
groupIV	32056000	32058500	1;2;	3;63;	3;0;3	0;0;0	0;0;0	0;0;0	0;0;0	0;0;0	0;0;0	0;0;0	0;0;0	0;0;0	0;0;0	3;0;3

groupIV	32063000	32066000	1;2;	4;62;	4;0;4	0;0;0	0;0;0	0;0;0	0;0;0	0;0;0	0;0;0	0;0;0	2;0;2	2;0;2	0;0;0	0;0;0
groupIV	32166500	32168500	1;2;	1;65;	1;0;1	1;0;1	0;0;0	0;0;0	0;0;0	0;0;0	0;0;0	0;0;0	0;0;0	0;0;0	0;0;0	0;0;0
groupIV	32204000	32208000	1;2;	1;65;	1;0;1	0;0;0	0;0;0	0;0;0	0;0;0	0;0;0	0;0;0	0;0;0	0;0;0	0;0;0	1;0;1	0;0;0
groupIV	32440500	32457500	2;3;	44;22;	22;0;22	0;0;0	0;0;0	0;0;0	0;0;0	0;0;0	0;0;0	0;0;0	6;0;6	6;0;6	5;0;5	5;0;5
groupIV	32566000	32575000	1;2;	2;64;	2;0;2	0;0;0	0;0;0	0;0;0	0;0;0	0;0;0	0;0;0	0;0;0	0;0;0	0;0;0	2;0;2	0;0;0
groupV	23000	28000	2;3;	64;2;	2;0;2	0;0;0	0;0;0	0;0;0	0;0;0	2;0;2	0;0;0	0;0;0	0;0;0	0;0;0	0;0;0	0;0;0
groupV	296500	300000	0;1;2;	1;1;64;	3;1;1	0;0;0	0;0;0	0;0;0	0;0;0	0;0;0	0;0;0	0;0;0	0;0;0	0;0;0	3;1;1	0;0;0
groupV	870500	872000	0;1;2;	4;8;54;	16;4;8	0;0;0	0;0;0	0;0;0	0;0;0	0;0;0	0;0;0	0;0;0	0;0;0	2;0;2	9;4;1	5;0;5
groupV	881500	884500	1;2;	4;62;	4;0;4	0;0;0	0;0;0	2;0;2	0;0;0	2;0;2	0;0;0	0;0;0	0;0;0	0;0;0	0;0;0	0;0;0
groupV	1396500	1405000	2;3;	65;1;	1;0;1	1;0;1	0;0;0	0;0;0	0;0;0	0;0;0	0;0;0	0;0;0	0;0;0	0;0;0	0;0;0	0;0;0
groupV	1524000	1531500	2;3;	65;1;	1;0;1	0;0;0	0;0;0	0;0;0	0;0;0	0;0;0	0;0;0	0;0;0	0;0;0	1;0;1	0;0;0	0;0;0
groupV	2042500	2043500	1;2;	6;60;	6;0;6	1;0;1	0;0;0	0;0;0	0;0;0	0;0;0	0;0;0	0;0;0	1;0;1	0;0;0	3;0;3	1;0;1
groupV	2178500	2187500	1;2;	12;54;	12;0;12	0;0;0	0;0;0	0;0;0	0;0;0	0;0;0	0;0;0	0;0;0	0;0;0	0;0;0	6;0;6	6;0;6
groupV	2708500	2712000	1;2;	1;65;	1;0;1	1;0;1	0;0;0	0;0;0	0;0;0	0;0;0	0;0;0	0;0;0	0;0;0	0;0;0	0;0;0	0;0;0
groupV	2799500	2807500	2;3;	57;9;	9;0;9	0;0;0	0;0;0	3;0;3	0;0;0	3;0;3	2;0;2	1;0;1	0;0;0	0;0;0	0;0;0	0;0;0
groupV	3053500	3062500	1;2;	1;65;	1;0;1	0;0;0	0;0;0	0;0;0	1;0;1	0;0;0	0;0;0	0;0;0	0;0;0	0;0;0	0;0;0	0;0;0
groupV	3322500	3331000	2;3;	65;1;	1;0;1	1;0;1	0;0;0	0;0;0	0;0;0	0;0;0	0;0;0	0;0;0	0;0;0	0;0;0	0;0;0	0;0;0
groupV	3376500	3381000	1;2;	1;65;	1;0;1	0;0;0	0;0;0	0;0;0	0;0;0	0;0;0	0;0;0	0;0;0	1;0;1	0;0;0	0;0;0	0;0;0
groupV	3800500	3803500	1;2;	1;65;	1;0;1	0;0;0	0;0;0	0;0;0	0;0;0	0;0;0	0;0;0	1;0;1	0;0;0	0;0;0	0;0;0	0;0;0
groupV	4227000	4230000	1;2;	1;65;	1;0;1	0;0;0	0;0;0	0;0;0	0;0;0	1;0;1	0;0;0	0;0;0	0;0;0	0;0;0	0;0;0	0;0;0
groupV	4246500	4248500	0;1;2;	1;1;64;	3;1;1	0;0;0	0;0;0	0;0;0	0;0;0	0;0;0	0;0;0	3;1;1	0;0;0	0;0;0	0;0;0	0;0;0
groupV	4318000	4323500	1;2;	2;64;	2;0;2	0;0;0	0;0;0	0;0;0	0;0;0	0;0;0	0;0;0	0;0;0	0;0;0	1;0;1	1;0;1	0;0;0
groupV	4633000	4638000	2;3;	65;1;	1;0;1	0;0;0	0;0;0	0;0;0	0;0;0	0;0;0	0;0;0	0;0;0	0;0;0	0;0;0	1;0;1	0;0;0
groupV	4726000	4728000	1;2;	2;64;	2;0;2	0;0;0	0;0;0	0;0;0	0;0;0	2;0;2	0;0;0	0;0;0	0;0;0	0;0;0	0;0;0	0;0;0
groupV	4774500	4782000	1;2;	1;65;	1;0;1	0;0;0	0;0;0	0;0;0	0;0;0	1;0;1	0;0;0	0;0;0	0;0;0	0;0;0	0;0;0	0;0;0
groupV	4816000	4822000	2;4;	65;1;	2;1;0	0;0;0	2;1;0	0;0;0	0;0;0	0;0;0	0;0;0	0;0;0	0;0;0	0;0;0	0;0;0	0;0;0
groupV	5428500	5432000	0;1;2;	5;12;49;	22;5;12	0;0;0	0;0;0	0;0;0	0;0;0	0;0;0	0;0;0	0;0;0	1;0;1	4;0;4	7;1;5	10;4;2
groupV	5563500	5574000	1;2;	2;64;	2;0;2	0;0;0	0;0;0	0;0;0	0;0;0	0;0;0	0;0;0	2;0;2	0;0;0	0;0;0	0;0;0	0;0;0
groupV	6223500	6226000	0;1;2;	1;1;64;	3;1;1	0;0;0	0;0;0	3;1;1	0;0;0	0;0;0	0;0;0	0;0;0	0;0;0	0;0;0	0;0;0	0;0;0
groupV	6281000	6284500	2;3;	65;1;	1;0;1	1;0;1	0;0;0	0;0;0	0;0;0	0;0;0	0;0;0	0;0;0	0;0;0	0;0;0	0;0;0	0;0;0
groupV	6386500	6407500	2;3;	63;3;	3;0;3	0;0;0	0;0;0	2;0;2	0;0;0	0;0;0	0;0;0	1;0;1	0;0;0	0;0;0	0;0;0	0;0;0
groupV	6550500	6557500	1;2;	3;63;	3;0;3	0;0;0	0;0;0	0;0;0	0;0;0	0;0;0	0;0;0	0;0;0	1;0;1	2;0;2	0;0;0	0;0;0
groupV	6629500	6647000	2;3;	65;1;	1;0;1	0;0;0	0;0;0	0;0;0	1;0;1	0;0;0	0;0;0	0;0;0	0;0;0	0;0;0	0;0;0	0;0;0
groupV	6731500	6736000	0;1;2;	6;3;57;	15;6;3	0;0;0	0;0;0	0;0;0	0;0;0	0;0;0	0;0;0	0;0;0	0;0;0	0;0;0	12;6;0	3;0;3

groupV	6797000	6804500	0;1;2;	1;2;63;	4;1;2	0;0;0	0;0;0	3;1;1	0;0;0	1;0;1	0;0;0	0;0;0	0;0;0	0;0;0	0;0;0	0;0;0
groupV	7119000	7120000	1;2;	7;59;	7;0;7	0;0;0	0;0;0	2;0;2	4;0;4	1;0;1	0;0;0	0;0;0	0;0;0	0;0;0	0;0;0	0;0;0
groupV	7156000	7159500	1;2;	1;65;	1;0;1	0;0;0	0;0;0	0;0;0	0;0;0	0;0;0	0;0;0	1;0;1	0;0;0	0;0;0	0;0;0	0;0;0
groupV	7313500	7315000	0;1;2;	2;9;55;	13;2;9	0;0;0	7;2;3	0;0;0	3;0;3	2;0;2	0;0;0	0;0;0	0;0;0	1;0;1	0;0;0	0;0;0
groupV	7490000	7493000	1;2;	5;61;	5;0;5	0;0;0	0;0;0	0;0;0	0;0;0	0;0;0	0;0;0	0;0;0	0;0;0	0;0;0	4;0;4	1;0;1
groupV	7538500	7540000	1;2;	1;65;	1;0;1	0;0;0	0;0;0	0;0;0	0;0;0	0;0;0	0;0;0	0;0;0	1;0;1	0;0;0	0;0;0	0;0;0
groupV	7708000	7709500	0;1;2;	2;11;53;	15;2;11	0;0;0	0;0;0	0;0;0	0;0;0	0;0;0	0;0;0	0;0;0	1;0;1	6;1;4	4;0;4	4;1;2
groupV	7719500	7722000	1;2;	2;64;	2;0;2	0;0;0	0;0;0	0;0;0	0;0;0	1;0;1	0;0;0	1;0;1	0;0;0	0;0;0	0;0;0	0;0;0
groupV	7808000	7813500	1;2;	2;64;	2;0;2	0;0;0	0;0;0	0;0;0	0;0;0	0;0;0	0;0;0	0;0;0	2;0;2	0;0;0	0;0;0	0;0;0
groupV	8119500	8126000	2;3;	65;1;	1;0;1	0;0;0	0;0;0	0;0;0	0;0;0	1;0;1	0;0;0	0;0;0	0;0;0	0;0;0	0;0;0	0;0;0
groupV	8335000	8344500	2;3;	59;7;	7;0;7	0;0;0	0;0;0	2;0;2	4;0;4	1;0;1	0;0;0	0;0;0	0;0;0	0;0;0	0;0;0	0;0;0
groupV	8732000	8734500	1;2;	1;65;	1;0;1	0;0;0	0;0;0	0;0;0	0;0;0	0;0;0	0;0;0	0;0;0	0;0;0	0;0;0	0;0;0	1;0;1
groupV	9033000	9039500	2;3;	65;1;	1;0;1	1;0;1	0;0;0	0;0;0	0;0;0	0;0;0	0;0;0	0;0;0	0;0;0	0;0;0	0;0;0	0;0;0
groupV	9156500	9170500	2;3;	65;1;	1;0;1	0;0;0	0;0;0	0;0;0	0;0;0	0;0;0	0;0;0	0;0;0	1;0;1	0;0;0	0;0;0	0;0;0
groupV	9277000	9279000	2;3;	63;3;	3;0;3	0;0;0	0;0;0	1;0;1	0;0;0	0;0;0	0;0;0	2;0;2	0;0;0	0;0;0	0;0;0	0;0;0
groupV	9463500	9466000	1;2;	3;63;	3;0;3	0;0;0	0;0;0	0;0;0	0;0;0	0;0;0	0;0;0	0;0;0	2;0;2	1;0;1	0;0;0	0;0;0
groupV	9667500	9669500	1;2;	9;57;	9;0;9	0;0;0	0;0;0	1;0;1	4;0;4	2;0;2	0;0;0	2;0;2	0;0;0	0;0;0	0;0;0	0;0;0
groupV	9698000	9739000	2;3;4;	52;12;2;	16;2;12	0;0;0	0;0;0	0;0;0	1;0;1	0;0;0	0;0;0	0;0;0	3;1;1	7;1;5	2;0;2	3;0;3
groupV	9798500	9800500	0;2;	12;54;	24;12;0	0;0;0	0;0;0	0;0;0	0;0;0	0;0;0	12;6;0	12;6;0	0;0;0	0;0;0	0;0;0	0;0;0
groupV	10715500	10757500	2;3;	60;6;	6;0;6	0;0;0	0;0;0	0;0;0	0;0;0	0;0;0	0;0;0	0;0;0	0;0;0	0;0;0	1;0;1	5;0;5
groupV	11063500	11067000	1;2;	3;63;	3;0;3	0;0;0	0;0;0	0;0;0	0;0;0	0;0;0	0;0;0	0;0;0	2;0;2	0;0;0	0;0;0	1;0;1
groupV	11878500	11885500	2;3;	65;1;	1;0;1	1;0;1	0;0;0	0;0;0	0;0;0	0;0;0	0;0;0	0;0;0	0;0;0	0;0;0	0;0;0	0;0;0
groupVI	214000	224500	2;3;	65;1;	1;0;1	0;0;0	0;0;0	0;0;0	0;0;0	1;0;1	0;0;0	0;0;0	0;0;0	0;0;0	0;0;0	0;0;0
groupVI	224000	230000	0;1;2;	5;14;47;	24;5;14	2;0;2	0;0;0	6;2;2	7;2;3	5;1;3	0;0;0	4;0;4	0;0;0	0;0;0	0;0;0	0;0;0
groupVI	274000	275500	0;1;2;	1;6;59;	8;1;6	0;0;0	0;0;0	0;0;0	0;0;0	0;0;0	0;0;0	0;0;0	0;0;0	0;0;0	6;1;4	2;0;2
groupVI	508500	512500	1;2;	7;59;	7;0;7	0;0;0	0;0;0	0;0;0	0;0;0	0;0;0	0;0;0	0;0;0	2;0;2	4;0;4	0;0;0	1;0;1
groupVI	800000	802000	1;2;	24;42;	24;0;24	0;0;0	0;0;0	0;0;0	0;0;0	0;0;0	0;0;0	0;0;0	6;0;6	6;0;6	6;0;6	6;0;6
groupVI	1356500	1362000	1;2;	1;65;	1;0;1	0;0;0	0;0;0	0;0;0	0;0;0	0;0;0	0;0;0	1;0;1	0;0;0	0;0;0	0;0;0	0;0;0
groupVI	1697500	1699500	1;2;	2;64;	2;0;2	0;0;0	0;0;0	0;0;0	0;0;0	0;0;0	0;0;0	0;0;0	2;0;2	0;0;0	0;0;0	0;0;0
groupVI	1818500	1819500	0;1;2;	5;5;56;	15;5;5	0;0;0	0;0;0	0;0;0	0;0;0	0;0;0	11;5;1	4;0;4	0;0;0	0;0;0	0;0;0	0;0;0
groupVI	1986500	1993000	2;3;	65;1;	1;0;1	0;0;0	0;0;0	0;0;0	0;0;0	0;0;0	0;0;0	0;0;0	1;0;1	0;0;0	0;0;0	0;0;0
groupVI	2274500	2276000	0;1;2;	1;4;61;	6;1;4	0;0;0	0;0;0	5;1;3	0;0;0	0;0;0	0;0;0	0;0;0	0;0;0	0;0;0	0;0;0	1;0;1
groupVI	2460000	2463000	1;2;	1;65;	1;0;1	0;0;0	0;0;0	0;0;0	0;0;0	1;0;1	0;0;0	0;0;0	0;0;0	0;0;0	0;0;0	0;0;0
groupVI	3279000	3289000	2;3;	60;6;	6;0;6	0;0;0	0;0;0	0;0;0	0;0;0	0;0;0	0;0;0	0;0;0	4;0;4	1;0;1	0;0;0	1;0;1

groupVI	3324000	3325500	0;2;	1;65;	2;1;0	0;0;0	0;0;0	0;0;0	0;0;0	0;0;0	0;0;0	0;0;0	0;0;0	0;0;0	2;1;0	0;0;0
groupVI	3726500	3732000	1;2;	1;65;	1;0;1	0;0;0	0;0;0	0;0;0	1;0;1	0;0;0	0;0;0	0;0;0	0;0;0	0;0;0	0;0;0	0;0;0
groupVI	3918000	3924000	1;2;	1;65;	1;0;1	0;0;0	0;0;0	0;0;0	0;0;0	1;0;1	0;0;0	0;0;0	0;0;0	0;0;0	0;0;0	0;0;0
groupVI	4267000	4268500	1;2;	11;55;	11;0;11	1;0;1	0;0;0	0;0;0	3;0;3	1;0;1	0;0;0	1;0;1	2;0;2	1;0;1	0;0;0	2;0;2
groupVI	5065000	5083000	2;3;	64;2;	2;0;2	0;0;0	0;0;0	1;0;1	0;0;0	0;0;0	0;0;0	0;0;0	0;0;0	0;0;0	1;0;1	0;0;0
groupVI	5158000	5164500	2;3;4;	64;1;1;	3;1;1	3;1;1	0;0;0	0;0;0	0;0;0	0;0;0	0;0;0	0;0;0	0;0;0	0;0;0	0;0;0	0;0;0
groupVI	5346000	5348000	1;2;	3;63;	3;0;3	0;0;0	0;0;0	3;0;3	0;0;0	0;0;0	0;0;0	0;0;0	0;0;0	0;0;0	0;0;0	0;0;0
groupVI	5783000	5786000	1;2;	1;65;	1;0;1	0;0;0	0;0;0	0;0;0	0;0;0	0;0;0	0;0;0	0;0;0	0;0;0	1;0;1	0;0;0	0;0;0
groupVI	6189000	6190500	1;2;	5;61;	5;0;5	0;0;0	0;0;0	0;0;0	0;0;0	0;0;0	0;0;0	0;0;0	0;0;0	0;0;0	2;0;2	3;0;3
groupVI	6419000	6425500	2;3;	65;1;	1;0;1	0;0;0	0;0;0	0;0;0	0;0;0	0;0;0	0;0;0	0;0;0	0;0;0	1;0;1	0;0;0	0;0;0
groupVI	6569500	6571500	0;1;2;	1;5;60;	7;1;5	0;0;0	0;0;0	0;0;0	0;0;0	0;0;0	0;0;0	0;0;0	0;0;0	0;0;0	0;0;0	7;1;5
groupVI	6768500	6771500	0;1;2;	1;9;56;	11;1;9	0;0;0	0;0;0	4;0;4	3;1;1	1;0;1	0;0;0	0;0;0	1;0;1	2;0;2	0;0;0	0;0;0
groupVI	6877000	6879000	1;2;	1;65;	1;0;1	0;0;0	0;0;0	0;0;0	0;0;0	0;0;0	0;0;0	0;0;0	1;0;1	0;0;0	0;0;0	0;0;0
groupVI	7214500	7216000	0;1;2;	1;1;64;	3;1;1	3;1;1	0;0;0	0;0;0	0;0;0	0;0;0	0;0;0	0;0;0	0;0;0	0;0;0	0;0;0	0;0;0
groupVI	7384000	7385500	1;2;	8;58;	8;0;8	0;0;0	0;0;0	0;0;0	0;0;0	0;0;0	4;0;4	1;0;1	1;0;1	1;0;1	1;0;1	0;0;0
groupVI	7544000	7546000	0;1;2;	1;2;63;	4;1;2	0;0;0	0;0;0	0;0;0	0;0;0	3;1;1	0;0;0	0;0;0	0;0;0	0;0;0	0;0;0	1;0;1
groupVI	7594500	7601000	2;3;	65;1;	1;0;1	0;0;0	1;0;1	0;0;0	0;0;0	0;0;0	0;0;0	0;0;0	0;0;0	0;0;0	0;0;0	0;0;0
groupVI	8415000	8430000	2;3;	65;1;	1;0;1	0;0;0	0;0;0	0;0;0	0;0;0	0;0;0	0;0;0	0;0;0	0;0;0	1;0;1	0;0;0	0;0;0
groupVI	8449000	8453000	2;3;4;	59;5;2;	9;2;5	0;0;0	0;0;0	6;2;2	1;0;1	2;0;2	0;0;0	0;0;0	0;0;0	0;0;0	0;0;0	0;0;0
groupVI	8790000	8802000	2;3;	65;1;	1;0;1	1;0;1	0;0;0	0;0;0	0;0;0	0;0;0	0;0;0	0;0;0	0;0;0	0;0;0	0;0;0	0;0;0
groupVI	9305000	9312500	2;3;	65;1;	1;0;1	0;0;0	0;0;0	0;0;0	0;0;0	0;0;0	0;0;0	1;0;1	0;0;0	0;0;0	0;0;0	0;0;0
groupVI	9933500	9944000	2;4;	65;1;	2;1;0	0;0;0	0;0;0	0;0;0	0;0;0	0;0;0	0;0;0	2;1;0	0;0;0	0;0;0	0;0;0	0;0;0
groupVI	10913500	10916000	2;3;	65;1;	1;0;1	0;0;0	0;0;0	0;0;0	1;0;1	0;0;0	0;0;0	0;0;0	0;0;0	0;0;0	0;0;0	0;0;0
groupVI	11689500	11692000	1;2;	1;65;	1;0;1	1;0;1	0;0;0	0;0;0	0;0;0	0;0;0	0;0;0	0;0;0	0;0;0	0;0;0	0;0;0	0;0;0
groupVI	11747500	11749000	0;1;2;	2;3;61;	7;2;3	0;0;0	0;0;0	0;0;0	0;0;0	0;0;0	6;2;2	0;0;0	0;0;0	0;0;0	1;0;1	0;0;0
groupVI	12005000	12008500	1;2;	1;65;	1;0;1	1;0;1	0;0;0	0;0;0	0;0;0	0;0;0	0;0;0	0;0;0	0;0;0	0;0;0	0;0;0	0;0;0
groupVI	13256500	13263000	2;3;	65;1;	1;0;1	0;0;0	0;0;0	0;0;0	1;0;1	0;0;0	0;0;0	0;0;0	0;0;0	0;0;0	0;0;0	0;0;0
groupVI	13824500	13826000	1;2;	5;61;	5;0;5	1;0;1	2;0;2	1;0;1	0;0;0	0;0;0	0;0;0	1;0;1	0;0;0	0;0;0	0;0;0	0;0;0
groupVI	14034500	14039500	1;2;	1;65;	1;0;1	0;0;0	0;0;0	0;0;0	0;0;0	0;0;0	0;0;0	1;0;1	0;0;0	0;0;0	0;0;0	0;0;0
groupVI	14293500	14304500	2;3;	65;1;	1;0;1	0;0;0	0;0;0	0;0;0	0;0;0	0;0;0	0;0;0	0;0;0	1;0;1	0;0;0	0;0;0	0;0;0
groupVI	14575000	14580500	2;3;4;	52;11;3;	17;3;11	0;0;0	6;2;2	2;0;2	4;1;2	1;0;1	0;0;0	0;0;0	0;0;0	2;0;2	0;0;0	2;0;2
groupVI	14726500	14730500	1;2;	3;63;	3;0;3	0;0;0	0;0;0	0;0;0	3;0;3	0;0;0	0;0;0	0;0;0	0;0;0	0;0;0	0;0;0	0;0;0
groupVI	15064500	15066500	1;2;	2;64;	2;0;2	0;0;0	2;0;2	0;0;0	0;0;0	0;0;0	0;0;0	0;0;0	0;0;0	0;0;0	0;0;0	0;0;0
groupVI	15071000	15078000	1;2;	1;65;	1;0;1	0;0;0	0;0;0	0;0;0	0;0;0	1;0;1	0;0;0	0;0;0	0;0;0	0;0;0	0;0;0	0;0;0

groupVI	15126000	15148000	1;2;	1;65;	1;0;1	0;0;0	0;0;0	0;0;0	0;0;0	0;0;0	0;0;0	1;0;1	0;0;0	0;0;0	0;0;0	0;0;0
groupVI	15428000	15436000	2;3;	65;1;	1;0;1	0;0;0	0;0;0	0;0;0	0;0;0	0;0;0	0;0;0	1;0;1	0;0;0	0;0;0	0;0;0	0;0;0
groupVI	15736500	15743000	1;2;	1;65;	1;0;1	0;0;0	0;0;0	0;0;0	1;0;1	0;0;0	0;0;0	0;0;0	0;0;0	0;0;0	0;0;0	0;0;0
groupVI	15753000	15758000	1;2;	1;65;	1;0;1	0;0;0	0;0;0	0;0;0	0;0;0	0;0;0	0;0;0	0;0;0	0;0;0	0;0;0	0;0;0	1;0;1
groupVI	16011000	16014500	1;2;	1;65;	1;0;1	0;0;0	0;0;0	0;0;0	0;0;0	1;0;1	0;0;0	0;0;0	0;0;0	0;0;0	0;0;0	0;0;0
groupVI	16352000	16353500	1;2;	12;54;	12;0;12	1;0;1	0;0;0	2;0;2	1;0;1	3;0;3	0;0;0	0;0;0	3;0;3	2;0;2	0;0;0	0;0;0
groupVI	16691500	16694500	0;1;2;	1;2;63;	4;1;2	0;0;0	0;0;0	0;0;0	0;0;0	0;0;0	0;0;0	0;0;0	0;0;0	0;0;0	4;1;2	0;0;0
groupVII	126500	128000	0;1;2;	1;17;48;	19;1;17	1;0;1	6;0;6	3;0;3	5;0;5	3;1;1	0;0;0	1;0;1	0;0;0	0;0;0	0;0;0	0;0;0
groupVII	214500	221500	2;3;4;	61;4;1;	6;1;4	0;0;0	0;0;0	1;0;1	1;0;1	4;1;2	0;0;0	0;0;0	0;0;0	0;0;0	0;0;0	0;0;0
groupVII	503500	509500	2;3;	65;1;	1;0;1	0;0;0	0;0;0	1;0;1	0;0;0	0;0;0	0;0;0	0;0;0	0;0;0	0;0;0	0;0;0	0;0;0
groupVII	862500	874500	1;2;	1;65;	1;0;1	0;0;0	0;0;0	0;0;0	0;0;0	0;0;0	0;0;0	0;0;0	0;0;0	0;0;0	1;0;1	0;0;0
groupVII	907000	910000	1;2;	2;64;	2;0;2	0;0;0	0;0;0	0;0;0	0;0;0	0;0;0	0;0;0	0;0;0	0;0;0	0;0;0	0;0;0	2;0;2
groupVII	1275000	1278000	1;2;	3;63;	3;0;3	0;0;0	0;0;0	0;0;0	0;0;0	0;0;0	0;0;0	0;0;0	1;0;1	1;0;1	1;0;1	0;0;0
groupVII	1555500	1573000	2;3;	63;3;	3;0;3	0;0;0	0;0;0	2;0;2	0;0;0	1;0;1	0;0;0	0;0;0	0;0;0	0;0;0	0;0;0	0;0;0
groupVII	1860000	1874000	2;3;	61;5;	5;0;5	0;0;0	0;0;0	0;0;0	0;0;0	0;0;0	0;0;0	0;0;0	2;0;2	0;0;0	3;0;3	0;0;0
groupVII	2779500	2783000	1;2;	2;64;	2;0;2	0;0;0	0;0;0	0;0;0	0;0;0	0;0;0	0;0;0	2;0;2	0;0;0	0;0;0	0;0;0	0;0;0
groupVII	2803500	2811000	2;3;	63;3;	3;0;3	1;0;1	0;0;0	0;0;0	2;0;2	0;0;0	0;0;0	0;0;0	0;0;0	0;0;0	0;0;0	0;0;0
groupVII	2945500	2951500	1;2;	1;65;	1;0;1	0;0;0	0;0;0	0;0;0	0;0;0	0;0;0	0;0;0	0;0;0	1;0;1	0;0;0	0;0;0	0;0;0
groupVII	2970000	2982500	2;3;	65;1;	1;0;1	0;0;0	0;0;0	0;0;0	1;0;1	0;0;0	0;0;0	0;0;0	0;0;0	0;0;0	0;0;0	0;0;0
groupVII	3089500	3096000	2;3;	65;1;	1;0;1	0;0;0	0;0;0	0;0;0	0;0;0	0;0;0	0;0;0	1;0;1	0;0;0	0;0;0	0;0;0	0;0;0
groupVII	3316000	3319500	1;2;	2;64;	2;0;2	0;0;0	0;0;0	0;0;0	0;0;0	0;0;0	0;0;0	0;0;0	0;0;0	0;0;0	0;0;0	2;0;2
groupVII	3433500	3435500	1;2;	4;62;	4;0;4	3;0;3	0;0;0	0;0;0	0;0;0	0;0;0	0;0;0	0;0;0	0;0;0	1;0;1	0;0;0	0;0;0
groupVII	3890500	3892500	1;2;	2;64;	2;0;2	0;0;0	0;0;0	0;0;0	0;0;0	0;0;0	0;0;0	0;0;0	1;0;1	1;0;1	0;0;0	0;0;0
groupVII	3964000	3967500	1;2;	3;63;	3;0;3	0;0;0	0;0;0	0;0;0	0;0;0	0;0;0	0;0;0	0;0;0	0;0;0	1;0;1	1;0;1	1;0;1
groupVII	4288500	4295500	2;3;	65;1;	1;0;1	0;0;0	0;0;0	0;0;0	0;0;0	0;0;0	0;0;0	0;0;0	0;0;0	0;0;0	0;0;0	1;0;1
groupVII	4685500	4689000	1;2;	3;63;	3;0;3	0;0;0	0;0;0	0;0;0	2;0;2	1;0;1	0;0;0	0;0;0	0;0;0	0;0;0	0;0;0	0;0;0
groupVII	4809000	4811000	0;1;2;	9;6;51;	24;9;6	0;0;0	0;0;0	0;0;0	0;0;0	0;0;0	0;0;0	0;0;0	4;1;2	10;4;2	10;4;2	0;0;0
groupVII	4961500	4964000	1;2;	2;64;	2;0;2	0;0;0	0;0;0	0;0;0	2;0;2	0;0;0	0;0;0	0;0;0	0;0;0	0;0;0	0;0;0	0;0;0
groupVII	4970000	4972000	1;2;	6;60;	6;0;6	0;0;0	0;0;0	0;0;0	0;0;0	2;0;2	0;0;0	0;0;0	1;0;1	2;0;2	0;0;0	1;0;1
groupVII	5032500	5041000	2;3;	65;1;	1;0;1	1;0;1	0;0;0	0;0;0	0;0;0	0;0;0	0;0;0	0;0;0	0;0;0	0;0;0	0;0;0	0;0;0
groupVII	6396500	6400500	1;2;	1;65;	1;0;1	0;0;0	0;0;0	1;0;1	0;0;0	0;0;0	0;0;0	0;0;0	0;0;0	0;0;0	0;0;0	0;0;0
groupVII	6514500	6516000	1;2;	2;64;	2;0;2	0;0;0	0;0;0	0;0;0	0;0;0	1;0;1	0;0;0	0;0;0	0;0;0	1;0;1	0;0;0	0;0;0
groupVII	6899500	6901500	0;1;2;	3;7;56;	13;3;7	0;0;0	0;0;0	0;0;0	0;0;0	0;0;0	0;0;0	0;0;0	7;1;5	4;2;0	1;0;1	1;0;1
groupVII	7228500	7230500	0;1;2;	1;9;56;	11;1;9	0;0;0	0;0;0	0;0;0	0;0;0	0;0;0	0;0;0	0;0;0	5;0;5	5;1;3	1;0;1	0;0;0

groupVII	7633500	7636000	1;2;	2;64;	2;0;2	0;0;0	0;0;0	1;0;1	0;0;0	1;0;1	0;0;0	0;0;0	0;0;0	0;0;0	0;0;0	0;0;0
groupVII	7833000	7844500	2;3;	64;2;	2;0;2	0;0;0	1;0;1	1;0;1	0;0;0	0;0;0	0;0;0	0;0;0	0;0;0	0;0;0	0;0;0	0;0;0
groupVII	8114500	8115500	0;1;2;	2;4;60;	8;2;4	0;0;0	0;0;0	0;0;0	0;0;0	0;0;0	0;0;0	0;0;0	1;0;1	0;0;0	7;2;3	0;0;0
groupVII	8159500	8168500	2;3;	65;1;	1;0;1	0;0;0	0;0;0	0;0;0	0;0;0	1;0;1	0;0;0	0;0;0	0;0;0	0;0;0	0;0;0	0;0;0
groupVII	8739000	8742000	1;2;	7;59;	7;0;7	0;0;0	0;0;0	0;0;0	2;0;2	0;0;0	0;0;0	0;0;0	1;0;1	0;0;0	3;0;3	1;0;1
groupVII	8851000	8852500	0;1;2;	8;8;50;	24;8;8	0;0;0	0;0;0	0;0;0	0;0;0	0;0;0	0;0;0	0;0;0	3;0;3	5;2;1	12;6;0	4;0;4
groupVII	8953500	8957000	1;2;	18;48;	18;0;18	0;0;0	0;0;0	0;0;0	0;0;0	0;0;0	0;0;0	0;0;0	4;0;4	5;0;5	6;0;6	3;0;3
groupVII	8988000	8992500	1;2;	20;46;	20;0;20	0;0;0	0;0;0	0;0;0	0;0;0	0;0;0	0;0;0	0;0;0	6;0;6	4;0;4	6;0;6	4;0;4
groupVII	9154000	9167500	1;2;	6;60;	6;0;6	1;0;1	0;0;0	0;0;0	0;0;0	0;0;0	0;0;0	0;0;0	0;0;0	1;0;1	2;0;2	2;0;2
groupVII	9241000	9243000	2;3;	64;2;	2;0;2	0;0;0	1;0;1	0;0;0	1;0;1	0;0;0	0;0;0	0;0;0	0;0;0	0;0;0	0;0;0	0;0;0
groupVII	9336000	9340500	2;3;	60;6;	6;0;6	0;0;0	0;0;0	0;0;0	0;0;0	0;0;0	0;0;0	0;0;0	3;0;3	2;0;2	1;0;1	0;0;0
groupVII	9409500	9414500	1;2;	8;58;	8;0;8	0;0;0	0;0;0	6;0;6	0;0;0	2;0;2	0;0;0	0;0;0	0;0;0	0;0;0	0;0;0	0;0;0
groupVII	9629500	9632500	1;2;	10;56;	10;0;10	0;0;0	0;0;0	0;0;0	0;0;0	0;0;0	0;0;0	0;0;0	0;0;0	0;0;0	6;0;6	4;0;4
groupVII	9649500	9652500	1;2;	1;65;	1;0;1	1;0;1	0;0;0	0;0;0	0;0;0	0;0;0	0;0;0	0;0;0	0;0;0	0;0;0	0;0;0	0;0;0
groupVII	9767000	9768000	0;1;2;	2;3;61;	7;2;3	0;0;0	0;0;0	0;0;0	0;0;0	0;0;0	0;0;0	0;0;0	0;0;0	0;0;0	6;2;2	1;0;1
groupVII	10031500	10035500	1;2;	5;61;	5;0;5	0;0;0	0;0;0	0;0;0	0;0;0	0;0;0	2;0;2	2;0;2	0;0;0	0;0;0	1;0;1	0;0;0
groupVII	10115500	10118000	0;1;2;	2;6;58;	10;2;6	0;0;0	0;0;0	0;0;0	0;0;0	0;0;0	0;0;0	0;0;0	1;0;1	1;0;1	7;2;3	1;0;1
groupVII	10200500	10210500	2;3;	65;1;	1;0;1	0;0;0	0;0;0	0;0;0	0;0;0	0;0;0	0;0;0	0;0;0	0;0;0	0;0;0	0;0;0	1;0;1
groupVII	10615000	10618500	1;2;	1;65;	1;0;1	0;0;0	0;0;0	0;0;0	0;0;0	0;0;0	0;0;0	1;0;1	0;0;0	0;0;0	0;0;0	0;0;0
groupVII	10624000	10625500	0;1;2;	1;2;63;	4;1;2	4;1;2	0;0;0	0;0;0	0;0;0	0;0;0	0;0;0	0;0;0	0;0;0	0;0;0	0;0;0	0;0;0
groupVII	10645000	10646500	1;2;	2;64;	2;0;2	0;0;0	0;0;0	1;0;1	0;0;0	1;0;1	0;0;0	0;0;0	0;0;0	0;0;0	0;0;0	0;0;0
groupVII	10745500	10750000	1;2;	2;64;	2;0;2	0;0;0	0;0;0	0;0;0	0;0;0	0;0;0	0;0;0	0;0;0	2;0;2	0;0;0	0;0;0	0;0;0
groupVII	10882500	10885500	1;2;	1;65;	1;0;1	0;0;0	0;0;0	0;0;0	0;0;0	0;0;0	0;0;0	0;0;0	1;0;1	0;0;0	0;0;0	0;0;0
groupVII	11375000	11378500	0;1;2;	1;6;59;	8;1;6	0;0;0	0;0;0	0;0;0	0;0;0	0;0;0	0;0;0	0;0;0	1;0;1	0;0;0	0;0;0	7;1;5
groupVII	11394000	11401000	2;3;	65;1;	1;0;1	1;0;1	0;0;0	0;0;0	0;0;0	0;0;0	0;0;0	0;0;0	0;0;0	0;0;0	0;0;0	0;0;0
groupVII	11428500	11436500	2;3;	65;1;	1;0;1	0;0;0	0;0;0	0;0;0	0;0;0	1;0;1	0;0;0	0;0;0	0;0;0	0;0;0	0;0;0	0;0;0
groupVII	11448000	11450500	0;1;2;	4;12;50;	20;4;12	0;0;0	7;1;5	4;1;2	6;1;4	3;1;1	0;0;0	0;0;0	0;0;0	0;0;0	0;0;0	0;0;0
groupVII	11518000	11520000	1;2;	6;60;	6;0;6	0;0;0	0;0;0	3;0;3	0;0;0	0;0;0	0;0;0	1;0;1	0;0;0	0;0;0	0;0;0	2;0;2
groupVII	11601500	11606000	1;2;	1;65;	1;0;1	0;0;0	0;0;0	0;0;0	0;0;0	0;0;0	0;0;0	1;0;1	0;0;0	0;0;0	0;0;0	0;0;0
groupVII	11647500	11651500	1;2;	2;64;	2;0;2	0;0;0	0;0;0	0;0;0	1;0;1	1;0;1	0;0;0	0;0;0	0;0;0	0;0;0	0;0;0	0;0;0
groupVII	11788500	11795000	0;1;2;	2;3;61;	7;2;3	0;0;0	0;0;0	0;0;0	0;0;0	0;0;0	0;0;0	0;0;0	0;0;0	0;0;0	5;2;1	2;0;2
groupVII	12006000	12008500	1;2;	1;65;	1;0;1	1;0;1	0;0;0	0;0;0	0;0;0	0;0;0	0;0;0	0;0;0	0;0;0	0;0;0	0;0;0	0;0;0
groupVII	12041000	12043500	0;1;2;	1;5;60;	7;1;5	0;0;0	0;0;0	0;0;0	0;0;0	0;0;0	0;0;0	0;0;0	5;1;3	0;0;0	1;0;1	1;0;1
groupVII	12084500	12090500	2;3;	60;6;	6;0;6	0;0;0	0;0;0	0;0;0	0;0;0	0;0;0	0;0;0	0;0;0	3;0;3	2;0;2	1;0;1	0;0;0

groupVII	12226500	12228000	1;2;	2;64;	2;0;2	1;0;1	0;0;0	0;0;0	0;0;0	0;0;0	0;0;0	1;0;1	0;0;0	0;0;0	0;0;0	0;0;0
groupVII	12263500	12265000	0;1;2;	3;11;52;	17;3;11	5;1;3	6;1;4	1;0;1	0;0;0	4;1;2	0;0;0	1;0;1	0;0;0	0;0;0	0;0;0	0;0;0
groupVII	12331000	12332500	2;3;4;	47;12;7;	26;7;12	0;0;0	0;0;0	0;0;0	0;0;0	0;0;0	0;0;0	1;0;1	2;0;2	6;1;4	10;4;2	7;2;3
groupVII	12691500	12692500	0;1;2;	8;13;45;	29;8;13	0;0;0	0;0;0	0;0;0	0;0;0	0;0;0	0;0;0	0;0;0	3;0;3	8;2;4	12;6;0	6;0;6
groupVII	12699000	12702000	0;1;2;	11;10;45;	32;11;1(	0;0;0	0;0;0	0;0;0	0;0;0	0;0;0	0;0;0	0;0;0	4;1;2	10;4;2	10;4;2	8;2;4
groupVII	12965500	12968500	2;3;4;	40;24;2;	28;2;24	1;0;1	2;1;0	0;0;0	0;0;0	0;0;0	0;0;0	2;0;2	5;0;5	6;0;6	7;1;5	5;0;5
groupVII	13013500	13023500	2;3;	60;6;	6;0;6	0;0;0	0;0;0	0;0;0	0;0;0	0;0;0	0;0;0	0;0;0	0;0;0	5;0;5	0;0;0	1;0;1
groupVII	13024500	13027500	1;2;	2;64;	2;0;2	0;0;0	0;0;0	0;0;0	0;0;0	1;0;1	0;0;0	1;0;1	0;0;0	0;0;0	0;0;0	0;0;0
groupVII	13083500	13086500	1;2;	2;64;	2;0;2	0;0;0	0;0;0	0;0;0	0;0;0	0;0;0	0;0;0	0;0;0	0;0;0	0;0;0	1;0;1	1;0;1
groupVII	13109000	13111000	2;3;	65;1;	1;0;1	0;0;0	0;0;0	1;0;1	0;0;0	0;0;0	0;0;0	0;0;0	0;0;0	0;0;0	0;0;0	0;0;0
groupVII	13197000	13203500	2;3;	48;18;	18;0;18	0;0;0	0;0;0	0;0;0	0;0;0	0;0;0	0;0;0	0;0;0	1;0;1	6;0;6	6;0;6	5;0;5
groupVII	13350500	13356000	1;2;	1;65;	1;0;1	1;0;1	0;0;0	0;0;0	0;0;0	0;0;0	0;0;0	0;0;0	0;0;0	0;0;0	0;0;0	0;0;0
groupVII	13467000	13469000	2;3;	44;22;	22;0;22	0;0;0	0;0;0	0;0;0	0;0;0	0;0;0	0;0;0	0;0;0	6;0;6	6;0;6	6;0;6	4;0;4
groupVII	13534500	13547500	2;3;	64;2;	2;0;2	2;0;2	0;0;0	0;0;0	0;0;0	0;0;0	0;0;0	0;0;0	0;0;0	0;0;0	0;0;0	0;0;0
groupVII	13838000	13839500	0;1;2;	2;7;57;	11;2;7	0;0;0	0;0;0	0;0;0	0;0;0	0;0;0	0;0;0	0;0;0	4;0;4	1;0;1	3;1;1	3;1;1
groupVII	14390000	14391000	0;1;2;	7;8;51;	22;7;8	0;0;0	0;0;0	0;0;0	0;0;0	0;0;0	0;0;0	0;0;0	10;4;2	8;2;4	0;0;0	4;1;2
groupVII	14780000	14781000	0;1;2;	1;14;51;	16;1;14	0;0;0	0;0;0	0;0;0	0;0;0	0;0;0	0;0;0	0;0;0	3;1;1	4;0;4	4;0;4	5;0;5
groupVII	15008500	15011500	1;2;	2;64;	2;0;2	0;0;0	2;0;2	0;0;0	0;0;0	0;0;0	0;0;0	0;0;0	0;0;0	0;0;0	0;0;0	0;0;0
groupVII	15214000	15217500	2;3;	61;5;	5;0;5	0;0;0	0;0;0	0;0;0	0;0;0	0;0;0	0;0;0	0;0;0	0;0;0	3;0;3	0;0;0	2;0;2
groupVII	15362000	15364000	0;1;2;	2;10;54;	14;2;10	0;0;0	0;0;0	0;0;0	0;0;0	0;0;0	0;0;0	0;0;0	7;2;3	0;0;0	6;0;6	1;0;1
groupVII	15535500	15538000	2;3;	54;12;	12;0;12	0;0;0	0;0;0	0;0;0	0;0;0	0;0;0	0;0;0	0;0;0	2;0;2	4;0;4	4;0;4	2;0;2
groupVII	16120500	16122500	0;1;2;	2;12;52;	16;2;12	0;0;0	0;0;0	0;0;0	0;0;0	0;0;0	0;0;0	0;0;0	2;0;2	0;0;0	7;1;5	7;1;5
groupVII	16480000	16481000	0;1;2;	2;4;60;	8;2;4	2;0;2	6;2;2	0;0;0	0;0;0	0;0;0	0;0;0	0;0;0	0;0;0	0;0;0	0;0;0	0;0;0
groupVII	16664000	16670500	2;3;	63;3;	3;0;3	0;0;0	0;0;0	3;0;3	0;0;0	0;0;0	0;0;0	0;0;0	0;0;0	0;0;0	0;0;0	0;0;0
groupVII	16814000	16823500	2;3;	65;1;	1;0;1	0;0;0	0;0;0	0;0;0	0;0;0	1;0;1	0;0;0	0;0;0	0;0;0	0;0;0	0;0;0	0;0;0
groupVII	17375000	17381000	2;3;	63;3;	3;0;3	0;0;0	3;0;3	0;0;0	0;0;0	0;0;0	0;0;0	0;0;0	0;0;0	0;0;0	0;0;0	0;0;0
groupVII	17431000	17434000	0;1;2;	1;3;62;	5;1;3	0;0;0	0;0;0	0;0;0	0;0;0	0;0;0	0;0;0	0;0;0	0;0;0	5;1;3	0;0;0	0;0;0
groupVII	17460500	17462000	2;3;	62;4;	4;0;4	0;0;0	0;0;0	0;0;0	0;0;0	0;0;0	0;0;0	0;0;0	2;0;2	2;0;2	0;0;0	0;0;0
groupVII	17730000	17755000	2;3;	49;17;	17;0;17	0;0;0	0;0;0	0;0;0	0;0;0	0;0;0	0;0;0	0;0;0	5;0;5	5;0;5	4;0;4	3;0;3
groupVII	18237000	18251500	2;3;	55;11;	11;0;11	0;0;0	0;0;0	0;0;0	0;0;0	0;0;0	0;0;0	0;0;0	4;0;4	4;0;4	0;0;0	3;0;3
groupVII	18546500	18554000	2;3;4;	49;9;8;	25;8;9	0;0;0	0;0;0	0;0;0	0;0;0	0;0;0	0;0;0	0;0;0	1;0;1	4;0;4	9;3;3	11;5;1
groupVII	18905500	18917000	2;3;	61;5;	5;0;5	1;0;1	1;0;1	2;0;2	0;0;0	1;0;1	0;0;0	0;0;0	0;0;0	0;0;0	0;0;0	0;0;0
groupVII	19445000	19447000	1;2;	2;64;	2;0;2	0;0;0	1;0;1	0;0;0	1;0;1	0;0;0	0;0;0	0;0;0	0;0;0	0;0;0	0;0;0	0;0;0
groupVII	19666000	19672500	1;2;	4;62;	4;0;4	0;0;0	0;0;0	0;0;0	2;0;2	0;0;0	0;0;0	0;0;0	0;0;0	1;0;1	1;0;1	0;0;0

groupVII	19672500	19726500	2;3;	59;7;	7;0;7	0;0;0	0;0;0	0;0;0	3;0;3	0;0;0	4;0;4	0;0;0	0;0;0	0;0;0	0;0;0	0;0;0
groupVII	19727500	19730000	1;2;	3;63;	3;0;3	0;0;0	0;0;0	0;0;0	0;0;0	0;0;0	0;0;0	0;0;0	0;0;0	3;0;3	0;0;0	0;0;0
groupVII	19761000	19793500	1;2;	5;61;	5;0;5	0;0;0	0;0;0	1;0;1	0;0;0	0;0;0	0;0;0	0;0;0	1;0;1	3;0;3	0;0;0	0;0;0
groupVII	19835000	19836500	0;1;2;	3;13;50;	19;3;13	0;0;0	0;0;0	0;0;0	0;0;0	0;0;0	0;0;0	0;0;0	8;2;4	3;0;3	3;0;3	5;1;3
groupVII	20617500	20620000	1;2;	1;65;	1;0;1	0;0;0	1;0;1	0;0;0	0;0;0	0;0;0	0;0;0	0;0;0	0;0;0	0;0;0	0;0;0	0;0;0
groupVII	20737000	20757000	2;3;	62;4;	4;0;4	1;0;1	0;0;0	0;0;0	0;0;0	0;0;0	0;0;0	0;0;0	0;0;0	0;0;0	3;0;3	0;0;0
groupVII	20815000	20816000	0;1;2;	2;5;59;	9;2;5	6;1;4	0;0;0	0;0;0	1;0;1	2;1;0	0;0;0	0;0;0	0;0;0	0;0;0	0;0;0	0;0;0
groupVII	21373500	21378000	2;3;	65;1;	1;0;1	1;0;1	0;0;0	0;0;0	0;0;0	0;0;0	0;0;0	0;0;0	0;0;0	0;0;0	0;0;0	0;0;0
groupVII	21563500	21569000	2;3;	64;2;	2;0;2	0;0;0	0;0;0	0;0;0	0;0;0	1;0;1	0;0;0	1;0;1	0;0;0	0;0;0	0;0;0	0;0;0
groupVII	24691500	24721000	1;2;	1;65;	1;0;1	0;0;0	0;0;0	1;0;1	0;0;0	0;0;0	0;0;0	0;0;0	0;0;0	0;0;0	0;0;0	0;0;0
groupVII	24727000	24737500	2;3;	65;1;	1;0;1	0;0;0	0;0;0	0;0;0	0;0;0	0;0;0	0;0;0	0;0;0	0;0;0	1;0;1	0;0;0	0;0;0
groupVII	25182500	25193000	2;3;	63;3;	3;0;3	0;0;0	0;0;0	0;0;0	1;0;1	2;0;2	0;0;0	0;0;0	0;0;0	0;0;0	0;0;0	0;0;0
groupVII	25623500	25626000	1;2;	1;65;	1;0;1	1;0;1	0;0;0	0;0;0	0;0;0	0;0;0	0;0;0	0;0;0	0;0;0	0;0;0	0;0;0	0;0;0
groupVII	25724500	25727000	1;2;	1;65;	1;0;1	0;0;0	0;0;0	0;0;0	0;0;0	0;0;0	0;0;0	1;0;1	0;0;0	0;0;0	0;0;0	0;0;0
groupVII	26005000	26007000	2;3;	65;1;	1;0;1	0;0;0	0;0;0	0;0;0	1;0;1	0;0;0	0;0;0	0;0;0	0;0;0	0;0;0	0;0;0	0;0;0
groupVII	26007500	26022500	2;3;	65;1;	1;0;1	0;0;0	0;0;0	1;0;1	0;0;0	0;0;0	0;0;0	0;0;0	0;0;0	0;0;0	0;0;0	0;0;0
groupVII	26090500	26093000	1;2;	1;65;	1;0;1	1;0;1	0;0;0	0;0;0	0;0;0	0;0;0	0;0;0	0;0;0	0;0;0	0;0;0	0;0;0	0;0;0
groupVII	26229500	26231500	1;2;	7;59;	7;0;7	1;0;1	0;0;0	4;0;4	0;0;0	1;0;1	0;0;0	0;0;0	0;0;0	0;0;0	0;0;0	1;0;1
groupVII	26277000	26279000	1;2;	1;65;	1;0;1	0;0;0	0;0;0	0;0;0	0;0;0	0;0;0	0;0;0	0;0;0	0;0;0	0;0;0	0;0;0	1;0;1
groupVII	26975500	26988500	2;4;	65;1;	2;1;0	0;0;0	0;0;0	0;0;0	0;0;0	2;1;0	0;0;0	0;0;0	0;0;0	0;0;0	0;0;0	0;0;0
groupVII	27145000	27164500	2;3;4;	51;12;3;	18;3;12	0;0;0	1;0;1	5;1;3	1;0;1	5;2;1	0;0;0	0;0;0	1;0;1	0;0;0	3;0;3	2;0;2
groupVII	27582000	27588000	2;3;	65;1;	1;0;1	0;0;0	0;0;0	0;0;0	0;0;0	0;0;0	0;0;0	1;0;1	0;0;0	0;0;0	0;0;0	0;0;0
groupVIII	39500	42000	0;1;2;	1;9;56;	11;1;9	0;0;0	6;1;4	3;0;3	1;0;1	1;0;1	0;0;0	0;0;0	0;0;0	0;0;0	0;0;0	0;0;0
groupVIII	200500	213500	2;3;	65;1;	1;0;1	0;0;0	0;0;0	0;0;0	0;0;0	0;0;0	0;0;0	1;0;1	0;0;0	0;0;0	0;0;0	0;0;0
groupVIII	251500	270500	1;2;	1;65;	1;0;1	0;0;0	0;0;0	0;0;0	0;0;0	1;0;1	0;0;0	0;0;0	0;0;0	0;0;0	0;0;0	0;0;0
groupVIII	326500	328500	1;2;	3;63;	3;0;3	1;0;1	0;0;0	0;0;0	2;0;2	0;0;0	0;0;0	0;0;0	0;0;0	0;0;0	0;0;0	0;0;0
groupVIII	369000	373500	0;1;2;	4;5;57;	13;4;5	0;0;0	0;0;0	8;3;2	0;0;0	4;1;2	0;0;0	0;0;0	0;0;0	1;0;1	0;0;0	0;0;0
groupVIII	408500	416500	1;2;	2;64;	2;0;2	0;0;0	0;0;0	0;0;0	0;0;0	0;0;0	0;0;0	0;0;0	0;0;0	0;0;0	0;0;0	2;0;2
groupVIII	577500	584000	2;3;4;	56;7;3;	13;3;7	0;0;0	0;0;0	5;1;3	2;0;2	6;2;2	0;0;0	0;0;0	0;0;0	0;0;0	0;0;0	0;0;0
groupVIII	638000	639500	0;1;2;	2;3;61;	7;2;3	0;0;0	0;0;0	0;0;0	0;0;0	0;0;0	2;0;2	5;2;1	0;0;0	0;0;0	0;0;0	0;0;0
groupVIII	1189500	1193000	1;2;	2;64;	2;0;2	1;0;1	0;0;0	0;0;0	0;0;0	0;0;0	0;0;0	1;0;1	0;0;0	0;0;0	0;0;0	0;0;0
groupVIII	1260500	1261500	0;1;2;	4;2;60;	10;4;2	0;0;0	0;0;0	0;0;0	0;0;0	0;0;0	0;0;0	0;0;0	0;0;0	0;0;0	9;4;1	1;0;1
groupVIII	1338000	1341000	2;3;4;	42;15;9;	33;9;15	0;0;0	0;0;0	0;0;0	0;0;0	0;0;0	0;0;0	0;0;0	7;1;5	6;0;6	10;4;2	10;4;2
groupVIII	1355000	1364000	2;4;	65;1;	2;1;0	2;1;0	0;0;0	0;0;0	0;0;0	0;0;0	0;0;0	0;0;0	0;0;0	0;0;0	0;0;0	0;0;0

groupVIII	2062000	2064500	1;2;	2;64;	2;0;2	0;0;0	0;0;0	0;0;0	0;0;0	2;0;2	0;0;0	0;0;0	0;0;0	0;0;0	0;0;0	0;0;0
groupVIII	2394500	2402000	2;3;	65;1;	1;0;1	1;0;1	0;0;0	0;0;0	0;0;0	0;0;0	0;0;0	0;0;0	0;0;0	0;0;0	0;0;0	0;0;0
groupVIII	3248500	3259500	2;3;	65;1;	1;0;1	0;0;0	0;0;0	0;0;0	1;0;1	0;0;0	0;0;0	0;0;0	0;0;0	0;0;0	0;0;0	0;0;0
groupVIII	3936500	3941500	1;2;	1;65;	1;0;1	1;0;1	0;0;0	0;0;0	0;0;0	0;0;0	0;0;0	0;0;0	0;0;0	0;0;0	0;0;0	0;0;0
groupVIII	4247500	4249000	0;1;2;	1;2;63;	4;1;2	0;0;0	0;0;0	2;1;0	0;0;0	1;0;1	0;0;0	0;0;0	0;0;0	1;0;1	0;0;0	0;0;0
groupVIII	4536500	4541000	0;1;2;	1;2;63;	4;1;2	0;0;0	2;0;2	0;0;0	2;1;0	0;0;0	0;0;0	0;0;0	0;0;0	0;0;0	0;0;0	0;0;0
groupVIII	4814500	4851000	2;3;	56;10;	10;0;10	3;0;3	1;0;1	0;0;0	0;0;0	1;0;1	0;0;0	0;0;0	2;0;2	1;0;1	2;0;2	0;0;0
groupVIII	5139500	5142000	1;2;	1;65;	1;0;1	0;0;0	0;0;0	0;0;0	1;0;1	0;0;0	0;0;0	0;0;0	0;0;0	0;0;0	0;0;0	0;0;0
groupVIII	5313000	5314000	0;1;2;	5;6;55;	16;5;6	0;0;0	0;0;0	0;0;0	0;0;0	0;0;0	0;0;0	0;0;0	2;0;2	0;0;0	8;3;2	6;2;2
groupVIII	5316500	5319000	1;2;	6;60;	6;0;6	1;0;1	0;0;0	0;0;0	0;0;0	0;0;0	0;0;0	0;0;0	1;0;1	1;0;1	3;0;3	0;0;0
groupVIII	5511500	5513500	0;1;2;	1;1;64;	3;1;1	0;0;0	0;0;0	3;1;1	0;0;0	0;0;0	0;0;0	0;0;0	0;0;0	0;0;0	0;0;0	0;0;0
groupVIII	6636000	6637000	0;1;2;	1;7;58;	9;1;7	0;0;0	0;0;0	0;0;0	0;0;0	0;0;0	0;0;0	0;0;0	3;0;3	6;1;4	0;0;0	0;0;0
groupVIII	7354000	7360500	1;2;	1;65;	1;0;1	0;0;0	0;0;0	0;0;0	0;0;0	0;0;0	0;0;0	0;0;0	1;0;1	0;0;0	0;0;0	0;0;0
groupVIII	7888500	7890000	1;2;	3;63;	3;0;3	0;0;0	0;0;0	0;0;0	1;0;1	1;0;1	0;0;0	0;0;0	0;0;0	1;0;1	0;0;0	0;0;0
groupVIII	8227500	8243000	2;4;	65;1;	2;1;0	0;0;0	0;0;0	0;0;0	0;0;0	2;1;0	0;0;0	0;0;0	0;0;0	0;0;0	0;0;0	0;0;0
groupVIII	9341500	9343000	0;1;2;	1;4;61;	6;1;4	0;0;0	0;0;0	3;1;1	0;0;0	3;0;3	0;0;0	0;0;0	0;0;0	0;0;0	0;0;0	0;0;0
groupVIII	9656000	9659500	1;2;	3;63;	3;0;3	0;0;0	0;0;0	0;0;0	0;0;0	0;0;0	3;0;3	0;0;0	0;0;0	0;0;0	0;0;0	0;0;0
groupVIII	9753500	9754500	0;1;2;	1;2;63;	4;1;2	0;0;0	0;0;0	0;0;0	0;0;0	0;0;0	0;0;0	0;0;0	2;0;2	2;1;0	0;0;0	0;0;0
groupVIII	10186000	10201000	2;3;	65;1;	1;0;1	0;0;0	0;0;0	0;0;0	0;0;0	0;0;0	0;0;0	0;0;0	0;0;0	1;0;1	0;0;0	0;0;0
groupVIII	10216500	10220500	1;2;	4;62;	4;0;4	0;0;0	0;0;0	2;0;2	0;0;0	2;0;2	0;0;0	0;0;0	0;0;0	0;0;0	0;0;0	0;0;0
groupVIII	10509500	10511000	1;2;	2;64;	2;0;2	0;0;0	0;0;0	1;0;1	0;0;0	0;0;0	0;0;0	0;0;0	0;0;0	0;0;0	1;0;1	0;0;0
groupVIII	10520000	10522500	1;2;	6;60;	6;0;6	0;0;0	0;0;0	1;0;1	0;0;0	0;0;0	0;0;0	0;0;0	0;0;0	0;0;0	5;0;5	0;0;0
groupVIII	11314500	11317000	1;2;	1;65;	1;0;1	0;0;0	0;0;0	1;0;1	0;0;0	0;0;0	0;0;0	0;0;0	0;0;0	0;0;0	0;0;0	0;0;0
groupVIII	11586000	11591000	2;3;	65;1;	1;0;1	0;0;0	0;0;0	0;0;0	1;0;1	0;0;0	0;0;0	0;0;0	0;0;0	0;0;0	0;0;0	0;0;0
groupVIII	12326000	12330500	2;3;4;	62;3;1;	5;1;3	0;0;0	0;0;0	4;1;2	0;0;0	1;0;1	0;0;0	0;0;0	0;0;0	0;0;0	0;0;0	0;0;0
groupVIII	12501500	12515000	1;2;	6;60;	6;0;6	0;0;0	0;0;0	0;0;0	0;0;0	0;0;0	0;0;0	0;0;0	1;0;1	5;0;5	0;0;0	0;0;0
groupVIII	12752000	12763500	2;3;	65;1;	1;0;1	1;0;1	0;0;0	0;0;0	0;0;0	0;0;0	0;0;0	0;0;0	0;0;0	0;0;0	0;0;0	0;0;0
groupVIII	12923500	12931000	2;3;	65;1;	1;0;1	0;0;0	0;0;0	0;0;0	1;0;1	0;0;0	0;0;0	0;0;0	0;0;0	0;0;0	0;0;0	0;0;0
groupVIII	13014500	13033000	2;3;	64;2;	2;0;2	0;0;0	2;0;2	0;0;0	0;0;0	0;0;0	0;0;0	0;0;0	0;0;0	0;0;0	0;0;0	0;0;0
groupVIII	14338000	14347000	1;2;	8;58;	8;0;8	3;0;3	0;0;0	1;0;1	0;0;0	2;0;2	0;0;0	1;0;1	1;0;1	0;0;0	0;0;0	0;0;0
groupVIII	14617000	14622500	1;2;	3;63;	3;0;3	0;0;0	0;0;0	0;0;0	0;0;0	0;0;0	0;0;0	0;0;0	0;0;0	0;0;0	0;0;0	3;0;3
groupVIII	14882000	14884000	1;2;	3;63;	3;0;3	0;0;0	0;0;0	0;0;0	0;0;0	0;0;0	0;0;0	0;0;0	3;0;3	0;0;0	0;0;0	0;0;0
groupVIII	14939000	14942000	1;2;	1;65;	1;0;1	0;0;0	0;0;0	0;0;0	0;0;0	1;0;1	0;0;0	0;0;0	0;0;0	0;0;0	0;0;0	0;0;0
groupVIII	15159500	15161500	0;1;2;	1;3;62;	5;1;3	4;1;2	0;0;0	0;0;0	1;0;1	0;0;0	0;0;0	0;0;0	0;0;0	0;0;0	0;0;0	0;0;0

groupVIII	16064500	16072500	1;2;	1;65;	1;0;1	1;0;1	0;0;0	0;0;0	0;0;0	0;0;0	0;0;0	0;0;0	0;0;0	0;0;0	0;0;0	0;0;0
groupVIII	16408000	16410000	2;3;	62;4;	4;0;4	1;0;1	0;0;0	1;0;1	0;0;0	1;0;1	0;0;0	1;0;1	0;0;0	0;0;0	0;0;0	0;0;0
groupVIII	17033500	17043500	2;3;	60;6;	6;0;6	1;0;1	0;0;0	0;0;0	0;0;0	1;0;1	0;0;0	0;0;0	1;0;1	2;0;2	1;0;1	0;0;0
groupVIII	17156500	17163000	1;2;	1;65;	1;0;1	0;0;0	0;0;0	0;0;0	0;0;0	0;0;0	0;0;0	0;0;0	0;0;0	1;0;1	0;0;0	0;0;0
groupVIII	17426500	17434000	2;3;4;	63;2;1;	4;1;2	0;0;0	0;0;0	0;0;0	0;0;0	0;0;0	0;0;0	0;0;0	1;0;1	0;0;0	1;0;1	2;1;0
groupVIII	17707500	17716000	2;3;	65;1;	1;0;1	0;0;0	1;0;1	0;0;0	0;0;0	0;0;0	0;0;0	0;0;0	0;0;0	0;0;0	0;0;0	0;0;0
groupVIII	18078000	18080000	2;3;	62;4;	4;0;4	2;0;2	0;0;0	0;0;0	1;0;1	0;0;0	0;0;0	1;0;1	0;0;0	0;0;0	0;0;0	0;0;0
groupVIII	18264500	18269500	2;3;	65;1;	1;0;1	0;0;0	0;0;0	0;0;0	0;0;0	0;0;0	1;0;1	0;0;0	0;0;0	0;0;0	0;0;0	0;0;0
groupVIII	18533000	18537500	2;3;	63;3;	3;0;3	0;0;0	3;0;3	0;0;0	0;0;0	0;0;0	0;0;0	0;0;0	0;0;0	0;0;0	0;0;0	0;0;0
groupVIII	18703500	18718500	2;3;4;	44;19;3;	25;3;19	0;0;0	0;0;0	0;0;0	0;0;0	0;0;0	0;0;0	0;0;0	7;1;5	7;1;5	6;1;4	5;0;5
groupVIII	18843500	18861000	2;3;	64;2;	2;0;2	0;0;0	0;0;0	0;0;0	2;0;2	0;0;0	0;0;0	0;0;0	0;0;0	0;0;0	0;0;0	0;0;0
groupVIII	18976000	18996500	2;3;	64;2;	2;0;2	0;0;0	0;0;0	0;0;0	0;0;0	0;0;0	0;0;0	0;0;0	0;0;0	0;0;0	0;0;0	2;0;2
groupIX	1484000	1487000	1;2;	3;63;	3;0;3	0;0;0	0;0;0	0;0;0	0;0;0	0;0;0	0;0;0	0;0;0	1;0;1	0;0;0	0;0;0	2;0;2
groupIX	1908500	1913000	1;2;	1;65;	1;0;1	0;0;0	0;0;0	0;0;0	0;0;0	0;0;0	0;0;0	1;0;1	0;0;0	0;0;0	0;0;0	0;0;0
groupIX	1936500	1940000	1;2;	4;62;	4;0;4	0;0;0	0;0;0	0;0;0	0;0;0	0;0;0	0;0;0	0;0;0	1;0;1	0;0;0	1;0;1	2;0;2
groupIX	1961500	1966000	2;3;	65;1;	1;0;1	1;0;1	0;0;0	0;0;0	0;0;0	0;0;0	0;0;0	0;0;0	0;0;0	0;0;0	0;0;0	0;0;0
groupIX	2322500	2339500	2;3;	63;3;	3;0;3	0;0;0	0;0;0	0;0;0	0;0;0	0;0;0	0;0;0	0;0;0	0;0;0	0;0;0	0;0;0	3;0;3
groupIX	2594000	2608000	1;2;	1;65;	1;0;1	0;0;0	0;0;0	0;0;0	1;0;1	0;0;0	0;0;0	0;0;0	0;0;0	0;0;0	0;0;0	0;0;0
groupIX	2846000	2848000	1;2;	1;65;	1;0;1	0;0;0	0;0;0	0;0;0	0;0;0	1;0;1	0;0;0	0;0;0	0;0;0	0;0;0	0;0;0	0;0;0
groupIX	3009000	3010500	1;2;	4;62;	4;0;4	0;0;0	0;0;0	0;0;0	3;0;3	1;0;1	0;0;0	0;0;0	0;0;0	0;0;0	0;0;0	0;0;0
groupIX	3064500	3069500	0;1;2;	1;11;54;	13;1;11	0;0;0	0;0;0	0;0;0	0;0;0	0;0;0	0;0;0	0;0;0	5;0;5	5;1;3	3;0;3	0;0;0
groupIX	3189500	3200500	1;2;	1;65;	1;0;1	0;0;0	0;0;0	1;0;1	0;0;0	0;0;0	0;0;0	0;0;0	0;0;0	0;0;0	0;0;0	0;0;0
groupIX	3424500	3430000	1;2;	1;65;	1;0;1	0;0;0	0;0;0	1;0;1	0;0;0	0;0;0	0;0;0	0;0;0	0;0;0	0;0;0	0;0;0	0;0;0
groupIX	3439500	3445500	1;2;	4;62;	4;0;4	1;0;1	1;0;1	0;0;0	0;0;0	1;0;1	0;0;0	0;0;0	1;0;1	0;0;0	0;0;0	0;0;0
groupIX	3546500	3551500	1;2;	6;60;	6;0;6	0;0;0	0;0;0	0;0;0	2;0;2	0;0;0	0;0;0	0;0;0	1;0;1	3;0;3	0;0;0	0;0;0
groupIX	3595500	3596500	0;1;2;	1;2;63;	4;1;2	0;0;0	0;0;0	0;0;0	0;0;0	0;0;0	0;0;0	4;1;2	0;0;0	0;0;0	0;0;0	0;0;0
groupIX	3720500	3729000	2;3;	65;1;	1;0;1	1;0;1	0;0;0	0;0;0	0;0;0	0;0;0	0;0;0	0;0;0	0;0;0	0;0;0	0;0;0	0;0;0
groupIX	3781000	3782500	0;2;	1;65;	2;1;0	0;0;0	0;0;0	0;0;0	0;0;0	0;0;0	0;0;0	2;1;0	0;0;0	0;0;0	0;0;0	0;0;0
groupIX	5449000	5452500	1;2;	1;65;	1;0;1	0;0;0	0;0;0	0;0;0	0;0;0	1;0;1	0;0;0	0;0;0	0;0;0	0;0;0	0;0;0	0;0;0
groupIX	5540500	5546500	2;3;4;	64;1;1;	3;1;1	0;0;0	0;0;0	0;0;0	0;0;0	0;0;0	3;1;1	0;0;0	0;0;0	0;0;0	0;0;0	0;0;0
groupIX	5741000	5744000	1;2;	2;64;	2;0;2	0;0;0	0;0;0	0;0;0	0;0;0	0;0;0	0;0;0	0;0;0	1;0;1	1;0;1	0;0;0	0;0;0
groupIX	5769500	5772000	1;2;	8;58;	8;0;8	0;0;0	0;0;0	0;0;0	0;0;0	0;0;0	4;0;4	3;0;3	1;0;1	0;0;0	0;0;0	0;0;0
groupIX	5912500	5918000	2;3;	65;1;	1;0;1	0;0;0	0;0;0	0;0;0	0;0;0	0;0;0	0;0;0	0;0;0	1;0;1	0;0;0	0;0;0	0;0;0
groupIX	7211500	7215000	0;1;2;	1;3;62;	5;1;3	0;0;0	0;0;0	0;0;0	5;1;3	0;0;0	0;0;0	0;0;0	0;0;0	0;0;0	0;0;0	0;0;0

groupIX	7909500	7925000	2;3;	65;1;	1;0;1	0;0;0	0;0;0	0;0;0	0;0;0	0;0;0	0;0;0	0;0;0	1;0;1	0;0;0	0;0;0	0;0;0
groupIX	7932000	7933000	1;2;	4;62;	4;0;4	0;0;0	0;0;0	0;0;0	0;0;0	0;0;0	3;0;3	1;0;1	0;0;0	0;0;0	0;0;0	0;0;0
groupIX	7997500	7999500	1;2;	4;62;	4;0;4	0;0;0	2;0;2	0;0;0	0;0;0	0;0;0	0;0;0	0;0;0	0;0;0	2;0;2	0;0;0	0;0;0
groupIX	8321000	8325000	1;2;	1;65;	1;0;1	0;0;0	0;0;0	1;0;1	0;0;0	0;0;0	0;0;0	0;0;0	0;0;0	0;0;0	0;0;0	0;0;0
groupIX	8832000	8846000	2;3;	55;11;	11;0;11	0;0;0	0;0;0	0;0;0	0;0;0	0;0;0	0;0;0	0;0;0	0;0;0	1;0;1	5;0;5	5;0;5
groupIX	9041000	9046500	2;3;	65;1;	1;0;1	0;0;0	0;0;0	0;0;0	0;0;0	1;0;1	0;0;0	0;0;0	0;0;0	0;0;0	0;0;0	0;0;0
groupIX	9102500	9106000	1;2;	1;65;	1;0;1	0;0;0	0;0;0	1;0;1	0;0;0	0;0;0	0;0;0	0;0;0	0;0;0	0;0;0	0;0;0	0;0;0
groupIX	9106500	9121500	2;3;	62;4;	4;0;4	0;0;0	0;0;0	0;0;0	0;0;0	0;0;0	0;0;0	0;0;0	1;0;1	1;0;1	2;0;2	0;0;0
groupIX	9429000	9434000	2;3;4;	56;8;2;	12;2;8	0;0;0	0;0;0	0;0;0	0;0;0	0;0;0	0;0;0	0;0;0	0;0;0	3;0;3	4;1;2	5;1;3
groupIX	9473000	9483500	1;2;	13;53;	13;0;13	0;0;0	0;0;0	0;0;0	0;0;0	0;0;0	0;0;0	0;0;0	0;0;0	3;0;3	5;0;5	5;0;5
groupIX	9496500	9498500	1;2;	1;65;	1;0;1	0;0;0	0;0;0	0;0;0	0;0;0	0;0;0	0;0;0	0;0;0	0;0;0	1;0;1	0;0;0	0;0;0
groupIX	9548000	9556500	2;3;	65;1;	1;0;1	0;0;0	0;0;0	0;0;0	0;0;0	0;0;0	0;0;0	0;0;0	1;0;1	0;0;0	0;0;0	0;0;0
groupIX	9725500	9757000	2;3;4;	57;8;1;	10;1;8	0;0;0	6;0;6	0;0;0	4;1;2	0;0;0	0;0;0	0;0;0	0;0;0	0;0;0	0;0;0	0;0;0
groupIX	10194000	10202000	2;3;4;	60;4;2;	8;2;4	0;0;0	7;2;3	0;0;0	1;0;1	0;0;0	0;0;0	0;0;0	0;0;0	0;0;0	0;0;0	0;0;0
groupIX	10396000	10398000	0;1;2;	1;7;58;	9;1;7	2;1;0	0;0;0	0;0;0	0;0;0	1;0;1	0;0;0	0;0;0	1;0;1	0;0;0	3;0;3	2;0;2
groupIX	10460500	10471500	1;2;	5;61;	5;0;5	0;0;0	0;0;0	0;0;0	0;0;0	0;0;0	0;0;0	0;0;0	0;0;0	0;0;0	5;0;5	0;0;0
groupIX	10471500	10477500	2;3;	65;1;	1;0;1	0;0;0	0;0;0	0;0;0	1;0;1	0;0;0	0;0;0	0;0;0	0;0;0	0;0;0	0;0;0	0;0;0
groupIX	10518000	10519000	0;1;2;	1;4;61;	6;1;4	0;0;0	0;0;0	0;0;0	0;0;0	0;0;0	0;0;0	5;1;3	1;0;1	0;0;0	0;0;0	0;0;0
groupIX	10674000	10680000	2;3;	64;2;	2;0;2	2;0;2	0;0;0	0;0;0	0;0;0	0;0;0	0;0;0	0;0;0	0;0;0	0;0;0	0;0;0	0;0;0
groupIX	11344000	11345500	0;1;2;	2;8;56;	12;2;8	0;0;0	7;2;3	0;0;0	4;0;4	1;0;1	0;0;0	0;0;0	0;0;0	0;0;0	0;0;0	0;0;0
groupIX	11419000	11421000	1;2;	1;65;	1;0;1	1;0;1	0;0;0	0;0;0	0;0;0	0;0;0	0;0;0	0;0;0	0;0;0	0;0;0	0;0;0	0;0;0
groupIX	11733500	11735500	0;1;2;	3;10;53;	16;3;10	0;0;0	7;2;3	0;0;0	3;1;1	0;0;0	0;0;0	0;0;0	0;0;0	0;0;0	4;0;4	2;0;2
groupIX	12081000	12084000	0;1;2;	4;7;55;	15;4;7	0;0;0	0;0;0	0;0;0	0;0;0	0;0;0	0;0;0	0;0;0	0;0;0	0;0;0	8;2;4	7;2;3
groupIX	12554000	12557500	1;2;	2;64;	2;0;2	0;0;0	0;0;0	0;0;0	1;0;1	1;0;1	0;0;0	0;0;0	0;0;0	0;0;0	0;0;0	0;0;0
groupIX	12703500	12727000	2;3;	61;5;	5;0;5	0;0;0	1;0;1	0;0;0	0;0;0	0;0;0	2;0;2	2;0;2	0;0;0	0;0;0	0;0;0	0;0;0
groupIX	12787000	12790500	1;2;	1;65;	1;0;1	1;0;1	0;0;0	0;0;0	0;0;0	0;0;0	0;0;0	0;0;0	0;0;0	0;0;0	0;0;0	0;0;0
groupIX	12893000	12894500	1;2;	1;65;	1;0;1	1;0;1	0;0;0	0;0;0	0;0;0	0;0;0	0;0;0	0;0;0	0;0;0	0;0;0	0;0;0	0;0;0
groupIX	13025000	13033000	2;4;	65;1;	2;1;0	0;0;0	0;0;0	0;0;0	0;0;0	2;1;0	0;0;0	0;0;0	0;0;0	0;0;0	0;0;0	0;0;0
groupIX	13077500	13295000	2;3;	64;2;	2;0;2	0;0;0	0;0;0	0;0;0	0;0;0	0;0;0	0;0;0	0;0;0	0;0;0	0;0;0	2;0;2	0;0;0
groupIX	13750500	13763000	2;3;	57;9;	9;0;9	1;0;1	0;0;0	2;0;2	0;0;0	3;0;3	0;0;0	0;0;0	0;0;0	0;0;0	3;0;3	0;0;0
groupIX	13857000	13862000	1;2;	1;65;	1;0;1	1;0;1	0;0;0	0;0;0	0;0;0	0;0;0	0;0;0	0;0;0	0;0;0	0;0;0	0;0;0	0;0;0
groupIX	13870000	13873500	1;2;	1;65;	1;0;1	1;0;1	0;0;0	0;0;0	0;0;0	0;0;0	0;0;0	0;0;0	0;0;0	0;0;0	0;0;0	0;0;0
groupIX	13881000	13884500	1;2;	2;64;	2;0;2	2;0;2	0;0;0	0;0;0	0;0;0	0;0;0	0;0;0	0;0;0	0;0;0	0;0;0	0;0;0	0;0;0
groupIX	14095000	14098000	1;2;	3;63;	3;0;3	0;0;0	0;0;0	3;0;3	0;0;0	0;0;0	0;0;0	0;0;0	0;0;0	0;0;0	0;0;0	0;0;0

groupIX	14187500	14286000	2;3;4;	48;13;5;	23;5;13	6;1;4	1;0;1	0;0;0	0;0;0	2;0;2	5;1;3	1;0;1	5;2;1	3;1;1	0;0;0	0;0;0
groupIX	14316500	14341000	2;3;	61;5;	5;0;5	0;0;0	0;0;0	0;0;0	0;0;0	0;0;0	0;0;0	0;0;0	3;0;3	2;0;2	0;0;0	0;0;0
groupIX	14879500	14887500	2;3;4;	61;4;1;	6;1;4	0;0;0	0;0;0	0;0;0	6;1;4	0;0;0	0;0;0	0;0;0	0;0;0	0;0;0	0;0;0	0;0;0
groupIX	15144000	15158500	1;2;	6;60;	6;0;6	1;0;1	0;0;0	0;0;0	0;0;0	0;0;0	2;0;2	1;0;1	0;0;0	2;0;2	0;0;0	0;0;0
groupIX	15313000	15328000	2;3;4;	60;5;1;	7;1;5	5;1;3	0;0;0	0;0;0	0;0;0	2;0;2	0;0;0	0;0;0	0;0;0	0;0;0	0;0;0	0;0;0
groupIX	15820000	15823000	0;1;2;	1;3;62;	5;1;3	0;0;0	0;0;0	0;0;0	0;0;0	0;0;0	0;0;0	0;0;0	2;0;2	3;1;1	0;0;0	0;0;0
groupIX	16215000	16217500	0;1;2;	2;2;62;	6;2;2	0;0;0	0;0;0	0;0;0	0;0;0	0;0;0	5;2;1	1;0;1	0;0;0	0;0;0	0;0;0	0;0;0
groupIX	16423500	16442500	2;3;	64;2;	2;0;2	0;0;0	0;0;0	0;0;0	0;0;0	0;0;0	0;0;0	0;0;0	1;0;1	1;0;1	0;0;0	0;0;0
groupIX	16609000	16613500	1;2;	8;58;	8;0;8	2;0;2	0;0;0	3;0;3	2;0;2	1;0;1	0;0;0	0;0;0	0;0;0	0;0;0	0;0;0	0;0;0
groupIX	16625500	16626500	0;1;2;	5;5;56;	15;5;5	0;0;0	0;0;0	0;0;0	0;0;0	0;0;0	6;2;2	9;3;3	0;0;0	0;0;0	0;0;0	0;0;0
groupIX	16734000	16792000	2;3;4;	62;2;2;	6;2;2	0;0;0	0;0;0	0;0;0	0;0;0	0;0;0	6;2;2	0;0;0	0;0;0	0;0;0	0;0;0	0;0;0
groupIX	16986500	16989500	1;2;	13;53;	13;0;13	2;0;2	1;0;1	5;0;5	3;0;3	2;0;2	0;0;0	0;0;0	0;0;0	0;0;0	0;0;0	0;0;0
groupIX	17040500	17042000	0;1;2;	1;12;53;	14;1;12	0;0;0	0;0;0	0;0;0	0;0;0	0;0;0	0;0;0	0;0;0	2;0;2	4;0;4	2;0;2	6;1;4
groupIX	17101000	17122500	1;2;	9;57;	9;0;9	0;0;0	0;0;0	4;0;4	0;0;0	0;0;0	0;0;0	0;0;0	2;0;2	3;0;3	0;0;0	0;0;0
groupIX	17143000	17147500	2;3;	65;1;	1;0;1	0;0;0	0;0;0	1;0;1	0;0;0	0;0;0	0;0;0	0;0;0	0;0;0	0;0;0	0;0;0	0;0;0
groupIX	17263500	17265500	2;3;	64;2;	2;0;2	0;0;0	0;0;0	0;0;0	2;0;2	0;0;0	0;0;0	0;0;0	0;0;0	0;0;0	0;0;0	0;0;0
groupIX	17624500	17630500	2;3;	65;1;	1;0;1	0;0;0	0;0;0	0;0;0	0;0;0	1;0;1	0;0;0	0;0;0	0;0;0	0;0;0	0;0;0	0;0;0
groupIX	17815500	17817500	0;1;2;	1;3;62;	5;1;3	0;0;0	0;0;0	0;0;0	0;0;0	0;0;0	0;0;0	0;0;0	2;0;2	3;1;1	0;0;0	0;0;0
groupIX	18152500	18153500	0;1;2;	2;5;59;	9;2;5	0;0;0	7;2;3	0;0;0	1;0;1	0;0;0	0;0;0	0;0;0	0;0;0	1;0;1	0;0;0	0;0;0
groupIX	18353500	18371500	1;2;	2;64;	2;0;2	0;0;0	0;0;0	0;0;0	0;0;0	1;0;1	0;0;0	1;0;1	0;0;0	0;0;0	0;0;0	0;0;0
groupIX	18477000	18478500	1;2;	4;62;	4;0;4	0;0;0	0;0;0	1;0;1	0;0;0	2;0;2	0;0;0	0;0;0	0;0;0	0;0;0	1;0;1	0;0;0
groupIX	18714500	18731500	2;3;	64;2;	2;0;2	0;0;0	0;0;0	0;0;0	0;0;0	1;0;1	0;0;0	0;0;0	0;0;0	1;0;1	0;0;0	0;0;0
groupIX	19089000	19213500	2;3;4;	43;20;3;	26;3;20	0;0;0	0;0;0	0;0;0	0;0;0	1;0;1	0;0;0	0;0;0	4;0;4	6;0;6	6;0;6	9;3;3
groupIX	19483000	19487500	1;2;	2;64;	2;0;2	0;0;0	0;0;0	0;0;0	0;0;0	0;0;0	0;0;0	0;0;0	1;0;1	0;0;0	1;0;1	0;0;0
groupIX	19510500	19517000	1;2;	1;65;	1;0;1	0;0;0	0;0;0	0;0;0	0;0;0	0;0;0	0;0;0	0;0;0	0;0;0	1;0;1	0;0;0	0;0;0
groupIX	19840500	19854500	1;2;	1;65;	1;0;1	0;0;0	0;0;0	0;0;0	0;0;0	0;0;0	0;0;0	0;0;0	0;0;0	0;0;0	1;0;1	0;0;0
groupX	293000	295500	1;2;	6;60;	6;0;6	2;0;2	1;0;1	1;0;1	2;0;2	0;0;0	0;0;0	0;0;0	0;0;0	0;0;0	0;0;0	0;0;0
groupX	308000	309500	1;2;	2;64;	2;0;2	0;0;0	0;0;0	2;0;2	0;0;0	0;0;0	0;0;0	0;0;0	0;0;0	0;0;0	0;0;0	0;0;0
groupX	1251000	1258000	2;3;	65;1;	1;0;1	1;0;1	0;0;0	0;0;0	0;0;0	0;0;0	0;0;0	0;0;0	0;0;0	0;0;0	0;0;0	0;0;0
groupX	1615000	1623000	2;3;	65;1;	1;0;1	0;0;0	0;0;0	0;0;0	0;0;0	0;0;0	0;0;0	0;0;0	1;0;1	0;0;0	0;0;0	0;0;0
groupX	1735000	1746500	2;3;	60;6;	6;0;6	1;0;1	0;0;0	0;0;0	0;0;0	0;0;0	0;0;0	0;0;0	1;0;1	4;0;4	0;0;0	0;0;0
groupX	1987500	1989500	0;1;2;	1;1;64;	3;1;1	0;0;0	0;0;0	3;1;1	0;0;0	0;0;0	0;0;0	0;0;0	0;0;0	0;0;0	0;0;0	0;0;0
groupX	2139500	2143500	2;3;	65;1;	1;0;1	0;0;0	0;0;0	0;0;0	0;0;0	0;0;0	1;0;1	0;0;0	0;0;0	0;0;0	0;0;0	0;0;0
groupX	2315000	2320500	2;3;	65;1;	1;0;1	0;0;0	0;0;0	0;0;0	0;0;0	1;0;1	0;0;0	0;0;0	0;0;0	0;0;0	0;0;0	0;0;0

groupX	2357500	2366500	2;3;	65;1;	1;0;1	0;0;0	0;0;0	0;0;0	0;0;0	0;0;0	0;0;0	0;0;0	0;0;0	0;0;0	0;0;0	1;0;1
groupX	2742500	2744500	1;2;	4;62;	4;0;4	0;0;0	0;0;0	0;0;0	0;0;0	0;0;0	0;0;0	1;0;1	2;0;2	1;0;1	0;0;0	0;0;0
groupX	2823500	2826500	1;2;	1;65;	1;0;1	1;0;1	0;0;0	0;0;0	0;0;0	0;0;0	0;0;0	0;0;0	0;0;0	0;0;0	0;0;0	0;0;0
groupX	3018000	3019500	1;2;	1;65;	1;0;1	0;0;0	1;0;1	0;0;0	0;0;0	0;0;0	0;0;0	0;0;0	0;0;0	0;0;0	0;0;0	0;0;0
groupX	4299500	4301500	0;1;2;	3;4;59;	10;3;4	0;0;0	0;0;0	0;0;0	0;0;0	0;0;0	0;0;0	0;0;0	8;3;2	2;0;2	0;0;0	0;0;0
groupX	5217000	5219500	0;2;	1;65;	2;1;0	0;0;0	0;0;0	0;0;0	2;1;0	0;0;0	0;0;0	0;0;0	0;0;0	0;0;0	0;0;0	0;0;0
groupX	5893500	5894500	0;1;2;	2;5;59;	9;2;5	0;0;0	0;0;0	0;0;0	0;0;0	0;0;0	0;0;0	0;0;0	0;0;0	0;0;0	6;2;2	3;0;3
groupX	7850000	7858000	2;3;	65;1;	1;0;1	0;0;0	0;0;0	0;0;0	1;0;1	0;0;0	0;0;0	0;0;0	0;0;0	0;0;0	0;0;0	0;0;0
groupX	8843500	8852000	1;2;	6;60;	6;0;6	0;0;0	0;0;0	0;0;0	0;0;0	0;0;0	0;0;0	0;0;0	0;0;0	0;0;0	3;0;3	3;0;3
groupX	9193500	9194500	2;3;	60;6;	6;0;6	0;0;0	1;0;1	0;0;0	2;0;2	0;0;0	0;0;0	0;0;0	0;0;0	3;0;3	0;0;0	0;0;0
groupX	9532500	9533500	0;1;2;	2;11;53;	15;2;11	1;0;1	7;2;3	0;0;0	4;0;4	2;0;2	0;0;0	1;0;1	0;0;0	0;0;0	0;0;0	0;0;0
groupX	9592500	9600500	2;3;	65;1;	1;0;1	0;0;0	0;0;0	0;0;0	0;0;0	0;0;0	0;0;0	1;0;1	0;0;0	0;0;0	0;0;0	0;0;0
groupX	10180500	10183500	1;2;	1;65;	1;0;1	0;0;0	0;0;0	0;0;0	0;0;0	0;0;0	0;0;0	0;0;0	0;0;0	1;0;1	0;0;0	0;0;0
groupX	10905500	10911500	2;3;	65;1;	1;0;1	0;0;0	0;0;0	0;0;0	0;0;0	0;0;0	0;0;0	0;0;0	0;0;0	1;0;1	0;0;0	0;0;0
groupX	11202500	11209500	2;3;	65;1;	1;0;1	0;0;0	0;0;0	0;0;0	1;0;1	0;0;0	0;0;0	0;0;0	0;0;0	0;0;0	0;0;0	0;0;0
groupX	11272500	11278000	1;2;	13;53;	13;0;13	0;0;0	0;0;0	0;0;0	0;0;0	0;0;0	0;0;0	0;0;0	5;0;5	6;0;6	1;0;1	1;0;1
groupX	11282000	11283500	1;2;	6;60;	6;0;6	0;0;0	0;0;0	2;0;2	0;0;0	4;0;4	0;0;0	0;0;0	0;0;0	0;0;0	0;0;0	0;0;0
groupX	11394000	11419500	2;3;	65;1;	1;0;1	0;0;0	0;0;0	0;0;0	0;0;0	0;0;0	0;0;0	1;0;1	0;0;0	0;0;0	0;0;0	0;0;0
groupX	12259000	12261500	1;2;	11;55;	11;0;11	2;0;2	1;0;1	3;0;3	0;0;0	2;0;2	0;0;0	3;0;3	0;0;0	0;0;0	0;0;0	0;0;0
groupX	12507000	12513000	1;2;	4;62;	4;0;4	0;0;0	0;0;0	4;0;4	0;0;0	0;0;0	0;0;0	0;0;0	0;0;0	0;0;0	0;0;0	0;0;0
groupX	12930500	12933500	1;2;	2;64;	2;0;2	0;0;0	0;0;0	0;0;0	1;0;1	0;0;0	0;0;0	0;0;0	1;0;1	0;0;0	0;0;0	0;0;0
groupX	13143500	13146500	1;2;	3;63;	3;0;3	0;0;0	0;0;0	1;0;1	0;0;0	2;0;2	0;0;0	0;0;0	0;0;0	0;0;0	0;0;0	0;0;0
groupX	13337500	13340500	0;1;2;	1;2;63;	4;1;2	4;1;2	0;0;0	0;0;0	0;0;0	0;0;0	0;0;0	0;0;0	0;0;0	0;0;0	0;0;0	0;0;0
groupX	13570000	13572000	1;2;	2;64;	2;0;2	0;0;0	0;0;0	0;0;0	0;0;0	0;0;0	0;0;0	2;0;2	0;0;0	0;0;0	0;0;0	0;0;0
groupX	13696500	13707000	1;2;	1;65;	1;0;1	0;0;0	0;0;0	0;0;0	0;0;0	0;0;0	0;0;0	0;0;0	0;0;0	0;0;0	0;0;0	1;0;1
groupX	13759500	13766000	2;3;	65;1;	1;0;1	0;0;0	0;0;0	0;0;0	1;0;1	0;0;0	0;0;0	0;0;0	0;0;0	0;0;0	0;0;0	0;0;0
groupX	13779000	13782000	1;2;	2;64;	2;0;2	0;0;0	2;0;2	0;0;0	0;0;0	0;0;0	0;0;0	0;0;0	0;0;0	0;0;0	0;0;0	0;0;0
groupX	14195000	14199500	1;2;	1;65;	1;0;1	0;0;0	0;0;0	0;0;0	0;0;0	0;0;0	0;0;0	0;0;0	0;0;0	0;0;0	1;0;1	0;0;0
groupX	14427000	14430500	0;1;2;	12;7;47;	31;12;7	0;0;0	0;0;0	0;0;0	0;0;0	0;0;0	0;0;0	0;0;0	4;0;4	3;0;3	12;6;0	12;6;0
groupX	14977500	14989500	2;3;4;	45;16;5;	26;5;16	1;0;1	0;0;0	6;1;4	9;3;3	3;0;3	0;0;0	1;0;1	2;0;2	1;0;1	0;0;0	3;1;1
groupX	15127000	15128500	1;2;	16;50;	16;0;16	1;0;1	5;0;5	2;0;2	4;0;4	1;0;1	0;0;0	3;0;3	0;0;0	0;0;0	0;0;0	0;0;0
groupXI	64000	82000	2;3;	65;1;	1;0;1	0;0;0	0;0;0	1;0;1	0;0;0	0;0;0	0;0;0	0;0;0	0;0;0	0;0;0	0;0;0	0;0;0
groupXI	325000	327500	1;2;	6;60;	6;0;6	0;0;0	0;0;0	0;0;0	0;0;0	0;0;0	0;0;0	0;0;0	3;0;3	2;0;2	0;0;0	1;0;1
groupXI	797000	811500	2;3;4;	60;5;1;	7;1;5	7;1;5	0;0;0	0;0;0	0;0;0	0;0;0	0;0;0	0;0;0	0;0;0	0;0;0	0;0;0	0;0;0

groupXI	955000	961000	0;1;2;	5;5;56;	15;5;5	0;0;0	0;0;0	0;0;0	0;0;0	0;0;0	0;0;0	0;0;0	9;4;1	6;1;4	0;0;0	0;0;0
groupXI	1055500	1058000	1;2;	2;64;	2;0;2	0;0;0	0;0;0	0;0;0	0;0;0	0;0;0	0;0;0	0;0;0	1;0;1	1;0;1	0;0;0	0;0;0
groupXI	1065500	1069000	0;1;2;	2;8;56;	12;2;8	0;0;0	2;0;2	0;0;0	0;0;0	0;0;0	8;2;4	2;0;2	0;0;0	0;0;0	0;0;0	0;0;0
groupXI	1573000	1576000	0;1;2;	1;2;63;	4;1;2	0;0;0	0;0;0	0;0;0	0;0;0	0;0;0	0;0;0	0;0;0	1;0;1	3;1;1	0;0;0	0;0;0
groupXI	2035500	2039000	0;1;2;	2;3;61;	7;2;3	0;0;0	0;0;0	0;0;0	0;0;0	0;0;0	0;0;0	0;0;0	2;0;2	3;1;1	0;0;0	2;1;0
groupXI	2240000	2245000	1;2;	2;64;	2;0;2	0;0;0	0;0;0	0;0;0	0;0;0	0;0;0	0;0;0	0;0;0	1;0;1	1;0;1	0;0;0	0;0;0
groupXI	2847000	2861500	2;3;	65;1;	1;0;1	0;0;0	0;0;0	0;0;0	1;0;1	0;0;0	0;0;0	0;0;0	0;0;0	0;0;0	0;0;0	0;0;0
groupXI	2865500	2867000	0;2;	1;65;	2;1;0	0;0;0	0;0;0	0;0;0	0;0;0	0;0;0	0;0;0	0;0;0	0;0;0	0;0;0	2;1;0	0;0;0
groupXI	3247000	3254000	2;3;	65;1;	1;0;1	0;0;0	0;0;0	0;0;0	0;0;0	0;0;0	0;0;0	0;0;0	0;0;0	0;0;0	0;0;0	1;0;1
groupXI	3594500	3597000	1;2;	9;57;	9;0;9	0;0;0	0;0;0	0;0;0	0;0;0	0;0;0	0;0;0	0;0;0	4;0;4	4;0;4	1;0;1	0;0;0
groupXI	5016000	5019500	2;3;4;	39;19;8;	35;8;19	1;0;1	8;2;4	8;3;2	6;2;2	6;0;6	0;0;0	0;0;0	6;1;4	0;0;0	0;0;0	0;0;0
groupXI	5856500	5860500	2;3;	53;13;	13;0;13	0;0;0	0;0;0	0;0;0	0;0;0	0;0;0	0;0;0	0;0;0	4;0;4	2;0;2	3;0;3	4;0;4
groupXI	6425000	6426000	0;1;2;	11;6;49;	28;11;6	0;0;0	0;0;0	0;0;0	0;0;0	0;0;0	0;0;0	0;0;0	2;0;2	5;2;1	9;3;3	12;6;0
groupXI	6431000	6446000	1;2;	17;49;	17;0;17	0;0;0	0;0;0	0;0;0	0;0;0	0;0;0	0;0;0	0;0;0	2;0;2	3;0;3	6;0;6	6;0;6
groupXI	6450500	6511500	1;2;	22;44;	22;0;22	0;0;0	0;0;0	0;0;0	0;0;0	0;0;0	0;0;0	0;0;0	6;0;6	4;0;4	6;0;6	6;0;6
groupXI	6579500	6581000	1;2;	4;62;	4;0;4	0;0;0	2;0;2	0;0;0	2;0;2	0;0;0	0;0;0	0;0;0	0;0;0	0;0;0	0;0;0	0;0;0
groupXI	6681500	6689000	2;3;4;	62;2;2;	6;2;2	0;0;0	2;1;0	2;0;2	2;1;0	0;0;0	0;0;0	0;0;0	0;0;0	0;0;0	0;0;0	0;0;0
groupXI	7003500	7006500	1;2;	4;62;	4;0;4	0;0;0	0;0;0	0;0;0	0;0;0	0;0;0	0;0;0	0;0;0	1;0;1	1;0;1	1;0;1	1;0;1
groupXI	7561000	7562000	1;2;	23;43;	23;0;23	0;0;0	0;0;0	0;0;0	0;0;0	0;0;0	0;0;0	0;0;0	5;0;5	6;0;6	6;0;6	6;0;6
groupXI	7767500	7784000	2;3;	65;1;	1;0;1	0;0;0	0;0;0	0;0;0	0;0;0	1;0;1	0;0;0	0;0;0	0;0;0	0;0;0	0;0;0	0;0;0
groupXI	8890000	8895000	1;2;	4;62;	4;0;4	0;0;0	0;0;0	1;0;1	2;0;2	1;0;1	0;0;0	0;0;0	0;0;0	0;0;0	0;0;0	0;0;0
groupXI	9200000	9207000	2;3;4;	64;1;1;	3;1;1	0;0;0	3;1;1	0;0;0	0;0;0	0;0;0	0;0;0	0;0;0	0;0;0	0;0;0	0;0;0	0;0;0
groupXI	9964500	9970500	2;3;	65;1;	1;0;1	0;0;0	1;0;1	0;0;0	0;0;0	0;0;0	0;0;0	0;0;0	0;0;0	0;0;0	0;0;0	0;0;0
groupXI	9995500	10002000	1;2;	1;65;	1;0;1	1;0;1	0;0;0	0;0;0	0;0;0	0;0;0	0;0;0	0;0;0	0;0;0	0;0;0	0;0;0	0;0;0
groupXI	10722500	10726500	1;2;	1;65;	1;0;1	0;0;0	0;0;0	0;0;0	0;0;0	0;0;0	0;0;0	0;0;0	1;0;1	0;0;0	0;0;0	0;0;0
groupXI	11027500	11050500	2;3;	65;1;	1;0;1	0;0;0	1;0;1	0;0;0	0;0;0	0;0;0	0;0;0	0;0;0	0;0;0	0;0;0	0;0;0	0;0;0
groupXI	11099000	11102500	1;2;	1;65;	1;0;1	0;0;0	0;0;0	0;0;0	0;0;0	0;0;0	0;0;0	1;0;1	0;0;0	0;0;0	0;0;0	0;0;0
groupXI	11288000	11295500	2;3;	64;2;	2;0;2	0;0;0	0;0;0	0;0;0	0;0;0	0;0;0	0;0;0	2;0;2	0;0;0	0;0;0	0;0;0	0;0;0
groupXI	11626000	11627000	0;1;2;	3;14;49;	20;3;14	2;0;2	3;1;1	8;2;4	4;0;4	3;0;3	0;0;0	0;0;0	0;0;0	0;0;0	0;0;0	0;0;0
groupXI	12082000	12091000	2;3;	47;19;	19;0;19	0;0;0	0;0;0	0;0;0	0;0;0	0;0;0	0;0;0	0;0;0	6;0;6	3;0;3	4;0;4	6;0;6
groupXI	12235500	12237000	0;1;2;	2;5;59;	9;2;5	0;0;0	0;0;0	0;0;0	0;0;0	0;0;0	7;2;3	2;0;2	0;0;0	0;0;0	0;0;0	0;0;0
groupXI	12716500	12721500	2;3;	65;1;	1;0;1	0;0;0	0;0;0	0;0;0	0;0;0	0;0;0	0;0;0	0;0;0	0;0;0	1;0;1	0;0;0	0;0;0
groupXI	12834500	12839000	1;2;	2;64;	2;0;2	0;0;0	0;0;0	0;0;0	1;0;1	0;0;0	0;0;0	0;0;0	0;0;0	0;0;0	1;0;1	0;0;0
groupXI	13011000	13018500	2;3;4;	53;12;1;	14;1;12	1;0;1	0;0;0	5;0;5	0;0;0	1;0;1	0;0;0	0;0;0	0;0;0	0;0;0	4;1;2	3;0;3

groupXI	13177500	13180500	1;2;	6;60;	6;0;6	0;0;0	0;0;0	0;0;0	0;0;0	0;0;0	0;0;0	0;0;0	1;0;1	2;0;2	2;0;2	1;0;1
groupXI	13614000	13625000	1;2;	1;65;	1;0;1	0;0;0	0;0;0	0;0;0	0;0;0	0;0;0	0;0;0	0;0;0	0;0;0	0;0;0	0;0;0	1;0;1
groupXI	13903000	13908000	1;2;	1;65;	1;0;1	0;0;0	1;0;1	0;0;0	0;0;0	0;0;0	0;0;0	0;0;0	0;0;0	0;0;0	0;0;0	0;0;0
groupXI	14108000	14111500	2;3;	64;2;	2;0;2	0;0;0	0;0;0	0;0;0	0;0;0	0;0;0	0;0;0	0;0;0	2;0;2	0;0;0	0;0;0	0;0;0
groupXI	14967000	14981000	2;3;	64;2;	2;0;2	0;0;0	0;0;0	0;0;0	2;0;2	0;0;0	0;0;0	0;0;0	0;0;0	0;0;0	0;0;0	0;0;0
groupXI	15088000	15101500	2;3;	64;2;	2;0;2	0;0;0	0;0;0	0;0;0	0;0;0	0;0;0	0;0;0	0;0;0	1;0;1	1;0;1	0;0;0	0;0;0
groupXI	15420500	15430000	1;2;	1;65;	1;0;1	0;0;0	1;0;1	0;0;0	0;0;0	0;0;0	0;0;0	0;0;0	0;0;0	0;0;0	0;0;0	0;0;0
groupXI	15858000	15863000	2;3;	65;1;	1;0;1	0;0;0	0;0;0	0;0;0	0;0;0	0;0;0	0;0;0	1;0;1	0;0;0	0;0;0	0;0;0	0;0;0
groupXI	16200000	16202500	1;2;	1;65;	1;0;1	0;0;0	0;0;0	0;0;0	0;0;0	1;0;1	0;0;0	0;0;0	0;0;0	0;0;0	0;0;0	0;0;0
groupXII	65000	69500	1;2;	2;64;	2;0;2	0;0;0	0;0;0	0;0;0	1;0;1	0;0;0	0;0;0	0;0;0	0;0;0	0;0;0	0;0;0	1;0;1
groupXII	99000	117500	2;3;	63;3;	3;0;3	0;0;0	0;0;0	0;0;0	0;0;0	0;0;0	0;0;0	0;0;0	0;0;0	1;0;1	2;0;2	0;0;0
groupXII	2272000	2275000	0;2;	1;65;	2;1;0	2;1;0	0;0;0	0;0;0	0;0;0	0;0;0	0;0;0	0;0;0	0;0;0	0;0;0	0;0;0	0;0;0
groupXII	2371000	2385000	2;3;	65;1;	1;0;1	0;0;0	0;0;0	0;0;0	0;0;0	0;0;0	0;0;0	0;0;0	0;0;0	1;0;1	0;0;0	0;0;0
groupXII	2653500	2657000	0;1;2;	4;3;59;	11;4;3	0;0;0	0;0;0	0;0;0	0;0;0	0;0;0	0;0;0	0;0;0	0;0;0	0;0;0	9;4;1	2;0;2
groupXII	2669500	2672000	2;3;	63;3;	3;0;3	0;0;0	2;0;2	1;0;1	0;0;0	0;0;0	0;0;0	0;0;0	0;0;0	0;0;0	0;0;0	0;0;0
groupXII	2708500	2710500	1;2;	8;58;	8;0;8	0;0;0	0;0;0	0;0;0	0;0;0	0;0;0	0;0;0	0;0;0	3;0;3	5;0;5	0;0;0	0;0;0
groupXII	2754000	2758500	2;3;	62;4;	4;0;4	0;0;0	0;0;0	0;0;0	0;0;0	0;0;0	0;0;0	0;0;0	3;0;3	1;0;1	0;0;0	0;0;0
groupXII	2839500	2847500	2;3;	65;1;	1;0;1	0;0;0	0;0;0	0;0;0	0;0;0	1;0;1	0;0;0	0;0;0	0;0;0	0;0;0	0;0;0	0;0;0
groupXII	2965000	2967500	0;1;2;	1;2;63;	4;1;2	0;0;0	2;1;0	1;0;1	1;0;1	0;0;0	0;0;0	0;0;0	0;0;0	0;0;0	0;0;0	0;0;0
groupXII	3512000	3514500	1;2;	1;65;	1;0;1	0;0;0	0;0;0	0;0;0	0;0;0	0;0;0	0;0;0	0;0;0	1;0;1	0;0;0	0;0;0	0;0;0
groupXII	3862500	3864000	0;1;2;	6;3;57;	15;6;3	0;0;0	0;0;0	0;0;0	0;0;0	0;0;0	10;4;2	5;2;1	0;0;0	0;0;0	0;0;0	0;0;0
groupXII	4317500	4320500	1;2;	1;65;	1;0;1	0;0;0	0;0;0	0;0;0	1;0;1	0;0;0	0;0;0	0;0;0	0;0;0	0;0;0	0;0;0	0;0;0
groupXII	4399000	4403500	0;1;2;	3;10;53;	16;3;10	1;0;1	0;0;0	1;0;1	1;0;1	0;0;0	7;2;3	6;1;4	0;0;0	0;0;0	0;0;0	0;0;0
groupXII	4730000	4734500	1;2;	1;65;	1;0;1	0;0;0	0;0;0	0;0;0	0;0;0	1;0;1	0;0;0	0;0;0	0;0;0	0;0;0	0;0;0	0;0;0
groupXII	5085000	5092000	2;3;	63;3;	3;0;3	0;0;0	0;0;0	0;0;0	0;0;0	3;0;3	0;0;0	0;0;0	0;0;0	0;0;0	0;0;0	0;0;0
groupXII	5114000	5115500	1;2;	6;60;	6;0;6	0;0;0	0;0;0	2;0;2	0;0;0	0;0;0	0;0;0	0;0;0	1;0;1	1;0;1	0;0;0	2;0;2
groupXII	5427500	5434000	2;3;	65;1;	1;0;1	0;0;0	0;0;0	0;0;0	0;0;0	0;0;0	0;0;0	0;0;0	1;0;1	0;0;0	0;0;0	0;0;0
groupXII	5433500	5435500	0;1;2;	2;5;59;	9;2;5	0;0;0	0;0;0	0;0;0	0;0;0	0;0;0	8;2;4	1;0;1	0;0;0	0;0;0	0;0;0	0;0;0
groupXII	6243000	6251000	1;2;	1;65;	1;0;1	0;0;0	0;0;0	0;0;0	0;0;0	1;0;1	0;0;0	0;0;0	0;0;0	0;0;0	0;0;0	0;0;0
groupXII	7160000	7163000	0;1;2;	1;4;61;	6;1;4	0;0;0	0;0;0	6;1;4	0;0;0	0;0;0	0;0;0	0;0;0	0;0;0	0;0;0	0;0;0	0;0;0
groupXII	7223500	7225500	0;1;2;	1;8;57;	10;1;8	0;0;0	0;0;0	0;0;0	0;0;0	0;0;0	0;0;0	0;0;0	3;0;3	6;1;4	0;0;0	1;0;1
groupXII	7230500	7234000	1;2;	5;61;	5;0;5	0;0;0	2;0;2	0;0;0	3;0;3	0;0;0	0;0;0	0;0;0	0;0;0	0;0;0	0;0;0	0;0;0
groupXII	7305500	7309500	1;2;	1;65;	1;0;1	0;0;0	0;0;0	0;0;0	0;0;0	0;0;0	0;0;0	1;0;1	0;0;0	0;0;0	0;0;0	0;0;0
groupXII	7361500	7364500	1;2;	1;65;	1;0;1	0;0;0	0;0;0	0;0;0	0;0;0	0;0;0	0;0;0	0;0;0	1;0;1	0;0;0	0;0;0	0;0;0

groupXII	7848000	7860000	2;3;	64;2;	2;0;2	0;0;0	0;0;0	0;0;0	2;0;2	0;0;0	0;0;0	0;0;0	0;0;0	0;0;0	0;0;0	0;0;0
groupXII	7865000	7878500	2;3;4;	61;4;1;	6;1;4	5;1;3	0;0;0	0;0;0	0;0;0	1;0;1	0;0;0	0;0;0	0;0;0	0;0;0	0;0;0	0;0;0
groupXII	8040000	8042500	0;1;2;	1;3;62;	5;1;3	0;0;0	3;1;1	0;0;0	0;0;0	1;0;1	0;0;0	0;0;0	1;0;1	0;0;0	0;0;0	0;0;0
groupXII	8104000	8110000	2;3;	65;1;	1;0;1	1;0;1	0;0;0	0;0;0	0;0;0	0;0;0	0;0;0	0;0;0	0;0;0	0;0;0	0;0;0	0;0;0
groupXII	8919500	8921500	0;1;2;	1;2;63;	4;1;2	0;0;0	0;0;0	0;0;0	0;0;0	0;0;0	1;0;1	0;0;0	3;1;1	0;0;0	0;0;0	0;0;0
groupXII	9330000	9331500	2;3;	64;2;	2;0;2	0;0;0	0;0;0	0;0;0	0;0;0	0;0;0	1;0;1	0;0;0	1;0;1	0;0;0	0;0;0	0;0;0
groupXII	9768000	9770500	1;2;	1;65;	1;0;1	0;0;0	0;0;0	0;0;0	0;0;0	0;0;0	0;0;0	0;0;0	0;0;0	1;0;1	0;0;0	0;0;0
groupXII	9995000	10004500	1;2;	5;61;	5;0;5	1;0;1	0;0;0	1;0;1	0;0;0	0;0;0	0;0;0	0;0;0	0;0;0	0;0;0	1;0;1	2;0;2
groupXII	10054000	10057500	1;2;	3;63;	3;0;3	0;0;0	2;0;2	0;0;0	1;0;1	0;0;0	0;0;0	0;0;0	0;0;0	0;0;0	0;0;0	0;0;0
groupXII	10097000	10105000	2;3;4;	63;2;1;	4;1;2	0;0;0	0;0;0	0;0;0	0;0;0	0;0;0	4;1;2	0;0;0	0;0;0	0;0;0	0;0;0	0;0;0
groupXII	10123000	10128000	2;3;	45;21;	21;0;21	0;0;0	0;0;0	0;0;0	0;0;0	0;0;0	0;0;0	0;0;0	4;0;4	5;0;5	6;0;6	6;0;6
groupXII	10266500	10270500	1;2;	2;64;	2;0;2	0;0;0	0;0;0	0;0;0	0;0;0	0;0;0	0;0;0	0;0;0	0;0;0	2;0;2	0;0;0	0;0;0
groupXII	10286000	10327000	2;3;	64;2;	2;0;2	0;0;0	0;0;0	0;0;0	0;0;0	0;0;0	0;0;0	2;0;2	0;0;0	0;0;0	0;0;0	0;0;0
groupXII	10786500	10793000	2;3;	62;4;	4;0;4	0;0;0	0;0;0	0;0;0	0;0;0	0;0;0	2;0;2	2;0;2	0;0;0	0;0;0	0;0;0	0;0;0
groupXII	11896500	11901500	1;2;	1;65;	1;0;1	0;0;0	0;0;0	0;0;0	0;0;0	0;0;0	0;0;0	1;0;1	0;0;0	0;0;0	0;0;0	0;0;0
groupXII	12998500	13015500	2;4;	65;1;	2;1;0	0;0;0	0;0;0	0;0;0	0;0;0	0;0;0	0;0;0	2;1;0	0;0;0	0;0;0	0;0;0	0;0;0
groupXII	13373000	13382000	2;3;4;	64;1;1;	3;1;1	0;0;0	0;0;0	2;1;0	0;0;0	1;0;1	0;0;0	0;0;0	0;0;0	0;0;0	0;0;0	0;0;0
groupXII	14437500	14460000	2;3;4;	45;17;4;	25;4;17	0;0;0	6;2;2	1;0;1	2;0;2	1;0;1	0;0;0	2;0;2	6;1;4	6;1;4	0;0;0	1;0;1
groupXII	14739500	14740500	0;1;2;	8;2;56;	18;8;2	0;0;0	0;0;0	0;0;0	0;0;0	0;0;0	0;0;0	0;0;0	0;0;0	0;0;0	12;6;0	6;2;2
groupXII	15918500	15922000	1;2;	1;65;	1;0;1	0;0;0	0;0;0	0;0;0	0;0;0	0;0;0	0;0;0	0;0;0	1;0;1	0;0;0	0;0;0	0;0;0
groupXII	16318000	16321000	1;2;	1;65;	1;0;1	0;0;0	1;0;1	0;0;0	0;0;0	0;0;0	0;0;0	0;0;0	0;0;0	0;0;0	0;0;0	0;0;0
groupXII	16457000	16469000	0;1;2;	2;11;53;	15;2;11	0;0;0	0;0;0	0;0;0	0;0;0	0;0;0	0;0;0	0;0;0	4;0;4	2;0;2	2;0;2	7;2;3
groupXII	16524000	16548500	2;3;	59;7;	7;0;7	0;0;0	0;0;0	0;0;0	0;0;0	0;0;0	0;0;0	0;0;0	4;0;4	3;0;3	0;0;0	0;0;0
groupXII	16581500	16586000	1;2;	2;64;	2;0;2	0;0;0	0;0;0	0;0;0	0;0;0	0;0;0	0;0;0	0;0;0	0;0;0	2;0;2	0;0;0	0;0;0
groupXII	16749000	16751000	1;2;	16;50;	16;0;16	1;0;1	0;0;0	0;0;0	1;0;1	0;0;0	0;0;0	1;0;1	1;0;1	3;0;3	4;0;4	5;0;5
groupXII	17056000	17059000	0;1;2;	1;9;56;	11;1;9	1;0;1	1;0;1	0;0;0	0;0;0	0;0;0	4;1;2	5;0;5	0;0;0	0;0;0	0;0;0	0;0;0
groupXII	17102000	17106000	1;2;	4;62;	4;0;4	1;0;1	0;0;0	0;0;0	0;0;0	0;0;0	0;0;0	0;0;0	3;0;3	0;0;0	0;0;0	0;0;0
groupXII	17233500	17234500	0;1;2;	1;2;63;	4;1;2	4;1;2	0;0;0	0;0;0	0;0;0	0;0;0	0;0;0	0;0;0	0;0;0	0;0;0	0;0;0	0;0;0
groupXII	17535000	17537000	1;2;	3;63;	3;0;3	0;0;0	0;0;0	0;0;0	0;0;0	0;0;0	2;0;2	1;0;1	0;0;0	0;0;0	0;0;0	0;0;0
groupXII	17629500	17634000	1;2;	11;55;	11;0;11	0;0;0	0;0;0	0;0;0	1;0;1	0;0;0	0;0;0	0;0;0	5;0;5	5;0;5	0;0;0	0;0;0
groupXII	18062000	18068500	2;3;4;	64;1;1;	3;1;1	0;0;0	0;0;0	0;0;0	0;0;0	2;1;0	0;0;0	1;0;1	0;0;0	0;0;0	0;0;0	0;0;0
groupXIII	549500	551500	0;1;2;	1;1;64;	3;1;1	1;0;1	0;0;0	0;0;0	0;0;0	0;0;0	0;0;0	2;1;0	0;0;0	0;0;0	0;0;0	0;0;0
groupXIII	1035500	1040000	1;2;	11;55;	11;0;11	0;0;0	0;0;0	0;0;0	0;0;0	0;0;0	0;0;0	0;0;0	3;0;3	1;0;1	6;0;6	1;0;1
groupXIII	1066500	1071000	1;2;	1;65;	1;0;1	1;0;1	0;0;0	0;0;0	0;0;0	0;0;0	0;0;0	0;0;0	0;0;0	0;0;0	0;0;0	0;0;0

groupXIII	1169000	1194000	1;2;	3;63;	3;0;3	0;0;0	0;0;0	0;0;0	0;0;0	0;0;0	0;0;0	0;0;0	1;0;1	2;0;2	0;0;0	0;0;0
groupXIII	1196000	1203500	2;3;	65;1;	1;0;1	0;0;0	0;0;0	0;0;0	0;0;0	0;0;0	0;0;0	1;0;1	0;0;0	0;0;0	0;0;0	0;0;0
groupXIII	2999000	3001500	1;2;	5;61;	5;0;5	1;0;1	4;0;4	0;0;0	0;0;0	0;0;0	0;0;0	0;0;0	0;0;0	0;0;0	0;0;0	0;0;0
groupXIII	3219000	3262000	2;3;	63;3;	3;0;3	1;0;1	0;0;0	0;0;0	0;0;0	1;0;1	0;0;0	0;0;0	0;0;0	1;0;1	0;0;0	0;0;0
groupXIII	3299000	3302500	1;2;	1;65;	1;0;1	1;0;1	0;0;0	0;0;0	0;0;0	0;0;0	0;0;0	0;0;0	0;0;0	0;0;0	0;0;0	0;0;0
groupXIII	3312000	3315500	1;2;	2;64;	2;0;2	0;0;0	0;0;0	0;0;0	0;0;0	0;0;0	0;0;0	0;0;0	2;0;2	0;0;0	0;0;0	0;0;0
groupXIII	3371000	3378000	2;3;	62;4;	4;0;4	0;0;0	3;0;3	0;0;0	1;0;1	0;0;0	0;0;0	0;0;0	0;0;0	0;0;0	0;0;0	0;0;0
groupXIII	3669500	3675500	1;2;	10;56;	10;0;10	0;0;0	0;0;0	0;0;0	0;0;0	0;0;0	0;0;0	0;0;0	5;0;5	4;0;4	0;0;0	1;0;1
groupXIII	3762500	3765000	0;1;2;	1;6;59;	8;1;6	0;0;0	0;0;0	4;1;2	1;0;1	1;0;1	0;0;0	0;0;0	1;0;1	0;0;0	1;0;1	0;0;0
groupXIII	3942000	3943500	0;1;2;	2;5;59;	9;2;5	5;2;1	0;0;0	0;0;0	3;0;3	1;0;1	0;0;0	0;0;0	0;0;0	0;0;0	0;0;0	0;0;0
groupXIII	4211500	4376000	2;3;	61;5;	5;0;5	0;0;0	1;0;1	1;0;1	1;0;1	1;0;1	0;0;0	1;0;1	0;0;0	0;0;0	0;0;0	0;0;0
groupXIII	5086000	5093500	2;3;	65;1;	1;0;1	0;0;0	0;0;0	0;0;0	0;0;0	0;0;0	0;0;0	0;0;0	0;0;0	0;0;0	0;0;0	1;0;1
groupXIII	5142500	5144500	1;2;	2;64;	2;0;2	0;0;0	0;0;0	2;0;2	0;0;0	0;0;0	0;0;0	0;0;0	0;0;0	0;0;0	0;0;0	0;0;0
groupXIII	5240000	5244000	1;2;	2;64;	2;0;2	0;0;0	2;0;2	0;0;0	0;0;0	0;0;0	0;0;0	0;0;0	0;0;0	0;0;0	0;0;0	0;0;0
groupXIII	5371000	5372500	0;1;2;	1;6;59;	8;1;6	0;0;0	0;0;0	5;1;3	0;0;0	2;0;2	0;0;0	0;0;0	1;0;1	0;0;0	0;0;0	0;0;0
groupXIII	5500500	5504000	1;2;	1;65;	1;0;1	1;0;1	0;0;0	0;0;0	0;0;0	0;0;0	0;0;0	0;0;0	0;0;0	0;0;0	0;0;0	0;0;0
groupXIII	5548000	5551000	1;2;	1;65;	1;0;1	0;0;0	1;0;1	0;0;0	0;0;0	0;0;0	0;0;0	0;0;0	0;0;0	0;0;0	0;0;0	0;0;0
groupXIII	5956500	5959000	1;2;	2;64;	2;0;2	0;0;0	0;0;0	2;0;2	0;0;0	0;0;0	0;0;0	0;0;0	0;0;0	0;0;0	0;0;0	0;0;0
groupXIII	6331500	6335000	1;2;	1;65;	1;0;1	1;0;1	0;0;0	0;0;0	0;0;0	0;0;0	0;0;0	0;0;0	0;0;0	0;0;0	0;0;0	0;0;0
groupXIII	6699000	6700500	1;2;	4;62;	4;0;4	0;0;0	0;0;0	3;0;3	0;0;0	1;0;1	0;0;0	0;0;0	0;0;0	0;0;0	0;0;0	0;0;0
groupXIII	6905500	6909000	1;2;	3;63;	3;0;3	0;0;0	0;0;0	0;0;0	0;0;0	2;0;2	0;0;0	0;0;0	1;0;1	0;0;0	0;0;0	0;0;0
groupXIII	7070500	7075000	0;1;2;	1;3;62;	5;1;3	0;0;0	5;1;3	0;0;0	0;0;0	0;0;0	0;0;0	0;0;0	0;0;0	0;0;0	0;0;0	0;0;0
groupXIII	7872500	7875000	0;1;2;	1;3;62;	5;1;3	0;0;0	1;0;1	0;0;0	3;1;1	1;0;1	0;0;0	0;0;0	0;0;0	0;0;0	0;0;0	0;0;0
groupXIII	8310000	8316500	1;2;	1;65;	1;0;1	0;0;0	0;0;0	0;0;0	0;0;0	0;0;0	0;0;0	0;0;0	0;0;0	0;0;0	0;0;0	1;0;1
groupXIII	8805000	8809000	1;2;	1;65;	1;0;1	1;0;1	0;0;0	0;0;0	0;0;0	0;0;0	0;0;0	0;0;0	0;0;0	0;0;0	0;0;0	0;0;0
groupXIII	8816500	8820000	1;2;	2;64;	2;0;2	0;0;0	0;0;0	0;0;0	0;0;0	0;0;0	0;0;0	0;0;0	0;0;0	1;0;1	1;0;1	0;0;0
groupXIII	8822000	8844500	2;3;4;	64;1;1;	3;1;1	0;0;0	0;0;0	0;0;0	3;1;1	0;0;0	0;0;0	0;0;0	0;0;0	0;0;0	0;0;0	0;0;0
groupXIII	9481000	9490500	2;3;	64;2;	2;0;2	0;0;0	0;0;0	1;0;1	0;0;0	1;0;1	0;0;0	0;0;0	0;0;0	0;0;0	0;0;0	0;0;0
groupXIII	9776500	9779000	1;2;	2;64;	2;0;2	0;0;0	0;0;0	0;0;0	0;0;0	0;0;0	0;0;0	0;0;0	0;0;0	1;0;1	1;0;1	0;0;0
groupXIII	10179000	10181500	0;1;2;	1;3;62;	5;1;3	0;0;0	2;1;0	2;0;2	0;0;0	1;0;1	0;0;0	0;0;0	0;0;0	0;0;0	0;0;0	0;0;0
groupXIII	10658500	10660000	0;1;2;	1;2;63;	4;1;2	0;0;0	4;1;2	0;0;0	0;0;0	0;0;0	0;0;0	0;0;0	0;0;0	0;0;0	0;0;0	0;0;0
groupXIII	11343000	11344000	0;1;2;	2;12;52;	16;2;12	2;0;2	0;0;0	2;0;2	0;0;0	0;0;0	7;2;3	4;0;4	0;0;0	1;0;1	0;0;0	0;0;0
groupXIII	11365500	11368000	1;2;	1;65;	1;0;1	0;0;0	0;0;0	1;0;1	0;0;0	0;0;0	0;0;0	0;0;0	0;0;0	0;0;0	0;0;0	0;0;0
groupXIII	11528500	11540000	2;3;	65;1;	1;0;1	0;0;0	0;0;0	0;0;0	0;0;0	1;0;1	0;0;0	0;0;0	0;0;0	0;0;0	0;0;0	0;0;0

groupXIII	11776500	11780000	1;2;	1;65;	1;0;1	0;0;0	0;0;0	0;0;0	0;0;0	0;0;0	0;0;0	0;0;0	0;0;0	0;0;0	0;0;0	1;0;1
groupXIII	11826500	11831500	2;3;	53;13;	13;0;13	0;0;0	0;0;0	0;0;0	0;0;0	0;0;0	0;0;0	0;0;0	6;0;6	4;0;4	3;0;3	0;0;0
groupXIII	12127500	12136000	2;3;	65;1;	1;0;1	0;0;0	0;0;0	0;0;0	0;0;0	0;0;0	0;0;0	1;0;1	0;0;0	0;0;0	0;0;0	0;0;0
groupXIII	12334000	12345000	2;3;	64;2;	2;0;2	0;0;0	0;0;0	0;0;0	2;0;2	0;0;0	0;0;0	0;0;0	0;0;0	0;0;0	0;0;0	0;0;0
groupXIII	13130500	13140000	2;3;	64;2;	2;0;2	0;0;0	0;0;0	2;0;2	0;0;0	0;0;0	0;0;0	0;0;0	0;0;0	0;0;0	0;0;0	0;0;0
groupXIII	13649000	13651500	1;2;	2;64;	2;0;2	0;0;0	0;0;0	0;0;0	0;0;0	0;0;0	0;0;0	0;0;0	0;0;0	0;0;0	0;0;0	2;0;2
groupXIII	13960500	13970500	1;2;	1;65;	1;0;1	0;0;0	0;0;0	0;0;0	0;0;0	0;0;0	0;0;0	0;0;0	0;0;0	1;0;1	0;0;0	0;0;0
groupXIII	14242500	14245500	0;1;2;	2;3;61;	7;2;3	0;0;0	7;2;3	0;0;0	0;0;0	0;0;0	0;0;0	0;0;0	0;0;0	0;0;0	0;0;0	0;0;0
groupXIII	14421000	14428000	2;3;	65;1;	1;0;1	0;0;0	0;0;0	0;0;0	0;0;0	0;0;0	0;0;0	0;0;0	0;0;0	1;0;1	0;0;0	0;0;0
groupXIII	14461000	14467500	2;3;4;	63;2;1;	4;1;2	0;0;0	0;0;0	0;0;0	0;0;0	0;0;0	4;1;2	0;0;0	0;0;0	0;0;0	0;0;0	0;0;0
groupXIII	14615500	14616500	0;1;2;	1;1;64;	3;1;1	0;0;0	0;0;0	0;0;0	0;0;0	0;0;0	0;0;0	0;0;0	3;1;1	0;0;0	0;0;0	0;0;0
groupXIII	14620000	14622500	1;2;	2;64;	2;0;2	0;0;0	0;0;0	0;0;0	1;0;1	0;0;0	0;0;0	0;0;0	0;0;0	1;0;1	0;0;0	0;0;0
groupXIII	14700500	14702000	0;1;2;	1;15;50;	17;1;15	2;0;2	1;0;1	0;0;0	5;0;5	1;0;1	2;0;2	6;1;4	0;0;0	0;0;0	0;0;0	0;0;0
groupXIII	14718500	14728000	2;3;	64;2;	2;0;2	0;0;0	0;0;0	0;0;0	0;0;0	0;0;0	0;0;0	0;0;0	0;0;0	2;0;2	0;0;0	0;0;0
groupXIII	15510000	15513500	1;2;	1;65;	1;0;1	1;0;1	0;0;0	0;0;0	0;0;0	0;0;0	0;0;0	0;0;0	0;0;0	0;0;0	0;0;0	0;0;0
groupXIII	16087500	16102000	2;3;	65;1;	1;0;1	0;0;0	0;0;0	0;0;0	0;0;0	0;0;0	0;0;0	0;0;0	1;0;1	0;0;0	0;0;0	0;0;0
groupXIII	16811000	16813500	0;1;2;	2;1;63;	5;2;1	0;0;0	0;0;0	0;0;0	0;0;0	0;0;0	5;2;1	0;0;0	0;0;0	0;0;0	0;0;0	0;0;0
groupXIII	16869000	16876500	2;3;4;	61;4;1;	6;1;4	0;0;0	0;0;0	0;0;0	0;0;0	0;0;0	4;1;2	2;0;2	0;0;0	0;0;0	0;0;0	0;0;0
groupXIII	16903500	16906000	1;2;	1;65;	1;0;1	0;0;0	1;0;1	0;0;0	0;0;0	0;0;0	0;0;0	0;0;0	0;0;0	0;0;0	0;0;0	0;0;0
groupXIII	17309500	17312500	1;2;	1;65;	1;0;1	1;0;1	0;0;0	0;0;0	0;0;0	0;0;0	0;0;0	0;0;0	0;0;0	0;0;0	0;0;0	0;0;0
groupXIII	17329500	17341500	2;3;	65;1;	1;0;1	0;0;0	0;0;0	1;0;1	0;0;0	0;0;0	0;0;0	0;0;0	0;0;0	0;0;0	0;0;0	0;0;0
groupXIII	17347000	17355500	2;3;	65;1;	1;0;1	0;0;0	0;0;0	0;0;0	0;0;0	0;0;0	0;0;0	0;0;0	0;0;0	1;0;1	0;0;0	0;0;0
groupXIII	17433500	17442500	2;3;	55;11;	11;0;11	3;0;3	0;0;0	5;0;5	2;0;2	1;0;1	0;0;0	0;0;0	0;0;0	0;0;0	0;0;0	0;0;0
groupXIII	17774500	17778500	0;1;2;	1;2;63;	4;1;2	0;0;0	0;0;0	0;0;0	0;0;0	0;0;0	0;0;0	0;0;0	0;0;0	0;0;0	4;1;2	0;0;0
groupXIII	17791000	17792500	1;2;	3;63;	3;0;3	0;0;0	0;0;0	2;0;2	0;0;0	0;0;0	0;0;0	0;0;0	0;0;0	1;0;1	0;0;0	0;0;0
groupXIII	17918500	17921000	1;2;	2;64;	2;0;2	0;0;0	0;0;0	2;0;2	0;0;0	0;0;0	0;0;0	0;0;0	0;0;0	0;0;0	0;0;0	0;0;0
groupXIII	18074500	18079500	1;2;	5;61;	5;0;5	3;0;3	0;0;0	0;0;0	1;0;1	1;0;1	0;0;0	0;0;0	0;0;0	0;0;0	0;0;0	0;0;0
groupXIII	18083000	18085500	1;2;	1;65;	1;0;1	1;0;1	0;0;0	0;0;0	0;0;0	0;0;0	0;0;0	0;0;0	0;0;0	0;0;0	0;0;0	0;0;0
groupXIII	18443000	18448500	1;2;	1;65;	1;0;1	0;0;0	0;0;0	0;0;0	0;0;0	0;0;0	0;0;0	0;0;0	0;0;0	0;0;0	0;0;0	1;0;1
groupXIII	18680500	18683000	1;2;	2;64;	2;0;2	1;0;1	0;0;0	0;0;0	0;0;0	0;0;0	0;0;0	0;0;0	0;0;0	1;0;1	0;0;0	0;0;0
groupXIII	18685500	18689000	0;1;2;	1;5;60;	7;1;5	0;0;0	2;0;2	0;0;0	1;0;1	0;0;0	4;1;2	0;0;0	0;0;0	0;0;0	0;0;0	0;0;0
groupXIII	19042000	19044500	1;2;	13;53;	13;0;13	3;0;3	5;0;5	0;0;0	1;0;1	0;0;0	0;0;0	3;0;3	1;0;1	0;0;0	0;0;0	0;0;0
groupXIII	19352000	19355500	1;2;	1;65;	1;0;1	0;0;0	0;0;0	0;0;0	1;0;1	0;0;0	0;0;0	0;0;0	0;0;0	0;0;0	0;0;0	0;0;0
groupXIII	19457500	19475000	2;3;	65;1;	1;0;1	1;0;1	0;0;0	0;0;0	0;0;0	0;0;0	0;0;0	0;0;0	0;0;0	0;0;0	0;0;0	0;0;0

groupXIII	19884500	19887000	0;1;2;	1;5;60;	7;1;5	0;0;0	0;0;0	0;0;0	0;0;0	0;0;0	0;0;0	0;0;0	2;0;2	1;0;1	0;0;0	4;1;2
groupXIV	72000	80000	2;3;	65;1;	1;0;1	0;0;0	0;0;0	0;0;0	1;0;1	0;0;0	0;0;0	0;0;0	0;0;0	0;0;0	0;0;0	0;0;0
groupXIV	122500	126000	1;2;	2;64;	2;0;2	0;0;0	0;0;0	0;0;0	0;0;0	0;0;0	0;0;0	0;0;0	1;0;1	1;0;1	0;0;0	0;0;0
groupXIV	388500	397000	2;3;	65;1;	1;0;1	1;0;1	0;0;0	0;0;0	0;0;0	0;0;0	0;0;0	0;0;0	0;0;0	0;0;0	0;0;0	0;0;0
groupXIV	544500	551500	2;3;	64;2;	2;0;2	1;0;1	0;0;0	0;0;0	0;0;0	1;0;1	0;0;0	0;0;0	0;0;0	0;0;0	0;0;0	0;0;0
groupXIV	873000	875000	1;2;	15;51;	15;0;15	2;0;2	3;0;3	1;0;1	2;0;2	0;0;0	0;0;0	0;0;0	0;0;0	0;0;0	1;0;1	6;0;6
groupXIV	1695500	1703000	2;3;	65;1;	1;0;1	1;0;1	0;0;0	0;0;0	0;0;0	0;0;0	0;0;0	0;0;0	0;0;0	0;0;0	0;0;0	0;0;0
groupXIV	1818000	1821000	1;2;	1;65;	1;0;1	0;0;0	0;0;0	1;0;1	0;0;0	0;0;0	0;0;0	0;0;0	0;0;0	0;0;0	0;0;0	0;0;0
groupXIV	1881500	1883500	1;2;	7;59;	7;0;7	0;0;0	0;0;0	0;0;0	0;0;0	0;0;0	0;0;0	0;0;0	2;0;2	2;0;2	0;0;0	3;0;3
groupXIV	2406000	2409500	1;2;	2;64;	2;0;2	0;0;0	1;0;1	0;0;0	1;0;1	0;0;0	0;0;0	0;0;0	0;0;0	0;0;0	0;0;0	0;0;0
groupXIV	2646000	2650500	1;2;	1;65;	1;0;1	0;0;0	0;0;0	0;0;0	0;0;0	0;0;0	0;0;0	0;0;0	0;0;0	0;0;0	0;0;0	1;0;1
groupXIV	3230000	3234500	1;2;	1;65;	1;0;1	0;0;0	0;0;0	0;0;0	0;0;0	0;0;0	0;0;0	0;0;0	0;0;0	1;0;1	0;0;0	0;0;0
groupXIV	3533500	3539500	2;3;	63;3;	3;0;3	0;0;0	0;0;0	0;0;0	0;0;0	0;0;0	3;0;3	0;0;0	0;0;0	0;0;0	0;0;0	0;0;0
groupXIV	3554500	3564000	2;3;	65;1;	1;0;1	0;0;0	0;0;0	0;0;0	0;0;0	0;0;0	0;0;0	0;0;0	1;0;1	0;0;0	0;0;0	0;0;0
groupXIV	4096000	4099000	2;3;	62;4;	4;0;4	0;0;0	1;0;1	0;0;0	2;0;2	1;0;1	0;0;0	0;0;0	0;0;0	0;0;0	0;0;0	0;0;0
groupXIV	5203000	5204500	0;1;2;	1;4;61;	6;1;4	0;0;0	1;0;1	1;0;1	1;0;1	2;1;0	0;0;0	0;0;0	0;0;0	0;0;0	0;0;0	1;0;1
groupXIV	6019000	6024500	2;3;4;	61;4;1;	6;1;4	0;0;0	0;0;0	4;1;2	1;0;1	1;0;1	0;0;0	0;0;0	0;0;0	0;0;0	0;0;0	0;0;0
groupXIV	6392500	6396000	1;2;	1;65;	1;0;1	1;0;1	0;0;0	0;0;0	0;0;0	0;0;0	0;0;0	0;0;0	0;0;0	0;0;0	0;0;0	0;0;0
groupXIV	6573500	6583500	2;3;	63;3;	3;0;3	0;0;0	0;0;0	0;0;0	0;0;0	0;0;0	0;0;0	0;0;0	2;0;2	1;0;1	0;0;0	0;0;0
groupXIV	6953000	6962500	2;3;	65;1;	1;0;1	0;0;0	0;0;0	0;0;0	0;0;0	0;0;0	0;0;0	0;0;0	0;0;0	1;0;1	0;0;0	0;0;0
groupXIV	7332500	7339500	2;3;	65;1;	1;0;1	0;0;0	0;0;0	0;0;0	0;0;0	0;0;0	0;0;0	0;0;0	0;0;0	1;0;1	0;0;0	0;0;0
groupXIV	7945500	7951500	2;4;	65;1;	2;1;0	0;0;0	0;0;0	0;0;0	0;0;0	0;0;0	0;0;0	0;0;0	2;1;0	0;0;0	0;0;0	0;0;0
groupXIV	7976500	7981000	2;3;	65;1;	1;0;1	0;0;0	0;0;0	0;0;0	0;0;0	1;0;1	0;0;0	0;0;0	0;0;0	0;0;0	0;0;0	0;0;0
groupXIV	8621000	8622000	0;1;2;	4;2;60;	10;4;2	0;0;0	0;0;0	0;0;0	0;0;0	0;0;0	0;0;0	0;0;0	0;0;0	0;0;0	1;0;1	9;4;1
groupXIV	8779500	8783500	1;2;	3;63;	3;0;3	0;0;0	0;0;0	0;0;0	0;0;0	0;0;0	0;0;0	0;0;0	1;0;1	0;0;0	2;0;2	0;0;0
groupXIV	8821500	8825500	2;3;4;	59;6;1;	8;1;6	0;0;0	5;1;3	1;0;1	1;0;1	0;0;0	0;0;0	1;0;1	0;0;0	0;0;0	0;0;0	0;0;0
groupXIV	9774000	9775500	0;1;2;	1;5;60;	7;1;5	0;0;0	0;0;0	0;0;0	0;0;0	0;0;0	4;0;4	3;1;1	0;0;0	0;0;0	0;0;0	0;0;0
groupXIV	9893000	9894500	0;1;2;	1;1;64;	3;1;1	0;0;0	0;0;0	0;0;0	0;0;0	0;0;0	0;0;0	0;0;0	3;1;1	0;0;0	0;0;0	0;0;0
groupXIV	10553500	10557500	1;2;	1;65;	1;0;1	0;0;0	0;0;0	0;0;0	0;0;0	1;0;1	0;0;0	0;0;0	0;0;0	0;0;0	0;0;0	0;0;0
groupXIV	10710000	10712500	1;2;	6;60;	6;0;6	0;0;0	0;0;0	0;0;0	0;0;0	0;0;0	5;0;5	1;0;1	0;0;0	0;0;0	0;0;0	0;0;0
groupXIV	11224000	11231000	2;4;	65;1;	2;1;0	2;1;0	0;0;0	0;0;0	0;0;0	0;0;0	0;0;0	0;0;0	0;0;0	0;0;0	0;0;0	0;0;0
groupXIV	11265000	11274000	2;3;	65;1;	1;0;1	1;0;1	0;0;0	0;0;0	0;0;0	0;0;0	0;0;0	0;0;0	0;0;0	0;0;0	0;0;0	0;0;0
groupXIV	11536000	11537500	0;1;2;	1;5;60;	7;1;5	1;0;1	2;1;0	1;0;1	0;0;0	0;0;0	0;0;0	0;0;0	0;0;0	1;0;1	0;0;0	2;0;2
groupXIV	11575500	11579000	1;2;	1;65;	1;0;1	0;0;0	0;0;0	0;0;0	0;0;0	0;0;0	0;0;0	0;0;0	0;0;0	0;0;0	1;0;1	0;0;0

groupXIV	11650500	11664000	2;3;	65;1;	1;0;1	0;0;0	0;0;0	0;0;0	0;0;0	0;0;0	0;0;0	1;0;1	0;0;0	0;0;0	0;0;0	0;0;0
groupXIV	12142500	12144000	0;1;2;	3;7;56;	13;3;7	0;0;0	0;0;0	0;0;0	0;0;0	0;0;0	0;0;0	0;0;0	0;0;0	0;0;0	4;0;4	9;3;3
groupXIV	12228000	12229500	0;1;2;	1;2;63;	4;1;2	2;1;0	0;0;0	1;0;1	1;0;1	0;0;0	0;0;0	0;0;0	0;0;0	0;0;0	0;0;0	0;0;0
groupXIV	12236500	12243500	1;2;	13;53;	13;0;13	2;0;2	0;0;0	3;0;3	6;0;6	2;0;2	0;0;0	0;0;0	0;0;0	0;0;0	0;0;0	0;0;0
groupXIV	12369000	12384000	2;3;	65;1;	1;0;1	0;0;0	0;0;0	1;0;1	0;0;0	0;0;0	0;0;0	0;0;0	0;0;0	0;0;0	0;0;0	0;0;0
groupXIV	12765000	12768000	1;2;	1;65;	1;0;1	0;0;0	0;0;0	1;0;1	0;0;0	0;0;0	0;0;0	0;0;0	0;0;0	0;0;0	0;0;0	0;0;0
groupXIV	13165500	13173000	1;2;	11;55;	11;0;11	0;0;0	1;0;1	0;0;0	0;0;0	0;0;0	0;0;0	0;0;0	2;0;2	1;0;1	5;0;5	2;0;2
groupXIV	13619500	13622500	0;1;2;	1;5;60;	7;1;5	2;1;0	0;0;0	0;0;0	0;0;0	0;0;0	0;0;0	0;0;0	2;0;2	2;0;2	1;0;1	0;0;0
groupXIV	13705000	13707500	0;1;2;	6;5;55;	17;6;5	0;0;0	0;0;0	0;0;0	0;0;0	0;0;0	11;5;1	6;1;4	0;0;0	0;0;0	0;0;0	0;0;0
groupXIV	13983000	13984500	0;1;2;	3;3;60;	9;3;3	0;0;0	0;0;0	0;0;0	0;0;0	0;0;0	0;0;0	0;0;0	3;1;1	6;2;2	0;0;0	0;0;0
groupXIV	14213000	14214500	0;1;2;	3;11;52;	17;3;11	0;0;0	0;0;0	0;0;0	0;0;0	0;0;0	0;0;0	0;0;0	8;2;4	5;1;3	0;0;0	4;0;4
groupXIV	14446000	14456500	2;3;	65;1;	1;0;1	0;0;0	0;0;0	0;0;0	0;0;0	1;0;1	0;0;0	0;0;0	0;0;0	0;0;0	0;0;0	0;0;0
groupXIV	14506000	14512500	2;3;4;	63;1;2;	5;2;1	0;0;0	0;0;0	0;0;0	0;0;0	0;0;0	0;0;0	0;0;0	3;1;1	2;1;0	0;0;0	0;0;0
groupXIV	14675000	14684500	2;3;	65;1;	1;0;1	0;0;0	0;0;0	0;0;0	0;0;0	0;0;0	0;0;0	0;0;0	0;0;0	1;0;1	0;0;0	0;0;0
groupXIV	14953500	14986500	2;3;	61;5;	5;0;5	0;0;0	0;0;0	2;0;2	1;0;1	2;0;2	0;0;0	0;0;0	0;0;0	0;0;0	0;0;0	0;0;0
groupXIV	15058000	15068500	1;2;	4;62;	4;0;4	0;0;0	0;0;0	2;0;2	2;0;2	0;0;0	0;0;0	0;0;0	0;0;0	0;0;0	0;0;0	0;0;0
groupXIV	15215500	15217500	0;1;2;	2;8;56;	12;2;8	0;0;0	0;0;0	0;0;0	0;0;0	0;0;0	0;0;0	0;0;0	5;1;3	7;1;5	0;0;0	0;0;0
groupXV	141000	151500	2;3;4;	62;2;2;	6;2;2	0;0;0	0;0;0	0;0;0	4;2;0	2;0;2	0;0;0	0;0;0	0;0;0	0;0;0	0;0;0	0;0;0
groupXV	272500	281500	2;3;	65;1;	1;0;1	1;0;1	0;0;0	0;0;0	0;0;0	0;0;0	0;0;0	0;0;0	0;0;0	0;0;0	0;0;0	0;0;0
groupXV	566000	580000	1;2;	1;65;	1;0;1	0;0;0	0;0;0	0;0;0	0;0;0	1;0;1	0;0;0	0;0;0	0;0;0	0;0;0	0;0;0	0;0;0
groupXV	702500	712000	2;3;	65;1;	1;0;1	0;0;0	0;0;0	0;0;0	0;0;0	0;0;0	0;0;0	0;0;0	1;0;1	0;0;0	0;0;0	0;0;0
groupXV	1612000	1616000	1;2;	1;65;	1;0;1	0;0;0	0;0;0	0;0;0	0;0;0	0;0;0	0;0;0	1;0;1	0;0;0	0;0;0	0;0;0	0;0;0
groupXV	1904000	1909500	1;2;	1;65;	1;0;1	0;0;0	0;0;0	0;0;0	0;0;0	0;0;0	0;0;0	0;0;0	1;0;1	0;0;0	0;0;0	0;0;0
groupXV	1914500	1919500	2;3;	64;2;	2;0;2	0;0;0	0;0;0	0;0;0	0;0;0	1;0;1	0;0;0	1;0;1	0;0;0	0;0;0	0;0;0	0;0;0
groupXV	2590500	2621500	2;3;	65;1;	1;0;1	0;0;0	0;0;0	0;0;0	0;0;0	0;0;0	0;0;0	0;0;0	1;0;1	0;0;0	0;0;0	0;0;0
groupXV	3149000	3153500	1;2;	1;65;	1;0;1	0;0;0	0;0;0	0;0;0	1;0;1	0;0;0	0;0;0	0;0;0	0;0;0	0;0;0	0;0;0	0;0;0
groupXV	5020500	5021500	0;1;2;	2;5;59;	9;2;5	0;0;0	0;0;0	6;1;4	0;0;0	2;1;0	0;0;0	0;0;0	0;0;0	0;0;0	1;0;1	0;0;0
groupXV	5257000	5263500	0;1;2;	3;4;59;	10;3;4	0;0;0	0;0;0	0;0;0	0;0;0	0;0;0	0;0;0	0;0;0	0;0;0	0;0;0	9;3;3	1;0;1
groupXV	5494000	5501000	2;3;	64;2;	2;0;2	0;0;0	0;0;0	0;0;0	0;0;0	2;0;2	0;0;0	0;0;0	0;0;0	0;0;0	0;0;0	0;0;0
groupXV	5579500	5594000	2;3;	65;1;	1;0;1	0;0;0	0;0;0	0;0;0	0;0;0	0;0;0	0;0;0	0;0;0	0;0;0	1;0;1	0;0;0	0;0;0
groupXV	5932500	5937000	1;2;	3;63;	3;0;3	0;0;0	0;0;0	0;0;0	0;0;0	0;0;0	0;0;0	3;0;3	0;0;0	0;0;0	0;0;0	0;0;0
groupXV	6037500	6044500	2;3;	65;1;	1;0;1	0;0;0	0;0;0	0;0;0	0;0;0	0;0;0	0;0;0	0;0;0	1;0;1	0;0;0	0;0;0	0;0;0
groupXV	7184000	7188500	1;2;	2;64;	2;0;2	0;0;0	0;0;0	0;0;0	2;0;2	0;0;0	0;0;0	0;0;0	0;0;0	0;0;0	0;0;0	0;0;0
groupXV	7368000	7372000	1;2;	1;65;	1;0;1	0;0;0	0;0;0	0;0;0	0;0;0	0;0;0	0;0;0	0;0;0	0;0;0	1;0;1	0;0;0	0;0;0

groupXV	7768000	7777500	2;3;	65;1;	1;0;1	0;0;0	0;0;0	0;0;0	0;0;0	0;0;0	0;0;0	0;0;0	0;0;0	0;0;0	1;0;1	0;0;0
groupXV	7889000	7899000	2;3;4;	54;7;5;	17;5;7	0;0;0	0;0;0	0;0;0	0;0;0	0;0;0	0;0;0	0;0;0	8;3;2	7;2;3	0;0;0	2;0;2
groupXV	8096500	8105000	2;3;	61;5;	5;0;5	0;0;0	0;0;0	0;0;0	0;0;0	0;0;0	0;0;0	0;0;0	0;0;0	0;0;0	4;0;4	1;0;1
groupXV	8483500	8494000	2;3;	65;1;	1;0;1	0;0;0	0;0;0	0;0;0	0;0;0	0;0;0	0;0;0	0;0;0	1;0;1	0;0;0	0;0;0	0;0;0
groupXV	8553500	8554500	0;1;2;	1;6;59;	8;1;6	0;0;0	0;0;0	6;1;4	1;0;1	1;0;1	0;0;0	0;0;0	0;0;0	0;0;0	0;0;0	0;0;0
groupXV	9811500	9820500	2;3;	62;4;	4;0;4	0;0;0	0;0;0	0;0;0	0;0;0	0;0;0	0;0;0	0;0;0	0;0;0	0;0;0	1;0;1	3;0;3
groupXV	10539000	10542000	1;2;	4;62;	4;0;4	0;0;0	0;0;0	3;0;3	0;0;0	1;0;1	0;0;0	0;0;0	0;0;0	0;0;0	0;0;0	0;0;0
groupXV	10652500	10653500	0;1;2;	1;7;58;	9;1;7	0;0;0	5;1;3	0;0;0	2;0;2	0;0;0	0;0;0	0;0;0	1;0;1	0;0;0	1;0;1	0;0;0
groupXV	10940000	10942000	1;2;	2;64;	2;0;2	0;0;0	1;0;1	0;0;0	1;0;1	0;0;0	0;0;0	0;0;0	0;0;0	0;0;0	0;0;0	0;0;0
groupXV	11803000	11808000	1;2;	1;65;	1;0;1	0;0;0	0;0;0	0;0;0	0;0;0	0;0;0	0;0;0	0;0;0	1;0;1	0;0;0	0;0;0	0;0;0
groupXV	11891000	11901000	2;3;	65;1;	1;0;1	0;0;0	0;0;0	0;0;0	0;0;0	0;0;0	0;0;0	1;0;1	0;0;0	0;0;0	0;0;0	0;0;0
groupXV	11964000	11968500	1;2;	1;65;	1;0;1	0;0;0	0;0;0	0;0;0	0;0;0	0;0;0	0;0;0	0;0;0	1;0;1	0;0;0	0;0;0	0;0;0
groupXV	12359500	12360500	0;1;2;	3;13;50;	19;3;13	0;0;0	0;0;0	0;0;0	0;0;0	0;0;0	0;0;0	0;0;0	4;0;4	1;0;1	7;1;5	7;2;3
groupXV	12614000	12619000	0;1;2;	5;2;59;	12;5;2	0;0;0	0;0;0	0;0;0	0;0;0	0;0;0	11;5;1	1;0;1	0;0;0	0;0;0	0;0;0	0;0;0
groupXV	12824500	12831500	1;2;	1;65;	1;0;1	0;0;0	0;0;0	0;0;0	0;0;0	0;0;0	0;0;0	0;0;0	1;0;1	0;0;0	0;0;0	0;0;0
groupXV	12852500	12857500	2;3;	65;1;	1;0;1	1;0;1	0;0;0	0;0;0	0;0;0	0;0;0	0;0;0	0;0;0	0;0;0	0;0;0	0;0;0	0;0;0
groupXV	13461000	13462500	0;1;2;	2;6;58;	10;2;6	1;0;1	0;0;0	7;2;3	0;0;0	2;0;2	0;0;0	0;0;0	0;0;0	0;0;0	0;0;0	0;0;0
groupXV	13614500	13619500	2;3;	65;1;	1;0;1	0;0;0	0;0;0	0;0;0	0;0;0	0;0;0	0;0;0	0;0;0	0;0;0	1;0;1	0;0;0	0;0;0
groupXV	13657500	13660000	0;1;2;	4;9;53;	17;4;9	0;0;0	0;0;0	0;0;0	0;0;0	0;0;0	0;0;0	0;0;0	3;0;3	4;0;4	10;4;2	0;0;0
groupXV	13680500	13683000	1;2;	3;63;	3;0;3	0;0;0	0;0;0	0;0;0	1;0;1	2;0;2	0;0;0	0;0;0	0;0;0	0;0;0	0;0;0	0;0;0
groupXV	13951500	13954500	1;2;	8;58;	8;0;8	0;0;0	2;0;2	0;0;0	1;0;1	0;0;0	0;0;0	0;0;0	0;0;0	1;0;1	0;0;0	4;0;4
groupXV	14034000	14037500	1;2;	1;65;	1;0;1	1;0;1	0;0;0	0;0;0	0;0;0	0;0;0	0;0;0	0;0;0	0;0;0	0;0;0	0;0;0	0;0;0
groupXV	14060000	14062000	0;1;2;	2;3;61;	7;2;3	0;0;0	5;2;1	0;0;0	2;0;2	0;0;0	0;0;0	0;0;0	0;0;0	0;0;0	0;0;0	0;0;0
groupXV	14237500	14239500	1;2;	2;64;	2;0;2	0;0;0	0;0;0	1;0;1	1;0;1	0;0;0	0;0;0	0;0;0	0;0;0	0;0;0	0;0;0	0;0;0
groupXV	14255500	14257500	1;2;	1;65;	1;0;1	0;0;0	0;0;0	0;0;0	0;0;0	0;0;0	0;0;0	1;0;1	0;0;0	0;0;0	0;0;0	0;0;0
groupXV	14346500	14351500	0;1;2;	2;8;56;	12;2;8	0;0;0	0;0;0	7;2;3	0;0;0	5;0;5	0;0;0	0;0;0	0;0;0	0;0;0	0;0;0	0;0;0
groupXV	14424000	14426500	1;2;	1;65;	1;0;1	1;0;1	0;0;0	0;0;0	0;0;0	0;0;0	0;0;0	0;0;0	0;0;0	0;0;0	0;0;0	0;0;0
groupXV	14468500	14473500	1;2;	1;65;	1;0;1	0;0;0	0;0;0	0;0;0	0;0;0	0;0;0	0;0;0	0;0;0	0;0;0	0;0;0	1;0;1	0;0;0
groupXV	14625000	14630500	1;2;	4;62;	4;0;4	0;0;0	0;0;0	0;0;0	0;0;0	0;0;0	0;0;0	0;0;0	0;0;0	4;0;4	0;0;0	0;0;0
groupXV	14711000	14714500	2;3;4;	44;20;2;	24;2;20	1;0;1	0;0;0	0;0;0	0;0;0	0;0;0	0;0;0	0;0;0	5;0;5	5;1;3	6;0;6	7;1;5
groupXV	14724000	14732500	1;2;	4;62;	4;0;4	0;0;0	0;0;0	2;0;2	0;0;0	2;0;2	0;0;0	0;0;0	0;0;0	0;0;0	0;0;0	0;0;0
groupXV	14775000	14776500	0;1;2;	3;17;46;	23;3;17	2;0;2	0;0;0	4;0;4	0;0;0	2;0;2	0;0;0	1;0;1	5;1;3	4;1;2	3;1;1	2;0;2
groupXV	14805500	14811500	1;2;	1;65;	1;0;1	1;0;1	0;0;0	0;0;0	0;0;0	0;0;0	0;0;0	0;0;0	0;0;0	0;0;0	0;0;0	0;0;0
groupXV	14966000	14968500	1;2;	1;65;	1;0;1	1;0;1	0;0;0	0;0;0	0;0;0	0;0;0	0;0;0	0;0;0	0;0;0	0;0;0	0;0;0	0;0;0

groupXV	15232000	15235000	0;1;2;	2;4;60;	8;2;4	0;0;0	0;0;0	2;0;2	0;0;0	0;0;0	0;0;0	0;0;0	4;1;2	2;1;0	0;0;0	0;0;0
groupXV	15631500	15635000	2;3;	58;8;	8;0;8	2;0;2	0;0;0	2;0;2	2;0;2	2;0;2	0;0;0	0;0;0	0;0;0	0;0;0	0;0;0	0;0;0
groupXV	15845000	15846500	1;2;	3;63;	3;0;3	0;0;0	0;0;0	2;0;2	0;0;0	1;0;1	0;0;0	0;0;0	0;0;0	0;0;0	0;0;0	0;0;0
groupXVI	17500	18500	1;2;	8;58;	8;0;8	0;0;0	0;0;0	2;0;2	2;0;2	0;0;0	0;0;0	4;0;4	0;0;0	0;0;0	0;0;0	0;0;0
groupXVI	39500	109500	2;3;	62;4;	4;0;4	2;0;2	0;0;0	2;0;2	0;0;0	0;0;0	0;0;0	0;0;0	0;0;0	0;0;0	0;0;0	0;0;0
groupXVI	219500	227000	2;3;	64;2;	2;0;2	0;0;0	0;0;0	2;0;2	0;0;0	0;0;0	0;0;0	0;0;0	0;0;0	0;0;0	0;0;0	0;0;0
groupXVI	243500	252500	2;3;	64;2;	2;0;2	0;0;0	0;0;0	0;0;0	0;0;0	0;0;0	0;0;0	0;0;0	0;0;0	0;0;0	0;0;0	2;0;2
groupXVI	343500	345500	2;3;	65;1;	1;0;1	0;0;0	0;0;0	0;0;0	0;0;0	0;0;0	0;0;0	0;0;0	0;0;0	0;0;0	0;0;0	1;0;1
groupXVI	347500	351000	1;2;	5;61;	5;0;5	1;0;1	0;0;0	0;0;0	0;0;0	0;0;0	0;0;0	1;0;1	2;0;2	1;0;1	0;0;0	0;0;0
groupXVI	354000	355000	0;1;2;	3;5;58;	11;3;5	0;0;0	0;0;0	0;0;0	0;0;0	0;0;0	0;0;0	0;0;0	0;0;0	0;0;0	2;0;2	9;3;3
groupXVI	377500	379000	1;2;	2;64;	2;0;2	0;0;0	2;0;2	0;0;0	0;0;0	0;0;0	0;0;0	0;0;0	0;0;0	0;0;0	0;0;0	0;0;0
groupXVI	474500	486500	2;3;	59;7;	7;0;7	0;0;0	0;0;0	0;0;0	0;0;0	0;0;0	0;0;0	0;0;0	2;0;2	2;0;2	1;0;1	2;0;2
groupXVI	588000	596000	2;3;	65;1;	1;0;1	0;0;0	0;0;0	0;0;0	0;0;0	0;0;0	0;0;0	0;0;0	0;0;0	1;0;1	0;0;0	0;0;0
groupXVI	807500	815000	2;3;	65;1;	1;0;1	0;0;0	0;0;0	0;0;0	1;0;1	0;0;0	0;0;0	0;0;0	0;0;0	0;0;0	0;0;0	0;0;0
groupXVI	919000	920500	0;1;2;	2;7;57;	11;2;7	0;0;0	0;0;0	0;0;0	0;0;0	0;0;0	0;0;0	1;0;1	4;1;2	5;1;3	0;0;0	1;0;1
groupXVI	950000	961000	2;3;4;	58;7;1;	9;1;7	0;0;0	0;0;0	0;0;0	0;0;0	0;0;0	7;1;5	2;0;2	0;0;0	0;0;0	0;0;0	0;0;0
groupXVI	972500	982500	1;2;	9;57;	9;0;9	1;0;1	3;0;3	0;0;0	1;0;1	0;0;0	3;0;3	1;0;1	0;0;0	0;0;0	0;0;0	0;0;0
groupXVI	994000	999500	1;2;	1;65;	1;0;1	1;0;1	0;0;0	0;0;0	0;0;0	0;0;0	0;0;0	0;0;0	0;0;0	0;0;0	0;0;0	0;0;0
groupXVI	1007000	1009500	1;2;	2;64;	2;0;2	0;0;0	1;0;1	0;0;0	1;0;1	0;0;0	0;0;0	0;0;0	0;0;0	0;0;0	0;0;0	0;0;0
groupXVI	1205500	1209000	1;2;	4;62;	4;0;4	0;0;0	0;0;0	4;0;4	0;0;0	0;0;0	0;0;0	0;0;0	0;0;0	0;0;0	0;0;0	0;0;0
groupXVI	1246000	1249500	0;1;2;	11;4;51;	26;11;4	0;0;0	0;0;0	0;0;0	0;0;0	0;0;0	0;0;0	0;0;0	2;0;2	1;0;1	12;6;0	11;5;1
groupXVI	1250000	1256500	1;2;	2;64;	2;0;2	0;0;0	0;0;0	0;0;0	0;0;0	0;0;0	0;0;0	0;0;0	1;0;1	1;0;1	0;0;0	0;0;0
groupXVI	1264000	1290000	1;2;	2;64;	2;0;2	1;0;1	0;0;0	1;0;1	0;0;0	0;0;0	0;0;0	0;0;0	0;0;0	0;0;0	0;0;0	0;0;0
groupXVI	1294500	1301500	1;2;	12;54;	12;0;12	0;0;0	0;0;0	1;0;1	0;0;0	0;0;0	0;0;0	0;0;0	3;0;3	2;0;2	5;0;5	1;0;1
groupXVI	1446500	1447500	0;1;2;	1;9;56;	11;1;9	0;0;0	1;0;1	7;1;5	3;0;3	0;0;0	0;0;0	0;0;0	0;0;0	0;0;0	0;0;0	0;0;0
groupXVI	1887000	1890500	1;2;	6;60;	6;0;6	0;0;0	1;0;1	0;0;0	3;0;3	0;0;0	0;0;0	2;0;2	0;0;0	0;0;0	0;0;0	0;0;0
groupXVI	1971500	2015500	2;3;	65;1;	1;0;1	1;0;1	0;0;0	0;0;0	0;0;0	0;0;0	0;0;0	0;0;0	0;0;0	0;0;0	0;0;0	0;0;0
groupXVI	2080000	2085500	2;3;	64;2;	2;0;2	0;0;0	0;0;0	0;0;0	0;0;0	0;0;0	0;0;0	0;0;0	0;0;0	0;0;0	2;0;2	0;0;0
groupXVI	2254000	2260500	2;3;	65;1;	1;0;1	0;0;0	0;0;0	0;0;0	0;0;0	0;0;0	0;0;0	0;0;0	0;0;0	0;0;0	0;0;0	1;0;1
groupXVI	2322000	2335500	1;2;	1;65;	1;0;1	0;0;0	0;0;0	0;0;0	0;0;0	0;0;0	0;0;0	0;0;0	0;0;0	1;0;1	0;0;0	0;0;0
groupXVI	2384500	2393000	1;2;	2;64;	2;0;2	0;0;0	0;0;0	0;0;0	0;0;0	0;0;0	2;0;2	0;0;0	0;0;0	0;0;0	0;0;0	0;0;0
groupXVI	2514000	2515500	0;1;2;	1;8;57;	10;1;8	0;0;0	0;0;0	0;0;0	0;0;0	0;0;0	5;0;5	5;1;3	0;0;0	0;0;0	0;0;0	0;0;0
groupXVI	2814000	2817000	1;2;	1;65;	1;0;1	0;0;0	0;0;0	0;0;0	1;0;1	0;0;0	0;0;0	0;0;0	0;0;0	0;0;0	0;0;0	0;0;0
groupXVI	3197500	3206000	2;3;4;	63;2;1;	4;1;2	0;0;0	0;0;0	0;0;0	0;0;0	0;0;0	4;1;2	0;0;0	0;0;0	0;0;0	0;0;0	0;0;0

groupXVI	3779500	3783000	1;2;	1;65;	1;0;1	0;0;0	0;0;0	0;0;0	0;0;0	0;0;0	0;0;0	0;0;0	0;0;0	1;0;1	0;0;0	0;0;0
groupXVI	3857000	3866000	2;3;	65;1;	1;0;1	0;0;0	0;0;0	0;0;0	1;0;1	0;0;0	0;0;0	0;0;0	0;0;0	0;0;0	0;0;0	0;0;0
groupXVI	4213000	4219500	2;3;4;	51;8;7;	22;7;8	9;3;3	2;0;2	5;2;1	2;1;0	3;1;1	0;0;0	1;0;1	0;0;0	0;0;0	0;0;0	0;0;0
groupXVI	4268500	4319500	0;1;2;	1;7;58;	9;1;7	0;0;0	0;0;0	0;0;0	0;0;0	0;0;0	0;0;0	1;0;1	1;0;1	0;0;0	3;1;1	4;0;4
groupXVI	4305500	4328000	1;2;	7;59;	7;0;7	0;0;0	0;0;0	0;0;0	0;0;0	0;0;0	0;0;0	1;0;1	0;0;0	0;0;0	2;0;2	4;0;4
groupXVI	4608500	4612000	1;2;	1;65;	1;0;1	0;0;0	0;0;0	0;0;0	0;0;0	0;0;0	0;0;0	0;0;0	0;0;0	1;0;1	0;0;0	0;0;0
groupXVI	4872500	4875500	1;2;	2;64;	2;0;2	0;0;0	0;0;0	2;0;2	0;0;0	0;0;0	0;0;0	0;0;0	0;0;0	0;0;0	0;0;0	0;0;0
groupXVI	4887000	4888500	0;1;2;	3;3;60;	9;3;3	0;0;0	0;0;0	0;0;0	0;0;0	0;0;0	0;0;0	0;0;0	0;0;0	0;0;0	9;3;3	0;0;0
groupXVI	5322500	5324500	1;2;	2;64;	2;0;2	0;0;0	2;0;2	0;0;0	0;0;0	0;0;0	0;0;0	0;0;0	0;0;0	0;0;0	0;0;0	0;0;0
groupXVI	5395500	5397000	1;2;	1;65;	1;0;1	1;0;1	0;0;0	0;0;0	0;0;0	0;0;0	0;0;0	0;0;0	0;0;0	0;0;0	0;0;0	0;0;0
groupXVI	5457000	5461000	1;2;	2;64;	2;0;2	0;0;0	2;0;2	0;0;0	0;0;0	0;0;0	0;0;0	0;0;0	0;0;0	0;0;0	0;0;0	0;0;0
groupXVI	5723500	5726500	1;2;	1;65;	1;0;1	0;0;0	0;0;0	0;0;0	0;0;0	0;0;0	0;0;0	0;0;0	0;0;0	0;0;0	0;0;0	1;0;1
groupXVI	5801000	5808500	2;3;	64;2;	2;0;2	0;0;0	0;0;0	0;0;0	0;0;0	0;0;0	0;0;0	2;0;2	0;0;0	0;0;0	0;0;0	0;0;0
groupXVI	6082500	6084500	0;1;2;	1;2;63;	4;1;2	0;0;0	0;0;0	0;0;0	3;1;1	1;0;1	0;0;0	0;0;0	0;0;0	0;0;0	0;0;0	0;0;0
groupXVI	7121500	7123500	2;3;	65;1;	1;0;1	0;0;0	0;0;0	0;0;0	0;0;0	1;0;1	0;0;0	0;0;0	0;0;0	0;0;0	0;0;0	0;0;0
groupXVI	7684500	7690000	2;3;	65;1;	1;0;1	0;0;0	0;0;0	0;0;0	0;0;0	0;0;0	0;0;0	0;0;0	0;0;0	1;0;1	0;0;0	0;0;0
groupXVI	8136500	8140000	2;3;	64;2;	2;0;2	0;0;0	0;0;0	0;0;0	2;0;2	0;0;0	0;0;0	0;0;0	0;0;0	0;0;0	0;0;0	0;0;0
groupXVI	8169500	8178500	2;3;	65;1;	1;0;1	0;0;0	0;0;0	1;0;1	0;0;0	0;0;0	0;0;0	0;0;0	0;0;0	0;0;0	0;0;0	0;0;0
groupXVI	8306500	8324000	2;3;	60;6;	6;0;6	1;0;1	0;0;0	0;0;0	0;0;0	0;0;0	0;0;0	2;0;2	0;0;0	0;0;0	1;0;1	2;0;2
groupXVI	8396000	8400500	2;3;	64;2;	2;0;2	0;0;0	0;0;0	2;0;2	0;0;0	0;0;0	0;0;0	0;0;0	0;0;0	0;0;0	0;0;0	0;0;0
groupXVI	9299500	9300500	0;1;2;	4;9;53;	17;4;9	0;0;0	0;0;0	0;0;0	0;0;0	0;0;0	0;0;0	0;0;0	2;0;2	0;0;0	5;0;5	10;4;2
groupXVI	9395500	9397000	0;1;2;	1;3;62;	5;1;3	0;0;0	0;0;0	0;0;0	5;1;3	0;0;0	0;0;0	0;0;0	0;0;0	0;0;0	0;0;0	0;0;0
groupXVI	11059000	11061500	1;2;	1;65;	1;0;1	1;0;1	0;0;0	0;0;0	0;0;0	0;0;0	0;0;0	0;0;0	0;0;0	0;0;0	0;0;0	0;0;0
groupXVI	11179000	11180500	2;3;	64;2;	2;0;2	0;0;0	1;0;1	0;0;0	0;0;0	0;0;0	1;0;1	0;0;0	0;0;0	0;0;0	0;0;0	0;0;0
groupXVI	11804000	11853000	1;2;	5;61;	5;0;5	4;0;4	0;0;0	0;0;0	0;0;0	0;0;0	0;0;0	1;0;1	0;0;0	0;0;0	0;0;0	0;0;0
groupXVI	12139000	12148000	1;2;	13;53;	13;0;13	0;0;0	0;0;0	0;0;0	0;0;0	0;0;0	0;0;0	0;0;0	3;0;3	2;0;2	5;0;5	3;0;3
groupXVI	12592000	12593000	0;1;2;	1;5;60;	7;1;5	0;0;0	0;0;0	5;1;3	0;0;0	2;0;2	0;0;0	0;0;0	0;0;0	0;0;0	0;0;0	0;0;0
groupXVI	12639000	12642500	1;2;	2;64;	2;0;2	0;0;0	0;0;0	0;0;0	0;0;0	0;0;0	0;0;0	0;0;0	0;0;0	0;0;0	2;0;2	0;0;0
groupXVI	13050500	13053500	1;2;	1;65;	1;0;1	0;0;0	0;0;0	0;0;0	0;0;0	0;0;0	0;0;0	0;0;0	1;0;1	0;0;0	0;0;0	0;0;0
groupXVI	13278500	13280500	0;1;2;	1;4;61;	6;1;4	0;0;0	0;0;0	4;1;2	0;0;0	2;0;2	0;0;0	0;0;0	0;0;0	0;0;0	0;0;0	0;0;0
groupXVI	13299500	13302500	1;2;	1;65;	1;0;1	0;0;0	0;0;0	0;0;0	0;0;0	1;0;1	0;0;0	0;0;0	0;0;0	0;0;0	0;0;0	0;0;0
groupXVI	13749000	13752500	1;2;	3;63;	3;0;3	0;0;0	0;0;0	0;0;0	0;0;0	0;0;0	0;0;0	0;0;0	0;0;0	2;0;2	1;0;1	0;0;0
groupXVI	13796000	13798000	2;3;	61;5;	5;0;5	1;0;1	0;0;0	0;0;0	0;0;0	0;0;0	0;0;0	0;0;0	2;0;2	1;0;1	0;0;0	1;0;1
groupXVI	13849000	13850000	0;1;2;	2;3;61;	7;2;3	0;0;0	0;0;0	0;0;0	7;2;3	0;0;0	0;0;0	0;0;0	0;0;0	0;0;0	0;0;0	0;0;0

groupXVI	13861000	13888500	2;3;	50;16;	16;0;16	0;0;0	0;0;0	0;0;0	0;0;0	0;0;0	0;0;0	0;0;0	3;0;3	3;0;3	5;0;5	5;0;5
groupXVI	13931000	13932000	0;1;2;	1;3;62;	5;1;3	0;0;0	0;0;0	0;0;0	5;1;3	0;0;0	0;0;0	0;0;0	0;0;0	0;0;0	0;0;0	0;0;0
groupXVI	13965500	13967000	0;1;2;	1;5;60;	7;1;5	0;0;0	0;0;0	0;0;0	0;0;0	0;0;0	6;1;4	1;0;1	0;0;0	0;0;0	0;0;0	0;0;0
groupXVI	14517500	14519500	1;2;	3;63;	3;0;3	0;0;0	0;0;0	0;0;0	0;0;0	0;0;0	0;0;0	0;0;0	2;0;2	1;0;1	0;0;0	0;0;0
groupXVI	14525000	14526000	1;2;	12;54;	12;0;12	2;0;2	1;0;1	4;0;4	3;0;3	2;0;2	0;0;0	0;0;0	0;0;0	0;0;0	0;0;0	0;0;0
groupXVI	15074500	15076500	1;2;	1;65;	1;0;1	0;0;0	0;0;0	0;0;0	0;0;0	0;0;0	0;0;0	1;0;1	0;0;0	0;0;0	0;0;0	0;0;0
groupXVI	15121500	15123000	2;3;	60;6;	6;0;6	0;0;0	0;0;0	1;0;1	1;0;1	1;0;1	1;0;1	0;0;0	0;0;0	1;0;1	1;0;1	0;0;0
groupXVI	16137000	16142500	1;2;	1;65;	1;0;1	1;0;1	0;0;0	0;0;0	0;0;0	0;0;0	0;0;0	0;0;0	0;0;0	0;0;0	0;0;0	0;0;0
groupXVI	16456500	16485500	2;3;4;	53;10;3;	16;3;10	5;1;3	0;0;0	2;1;0	2;0;2	7;1;5	0;0;0	0;0;0	0;0;0	0;0;0	0;0;0	0;0;0
groupXVI	16558000	16561500	2;3;	45;21;	21;0;21	0;0;0	0;0;0	0;0;0	0;0;0	0;0;0	0;0;0	0;0;0	6;0;6	3;0;3	6;0;6	6;0;6
groupXVI	16658000	16662500	2;3;	62;4;	4;0;4	0;0;0	0;0;0	0;0;0	0;0;0	0;0;0	0;0;0	0;0;0	1;0;1	0;0;0	1;0;1	2;0;2
groupXVI	16667000	16674000	2;3;	52;14;	14;0;14	0;0;0	0;0;0	0;0;0	0;0;0	0;0;0	0;0;0	0;0;0	5;0;5	2;0;2	6;0;6	1;0;1
groupXVI	16819000	16824000	2;3;	64;2;	2;0;2	0;0;0	0;0;0	0;0;0	0;0;0	0;0;0	0;0;0	0;0;0	0;0;0	0;0;0	1;0;1	1;0;1
groupXVI	16837000	16839500	1;2;	1;65;	1;0;1	0;0;0	0;0;0	0;0;0	0;0;0	0;0;0	0;0;0	0;0;0	0;0;0	0;0;0	0;0;0	1;0;1
groupXVI	17203000	17207000	1;2;	2;64;	2;0;2	1;0;1	0;0;0	0;0;0	0;0;0	0;0;0	0;0;0	0;0;0	0;0;0	1;0;1	0;0;0	0;0;0
groupXVI	17297000	17302000	1;2;	1;65;	1;0;1	1;0;1	0;0;0	0;0;0	0;0;0	0;0;0	0;0;0	0;0;0	0;0;0	0;0;0	0;0;0	0;0;0
groupXVI	17477500	17480000	2;3;	61;5;	5;0;5	0;0;0	0;0;0	1;0;1	0;0;0	0;0;0	0;0;0	0;0;0	0;0;0	3;0;3	0;0;0	1;0;1
groupXVI	17729500	17731000	0;1;2;	3;7;56;	13;3;7	3;0;3	0;0;0	0;0;0	0;0;0	0;0;0	8;3;2	1;0;1	0;0;0	1;0;1	0;0;0	0;0;0
groupXVII	76000	112000	2;3;	64;2;	2;0;2	0;0;0	0;0;0	0;0;0	0;0;0	1;0;1	1;0;1	0;0;0	0;0;0	0;0;0	0;0;0	0;0;0
groupXVII	158000	160500	1;2;	1;65;	1;0;1	0;0;0	0;0;0	0;0;0	0;0;0	0;0;0	0;0;0	0;0;0	1;0;1	0;0;0	0;0;0	0;0;0
groupXVII	212000	214500	1;2;	1;65;	1;0;1	1;0;1	0;0;0	0;0;0	0;0;0	0;0;0	0;0;0	0;0;0	0;0;0	0;0;0	0;0;0	0;0;0
groupXVII	355000	361000	1;2;	1;65;	1;0;1	0;0;0	0;0;0	0;0;0	0;0;0	0;0;0	0;0;0	0;0;0	0;0;0	0;0;0	0;0;0	1;0;1
groupXVII	564000	567500	1;2;	1;65;	1;0;1	0;0;0	1;0;1	0;0;0	0;0;0	0;0;0	0;0;0	0;0;0	0;0;0	0;0;0	0;0;0	0;0;0
groupXVII	579500	582500	2;3;	64;2;	2;0;2	0;0;0	0;0;0	0;0;0	0;0;0	2;0;2	0;0;0	0;0;0	0;0;0	0;0;0	0;0;0	0;0;0
groupXVII	1309000	1324000	2;3;	65;1;	1;0;1	1;0;1	0;0;0	0;0;0	0;0;0	0;0;0	0;0;0	0;0;0	0;0;0	0;0;0	0;0;0	0;0;0
groupXVII	1352000	1354500	1;2;	3;63;	3;0;3	0;0;0	0;0;0	0;0;0	0;0;0	0;0;0	0;0;0	0;0;0	0;0;0	0;0;0	3;0;3	0;0;0
groupXVII	2376000	2378000	0;1;2;	2;4;60;	8;2;4	0;0;0	0;0;0	0;0;0	0;0;0	0;0;0	0;0;0	0;0;0	0;0;0	0;0;0	1;0;1	7;2;3
groupXVII	2392000	2403000	2;3;	65;1;	1;0;1	0;0;0	0;0;0	0;0;0	0;0;0	0;0;0	0;0;0	1;0;1	0;0;0	0;0;0	0;0;0	0;0;0
groupXVII	3133000	3137000	1;2;	1;65;	1;0;1	0;0;0	0;0;0	0;0;0	1;0;1	0;0;0	0;0;0	0;0;0	0;0;0	0;0;0	0;0;0	0;0;0
groupXVII	3473500	3483500	2;3;	65;1;	1;0;1	1;0;1	0;0;0	0;0;0	0;0;0	0;0;0	0;0;0	0;0;0	0;0;0	0;0;0	0;0;0	0;0;0
groupXVII	3730000	3735000	2;3;	61;5;	5;0;5	0;0;0	5;0;5	0;0;0	0;0;0	0;0;0	0;0;0	0;0;0	0;0;0	0;0;0	0;0;0	0;0;0
groupXVII	5727000	5729500	1;2;	2;64;	2;0;2	0;0;0	0;0;0	0;0;0	2;0;2	0;0;0	0;0;0	0;0;0	0;0;0	0;0;0	0;0;0	0;0;0
groupXVII	6519000	6522000	0;1;2;	3;7;56;	13;3;7	1;0;1	0;0;0	0;0;0	0;0;0	0;0;0	6;2;2	6;1;4	0;0;0	0;0;0	0;0;0	0;0;0
groupXVII	6573000	6588000	1;2;	2;64;	2;0;2	0;0;0	0;0;0	1;0;1	1;0;1	0;0;0	0;0;0	0;0;0	0;0;0	0;0;0	0;0;0	0;0;0

groupXVII	7043500	7046000	0;1;2;	1;3;62;	5;1;3	0;0;0	0;0;0	0;0;0	0;0;0	0;0;0	4;1;2	1;0;1	0;0;0	0;0;0	0;0;0	0;0;0
groupXVII	7070000	7075500	1;2;	2;64;	2;0;2	0;0;0	0;0;0	1;0;1	0;0;0	1;0;1	0;0;0	0;0;0	0;0;0	0;0;0	0;0;0	0;0;0
groupXVII	7298000	7303500	1;2;	7;59;	7;0;7	0;0;0	2;0;2	0;0;0	2;0;2	0;0;0	0;0;0	0;0;0	3;0;3	0;0;0	0;0;0	0;0;0
groupXVII	7475000	7479500	1;2;	3;63;	3;0;3	1;0;1	0;0;0	0;0;0	0;0;0	0;0;0	0;0;0	0;0;0	0;0;0	2;0;2	0;0;0	0;0;0
groupXVII	7480000	7488500	1;2;	1;65;	1;0;1	0;0;0	0;0;0	0;0;0	0;0;0	0;0;0	0;0;0	0;0;0	1;0;1	0;0;0	0;0;0	0;0;0
groupXVII	7729000	7735000	2;3;	65;1;	1;0;1	0;0;0	0;0;0	0;0;0	0;0;0	0;0;0	0;0;0	1;0;1	0;0;0	0;0;0	0;0;0	0;0;0
groupXVII	8347000	8353000	1;2;	1;65;	1;0;1	0;0;0	0;0;0	0;0;0	0;0;0	0;0;0	0;0;0	0;0;0	0;0;0	0;0;0	1;0;1	0;0;0
groupXVII	8435000	8442500	2;3;	65;1;	1;0;1	0;0;0	0;0;0	0;0;0	0;0;0	0;0;0	0;0;0	1;0;1	0;0;0	0;0;0	0;0;0	0;0;0
groupXVII	8582500	8617000	2;3;	65;1;	1;0;1	0;0;0	0;0;0	0;0;0	0;0;0	0;0;0	0;0;0	0;0;0	0;0;0	0;0;0	0;0;0	1;0;1
groupXVII	8739500	8745000	2;3;	65;1;	1;0;1	0;0;0	0;0;0	0;0;0	1;0;1	0;0;0	0;0;0	0;0;0	0;0;0	0;0;0	0;0;0	0;0;0
groupXVII	8808000	8818000	2;3;4;	53;10;3;	16;3;10	0;0;0	0;0;0	0;0;0	0;0;0	0;0;0	0;0;0	3;0;3	3;0;3	1;0;1	7;3;1	2;0;2
groupXVII	9225500	9229500	2;3;	64;2;	2;0;2	0;0;0	0;0;0	0;0;0	0;0;0	2;0;2	0;0;0	0;0;0	0;0;0	0;0;0	0;0;0	0;0;0
groupXVII	9243000	9245000	1;2;	3;63;	3;0;3	0;0;0	0;0;0	0;0;0	2;0;2	0;0;0	0;0;0	0;0;0	1;0;1	0;0;0	0;0;0	0;0;0
groupXVII	9245500	9248500	1;2;	2;64;	2;0;2	2;0;2	0;0;0	0;0;0	0;0;0	0;0;0	0;0;0	0;0;0	0;0;0	0;0;0	0;0;0	0;0;0
groupXVII	9857000	9860000	1;2;	1;65;	1;0;1	0;0;0	0;0;0	0;0;0	1;0;1	0;0;0	0;0;0	0;0;0	0;0;0	0;0;0	0;0;0	0;0;0
groupXVII	11009500	11012000	0;1;2;	1;5;60;	7;1;5	0;0;0	0;0;0	0;0;0	0;0;0	0;0;0	6;1;4	1;0;1	0;0;0	0;0;0	0;0;0	0;0;0
groupXVII	11083500	11088000	1;2;	1;65;	1;0;1	0;0;0	0;0;0	0;0;0	0;0;0	1;0;1	0;0;0	0;0;0	0;0;0	0;0;0	0;0;0	0;0;0
groupXVII	11374500	11382000	0;1;2;	2;1;63;	5;2;1	0;0;0	0;0;0	5;2;1	0;0;0	0;0;0	0;0;0	0;0;0	0;0;0	0;0;0	0;0;0	0;0;0
groupXVII	11501500	11503000	0;1;2;	8;5;53;	21;8;5	0;0;0	0;0;0	0;0;0	0;0;0	0;0;0	0;0;0	0;0;0	1;0;1	2;0;2	12;6;0	6;2;2
groupXVII	11598000	11601000	1;2;	2;64;	2;0;2	0;0;0	1;0;1	0;0;0	1;0;1	0;0;0	0;0;0	0;0;0	0;0;0	0;0;0	0;0;0	0;0;0
groupXVII	11676500	11678000	1;2;	5;61;	5;0;5	0;0;0	0;0;0	0;0;0	0;0;0	0;0;0	5;0;5	0;0;0	0;0;0	0;0;0	0;0;0	0;0;0
groupXVII	11731000	11739500	2;3;	65;1;	1;0;1	0;0;0	0;0;0	1;0;1	0;0;0	0;0;0	0;0;0	0;0;0	0;0;0	0;0;0	0;0;0	0;0;0
groupXVII	12163000	12173000	2;3;	65;1;	1;0;1	1;0;1	0;0;0	0;0;0	0;0;0	0;0;0	0;0;0	0;0;0	0;0;0	0;0;0	0;0;0	0;0;0
groupXVII	12203500	12205500	1;2;	7;59;	7;0;7	0;0;0	0;0;0	0;0;0	0;0;0	0;0;0	0;0;0	0;0;0	1;0;1	0;0;0	1;0;1	5;0;5
groupXVII	12323000	12325500	1;2;	3;63;	3;0;3	0;0;0	0;0;0	0;0;0	0;0;0	0;0;0	0;0;0	0;0;0	1;0;1	0;0;0	1;0;1	1;0;1
groupXVII	12429000	12433500	2;3;	64;2;	2;0;2	0;0;0	0;0;0	0;0;0	1;0;1	0;0;0	0;0;0	1;0;1	0;0;0	0;0;0	0;0;0	0;0;0
groupXVII	12583000	12587500	1;2;	1;65;	1;0;1	0;0;0	0;0;0	0;0;0	0;0;0	0;0;0	0;0;0	0;0;0	1;0;1	0;0;0	0;0;0	0;0;0
groupXVII	12838000	12840000	0;1;2;	1;11;54;	13;1;11	1;0;1	1;0;1	5;1;3	3;0;3	3;0;3	0;0;0	0;0;0	0;0;0	0;0;0	0;0;0	0;0;0
groupXVII	13273000	13276500	1;2;	4;62;	4;0;4	0;0;0	0;0;0	0;0;0	0;0;0	0;0;0	4;0;4	0;0;0	0;0;0	0;0;0	0;0;0	0;0;0
groupXVII	13365500	13368000	1;2;	2;64;	2;0;2	0;0;0	0;0;0	0;0;0	2;0;2	0;0;0	0;0;0	0;0;0	0;0;0	0;0;0	0;0;0	0;0;0
groupXVII	13837500	13841500	0;2;	1;65;	2;1;0	2;1;0	0;0;0	0;0;0	0;0;0	0;0;0	0;0;0	0;0;0	0;0;0	0;0;0	0;0;0	0;0;0
groupXVIII	356000	358000	1;2;	16;50;	16;0;16	0;0;0	0;0;0	0;0;0	0;0;0	0;0;0	0;0;0	0;0;0	6;0;6	2;0;2	6;0;6	2;0;2
groupXVIII	1053500	1060500	2;3;	64;2;	2;0;2	0;0;0	0;0;0	0;0;0	1;0;1	1;0;1	0;0;0	0;0;0	0;0;0	0;0;0	0;0;0	0;0;0
groupXVIII	1381500	1390000	2;3;	65;1;	1;0;1	0;0;0	0;0;0	0;0;0	0;0;0	0;0;0	0;0;0	0;0;0	0;0;0	0;0;0	0;0;0	1;0;1

groupXVIII	1716500	1722000	2;3;	64;2;	2;0;2	0;0;0	0;0;0	0;0;0	0;0;0	2;0;2	0;0;0	0;0;0	0;0;0	0;0;0	0;0;0	0;0;0
groupXVIII	1804500	1806500	1;2;	1;65;	1;0;1	1;0;1	0;0;0	0;0;0	0;0;0	0;0;0	0;0;0	0;0;0	0;0;0	0;0;0	0;0;0	0;0;0
groupXVIII	1904000	1907000	1;2;	1;65;	1;0;1	0;0;0	0;0;0	0;0;0	1;0;1	0;0;0	0;0;0	0;0;0	0;0;0	0;0;0	0;0;0	0;0;0
groupXVIII	2831000	2833500	1;2;	1;65;	1;0;1	0;0;0	0;0;0	0;0;0	0;0;0	0;0;0	0;0;0	0;0;0	0;0;0	0;0;0	0;0;0	1;0;1
groupXVIII	2893000	2894000	0;2;	1;65;	2;1;0	0;0;0	0;0;0	0;0;0	0;0;0	0;0;0	0;0;0	0;0;0	0;0;0	2;1;0	0;0;0	0;0;0
groupXVIII	3092500	3101500	2;3;	65;1;	1;0;1	1;0;1	0;0;0	0;0;0	0;0;0	0;0;0	0;0;0	0;0;0	0;0;0	0;0;0	0;0;0	0;0;0
groupXVIII	3117000	3128500	2;3;4;	64;1;1;	3;1;1	2;1;0	0;0;0	0;0;0	1;0;1	0;0;0	0;0;0	0;0;0	0;0;0	0;0;0	0;0;0	0;0;0
groupXVIII	3512500	3523000	1;2;	1;65;	1;0;1	1;0;1	0;0;0	0;0;0	0;0;0	0;0;0	0;0;0	0;0;0	0;0;0	0;0;0	0;0;0	0;0;0
groupXVIII	3534500	3544500	2;3;	65;1;	1;0;1	0;0;0	0;0;0	0;0;0	0;0;0	0;0;0	0;0;0	0;0;0	1;0;1	0;0;0	0;0;0	0;0;0
groupXVIII	3751000	3752000	0;1;2;	2;6;58;	10;2;6	0;0;0	0;0;0	0;0;0	0;0;0	0;0;0	8;2;4	2;0;2	0;0;0	0;0;0	0;0;0	0;0;0
groupXVIII	3813000	3818000	1;2;	2;64;	2;0;2	1;0;1	0;0;0	0;0;0	0;0;0	0;0;0	0;0;0	1;0;1	0;0;0	0;0;0	0;0;0	0;0;0
groupXVIII	4128500	4136000	2;3;	65;1;	1;0;1	1;0;1	0;0;0	0;0;0	0;0;0	0;0;0	0;0;0	0;0;0	0;0;0	0;0;0	0;0;0	0;0;0
groupXVIII	4167500	4171500	1;2;	2;64;	2;0;2	0;0;0	0;0;0	0;0;0	0;0;0	0;0;0	0;0;0	2;0;2	0;0;0	0;0;0	0;0;0	0;0;0
groupXVIII	4347500	4351000	0;1;2;	2;6;58;	10;2;6	0;0;0	0;0;0	0;0;0	0;0;0	0;0;0	8;2;4	2;0;2	0;0;0	0;0;0	0;0;0	0;0;0
groupXVIII	4585000	4588000	1;2;	1;65;	1;0;1	0;0;0	0;0;0	0;0;0	1;0;1	0;0;0	0;0;0	0;0;0	0;0;0	0;0;0	0;0;0	0;0;0
groupXVIII	4788000	4795000	2;3;	65;1;	1;0;1	1;0;1	0;0;0	0;0;0	0;0;0	0;0;0	0;0;0	0;0;0	0;0;0	0;0;0	0;0;0	0;0;0
groupXVIII	4823500	4844500	2;3;	64;2;	2;0;2	0;0;0	0;0;0	0;0;0	0;0;0	0;0;0	0;0;0	2;0;2	0;0;0	0;0;0	0;0;0	0;0;0
groupXVIII	5051000	5055000	1;2;	4;62;	4;0;4	0;0;0	0;0;0	0;0;0	0;0;0	0;0;0	0;0;0	0;0;0	3;0;3	1;0;1	0;0;0	0;0;0
groupXVIII	5056500	5057500	0;1;2;	1;6;59;	8;1;6	0;0;0	2;0;2	0;0;0	6;1;4	0;0;0	0;0;0	0;0;0	0;0;0	0;0;0	0;0;0	0;0;0
groupXVIII	5166000	5168500	0;1;2;	1;3;62;	5;1;3	1;0;1	0;0;0	3;1;1	0;0;0	1;0;1	0;0;0	0;0;0	0;0;0	0;0;0	0;0;0	0;0;0
groupXVIII	5239500	5242500	0;1;2;	7;6;53;	20;7;6	1;0;1	0;0;0	1;0;1	2;1;0	0;0;0	11;5;1	5;1;3	0;0;0	0;0;0	0;0;0	0;0;0
groupXVIII	5642000	5643000	0;1;2;	1;5;60;	7;1;5	0;0;0	0;0;0	1;0;1	0;0;0	4;1;2	0;0;0	0;0;0	0;0;0	0;0;0	2;0;2	0;0;0
groupXVIII	5755000	5766000	2;3;	65;1;	1;0;1	0;0;0	0;0;0	0;0;0	1;0;1	0;0;0	0;0;0	0;0;0	0;0;0	0;0;0	0;0;0	0;0;0
groupXVIII	5917000	5919000	1;2;	1;65;	1;0;1	0;0;0	0;0;0	1;0;1	0;0;0	0;0;0	0;0;0	0;0;0	0;0;0	0;0;0	0;0;0	0;0;0
groupXVIII	5960500	5963500	1;2;	15;51;	15;0;15	1;0;1	0;0;0	0;0;0	0;0;0	0;0;0	0;0;0	0;0;0	2;0;2	0;0;0	6;0;6	6;0;6
groupXVIII	6203000	6208000	1;2;	1;65;	1;0;1	0;0;0	0;0;0	0;0;0	0;0;0	0;0;0	0;0;0	0;0;0	1;0;1	0;0;0	0;0;0	0;0;0
groupXVIII	7411500	7415500	1;2;	1;65;	1;0;1	0;0;0	0;0;0	0;0;0	1;0;1	0;0;0	0;0;0	0;0;0	0;0;0	0;0;0	0;0;0	0;0;0
groupXVIII	7724000	7726000	1;2;	2;64;	2;0;2	0;0;0	2;0;2	0;0;0	0;0;0	0;0;0	0;0;0	0;0;0	0;0;0	0;0;0	0;0;0	0;0;0
groupXVIII	8141500	8142500	0;1;2;	3;10;53;	16;3;10	0;0;0	6;2;2	1;0;1	6;1;4	3;0;3	0;0;0	0;0;0	0;0;0	0;0;0	0;0;0	0;0;0
groupXVIII	8184500	8188500	1;2;	1;65;	1;0;1	1;0;1	0;0;0	0;0;0	0;0;0	0;0;0	0;0;0	0;0;0	0;0;0	0;0;0	0;0;0	0;0;0
groupXVIII	10936500	10961000	2;3;4;	62;3;1;	5;1;3	1;0;1	0;0;0	1;0;1	0;0;0	3;1;1	0;0;0	0;0;0	0;0;0	0;0;0	0;0;0	0;0;0
groupXVIII	11055500	11058000	1;2;	4;62;	4;0;4	1;0;1	0;0;0	0;0;0	0;0;0	0;0;0	0;0;0	0;0;0	1;0;1	1;0;1	1;0;1	0;0;0
groupXVIII	11175500	11189500	2;3;	63;3;	3;0;3	0;0;0	0;0;0	0;0;0	3;0;3	0;0;0	0;0;0	0;0;0	0;0;0	0;0;0	0;0;0	0;0;0
groupXVIII	11242000	11245000	0;1;2;	1;15;50;	17;1;15	0;0;0	0;0;0	0;0;0	0;0;0	0;0;0	0;0;0	0;0;0	5;0;5	4;1;2	2;0;2	6;0;6

groupXVIII	11324000	11325500	1;2;	2;64;	2;0;2	0;0;0	0;0;0	0;0;0	0;0;0	0;0;0	0;0;0	1;0;1	0;0;0	1;0;1	0;0;0	0;0;0
groupXVIII	11620000	11624000	0;1;2;	3;4;59;	10;3;4	0;0;0	0;0;0	8;2;4	0;0;0	2;1;0	0;0;0	0;0;0	0;0;0	0;0;0	0;0;0	0;0;0
groupXVIII	11954000	11960000	1;2;	1;65;	1;0;1	0;0;0	0;0;0	0;0;0	0;0;0	0;0;0	0;0;0	1;0;1	0;0;0	0;0;0	0;0;0	0;0;0
groupXVIII	12093500	12096000	1;2;	2;64;	2;0;2	0;0;0	0;0;0	0;0;0	1;0;1	1;0;1	0;0;0	0;0;0	0;0;0	0;0;0	0;0;0	0;0;0
groupXVIII	12669500	12690500	2;3;	57;9;	9;0;9	0;0;0	0;0;0	0;0;0	0;0;0	0;0;0	0;0;0	1;0;1	0;0;0	0;0;0	4;0;4	4;0;4
groupXVIII	12762500	12763500	0;1;2;	1;8;57;	10;1;8	6;1;4	0;0;0	0;0;0	0;0;0	1;0;1	1;0;1	2;0;2	0;0;0	0;0;0	0;0;0	0;0;0
groupXVIII	12988000	12991000	1;2;	2;64;	2;0;2	0;0;0	0;0;0	0;0;0	0;0;0	0;0;0	0;0;0	0;0;0	2;0;2	0;0;0	0;0;0	0;0;0
groupXVIII	13562500	13571500	2;3;	64;2;	2;0;2	2;0;2	0;0;0	0;0;0	0;0;0	0;0;0	0;0;0	0;0;0	0;0;0	0;0;0	0;0;0	0;0;0
groupXVIII	13811500	13817000	1;2;	1;65;	1;0;1	0;0;0	1;0;1	0;0;0	0;0;0	0;0;0	0;0;0	0;0;0	0;0;0	0;0;0	0;0;0	0;0;0
groupXVIII	14172000	14176500	1;2;	2;64;	2;0;2	1;0;1	0;0;0	0;0;0	0;0;0	0;0;0	0;0;0	0;0;0	0;0;0	1;0;1	0;0;0	0;0;0
groupXVIII	14676500	14681500	1;2;	2;64;	2;0;2	1;0;1	0;0;0	0;0;0	0;0;0	0;0;0	0;0;0	0;0;0	0;0;0	0;0;0	0;0;0	1;0;1
groupXVIII	14764000	14775000	2;3;	64;2;	2;0;2	0;0;0	0;0;0	0;0;0	2;0;2	0;0;0	0;0;0	0;0;0	0;0;0	0;0;0	0;0;0	0;0;0
groupXVIII	15417000	15426000	2;3;	65;1;	1;0;1	0;0;0	0;0;0	0;0;0	0;0;0	0;0;0	0;0;0	0;0;0	1;0;1	0;0;0	0;0;0	0;0;0
groupXVIII	15616000	15618500	1;2;	1;65;	1;0;1	0;0;0	0;0;0	0;0;0	0;0;0	0;0;0	0;0;0	0;0;0	0;0;0	0;0;0	0;0;0	1;0;1
groupXVIII	15712500	15720500	2;3;	60;6;	6;0;6	0;0;0	1;0;1	0;0;0	2;0;2	2;0;2	0;0;0	0;0;0	0;0;0	1;0;1	0;0;0	0;0;0
groupXVIII	15817000	15834500	1;2;	9;57;	9;0;9	0;0;0	0;0;0	0;0;0	1;0;1	1;0;1	0;0;0	0;0;0	2;0;2	1;0;1	0;0;0	4;0;4
groupXVIII	16012000	16029000	2;3;	65;1;	1;0;1	0;0;0	0;0;0	0;0;0	0;0;0	0;0;0	0;0;0	1;0;1	0;0;0	0;0;0	0;0;0	0;0;0
groupXX	245500	304500	2;3;	65;1;	1;0;1	1;0;1	0;0;0	0;0;0	0;0;0	0;0;0	0;0;0	0;0;0	0;0;0	0;0;0	0;0;0	0;0;0
groupXX	904000	906000	0;1;2;	6;9;51;	21;6;9	4;1;2	0;0;0	0;0;0	0;0;0	0;0;0	8;2;4	9;3;3	0;0;0	0;0;0	0;0;0	0;0;0
groupXX	922000	923500	0;1;2;	4;2;60;	10;4;2	0;0;0	0;0;0	0;0;0	0;0;0	0;0;0	0;0;0	10;4;2	0;0;0	0;0;0	0;0;0	0;0;0
groupXX	929500	933500	2;3;4;	48;15;3;	21;3;15	0;0;0	7;1;5	3;0;3	5;1;3	6;1;4	0;0;0	0;0;0	0;0;0	0;0;0	0;0;0	0;0;0
groupXX	1014000	1015000	0;1;2;	2;4;60;	8;2;4	0;0;0	0;0;0	0;0;0	0;0;0	0;0;0	6;2;2	1;0;1	0;0;0	1;0;1	0;0;0	0;0;0
groupXX	1529000	1531000	0;1;2;	1;1;64;	3;1;1	0;0;0	0;0;0	3;1;1	0;0;0	0;0;0	0;0;0	0;0;0	0;0;0	0;0;0	0;0;0	0;0;0
groupXX	1924500	1950500	2;3;	65;1;	1;0;1	1;0;1	0;0;0	0;0;0	0;0;0	0;0;0	0;0;0	0;0;0	0;0;0	0;0;0	0;0;0	0;0;0
groupXX	2077500	2109000	2;3;4;	62;2;2;	6;2;2	0;0;0	0;0;0	0;0;0	0;0;0	0;0;0	0;0;0	6;2;2	0;0;0	0;0;0	0;0;0	0;0;0
groupXX	2361000	2377000	1;2;	4;62;	4;0;4	0;0;0	0;0;0	0;0;0	0;0;0	0;0;0	0;0;0	4;0;4	0;0;0	0;0;0	0;0;0	0;0;0
groupXX	2363500	2374500	1;2;	4;62;	4;0;4	0;0;0	0;0;0	0;0;0	0;0;0	0;0;0	0;0;0	4;0;4	0;0;0	0;0;0	0;0;0	0;0;0
groupXX	2687500	2695500	1;2;	1;65;	1;0;1	1;0;1	0;0;0	0;0;0	0;0;0	0;0;0	0;0;0	0;0;0	0;0;0	0;0;0	0;0;0	0;0;0
groupXX	3756500	3759500	1;2;	4;62;	4;0;4	0;0;0	0;0;0	0;0;0	4;0;4	0;0;0	0;0;0	0;0;0	0;0;0	0;0;0	0;0;0	0;0;0
groupXX	3766000	3767500	0;1;2;	2;5;59;	9;2;5	0;0;0	0;0;0	0;0;0	0;0;0	0;0;0	6;2;2	3;0;3	0;0;0	0;0;0	0;0;0	0;0;0
groupXX	3934000	3942000	2;3;4;	50;15;1;	17;1;15	3;0;3	7;1;5	2;0;2	3;0;3	2;0;2	0;0;0	0;0;0	0;0;0	0;0;0	0;0;0	0;0;0
groupXX	4571000	4577000	2;3;	64;2;	2;0;2	0;0;0	0;0;0	0;0;0	2;0;2	0;0;0	0;0;0	0;0;0	0;0;0	0;0;0	0;0;0	0;0;0
groupXX	4841000	4842500	0;1;2;	1;3;62;	5;1;3	0;0;0	5;1;3	0;0;0	0;0;0	0;0;0	0;0;0	0;0;0	0;0;0	0;0;0	0;0;0	0;0;0
groupXX	4927000	4928000	0;1;2;	5;5;56;	15;5;5	1;0;1	0;0;0	0;0;0	0;0;0	0;0;0	0;0;0	0;0;0	5;1;3	7;3;1	0;0;0	2;1;0

groupXX	5298000	5301500	1;2;	1;65;	1;0;1	1;0;1	0;0;0	0;0;0	0;0;0	0;0;0	0;0;0	0;0;0	0;0;0	0;0;0	0;0;0	0;0;0
groupXX	5454500	5457000	1;2;	1;65;	1;0;1	0;0;0	0;0;0	1;0;1	0;0;0	0;0;0	0;0;0	0;0;0	0;0;0	0;0;0	0;0;0	0;0;0
groupXX	6318000	6328500	2;3;	64;2;	2;0;2	0;0;0	0;0;0	0;0;0	0;0;0	1;0;1	0;0;0	1;0;1	0;0;0	0;0;0	0;0;0	0;0;0
groupXX	6335000	6339500	1;2;	19;47;	19;0;19	0;0;0	0;0;0	0;0;0	0;0;0	0;0;0	0;0;0	0;0;0	6;0;6	1;0;1	6;0;6	6;0;6
groupXX	7081500	7083500	0;1;2;	1;1;64;	3;1;1	0;0;0	0;0;0	0;0;0	0;0;0	0;0;0	0;0;0	0;0;0	0;0;0	0;0;0	3;1;1	0;0;0
groupXX	7191000	7200000	2;3;	64;2;	2;0;2	0;0;0	0;0;0	1;0;1	0;0;0	1;0;1	0;0;0	0;0;0	0;0;0	0;0;0	0;0;0	0;0;0
groupXX	7358500	7359500	0;1;2;	1;2;63;	4;1;2	0;0;0	0;0;0	4;1;2	0;0;0	0;0;0	0;0;0	0;0;0	0;0;0	0;0;0	0;0;0	0;0;0
groupXX	7528500	7531500	1;2;	2;64;	2;0;2	0;0;0	0;0;0	0;0;0	0;0;0	0;0;0	0;0;0	0;0;0	0;0;0	0;0;0	2;0;2	0;0;0
groupXX	7597000	7601000	1;2;	1;65;	1;0;1	0;0;0	0;0;0	0;0;0	0;0;0	0;0;0	0;0;0	1;0;1	0;0;0	0;0;0	0;0;0	0;0;0
groupXX	8306500	8311000	2;3;4;	60;5;1;	7;1;5	0;0;0	0;0;0	3;0;3	0;0;0	4;1;2	0;0;0	0;0;0	0;0;0	0;0;0	0;0;0	0;0;0
groupXX	8660000	8677500	0;1;2;	1;9;56;	11;1;9	0;0;0	0;0;0	0;0;0	0;0;0	0;0;0	0;0;0	0;0;0	2;0;2	2;0;2	3;0;3	4;1;2
groupXX	8893500	8897500	2;3;	64;2;	2;0;2	0;0;0	0;0;0	0;0;0	0;0;0	0;0;0	0;0;0	0;0;0	0;0;0	2;0;2	0;0;0	0;0;0
groupXX	9148500	9152000	2;3;4;	49;12;5;	22;5;12	0;0;0	9;3;3	4;1;2	3;0;3	6;1;4	0;0;0	0;0;0	0;0;0	0;0;0	0;0;0	0;0;0
groupXX	10319000	10321500	0;1;2;	12;8;46;	32;12;8	0;0;0	0;0;0	0;0;0	0;0;0	0;0;0	0;0;0	0;0;0	4;0;4	6;2;2	12;6;0	10;4;2
groupXX	10332000	10336000	2;3;	65;1;	1;0;1	0;0;0	0;0;0	0;0;0	0;0;0	0;0;0	0;0;0	0;0;0	0;0;0	1;0;1	0;0;0	0;0;0
groupXX	10345500	10346500	1;2;	15;51;	15;0;15	0;0;0	0;0;0	0;0;0	0;0;0	0;0;0	0;0;0	0;0;0	3;0;3	0;0;0	6;0;6	6;0;6
groupXX	13093500	13103000	2;3;	60;6;	6;0;6	0;0;0	0;0;0	0;0;0	0;0;0	0;0;0	0;0;0	0;0;0	4;0;4	2;0;2	0;0;0	0;0;0
groupXX	13896000	13903000	2;3;	64;2;	2;0;2	0;0;0	0;0;0	0;0;0	0;0;0	0;0;0	0;0;0	2;0;2	0;0;0	0;0;0	0;0;0	0;0;0
groupXX	14212000	14232000	2;3;	60;6;	6;0;6	1;0;1	0;0;0	1;0;1	0;0;0	4;0;4	0;0;0	0;0;0	0;0;0	0;0;0	0;0;0	0;0;0
groupXX	14257000	14264000	2;3;	61;5;	5;0;5	0;0;0	4;0;4	0;0;0	1;0;1	0;0;0	0;0;0	0;0;0	0;0;0	0;0;0	0;0;0	0;0;0
groupXX	14277000	14283500	1;2;	1;65;	1;0;1	0;0;0	0;0;0	0;0;0	0;0;0	0;0;0	0;0;0	0;0;0	0;0;0	0;0;0	0;0;0	1;0;1
groupXX	14342500	14346500	2;3;4;	41;4;21;	46;21;4	1;0;1	0;0;0	0;0;0	0;0;0	0;0;0	0;0;0	0;0;0	10;4;2	11;5;1	12;6;0	12;6;0
groupXX	14353500	14373000	2;3;	64;2;	2;0;2	2;0;2	0;0;0	0;0;0	0;0;0	0;0;0	0;0;0	0;0;0	0;0;0	0;0;0	0;0;0	0;0;0
groupXX	14420000	14425000	1;2;	3;63;	3;0;3	0;0;0	0;0;0	0;0;0	0;0;0	0;0;0	0;0;0	0;0;0	1;0;1	0;0;0	1;0;1	1;0;1
groupXX	14457500	14460000	1;2;	1;65;	1;0;1	0;0;0	0;0;0	0;0;0	0;0;0	1;0;1	0;0;0	0;0;0	0;0;0	0;0;0	0;0;0	0;0;0
groupXX	15681500	15687000	2;3;	65;1;	1;0;1	0;0;0	0;0;0	0;0;0	0;0;0	0;0;0	0;0;0	0;0;0	0;0;0	0;0;0	1;0;1	0;0;0
groupXX	15877000	15884500	2;3;	62;4;	4;0;4	0;0;0	0;0;0	0;0;0	0;0;0	0;0;0	0;0;0	0;0;0	1;0;1	2;0;2	1;0;1	0;0;0
groupXX	15887500	15895500	2;3;	63;3;	3;0;3	0;0;0	0;0;0	0;0;0	0;0;0	0;0;0	0;0;0	0;0;0	1;0;1	1;0;1	1;0;1	0;0;0
groupXX	15972000	15983000	2;3;4;	59;6;1;	8;1;6	0;0;0	0;0;0	0;0;0	0;0;0	0;0;0	0;0;0	0;0;0	2;0;2	6;1;4	0;0;0	0;0;0
groupXX	16246000	16247000	0;1;2;	2;9;55;	13;2;9	0;0;0	5;1;3	1;0;1	3;1;1	2;0;2	0;0;0	1;0;1	0;0;0	0;0;0	0;0;0	1;0;1
groupXX	16386000	16397000	2;3;	61;5;	5;0;5	0;0;0	0;0;0	0;0;0	0;0;0	0;0;0	0;0;0	0;0;0	3;0;3	1;0;1	0;0;0	1;0;1
groupXX	16404000	16407000	1;2;	1;65;	1;0;1	0;0;0	0;0;0	0;0;0	0;0;0	0;0;0	0;0;0	1;0;1	0;0;0	0;0;0	0;0;0	0;0;0
groupXX	16473000	16480000	2;3;	64;2;	2;0;2	2;0;2	0;0;0	0;0;0	0;0;0	0;0;0	0;0;0	0;0;0	0;0;0	0;0;0	0;0;0	0;0;0
groupXX	16501500	16516000	1;2;	1;65;	1;0;1	0;0;0	0;0;0	0;0;0	0;0;0	0;0;0	0;0;0	0;0;0	0;0;0	0;0;0	1;0;1	0;0;0

groupXX	16829500	16831500	0;1;2;	1;3;62;	5;1;3	0;0;0	3;1;1	0;0;0	2;0;2	0;0;0	0;0;0	0;0;0	0;0;0	0;0;0	0;0;0	0;0;0
groupXX	17266500	17274500	1;2;	1;65;	1;0;1	0;0;0	0;0;0	0;0;0	0;0;0	0;0;0	0;0;0	0;0;0	0;0;0	0;0;0	1;0;1	0;0;0
groupXX	18122000	18125000	0;1;2;	6;5;55;	17;6;5	0;0;0	0;0;0	0;0;0	0;0;0	0;0;0	12;6;0	2;0;2	2;0;2	1;0;1	0;0;0	0;0;0
groupXX	18219500	18235500	2;3;	65;1;	1;0;1	1;0;1	0;0;0	0;0;0	0;0;0	0;0;0	0;0;0	0;0;0	0;0;0	0;0;0	0;0;0	0;0;0
groupXX	18316000	18320500	1;2;	4;62;	4;0;4	0;0;0	0;0;0	0;0;0	0;0;0	0;0;0	0;0;0	0;0;0	1;0;1	3;0;3	0;0;0	0;0;0
groupXX	18357500	18358500	0;1;2;	4;8;54;	16;4;8	0;0;0	0;0;0	0;0;0	0;0;0	0;0;0	0;0;0	0;0;0	4;0;4	3;0;3	0;0;0	9;4;1
groupXX	18795000	18806500	2;3;	65;1;	1;0;1	0;0;0	0;0;0	0;0;0	0;0;0	0;0;0	0;0;0	1;0;1	0;0;0	0;0;0	0;0;0	0;0;0
groupXX	18842500	18851500	1;2;	1;65;	1;0;1	0;0;0	0;0;0	0;0;0	0;0;0	0;0;0	0;0;0	1;0;1	0;0;0	0;0;0	0;0;0	0;0;0
groupXX	19093000	19099000	1;2;	2;64;	2;0;2	0;0;0	0;0;0	0;0;0	0;0;0	0;0;0	0;0;0	0;0;0	1;0;1	1;0;1	0;0;0	0;0;0
groupXX	19166000	19171000	1;2;	1;65;	1;0;1	1;0;1	0;0;0	0;0;0	0;0;0	0;0;0	0;0;0	0;0;0	0;0;0	0;0;0	0;0;0	0;0;0
groupXX	19248500	19249500	0;1;2;	1;3;62;	5;1;3	0;0;0	0;0;0	3;1;1	0;0;0	2;0;2	0;0;0	0;0;0	0;0;0	0;0;0	0;0;0	0;0;0
groupXX	19307000	19309000	0;1;2;	3;8;55;	14;3;8	0;0;0	0;0;0	0;0;0	0;0;0	0;0;0	0;0;0	0;0;0	1;0;1	0;0;0	8;2;4	5;1;3
groupXX	19487500	19490000	1;2;	2;64;	2;0;2	0;0;0	0;0;0	0;0;0	0;0;0	0;0;0	0;0;0	0;0;0	2;0;2	0;0;0	0;0;0	0;0;0
groupXX	19496000	19497000	0;2;	1;65;	2;1;0	2;1;0	0;0;0	0;0;0	0;0;0	0;0;0	0;0;0	0;0;0	0;0;0	0;0;0	0;0;0	0;0;0
groupXX	19567000	19588000	1;2;	1;65;	1;0;1	0;0;0	0;0;0	0;0;0	1;0;1	0;0;0	0;0;0	0;0;0	0;0;0	0;0;0	0;0;0	0;0;0
groupXX	19659000	19662000	0;1;2;	2;8;56;	12;2;8	7;2;3	0;0;0	2;0;2	0;0;0	0;0;0	0;0;0	3;0;3	0;0;0	0;0;0	0;0;0	0;0;0
groupXXI	322500	328500	1;2;	10;56;	10;0;10	0;0;0	0;0;0	0;0;0	0;0;0	0;0;0	0;0;0	0;0;0	2;0;2	0;0;0	5;0;5	3;0;3
groupXXI	375000	377500	0;1;2;	1;4;61;	6;1;4	0;0;0	0;0;0	0;0;0	0;0;0	1;0;1	5;1;3	0;0;0	0;0;0	0;0;0	0;0;0	0;0;0
groupXXI	416500	419500	0;1;2;	3;7;56;	13;3;7	0;0;0	0;0;0	0;0;0	0;0;0	0;0;0	0;0;0	0;0;0	9;3;3	4;0;4	0;0;0	0;0;0
groupXXI	493500	495500	2;3;	63;3;	3;0;3	0;0;0	0;0;0	2;0;2	0;0;0	0;0;0	1;0;1	0;0;0	0;0;0	0;0;0	0;0;0	0;0;0
groupXXI	674500	676000	0;1;2;	1;9;56;	11;1;9	1;0;1	0;0;0	0;0;0	0;0;0	0;0;0	0;0;0	0;0;0	1;0;1	0;0;0	6;1;4	3;0;3
groupXXI	818500	821500	2;3;	65;1;	1;0;1	0;0;0	0;0;0	0;0;0	0;0;0	0;0;0	0;0;0	1;0;1	0;0;0	0;0;0	0;0;0	0;0;0
groupXXI	1273500	1275500	0;1;2;	3;8;55;	14;3;8	0;0;0	0;0;0	0;0;0	0;0;0	0;0;0	0;0;0	0;0;0	2;0;2	0;0;0	6;1;4	6;2;2
groupXXI	1555500	1571500	2;3;	64;2;	2;0;2	0;0;0	0;0;0	0;0;0	2;0;2	0;0;0	0;0;0	0;0;0	0;0;0	0;0;0	0;0;0	0;0;0
groupXXI	1592000	1593500	0;1;2;	4;16;46;	24;4;16	0;0;0	0;0;0	0;0;0	0;0;0	0;0;0	0;0;0	0;0;0	4;0;4	4;0;4	7;1;5	9;3;3
groupXXI	1959000	1960000	0;1;2;	12;3;51;	27;12;3	0;0;0	0;0;0	0;0;0	0;0;0	0;0;0	0;0;0	0;0;0	3;0;3	0;0;0	12;6;0	12;6;0
groupXXI	1971000	1982000	2;3;	65;1;	1;0;1	0;0;0	0;0;0	0;0;0	0;0;0	0;0;0	0;0;0	0;0;0	0;0;0	0;0;0	0;0;0	1;0;1
groupXXI	2104000	2121500	1;2;	14;52;	14;0;14	0;0;0	0;0;0	0;0;0	0;0;0	0;0;0	1;0;1	0;0;0	0;0;0	5;0;5	3;0;3	5;0;5
groupXXI	2866500	2867500	0;1;2;	5;8;53;	18;5;8	0;0;0	0;0;0	0;0;0	0;0;0	0;0;0	0;0;0	0;0;0	0;0;0	2;0;2	7;2;3	9;3;3
groupXXI	2877000	2879000	1;2;	7;59;	7;0;7	0;0;0	0;0;0	0;0;0	0;0;0	0;0;0	0;0;0	0;0;0	1;0;1	0;0;0	2;0;2	4;0;4
groupXXI	3774000	3797000	2;3;	65;1;	1;0;1	0;0;0	0;0;0	0;0;0	0;0;0	0;0;0	0;0;0	1;0;1	0;0;0	0;0;0	0;0;0	0;0;0
groupXXI	4372500	4381000	2;3;	63;3;	3;0;3	0;0;0	0;0;0	3;0;3	0;0;0	0;0;0	0;0;0	0;0;0	0;0;0	0;0;0	0;0;0	0;0;0
groupXXI	5665500	5667000	1;2;	2;64;	2;0;2	0;0;0	0;0;0	0;0;0	0;0;0	0;0;0	0;0;0	2;0;2	0;0;0	0;0;0	0;0;0	0;0;0
groupXXI	5863500	5870500	2;3;	64;2;	2;0;2	0;0;0	0;0;0	0;0;0	0;0;0	0;0;0	0;0;0	0;0;0	2;0;2	0;0;0	0;0;0	0;0;0

groupXXI	5975500	5980500	1;2;	2;64;	2;0;2	0;0;0	0;0;0	1;0;1	0;0;0	1;0;1	0;0;0	0;0;0	0;0;0	0;0;0	0;0;0	0;0;0
groupXXI	6009500	6011500	0;1;2;	1;5;60;	7;1;5	0;0;0	0;0;0	0;0;0	0;0;0	1;0;1	0;0;0	0;0;0	0;0;0	0;0;0	6;1;4	0;0;0
groupXXI	6920000	6931000	2;3;	54;12;	12;0;12	0;0;0	0;0;0	0;0;0	0;0;0	0;0;0	0;0;0	0;0;0	6;0;6	6;0;6	0;0;0	0;0;0
groupXXI	8127500	8136500	2;3;4;	59;5;2;	9;2;5	0;0;0	0;0;0	2;0;2	1;0;1	6;2;2	0;0;0	0;0;0	0;0;0	0;0;0	0;0;0	0;0;0
groupXXI	8525500	8528500	1;2;	1;65;	1;0;1	0;0;0	0;0;0	0;0;0	0;0;0	0;0;0	0;0;0	1;0;1	0;0;0	0;0;0	0;0;0	0;0;0
groupXXI	9267000	9274000	2;3;	60;6;	6;0;6	0;0;0	3;0;3	1;0;1	2;0;2	0;0;0	0;0;0	0;0;0	0;0;0	0;0;0	0;0;0	0;0;0
groupXXI	9313500	9320000	2;3;4;	64;1;1;	3;1;1	0;0;0	0;0;0	3;1;1	0;0;0	0;0;0	0;0;0	0;0;0	0;0;0	0;0;0	0;0;0	0;0;0
groupXXI	10137500	10146500	2;3;	65;1;	1;0;1	0;0;0	0;0;0	0;0;0	0;0;0	0;0;0	0;0;0	1;0;1	0;0;0	0;0;0	0;0;0	0;0;0
groupXXI	10351500	10364500	2;4;	63;3;	6;3;0	0;0;0	0;0;0	0;0;0	0;0;0	0;0;0	0;0;0	0;0;0	0;0;0	0;0;0	2;1;0	4;2;0
groupXXI	10641500	10664500	1;2;	3;63;	3;0;3	1;0;1	0;0;0	0;0;0	0;0;0	0;0;0	1;0;1	0;0;0	1;0;1	0;0;0	0;0;0	0;0;0
groupXXI	10832500	10835500	1;2;	6;60;	6;0;6	0;0;0	0;0;0	3;0;3	0;0;0	3;0;3	0;0;0	0;0;0	0;0;0	0;0;0	0;0;0	0;0;0
groupXXI	11081000	11083500	1;2;	11;55;	11;0;11	0;0;0	0;0;0	0;0;0	0;0;0	0;0;0	0;0;0	0;0;0	1;0;1	0;0;0	6;0;6	4;0;4
scaffold_101	59500	61500	0;1;2;	1;1;64;	3;1;1	0;0;0	2;1;0	0;0;0	0;0;0	1;0;1	0;0;0	0;0;0	0;0;0	0;0;0	0;0;0	0;0;0
scaffold_101	119500	122500	1;2;	2;64;	2;0;2	0;0;0	0;0;0	0;0;0	0;0;0	0;0;0	0;0;0	0;0;0	0;0;0	0;0;0	1;0;1	1;0;1
scaffold_106	32000	35000	1;2;	1;65;	1;0;1	1;0;1	0;0;0	0;0;0	0;0;0	0;0;0	0;0;0	0;0;0	0;0;0	0;0;0	0;0;0	0;0;0
scaffold_106	36500	39000	1;2;	1;65;	1;0;1	1;0;1	0;0;0	0;0;0	0;0;0	0;0;0	0;0;0	0;0;0	0;0;0	0;0;0	0;0;0	0;0;0
scaffold_106	132000	134000	0;2;	1;65;	2;1;0	0;0;0	0;0;0	0;0;0	2;1;0	0;0;0	0;0;0	0;0;0	0;0;0	0;0;0	0;0;0	0;0;0
scaffold_108	177000	178000	0;1;2;	1;8;57;	10;1;8	0;0;0	6;1;4	2;0;2	0;0;0	2;0;2	0;0;0	0;0;0	0;0;0	0;0;0	0;0;0	0;0;0
scaffold_111	109500	112000	0;1;2;	1;2;63;	4;1;2	0;0;0	0;0;0	0;0;0	0;0;0	0;0;0	0;0;0	4;1;2	0;0;0	0;0;0	0;0;0	0;0;0
scaffold_121	0	30000	2;3;	64;2;	2;0;2	1;0;1	1;0;1	0;0;0	0;0;0	0;0;0	0;0;0	0;0;0	0;0;0	0;0;0	0;0;0	0;0;0
scaffold_126	105500	116500	2;3;	64;2;	2;0;2	0;0;0	0;0;0	1;0;1	0;0;0	1;0;1	0;0;0	0;0;0	0;0;0	0;0;0	0;0;0	0;0;0
scaffold_128	139500	142500	0;1;2;	1;10;55;	12;1;10	1;0;1	0;0;0	1;0;1	4;1;2	4;0;4	0;0;0	2;0;2	0;0;0	0;0;0	0;0;0	0;0;0
scaffold_130	34000	41000	2;3;	63;3;	3;0;3	0;0;0	0;0;0	1;0;1	1;0;1	0;0;0	0;0;0	1;0;1	0;0;0	0;0;0	0;0;0	0;0;0
scaffold_135	65000	121500	2;3;4;	60;5;1;	7;1;5	7;1;5	0;0;0	0;0;0	0;0;0	0;0;0	0;0;0	0;0;0	0;0;0	0;0;0	0;0;0	0;0;0
scaffold_146	83500	111500	2;3;	65;1;	1;0;1	0;0;0	0;0;0	0;0;0	1;0;1	0;0;0	0;0;0	0;0;0	0;0;0	0;0;0	0;0;0	0;0;0
scaffold_27	393000	396000	1;2;	1;65;	1;0;1	1;0;1	0;0;0	0;0;0	0;0;0	0;0;0	0;0;0	0;0;0	0;0;0	0;0;0	0;0;0	0;0;0
scaffold_27	721500	722500	1;2;	4;62;	4;0;4	0;0;0	0;0;0	4;0;4	0;0;0	0;0;0	0;0;0	0;0;0	0;0;0	0;0;0	0;0;0	0;0;0
scaffold_27	843000	845000	0;1;2;	1;4;61;	6;1;4	0;0;0	0;0;0	0;0;0	0;0;0	0;0;0	0;0;0	0;0;0	2;1;0	4;0;4	0;0;0	0;0;0
scaffold_27	1413000	1415500	1;2;	8;58;	8;0;8	0;0;0	4;0;4	0;0;0	4;0;4	0;0;0	0;0;0	0;0;0	0;0;0	0;0;0	0;0;0	0;0;0
scaffold_27	1613500	1617000	1;2;	1;65;	1;0;1	1;0;1	0;0;0	0;0;0	0;0;0	0;0;0	0;0;0	0;0;0	0;0;0	0;0;0	0;0;0	0;0;0
scaffold_27	1918000	1919000	0;1;2;	2;5;59;	9;2;5	0;0;0	0;0;0	0;0;0	0;0;0	0;0;0	0;0;0	0;0;0	1;0;1	0;0;0	0;0;0	8;2;4
scaffold_27	1969500	1972000	1;2;	1;65;	1;0;1	0;0;0	0;0;0	0;0;0	0;0;0	0;0;0	0;0;0	1;0;1	0;0;0	0;0;0	0;0;0	0;0;0
scaffold_27	2307000	2309500	1;2;	4;62;	4;0;4	0;0;0	0;0;0	0;0;0	0;0;0	0;0;0	0;0;0	4;0;4	0;0;0	0;0;0	0;0;0	0;0;0
scaffold_27	2624000	2640500	2;3;4;	59;4;3;	10;3;4	2;0;2	2;1;0	0;0;0	2;1;0	0;0;0	0;0;0	0;0;0	3;1;1	1;0;1	0;0;0	0;0;0

scaffold_27	2669500	2672500	1;2;	1;65;	1;0;1	1;0;1	0;0;0	0;0;0	0;0;0	0;0;0	0;0;0	0;0;0	0;0;0	0;0;0	0;0;0	0;0;0
scaffold_27	2918000	2931500	2;3;	63;3;	3;0;3	3;0;3	0;0;0	0;0;0	0;0;0	0;0;0	0;0;0	0;0;0	0;0;0	0;0;0	0;0;0	0;0;0
scaffold_27	3092500	3094500	1;2;	1;65;	1;0;1	0;0;0	0;0;0	0;0;0	0;0;0	0;0;0	0;0;0	0;0;0	0;0;0	1;0;1	0;0;0	0;0;0
scaffold_27	3850000	3852000	1;2;	2;64;	2;0;2	1;0;1	0;0;0	0;0;0	0;0;0	1;0;1	0;0;0	0;0;0	0;0;0	0;0;0	0;0;0	0;0;0
scaffold_27	4006000	4007500	1;2;	9;57;	9;0;9	0;0;0	0;0;0	0;0;0	0;0;0	0;0;0	0;0;0	0;0;0	1;0;1	1;0;1	5;0;5	2;0;2
scaffold_27	4326000	4341000	0;1;2;	2;2;62;	6;2;2	0;0;0	0;0;0	6;2;2	0;0;0	0;0;0	0;0;0	0;0;0	0;0;0	0;0;0	0;0;0	0;0;0
scaffold_27	4426500	4433000	2;3;	65;1;	1;0;1	1;0;1	0;0;0	0;0;0	0;0;0	0;0;0	0;0;0	0;0;0	0;0;0	0;0;0	0;0;0	0;0;0
scaffold_27	4801500	4808000	2;3;	65;1;	1;0;1	0;0;0	0;0;0	0;0;0	1;0;1	0;0;0	0;0;0	0;0;0	0;0;0	0;0;0	0;0;0	0;0;0
scaffold_27	4975500	4977500	1;2;	7;59;	7;0;7	0;0;0	0;0;0	0;0;0	0;0;0	0;0;0	0;0;0	0;0;0	1;0;1	0;0;0	4;0;4	2;0;2
scaffold_37	13500	59000	2;3;	56;10;	10;0;10	0;0;0	0;0;0	0;0;0	0;0;0	0;0;0	0;0;0	0;0;0	4;0;4	6;0;6	0;0;0	0;0;0
scaffold_37	371000	431000	2;3;4;	54;10;2;	14;2;10	2;0;2	1;0;1	0;0;0	4;1;2	4;0;4	0;0;0	3;1;1	0;0;0	0;0;0	0;0;0	0;0;0
scaffold_37	710000	712500	1;2;	17;49;	17;0;17	0;0;0	0;0;0	0;0;0	0;0;0	0;0;0	0;0;0	0;0;0	1;0;1	4;0;4	6;0;6	6;0;6
scaffold_37	780500	789500	2;3;	63;3;	3;0;3	3;0;3	0;0;0	0;0;0	0;0;0	0;0;0	0;0;0	0;0;0	0;0;0	0;0;0	0;0;0	0;0;0
scaffold_37	864000	868000	0;1;2;	9;11;46;	29;9;11	0;0;0	0;0;0	0;0;0	0;0;0	0;0;0	0;0;0	0;0;0	2;0;2	10;4;2	10;4;2	7;1;5
scaffold_37	1116000	1120500	1;2;	2;64;	2;0;2	1;0;1	0;0;0	0;0;0	0;0;0	0;0;0	0;0;0	0;0;0	1;0;1	0;0;0	0;0;0	0;0;0
scaffold_37	1121500	1125000	0;1;2;	1;5;60;	7;1;5	0;0;0	1;0;1	1;0;1	4;1;2	0;0;0	0;0;0	0;0;0	1;0;1	0;0;0	0;0;0	0;0;0
scaffold_37	1212500	1213500	0;1;2;	3;14;49;	20;3;14	0;0;0	0;0;0	3;0;3	0;0;0	0;0;0	0;0;0	0;0;0	1;0;1	1;0;1	8;2;4	7;1;5
scaffold_37	1243500	1247000	0;1;2;	3;6;57;	12;3;6	0;0;0	0;0;0	0;0;0	1;0;1	0;0;0	0;0;0	0;0;0	0;0;0	1;0;1	2;0;2	8;3;2
scaffold_37	1310500	1314000	1;2;	4;62;	4;0;4	0;0;0	1;0;1	0;0;0	2;0;2	0;0;0	0;0;0	0;0;0	0;0;0	0;0;0	1;0;1	0;0;0
scaffold_37	1447000	1463000	2;3;	62;4;	4;0;4	0;0;0	0;0;0	0;0;0	0;0;0	0;0;0	0;0;0	0;0;0	1;0;1	1;0;1	0;0;0	2;0;2
scaffold_37	1664500	1667500	0;1;2;	1;7;58;	9;1;7	0;0;0	0;0;0	0;0;0	0;0;0	0;0;0	0;0;0	0;0;0	1;0;1	0;0;0	3;1;1	5;0;5
scaffold_37	2149000	2151000	2;3;	62;4;	4;0;4	0;0;0	0;0;0	0;0;0	0;0;0	0;0;0	0;0;0	0;0;0	2;0;2	0;0;0	2;0;2	0;0;0
scaffold_37	2228000	2260000	2;4;	64;2;	4;2;0	0;0;0	4;2;0	0;0;0	0;0;0	0;0;0	0;0;0	0;0;0	0;0;0	0;0;0	0;0;0	0;0;0
scaffold_37	2586000	2590000	1;2;	11;55;	11;0;11	0;0;0	0;0;0	0;0;0	0;0;0	1;0;1	0;0;0	0;0;0	2;0;2	0;0;0	5;0;5	3;0;3
scaffold_47	428500	430500	0;1;2;	4;6;56;	14;4;6	1;0;1	0;0;0	0;0;0	0;0;0	1;0;1	0;0;0	0;0;0	0;0;0	0;0;0	9;3;3	3;1;1
scaffold_47	451500	481500	2;3;4;	52;10;4;	18;4;10	0;0;0	0;0;0	0;0;0	0;0;0	0;0;0	0;0;0	0;0;0	8;2;4	3;0;3	0;0;0	7;2;3
scaffold_47	500000	503000	1;2;	1;65;	1;0;1	0;0;0	0;0;0	1;0;1	0;0;0	0;0;0	0;0;0	0;0;0	0;0;0	0;0;0	0;0;0	0;0;0
scaffold_47	510500	512500	0;1;2;	1;5;60;	7;1;5	0;0;0	0;0;0	0;0;0	0;0;0	0;0;0	5;1;3	1;0;1	0;0;0	0;0;0	0;0;0	1;0;1
scaffold_47	635500	655000	1;2;	24;42;	24;0;24	0;0;0	0;0;0	0;0;0	0;0;0	0;0;0	0;0;0	0;0;0	6;0;6	6;0;6	6;0;6	6;0;6
scaffold_47	937500	939000	0;1;2;	3;1;62;	7;3;1	0;0;0	0;0;0	0;0;0	0;0;0	0;0;0	0;0;0	0;0;0	0;0;0	7;3;1	0;0;0	0;0;0
scaffold_47	950000	951500	1;2;	3;63;	3;0;3	0;0;0	0;0;0	0;0;0	0;0;0	0;0;0	0;0;0	0;0;0	0;0;0	3;0;3	0;0;0	0;0;0
scaffold_47	953500	966500	1;2;	3;63;	3;0;3	3;0;3	0;0;0	0;0;0	0;0;0	0;0;0	0;0;0	0;0;0	0;0;0	0;0;0	0;0;0	0;0;0
scaffold_47	986500	992500	1;2;	3;63;	3;0;3	0;0;0	0;0;0	0;0;0	0;0;0	0;0;0	3;0;3	0;0;0	0;0;0	0;0;0	0;0;0	0;0;0
scaffold_47	1028500	1029500	0;1;2;	2;3;61;	7;2;3	0;0;0	0;0;0	0;0;0	0;0;0	0;0;0	0;0;0	0;0;0	7;2;3	0;0;0	0;0;0	0;0;0

scaffold_47	1488000	1492000	0;1;2;	1;6;59;	8;1;6	2;0;2	0;0;0	0;0;0	0;0;0	0;0;0	5;1;3	1;0;1	0;0;0	0;0;0	0;0;0	0;0;0
scaffold_47	1595000	1600500	1;2;	6;60;	6;0;6	0;0;0	0;0;0	2;0;2	1;0;1	2;0;2	0;0;0	0;0;0	0;0;0	1;0;1	0;0;0	0;0;0
scaffold_470	0	17500	1;2;	5;61;	5;0;5	0;0;0	0;0;0	0;0;0	0;0;0	0;0;0	0;0;0	0;0;0	4;0;4	1;0;1	0;0;0	0;0;0
scaffold_48	47000	55000	2;3;	64;2;	2;0;2	0;0;0	1;0;1	0;0;0	1;0;1	0;0;0	0;0;0	0;0;0	0;0;0	0;0;0	0;0;0	0;0;0
scaffold_48	348000	354500	1;2;	1;65;	1;0;1	0;0;0	0;0;0	1;0;1	0;0;0	0;0;0	0;0;0	0;0;0	0;0;0	0;0;0	0;0;0	0;0;0
scaffold_48	1459500	1461500	0;1;2;	3;18;45;	24;3;18	5;1;3	1;0;1	4;0;4	5;1;3	3;0;3	0;0;0	6;1;4	0;0;0	0;0;0	0;0;0	0;0;0
scaffold_508	0	16000	2;3;	62;4;	4;0;4	0;0;0	0;0;0	0;0;0	0;0;0	0;0;0	0;0;0	2;0;2	0;0;0	0;0;0	0;0;0	2;0;2
scaffold_54	22000	60000	1;2;	6;60;	6;0;6	2;0;2	0;0;0	0;0;0	0;0;0	0;0;0	0;0;0	0;0;0	3;0;3	1;0;1	0;0;0	0;0;0
scaffold_54	628000	633500	0;2;	1;65;	2;1;0	0;0;0	0;0;0	0;0;0	0;0;0	0;0;0	0;0;0	0;0;0	2;1;0	0;0;0	0;0;0	0;0;0
scaffold_56	89000	92000	1;2;	2;64;	2;0;2	0;0;0	0;0;0	0;0;0	0;0;0	0;0;0	0;0;0	0;0;0	0;0;0	2;0;2	0;0;0	0;0;0
scaffold_56	291000	298000	1;2;	1;65;	1;0;1	1;0;1	0;0;0	0;0;0	0;0;0	0;0;0	0;0;0	0;0;0	0;0;0	0;0;0	0;0;0	0;0;0
scaffold_56	298500	360000	1;2;	1;65;	1;0;1	0;0;0	0;0;0	0;0;0	0;0;0	0;0;0	0;0;0	1;0;1	0;0;0	0;0;0	0;0;0	0;0;0
scaffold_56	430000	436000	1;2;	1;65;	1;0;1	0;0;0	0;0;0	0;0;0	0;0;0	0;0;0	0;0;0	0;0;0	0;0;0	1;0;1	0;0;0	0;0;0
scaffold_56	639000	643000	1;2;	1;65;	1;0;1	0;0;0	1;0;1	0;0;0	0;0;0	0;0;0	0;0;0	0;0;0	0;0;0	0;0;0	0;0;0	0;0;0
scaffold_56	798000	801500	0;1;2;	1;12;53;	14;1;12	0;0;0	0;0;0	0;0;0	0;0;0	0;0;0	0;0;0	0;0;0	1;0;1	6;1;4	2;0;2	5;0;5
scaffold_56	874500	878500	0;1;2;	3;20;43;	26;3;20	0;0;0	0;0;0	0;0;0	0;0;0	0;0;0	0;0;0	0;0;0	5;0;5	8;2;4	6;0;6	7;1;5
scaffold_56	1076000	1169000	2;3;4;	50;13;3;	19;3;13	0;0;0	0;0;0	0;0;0	0;0;0	1;0;1	0;0;0	0;0;0	4;1;2	3;0;3	7;1;5	4;1;2
scaffold_58	0	725000	1;2;	2;64;	2;0;2	0;0;0	0;0;0	2;0;2	0;0;0	0;0;0	0;0;0	0;0;0	0;0;0	0;0;0	0;0;0	0;0;0
scaffold_61	70500	76500	1;2;	1;65;	1;0;1	0;0;0	0;0;0	0;0;0	0;0;0	1;0;1	0;0;0	0;0;0	0;0;0	0;0;0	0;0;0	0;0;0
scaffold_61	178500	186000	2;3;	64;2;	2;0;2	1;0;1	0;0;0	0;0;0	0;0;0	0;0;0	0;0;0	1;0;1	0;0;0	0;0;0	0;0;0	0;0;0
scaffold_67	43500	48500	0;1;2;	2;5;59;	9;2;5	0;0;0	8;2;4	0;0;0	1;0;1	0;0;0	0;0;0	0;0;0	0;0;0	0;0;0	0;0;0	0;0;0
scaffold_67	713000	716000	0;2;	1;65;	2;1;0	2;1;0	0;0;0	0;0;0	0;0;0	0;0;0	0;0;0	0;0;0	0;0;0	0;0;0	0;0;0	0;0;0
scaffold_68	381500	401500	1;2;	28;38;	28;0;28	4;0;4	0;0;0	0;0;0	0;0;0	5;0;5	6;0;6	1;0;1	6;0;6	6;0;6	0;0;0	0;0;0
scaffold_68	717000	734500	2;3;	65;1;	1;0;1	1;0;1	0;0;0	0;0;0	0;0;0	0;0;0	0;0;0	0;0;0	0;0;0	0;0;0	0;0;0	0;0;0
scaffold_69	747000	756000	2;3;	65;1;	1;0;1	0;0;0	0;0;0	0;0;0	0;0;0	1;0;1	0;0;0	0;0;0	0;0;0	0;0;0	0;0;0	0;0;0
scaffold_74	247000	252000	2;3;4;	50;12;4;	20;4;12	0;0;0	8;2;4	6;1;4	4;1;2	2;0;2	0;0;0	0;0;0	0;0;0	0;0;0	0;0;0	0;0;0
scaffold_76	106500	109500	0;1;2;	2;4;60;	8;2;4	0;0;0	0;0;0	0;0;0	0;0;0	0;0;0	0;0;0	1;0;1	0;0;0	0;0;0	5;2;1	2;0;2
scaffold_76	122000	124500	0;1;2;	2;3;61;	7;2;3	0;0;0	0;0;0	0;0;0	0;0;0	0;0;0	0;0;0	0;0;0	0;0;0	0;0;0	5;2;1	2;0;2
scaffold_80	476000	489500	2;3;	58;8;	8;0;8	0;0;0	0;0;0	3;0;3	0;0;0	2;0;2	2;0;2	1;0;1	0;0;0	0;0;0	0;0;0	0;0;0
scaffold_84	164000	165000	0;1;2;	1;6;59;	8;1;6	0;0;0	0;0;0	0;0;0	0;0;0	0;0;0	0;0;0	0;0;0	1;0;1	1;0;1	0;0;0	6;1;4
scaffold_84	354000	356000	0;1;2;	2;4;60;	8;2;4	1;0;1	0;0;0	6;2;2	1;0;1	0;0;0	0;0;0	0;0;0	0;0;0	0;0;0	0;0;0	0;0;0
scaffold_84	460500	463500	0;1;2;	1;11;54;	13;1;11	0;0;0	2;0;2	0;0;0	0;0;0	0;0;0	6;1;4	3;0;3	1;0;1	1;0;1	0;0;0	0;0;0
scaffold_84	470000	472500	1;2;	1;65;	1;0;1	0;0;0	0;0;0	0;0;0	0;0;0	1;0;1	0;0;0	0;0;0	0;0;0	0;0;0	0;0;0	0;0;0
scaffold_89	26000	55000	2;3;	65;1;	1;0;1	0;0;0	0;0;0	0;0;0	0;0;0	0;0;0	0;0;0	0;0;0	0;0;0	0;0;0	0;0;0	1;0;1

scaffold_90	78500	110000	1;2;	1;65;	1;0;1	0;0;0	0;0;0	0;0;0	0;0;0	0;0;0	0;0;0	0;0;0	0;0;0	0;0;0	0;0;0	1;0;1
scaffold_90	193500	202000	2;3;	65;1;	1;0;1	0;0;0	0;0;0	0;0;0	0;0;0	0;0;0	0;0;0	0;0;0	0;0;0	0;0;0	0;0;0	1;0;1
