# Supplementary material for: Extensive Copy-Number Variation of Young Genes across Stickleback Populations
Source: PLoS Genet. 2014 Dec 4;10(12):e1004830. doi: 10.1371/journal.pgen.1004830 (PMC4256280; doi:10.1371/journal.pgen.1004830)
Supplement: Table S3 — CNVs that are private to a population or region and found in each individual from that population or region. Asterisk indicates fixed CNVs in the population or region. (PDF) [file pgen.1004830.s025.pdf]

Supplementary Table 3 - CNVs that are private to a population or region and found in each individual from that population or region. Asterisk indicates fixed CNVs in the population or region.

Chrom.	Start	End	Inds	Pops	Private to	CNV	Gene ID	Gene Position	Gene	Gene Name	Gene Function
groupI	19765500	19794500	6	1	G2_L	del	ENSGACG00000013689	overlap	PIPOX	pipecolic acid oxidase	oxidoreductase activity
groupIII	10259500	10261000	6	1	G1_R*	del	ENSGACG00000016391	upstream	TMTOPSA	teleost multiple tissue opsin a	G-protein coupled receptor signaling pathway, visual perception
groupIV	3558500	3566000	6	1	Ca_R	dup	ENSGACG00000016734	upstream	ANKRD50	ankyrin repeat domain 50	protein binding
groupIV	13212500	13221000	6	1	No_R	dup	ENSGACG00000018337	overlap	UNC5A	unc-5 homolog A	signal transduction,protein binding
groupIV	13212500	13221000	6	1	No_R	dup	ENSGACG00000018344	overlap	PDLIM7	PDZ and LIM domain 7 (enigma)	zinc ion binding,protein binding
groupXI	797000	811500	6	1	Dk_M	dup	ENSGACG00000005131	overlap	B4GALNT2	beta-1,4-N-acetyl-galactosaminyl transferase 2	-
groupV	9798500	9800500	12	2	No*	del	ENSGACG00000007890	overlap	-	-	one-carbon metabolic process,zinc ion binding,carbonate dehydratase activity
groupIV	17364000	17372500	12	2	Ca	del	ENSGACG00000018760	downstream	.	.	regulation of transcription, DNA-dependent, sequence-specific DNA binding transcription factor activity
groupI	3166500	3169000	42	7	Atlantic	del	ENSGACG00000006463	overlap	MMP20	matrix metallopeptidase 20	proteolysis, metabolic process, extracellular matrix, zinc ion binding, metalloendopeptidase activity
groupI	17595500	17598000	42	7	Atlantic	del	.		.	.	.
groupII	10997500	11000000	42	7	Atlantic	del	ENSGACG00000015782	overlap	POGK	pogo transposable element with KRAB domain	nucleic acid binding,protein binding
groupIV	17193500	17196000	42	7	Atlantic	del	ENSGACG00000018757	upstream	SLITRK4	SLIT and NTRK-like family member 4	protein binding
groupIX	12447000	12449500	42	7	Atlantic	del	ENSGACG00000018479	overlap	CLGN	calmegin	calcium ion binding
groupVIII	7788000	7791000	42	7	Atlantic	del	ENSGACG00000006964	upstream	PGM1	phosphoglucomutase 1	carbohydrate metabolic process, magnesium ion binding, intramolecular transferase activity, phosphotransferases
groupVIII	7788000	7791000	42	7	Atlantic	del	ENSGACG00000007064	downstream	ROR1	receptor tyronsine kinase-like orphan receptor 1	protein phosphorylation, ATP binding, protein binding, transferase activity, protein serine/threonine kinase activity, protein tyrosine kinase activity
groupX	5938000	5940500	42	7	Atlantic	del	ENSGACG00000003970	overlap	TRPS1	trichorhinophalangeal syndrome I	-
groupXI	12099500	12102000	42	7	Atlantic	del	ENSGACG00000012637	overlap	SGSM3	small G protein signaling modulator 3	regulation of Rab GTPase activity, protein binding
