# Supplementary material for: Extensive Copy-Number Variation of Young Genes across Stickleback Populations
Source: PLoS Genet. 2014 Dec 4;10(12):e1004830. doi: 10.1371/journal.pgen.1004830 (PMC4256280; doi:10.1371/journal.pgen.1004830)
Supplement: Table S4 — Lineage-specific nuclear genes and other biotypes in the three-spined stickleback genome based on annotations from Ensembl v68. Columns represent total numbers of genes in each category across the autosomal genome, and the number and proportions of genes in each category that are found partially or completely in copy-number variation regions (CNVRs), including deletions (DEL) and duplications (DUP). The numbers and proportion of genes fully overlapping CNVRs that do not overlap segmental duplications (SDs) are reported. The number of gene losses are also reported. (PDF) [file pgen.1004830.s026.pdf]

Supplementary Table 4 - Lineage-specific nuclear genes and other biotypes in the three-spined stickleback genome based on annotations from Ensembl v68. Columns represent total numbers of genes in each category across the autosomal genome, and the number and proportions of genes in each category that are found partially or completely in copy-number variation regions (CNVRs), including deletions (DEL) and duplications (DUP). The numbers and proportion of genes fully overlapping CNVRs that do not overlap segmental duplications (SDs) are reported. The number of Gene Losses are also reported.

		Any Overlap						Complete Overlap						No SD						
		Numbers			Proportions			Numbers			Proportions			Numbers			Proportions			
	**Total**	**CNVRs**	**DEL**	**DUP**	**CNVRs**	**DEL**	**DUP**	**CNVRs**	**DEL**	**DUP**	**CNVRs**	**DEL**	**DUP**	**CNVRs**	**DEL**	**DUP**	**CNVRs**	**DEL**	**DUP**	**Gene Losses**
Protein coding genes	19766	2437	1706	1055	0.123	0.086	0.053	1016	571	552	0.051	0.029	0.028	440	173	275	0.022	0.009	0.014	86
LSG singletons	972	144	97	80	0.148	0.100	0.082	90	49	49	0.093	0.050	0.050	44	22	24	0.045	0.023	0.025	8
LSG LSD	215	62	43	33	0.288	0.200	0.153	53	32	29	0.247	0.149	0.135	14	8	8	0.065	0.037	0.037	9
Non-LSG LSD	1585	665	512	343	0.420	0.323	0.216	545	381	244	0.344	0.240	0.154	113	72	45	0.071	0.045	0.028	58
Non-LSG Paralogs	13158	1263	884	444	0.096	0.067	0.034	228	79	154	0.017	0.006	0.012	188	53	135	0.014	0.004	0.010	8
Non-LSG Singletons	3836	303	170	155	0.079	0.044	0.040	100	30	76	0.026	0.008	0.020	81	18	63	0.021	0.005	0.016	3
RNA genes	1544	179	80	123	0.116	0.052	0.080	174	68	114	0.113	0.044	0.074	52	39	14	0.034	0.025	0.009	7
miRNA	485	62	44	21	0.128	0.091	0.043	61	42	20	0.126	0.087	0.041	21	15	6	0.043	0.031	0.012	4
rRNA	416	21	3	18	0.050	0.007	0.043	20	3	17	0.048	0.007	0.041	4	2	2	0.010	0.005	0.005	0
snoRNA	280	12	3	9	0.043	0.011	0.032	10	1	9	0.036	0.004	0.032	21	16	6	0.075	0.057	0.021	0
snRNA	363	84	30	75	0.231	0.083	0.207	83	22	68	0.229	0.061	0.187	6	6	0	0.017	0.017	0.000	3
Pseudogenes	50	8	7	3	0.160	0.140	0.060	7	6	3	0.140	0.120	0.060	1	1	0	0.020	0.020	0.000	2
