# Supplementary material for: Extensive Copy-Number Variation of Young Genes across Stickleback Populations
Source: PLoS Genet. 2014 Dec 4;10(12):e1004830. doi: 10.1371/journal.pgen.1004830 (PMC4256280; doi:10.1371/journal.pgen.1004830)
Supplement: Table S5 — Putatively lost genes (with an average normalized read depth below 0.25), and number of individuals from each population with the putative loss. Information includes linkage group (LG), start and end positions, Ensembl gene ID, biotype, category based on orthology and paralogy (see Fig. 4 ), the number of individuals (Ind) with the gene loss polymorphism, and the number of individuals from each population (see Fig. 1 ) with the gene loss polymorphism. (PDF) [file pgen.1004830.s027.pdf]

Supplementary Table 5 - Putatively lost genes (with an average normalized read depth below 0.25), and number of individuals from each population with the putative loss. Information includes category (Cat) based on orthology and paralogy (see Fig. 4), number of populations containing the gene loss polymorphism, the number of individuals with the gene loss polymorphism, and for each population (see Fig.1), the number of individuals with the gene loss polymorphism.

LG	Start	End	Gene ID	Biotype	Orthology	Dk_M	G1_R	G1_L	G2_R	G2_L	No_R	No_L	Us_R	Us_L	Ca_R	Ca_L
groupI	313268	314198	ENSGACG00000004606	protein_coding	Non-LSG LSD	4	5	6	5	3	5	5	0	0	5	2
groupI	3022737	3023274	ENSGACG00000006290	protein_coding	Non-LSG LSD	2	0	0	0	0	0	0	0	0	0	0
groupI	10754992	10759116	ENSGACG00000010344	protein_coding	Non-LSG LSD	0	0	0	0	0	2	0	0	0	0	0
groupI	12433155	12433563	ENSGACG00000011322	protein_coding	Non-LSG LSD	0	1	1	1	0	0	0	0	0	0	0
groupI	17406897	17407088	ENSGACG00000022136	snRNA	LSG LSD	1	2	3	5	3	2	2	0	1	0	0
groupII	576241	577205	ENSGACG00000014152	protein_coding	LSG LSD	1	2	4	3	1	2	2	0	0	1	0
groupII	1105861	1107275	ENSGACG00000014232	protein_coding	Non-LSG LSD	4	5	5	4	5	2	3	0	0	0	0
groupII	10998750	10999885	ENSGACG00000015782	protein_coding	Non-LSG LSD	6	6	6	6	6	6	6	0	0	0	0
groupIII	5908387	5908501	ENSGACG00000021792	snRNA	LSG LSD	0	0	2	0	2	0	0	0	0	0	1
groupIII	15085595	15086060	ENSGACG00000017654	protein_coding	Non-LSG LSD	0	0	0	0	0	0	0	0	1	0	1
groupIII	15594828	15596532	ENSGACG00000017700	protein_coding	Para	4	5	3	3	3	5	2	4	2	3	1
groupIII	16775134	16784646	ENSGACG00000018003	protein_coding	Para	5	2	5	4	6	6	4	0	0	3	2
groupIV	16793577	16794725	ENSGACG00000018755	protein_coding	Non-LSG LSD	1	2	0	3	0	0	0	0	0	1	0
groupIV	16797277	16798765	ENSGACG00000018756	protein_coding	Para	1	3	0	3	0	0	0	0	0	1	0
groupIV	23095214	23095958	ENSGACG00000019239	protein_coding	Non-LSG LSD	4	2	3	1	3	4	1	0	0	0	1
groupIV	28145640	28145721	ENSGACG00000021378	miRNA	LSG sing	0	0	0	0	0	1	0	0	0	0	0
groupIV	28179002	28179079	ENSGACG00000021227	miRNA	LSG sing	2	0	0	0	0	1	0	0	0	0	0
groupIV	30659528	30660068	ENSGACG00000019832	protein_coding	Non-LSG LSD	5	1	0	0	2	5	4	0	4	3	1
groupIX	19435465	19454481	ENSGACG00000019775	protein_coding	LSG LSD	0	1	0	2	1	2	2	0	0	0	0
groupIX	19452539	19459616	ENSGACG00000019778	protein_coding	LSG LSD	1	1	1	2	1	2	2	0	1	0	0
groupV	298073	298954	ENSGACG00000002193	protein_coding	Para	0	0	0	0	0	0	0	0	0	2	0
groupV	2282379	2283414	ENSGACG00000003104	protein_coding	Non-LSG LSD	1	3	0	4	1	6	5	6	5	1	4
groupV	2818156	2820505	ENSGACG00000003300	protein_coding	Non-LSG LSD	0	0	0	1	1	0	0	0	0	1	5
groupVI	15480971	15482728	ENSGACG00000011609	protein_coding	Non-LSG LSD	0	0	0	0	0	1	0	0	0	0	0
groupVII	518791	520048	ENSGACG00000018668	protein_coding	Para	2	4	2	3	2	4	0	1	1	1	0
groupVII	1079750	1092181	ENSGACG00000018782	protein_coding	LSG sing	4	5	5	5	3	6	5	3	1	5	1
groupVII	3010046	3032872	ENSGACG00000019210	protein_coding	LSG sing	4	1	3	3	1	0	1	3	6	0	1
groupVII	9056616	9058716	ENSGACG00000019965	protein_coding	Non-LSG LSD	6	6	2	5	4	5	3	2	2	1	3
groupVII	9109075	9111092	ENSGACG00000019969	protein_coding	Non-LSG LSD	1	1	2	3	2	4	1	4	3	0	0
groupVIII	4797418	4801222	ENSGACG00000005415	protein_coding	Non-LSG LSD	3	3	3	4	4	5	5	1	1	0	0

groupX	891778	894040	ENSGACG00000001941	protein_coding	Non-LSG LSD	2	3	3	4	5	3	0	1	1	1	1
groupX	1148456	1148636	ENSGACG00000001979	protein_coding	Non-LSG LSD	1	0	0	0	0	0	0	0	3	1	0
groupX	12654371	12658742	ENSGACG00000008242	protein_coding	Non-LSG LSD	0	0	3	6	2	0	0	3	1	0	1
groupX	12683621	12684212	ENSGACG00000008250	protein_coding	Non-LSG LSD	6	6	5	6	5	0	1	3	1	0	3
groupX	13219610	13223472	ENSGACG00000008831	protein_coding	Non-LSG LSD	0	0	0	0	0	0	0	5	5	0	0
groupX	13226407	13227888	ENSGACG00000008840	protein_coding	LSG LSD	5	0	0	0	0	0	1	0	0	0	0
groupX	13227912	13229965	ENSGACG00000008842	protein_coding	Sing	5	0	0	0	0	0	1	0	0	0	0
groupX	14400506	14401518	ENSGACG00000009322	protein_coding	Non-LSG LSD	0	4	1	1	2	1	0	0	0	0	1
groupX	14405020	14405560	ENSGACG00000009325	protein_coding	Non-LSG LSD	0	4	1	1	2	1	0	1	1	0	1
groupX	14407419	14408582	ENSGACG00000009327	protein_coding	Non-LSG LSD	0	3	1	1	2	1	0	1	1	0	1
groupX	14618821	14619983	ENSGACG00000009497	protein_coding	Non-LSG LSD	4	1	4	4	3	0	2	0	1	1	0
groupX	14621895	14623873	ENSGACG00000009499	protein_coding	Non-LSG LSD	3	1	4	4	3	0	2	0	1	1	0
groupXI	4320058	4324681	ENSGACG00000007447	protein_coding	Non-LSG LSD	2	4	6	6	5	4	5	4	2	0	1
groupXI	4446709	4449166	ENSGACG00000007451	protein_coding	Non-LSG LSD	2	6	6	6	6	5	6	3	0	0	0
groupXI	4496074	4496580	ENSGACG00000007452	pseudogene	Sing	1	6	6	6	6	5	6	3	0	0	0
groupXI	4500951	4505292	ENSGACG00000007454	protein_coding	Non-LSG LSD	1	6	6	6	6	5	6	3	0	0	0
groupXI	9650420	9654080	ENSGACG00000011265	protein_coding	Non-LSG LSD	0	0	0	0	0	0	0	2	0	0	1
groupXI	9794034	9795077	ENSGACG00000011276	protein_coding	Para	0	0	2	0	0	0	0	0	0	0	0
groupXI	10220642	10222287	ENSGACG00000011417	protein_coding	LSG sing	0	6	4	4	4	0	0	3	4	0	1
groupXI	12398198	12399317	ENSGACG00000012790	protein_coding	Non-LSG LSD	5	5	6	6	4	5	5	3	0	1	1
groupXI	15616018	15617314	ENSGACG00000014557	protein_coding	Non-LSG LSD	1	1	0	4	2	1	0	1	0	0	0
groupXII	16461684	16464104	ENSGACG00000012433	protein_coding	Non-LSG LSD	0	0	0	0	0	0	0	1	0	2	5
groupXIII	7070566	7075041	ENSGACG00000007399	protein_coding	Sing	0	4	0	0	0	0	0	0	0	0	0
groupXIII	18539982	18541069	ENSGACG00000014282	protein_coding	LSG LSD	6	6	5	6	5	6	6	3	1	1	3
groupXIII	18748570	18752248	ENSGACG00000014305	protein_coding	Non-LSG LSD	1	4	3	3	5	2	2	0	0	0	0
groupXIV	7842402	7845250	ENSGACG00000017452	protein_coding	LSG LSD	2	0	0	2	3	2	3	0	2	0	0
groupXIV	14271041	14272180	ENSGACG00000018423	protein_coding	Non-LSG LSD	1	1	2	1	4	2	2	0	0	0	0
groupXV	9778276	9790801	ENSGACG00000010720	protein_coding	Para	4	1	2	6	2	5	4	1	0	2	0
groupXVI	4234862	4236981	ENSGACG00000002206	protein_coding	Non-LSG LSD	2	6	4	6	4	0	0	0	0	0	0
groupXVI	4252437	4253460	ENSGACG00000002211	protein_coding	Non-LSG LSD	0	0	0	0	0	0	0	0	0	2	3
groupXVI	4293606	4294530	ENSGACG00000002214	protein_coding	Non-LSG LSD	0	0	0	0	0	0	1	1	0	2	1
groupXVI	4301584	4303257	ENSGACG00000002215	protein_coding	Non-LSG LSD	0	0	0	0	0	0	1	1	0	2	1
groupXVI	4784786	4787328	ENSGACG00000002255	protein_coding	Para	0	1	0	2	0	0	0	1	0	0	0
groupXVI	4814441	4815533	ENSGACG00000002259	protein_coding	Non-LSG LSD	0	2	5	5	2	2	1	1	1	3	0
groupXVI	13896722	13897679	ENSGACG00000006973	protein_coding	Non-LSG LSD	0	0	0	5	0	0	0	1	0	0	0

groupXVI	13901515	13901815	ENSGACG00000006974	protein_coding	LSG LSD	0	0	0	1	0	0	0	0	0	0	1
groupXVI	17028080	17029718	ENSGACG00000008551	protein_coding	Non-LSG LSD	1	3	5	4	2	1	0	1	2	5	0
groupXVI	17216675	17216755	ENSGACG00000022535	miRNA	LSG sing	0	0	0	2	2	0	0	0	0	0	0
groupXVII	6514459	6515044	ENSGACG00000007607	protein_coding	Non-LSG LSD	2	4	3	3	3	1	1	0	0	0	0
groupXVII	11175540	11184052	ENSGACG00000010910	protein_coding	LSG sing	0	2	1	1	0	4	1	0	3	0	0
groupXVIII	13185259	13186039	ENSGACG00000012073	protein_coding	Non-LSG LSD	0	2	0	0	0	0	0	1	1	5	5
groupXVIII	15820422	15820995	ENSGACG00000013545	protein_coding	Non-LSG LSD	1	0	0	1	2	0	2	0	0	0	0
groupXX	13534108	13540477	ENSGACG00000012354	protein_coding	LSG sing	4	4	5	5	4	1	3	1	2	4	0
groupXX	13537537	13543688	ENSGACG00000012355	protein_coding	LSG sing	2	0	2	0	1	0	1	1	1	0	0
groupXX	13545827	13546986	ENSGACG00000012356	protein_coding	Non-LSG LSD	1	0	2	0	1	0	1	1	1	0	0
groupXX	14209795	14211021	ENSGACG00000012600	protein_coding	LSG sing	0	0	1	0	0	0	0	0	0	0	0
groupXXI	3413507	3415036	ENSGACG00000002237	pseudogene	Sing	6	6	5	6	5	5	5	1	3	3	2
scaffold_137	45887	47060	ENSGACG00000017732	protein_coding	LSG LSD	0	0	1	0	0	1	1	0	0	0	0
scaffold_137	96946	97474	ENSGACG00000017734	protein_coding	Non-LSG LSD	1	0	4	0	2	4	1	1	1	6	5
scaffold_137	101655	102467	ENSGACG00000017736	protein_coding	LSG LSD	1	0	4	0	1	4	1	1	2	6	5
scaffold_178	22787	24269	ENSGACG00000001724	protein_coding	Non-LSG LSD	0	0	0	1	2	0	0	0	0	0	0
scaffold_198	75484	76330	ENSGACG00000000194	protein_coding	Non-LSG LSD	0	0	1	0	0	0	0	0	0	0	0
scaffold_229	61941	62349	ENSGACG00000000549	protein_coding	Non-LSG LSD	0	0	0	0	0	0	0	1	0	0	0
scaffold_253	38409	42330	ENSGACG00000000918	protein_coding	Non-LSG LSD	0	0	0	0	0	0	0	0	0	4	0
scaffold_290	58570	59630	ENSGACG00000000780	protein_coding	LSG sing	0	0	0	0	0	0	0	0	1	0	0
scaffold_327	90	8820	ENSGACG00000014758	protein_coding	Non-LSG LSD	0	0	0	0	1	0	0	0	0	0	0
scaffold_327	11415	11506	ENSGACG00000022829	miRNA	LSG sing	0	0	0	0	1	0	0	0	0	0	0
scaffold_332	23002	24274	ENSGACG00000001264	protein_coding	Sing	0	0	0	0	1	0	0	0	0	0	0
scaffold_332	25355	28790	ENSGACG00000001267	protein_coding	Non-LSG LSD	0	0	0	0	1	0	0	0	0	0	0
scaffold_37	815922	817008	ENSGACG00000000904	protein_coding	Non-LSG LSD	0	0	0	0	0	2	0	0	0	0	0
scaffold_54	712866	712972	ENSGACG00000021096	snRNA	LSG sing	0	0	1	0	0	1	0	0	0	0	0
scaffold_56	1109846	1110753	ENSGACG00000002169	protein_coding	Non-LSG LSD	1	3	2	3	1	0	1	1	1	2	0
scaffold_56	1117520	1124196	ENSGACG00000002171	protein_coding	Non-LSG LSD	1	3	2	3	1	0	1	1	1	0	0
scaffold_67	618744	619392	ENSGACG00000000591	protein_coding	Non-LSG LSD	0	0	0	0	0	0	0	6	4	2	4
scaffold_95	93512	94037	ENSGACG00000000077	protein_coding	Non-LSG LSD	4	0	1	5	3	0	0	1	5	3	1
