# Supplementary material for: Extensive Copy-Number Variation of Young Genes across Stickleback Populations
Source: PLoS Genet. 2014 Dec 4;10(12):e1004830. doi: 10.1371/journal.pgen.1004830 (PMC4256280; doi:10.1371/journal.pgen.1004830)
Supplement: Table S6 — Annotated gene names from the top nr BLASTx hits of unmapped reads using Blast2GO, and also from BLASTx for genes without blast2GO annotations. (PDF) [file pgen.1004830.s028.pdf]

Supplementary Table 6 - Annotated gene names from the top nr BLASTx hits of unmapped reads using Blast2GO, and also from BLASTx for genes without blast2GO annotations

Blast2GO	BLASTx
1-phosphatidylinositol- -bisphosphate phosphodiesterase gamma-1-like	1-phosphatidylinositol-4,5-bisphosphate phosphodiesterase gamma-1
39s ribosomal protein mitochondrial	28S ribosomal protein S9, mitochondrial
a-kinase anchor protein 6	50 kDa midgut protein
a-kinase anchor protein 9	5-hydroxytryptamine receptor 2C
acetylcholine receptor subunit delta-like	ABC transporter G family member 20
actin-binding rho-activating protein	acid phosphatase protein 2
adenylate cyclase type 8-like	adapter protein CIKS
alpha-2,8-sialyltransferase 8e isoform 1	adenylate cyclase type 8
androgen-induced gene 1	adenylate kinase 8
apoptotic protease-activating factor 1-like	a-kinase anchor protein 6
arf-gap with ank repeat and ph domain-containing protein 1	alpha-2-macroglobulin protein 1
arf-gap with ank repeat and ph domain-containing protein 3	amino-terminal enhancer of split
aristaless related homeobox	anthrax toxin receptor 2
aspartyl asparaginyl beta-hydroxylase	AP-3 complex subunit beta-1
atp-dependent rna helicase a	arf-GAP domain and FG repeats-containing protein 1
atrial natriuretic peptide-converting enzyme-like	arf-GAP with GTPase, ANK repeat and PH domain-containing protein 1
bah and coiled-coil domain-containing protein 1	arf GTPase-activating protein GIT1
bartter with sensorineural deafness	atrial natriuretic peptide-converting enzyme
beta-1,4-mannosyl-glycoprotein 4-beta-n-acetylglucosaminyltransferase	attractin
beta-adrenergic receptor kinase 2	BAH and coiled-coil domain-containing protein 1
beta-citryl-glutamate synthase b	BCL-6 corepressor
beta-lactamase	beta-1,4-mannosyl-glycoprotein 4-beta-N-acetylglucosaminyltransferase
bmp-2-inducible protein kinase	beta-adrenergic receptor kinase 2
bmp-binding endothelial regulator protein	beta-citryl-glutamate synthase B
breast cancer anti-estrogen resistance 1	breast cancer anti-estrogen resistance protein 1
c-maf-inducing protein	BRICHOS domain-containing protein C16orf79
calcium-activated potassium channel subunit beta-4	cadherin-24
calcium-transporting atpase type 2c member 1	calmodulin-binding transcription activator 2
capsid protein f	CAP-Gly domain-containing linker protein 2
carboxypeptidase m	caskin-2
cell adhesion molecule 4	Cdc4p
centrosomal protein of 120 kda	cell adhesion molecule 3
cgmp-dependent protein kinase 2	centrosomal protein of 120 kDa
checkpoint protein hus1	chordin

cholecystokinin
chordin
chromosome 10 open reading frame 65
cip1-interacting zinc finger protein
comm domain-containing protein 1
core histone macro-H2A.1 isoform 1
cyclic amp-dependent transcription factor atf-5
cytokine receptor-like factor 3
cytoplasmic polyadenylation element-binding protein 1
dedicator of cytokinesis protein 3
deoxycytidine kinase
diacylglycerol kinase delta
diacylglycerol kinase eta
dna damage-binding protein 1
e3 ubiquitin-protein ligase znrf1
endothelin-converting enzyme-like 1-like
enterohemolysin 2
exocyst complex component 7
extracellular matrix protein fras1
fibroblast growth factor receptor 3
formin-binding protein 1
g protein-coupled receptor kinase 1-like
g protein-coupled receptor kinase type 2
g2 mitotic-specific cyclin-b3
gag-pol precursor polyprotein
glutathione s-transferase theta-1
growth differentiation factor 5
hepatic leukemia factor-like
histone-lysine n-methyltransferase prdm6
homeobox protein hox-b6
homeobox protein nkx-
immunoglobulin superfamily member 4b
inositol hexakisphosphate and diphosphoinositol-pentakisphosphate kinase 1
insulin-degrading enzyme
intraflagellar transport protein 122 homolog
kinase d-interacting substrate of 220 kda
krueppel-like factor 8-like
chorion-specific transcription factor GCMb
chromodomain-helicase-DNA-binding protein 3
C-myc promoter-binding protein
coiled-coil domain containing 57
coiled-coil domain-containing protein 120
Collectrin precursor
COMM domain containing 10
complement C5
coronin-7
CR1-3
craniofacial development protein 1
Cysteine and histidine-rich domain-containing protein 1
cytohesin-4
dachshund 2
dachshund protein
DC-STAMP domain-containing protein 1
DC-STAMP domain-containing protein 2
dedicator of cytokinesis protein 11
dedicator of cytokinesis protein 3
disabled 1
disco-interacting protein 2 C
DNA damage-binding protein 1
DNA ligase 1
dysferlin
E3 ubiquitin-protein ligase pellino 1
EH domain-binding protein 1
EMILIN-3
endonuclease-reverse transcriptase
ephrin-A2
ER degradation-enhancing alpha-mannosidase 3
Ewing tumor-associated antigen 1
excitatory amino acid transporter 2
extracellular matrix protein FRAS1
FAM131A
FAM184B
FAM65C
far upstream element-binding protein 3

laminin subunit alpha-1
low quality protein: protein prrc2c-like
melanoma-associated antigen g1
metal transporter cnnm4-like
metalloendopeptidase mitochondrial-like
mitochondrial ribosomal protein l1
mitochondrial ribosomal protein s9
mitogen-activated protein kinase kinase kinase 7 interacting protein 1
myosin x
n-acetylaspartyl-glutamate synthetase a
neural retina-specific leucine zipper protein
neuritin precursor
neurofibromin isoform 1
neuronal pas domain-containing protein 3
neutral ceramidase
neutrophil cytosolic factor 4
novel protein vertebrate nebulin
novel protein vertebrate protein disulfide isomerase family member 5
nuclear factor 1 b-type-like
nucleoside diphosphate-linked moiety x motif 18
oma1 zinc metallopeptidase (cerevisiae)
otu domain-containing protein 5-like
p protein
par-3 partitioning defective 3 homolog (elegans)
peptidyl-prolyl cis-trans isomerase cwc27 homolog
phosphatidylinositol 3-kinase catalytic subunit type 3
phosphoribosyl pyrophosphate amidotransferase
phosphoribosylaminoimidazole succinocarboxamide synthetase
phosphoribosylformylglycinamidine synthase
platelet endothelial aggregation receptor 1
plexin d1
pol polyprotein
pou class transcription factor 3-b isoform 2
probable 4-hydroxy-2-oxoglutarate mitochondrial-like
probable atp-dependent rna helicase ddx20
protein casc3-like
protein efr3 homolog a
FCH domain only protein 2
fibronectin type III domain-containing protein 3B
fibronectin type III domain-containing protein 4
follistatin-related protein 4
Forkhead box G1
forkhead box protein N3
glutamate receptor-interacting protein 1
G protein-coupled receptor kinase 5
growth inhibition and differentiation-related protein 88
guanine nucleotide exchange factor VAV3
heme-binding protein 1
hepatocyte nuclear factor 3-beta
Histone acetyltransferase MYST3
Histone-lysine N-methyltransferase PRDM6
Homeobox protein Meis2
homocysteine-responsive ER-resident ubiquitin domain member 1
Immunoglobulin G-binding protein A
IMPACT
inositol hexakisphosphate and diphosphoinositol-pentakisphosphate kinase 1 isoform 2
insulin-degrading enzyme
insulin growth factor binding protein 3
interleukin-17A/F-2
interleukin-1 receptor type II
intraflagellar transport protein 122
intraflagellar transport protein 27
IQ motif and SEC7 domain-containing protein 3
Jade-2
jagged-2
kelch protein 29
KH domain-containing, RNA-binding, signal transduction-associated protein 1
kinase D-interacting substrate of 220 kDa
kinesin protein KIF1C
Krueppel factor 5
kyphoscoliosis peptidase
laminin subunit alpha-1
latrophilin-2
LOC494675 protein

protein pthb1-like
protein sec13 homolog
protein spinster homolog 2-like
protein sprouty homolog 1-like
rap guanine nucleotide exchange factor 2
rap guanine nucleotide exchange factor 6 isoform 2
ras gtpase-activating protein ngap
ras protein activator like 2
ras suppressor protein 1
ras-associated and pleckstrin homology domains-containing protein 1
ras-related protein rab-21
ras-specific guanine nucleotide-releasing factor 2-like
regulating synaptic membrane exocytosis protein 2 isoform b
replication-associated protein partial
retinoic acid receptor alpha isoform 1
retrotransposable element tf2 155 kda protein type 1-like
retrotransposon-like family member (retr-1)-like
rho guanine nucleotide exchange factor 1
ribonuclease 3
ring finger protein 114
sam and sh3 domain-containing protein 1
sec1 family domain-containing protein 1
sec1 family domain-containing protein 2
seizure related 6 homolog -like
serine threonine-protein kinase mrck alpha
sncaip protein
sodium hydrogen exchanger 5-like
sortilin-related receptor
staphylococcal nuclease domain containing 1
succinate dehydrogenase
suppression of tumorigenicity 7
synphilin-1 isoform 1
t cell-specific transcription factor isoform c
testican-3 isoform 1
tgf-beta-activated kinase 1 and map3k7-binding protein 1-like
thyrotropin-releasing hormone-degrading ectoenzyme
toll-interacting protein
LON peptidase N-terminal domain and RING finger protein 2
MAP7 domain-containing protein 2
MAP kinase-activating death domain protein
melanophilin
metalloendopeptidase OMA1, mitochondrial
mitochondrial carnitine/acylcarnitine carrier protein
mitochondrial sodium/hydrogen exchanger NHA2
mitotic checkpoint serine/threonine-protein kinase BUB1
multiple C2 and transmembrane domain-containing protein 1
myelin protein zero protein 1
myosin-X
myosin-XVIIIb
NACHT, LRR and PYD domains-containing protein 12
neogenin
nephrocystin-1
neural cell adhesion molecule L1
neuronal PAS domain-containing protein 3
neutrophil cytosolic factor 4
novel protein similar to vertebrate enabled (ENAH)
novel protein similar to vetebrate diacylglycerol kinase, delta 130kDa (DGKD)
nuclear factor 1 A-type
nuclear factor 1 X-type
nuclear factor 7 ovary
nuclear receptor corepressor 2
osteopotentia
otogelin
OTU domain-containing protein 4
p40phox protein
palladin
parathyroid hormone-responsive B1 isoform 3
partitioning defective 3
perilipin-2
phosphatidylinositol-4-phosphate 3-kinase C2 domain-containing subunit beta
phosphatidylinositol-binding clathrin assembly protein
phosphatidylserine synthase 2
phosphofurin acidic cluster sorting protein 1
plexin-B1

transcription factor 7-like 2
transcription factor gata-3
transcription factor sox-13
transcription factor sox-5
transmembrane protein 145
transmembrane protein 8c-like
transposable element tcb1 transposase
transposase
tryptophanyl-trna mitochondrial-like
ubiquitin carboxyl-terminal hydrolase 24
vesicle-associated membrane protein 4
vitellogenin
voltage-dependent p q-type calcium channel subunit alpha-1a
voltage-dependent t-type calcium channel subunit alpha-1i-like
wd repeat domain isoform cra_b
wd repeat-containing and planar cell polarity effector protein fritz homolog
wd repeat-containing protein 3
wdhd1 protein
zinc finger and btb domain-containing protein 46
zinc finger e-box-binding homeobox 2-like isoform 2
zinc finger protein 536
zinc ring finger protein 3
zinc transporter slc39a7
plexin-D1
pol polyprotein
polypeptide N-acetylgalactosaminyltransferase 10
polypeptide N-acetylgalactosaminyltransferase 3
polyphosphate kinase 2 family
potassium voltage-gated channel subfamily A member 4
potassium voltage-gated channel subfamily D member 1
POU domain class 2-associating factor 1
P protein
probable E3 ubiquitin-protein ligase HERC1
probable G-protein coupled receptor 153
probable RNA-binding protein 46
progesterone-induced-blocking factor 1
protocadherin-15
proto-oncogene Wnt-3
PTHB1
pumilio 2
purine-rich element-binding protein gamma
ralBP1-associated Eps domain-containing protein 2
rap guanine nucleotide exchange factor 6
ras GTPase-activating protein nGAP
ras guanine nucleotide exchange factor F
recombining binding protein suppressor of hairless protein
retrotransposable element Tf2 155 kDa protein type 1
RETRotransposon family member (retr-1)
retrovirus-related Pol polyprotein LINE-1
reverse transcriptase
rho GTPase-activating protein 17
rho GTPase-activating protein 6
ribonuclease 3
RING finger protein 214
RNA binding protein fox-1 2
RRNAD1
S-antigen protein
sec1 family domain-containing protein 1
sec1 family domain-containing protein 2
senescence-associated protein

sentrin-specific protease 7
serine/threonine-protein kinase BRSK2
serine/threonine-protein kinase ULK4
serine/threonine-protein kinase WNK1
SH2B adapter protein 1
SH2 domain-containing protein 5
SH3 and multiple ankyrin repeat domains protein 2
SH3 and multiple ankyrin repeat domains protein 3
shisa-6
si:dkey-100n23.5
slit 2 protein
snRNA-activating protein complex subunit 4
sodium channel protein type 3 subunit alpha
sodium/hydrogen exchanger 1
sodium/hydrogen exchanger 5 precursor
solute carrier family 35 member D3
solute carrier family 35 member F3 isoform 2
sortilin-related receptor
SPARC-related modular calcium-binding protein 2
Spermatogenesis-associated protein 2
stromelysin-3
synphilin-1
T-box transcription factor TBX15
T-cell leukemia homeobox protein 1
T-cell receptor alpha chain V region 2B4 precursor
tetratricopeptide repeat protein 16
TGF-beta-activated kinase 1 and MAP3K7-binding protein 1
thymus-specific serine protease
thyroglobulin
thyroid adenoma-associated protein
thyrotropin-releasing hormone-degrading ectoenzyme
toll-interacting protein
toll-interacting protein A
tomoregulin-1
TOX high mobility group box family member 3
transcription factor 7 2
transmembrane and TPR repeat-containing protein 2

transmembrane protein 145
tRNA-specific adenosine deaminase 1
TSSC1
tumor necrosis factor ligand superfamily member 10
tumor necrosis factor ligand superfamily member 11
tumor protein 63 isoform 2
tumor protein p53-inducible nuclear protein 2
ubinuclein-1
ubiquitin-conjugating enzyme E2, J1
UPF0249 protein ydjC
vacuolar protein sorting-associated protein 13B
VHSV-induced protein
voltage-dependent L-type calcium channel subunit alpha-1F
voltage-dependent L-type calcium channel subunit beta-3
voltage-dependent P/Q-type calcium channel subunit alpha-1A
voltage-gated potassium channel subunit beta-3
WD repeat-containing and planar cell polarity effector protein fritz
wiskott-Aldrich syndrome protein family member 3
xin actin-binding repeat-containing protein 2
zinc finger and BTB domain-containing protein 46
zinc finger E-box-binding homeobox 2 isoform 1
zinc finger HIT domain-containing protein 1
zinc finger MYM-type protein 1
zinc finger protein 313
zinc finger protein 704
zinc finger protein 865
zinc ring finger protein 3
Zygotic DNA replication licensing factor mcm6-B
