# Supplementary material for: Extensive Copy-Number Variation of Young Genes across Stickleback Populations
Source: PLoS Genet. 2014 Dec 4;10(12):e1004830. doi: 10.1371/journal.pgen.1004830 (PMC4256280; doi:10.1371/journal.pgen.1004830)
Supplement: Table S7 — Number of CNVs, CNV genes and gene losses for each population and individual within the population. (PDF) [file pgen.1004830.s029.pdf]

Supplementary Table 7 - Number of CNVs, CNV genes and gene losses for each population and individual

	CNVs		CNV Genes		
ID	Deletions	Duplications	Deletions	Duplications	Gene losses
**Dk_M**	**1087**	**200**	**273**	**188**	**52**
BS25	498	61	140	55	26
BS26	476	64	136	77	29
BS27	423	81	97	70	24
BS28	263	61	78	50	14
BS29	375	61	99	43	23
BS30	449	71	119	81	25
**G1_R**	**1182**	**137**	**272**	**119**	**51**
BS1	531	57	95	46	25
BS3	517	69	143	60	31
BS5	363	55	78	39	20
BS7	530	53	110	40	30
BS9	526	57	116	38	28
BS11	550	53	146	64	35
**G1_L**	**1216**	**169**	**270**	**154**	**54**
BS2	648	74	162	57	33
BS4	437	63	97	55	26
BS6	366	51	85	57	25
BS8	467	64	115	51	32
BS10	635	49	132	33	33
BS12	489	62	123	51	26
**G2_R**	**1181**	**191**	**303**	**156**	**57**
BS13	512	74	167	69	36
BS15	532	51	115	36	31
BS17	485	72	123	58	33
BS19	523	54	126	47	32
BS21	493	72	166	63	39
BS23	560	67	140	49	35
**G2_L**	**1052**	**189**	**296**	**165**	**60**
BS14	438	54	120	43	34
BS16	510	67	150	42	28
BS18	477	69	134	47	31
BS20	442	64	126	79	29
BS22	361	67	98	31	23
BS24	479	44	101	26	25
**No_R**	**963**	**98**	**254**	**128**	**47**
BS55	441	44	75	47	20
BS57	560	51	128	63	32
BS59	404	50	116	41	29
BS61	457	43	102	44	28
BS63b	562	49	100	47	21
BS65	383	47	117	42	20
**No_L**	**937**	**121**	**254**	**136**	**47**
BS56	287	32	70	42	17
BS58b	455	44	118	31	17
BS60	290	40	73	41	20
BS62	491	50	120	32	22
BS64b	415	39	112	40	27

BS66	428	49	88	62	24
**Us_R**	**930**	**165**	**187**	**152**	**43**
BS31b	215	48	13	55	5
BS33	364	55	62	28	19
BS35	344	52	52	50	10
BS37	213	60	52	46	13
BS39	311	44	91	29	26
BS41	334	58	49	58	15
**Us_L**	**785**	**191**	**196**	**202**	**40**
BS32b	271	57	44	62	8
BS34b	231	54	55	49	15
BS36b	278	58	47	45	8
BS38b	165	56	47	43	17
BS40b	364	65	62	56	11
BS42b	262	55	96	69	22
**Ca_R**	**859**	**141**	**150**	**141**	**35**
BS43	336	55	39	49	13
BS45	353	58	37	54	13
BS47	235	51	40	50	8
BS49	435	60	62	63	16
BS51	273	80	70	62	19
BS53	401	57	49	43	18
**Ca_L**	**854**	**143**	**151**	**134**	**34**
BS44	194	58	45	32	10
BS46	305	41	45	28	8
BS48	240	54	42	64	12
BS50b	376	47	60	37	20
BS52b	433	63	44	55	10
BS54	358	41	41	30	11
