# Supplementary material for: Extensive Copy-Number Variation of Young Genes across Stickleback Populations
Source: PLoS Genet. 2014 Dec 4;10(12):e1004830. doi: 10.1371/journal.pgen.1004830 (PMC4256280; doi:10.1371/journal.pgen.1004830)
Supplement: Table S8 — Gene ontology (GO) enrichment analysis among CNV genes (genes that are entirely overlapping CNVRs). The number of genes from each GO category and the number of genes completely overlapping CNVRs, including deletions and duplications reported separately, with significantly overrepresented categories in bold. (PDF) [file pgen.1004830.s030.pdf]

Supplementary Table 8 - Gene ontology (GO) enrichment analysis among CNV genes (genes that are entirely overlapping CNVRs). The number of genes from each GO category and the number of genes completely overlapping CNVRs, including deletions and duplications reported separately, with significantly overrepresented categories in bold. Enrichment FDR-corrected p-values (from the weighted scheme from topGO) are reported.

**GO category**	**GO id**	**Annotated**	CNVRs	p-value	DEL	p-value	DUP	p-value	BOTH	p-value	DEL non-singleton	p-value	DUP non-singleton	p-value	DEL singleton	p-value	DUP singleton	p-value
Biological Process																		
G-protein coupled receptor protein signaling pathway	GO:0007186	719	**91**	**5.9E-21**	**56**	**1.6E-14**	**45**	**4.71E-07**	12	0.18862	**43**	**6.99E-11**	**31**	**1.03E-06**	13	0.6022	14	1
antigen processing and presentation	GO:0019882	32	**18**	**2.1E-14**	**17**	**6.6E-17**	**9**	**2.11E-05**	**8**	**9.06E-09**	**14**	**8.08E-14**	2	1	3	0.90918	**7**	**3.23E-05**
protein ubiquitination	GO:0016567	111	**30**	**2.4E-14**	**14**	**0.00011**	**15**	**2.11E-05**	1	1	**12**	**0.00018**	3	1	2	1	**12**	**2.33E-06**
protein glycosylation	GO:0006486	82	**18**	**2.1E-06**	**15**	**2.4E-07**	9	0.05089	**6**	**0.00406**	**13**	**8.08E-07**	6	0.54339	2	1	3	1
immune response	GO:0006955	101	**19**	**9.1E-06**	**17**	**8E-08**	10	0.05089	**8**	**6.66E-05**	**14**	**8.22E-07**	2	1	3	1	**8**	**0.00682**
transposition	GO:0006313	24	6	0.15824	**6**	**0.00629**	1	1	1	1	**5**	**0.02158**	1	1	1	1	0	1
Cellular Component																		
ubiquitin ligase complex	GO:0000151	117	**31**	**6.6E-14**	15	**4.3E-05**	**15**	**0.00018**	1	1	**12**	**0.00045**	3	1	3	1	**12**	**4.38E-06**
MHC class I protein complex	GO:0042612	20	**11**	**3.8E-08**	**10**	**4.7E-09**	4	0.15872	3	0.06317	**10**	**3.27E-10**	2	1	0	1	2	1
integral to membrane	GO:0016021	1676	**117**	**2.6E-05**	**68**	**0.0024**	65	0.15872	17	1	**55**	**0.00543**	**44**	**0.01575**	13	1	21	1
MHC class II protein complex	GO:0042613	12	**7**	**2.7E-05**	**7**	**8.3E-07**	**5**	**0.0019**	**5**	**3.52E-06**	**4**	**0.00611**	0	**1**	**3**	**0.01657**	**5**	**3.40E-05**
integral to Golgi membrane	GO:0030173	36	7	0.0884	4	0.85222	**6**	**0.04833**	3	0.24473	4	**0.40708**	3	1	0	1	3	1
Molecular Function																		
ubiquitin-protein ligase activity	GO:0004842	117	**30**	**2.1E-12**	**14**	**0.00102**	**25**	**8.23E-05**	1	1	**12**	**0.00147**	3	1	2	1	**22**	**2.31E-06**
fucosyltransferase activity	GO:0008417	17	**10**	**2.3E-07**	**10**	**3E-09**	3	1	3	0.16605	**8**	**7.12E-07**	3	1	2	1	0	1
hydrolase activity, acting on acid anhydrides	GO:0016817	755	**26**	**2.3E-05**	**8**	**0.00021**	19	0.16715	2	1	**8**	**5.59E-05**	12	1	0	1	7	1
transposase activity	GO:0004803	24	6	0.15143	**6**	**0.00748**	1	1	1	1	**5**	**0.02698**	1	1	1	1	0	1
protein binding	GO:0005515	5812	280	0.93439	**192**	**0.00319**	118	1	35	1	**156**	**0.00052**	60	1	36	1	58	1
