# Supplementary material for: Extensive Copy-Number Variation of Young Genes across Stickleback Populations
Source: PLoS Genet. 2014 Dec 4;10(12):e1004830. doi: 10.1371/journal.pgen.1004830 (PMC4256280; doi:10.1371/journal.pgen.1004830)
Supplement: Table S9 — Gene ontology (GO) enrichment analysis among stickleback LSDs. (PDF) [file pgen.1004830.s031.pdf]

Supplementary Table 9 - Gene ontology (GO) enrichment analysis among stickleback LSDs (Lineage-Specific Duplications)

GO category	GO id	Annotated	LSDs	Expected	Fisher p-value	Weighted p-value
Biological Process						
G-protein coupled receptor protein signalling pathway	GO:0007186	719	137	45.88	2.0E-30	5.3E-32
protein ubiquitination	GO:0016567	111	51	7.08	8.1E-29	9.4E-29
transposition, DNA-mediated	GO:0006313	24	23	1.53	2.8E-24	5.5E-24
antigen processing and presentation	GO:0019882	32	26	2.04	1.5E-23	2.7E-23
proteolysis involved in cellular protein catabolic process	GO:0051603	124	29	7.91	1.7E-07	5.2E-21
immune response	GO:0006955	101	27	6.45	3.0E-08	5.0E-08
protein glycosylation	GO:0006486	82	22	5.23	1.1E-06	2.3E-06
nucleosome assembly	GO:0006334	127	26	8.1	1.1E-05	3.6E-05
Cellular Component						
ubiquitin ligase complex	GO:0000151	117	52	8.87	1.3E-25	3.7E-27
proteasome core complex	GO:0005839	46	26	3.49	7.9E-16	1.2E-15
MHC class I protein complex	GO:0042612	20	17	1.52	6.0E-15	9.3E-15
MHC class II protein complex	GO:0042613	12	9	0.91	6.6E-07	1.5E-06
nucleosome	GO:0000786	123	26	9.33	3.9E-05	1.0E-04
integral to membrane	GO:0016021	1676	177	127.12	8.4E-06	1.7E-04
integral to Golgi membrane	GO:0030173	36	11	2.73	1.1E-03	2.7E-03
Molecular Function						
ubiquitin-protein ligase activity	GO:0004842	117	52	8.7	3.3E-25	4.0E-25
transposase activity	GO:0004803	24	23	1.78	8.2E-23	1.2E-22
threonine-type endopeptidase activity	GO:0004298	46	26	3.42	8.0E-16	1.9E-15
fucosyltransferase activity	GO:0008417	17	11	1.26	4.3E-07	9.6E-07
DNA binding	GO:0003677	1365	110	101.45	1.0E+00	4.1E-06
hydrolase activity, acting on acid anhydrides	GO:0016817	755	39	56.11	1.0E+00	2.7E-04
electron carrier activity	GO:0009055	148	28	11	3.7E-04	6.8E-04
endopeptidase inhibitor activity	GO:0004866	65	14	4.83	1.7E-02	8.5E-04
sialyltransferase activity	GO:0008373	35	11	2.6	2.7E-03	4.1E-03
heme binding	GO:0020037	126	23	9.36	4.2E-03	6.2E-03
