# Supplementary material for: Extensive Copy-Number Variation of Young Genes across Stickleback Populations
Source: PLoS Genet. 2014 Dec 4;10(12):e1004830. doi: 10.1371/journal.pgen.1004830 (PMC4256280; doi:10.1371/journal.pgen.1004830)
Supplement: Table S10 — Exons with extreme VST values (>0.89) between lake-river population pairs, where positive values represent higher copy-numbers in rivers and negative values represent higher copy-numbers in lakes. (PDF) [file pgen.1004830.s032.pdf]

Supplementary Table 10 - Exons with the most extreme Vst values (>0.89) between lake-river population pairs, where positive values represent higher copy-numbers in rivers and negative values represent higher copy-numbers in lakes.

| **Chromosome** | **Start** | **End** | **Gene ID** | **Gene Name** | **Orthology** | **G1** | **G2** | **No** | **Us** | **Ca** |
|---|---|---|---|---|---|---|---|---|---|---|
| groupI | 10754992 | 10755225 | ENSGACG00000010344 | CASR | Non-LSG LSD | -0.080 | 0.122 | -0.890 | 0.050 | 0.110 |
| groupI | 10755421 | 10755716 | ENSGACG00000010344 | CASR | Non-LSG LSD | -0.035 | 0.023 | -0.900 | 0.028 | 0.026 |
| groupI | 10755849 | 10755936 | ENSGACG00000010344 | CASR | Non-LSG LSD | -0.018 | 0.003 | -0.892 | 0.012 | 0.010 |
| groupI | 10758018 | 10758074 | ENSGACG00000010344 | CASR | Non-LSG LSD | -0.026 | 0.000 | -0.930 | -0.410 | 0.266 |
| groupI | 19769585 | 19769735 | ENSGACG00000013689 | PIPOX | Non-LSG Sing | -0.004 | 0.916 | 0.066 | -0.002 | 0.100 |
| groupI | 19772822 | 19772898 | ENSGACG00000013689 | PIPOX | Non-LSG Sing | 0.015 | 0.890 | 0.314 | 0.131 | 0.054 |
| groupI | 19779148 | 19779359 | ENSGACG00000013689 | PIPOX | Non-LSG Sing | -0.009 | 0.940 | 0.013 | 0.015 | 0.279 |
| groupII | 11734675 | 11734823 | ENSGACG00000015895 | SLC38A7 | Non-LSG Para | 0.950 | 0.455 | 0.005 | -0.012 | -0.173 |
| groupII | 11734944 | 11735144 | ENSGACG00000015895 | SLC38A7 | Non-LSG Para | 0.951 | 0.515 | -0.002 | 0.059 | -0.059 |
| groupII | 11735237 | 11735289 | ENSGACG00000015895 | SLC38A7 | Non-LSG Para | 0.933 | 0.513 | -0.007 | 0.084 | -0.019 |
| groupII | 11735781 | 11736286 | ENSGACG00000015895 | SLC38A7 | Non-LSG Para | 0.934 | 0.589 | 0.000 | 0.145 | 0.023 |
| groupII | 11736098 | 11736184 | ENSGACG00000015897 | CTSA | Non-LSG Para | 0.936 | 0.612 | -0.013 | 0.067 | -0.062 |
| groupII | 11737450 | 11738182 | ENSGACG00000015897 | CTSA | Non-LSG Para | 0.956 | 0.406 | 0.117 | 0.056 | -0.001 |
| groupII | 11738290 | 11738380 | ENSGACG00000015897 | CTSA | Non-LSG Para | 0.923 | 0.403 | -0.217 | 0.006 | -0.190 |
| groupII | 11738474 | 11738637 | ENSGACG00000015897 | CTSA | Non-LSG Para | 0.913 | 0.476 | 0.038 | 0.001 | -0.017 |
| groupII | 11738940 | 11739089 | ENSGACG00000015897 | CTSA | Non-LSG Para | 0.945 | 0.490 | 0.018 | 0.003 | -0.024 |
| groupII | 11739165 | 11739318 | ENSGACG00000015897 | CTSA | Non-LSG Para | 0.949 | 0.479 | -0.008 | 0.006 | -0.020 |
| groupII | 11739403 | 11739488 | ENSGACG00000015897 | CTSA | Non-LSG Para | 0.949 | 0.479 | -0.008 | 0.006 | -0.020 |
| groupII | 11739749 | 11739841 | ENSGACG00000015897 | CTSA | Non-LSG Para | 0.920 | 0.446 | -0.001 | -0.027 | -0.303 |
| groupII | 11740979 | 11741091 | ENSGACG00000015897 | CTSA | Non-LSG Para | 0.936 | 0.329 | -0.026 | 0.085 | -0.005 |
| groupII | 11741180 | 11741433 | ENSGACG00000015897 | CTSA | Non-LSG Para | 0.922 | 0.294 | -0.053 | 0.101 | -0.006 |
| groupII | 11741761 | 11741776 | ENSGACG00000015897 | CTSA | Non-LSG Para | 0.909 | 0.552 | 0.016 | -0.016 | 0.119 |
| groupII | 11741839 | 11741866 | ENSGACG00000015897 | CTSA | Non-LSG Para | 0.909 | 0.552 | 0.016 | -0.016 | 0.119 |
| groupIX | 17105470 | 17105646 | ENSGACG00000019395 | - | LSG singleton | 0.915 | 0.083 | 0.524 | -0.004 | -0.021 |
| groupIX | 17105737 | 17106058 | ENSGACG00000019395 | - | LSG singleton | 0.904 | 0.073 | 0.554 | 0.000 | 0.000 |
| groupVI | 10479091 | 10479406 | ENSGACG00000008985 | PIGR | Non-LSG Para | 0.446 | -0.047 | 0.016 | 0.207 | -0.931 |

| | | | | | | | | | | |
|---|---|---|---|---|---|---|---|---|---|---|
| groupVII | 439558 | 439731 | ENSGACG00000018648 | BTN1A1 | Non-LSG LSD | -0.332 | 0.073 | 0.897 | -0.009 | -0.185 |
| groupVII | 19324065 | 19324109 | ENSGACG00000020509 | C1QL | Non-LSG LSD | -0.016 | -0.260 | 0.002 | 0.100 | -0.903 |
| groupVII | 19327521 | 19327909 | ENSGACG00000020509 | C1QL | Non-LSG LSD | -0.271 | 0.008 | -0.047 | 0.085 | 0.910 |
| groupVII | 19779212 | 19779232 | ENSGACG00000020542 | OR1 | Non-LSG LSD | 0.163 | -0.019 | -0.135 | 0.048 | 0.895 |
| groupVII | 21451027 | 21451125 | ENSGACG00000020614 | SLC47A1 | Non-LSG LSD | -0.033 | -0.011 | -0.670 | -0.431 | -0.911 |
| groupVII | 21451211 | 21451309 | ENSGACG00000020614 | SLC47A1 | Non-LSG LSD | -0.033 | -0.011 | -0.670 | -0.431 | -0.911 |
| groupVII | 21456003 | 21456062 | ENSGACG00000020614 | SLC47A1 | Non-LSG LSD | 0.033 | 0.010 | -0.684 | -0.476 | -0.930 |
| groupVII | 21456126 | 21456144 | ENSGACG00000020614 | SLC47A1 | Non-LSG LSD | 0.033 | 0.010 | -0.684 | -0.476 | -0.930 |
| groupVIII | 5803761 | 5803867 | ENSGACG00000005883 | F13B | Non-LSG LSD | 0.014 | -0.243 | 0.044 | -0.968 | 0.492 |
| groupX | 6373705 | 6373853 | ENSGACG00000004084 | CNDP2 | Non-LSG Para | 0.257 | 0.894 | 0.056 | -0.055 | -0.001 |
| groupXIII | 3170008 | 3170069 | ENSGACG00000005319 | NELF | Non-LSG LSD | -0.861 | -0.119 | 0.000 | -0.035 | -0.911 |
| groupXIII | 3288790 | 3288874 | ENSGACG00000005345 | CD53 | Non-LSG Para | -0.925 | -0.265 | -0.101 | 0.046 | -0.311 |
| groupXIII | 18327848 | 18328184 | ENSGACG00000014249 | - | LSG LSD | -0.103 | -0.344 | -0.017 | -0.045 | -0.921 |
| groupXIII | 18573306 | 18573822 | ENSGACG00000014283 | NLRC3-like | Non-LSG LSD | -0.907 | 0.680 | 0.120 | -0.047 | 0.348 |
| groupXX | 8675984 | 8676444 | ENSGACG00000007491 | CMKLR1 | Non-LSG LSD | -0.890 | -0.534 | 0.238 | -0.224 | 0.045 |
| groupXX | 8676495 | 8677025 | ENSGACG00000007491 | CMKLR1 | Non-LSG LSD | -0.896 | -0.391 | 0.235 | -0.611 | 0.021 |
| groupXX | 13516594 | 13516696 | ENSGACG00000012348 | PYCARD1 | Non-LSG LSD | 0.131 | -0.196 | -0.134 | -0.023 | 0.940 |
| groupXX | 13527700 | 13528280 | ENSGACG00000012351 | PYCARD2 | Non-LSG LSD | -0.461 | 0.056 | -0.174 | -0.125 | 0.900 |
| groupXX | 13537537 | 13537586 | ENSGACG00000012355 | - | LSG singleton | 0.226 | 0.000 | -0.253 | -0.135 | -0.930 |
| groupXX | 13540167 | 13540477 | ENSGACG00000012354 | - | LSG singleton | 0.100 | 0.013 | -0.178 | -0.094 | -0.900 |
| groupXXI | 7957299 | 7957358 | ENSGACG00000003405 | NEOVTXA | Non-LSG LSD | -0.927 | -0.380 | 0.176 | 0.086 | 0.715 |
| groupXXI | 7957488 | 7957594 | ENSGACG00000003405 | NEOVTXA | Non-LSG LSD | -0.924 | -0.477 | 0.014 | 0.123 | 0.938 |
| groupXXI | 7994878 | 7996339 | ENSGACG00000003408 | SNTX | Non-LSG LSD | -0.954 | -0.114 | 0.028 | 0.003 | 0.724 |
| scaffold_320 | 2284 | 3683 | ENSGACG00000001341 | TRIM35 | Non-LSG LSD | -0.071 | -0.494 | -0.149 | 0.000 | -0.902 |
| scaffold_54 | 671409 | 671550 | ENSGACG00000002279 | - | LSG singleton | 0.916 | 0.093 | -0.011 | -0.015 | -0.372 |
