# Supplementary material for: Extensive Copy-Number Variation of Young Genes across Stickleback Populations
Source: PLoS Genet. 2014 Dec 4;10(12):e1004830. doi: 10.1371/journal.pgen.1004830 (PMC4256280; doi:10.1371/journal.pgen.1004830)
Supplement: Table S11 — CNVs and CNV genes separated into (a) deletion calls and duplication calls, (b) CNV gene deletions and CNV gene duplications. (PDF) [file pgen.1004830.s033.pdf]

Supplementary Table 11 - Position of all (a) CNVs and (b) CNV genes separated into deletion calls and duplication calls.

a)

CNV Deletions		
Chromosome	Start	End
groupI	44000	45500
groupI	52500	53500
groupI	84500	86500
groupI	286500	318000
groupI	324000	337000
groupI	371500	375000
groupI	561000	565000
groupI	581000	583000
groupI	657500	669000
groupI	711000	717000
groupI	815000	816500
groupI	915000	917000
groupI	965000	966500
groupI	985500	986500
groupI	1002500	1005000
groupI	1030500	1032000
groupI	1086000	1088500
groupI	1206000	1207000
groupI	1213500	1215500
groupI	1374000	1376000
groupI	1403500	1404500
groupI	1408000	1422000
groupI	1460000	1462500
groupI	1487500	1491000
groupI	1585000	1591500
groupI	1747000	1754000
groupI	1802000	1804000
groupI	1859500	1863000
groupI	2312000	2314500

CNV Duplications		
Chromosome	Start	End
groupI	324000	338000
groupI	457500	463000
groupI	852000	892000
groupI	2149500	2159500
groupI	2596000	2601500
groupI	2936000	2955500
groupI	2994500	3005500
groupI	3087000	3095000
groupI	3179500	3186500
groupI	4253500	4275000
groupI	5241500	5251500
groupI	5737000	5760500
groupI	6210000	6220500
groupI	7051500	7064500
groupI	7113000	7207500
groupI	10048500	10070000
groupI	10736500	10744500
groupI	11300000	11309500
groupI	11429000	11437500
groupI	12430000	12457500
groupI	13198500	13203000
groupI	14085000	14092000
groupI	14414500	14438000
groupI	14803500	14815000
groupI	16438500	16466500
groupI	16493500	16502000
groupI	16778000	16784500
groupI	16795500	16810000
groupI	17585500	17591500

groupI	2332000	2335500
groupI	2381500	2383500
groupI	2433500	2437000
groupI	2462500	2466000
groupI	2478500	2482500
groupI	2530000	2531000
groupI	2571500	2572500
groupI	2802500	2806500
groupI	2812000	2820500
groupI	2839000	2844000
groupI	2861500	2862500
groupI	2878000	2879500
groupI	2885000	2982000
groupI	2995500	3028500
groupI	3142500	3143500
groupI	3166500	3169000
groupI	3179500	3191500
groupI	3450000	3452000
groupI	3493000	3494500
groupI	3562500	3563500
groupI	3644500	3645500
groupI	3724000	3726000
groupI	3991500	3993500
groupI	4072000	4077000
groupI	4236500	4241500
groupI	4307000	4308000
groupI	4804000	4805500
groupI	4939500	4941000
groupI	5017500	5018500
groupI	5160000	5162000
groupI	5179500	5181500
groupI	5350500	5353000
groupI	5371000	5372000
groupI	5429000	5432000

groupI	17743500	17766500
groupI	17802000	17811500
groupI	18218000	18226000
groupI	18991000	19000500
groupI	19183500	19191000
groupI	19402500	19416500
groupI	19761000	19783500
groupI	19903500	19920000
groupI	20212500	20221000
groupI	21309000	21314500
groupI	22157500	22164000
groupI	22304500	22329500
groupI	22366000	22390500
groupI	23349000	23359500
groupI	23665500	23678500
groupI	24067000	24073500
groupI	25120000	25129500
groupI	26478500	26485000
groupI	27570500	27585000
groupI	27646500	27662500
groupII	260500	268000
groupII	2511000	2521000
groupII	2558000	2565000
groupII	2969500	3001500
groupII	3371500	3379500
groupII	3610000	3667500
groupII	3999000	4011000
groupII	5261000	5302500
groupII	6497500	6507500
groupII	7237500	7243500
groupII	10197500	10206000
groupII	10448500	10458000
groupII	11310000	11315500
groupII	11364000	11371500

groupI	5600500	5602500
groupI	5969500	5972500
groupI	6209000	6210000
groupI	6328500	6329500
groupI	6550000	6552000
groupI	6656500	6657500
groupI	6741500	6747000
groupI	7263000	7265000
groupI	7294500	7300000
groupI	7314000	7318500
groupI	7354000	7357000
groupI	7674000	7676000
groupI	7759500	7761000
groupI	7890500	7894000
groupI	8157000	8159000
groupI	8170000	8172000
groupI	8183000	8185500
groupI	8545500	8546500
groupI	8581000	8584000
groupI	8633500	8635000
groupI	8966000	8967500
groupI	9154000	9156000
groupI	9226000	9229000
groupI	9322000	9323000
groupI	9350500	9352500
groupI	9408500	9418500
groupI	9427000	9428500
groupI	9431500	9432500
groupI	9458000	9460500
groupI	9478000	9479000
groupI	9658500	9659500
groupI	9736000	9739500
groupI	10321500	10325500
groupI	10651000	10663500

groupII	11733500	11744000
groupII	13377500	13395500
groupII	13408000	13422000
groupII	13487500	13494000
groupII	14001500	14008000
groupII	14617000	14632500
groupII	16415500	16424000
groupII	17208000	17214000
groupII	18004000	18013000
groupII	18280500	18301000
groupII	18347000	18355000
groupII	18511500	18523500
groupII	18948500	18961500
groupII	19395500	19408000
groupII	19414000	19423500
groupII	20485000	20491000
groupII	21490000	21504000
groupII	21529000	21538500
groupII	21805500	21814500
groupII	22604000	22735000
groupII	22820500	22828000
groupIII	703500	710000
groupIII	1300500	1310000
groupIII	1797000	1803500
groupIII	2155500	2167000
groupIII	2668000	2676500
groupIII	2832500	2840000
groupIII	2915500	2924500
groupIII	2945000	2950500
groupIII	3213000	3220500
groupIII	3310500	3316000
groupIII	5086500	5101500
groupIII	5342000	5350000
groupIII	6656500	6709000

groupI	10667000	10669000
groupI	10687500	10737500
groupI	10744500	10745500
groupI	10753500	10759000
groupI	11309500	11311000
groupI	11341000	11343000
groupI	11422500	11427000
groupI	11689500	11692000
groupI	11729000	11730000
groupI	11996000	11997000
groupI	12027000	12028000
groupI	12065000	12069000
groupI	12070000	12074000
groupI	12217000	12218000
groupI	12431500	12436500
groupI	12459000	12463500
groupI	12777500	12778500
groupI	13179000	13185000
groupI	13196000	13197000
groupI	13197500	13198500
groupI	13302500	13305000
groupI	13643500	13647000
groupI	13821500	13822500
groupI	14198500	14200000
groupI	14879000	14881500
groupI	15006500	15007500
groupI	15091500	15096000
groupI	15304500	15306500
groupI	15522500	15524000
groupI	15579500	15582500
groupI	15588000	15591500
groupI	15606500	15610000
groupI	15625500	15626500
groupI	15635500	15637000

groupIII	7587000	7593500
groupIII	8817500	8836000
groupIII	9901500	9907500
groupIII	10130000	10140000
groupIII	10177500	10186000
groupIII	10700500	10705500
groupIII	12409000	12416000
groupIII	13789500	13805000
groupIII	15011000	15040000
groupIII	15041000	15056500
groupIII	15080000	15095000
groupIII	15307500	15324000
groupIII	15349000	15367500
groupIII	15434500	15463500
groupIII	15472000	15492500
groupIII	15527500	15538000
groupIII	15566000	15576000
groupIII	15679500	15691000
groupIII	15762000	15776000
groupIII	15783500	15792500
groupIII	15892500	15912500
groupIII	15948000	15958500
groupIII	16002500	16017000
groupIII	16075500	16080000
groupIII	16771000	16785500
groupIV	144500	161500
groupIV	1026500	1051500
groupIV	1061500	1068500
groupIV	1158000	1172000
groupIV	2037500	2048500
groupIV	2383000	2400000
groupIV	3558500	3566000
groupIV	5221500	5237500
groupIV	5864500	5876500

groupI	15737000	15739000
groupI	15747500	15749500
groupI	15976500	15980000
groupI	16145000	16147500
groupI	16212500	16215500
groupI	16346500	16347500
groupI	16410000	16412000
groupI	16464500	16465500
groupI	16506000	16508500
groupI	16919000	16920500
groupI	16926000	16940000
groupI	16945500	16946500
groupI	17204500	17210000
groupI	17321500	17326000
groupI	17379000	17420500
groupI	17481500	17482500
groupI	17500500	17512500
groupI	17567500	17568500
groupI	17595500	17598000
groupI	17628000	17629000
groupI	17630000	17633000
groupI	17719500	17721000
groupI	17967000	17970000
groupI	18329500	18332500
groupI	18357500	18360000
groupI	18497000	18502000
groupI	18671000	18674000
groupI	18984000	18985500
groupI	19013000	19014000
groupI	19023500	19027000
groupI	19110000	19113000
groupI	19136000	19137500
groupI	19171500	19175500
groupI	19183000	19191500

groupIV	8178000	8188000
groupIV	8353500	8358500
groupIV	8697500	8707000
groupIV	9438500	9461000
groupIV	9853000	9867000
groupIV	9994000	10007000
groupIV	10846500	10893000
groupIV	11473500	11477000
groupIV	12109000	12171000
groupIV	12173500	12184000
groupIV	12436500	12442500
groupIV	13212500	13221000
groupIV	14251500	14260500
groupIV	14281000	14289500
groupIV	15016000	15028000
groupIV	15729500	15742500
groupIV	16092000	16102000
groupIV	16955500	16964500
groupIV	17031000	17037500
groupIV	17184000	17188500
groupIV	17639500	17681500
groupIV	17692500	17717500
groupIV	17958000	17985000
groupIV	21066500	21106000
groupIV	21950500	21959000
groupIV	22045500	22053500
groupIV	22122500	22132000
groupIV	22853000	22870000
groupIV	23022000	23031500
groupIV	23073500	23095500
groupIV	23634000	23639000
groupIV	23816500	23833500
groupIV	24015500	24024000
groupIV	24539500	24547000

groupI	19275500	19298500
groupI	19312000	19313500
groupI	19315500	19317500
groupI	19331500	19333500
groupI	19383500	19384500
groupI	19434500	19438500
groupI	19645500	19647000
groupI	19765500	19794500
groupI	19803500	19805500
groupI	19855000	19867000
groupI	20002000	20005000
groupI	20009500	20021500
groupI	20091500	20093000
groupI	20110000	20112000
groupI	20128500	20130500
groupI	20148500	20151000
groupI	20211500	20212500
groupI	20346500	20372000
groupI	20402500	20405000
groupI	20424500	20426500
groupI	20503000	20504500
groupI	20581000	20582000
groupI	20677000	20678000
groupI	20696000	20716500
groupI	20737500	20739000
groupI	20783500	20784500
groupI	20797000	20798000
groupI	20969500	20971000
groupI	21134500	21137000
groupI	21201500	21203000
groupI	21231500	21232500
groupI	21266000	21267000
groupI	21343500	21348500
groupI	21477000	21479000

groupIV	24614000	24627000
groupIV	25442000	25449000
groupIV	25512000	25519000
groupIV	25535500	25657500
groupIV	25660000	25679000
groupIV	26367500	26377000
groupIV	26852500	26857000
groupIX	530000	540000
groupIX	938500	949500
groupIX	1068500	1089000
groupIX	2322500	2339500
groupIX	3620000	3623500
groupIX	3720500	3729000
groupIX	4853000	4869000
groupIX	5540500	5546500
groupIX	5912500	5918000
groupIX	7909500	7925000
groupIX	8832000	8846000
groupIX	9041000	9046500
groupIX	9429000	9434000
groupIX	9449500	9472500
groupIX	9548000	9556500
groupIX	9725500	9757000
groupIX	10194000	10202000
groupIX	10471500	10477500
groupIX	10674000	10680000
groupIX	10690000	10707000
groupIX	12143500	12160500
groupIX	12703500	12727000
groupIX	13025000	13033000
groupIX	13036000	13044500
groupIX	13077500	13295000
groupIX	13414500	13458000
groupIX	13631000	13637000

groupI	21480500	21490000
groupI	21693500	21698000
groupI	21714000	21718000
groupI	21731000	21733500
groupI	21739000	21741500
groupI	21844500	21847000
groupI	21936000	21949000
groupI	22017500	22032500
groupI	22210000	22216000
groupI	22295000	22353000
groupI	22366500	22397500
groupI	22449500	22454500
groupI	22509000	22512000
groupI	22560000	22562500
groupI	22574000	22593500
groupI	22610000	22611500
groupI	22734500	22736000
groupI	22992500	22993500
groupI	23091000	23093500
groupI	23489000	23491000
groupI	23536000	23541000
groupI	23547000	23549500
groupI	23616500	23617500
groupI	23790000	23791500
groupI	24155500	24157000
groupI	24160500	24162500
groupI	24176500	24178000
groupI	24741500	24750000
groupI	24832000	24838000
groupI	24885000	24886500
groupI	24947500	24948500
groupI	24991500	24993500
groupI	25048500	25050000
groupI	25097500	25098500

groupIX	13750500	13763000
groupIX	14187500	14286000
groupIX	14316500	14341000
groupIX	14763000	14767500
groupIX	14779500	14801000
groupIX	14833500	14875000
groupIX	14879500	14887500
groupIX	14945500	14952500
groupIX	15313000	15328000
groupIX	16423500	16442500
groupIX	16452000	16466000
groupIX	16734000	16792000
groupIX	17143000	17147500
groupIX	17624500	17630500
groupIX	18001000	18017000
groupIX	18611500	18633000
groupIX	19219000	19230000
groupIX	19355000	19371500
groupIX	19400500	19415000
groupIX	19436000	19448500
groupV	1396500	1405000
groupV	1524000	1531500
groupV	2342000	2365500
groupV	2403000	2461500
groupV	2799500	2807500
groupV	3322500	3331000
groupV	4633000	4638000
groupV	4816000	4822000
groupV	5410000	5428500
groupV	5913000	5925000
groupV	6386500	6407500
groupV	6430000	6441000
groupV	6629500	6647000
groupV	7407000	7413500

groupI	25207500	25209500
groupI	25233000	25236000
groupI	25272500	25273500
groupI	25291500	25292500
groupI	25492500	25494500
groupI	25598500	25603000
groupI	25610500	25622500
groupI	25633500	25634500
groupI	25911500	25927000
groupI	25951000	25953500
groupI	25964000	25968000
groupI	25979000	25983500
groupI	26007000	26010000
groupI	26039000	26042500
groupI	26257500	26258500
groupI	26346000	26347000
groupI	26536000	26553500
groupI	26582000	26584000
groupI	26593500	26605000
groupI	26661500	26665000
groupI	26700500	26701500
groupI	26734500	26741500
groupI	26785000	26787500
groupI	26817000	26818500
groupI	26822000	26823500
groupI	26873500	26874500
groupI	27201500	27204500
groupI	27418000	27419000
groupI	27468500	27471000
groupI	27496500	27499500
groupI	27599000	27600000
groupI	27717000	27718000
groupI	27730000	27732000
groupI	27744000	27745000

groupV	8119500	8126000
groupV	8335000	8344500
groupV	9033000	9039500
groupV	9156500	9170500
groupV	9335500	9359000
groupV	9387000	9395500
groupV	9709000	9717500
groupV	9739000	9757000
groupV	9895500	9905000
groupV	10722500	10735000
groupV	11878500	11885500
groupVI	214000	224500
groupVI	347500	408000
groupVI	706000	715500
groupVI	1986500	1993000
groupVI	2291500	2304500
groupVI	2584500	2599500
groupVI	3602000	3645000
groupVI	5065000	5083000
groupVI	5158000	5164500
groupVI	5586500	5593500
groupVI	6419000	6425500
groupVI	7594500	7601000
groupVI	8270500	8293500
groupVI	8415000	8430000
groupVI	8449000	8453000
groupVI	8790000	8802000
groupVI	9253000	9260500
groupVI	9305000	9312500
groupVI	10458500	10474500
groupVI	10480000	10496000
groupVI	13256500	13263000
groupVI	13970000	13982500
groupVI	14293500	14304500

groupI	27753500	27784000
groupI	27814500	27824500
groupI	27861000	27862000
groupI	27863500	27866000
groupI	27896000	27898500
groupI	28020500	28026000
groupI	28058000	28060000
groupI	28103500	28120500
groupII	45000	46000
groupII	64000	65500
groupII	97500	98500
groupII	109000	110500
groupII	117500	120000
groupII	229000	234000
groupII	242000	243000
groupII	260500	269500
groupII	384500	386000
groupII	413000	416000
groupII	431000	432500
groupII	437000	440000
groupII	534500	537000
groupII	541000	544000
groupII	558500	587000
groupII	597000	608500
groupII	613500	626500
groupII	629000	638000
groupII	663500	669000
groupII	693000	694500
groupII	924000	937000
groupII	947500	950000
groupII	978500	982000
groupII	1011000	1013000
groupII	1055000	1057000
groupII	1104500	1107500

groupVI	14575000	14580500
groupVI	15428000	15436000
groupVII	110000	114500
groupVII	135000	147000
groupVII	191500	195500
groupVII	1530000	1541000
groupVII	1860000	1874000
groupVII	1943000	1953500
groupVII	2012500	2028000
groupVII	2030500	2047000
groupVII	2088500	2103000
groupVII	2139500	2238500
groupVII	2791500	2803000
groupVII	2803500	2811000
groupVII	2970000	2982500
groupVII	3089500	3096000
groupVII	4063000	4083000
groupVII	4288500	4295500
groupVII	4623000	4626500
groupVII	4632500	4673500
groupVII	5032500	5041000
groupVII	6920500	6945500
groupVII	7735000	7745000
groupVII	7833000	7844500
groupVII	8159500	8168500
groupVII	8625000	8633500
groupVII	9013500	9020500
groupVII	9210500	9219000
groupVII	9616000	9623000
groupVII	10200500	10210500
groupVII	10597000	10605500
groupVII	11394000	11401000
groupVII	11428500	11436500
groupVII	13534500	13547500

groupII	1127000	1128500
groupII	1506000	1508000
groupII	1781500	1784000
groupII	1854500	1855500
groupII	1876000	1877000
groupII	1947000	1951500
groupII	2082000	2084500
groupII	2117500	2119000
groupII	2371500	2373000
groupII	2421000	2423000
groupII	2440000	2443000
groupII	2446000	2452500
groupII	2789500	2790500
groupII	2865500	2867500
groupII	2985000	2989500
groupII	3158500	3159500
groupII	3303000	3304500
groupII	3305500	3308000
groupII	3376000	3377000
groupII	3485000	3500000
groupII	3590000	3601500
groupII	3604500	3666500
groupII	3746000	3747500
groupII	4110500	4112500
groupII	4127500	4132000
groupII	4415500	4430000
groupII	5307500	5311500
groupII	5515500	5516500
groupII	5678000	5679000
groupII	5791500	5794000
groupII	5906500	5908000
groupII	5989000	5993500
groupII	6072500	6081500
groupII	6101000	6113500

groupVII	16664000	16670500
groupVII	16814000	16823500
groupVII	17375000	17381000
groupVII	18546500	18554000
groupVII	19672500	19726500
groupVII	19781000	19787000
groupVII	20737000	20757000
groupVII	21373500	21378000
groupVII	21563500	21569000
groupVII	21702000	21715500
groupVII	22629000	22638500
groupVII	24727000	24737500
groupVII	25182500	25193000
groupVII	26097000	26105000
groupVII	26975500	26988500
groupVIII	200500	213500
groupVIII	577500	584000
groupVIII	1266500	1283500
groupVIII	1355000	1364000
groupVIII	1463500	1496500
groupVIII	1500000	1547000
groupVIII	2394500	2402000
groupVIII	3248500	3259500
groupVIII	3451000	3499500
groupVIII	3976000	3985000
groupVIII	4405500	4424000
groupVIII	4796500	4815000
groupVIII	4873500	4885500
groupVIII	5819000	5844000
groupVIII	6010500	6025000
groupVIII	7380500	7388000
groupVIII	8227500	8243000
groupVIII	9798500	9805500
groupVIII	10186000	10201000

groupII	6198500	6204000
groupII	6856000	6857000
groupII	6861000	6872000
groupII	6909000	6911000
groupII	6953000	6963500
groupII	6970500	6971500
groupII	7028500	7029500
groupII	7121000	7122000
groupII	7200500	7205500
groupII	7313000	7314500
groupII	7447000	7448000
groupII	7457000	7460000
groupII	7581000	7582500
groupII	7587000	7590000
groupII	7593500	7596000
groupII	8702000	8703000
groupII	8922500	8923500
groupII	9358500	9362500
groupII	9465000	9468000
groupII	10565000	10567000
groupII	10980500	10982500
groupII	10997500	11000000
groupII	11279500	11280500
groupII	11998500	11999500
groupII	12025000	12026000
groupII	12134500	12136500
groupII	12144500	12145500
groupII	12294000	12295500
groupII	12482500	12483500
groupII	12941500	12944500
groupII	13176000	13178000
groupII	13213000	13215000
groupII	13311500	13312500
groupII	13370500	13376000

groupVIII	12326000	12330500
groupVIII	12490000	12499500
groupVIII	12501500	12515000
groupVIII	12752000	12763500
groupVIII	12923500	12931000
groupVIII	13014500	13033000
groupVIII	13874000	13883500
groupVIII	17182000	17191000
groupVIII	17426500	17434000
groupVIII	17707500	17716000
groupVIII	18843500	18861000
groupVIII	18885000	18911000
groupVIII	18976000	18996500
groupX	335000	343000
groupX	474500	527000
groupX	585000	596500
groupX	672500	688500
groupX	706500	718500
groupX	799500	884500
groupX	901500	918000
groupX	1070000	1090500
groupX	1251000	1258000
groupX	1615000	1623000
groupX	2022000	2030000
groupX	2315000	2320500
groupX	2357500	2366500
groupX	7009000	7014000
groupX	7199500	7213500
groupX	7273000	7294000
groupX	7850000	7858000
groupX	9146500	9163500
groupX	9592500	9600500
groupX	10905500	10911500
groupX	11202500	11209500

groupII	13378000	13382500
groupII	13478500	13480000
groupII	13532500	13535500
groupII	13681500	13683000
groupII	13954500	13956000
groupII	14028000	14029000
groupII	14526500	14528500
groupII	14544000	14575000
groupII	14622000	14635500
groupII	14727000	14728000
groupII	14828500	14832500
groupII	14840500	14843000
groupII	14948000	14949000
groupII	15047500	15048500
groupII	15605000	15606500
groupII	15664000	15678500
groupII	15776000	15792000
groupII	16361000	16424500
groupII	16955500	16958000
groupII	16985000	16992500
groupII	17058500	17060500
groupII	17207000	17210000
groupII	17390500	17391500
groupII	17407500	17409500
groupII	17568500	17570000
groupII	17879500	17880500
groupII	18287500	18291500
groupII	18297500	18301500
groupII	18912000	18913500
groupII	19157000	19172500
groupII	19354000	19356000
groupII	19767500	19769000
groupII	19843000	19848000
groupII	20050500	20051500

groupX	11394000	11419500
groupX	12659500	12679500
groupX	12686500	12732000
groupX	13189500	13228000
groupX	13234500	13264500
groupX	13759500	13766000
groupX	14431000	14444000
groupX	14670000	14679000
groupX	14747000	14784000
groupX	14977500	14989500
groupXI	64000	82000
groupXI	797000	811500
groupXI	1106000	1126000
groupXI	1144500	1176000
groupXI	1906500	1916000
groupXI	2407000	2449000
groupXI	2517000	2530500
groupXI	2847000	2861500
groupXI	3247000	3254000
groupXI	4070000	4081500
groupXI	4309000	4356500
groupXI	4363000	4432000
groupXI	4532500	4540500
groupXI	5216000	5243500
groupXI	6451500	6474500
groupXI	6487000	6500000
groupXI	6681500	6689000
groupXI	7767500	7784000
groupXI	8753500	8768500
groupXI	9200000	9207000
groupXI	9648500	9687500
groupXI	9779500	9790500
groupXI	10204500	10219500
groupXI	10225500	10243000

groupII	20111500	20117000
groupII	20130500	20135500
groupII	20241000	20242000
groupII	20244000	20245500
groupII	20262500	20269000
groupII	20321000	20333500
groupII	20342500	20346500
groupII	20348000	20350000
groupII	20405500	20414500
groupII	20431000	20432500
groupII	20519000	20521500
groupII	20545500	20548000
groupII	20672500	20676500
groupII	20771500	20773500
groupII	20780500	20782000
groupII	20789500	20794000
groupII	20914000	20915000
groupII	20921500	20927500
groupII	21118000	21134000
groupII	21263500	21264500
groupII	21286000	21289500
groupII	21296000	21301000
groupII	21330500	21334500
groupII	21348500	21351500
groupII	21360000	21361000
groupII	21415500	21418500
groupII	21478500	21480000
groupII	21487000	21503500
groupII	21504000	21529000
groupII	21610000	21620500
groupII	21713000	21716500
groupII	21722000	21736000
groupII	21737000	21741000
groupII	21767500	21768500

groupXI	11027500	11050500
groupXI	11288000	11295500
groupXI	11534000	11571500
groupXI	11764500	11842000
groupXI	12066500	12083000
groupXI	12318500	12461000
groupXI	12681000	12700000
groupXI	12716500	12721500
groupXI	13011000	13018500
groupXI	14967000	14981000
groupXI	15605500	15633500
groupXI	15996000	16012500
groupXII	906000	926500
groupXII	949000	991000
groupXII	993500	1025500
groupXII	1027500	1044000
groupXII	2371000	2385000
groupXII	2495000	2504000
groupXII	2839500	2847500
groupXII	5085000	5092000
groupXII	5427500	5434000
groupXII	7848000	7860000
groupXII	7865000	7878500
groupXII	8048500	8100500
groupXII	8104000	8110000
groupXII	8588500	8598500
groupXII	10097000	10105000
groupXII	10286000	10327000
groupXII	10786500	10793000
groupXII	12998500	13015500
groupXII	13225500	13229500
groupXII	13373000	13382000
groupXII	13477500	13487000
groupXII	14226000	14233000

groupII	21776500	21790500
groupII	21795000	21796000
groupII	21802000	21805000
groupII	21809000	21810000
groupII	21814500	21831500
groupII	21832000	21833500
groupII	21909500	21932000
groupII	22016500	22020500
groupII	22065500	22068000
groupII	22103500	22116000
groupII	22220500	22225000
groupII	22244500	22249000
groupII	22320000	22322500
groupII	22585500	22586500
groupII	22617500	22618500
groupII	22731000	22732000
groupII	22867000	22868500
groupII	22914500	22921500
groupII	23027500	23030000
groupII	23063000	23065500
groupII	23082000	23086000
groupII	23091000	23093500
groupII	23163500	23176500
groupII	23181500	23189500
groupIII	220500	222000
groupIII	406500	414500
groupIII	427500	432000
groupIII	509000	516000
groupIII	713000	736000
groupIII	883500	887000
groupIII	919500	920500
groupIII	976500	978000
groupIII	1177000	1179500
groupIII	1441500	1443000

groupXII	17196500	17201000
groupXII	17247500	17263000
groupXII	17741000	17757000
groupXII	18062000	18068500
groupXIII	1196000	1203500
groupXIII	1728000	1737500
groupXIII	2228500	2234000
groupXIII	2403000	2464500
groupXIII	3161000	3178000
groupXIII	3219000	3262000
groupXIII	3267500	3273500
groupXIII	3371000	3378000
groupXIII	4301000	4356000
groupXIII	5021000	5033000
groupXIII	5086000	5093500
groupXIII	8822000	8844500
groupXIII	9481000	9490500
groupXIII	11528500	11540000
groupXIII	12127500	12136000
groupXIII	12334000	12345000
groupXIII	12438500	12446000
groupXIII	13130500	13140000
groupXIII	14421000	14428000
groupXIII	14461000	14467500
groupXIII	14718500	14728000
groupXIII	16087500	16102000
groupXIII	16767000	16798500
groupXIII	16869000	16876500
groupXIII	17329500	17341500
groupXIII	17347000	17355500
groupXIII	17433500	17442500
groupXIII	18494500	18503500
groupXIII	18569000	18589000
groupXIII	19566000	19582500

groupIII	1530000	1532000
groupIII	1753500	1756500
groupIII	1761500	1767000
groupIII	1889000	1891000
groupIII	2040000	2041500
groupIII	2105500	2110000
groupIII	2263000	2268000
groupIII	2378500	2381500
groupIII	2508000	2509500
groupIII	2564500	2566500
groupIII	2601000	2605000
groupIII	2779500	2782500
groupIII	2946000	2951000
groupIII	3314500	3319000
groupIII	3338000	3339000
groupIII	3531500	3534500
groupIII	3766000	3769000
groupIII	4069000	4070000
groupIII	4157000	4158500
groupIII	4177500	4180000
groupIII	4213000	4214000
groupIII	4317000	4318000
groupIII	4325500	4332500
groupIII	4349000	4361000
groupIII	4368500	4373500
groupIII	4492500	4513000
groupIII	4520000	4521000
groupIII	4537000	4538000
groupIII	4615500	4617500
groupIII	4660000	4665500
groupIII	4706000	4718500
groupIII	4754000	4756500
groupIII	4800000	4801500
groupIII	4992500	4999000

groupXIV	72000	80000
groupXIV	388500	397000
groupXIV	3533500	3539500
groupXIV	3554500	3564000
groupXIV	6019000	6024500
groupXIV	6573500	6583500
groupXIV	6953000	6962500
groupXIV	7332500	7339500
groupXIV	7945500	7951500
groupXIV	7976500	7981000
groupXIV	8821500	8825500
groupXIV	11224000	11231000
groupXIV	11265000	11274000
groupXIV	11650500	11664000
groupXIV	12369000	12384000
groupXIV	14446000	14456500
groupXIV	14506000	14512500
groupXIV	14675000	14684500
groupXIV	14953500	14986500
groupXV	141000	151500
groupXV	272500	281500
groupXV	702500	712000
groupXV	1523000	1541500
groupXV	2253000	2260500
groupXV	2590500	2621500
groupXV	2978000	3037000
groupXV	5494000	5501000
groupXV	5579500	5594000
groupXV	6037500	6044500
groupXV	6049500	6059500
groupXV	7768000	7777500
groupXV	7889000	7899000
groupXV	8096500	8105000
groupXV	8483500	8494000

groupIII	5035500	5037000
groupIII	5071000	5076000
groupIII	5097500	5099000
groupIII	5114000	5116000
groupIII	5549500	5553500
groupIII	5555500	5558500
groupIII	5606000	5607000
groupIII	5693000	5694500
groupIII	5707000	5717500
groupIII	5796000	5799500
groupIII	5868500	5880000
groupIII	5904000	5905000
groupIII	5907000	5910500
groupIII	5957000	5961500
groupIII	5995000	5996500
groupIII	6040500	6042000
groupIII	6179000	6180500
groupIII	6380000	6381000
groupIII	6406000	6407500
groupIII	6469500	6476500
groupIII	6603000	6604500
groupIII	6703500	6723000
groupIII	6726000	6728000
groupIII	6901000	6904000
groupIII	6977000	6978000
groupIII	7093500	7096000
groupIII	7172500	7175500
groupIII	7576500	7587500
groupIII	7604500	7605500
groupIII	8511500	8515000
groupIII	8607000	8611500
groupIII	9229000	9230000
groupIII	9354500	9361000
groupIII	9527500	9531000

groupXV	9811500	9820500
groupXV	11855000	11882500
groupXV	11891000	11901000
groupXV	12852500	12857500
groupXV	13200000	13208000
groupXV	13211500	13222000
groupXV	13614500	13619500
groupXVI	39500	109500
groupXVI	219500	227000
groupXVI	243500	252500
groupXVI	474500	486500
groupXVI	487500	498500
groupXVI	588000	596000
groupXVI	807500	815000
groupXVI	950000	961000
groupXVI	1250000	1256500
groupXVI	1971500	2015500
groupXVI	2080000	2085500
groupXVI	2254000	2260500
groupXVI	3197500	3206000
groupXVI	3367500	3372500
groupXVI	3857000	3866000
groupXVI	4213000	4219500
groupXVI	4305500	4328000
groupXVI	4666500	4796500
groupXVI	4801500	4811500
groupXVI	5801000	5808500
groupXVI	7684500	7690000
groupXVI	8169500	8178500
groupXVI	8269000	8303500
groupXVI	8306500	8324000
groupXVI	8396000	8400500
groupXVI	11787500	11822000
groupXVI	11849000	11864000

groupIII	10140000	10154500
groupIII	10156500	10159500
groupIII	10194000	10196000
groupIII	10253500	10255500
groupIII	10259500	10261000
groupIII	10594000	10595500
groupIII	10956000	10957000
groupIII	11081000	11083000
groupIII	11645000	11669000
groupIII	12023000	12025500
groupIII	12632500	12642500
groupIII	12644000	12647500
groupIII	12807000	12809000
groupIII	13447500	13451000
groupIII	13465000	13478000
groupIII	13765500	13767000
groupIII	14084000	14086500
groupIII	14107500	14108500
groupIII	14265500	14271000
groupIII	14352000	14354500
groupIII	14559500	14562500
groupIII	14630000	14632500
groupIII	14758000	14760000
groupIII	14780500	14781500
groupIII	14785000	14787000
groupIII	14802500	14809000
groupIII	14837500	14839500
groupIII	14842500	14843500
groupIII	14961000	14962500
groupIII	15012500	15101500
groupIII	15182000	15184000
groupIII	15274500	15486000
groupIII	15489500	15491000
groupIII	15492500	15497000

groupXVI	13798000	13810500
groupXVI	13855500	13870000
groupXVI	13899500	13905500
groupXVI	15493000	15520000
groupXVI	15945000	15952000
groupXVI	16333500	16437500
groupXVI	16456500	16485500
groupXVI	16907500	16971500
groupXVI	16992000	17041000
groupXVI	17065000	17076000
groupXVI	17120000	17134500
groupXVI	17146000	17178500
groupXVII	2500	7000
groupXVII	270500	276500
groupXVII	418000	428500
groupXVII	1309000	1324000
groupXVII	1834500	1867000
groupXVII	2392000	2403000
groupXVII	3473500	3483500
groupXVII	3730000	3735000
groupXVII	7729000	7735000
groupXVII	8435000	8442500
groupXVII	8582500	8617000
groupXVII	8739500	8745000
groupXVII	8808000	8818000
groupXVII	9225500	9229500
groupXVII	9633000	9648000
groupXVII	9667500	9679500
groupXVII	11175500	11186500
groupXVII	11731000	11739500
groupXVII	12163000	12173000
groupXVIII	761000	819000
groupXVIII	1381500	1390000
groupXVIII	1716500	1722000

groupIII	15504500	15528500
groupIII	15537500	15772500
groupIII	15775000	15779500
groupIII	15896000	15897000
groupIII	15946500	15948500
groupIII	15969500	15971500
groupIII	16001500	16017000
groupIII	16132500	16134000
groupIII	16580500	16585000
groupIII	16771000	16786000
groupIV	467000	471000
groupIV	528000	533000
groupIV	708500	710500
groupIV	839500	841500
groupIV	862500	865000
groupIV	895500	898500
groupIV	1035500	1039000
groupIV	1084000	1103000
groupIV	1130000	1164500
groupIV	1172000	1175000
groupIV	1231500	1237500
groupIV	1277500	1293000
groupIV	1354000	1355000
groupIV	1419000	1420000
groupIV	1536500	1537500
groupIV	1559000	1562500
groupIV	1577500	1580500
groupIV	1618500	1620000
groupIV	1635000	1636000
groupIV	1671000	1672000
groupIV	1730500	1732000
groupIV	1770500	1780000
groupIV	1833000	1834000
groupIV	1952500	1954000

groupXVIII	2448000	2454000
groupXVIII	2967000	2985500
groupXVIII	3092500	3101500
groupXVIII	3117000	3128500
groupXVIII	4128500	4136000
groupXVIII	4788000	4795000
groupXVIII	4823500	4844500
groupXVIII	5328500	5335500
groupXVIII	5755000	5766000
groupXVIII	6216000	6254500
groupXVIII	6578500	6627500
groupXVIII	10936500	10961000
groupXVIII	11175500	11189500
groupXVIII	11441500	11451500
groupXVIII	13121500	13137000
groupXVIII	13450000	13468000
groupXVIII	13562500	13571500
groupXVIII	14764000	14775000
groupXVIII	15417000	15426000
groupXVIII	15515000	15520000
groupXVIII	16012000	16029000
groupXX	709000	740500
groupXX	929500	933500
groupXX	1924500	1950500
groupXX	2077500	2109000
groupXX	2144500	2163500
groupXX	2348000	2361000
groupXX	2363500	2374500
groupXX	2456000	2528500
groupXX	3934000	3942000
groupXX	4571000	4577000
groupXX	6289000	6313000
groupXX	6318000	6328500
groupXX	7191000	7200000

groupIV	1966000	1969000
groupIV	2018500	2023000
groupIV	2035000	2040000
groupIV	2071500	2073000
groupIV	2091000	2093000
groupIV	2210000	2212500
groupIV	2220000	2225500
groupIV	2266000	2267000
groupIV	2475000	2476500
groupIV	2508000	2516500
groupIV	2569500	2571000
groupIV	2693500	2700000
groupIV	2701000	2702000
groupIV	2705500	2709500
groupIV	2714000	2716000
groupIV	2755000	2758000
groupIV	2780500	2784000
groupIV	2793500	2803500
groupIV	2837000	2840000
groupIV	2959000	2963000
groupIV	2996000	2999500
groupIV	3048500	3050500
groupIV	3084000	3085000
groupIV	3150000	3151000
groupIV	3207000	3208000
groupIV	3236000	3247500
groupIV	3254000	3255000
groupIV	3282000	3284000
groupIV	3345500	3349500
groupIV	3353000	3419000
groupIV	3420500	3422500
groupIV	3519500	3532500
groupIV	3565500	3570000
groupIV	3682000	3689000

groupXX	8306500	8311000
groupXX	8593000	8598000
groupXX	8810500	8827000
groupXX	8865500	8873500
groupXX	8893500	8897500
groupXX	9148500	9152000
groupXX	10332000	10336000
groupXX	13006000	13022500
groupXX	13539000	13550000
groupXX	13896000	13903000
groupXX	13950000	13960000
groupXX	14054500	14085000
groupXX	14149000	14170500
groupXX	14172500	14186000
groupXX	14197000	14211500
groupXX	14212000	14232000
groupXX	14257000	14264000
groupXX	14322000	14338000
groupXX	14353500	14373000
groupXX	15681500	15687000
groupXX	15877000	15884500
groupXX	15887500	15895500
groupXX	15972000	15983000
groupXX	16473000	16480000
groupXX	18219500	18235500
groupXX	18795000	18806500
groupXX	18814500	18834500
groupXX	19702500	19722000
groupXXI	1555500	1571500
groupXXI	1971000	1982000
groupXXI	3178000	3199500
groupXXI	3267500	3280500
groupXXI	3287000	3312000
groupXXI	3381000	3399000

groupIV	3697000	3698500
groupIV	4004000	4015500
groupIV	4021500	4028000
groupIV	4170000	4171500
groupIV	4209500	4235000
groupIV	4381000	4383000
groupIV	4481500	4483000
groupIV	4665500	4667000
groupIV	4702000	4703000
groupIV	4857500	4859500
groupIV	5174500	5207500
groupIV	6122000	6127500
groupIV	6337500	6338500
groupIV	6410000	6411500
groupIV	6485000	6486500
groupIV	6553500	6556000
groupIV	6795500	6796500
groupIV	6827000	6829000
groupIV	7142500	7144500
groupIV	7539500	7549000
groupIV	7590000	7593000
groupIV	7668000	7677500
groupIV	7845500	7848500
groupIV	7855500	7872000
groupIV	7888000	7893000
groupIV	8114500	8117500
groupIV	8183500	8186000
groupIV	8310000	8311500
groupIV	8328000	8329500
groupIV	8342000	8343000
groupIV	8430500	8431500
groupIV	8598000	8603000
groupIV	9096500	9098000
groupIV	9340000	9344500

groupXXI	3404000	3479500
groupXXI	3506500	3527500
groupXXI	3551500	3568000
groupXXI	3678500	3686000
groupXXI	3707500	3718500
groupXXI	3774000	3797000
groupXXI	4372500	4381000
groupXXI	4602000	4669000
groupXXI	5863500	5870500
groupXXI	7936000	7978000
groupXXI	7981500	7999500
groupXXI	8127500	8136500
groupXXI	9267000	9274000
groupXXI	9313500	9320000
groupXXI	10137500	10146500
groupXXI	10351500	10364500
scaffold_111	310500	317500
scaffold_1168	0	6500
scaffold_119	44500	57500
scaffold_119	131000	137500
scaffold_119	230000	240000
scaffold_1255	500	4500
scaffold_126	105500	116500
scaffold_129	129500	147500
scaffold_130	34000	41000
scaffold_131	0	124000
scaffold_132	166500	175000
scaffold_135	65000	121500
scaffold_137	36000	54000
scaffold_137	78500	104500
scaffold_147	0	216000
scaffold_160	48500	158500
scaffold_171	0	186500
scaffold_172	0	175000

groupIV	9455000	9456000
groupIV	9617000	9619500
groupIV	9731000	9734500
groupIV	9840500	9843500
groupIV	9859500	9862000
groupIV	9863000	9865500
groupIV	9879000	9882500
groupIV	9892500	9899500
groupIV	9912500	9914000
groupIV	9917500	9931500
groupIV	9937500	9953500
groupIV	9954500	9957000
groupIV	9957500	9961500
groupIV	9975500	9980000
groupIV	9981500	10019500
groupIV	10020000	10030000
groupIV	10034000	10037500
groupIV	10088000	10092500
groupIV	10093000	10095500
groupIV	10096000	10099000
groupIV	10099500	10110000
groupIV	10110500	10138500
groupIV	10159000	10162000
groupIV	10167500	10172500
groupIV	10176500	10179000
groupIV	10338000	10345500
groupIV	10363500	10364500
groupIV	10392000	10393500
groupIV	10419000	10420000
groupIV	10428500	10431000
groupIV	10445000	10446500
groupIV	10467000	10469000
groupIV	10475500	10477000
groupIV	10711000	10726500

scaffold_173	0	210000
scaffold_180	0	138000
scaffold_185	0	151500
scaffold_193	0	101500
scaffold_197	0	34000
scaffold_201	89500	105000
scaffold_216	2000	7000
scaffold_224	0	49500
scaffold_229	0	65500
scaffold_232	0	172500
scaffold_252	0	51000
scaffold_27	2624000	2640500
scaffold_27	2918000	2931500
scaffold_27	3347500	3384000
scaffold_27	3443000	3449000
scaffold_27	4426500	4433000
scaffold_27	4608500	4642000
scaffold_27	4801500	4808000
scaffold_290	0	63500
scaffold_299	0	36000
scaffold_316	0	32500
scaffold_320	0	32000
scaffold_321	36000	50000
scaffold_327	0	30500
scaffold_330	0	30000
scaffold_332	0	31500
scaffold_337	0	29500
scaffold_346	0	27000
scaffold_358	0	25500
scaffold_360	0	49000
scaffold_364	0	24500
scaffold_37	359000	370000
scaffold_37	371000	431000
scaffold_37	499500	535000

groupIV	10858000	10869500
groupIV	10883000	10884500
groupIV	10887000	10893000
groupIV	10895000	10896500
groupIV	10956000	10957500
groupIV	11147500	11148500
groupIV	11259000	11269500
groupIV	11671500	11672500
groupIV	11875000	11879000
groupIV	11973500	11974500
groupIV	12138500	12141000
groupIV	12147000	12150500
groupIV	12154000	12157000
groupIV	12317500	12319500
groupIV	12638000	12643000
groupIV	12690500	12695500
groupIV	12743500	12746500
groupIV	12800500	12801500
groupIV	12992500	12996500
groupIV	13073000	13074000
groupIV	13086000	13087000
groupIV	13621500	13629500
groupIV	13686500	13687500
groupIV	13692500	13695500
groupIV	13707000	13709000
groupIV	13712500	13714000
groupIV	13736500	13738000
groupIV	13781500	13783000
groupIV	13785000	13800000
groupIV	13957000	13959500
groupIV	14171500	14173500
groupIV	14179500	14180500
groupIV	14260500	14269500
groupIV	14327500	14331500

scaffold_37	578500	587000
scaffold_37	624000	664500
scaffold_37	780500	789500
scaffold_37	815500	827500
scaffold_37	1626500	1636500
scaffold_37	2228000	2260000
scaffold_374	0	23500
scaffold_416	0	20500
scaffold_434	0	19500
scaffold_454	0	17500
scaffold_470	0	17500
scaffold_48	47000	55000
scaffold_508	0	16000
scaffold_516	0	15500
scaffold_519	0	16000
scaffold_521	0	15500
scaffold_528	0	15500
scaffold_533	0	15000
scaffold_536	0	15500
scaffold_54	0	21500
scaffold_54	255000	263000
scaffold_54	658000	694500
scaffold_54	697500	716500
scaffold_56	259000	266000
scaffold_56	973500	1007500
scaffold_56	1066500	1093000
scaffold_56	1111000	1140500
scaffold_571	0	14000
scaffold_58	0	725000
scaffold_590	0	13500
scaffold_599	0	13000
scaffold_615	0	13000
scaffold_68	0	34000
scaffold_68	54500	66500

groupIV	14365500	14366500
groupIV	14497000	14499000
groupIV	14545000	14559500
groupIV	14596000	14599500
groupIV	14753000	14755000
groupIV	14760500	14762500
groupIV	14814500	14815500
groupIV	14850000	14851500
groupIV	14866000	14869000
groupIV	14987500	15010000
groupIV	15031000	15032500
groupIV	15193000	15194000
groupIV	15209000	15210500
groupIV	15273000	15280000
groupIV	15346500	15349500
groupIV	15359500	15362000
groupIV	15436500	15437500
groupIV	15554500	15555500
groupIV	15674500	15675500
groupIV	15742500	15763500
groupIV	15776000	15778500
groupIV	15926000	15927500
groupIV	15959000	15961500
groupIV	16011000	16012500
groupIV	16210500	16211500
groupIV	16430000	16432500
groupIV	16528500	16529500
groupIV	16576500	16577500
groupIV	16692000	16693500
groupIV	16755000	16758500
groupIV	16783000	16803000
groupIV	16818000	16823500
groupIV	16838500	16845000
groupIV	16879500	16880500

scaffold_68	734500	787000
scaffold_69	747000	756000
scaffold_702	0	11000
scaffold_74	247000	252000
scaffold_777	0	10500
scaffold_797	0	10000
scaffold_844	0	9500
scaffold_88	31500	63000
scaffold_90	193500	202000
scaffold_94	175500	215000
scaffold_94	228500	517500
scaffold_95	0	67000
scaffold_98	219500	296500
scaffold_99	367500	381500
scaffold_99	398000	421000

groupIV	16904000	16907000
groupIV	16958500	16960000
groupIV	16979000	16981500
groupIV	17083000	17084000
groupIV	17141000	17152500
groupIV	17193500	17196000
groupIV	17257500	17260000
groupIV	17357000	17360000
groupIV	17364000	17372500
groupIV	17378000	17381000
groupIV	17424500	17430500
groupIV	17434000	17437500
groupIV	17444000	17446000
groupIV	17447000	17449000
groupIV	17473500	17480000
groupIV	17525500	17529000
groupIV	17539500	17545500
groupIV	17557000	17559500
groupIV	17561000	17563500
groupIV	17601500	17607000
groupIV	17641000	17649500
groupIV	17651500	17704000
groupIV	17958000	17985000
groupIV	18105500	18107000
groupIV	18703000	18704000
groupIV	18704500	18706500
groupIV	18820000	18823000
groupIV	19068000	19069500
groupIV	19081500	19082500
groupIV	19174500	19177000
groupIV	19221500	19229000
groupIV	19244500	19251000
groupIV	19535000	19543500
groupIV	19581000	19582000

groupIV	19591500	19592500
groupIV	19653000	19655000
groupIV	19725500	19726500
groupIV	19754000	19765000
groupIV	19793500	19795000
groupIV	19807500	19809500
groupIV	19822500	19826000
groupIV	19836000	19848500
groupIV	19922000	19935000
groupIV	19936500	19940500
groupIV	19954500	19963500
groupIV	19988500	19990000
groupIV	20092500	20093500
groupIV	20130000	20137500
groupIV	20195500	20197000
groupIV	20198000	20199500
groupIV	20233000	20235500
groupIV	20385000	20387000
groupIV	20408500	20410500
groupIV	20496500	20498000
groupIV	20540500	20544500
groupIV	20552500	20557000
groupIV	20563500	20564500
groupIV	20581500	20585000
groupIV	20697000	20698000
groupIV	20785000	20786000
groupIV	20797000	20798000
groupIV	20995500	20997000
groupIV	21017000	21020500
groupIV	21034500	21047000
groupIV	21047500	21192000
groupIV	21210000	21221500
groupIV	21293500	21296500
groupIV	21328000	21338000

groupIV	21344000	21345500
groupIV	21424500	21426000
groupIV	21459500	21463000
groupIV	21494500	21497000
groupIV	21716500	21723500
groupIV	21755000	21757500
groupIV	21812500	21815500
groupIV	21818000	21821000
groupIV	21823000	21824500
groupIV	21925500	21945000
groupIV	22014500	22020000
groupIV	22045500	22047500
groupIV	22127000	22139500
groupIV	22160500	22165000
groupIV	22179000	22190500
groupIV	22230500	22234500
groupIV	22265000	22267500
groupIV	22376000	22380000
groupIV	22394000	22397500
groupIV	22429000	22434000
groupIV	22483500	22486500
groupIV	22613500	22619500
groupIV	22652500	22669000
groupIV	22703000	22705500
groupIV	22785500	22786500
groupIV	22930500	22934500
groupIV	23094000	23103500
groupIV	23470000	23471000
groupIV	23500500	23504500
groupIV	23518500	23520500
groupIV	23534500	23536000
groupIV	23597500	23599500
groupIV	23651000	23656500
groupIV	23660000	23661500

groupIV	23767000	23768500
groupIV	23875000	23900000
groupIV	23904000	23905000
groupIV	23949500	23956500
groupIV	23992500	23993500
groupIV	24002500	24004500
groupIV	24009500	24015500
groupIV	24040500	24041500
groupIV	24150500	24152500
groupIV	24188000	24191500
groupIV	24237500	24238500
groupIV	24248500	24257000
groupIV	24274000	24275500
groupIV	24312000	24313500
groupIV	24316000	24319000
groupIV	24407000	24408000
groupIV	24678500	24680000
groupIV	24730500	24734500
groupIV	24749000	24755000
groupIV	24789000	24790000
groupIV	24812500	24815000
groupIV	24839000	24841500
groupIV	24859500	24860500
groupIV	24869500	24870500
groupIV	24875500	24877500
groupIV	24882500	24884000
groupIV	24893500	24910500
groupIV	24915000	24934000
groupIV	24951000	24953000
groupIV	24989500	24991500
groupIV	24997500	24999500
groupIV	25003500	25006000
groupIV	25018500	25021000
groupIV	25081000	25082500

groupIV	25130000	25132500
groupIV	25276500	25278000
groupIV	25425000	25428000
groupIV	25451500	25467000
groupIV	25471000	25492500
groupIV	25494500	25503500
groupIV	25531000	25551000
groupIV	25566000	25567000
groupIV	25567500	25612000
groupIV	25619500	25622500
groupIV	25630500	25664000
groupIV	25679000	25681000
groupIV	25687000	25730500
groupIV	25743000	25751000
groupIV	25820500	25829000
groupIV	25965000	25967500
groupIV	25982500	25985000
groupIV	26001000	26009500
groupIV	26030500	26032000
groupIV	26044500	26047000
groupIV	26052000	26067500
groupIV	26073000	26079500
groupIV	26089000	26121500
groupIV	26122500	26126500
groupIV	26173000	26183000
groupIV	26419500	26441500
groupIV	26503500	26530500
groupIV	26543000	26544500
groupIV	26619500	26621500
groupIV	26648000	26654000
groupIV	26676000	26678000
groupIV	26682500	26686000
groupIV	26696000	26699500
groupIV	26723500	26727500

groupIV	26808500	26810500
groupIV	26817500	26823000
groupIV	26894500	26899000
groupIV	26916000	26917000
groupIV	26998000	26999500
groupIV	27011500	27012500
groupIV	27072000	27073500
groupIV	27100500	27101500
groupIV	27125000	27126500
groupIV	27128500	27129500
groupIV	27133500	27140000
groupIV	27167000	27178000
groupIV	27226500	27232500
groupIV	27306500	27307500
groupIV	27389500	27393000
groupIV	27400500	27404000
groupIV	27427500	27429000
groupIV	27517000	27520000
groupIV	27537000	27540000
groupIV	27588500	27606000
groupIV	27784000	27785000
groupIV	27809500	27810500
groupIV	27824500	27835500
groupIV	27841000	27843000
groupIV	27851000	27852500
groupIV	28013000	28017000
groupIV	28095000	28098500
groupIV	28138500	28182000
groupIV	28196000	28205500
groupIV	28228000	28232000
groupIV	28259000	28261000
groupIV	28273000	28277500
groupIV	28283000	28290500
groupIV	28495500	28497000

groupIV	28626500	28627500
groupIV	28663500	28665500
groupIV	28927000	28930000
groupIV	29001500	29003000
groupIV	29133000	29134000
groupIV	29354000	29356500
groupIV	29369000	29371000
groupIV	29556000	29557000
groupIV	30377500	30378500
groupIV	30425000	30426500
groupIV	30482000	30484500
groupIV	30613500	30702000
groupIV	30724500	30727000
groupIV	31114000	31115500
groupIV	31285000	31287500
groupIV	31388500	31391500
groupIV	31463500	31465000
groupIV	31481500	31483500
groupIV	31525500	31526500
groupIV	31745000	31749000
groupIV	31823000	31824000
groupIV	31897500	31899000
groupIV	31992000	31995500
groupIV	32056000	32058500
groupIV	32063000	32066000
groupIV	32166500	32168500
groupIV	32204000	32208000
groupIV	32257000	32259500
groupIV	32265500	32266500
groupIV	32440500	32457500
groupIV	32566000	32575000
groupIX	205500	207000
groupIX	249000	256500
groupIX	262500	264000

groupIX	533500	537000
groupIX	777000	790500
groupIX	957000	1104500
groupIX	1133500	1134500
groupIX	1190000	1191500
groupIX	1453500	1458000
groupIX	1484000	1487000
groupIX	1625000	1626500
groupIX	1814500	1858500
groupIX	1875000	1876500
groupIX	1908500	1913000
groupIX	1933000	1934000
groupIX	1936500	1940000
groupIX	1961500	1966000
groupIX	2011000	2023500
groupIX	2062000	2074500
groupIX	2103000	2105500
groupIX	2108500	2109500
groupIX	2312000	2316000
groupIX	2524500	2525500
groupIX	2535500	2538000
groupIX	2594000	2608000
groupIX	2740000	2743000
groupIX	2846000	2848000
groupIX	2915500	2916500
groupIX	2935500	2936500
groupIX	2950500	2958500
groupIX	3009000	3010500
groupIX	3057000	3062000
groupIX	3064500	3069500
groupIX	3137000	3139000
groupIX	3189500	3200500
groupIX	3357500	3364500
groupIX	3424500	3430000

groupIX	3439500	3445500
groupIX	3450500	3452000
groupIX	3530000	3532500
groupIX	3546500	3551500
groupIX	3595500	3596500
groupIX	3781000	3782500
groupIX	4169000	4170500
groupIX	4279500	4282000
groupIX	4304500	4307500
groupIX	4855000	4877000
groupIX	5428500	5429500
groupIX	5449000	5452500
groupIX	5741000	5744000
groupIX	5769500	5772000
groupIX	5919000	5921000
groupIX	6045500	6047000
groupIX	6062000	6063500
groupIX	6264000	6265000
groupIX	7211500	7215000
groupIX	7932000	7933000
groupIX	7997500	7999500
groupIX	8297500	8298500
groupIX	8321000	8325000
groupIX	8831000	8841000
groupIX	8937500	8946500
groupIX	8962500	8964000
groupIX	8986500	8988000
groupIX	9002500	9003500
groupIX	9102500	9106000
groupIX	9106500	9121500
groupIX	9280500	9281500
groupIX	9351000	9352500
groupIX	9421000	9422500
groupIX	9437500	9465500

groupIX	9473000	9483500
groupIX	9491500	9493000
groupIX	9496500	9498500
groupIX	9641000	9642000
groupIX	9922500	9923500
groupIX	10215500	10216500
groupIX	10223500	10225000
groupIX	10354500	10360000
groupIX	10396000	10398000
groupIX	10400500	10402000
groupIX	10460500	10471500
groupIX	10518000	10519000
groupIX	10612000	10627000
groupIX	10689500	10713500
groupIX	11215000	11219500
groupIX	11344000	11345500
groupIX	11368500	11369500
groupIX	11419000	11421000
groupIX	11733500	11735500
groupIX	11766000	11767000
groupIX	11769500	11770500
groupIX	11799000	11801000
groupIX	11992000	12000000
groupIX	12047000	12061000
groupIX	12081000	12084000
groupIX	12134500	12136500
groupIX	12214000	12215000
groupIX	12239000	12241500
groupIX	12447000	12449500
groupIX	12481500	12483000
groupIX	12554000	12557500
groupIX	12669500	12670500
groupIX	12696000	12707500
groupIX	12787000	12790500

groupIX	12857500	12859000
groupIX	12885000	12888000
groupIX	12893000	12894500
groupIX	13033500	13037500
groupIX	13068500	13084000
groupIX	13107000	13109000
groupIX	13247500	13251000
groupIX	13253500	13254500
groupIX	13425000	13465000
groupIX	13655000	13659000
groupIX	13769000	13775500
groupIX	13857000	13862000
groupIX	13870000	13873500
groupIX	13881000	13884500
groupIX	14018500	14019500
groupIX	14095000	14098000
groupIX	14162500	14164000
groupIX	14172000	14178000
groupIX	14184000	14188500
groupIX	14197000	14198000
groupIX	14338000	14340000
groupIX	14477500	14478500
groupIX	14612000	14678000
groupIX	14762000	14767000
groupIX	14840500	14844000
groupIX	15144000	15158500
groupIX	15159000	15160500
groupIX	15230000	15231000
groupIX	15331500	15338500
groupIX	15820000	15823000
groupIX	16211500	16214000
groupIX	16215000	16217500
groupIX	16442500	16452000
groupIX	16453500	16456000

groupIX	16567500	16568500
groupIX	16585000	16587500
groupIX	16609000	16613500
groupIX	16625500	16626500
groupIX	16666000	16667000
groupIX	16738000	16740500
groupIX	16986500	16989500
groupIX	17022500	17023500
groupIX	17039000	17040000
groupIX	17040500	17042000
groupIX	17101000	17122500
groupIX	17263500	17265500
groupIX	17334000	17336000
groupIX	17493000	17495000
groupIX	17815500	17817500
groupIX	18007500	18021500
groupIX	18152500	18153500
groupIX	18209500	18217000
groupIX	18353500	18371500
groupIX	18378000	18379000
groupIX	18394500	18397000
groupIX	18477000	18478500
groupIX	18517000	18518000
groupIX	18534500	18649500
groupIX	18694000	18695500
groupIX	18714500	18731500
groupIX	18749000	18759000
groupIX	19012000	19013500
groupIX	19089000	19213500
groupIX	19218000	19224000
groupIX	19355500	19477500
groupIX	19483000	19487500
groupIX	19510500	19517000
groupIX	19607000	19622000

groupIX	19624000	19627500
groupIX	19687000	19730500
groupIX	19827500	19829000
groupIX	19840500	19854500
groupIX	19860000	19861500
groupIX	19872500	19874500
groupIX	19893000	19894000
groupIX	19901000	19917000
groupIX	19932500	19933500
groupV	4000	9500
groupV	23000	28000
groupV	296500	300000
groupV	574500	575500
groupV	603000	604000
groupV	628000	629500
groupV	870500	872000
groupV	881500	884500
groupV	1034000	1035000
groupV	1199500	1200500
groupV	1350000	1360500
groupV	1545500	1550000
groupV	1701500	1702500
groupV	2042500	2043500
groupV	2127000	2128000
groupV	2178500	2187500
groupV	2256000	2310500
groupV	2343000	2420500
groupV	2438500	2475000
groupV	2489500	2494000
groupV	2497500	2500500
groupV	2708500	2712000
groupV	2804000	2839500
groupV	3053500	3062500
groupV	3376500	3381000

groupV	3780000	3788500
groupV	3800500	3803500
groupV	4201500	4209000
groupV	4227000	4230000
groupV	4246500	4248500
groupV	4318000	4323500
groupV	4431000	4432000
groupV	4440500	4441500
groupV	4453500	4454500
groupV	4726000	4728000
groupV	4774500	4782000
groupV	4784000	4785000
groupV	4849500	4853500
groupV	4871500	4873000
groupV	4909500	4914000
groupV	5220000	5222000
groupV	5407000	5423500
groupV	5428500	5432000
groupV	5493000	5494000
groupV	5563500	5574000
groupV	6218500	6222500
groupV	6223500	6226000
groupV	6281000	6284500
groupV	6410500	6412000
groupV	6430500	6432000
groupV	6550500	6557500
groupV	6585000	6586000
groupV	6647000	6648500
groupV	6650000	6654000
groupV	6731500	6736000
groupV	6742500	6744000
groupV	6765000	6767500
groupV	6797000	6804500
groupV	6858500	6860000

groupV	6896000	6899500
groupV	6930500	6935000
groupV	7030000	7031000
groupV	7061500	7064500
groupV	7068000	7070000
groupV	7119000	7120000
groupV	7156000	7159500
groupV	7313500	7315000
groupV	7339000	7347000
groupV	7375500	7376500
groupV	7407000	7413500
groupV	7490000	7493000
groupV	7503000	7505500
groupV	7538500	7540000
groupV	7708000	7709500
groupV	7719500	7722000
groupV	7762000	7765500
groupV	7778000	7779500
groupV	7808000	7813500
groupV	8005500	8007000
groupV	8599500	8601000
groupV	8732000	8734500
groupV	9101500	9114000
groupV	9277000	9279000
groupV	9346500	9359500
groupV	9389000	9391000
groupV	9392000	9421000
groupV	9463500	9466000
groupV	9487500	9488500
groupV	9501500	9512000
groupV	9667500	9669500
groupV	9674000	9678000
groupV	9698000	9739000
groupV	9751500	9756000

groupV	9792000	9793500
groupV	9798500	9800500
groupV	9940000	9942000
groupV	9995500	9998000
groupV	10126500	10127500
groupV	10146500	10147500
groupV	10343000	10345500
groupV	10379000	10381000
groupV	10567500	10573000
groupV	10715500	10757500
groupV	11063500	11067000
groupV	11068500	11070000
groupV	11169500	11171000
groupV	11188000	11189500
groupV	11356500	11359000
groupV	11442500	11443500
groupV	11495000	11496000
groupV	11597000	11598000
groupV	11645500	11647500
groupV	11960000	11964000
groupV	11996500	11998500
groupV	12054000	12056000
groupV	12087500	12096000
groupVI	6500	9500
groupVI	140000	141500
groupVI	224000	230000
groupVI	242500	244500
groupVI	274000	275500
groupVI	375500	415500
groupVI	421500	423500
groupVI	443000	445000
groupVI	508500	512500
groupVI	561500	564500
groupVI	675500	677000

groupVI	753500	757000
groupVI	800000	802000
groupVI	822000	823500
groupVI	837500	839000
groupVI	1271500	1276500
groupVI	1356500	1362000
groupVI	1499000	1500500
groupVI	1697500	1699500
groupVI	1801000	1807500
groupVI	1818500	1819500
groupVI	2029000	2030500
groupVI	2274500	2276000
groupVI	2290500	2305000
groupVI	2369000	2370500
groupVI	2398000	2399500
groupVI	2405500	2406500
groupVI	2460000	2463000
groupVI	2570000	2571000
groupVI	2579000	2591500
groupVI	2593500	2600500
groupVI	2841000	2842500
groupVI	2994500	3000500
groupVI	3096000	3097000
groupVI	3279000	3289000
groupVI	3324000	3325500
groupVI	3569500	3572500
groupVI	3726500	3732000
groupVI	3904000	3909500
groupVI	3918000	3924000
groupVI	4267000	4268500
groupVI	4612500	4615000
groupVI	4680000	4681500
groupVI	5346000	5348000
groupVI	5586000	5594000

groupVI	5783000	5786000
groupVI	5911000	5912500
groupVI	6166000	6167000
groupVI	6189000	6190500
groupVI	6532500	6538000
groupVI	6569500	6571500
groupVI	6768500	6771500
groupVI	6877000	6879000
groupVI	7214500	7216000
groupVI	7384000	7385500
groupVI	7544000	7546000
groupVI	7961000	7969500
groupVI	8290500	8291500
groupVI	9513500	9514500
groupVI	9909000	9911500
groupVI	9933500	9944000
groupVI	10414500	10507500
groupVI	10913500	10916000
groupVI	11361500	11362500
groupVI	11601000	11602000
groupVI	11620500	11622500
groupVI	11689500	11692000
groupVI	11747500	11749000
groupVI	11776500	11778500
groupVI	11844000	11846000
groupVI	12005000	12008500
groupVI	12559500	12568000
groupVI	13016000	13017000
groupVI	13156500	13157500
groupVI	13559500	13560500
groupVI	13749000	13750500
groupVI	13766500	13767500
groupVI	13824500	13826000
groupVI	13967500	13984500

groupVI	14034500	14039500
groupVI	14113000	14114000
groupVI	14145500	14146500
groupVI	14251000	14252000
groupVI	14525000	14528000
groupVI	14726500	14730500
groupVI	14892000	14897500
groupVI	15064500	15066500
groupVI	15071000	15078000
groupVI	15111000	15113000
groupVI	15126000	15148000
groupVI	15190500	15191500
groupVI	15239000	15246000
groupVI	15369000	15376000
groupVI	15474500	15484500
groupVI	15607000	15609000
groupVI	15736500	15743000
groupVI	15753000	15758000
groupVI	15788000	15789500
groupVI	15852000	15853000
groupVI	15940500	15942500
groupVI	16011000	16014500
groupVI	16352000	16353500
groupVI	16503000	16504000
groupVI	16691500	16694500
groupVI	16825500	16826500
groupVI	16830500	16832000
groupVI	16884500	16892500
groupVII	55000	62000
groupVII	98000	125500
groupVII	126500	128000
groupVII	132000	135000
groupVII	135500	141000
groupVII	146500	159500

groupVII	166500	170500
groupVII	188000	196000
groupVII	214500	221500
groupVII	263500	265000
groupVII	290000	292000
groupVII	293000	313000
groupVII	350000	354000
groupVII	399000	403500
groupVII	421500	494500
groupVII	503500	509500
groupVII	514000	520500
groupVII	823500	825000
groupVII	862500	874500
groupVII	907000	910000
groupVII	996000	997000
groupVII	1023500	1028000
groupVII	1044500	1100000
groupVII	1242000	1243500
groupVII	1246500	1248000
groupVII	1275000	1278000
groupVII	1299500	1301500
groupVII	1303000	1305500
groupVII	1416500	1440500
groupVII	1535500	1541500
groupVII	1555500	1573000
groupVII	1592000	1621000
groupVII	1661500	1665500
groupVII	2275500	2298500
groupVII	2328000	2343500
groupVII	2347000	2348500
groupVII	2437000	2438500
groupVII	2440500	2463000
groupVII	2497000	2498000
groupVII	2598500	2602500

groupVII	2634000	2652000
groupVII	2779500	2783000
groupVII	2945500	2951500
groupVII	2993500	3034500
groupVII	3316000	3319500
groupVII	3416000	3418500
groupVII	3433500	3435500
groupVII	3836500	3839000
groupVII	3842000	3853000
groupVII	3890500	3892500
groupVII	3920000	3921000
groupVII	3964000	3967500
groupVII	4062000	4087000
groupVII	4171500	4173500
groupVII	4202500	4203500
groupVII	4279000	4281000
groupVII	4301500	4303500
groupVII	4681500	4683000
groupVII	4685500	4689000
groupVII	4786500	4790500
groupVII	4793000	4795500
groupVII	4809000	4811000
groupVII	4838500	4842000
groupVII	4961500	4964000
groupVII	4970000	4972000
groupVII	6141000	6144500
groupVII	6396500	6400500
groupVII	6514500	6516000
groupVII	6899500	6901500
groupVII	6905500	6926000
groupVII	6930000	6933000
groupVII	7094000	7095000
groupVII	7228500	7230500
groupVII	7345000	7346000

groupVII	7471000	7472500
groupVII	7594500	7596000
groupVII	7633500	7636000
groupVII	7664500	7666500
groupVII	7731500	7733000
groupVII	7981500	7989000
groupVII	8004500	8006500
groupVII	8045000	8061000
groupVII	8113000	8114000
groupVII	8114500	8115500
groupVII	8633500	8641000
groupVII	8739000	8742000
groupVII	8851000	8852500
groupVII	8953500	8957000
groupVII	8988000	8992500
groupVII	8993000	8997500
groupVII	9008000	9013000
groupVII	9034500	9037500
groupVII	9040500	9148500
groupVII	9154000	9167500
groupVII	9178500	9180500
groupVII	9183500	9187500
groupVII	9190000	9229500
groupVII	9241000	9243000
groupVII	9312000	9313000
groupVII	9336000	9340500
groupVII	9358000	9359500
groupVII	9409500	9414500
groupVII	9500000	9504500
groupVII	9555500	9557000
groupVII	9618000	9620500
groupVII	9624000	9625000
groupVII	9629500	9632500
groupVII	9649500	9652500

groupVII	9677000	9681500
groupVII	9727000	9729500
groupVII	9767000	9768000
groupVII	9852000	9853000
groupVII	10031500	10035500
groupVII	10080000	10081500
groupVII	10115500	10118000
groupVII	10181000	10182000
groupVII	10214500	10294500
groupVII	10308000	10312000
groupVII	10615000	10618500
groupVII	10624000	10625500
groupVII	10645000	10646500
groupVII	10745500	10750000
groupVII	10793000	10795500
groupVII	10805500	10806500
groupVII	10882500	10885500
groupVII	11073500	11074500
groupVII	11347500	11349000
groupVII	11375000	11378500
groupVII	11448000	11450500
groupVII	11451500	11456000
groupVII	11518000	11520000
groupVII	11601500	11606000
groupVII	11647500	11651500
groupVII	11703000	11705000
groupVII	11788500	11795000
groupVII	12006000	12008500
groupVII	12041000	12043500
groupVII	12084500	12090500
groupVII	12226500	12228000
groupVII	12263500	12265000
groupVII	12294000	12296500
groupVII	12315000	12316500

groupVII	12324500	12326500
groupVII	12331000	12332500
groupVII	12428000	12435500
groupVII	12501000	12502000
groupVII	12525000	12526000
groupVII	12588000	12589000
groupVII	12643000	12649500
groupVII	12691500	12692500
groupVII	12699000	12702000
groupVII	12743000	12744000
groupVII	12795500	12797000
groupVII	12869000	12874000
groupVII	12875500	12887000
groupVII	12965500	12968500
groupVII	12971000	12973000
groupVII	13013500	13023500
groupVII	13024500	13027500
groupVII	13083500	13086500
groupVII	13109000	13111000
groupVII	13197000	13203500
groupVII	13316000	13317500
groupVII	13350500	13356000
groupVII	13426000	13428500
groupVII	13467000	13469000
groupVII	13496000	13498500
groupVII	13814500	13815500
groupVII	13838000	13839500
groupVII	13850500	13858000
groupVII	13921500	13923500
groupVII	14041500	14042500
groupVII	14049000	14050500
groupVII	14169500	14171000
groupVII	14175500	14185000
groupVII	14192000	14193000

groupVII	14334500	14341000
groupVII	14388000	14389500
groupVII	14390000	14391000
groupVII	14480000	14481000
groupVII	14492500	14493500
groupVII	14520500	14522000
groupVII	14631500	14634500
groupVII	14636000	14637500
groupVII	14780000	14781000
groupVII	14785500	14789500
groupVII	15008500	15011500
groupVII	15214000	15217500
groupVII	15362000	15364000
groupVII	15535500	15538000
groupVII	15744500	15746000
groupVII	15957500	15958500
groupVII	16101500	16102500
groupVII	16120500	16122500
groupVII	16337000	16338000
groupVII	16480000	16481000
groupVII	16637000	16643500
groupVII	17431000	17434000
groupVII	17460500	17462000
groupVII	17538000	17539000
groupVII	17644500	17645500
groupVII	17730000	17755000
groupVII	18237000	18251500
groupVII	18332500	18333500
groupVII	18372500	18374500
groupVII	18426000	18434000
groupVII	18713000	18718000
groupVII	18905500	18917000
groupVII	19026000	19046500
groupVII	19319500	19320500

groupVII	19323000	19324500
groupVII	19325500	19328500
groupVII	19352500	19353500
groupVII	19418500	19421500
groupVII	19445000	19447000
groupVII	19505500	19506500
groupVII	19666000	19672500
groupVII	19681500	19694500
groupVII	19704000	19726000
groupVII	19727500	19730000
groupVII	19740500	19756000
groupVII	19761000	19793500
groupVII	19835000	19836500
groupVII	20425500	20426500
groupVII	20553500	20555000
groupVII	20617500	20620000
groupVII	20815000	20816000
groupVII	21220000	21221000
groupVII	21426000	21428500
groupVII	21445500	21496000
groupVII	21601000	21602000
groupVII	21782500	21783500
groupVII	21970000	21971500
groupVII	21984000	21985000
groupVII	22014000	22016000
groupVII	22341500	22343000
groupVII	22600000	22602000
groupVII	23913500	23914500
groupVII	24077500	24078500
groupVII	24691500	24721000
groupVII	24794500	24795500
groupVII	24930000	24931000
groupVII	25142000	25143500
groupVII	25445000	25453000

groupVII	25613500	25618000
groupVII	25623500	25626000
groupVII	25638500	25639500
groupVII	25643500	25644500
groupVII	25711500	25712500
groupVII	25724500	25727000
groupVII	25855500	25856500
groupVII	25927500	25929500
groupVII	26005000	26007000
groupVII	26007500	26022500
groupVII	26041000	26042000
groupVII	26090500	26093000
groupVII	26094000	26105000
groupVII	26229500	26231500
groupVII	26277000	26279000
groupVII	26370000	26383500
groupVII	26456500	26469500
groupVII	26540000	26553000
groupVII	26634000	26635000
groupVII	27145000	27164500
groupVII	27447000	27459500
groupVII	27582000	27588000
groupVII	27661500	27665000
groupVII	27670500	27672500
groupVII	27680500	27681500
groupVII	27763500	27768000
groupVII	27889500	27893500
groupVIII	39500	42000
groupVIII	149000	151000
groupVIII	251500	270500
groupVIII	326500	328500
groupVIII	358500	360000
groupVIII	369000	373500
groupVIII	408500	416500

groupVIII	522500	529000
groupVIII	531000	532500
groupVIII	638000	639500
groupVIII	872000	885500
groupVIII	939000	940500
groupVIII	954500	956000
groupVIII	978000	979000
groupVIII	989000	991500
groupVIII	1045000	1061500
groupVIII	1102500	1105000
groupVIII	1189500	1193000
groupVIII	1260500	1261500
groupVIII	1266000	1292500
groupVIII	1298500	1301500
groupVIII	1338000	1341000
groupVIII	1372500	1412500
groupVIII	1438500	1440000
groupVIII	1474500	1475500
groupVIII	1476000	1479000
groupVIII	1484000	1486500
groupVIII	1495500	1501500
groupVIII	1508500	1510500
groupVIII	1511000	1513000
groupVIII	1541000	1545000
groupVIII	1559000	1560000
groupVIII	1563000	1564500
groupVIII	2062000	2064500
groupVIII	2936500	2950000
groupVIII	3195500	3196500
groupVIII	3639000	3640000
groupVIII	3936500	3941500
groupVIII	4010500	4013500
groupVIII	4065000	4066000
groupVIII	4224000	4225000

groupVIII	4247500	4249000
groupVIII	4328500	4329500
groupVIII	4374500	4406500
groupVIII	4412500	4502000
groupVIII	4536500	4541000
groupVIII	4782000	4789500
groupVIII	4790000	4806000
groupVIII	4814500	4851000
groupVIII	4942500	4944000
groupVIII	5018500	5019500
groupVIII	5045000	5050500
groupVIII	5080500	5082000
groupVIII	5083000	5100000
groupVIII	5139500	5142000
groupVIII	5313000	5314000
groupVIII	5316500	5319000
groupVIII	5511500	5513500
groupVIII	5514000	5522500
groupVIII	5745000	5746000
groupVIII	5794000	5819000
groupVIII	5825000	5827000
groupVIII	6636000	6637000
groupVIII	6873000	6876500
groupVIII	7354000	7360500
groupVIII	7446000	7447500
groupVIII	7788000	7791000
groupVIII	7814000	7816000
groupVIII	7869000	7874500
groupVIII	7888500	7890000
groupVIII	7934500	7935500
groupVIII	7963500	7965000
groupVIII	8191500	8192500
groupVIII	8460500	8461500
groupVIII	8533000	8534000

groupVIII	8676500	8679000
groupVIII	8942500	8949000
groupVIII	9297500	9299000
groupVIII	9341500	9343000
groupVIII	9355000	9360000
groupVIII	9388500	9389500
groupVIII	9656000	9659500
groupVIII	9753500	9754500
groupVIII	9805500	9818000
groupVIII	9833500	9835500
groupVIII	10050000	10074500
groupVIII	10216500	10220500
groupVIII	10323000	10324000
groupVIII	10509500	10511000
groupVIII	10520000	10522500
groupVIII	11071500	11078000
groupVIII	11128500	11130000
groupVIII	11314500	11317000
groupVIII	11586000	11591000
groupVIII	11803000	11810000
groupVIII	12456000	12462000
groupVIII	13944000	13947000
groupVIII	14338000	14347000
groupVIII	14512000	14513500
groupVIII	14617000	14622500
groupVIII	14727000	14729000
groupVIII	14882000	14884000
groupVIII	14886000	14887000
groupVIII	14918000	14919000
groupVIII	14939000	14942000
groupVIII	15146500	15149500
groupVIII	15159500	15161500
groupVIII	15166500	15169000
groupVIII	15684000	15685500

groupVIII	16064500	16072500
groupVIII	16117500	16124000
groupVIII	16408000	16410000
groupVIII	16488000	16491500
groupVIII	16519500	16529000
groupVIII	16582500	16584500
groupVIII	16850000	16852000
groupVIII	16898000	16900000
groupVIII	16917000	16919000
groupVIII	17033500	17043500
groupVIII	17045500	17047500
groupVIII	17066000	17069500
groupVIII	17156500	17163000
groupVIII	17172500	17174500
groupVIII	17185500	17210500
groupVIII	17264500	17268000
groupVIII	18078000	18080000
groupVIII	18154000	18155000
groupVIII	18218500	18221000
groupVIII	18260500	18262000
groupVIII	18264500	18269500
groupVIII	18271500	18273000
groupVIII	18366000	18373500
groupVIII	18422500	18425500
groupVIII	18533000	18537500
groupVIII	18542000	18544000
groupVIII	18625000	18629500
groupVIII	18703500	18718500
groupVIII	18736000	18738000
groupVIII	18857500	18885500
groupVIII	18988500	18990000
groupVIII	19140000	19141500
groupVIII	19187500	19190000
groupVIII	19343500	19352500

groupX	7500	10500
groupX	144000	146500
groupX	153500	155500
groupX	293000	295500
groupX	308000	309500
groupX	319000	335000
groupX	387000	389500
groupX	394500	398500
groupX	400500	938000
groupX	1042500	1153500
groupX	1178000	1179000
groupX	1391000	1394000
groupX	1735000	1746500
groupX	1755500	1757000
groupX	1987500	1989500
groupX	1995000	1998000
groupX	2139500	2143500
groupX	2705500	2708000
groupX	2742500	2744500
groupX	2823500	2826500
groupX	2847500	2849500
groupX	2898000	2899000
groupX	2929000	2930500
groupX	3018000	3019500
groupX	3084500	3086000
groupX	3480000	3481000
groupX	3492500	3498500
groupX	3544500	3545500
groupX	3581500	3583000
groupX	3717500	3718500
groupX	3946500	3950000
groupX	4289000	4290000
groupX	4299500	4301500
groupX	4615000	4619500

groupX	5217000	5219500
groupX	5273000	5274000
groupX	5542000	5543000
groupX	5756500	5764000
groupX	5893500	5894500
groupX	5938000	5940500
groupX	5956500	5957500
groupX	6119500	6120500
groupX	6278000	6281500
groupX	6339500	6344000
groupX	6362000	6375000
groupX	6447500	6486500
groupX	6510500	6512000
groupX	7014000	7034000
groupX	7225000	7299000
groupX	7890500	7893500
groupX	8245000	8247500
groupX	8843500	8852000
groupX	9142500	9164000
groupX	9165000	9166500
groupX	9193500	9194500
groupX	9235500	9240000
groupX	9532500	9533500
groupX	10180500	10183500
groupX	10238000	10239000
groupX	10708500	10709500
groupX	11272500	11278000
groupX	11282000	11283500
groupX	11530500	11532000
groupX	11748000	11754000
groupX	11837000	11838000
groupX	12002500	12006000
groupX	12081500	12083000
groupX	12159500	12160500

groupX	12226500	12228000
groupX	12259000	12261500
groupX	12281000	12282000
groupX	12507000	12513000
groupX	12627000	12631500
groupX	12653000	12753500
groupX	12930500	12933500
groupX	13143500	13146500
groupX	13154000	13155500
groupX	13190000	13225500
groupX	13226000	13231000
groupX	13239000	13268000
groupX	13337500	13340500
groupX	13570000	13572000
groupX	13696500	13707000
groupX	13779000	13782000
groupX	13948500	13949500
groupX	13981500	13984500
groupX	14195000	14199500
groupX	14265000	14269000
groupX	14270500	14272500
groupX	14301500	14418500
groupX	14427000	14430500
groupX	14433000	14444500
groupX	14446000	14454000
groupX	14458500	14461000
groupX	14527500	14534000
groupX	14561000	14662500
groupX	14718000	14721000
groupX	14751000	14757000
groupX	14866500	14870500
groupX	14903500	14914500
groupX	14921500	14922500
groupX	15034000	15043000

groupX	15085500	15096500
groupX	15127000	15128500
groupX	15136000	15138000
groupX	15249000	15258500
groupX	15442000	15443000
groupX	15480500	15481500
groupX	15495500	15497000
groupX	15524000	15532000
groupX	15629000	15632000
groupXI	145500	146500
groupXI	207000	209000
groupXI	325000	327500
groupXI	344000	346000
groupXI	527500	529500
groupXI	534500	539500
groupXI	549000	551000
groupXI	694000	695500
groupXI	781000	782500
groupXI	787000	799000
groupXI	839000	841000
groupXI	848500	852000
groupXI	869500	871000
groupXI	955000	961000
groupXI	1055500	1058000
groupXI	1065500	1069000
groupXI	1070500	1071500
groupXI	1107000	1196000
groupXI	1201000	1203000
groupXI	1254000	1255000
groupXI	1307500	1308500
groupXI	1478000	1479500
groupXI	1523000	1535500
groupXI	1573000	1576000
groupXI	1902000	1906000

groupXI	1908500	1911000
groupXI	2035500	2039000
groupXI	2047500	2050500
groupXI	2112500	2117000
groupXI	2240000	2245000
groupXI	2358000	2360500
groupXI	2408500	2447000
groupXI	2464000	2471500
groupXI	2513500	2520500
groupXI	2828500	2829500
groupXI	2836500	2842000
groupXI	2843500	2847000
groupXI	2865500	2867000
groupXI	2910000	2918500
groupXI	3088500	3089500
groupXI	3209000	3213000
groupXI	3222000	3223500
groupXI	3459500	3460500
groupXI	3594500	3597000
groupXI	4069000	4076500
groupXI	4309500	4597000
groupXI	5016000	5019500
groupXI	5025500	5050500
groupXI	5108500	5109500
groupXI	5216500	5244500
groupXI	5505500	5506500
groupXI	5856500	5860500
groupXI	6311000	6313500
groupXI	6340500	6344000
groupXI	6425000	6426000
groupXI	6431000	6446000
groupXI	6450500	6511500
groupXI	6512500	6516500
groupXI	6579500	6581000

groupXI	7003500	7006500
groupXI	7033500	7035000
groupXI	7193000	7194500
groupXI	7508500	7509500
groupXI	7561000	7562000
groupXI	8224000	8226000
groupXI	8303000	8304000
groupXI	8457000	8459500
groupXI	8739000	8828000
groupXI	8859000	8861500
groupXI	8890000	8895000
groupXI	8898500	8899500
groupXI	8961000	8963000
groupXI	9206500	9211000
groupXI	9607000	9610000
groupXI	9647500	9672000
groupXI	9699500	9701500
groupXI	9712500	9797000
groupXI	9863500	9869000
groupXI	9956000	9957500
groupXI	9964500	9970500
groupXI	9995500	10002000
groupXI	10096000	10098500
groupXI	10200000	10204500
groupXI	10216500	10225500
groupXI	10233500	10275000
groupXI	10416500	10417500
groupXI	10672000	10674000
groupXI	10722500	10726500
groupXI	10867000	10871000
groupXI	11099000	11102500
groupXI	11136000	11139000
groupXI	11261000	11273500
groupXI	11445500	11451000

groupXI	11524000	11525000
groupXI	11543500	11555000
groupXI	11610000	11611500
groupXI	11626000	11627000
groupXI	11739500	11778000
groupXI	11778500	11836000
groupXI	12019000	12021000
groupXI	12064000	12070500
groupXI	12075500	12076500
groupXI	12082000	12091000
groupXI	12099500	12102000
groupXI	12235500	12237000
groupXI	12307500	12483500
groupXI	12682000	12703000
groupXI	12834500	12839000
groupXI	13131000	13133500
groupXI	13177500	13180500
groupXI	13614000	13625000
groupXI	13645000	13649500
groupXI	13845000	13852500
groupXI	13903000	13908000
groupXI	14022500	14023500
groupXI	14108000	14111500
groupXI	14329500	14331500
groupXI	14719000	14737000
groupXI	14847000	14849000
groupXI	15061500	15065000
groupXI	15066000	15068000
groupXI	15088000	15101500
groupXI	15337000	15338000
groupXI	15373500	15375500
groupXI	15420500	15430000
groupXI	15607500	15625000
groupXI	15627000	15630500

groupXI	15646000	15647500
groupXI	15858000	15863000
groupXI	15937000	15941500
groupXI	16014000	16015500
groupXI	16110500	16111500
groupXI	16119500	16124000
groupXI	16181500	16183000
groupXI	16200000	16202500
groupXI	16250000	16251000
groupXI	16267500	16269000
groupXI	16549000	16552000
groupXI	16597500	16600000
groupXII	65000	69500
groupXII	99000	117500
groupXII	146000	148500
groupXII	191000	222000
groupXII	255000	260000
groupXII	295500	297500
groupXII	304500	306000
groupXII	348000	359500
groupXII	383000	384000
groupXII	386000	406500
groupXII	422000	425500
groupXII	529500	533000
groupXII	766000	768500
groupXII	857500	859000
groupXII	883000	1046500
groupXII	1144500	1145500
groupXII	1152000	1168000
groupXII	1194500	1332000
groupXII	1559000	1560000
groupXII	1588500	1589500
groupXII	1680000	1690000
groupXII	1923000	1926000

groupXII	2188500	2203000
groupXII	2208000	2213500
groupXII	2272000	2275000
groupXII	2302000	2303500
groupXII	2494000	2495000
groupXII	2539000	2542500
groupXII	2576500	2577500
groupXII	2653500	2657000
groupXII	2669500	2672000
groupXII	2708500	2710500
groupXII	2717500	2720500
groupXII	2754000	2758500
groupXII	2831500	2833000
groupXII	2854500	2855500
groupXII	2918500	2920500
groupXII	2965000	2967500
groupXII	3051500	3058500
groupXII	3512000	3514500
groupXII	3758000	3760500
groupXII	3862500	3864000
groupXII	4000500	4008000
groupXII	4228500	4229500
groupXII	4250000	4251000
groupXII	4317500	4320500
groupXII	4399000	4403500
groupXII	4413500	4417000
groupXII	4730000	4734500
groupXII	4769000	4776000
groupXII	4898000	4900500
groupXII	5114000	5115500
groupXII	5433500	5435500
groupXII	6243000	6251000
groupXII	6441000	6451000
groupXII	6469500	6477000

groupXII	6484500	6511500
groupXII	6567500	6568500
groupXII	6724000	6726000
groupXII	6798000	6800500
groupXII	7045500	7048500
groupXII	7091000	7092000
groupXII	7160000	7163000
groupXII	7223500	7225500
groupXII	7230500	7234000
groupXII	7300500	7303000
groupXII	7305500	7309500
groupXII	7361500	7364500
groupXII	7389000	7404500
groupXII	7671500	7675000
groupXII	8006500	8008000
groupXII	8040000	8042500
groupXII	8043500	8047000
groupXII	8055000	8102000
groupXII	8250500	8252000
groupXII	8591000	8595000
groupXII	8801000	8805500
groupXII	8821000	8822500
groupXII	8919500	8921500
groupXII	9330000	9331500
groupXII	9518500	9519500
groupXII	9768000	9770500
groupXII	9788000	9790000
groupXII	9995000	10004500
groupXII	10054000	10057500
groupXII	10123000	10128000
groupXII	10266500	10270500
groupXII	10728500	10729500
groupXII	11752000	11755500
groupXII	11896500	11901500

groupXII	12127500	12129000
groupXII	12288000	12289500
groupXII	12332000	12347000
groupXII	12522500	12530500
groupXII	12854500	12855500
groupXII	12988500	12989500
groupXII	13280500	13281500
groupXII	13487000	13488000
groupXII	14227500	14228500
groupXII	14437500	14460000
groupXII	14739500	14740500
groupXII	14806500	14807500
groupXII	14822000	14823000
groupXII	14887500	14889500
groupXII	15508000	15509500
groupXII	15918500	15922000
groupXII	15951500	15956500
groupXII	16318000	16321000
groupXII	16457000	16469000
groupXII	16524000	16548500
groupXII	16581500	16586000
groupXII	16608500	16610000
groupXII	16734000	16738500
groupXII	16749000	16751000
groupXII	16758000	16761500
groupXII	16861000	16864500
groupXII	17026500	17027500
groupXII	17056000	17059000
groupXII	17102000	17106000
groupXII	17233500	17234500
groupXII	17351000	17353000
groupXII	17364500	17365500
groupXII	17372500	17374500
groupXII	17457500	17458500

groupXII	17464000	17465000
groupXII	17501500	17503500
groupXII	17530000	17533000
groupXII	17535000	17537000
groupXII	17629500	17634000
groupXII	17986000	17989000
groupXII	18018500	18020000
groupXII	18039500	18040500
groupXII	18154000	18166000
groupXII	18324000	18325000
groupXII	18358500	18369000
groupXII	18382000	18385500
groupXII	18393000	18397000
groupXIII	84500	87000
groupXIII	91500	92500
groupXIII	420500	422000
groupXIII	549500	551500
groupXIII	733500	735000
groupXIII	873000	876000
groupXIII	1035500	1040000
groupXIII	1066500	1071000
groupXIII	1138500	1140000
groupXIII	1169000	1194000
groupXIII	1273500	1276500
groupXIII	1337500	1338500
groupXIII	1485500	1487500
groupXIII	1542500	1545000
groupXIII	1587000	1588500
groupXIII	1604500	1607000
groupXIII	1996500	1999500
groupXIII	2464000	2467000
groupXIII	2490000	2500500
groupXIII	2519000	2520000
groupXIII	2999000	3001500

groupXIII	3026500	3027500
groupXIII	3134000	3165000
groupXIII	3173500	3184500
groupXIII	3262000	3267500
groupXIII	3274000	3293500
groupXIII	3299000	3302500
groupXIII	3312000	3315500
groupXIII	3331000	3333000
groupXIII	3455500	3457000
groupXIII	3564000	3566000
groupXIII	3669500	3675500
groupXIII	3681500	3694000
groupXIII	3695500	3699000
groupXIII	3762500	3765000
groupXIII	3942000	3943500
groupXIII	4073000	4074000
groupXIII	4178500	4179500
groupXIII	4211500	4376000
groupXIII	4517000	4518000
groupXIII	4613000	4615500
groupXIII	4689500	4691500
groupXIII	5020500	5033500
groupXIII	5089000	5092000
groupXIII	5142500	5144500
groupXIII	5227500	5228500
groupXIII	5240000	5244000
groupXIII	5257500	5258500
groupXIII	5371000	5372500
groupXIII	5500500	5504000
groupXIII	5548000	5551000
groupXIII	5612000	5613000
groupXIII	5686500	5687500
groupXIII	5750500	5753500
groupXIII	5956500	5959000

groupXIII	6230000	6231500
groupXIII	6331500	6335000
groupXIII	6426000	6427000
groupXIII	6699000	6700500
groupXIII	6833500	6835000
groupXIII	6905500	6909000
groupXIII	6915500	6917000
groupXIII	6989000	6990500
groupXIII	7053500	7057500
groupXIII	7070500	7075000
groupXIII	7768500	7769500
groupXIII	7801500	7802500
groupXIII	7872500	7875000
groupXIII	8047500	8052000
groupXIII	8072500	8073500
groupXIII	8074000	8075000
groupXIII	8185500	8187500
groupXIII	8310000	8316500
groupXIII	8805000	8809000
groupXIII	8816500	8820000
groupXIII	8822500	8824500
groupXIII	8978500	8990000
groupXIII	9008000	9020500
groupXIII	9124000	9125000
groupXIII	9603000	9605000
groupXIII	9751000	9758000
groupXIII	9776500	9779000
groupXIII	9929000	9930000
groupXIII	10082000	10083000
groupXIII	10179000	10181500
groupXIII	10658500	10660000
groupXIII	11343000	11344000
groupXIII	11365500	11368000
groupXIII	11431000	11438000

groupXIII	11547000	11548000
groupXIII	11776500	11780000
groupXIII	11784500	11802000
groupXIII	11826500	11831500
groupXIII	11842500	11844500
groupXIII	12018500	12019500
groupXIII	12166500	12168000
groupXIII	12438500	12446000
groupXIII	12513500	12522500
groupXIII	12588000	12589000
groupXIII	12767000	12768000
groupXIII	13315000	13316500
groupXIII	13649000	13651500
groupXIII	13915500	13917500
groupXIII	13960500	13970500
groupXIII	14105000	14106000
groupXIII	14207000	14208000
groupXIII	14242500	14245500
groupXIII	14615500	14616500
groupXIII	14620000	14622500
groupXIII	14700500	14702000
groupXIII	14885000	14886000
groupXIII	14949000	14950000
groupXIII	15183500	15184500
groupXIII	15510000	15513500
groupXIII	15944000	15945500
groupXIII	15988500	16002500
groupXIII	16247500	16249000
groupXIII	16305000	16307000
groupXIII	16448000	16450500
groupXIII	16584000	16597000
groupXIII	16624000	16645500
groupXIII	16701000	16702000
groupXIII	16762500	16798000

groupXIII	16811000	16813500
groupXIII	16884500	16890000
groupXIII	16903500	16906000
groupXIII	16934000	16935000
groupXIII	17024000	17025000
groupXIII	17106000	17109000
groupXIII	17116500	17120500
groupXIII	17182000	17183000
groupXIII	17210000	17211500
groupXIII	17309500	17312500
groupXIII	17417000	17433000
groupXIII	17466500	17468500
groupXIII	17626000	17627000
groupXIII	17703000	17704500
groupXIII	17774500	17778500
groupXIII	17791000	17792500
groupXIII	17918500	17921000
groupXIII	18034500	18036000
groupXIII	18074500	18079500
groupXIII	18083000	18085500
groupXIII	18136000	18137500
groupXIII	18190000	18194000
groupXIII	18247500	18250500
groupXIII	18326500	18328500
groupXIII	18335500	18343000
groupXIII	18395000	18403500
groupXIII	18443000	18448500
groupXIII	18457000	18610000
groupXIII	18647000	18648500
groupXIII	18680500	18683000
groupXIII	18685500	18689000
groupXIII	18702000	18760500
groupXIII	18768000	18769000
groupXIII	18800500	18802500

groupXIII	19042000	19044500
groupXIII	19162000	19163000
groupXIII	19298500	19299500
groupXIII	19352000	19355500
groupXIII	19457500	19475000
groupXIII	19479500	19493000
groupXIII	19884500	19887000
groupXIII	19965500	19979000
groupXIV	63500	68500
groupXIV	122500	126000
groupXIV	135000	136000
groupXIV	318500	324500
groupXIV	329000	330500
groupXIV	491000	492500
groupXIV	514500	520000
groupXIV	544500	551500
groupXIV	566000	599500
groupXIV	873000	875000
groupXIV	1155000	1157500
groupXIV	1226500	1228000
groupXIV	1507500	1509000
groupXIV	1515000	1516500
groupXIV	1601500	1604000
groupXIV	1675000	1676000
groupXIV	1695500	1703000
groupXIV	1729500	1731000
groupXIV	1818000	1821000
groupXIV	1881500	1883500
groupXIV	1885500	1891500
groupXIV	1990500	1991500
groupXIV	2406000	2409500
groupXIV	2646000	2650500
groupXIV	3039500	3043500
groupXIV	3230000	3234500

groupXIV	3350500	3352000
groupXIV	3458000	3467000
groupXIV	3669500	3670500
groupXIV	3695500	3696500
groupXIV	3783500	3785000
groupXIV	4096000	4099000
groupXIV	4189500	4191500
groupXIV	4494500	4497500
groupXIV	5020000	5021000
groupXIV	5203000	5204500
groupXIV	5210500	5212500
groupXIV	6392500	6396000
groupXIV	6497500	6501500
groupXIV	7097000	7112500
groupXIV	7302000	7304000
groupXIV	7841000	7928500
groupXIV	8195000	8197500
groupXIV	8621000	8622000
groupXIV	8779500	8783500
groupXIV	8915000	8927500
groupXIV	9103000	9109000
groupXIV	9142000	9143500
groupXIV	9560500	9561500
groupXIV	9774000	9775500
groupXIV	9893000	9894500
groupXIV	10553500	10557500
groupXIV	10710000	10712500
groupXIV	10841500	10845500
groupXIV	11536000	11537500
groupXIV	11572500	11574000
groupXIV	11575500	11579000
groupXIV	12142500	12144000
groupXIV	12228000	12229500
groupXIV	12236500	12243500

groupXIV	12389000	12391500
groupXIV	12584500	12586000
groupXIV	12611500	12613000
groupXIV	12658500	12660500
groupXIV	12692000	12695000
groupXIV	12765000	12768000
groupXIV	12973500	12976000
groupXIV	13130500	13138000
groupXIV	13143500	13145500
groupXIV	13165500	13173000
groupXIV	13204500	13206500
groupXIV	13363000	13364500
groupXIV	13378500	13389000
groupXIV	13619500	13622500
groupXIV	13649000	13650500
groupXIV	13673500	13676000
groupXIV	13677000	13680000
groupXIV	13686000	13687500
groupXIV	13705000	13707500
groupXIV	13744000	13745500
groupXIV	13829500	13830500
groupXIV	13983000	13984500
groupXIV	14207500	14208500
groupXIV	14213000	14214500
groupXIV	14268000	14272500
groupXIV	14456500	14458000
groupXIV	14594000	14597500
groupXIV	14943500	14954500
groupXIV	14984500	14986000
groupXIV	15039500	15040500
groupXIV	15058000	15068500
groupXIV	15186000	15188000
groupXIV	15197500	15199000
groupXIV	15215500	15217500

groupXV	68000	73500
groupXV	82000	87000
groupXV	114500	116500
groupXV	310500	311500
groupXV	323500	329000
groupXV	458500	461000
groupXV	566000	580000
groupXV	599500	616000
groupXV	817000	841000
groupXV	911500	912500
groupXV	1196500	1198000
groupXV	1258500	1259500
groupXV	1441500	1458500
groupXV	1523000	1539000
groupXV	1600000	1603000
groupXV	1612000	1616000
groupXV	1859500	1872500
groupXV	1904000	1909500
groupXV	1914500	1919500
groupXV	2205000	2207000
groupXV	2359000	2360000
groupXV	2625500	2632000
groupXV	2703500	2705000
groupXV	2752000	2753000
groupXV	2766000	2769000
groupXV	2989500	2991500
groupXV	2992500	3034500
groupXV	3096000	3103500
groupXV	3135000	3136500
groupXV	3149000	3153500
groupXV	3195000	3196500
groupXV	3209500	3219000
groupXV	4112000	4118500
groupXV	4359000	4370500

groupXV	4651500	4652500
groupXV	5020500	5021500
groupXV	5049500	5051500
groupXV	5257000	5263500
groupXV	5306500	5307500
groupXV	5349000	5357000
groupXV	5932500	5937000
groupXV	6052500	6060500
groupXV	6331000	6332500
groupXV	6341000	6343500
groupXV	6428000	6429500
groupXV	6683500	6692500
groupXV	6884000	6893000
groupXV	7184000	7188500
groupXV	7368000	7372000
groupXV	7667500	7668500
groupXV	7886500	7895500
groupXV	8064000	8072000
groupXV	8089000	8091500
groupXV	8093000	8094500
groupXV	8116500	8122000
groupXV	8393000	8394500
groupXV	8553500	8554500
groupXV	9129000	9130500
groupXV	9535000	9547000
groupXV	9563500	9564500
groupXV	9767000	9798000
groupXV	10337500	10339000
groupXV	10539000	10542000
groupXV	10620000	10621500
groupXV	10652500	10653500
groupXV	10853500	10854500
groupXV	10918000	10919000
groupXV	10940000	10942000

groupXV	10984500	10985500
groupXV	11502500	11503500
groupXV	11759500	11761000
groupXV	11764000	11765000
groupXV	11803000	11808000
groupXV	11817000	11819000
groupXV	11827000	11828000
groupXV	11964000	11968500
groupXV	12206500	12207500
groupXV	12359500	12360500
groupXV	12364500	12391000
groupXV	12550000	12554500
groupXV	12614000	12619000
groupXV	12652000	12653000
groupXV	12662500	12663500
groupXV	12684500	12685500
groupXV	12691000	12692000
groupXV	12719500	12724000
groupXV	12763500	12764500
groupXV	12824500	12831500
groupXV	12840500	12847500
groupXV	12865000	12866500
groupXV	12949500	12955500
groupXV	13009500	13011000
groupXV	13012000	13014000
groupXV	13461000	13462500
groupXV	13584000	13587500
groupXV	13591500	13596500
groupXV	13657500	13660000
groupXV	13680500	13683000
groupXV	13710000	13712500
groupXV	13725500	13726500
groupXV	13766000	13771500
groupXV	13782000	13786500

groupXV	13836500	13837500
groupXV	13918500	13919500
groupXV	13921000	13922500
groupXV	13942500	13944000
groupXV	13951500	13954500
groupXV	13992500	14004000
groupXV	14034000	14037500
groupXV	14060000	14062000
groupXV	14069500	14076000
groupXV	14212500	14221000
groupXV	14237500	14239500
groupXV	14255500	14257500
groupXV	14327500	14333000
groupXV	14335000	14338500
groupXV	14346500	14351500
groupXV	14424000	14426500
groupXV	14468500	14473500
groupXV	14575500	14576500
groupXV	14625000	14630500
groupXV	14711000	14714500
groupXV	14724000	14732500
groupXV	14775000	14776500
groupXV	14799500	14800500
groupXV	14805500	14811500
groupXV	14815500	14834500
groupXV	14847500	14849500
groupXV	14966000	14968500
groupXV	15078500	15079500
groupXV	15230500	15231500
groupXV	15232000	15235000
groupXV	15259500	15261000
groupXV	15344000	15345000
groupXV	15542000	15543500
groupXV	15546500	15548000

groupXV	15570000	15571500
groupXV	15631500	15635000
groupXV	15657000	15659500
groupXV	15689000	15690500
groupXV	15785000	15788500
groupXV	15796500	15799500
groupXV	15845000	15846500
groupXV	15960500	15961500
groupXV	16109500	16112000
groupXVI	17500	18500
groupXVI	37500	39500
groupXVI	69500	74500
groupXVI	343500	345500
groupXVI	347500	351000
groupXVI	354000	355000
groupXVI	377500	379000
groupXVI	480500	527500
groupXVI	573000	574500
groupXVI	919000	920500
groupXVI	972500	982500
groupXVI	994000	999500
groupXVI	1007000	1009500
groupXVI	1038000	1040000
groupXVI	1056500	1060500
groupXVI	1065000	1072000
groupXVI	1083000	1084500
groupXVI	1112000	1115000
groupXVI	1205500	1209000
groupXVI	1246000	1249500
groupXVI	1264000	1290000
groupXVI	1294500	1301500
groupXVI	1342000	1343000
groupXVI	1419000	1435000
groupXVI	1446500	1447500

groupXVI	1641000	1642000
groupXVI	1654500	1656500
groupXVI	1664000	1666000
groupXVI	1751000	1753500
groupXVI	1887000	1890500
groupXVI	1958500	1960500
groupXVI	1971500	1976000
groupXVI	1990500	1993500
groupXVI	1999500	2005000
groupXVI	2213500	2217000
groupXVI	2322000	2335500
groupXVI	2341500	2350000
groupXVI	2384500	2393000
groupXVI	2514000	2515500
groupXVI	2781500	2782500
groupXVI	2814000	2817000
groupXVI	2908000	2909000
groupXVI	2941000	2946500
groupXVI	3262000	3263500
groupXVI	3367500	3373000
groupXVI	3420500	3422500
groupXVI	3607500	3608500
groupXVI	3761000	3762000
groupXVI	3779500	3783000
groupXVI	3895000	3897500
groupXVI	3995500	3997000
groupXVI	4035500	4037000
groupXVI	4223000	4259000
groupXVI	4268500	4319500
groupXVI	4327500	4348500
groupXVI	4371000	4388000
groupXVI	4388500	4390000
groupXVI	4608500	4612000
groupXVI	4665500	4672000

groupXVI	4681000	4869000
groupXVI	4872500	4875500
groupXVI	4887000	4888500
groupXVI	5322500	5324500
groupXVI	5395500	5397000
groupXVI	5457000	5461000
groupXVI	5466500	5469000
groupXVI	5723500	5726500
groupXVI	5897500	5899500
groupXVI	6029500	6030500
groupXVI	6082500	6084500
groupXVI	6330000	6333500
groupXVI	6617500	6618500
groupXVI	6674000	6675500
groupXVI	6743000	6745000
groupXVI	6903500	6904500
groupXVI	6968000	6969000
groupXVI	7103500	7104500
groupXVI	7121500	7123500
groupXVI	7165500	7166500
groupXVI	7188000	7189000
groupXVI	7558500	7563500
groupXVI	7695000	7697500
groupXVI	7785000	7787000
groupXVI	8136500	8140000
groupXVI	8246500	8280500
groupXVI	8319000	8360000
groupXVI	8429500	8447500
groupXVI	8539000	8540000
groupXVI	8548500	8550500
groupXVI	8553000	8558500
groupXVI	8564000	8565500
groupXVI	8682500	8684000
groupXVI	8696500	8721000

groupXVI	9243000	9244500
groupXVI	9299500	9300500
groupXVI	9395500	9397000
groupXVI	9898000	9899000
groupXVI	10362000	10363000
groupXVI	10467000	10468000
groupXVI	11039000	11040000
groupXVI	11059000	11061500
groupXVI	11179000	11180500
groupXVI	11182500	11184000
groupXVI	11359000	11360500
groupXVI	11774000	11792500
groupXVI	11804000	11853000
groupXVI	11938000	11940000
groupXVI	11984000	11995500
groupXVI	12139000	12148000
groupXVI	12187500	12191000
groupXVI	12475500	12476500
groupXVI	12570500	12571500
groupXVI	12592000	12593000
groupXVI	12639000	12642500
groupXVI	13050500	13053500
groupXVI	13278500	13280500
groupXVI	13299500	13302500
groupXVI	13700000	13701000
groupXVI	13749000	13752500
groupXVI	13796000	13798000
groupXVI	13809000	13848000
groupXVI	13849000	13850000
groupXVI	13861000	13888500
groupXVI	13892500	13895000
groupXVI	13896000	13929000
groupXVI	13931000	13932000
groupXVI	13965500	13967000

groupXVI	14207000	14209000
groupXVI	14506500	14507500
groupXVI	14517500	14519500
groupXVI	14525000	14526000
groupXVI	14678500	14679500
groupXVI	14961000	14962500
groupXVI	15074500	15076500
groupXVI	15121500	15123000
groupXVI	15133500	15134500
groupXVI	15199000	15200500
groupXVI	15313500	15315500
groupXVI	15849500	15850500
groupXVI	15944500	15950000
groupXVI	16049500	16050500
groupXVI	16137000	16142500
groupXVI	16186500	16188500
groupXVI	16332000	16433500
groupXVI	16440000	16448500
groupXVI	16488500	16490500
groupXVI	16493500	16499000
groupXVI	16528500	16532000
groupXVI	16558000	16561500
groupXVI	16625500	16653000
groupXVI	16658000	16662500
groupXVI	16667000	16674000
groupXVI	16709000	16710000
groupXVI	16711000	16712000
groupXVI	16716500	16730000
groupXVI	16755000	16782500
groupXVI	16819000	16824000
groupXVI	16837000	16839500
groupXVI	16900000	16907500
groupXVI	16923500	16931000
groupXVI	16937500	16938500

groupXVI	16941500	16946500
groupXVI	16951500	16954500
groupXVI	16967500	17127000
groupXVI	17134000	17189500
groupXVI	17203000	17207000
groupXVI	17215500	17218500
groupXVI	17272500	17278000
groupXVI	17283500	17292500
groupXVI	17297000	17302000
groupXVI	17317500	17322500
groupXVI	17477500	17480000
groupXVI	17713500	17714500
groupXVI	17729500	17731000
groupXVI	17912500	17913500
groupXVII	72000	74000
groupXVII	76000	112000
groupXVII	158000	160500
groupXVII	212000	214500
groupXVII	231000	238500
groupXVII	267500	271000
groupXVII	272000	274000
groupXVII	355000	361000
groupXVII	366000	379000
groupXVII	436000	439500
groupXVII	564000	567500
groupXVII	579500	582500
groupXVII	608000	628000
groupXVII	690500	699500
groupXVII	795000	796000
groupXVII	1248000	1249000
groupXVII	1352000	1354500
groupXVII	1832000	1851000
groupXVII	1985500	1987000
groupXVII	2121000	2123000

groupXVII	2376000	2378000
groupXVII	2718500	2721000
groupXVII	3133000	3137000
groupXVII	3356000	3359000
groupXVII	3581500	3582500
groupXVII	3792000	3793000
groupXVII	4009000	4010000
groupXVII	4060000	4061000
groupXVII	4289500	4299500
groupXVII	5290500	5293500
groupXVII	5727000	5729500
groupXVII	5911500	5912500
groupXVII	5973500	5974500
groupXVII	6031000	6034000
groupXVII	6049500	6053500
groupXVII	6199000	6200000
groupXVII	6269500	6270500
groupXVII	6355500	6359500
groupXVII	6508500	6516500
groupXVII	6519000	6522000
groupXVII	6573000	6588000
groupXVII	7043500	7046000
groupXVII	7070000	7075500
groupXVII	7282000	7283500
groupXVII	7298000	7303500
groupXVII	7475000	7479500
groupXVII	7480000	7488500
groupXVII	7619000	7620000
groupXVII	7691500	7692500
groupXVII	8347000	8353000
groupXVII	8857000	8865000
groupXVII	8982500	8983500
groupXVII	9008000	9014000
groupXVII	9243000	9245000

groupXVII	9245500	9248500
groupXVII	9520000	9521000
groupXVII	9635500	9636500
groupXVII	9857000	9860000
groupXVII	10169500	10173000
groupXVII	10685000	10689000
groupXVII	10727000	10728500
groupXVII	10916500	10917500
groupXVII	10975000	10976500
groupXVII	11009500	11012000
groupXVII	11083500	11088000
groupXVII	11146500	11148000
groupXVII	11170500	11188000
groupXVII	11353500	11355000
groupXVII	11374500	11382000
groupXVII	11385500	11387000
groupXVII	11501500	11503000
groupXVII	11598000	11601000
groupXVII	11676500	11678000
groupXVII	11715000	11716500
groupXVII	11753500	11768000
groupXVII	11794500	11797000
groupXVII	12203500	12205500
groupXVII	12319000	12320000
groupXVII	12323000	12325500
groupXVII	12355000	12356500
groupXVII	12429000	12433500
groupXVII	12583000	12587500
groupXVII	12648000	12649000
groupXVII	12687500	12688500
groupXVII	12829500	12830500
groupXVII	12838000	12840000
groupXVII	13220000	13221000
groupXVII	13273000	13276500

groupXVII	13284000	13285500
groupXVII	13365500	13368000
groupXVII	13553500	13554500
groupXVII	13837500	13841500
groupXVII	13894500	13897500
groupXVII	13904000	13906000
groupXVII	13947000	13951500
groupXVII	13990500	13991500
groupXVII	14135000	14137000
groupXVII	14403500	14414000
groupXVIII	303000	304500
groupXVIII	356000	358000
groupXVIII	438000	440500
groupXVIII	445000	446500
groupXVIII	449500	450500
groupXVIII	593500	594500
groupXVIII	680500	683000
groupXVIII	685000	686500
groupXVIII	732000	735000
groupXVIII	801500	803500
groupXVIII	818500	831500
groupXVIII	960500	961500
groupXVIII	1006000	1007000
groupXVIII	1053500	1060500
groupXVIII	1069500	1074000
groupXVIII	1227500	1234500
groupXVIII	1237500	1241500
groupXVIII	1408000	1411000
groupXVIII	1412000	1413500
groupXVIII	1415000	1416500
groupXVIII	1597000	1598000
groupXVIII	1624000	1625500
groupXVIII	1690000	1692500
groupXVIII	1804500	1806500

groupXVIII	1904000	1907000
groupXVIII	2044500	2047500
groupXVIII	2580500	2581500
groupXVIII	2623000	2624000
groupXVIII	2831000	2833500
groupXVIII	2893000	2894000
groupXVIII	2962000	2967500
groupXVIII	2970500	2976000
groupXVIII	3006500	3009500
groupXVIII	3071000	3073000
groupXVIII	3173000	3179500
groupXVIII	3193000	3194000
groupXVIII	3253000	3254500
groupXVIII	3512500	3523000
groupXVIII	3534500	3544500
groupXVIII	3614500	3621000
groupXVIII	3751000	3752000
groupXVIII	3775000	3777500
groupXVIII	3813000	3818000
groupXVIII	4034000	4048000
groupXVIII	4167500	4171500
groupXVIII	4347500	4351000
groupXVIII	4405000	4419000
groupXVIII	4585000	4588000
groupXVIII	4634500	4636000
groupXVIII	4651000	4653500
groupXVIII	4988000	4989500
groupXVIII	5051000	5055000
groupXVIII	5056500	5057500
groupXVIII	5166000	5168500
groupXVIII	5239500	5242500
groupXVIII	5446000	5447500
groupXVIII	5627500	5629500
groupXVIII	5642000	5643000

groupXVIII	5657500	5660500
groupXVIII	5667000	5677000
groupXVIII	5851000	5852000
groupXVIII	5917000	5919000
groupXVIII	5944000	5957500
groupXVIII	5960500	5963500
groupXVIII	6203000	6208000
groupXVIII	6216500	6220500
groupXVIII	6226000	6231000
groupXVIII	6232500	6235000
groupXVIII	6238500	6240000
groupXVIII	6574000	6578500
groupXVIII	6840500	6844000
groupXVIII	6998500	7000500
groupXVIII	7029000	7030000
groupXVIII	7411500	7415500
groupXVIII	7549500	7551500
groupXVIII	7724000	7726000
groupXVIII	7831000	7832500
groupXVIII	8141500	8142500
groupXVIII	8184500	8188500
groupXVIII	8829000	8830000
groupXVIII	8935500	8939000
groupXVIII	9561000	9562500
groupXVIII	9799500	9800500
groupXVIII	9957500	9960500
groupXVIII	9984500	9990000
groupXVIII	10644000	10658500
groupXVIII	10817500	10819500
groupXVIII	10876000	10877000
groupXVIII	11055500	11058000
groupXVIII	11231000	11239500
groupXVIII	11242000	11245000
groupXVIII	11324000	11325500

groupXVIII	11487000	11489500
groupXVIII	11620000	11624000
groupXVIII	11896500	11897500
groupXVIII	11954000	11960000
groupXVIII	12041000	12050000
groupXVIII	12093500	12096000
groupXVIII	12227500	12229500
groupXVIII	12669500	12690500
groupXVIII	12762500	12763500
groupXVIII	12800000	12802500
groupXVIII	12811500	12813500
groupXVIII	12972500	12976000
groupXVIII	12988000	12991000
groupXVIII	12996500	13196500
groupXVIII	13207000	13208500
groupXVIII	13230500	13238000
groupXVIII	13258500	13260000
groupXVIII	13340500	13341500
groupXVIII	13440500	13443500
groupXVIII	13445500	13447000
groupXVIII	13566500	13568000
groupXVIII	13811500	13817000
groupXVIII	14002500	14005500
groupXVIII	14172000	14176500
groupXVIII	14631500	14632500
groupXVIII	14676500	14681500
groupXVIII	15030500	15032500
groupXVIII	15151500	15154000
groupXVIII	15213000	15215500
groupXVIII	15305000	15355000
groupXVIII	15395000	15398000
groupXVIII	15515500	15520500
groupXVIII	15616000	15618500
groupXVIII	15712500	15720500

groupXVIII	15726000	15728000
groupXVIII	15792000	15794000
groupXVIII	15817000	15834500
groupXVIII	15945500	15947000
groupXX	148000	158000
groupXX	204000	216500
groupXX	245500	304500
groupXX	371500	373000
groupXX	521500	526000
groupXX	591000	593000
groupXX	701500	717000
groupXX	723000	728000
groupXX	766500	777500
groupXX	851000	854500
groupXX	904000	906000
groupXX	917000	919500
groupXX	922000	923500
groupXX	1014000	1015000
groupXX	1295500	1296500
groupXX	1529000	1531000
groupXX	1664000	1665000
groupXX	1767500	1768500
groupXX	1898000	1899000
groupXX	2161500	2165000
groupXX	2175500	2177000
groupXX	2328500	2330000
groupXX	2361000	2377000
groupXX	2379000	2381500
groupXX	2476000	2478500
groupXX	2494000	2503000
groupXX	2520000	2529000
groupXX	2653000	2654500
groupXX	2687500	2695500
groupXX	2778500	2779500

groupXX	2800500	2801500
groupXX	2805500	2809000
groupXX	2906500	2908000
groupXX	3378000	3379000
groupXX	3605000	3608000
groupXX	3617000	3618500
groupXX	3756500	3759500
groupXX	3766000	3767500
groupXX	4464500	4466000
groupXX	4499500	4503500
groupXX	4678500	4680000
groupXX	4841000	4842500
groupXX	4927000	4928000
groupXX	5073000	5080500
groupXX	5261500	5262500
groupXX	5298000	5301500
groupXX	5430000	5431500
groupXX	5454500	5457000
groupXX	5504500	5506000
groupXX	5517500	5518500
groupXX	6093000	6094500
groupXX	6107000	6108000
groupXX	6312000	6315500
groupXX	6335000	6339500
groupXX	6501000	6502500
groupXX	7081500	7083500
groupXX	7358500	7359500
groupXX	7523000	7524000
groupXX	7528500	7531500
groupXX	7555000	7556000
groupXX	7597000	7601000
groupXX	7682000	7683000
groupXX	7848000	7850500
groupXX	7936500	7938000

groupXX	7960500	7961500
groupXX	8018000	8019500
groupXX	8257000	8260000
groupXX	8522500	8523500
groupXX	8660000	8677500
groupXX	9002000	9007500
groupXX	9686500	9691500
groupXX	10256500	10259000
groupXX	10319000	10321500
groupXX	10345500	10346500
groupXX	10388500	10389500
groupXX	10624500	10626500
groupXX	10999000	11000500
groupXX	12243500	12245000
groupXX	13005000	13019000
groupXX	13093500	13103000
groupXX	13173000	13175000
groupXX	13507000	13560500
groupXX	13776000	13782500
groupXX	13807000	13808000
groupXX	13909000	13954500
groupXX	14043500	14093000
groupXX	14097000	14140000
groupXX	14149000	14212000
groupXX	14230500	14234000
groupXX	14277000	14283500
groupXX	14331500	14332500
groupXX	14333000	14340500
groupXX	14342500	14346500
groupXX	14348000	14350500
groupXX	14420000	14425000
groupXX	14457500	14460000
groupXX	14729500	14730500
groupXX	14763500	14765000

groupXX	14778500	14782500
groupXX	14947500	14964000
groupXX	15164500	15165500
groupXX	15338000	15340000
groupXX	15532000	15533000
groupXX	15608000	15609500
groupXX	15890500	15892500
groupXX	16174500	16175500
groupXX	16225000	16227000
groupXX	16246000	16247000
groupXX	16259500	16262000
groupXX	16386000	16397000
groupXX	16404000	16407000
groupXX	16501500	16516000
groupXX	16547500	16549000
groupXX	16556500	16558000
groupXX	16752000	16768000
groupXX	16829500	16831500
groupXX	17188500	17201500
groupXX	17266500	17274500
groupXX	17310000	17311500
groupXX	17829500	17834000
groupXX	17887000	17890000
groupXX	17969000	17975500
groupXX	18122000	18125000
groupXX	18235500	18238500
groupXX	18316000	18320500
groupXX	18323000	18324000
groupXX	18331500	18341000
groupXX	18353000	18356500
groupXX	18357500	18358500
groupXX	18418500	18422000
groupXX	18424500	18425500
groupXX	18449000	18451000

groupXX	18501000	18505500
groupXX	18519000	18520000
groupXX	18738500	18743000
groupXX	18805500	18808500
groupXX	18842500	18851500
groupXX	18938000	18941000
groupXX	18976000	18981500
groupXX	19046500	19059000
groupXX	19093000	19099000
groupXX	19123000	19126000
groupXX	19134000	19135500
groupXX	19139000	19141000
groupXX	19144000	19145000
groupXX	19166000	19171000
groupXX	19224000	19225000
groupXX	19229000	19234000
groupXX	19248500	19249500
groupXX	19291000	19293500
groupXX	19307000	19309000
groupXX	19311500	19313500
groupXX	19487500	19490000
groupXX	19496000	19497000
groupXX	19547000	19559000
groupXX	19567000	19588000
groupXX	19590000	19591000
groupXX	19611500	19612500
groupXX	19659000	19662000
groupXX	19677000	19678000
groupXXI	0	280500
groupXXI	293500	298000
groupXXI	322500	328500
groupXXI	375000	377500
groupXXI	416500	419500
groupXXI	493500	495500

groupXXI	636000	637000
groupXXI	674500	676000
groupXXI	770000	771000
groupXXI	818500	821500
groupXXI	1246000	1247000
groupXXI	1273500	1275500
groupXXI	1289000	1290000
groupXXI	1410500	1411500
groupXXI	1592000	1593500
groupXXI	1688000	1689000
groupXXI	1959000	1960000
groupXXI	1981500	1985500
groupXXI	2104000	2121500
groupXXI	2756500	2759500
groupXXI	2857500	2858500
groupXXI	2866500	2867500
groupXXI	2877000	2879000
groupXXI	3035000	3037000
groupXXI	3100500	3267500
groupXXI	3279000	3639000
groupXXI	3641500	3763500
groupXXI	4422000	4423000
groupXXI	4524000	4537500
groupXXI	4892000	4894000
groupXXI	4950500	4952000
groupXXI	5327000	5329500
groupXXI	5445500	5450000
groupXXI	5461000	5468500
groupXXI	5513500	5524500
groupXXI	5665500	5667000
groupXXI	5913500	5925000
groupXXI	5975500	5980500
groupXXI	6009500	6011500
groupXXI	6107000	6115500

groupXXI	6920000	6931000
groupXXI	7685000	7686500
groupXXI	7941000	7957000
groupXXI	7959500	7990000
groupXXI	8110000	8113500
groupXXI	8525500	8528500
groupXXI	9408000	9418500
groupXXI	9493000	9496000
groupXXI	9799000	9800000
groupXXI	9923000	9924000
groupXXI	9988000	9989500
groupXXI	10063000	10064000
groupXXI	10283000	10284000
groupXXI	10557500	10560500
groupXXI	10641500	10664500
groupXXI	10718000	10719000
groupXXI	10832500	10835500
groupXXI	10853000	10865000
groupXXI	10889000	10892500
groupXXI	11081000	11083500
groupXXI	11398500	11494500
groupXXI	11601000	11604000
scaffold_101	23500	28000
scaffold_101	36000	37000
scaffold_101	59500	61500
scaffold_101	119500	122500
scaffold_101	197000	198000
scaffold_101	217500	230000
scaffold_101	263500	277000
scaffold_101	295000	296500
scaffold_1026	0	8000
scaffold_106	32000	35000
scaffold_106	36500	39000
scaffold_106	57500	60000

scaffold_106	132000	134000
scaffold_1067	0	7500
scaffold_108	11000	15500
scaffold_108	177000	178000
scaffold_108	196000	204500
scaffold_111	109500	112000
scaffold_111	248500	252000
scaffold_111	301500	304500
scaffold_111	312500	318500
scaffold_112	113000	115500
scaffold_115	16000	22500
scaffold_115	289500	294000
scaffold_119	43500	53000
scaffold_119	64000	68500
scaffold_119	233500	237000
scaffold_121	0	30000
scaffold_126	185000	192500
scaffold_126	254500	286000
scaffold_128	139500	142500
scaffold_129	10500	20000
scaffold_129	115500	117500
scaffold_130	135500	138000
scaffold_130	188500	191000
scaffold_131	51500	82000
scaffold_131	96500	122000
scaffold_137	0	111500
scaffold_139	177500	200000
scaffold_1405	0	6000
scaffold_146	83500	111500
scaffold_159	30500	78500
scaffold_160	21500	48500
scaffold_173	45000	95000
scaffold_178	0	30000
scaffold_180	103000	138000

scaffold_185	0	31000
scaffold_198	0	109000
scaffold_200	69000	95000
scaffold_201	0	143000
scaffold_224	16000	19500
scaffold_224	162500	208500
scaffold_225	17500	26500
scaffold_229	0	65500
scaffold_232	0	172500
scaffold_252	33000	51000
scaffold_253	29000	65500
scaffold_27	393000	396000
scaffold_27	721500	722500
scaffold_27	739000	741000
scaffold_27	843000	845000
scaffold_27	1066000	1067000
scaffold_27	1413000	1415500
scaffold_27	1613500	1617000
scaffold_27	1741500	1742500
scaffold_27	1904500	1908000
scaffold_27	1918000	1919000
scaffold_27	1969500	1972000
scaffold_27	2047000	2051000
scaffold_27	2207500	2210000
scaffold_27	2230000	2231500
scaffold_27	2307000	2309500
scaffold_27	2669500	2672500
scaffold_27	2694000	2709500
scaffold_27	2753500	2756500
scaffold_27	2903000	2905000
scaffold_27	3003000	3007000
scaffold_27	3092500	3094500
scaffold_27	3661500	3662500
scaffold_27	3850000	3852000

scaffold_27	3924000	3927500
scaffold_27	4006000	4007500
scaffold_27	4023500	4024500
scaffold_27	4041000	4042000
scaffold_27	4202000	4204500
scaffold_27	4326000	4341000
scaffold_27	4457000	4458000
scaffold_27	4524000	4525500
scaffold_27	4609000	4616500
scaffold_27	4619500	4623000
scaffold_27	4625000	4629500
scaffold_27	4790000	4795500
scaffold_27	4975500	4977500
scaffold_279	21500	22500
scaffold_281	5500	18500
scaffold_290	0	63500
scaffold_327	0	30500
scaffold_332	0	31500
scaffold_37	13500	59000
scaffold_37	61000	63500
scaffold_37	79500	80500
scaffold_37	91500	93000
scaffold_37	117500	118500
scaffold_37	123000	125500
scaffold_37	129500	132500
scaffold_37	133500	141500
scaffold_37	165000	171000
scaffold_37	188000	193500
scaffold_37	216000	217500
scaffold_37	244000	246000
scaffold_37	339000	340000
scaffold_37	348000	359500
scaffold_37	363500	364500
scaffold_37	368500	371000

scaffold_37	398000	401500
scaffold_37	414500	415500
scaffold_37	418500	421500
scaffold_37	457500	489500
scaffold_37	535500	573500
scaffold_37	586500	686000
scaffold_37	710000	712500
scaffold_37	717000	718500
scaffold_37	760000	762500
scaffold_37	815000	862000
scaffold_37	864000	868000
scaffold_37	914000	966500
scaffold_37	1116000	1120500
scaffold_37	1121500	1125000
scaffold_37	1134000	1137500
scaffold_37	1147500	1158000
scaffold_37	1212500	1213500
scaffold_37	1243500	1247000
scaffold_37	1253000	1254000
scaffold_37	1310500	1314000
scaffold_37	1447000	1463000
scaffold_37	1468000	1472000
scaffold_37	1500500	1511000
scaffold_37	1664500	1667500
scaffold_37	1858000	1931500
scaffold_37	1936500	1938500
scaffold_37	1990500	1992000
scaffold_37	2021000	2022000
scaffold_37	2040000	2044000
scaffold_37	2125000	2127000
scaffold_37	2149000	2151000
scaffold_37	2218500	2220000
scaffold_37	2362500	2364500
scaffold_37	2461500	2463500

scaffold_37	2586000	2590000
scaffold_380	4500	6000
scaffold_47	17000	22000
scaffold_47	43000	72500
scaffold_47	166000	178000
scaffold_47	189500	199000
scaffold_47	404500	410000
scaffold_47	428500	430500
scaffold_47	451500	481500
scaffold_47	488500	491500
scaffold_47	494000	499500
scaffold_47	500000	503000
scaffold_47	510500	512500
scaffold_47	522500	523500
scaffold_47	527500	529500
scaffold_47	597000	599000
scaffold_47	635500	655000
scaffold_47	700000	702500
scaffold_47	703500	716000
scaffold_47	737500	751500
scaffold_47	937500	939000
scaffold_47	950000	951500
scaffold_47	953500	966500
scaffold_47	986500	992500
scaffold_47	1009500	1011000
scaffold_47	1028500	1029500
scaffold_47	1108500	1111000
scaffold_47	1191000	1192000
scaffold_47	1290500	1294000
scaffold_47	1465000	1467000
scaffold_47	1488000	1492000
scaffold_47	1519000	1525000
scaffold_47	1595000	1600500
scaffold_47	1674500	1682000

scaffold_475	11000	12000
scaffold_48	348000	354500
scaffold_48	426000	427500
scaffold_48	638000	639500
scaffold_48	679000	681000
scaffold_48	921000	962000
scaffold_48	1459500	1461500
scaffold_48	1584500	1588000
scaffold_54	22000	60000
scaffold_54	116000	117500
scaffold_54	196500	226500
scaffold_54	242500	255500
scaffold_54	440000	441500
scaffold_54	467000	469500
scaffold_54	498500	538000
scaffold_54	550000	552000
scaffold_54	563000	566500
scaffold_54	628000	633500
scaffold_54	688000	732000
scaffold_54	770500	773000
scaffold_54	775000	777000
scaffold_54	1191500	1194500
scaffold_56	89000	92000
scaffold_56	133000	135000
scaffold_56	285500	286500
scaffold_56	291000	298000
scaffold_56	298500	360000
scaffold_56	430000	436000
scaffold_56	452500	454500
scaffold_56	569500	571500
scaffold_56	582500	584500
scaffold_56	600500	602000
scaffold_56	629500	630500
scaffold_56	639000	643000

scaffold_56	650000	659500
scaffold_56	675500	677000
scaffold_56	729500	734000
scaffold_56	773000	775500
scaffold_56	778000	779000
scaffold_56	798000	801500
scaffold_56	874500	878500
scaffold_56	950500	953000
scaffold_56	988000	991500
scaffold_56	1003000	1008500
scaffold_56	1035500	1036500
scaffold_56	1055000	1068000
scaffold_56	1076000	1169000
scaffold_578	0	14000
scaffold_58	213000	215000
scaffold_58	269500	272500
scaffold_58	307500	309000
scaffold_58	326000	328500
scaffold_58	466000	468500
scaffold_58	655000	659000
scaffold_590	5000	13500
scaffold_605	0	13500
scaffold_61	70500	76500
scaffold_61	112000	113500
scaffold_61	178500	186000
scaffold_61	329500	331500
scaffold_61	436500	439000
scaffold_61	652000	654000
scaffold_61	735500	739000
scaffold_61	764500	766000
scaffold_61	832500	834500
scaffold_61	894500	896000
scaffold_61	904000	928500
scaffold_67	43500	48500

scaffold_67	617500	620000
scaffold_67	713000	716000
scaffold_678	0	12000
scaffold_68	32000	83000
scaffold_68	176500	181000
scaffold_68	381500	401500
scaffold_68	584000	593500
scaffold_68	599000	604000
scaffold_68	717000	734500
scaffold_68	744000	811000
scaffold_69	65500	78000
scaffold_69	129000	130000
scaffold_69	156500	159500
scaffold_69	190500	191500
scaffold_69	432000	437000
scaffold_74	301500	303500
scaffold_74	370000	371000
scaffold_76	106500	109500
scaffold_76	122000	124500
scaffold_76	386500	398500
scaffold_76	439500	441500
scaffold_76	528000	529500
scaffold_80	66500	68500
scaffold_80	259000	263000
scaffold_80	302500	335500
scaffold_80	476000	489500
scaffold_84	68000	129500
scaffold_84	159500	161500
scaffold_84	164000	165000
scaffold_84	340000	341500
scaffold_84	354000	356000
scaffold_84	382000	384000
scaffold_84	387500	393500
scaffold_84	403500	404500

scaffold_84	415500	416500
scaffold_84	436000	437500
scaffold_84	460500	463500
scaffold_84	470000	472500
scaffold_84	503000	507500
scaffold_88	269000	278500
scaffold_88	345000	375500
scaffold_89	5500	12000
scaffold_89	26000	55000
scaffold_89	113000	131000
scaffold_89	179000	180000
scaffold_89	183000	184500
scaffold_89	247500	257500
scaffold_90	78500	110000
scaffold_90	178500	180500
scaffold_94	263500	271000
scaffold_95	66000	139500
scaffold_95	167000	170000
scaffold_99	372500	421000

b)

CNV Genes - Deletions					CNV Genes - Duplications				
Chromosome	Start	End	ID	Biotype	Chromosome	Start	End	ID	Biotype
groupI	295707	297034	ENSGACG00000004603	protein_coding	groupI	853150	864471	ENSGACG00000005067	protein_coding
groupI	313268	314198	ENSGACG00000004606	protein_coding	groupI	871939	888344	ENSGACG00000005083	protein_coding
groupI	372833	374001	ENSGACG00000004636	protein_coding	groupI	2995002	2999589	ENSGACG00000006282	protein_coding
groupI	1585297	1585992	ENSGACG00000005584	pseudogene	groupI	4256609	4274453	ENSGACG00000006908	protein_coding
groupI	1751567	1753282	ENSGACG00000005598	protein_coding	groupI	4261168	4274393	ENSGACG00000006915	protein_coding
groupI	2898938	2945757	ENSGACG00000006248	protein_coding	groupI	7056586	7057788	ENSGACG00000008332	protein_coding
groupI	2954357	2967016	ENSGACG00000006254	protein_coding	groupI	7058415	7060534	ENSGACG00000008339	protein_coding
groupI	3022737	3023274	ENSGACG00000006290	protein_coding	groupI	10068109	10069294	ENSGACG00000010096	protein_coding
groupI	10754992	10759116	ENSGACG00000010344	protein_coding	groupI	11429257	11431685	ENSGACG00000010778	protein_coding
groupI	12433155	12433563	ENSGACG00000011322	protein_coding	groupI	12433155	12433563	ENSGACG00000011322	protein_coding
groupI	13179251	13180187	ENSGACG00000011434	protein_coding	groupI	12436173	12437337	ENSGACG00000011325	protein_coding

groupI	15636450	15636945	ENSGACG00000012299	protein_coding	groupI	12452945	12454113	ENSGACG00000011326	protein_coding
groupI	16938590	16938754	ENSGACG00000021554	snRNA	groupI	13198663	13202494	ENSGACG00000011439	protein_coding
groupI	16939782	16939973	ENSGACG00000021608	snRNA	groupI	14805097	14807616	ENSGACG00000012151	protein_coding
groupI	17380379	17380543	ENSGACG00000022077	snRNA	groupI	16496807	16497485	ENSGACG00000012714	protein_coding
groupI	17381592	17381783	ENSGACG00000022118	snRNA	groupI	16498881	16499517	ENSGACG00000012717	protein_coding
groupI	17391751	17392549	ENSGACG00000013361	protein_coding	groupI	16804581	16810061	ENSGACG00000013023	protein_coding
groupI	17396655	17397508	ENSGACG00000013364	protein_coding	groupI	22159094	22160780	ENSGACG00000014493	protein_coding
groupI	17402687	17402851	ENSGACG00000021089	snRNA	groupI	23354931	23355747	ENSGACG00000014931	protein_coding
groupI	17406897	17407088	ENSGACG00000022136	snRNA	groupI	27575833	27583633	ENSGACG00000015498	protein_coding
groupI	17414337	17414501	ENSGACG00000021911	snRNA	groupI	27651833	27657602	ENSGACG00000015524	protein_coding
groupI	17415627	17415818	ENSGACG00000021752	snRNA	groupII	3632044	3665875	ENSGACG00000014524	protein_coding
groupI	19297007	19297526	ENSGACG00000013612	protein_coding	groupII	5274052	5289474	ENSGACG00000014858	protein_coding
groupI	20696730	20717429	ENSGACG00000013989	protein_coding	groupII	6500747	6501739	ENSGACG00000015250	protein_coding
groupI	21938318	21939140	ENSGACG00000014376	protein_coding	groupII	6502185	6507763	ENSGACG00000015251	protein_coding
groupI	22301106	22324840	ENSGACG00000014595	protein_coding	groupII	11736098	11741866	ENSGACG00000015897	protein_coding
groupI	22327880	22330473	ENSGACG00000014596	protein_coding	groupII	13383101	13386019	ENSGACG00000016064	protein_coding
groupI	22332778	22351627	ENSGACG00000014598	protein_coding	groupII	13387600	13389773	ENSGACG00000016067	protein_coding
groupI	26542836	26544555	ENSGACG00000015234	protein_coding	groupII	13414413	13420096	ENSGACG00000016077	protein_coding
groupI	26547457	26548597	ENSGACG00000015235	protein_coding	groupII	14617749	14621316	ENSGACG00000016317	protein_coding
groupI	27757277	27759800	ENSGACG00000015572	protein_coding	groupII	14622742	14624252	ENSGACG00000016325	protein_coding
groupI	27818572	27823703	ENSGACG00000015586	protein_coding	groupII	18349239	18352877	ENSGACG00000016770	protein_coding
groupII	570408	570951	ENSGACG00000014151	protein_coding	groupII	18514397	18516038	ENSGACG00000016853	protein_coding
groupII	576241	577205	ENSGACG00000014152	protein_coding	groupII	22611978	22619904	ENSGACG00000017544	protein_coding
groupII	582588	586680	ENSGACG00000014153	protein_coding	groupII	22624548	22641984	ENSGACG00000017547	protein_coding
groupII	1105861	1107275	ENSGACG00000014232	protein_coding	groupII	22642307	22645743	ENSGACG00000017552	protein_coding
groupII	3621489	3652936	ENSGACG00000014522	protein_coding	groupII	22645722	22648447	ENSGACG00000017562	protein_coding
groupII	3632044	3665875	ENSGACG00000014524	protein_coding	groupII	22651584	22661540	ENSGACG00000017570	protein_coding
groupII	6073193	6074507	ENSGACG00000015112	protein_coding	groupII	22662091	22672812	ENSGACG00000017572	protein_coding
groupII	6077774	6081378	ENSGACG00000015113	protein_coding	groupII	22689792	22705772	ENSGACG00000017581	protein_coding
groupII	6199287	6200592	ENSGACG00000015142	protein_coding	groupII	22706043	22707298	ENSGACG00000017588	protein_coding
groupII	10998750	10999885	ENSGACG00000015782	protein_coding	groupII	22718784	22722344	ENSGACG00000017589	protein_coding
groupII	14544920	14546712	ENSGACG00000016295	protein_coding	groupIII	703524	708437	ENSGACG00000013163	protein_coding
groupII	14551247	14569051	ENSGACG00000016298	protein_coding	groupIII	2158122	2160012	ENSGACG00000013849	protein_coding
groupII	14572400	14574583	ENSGACG00000016299	protein_coding	groupIII	2160097	2162816	ENSGACG00000013852	protein_coding

groupII	14622742	14624252	ENSGACG00000016325	protein_coding	groupIII	2838103	2840095	ENSGACG00000014075	protein_coding
groupII	14627366	14634689	ENSGACG00000016327	protein_coding	groupIII	2920904	2922961	ENSGACG00000014164	protein_coding
groupII	20790512	20790598	ENSGACG00000021287	miRNA	groupIII	5088853	5093804	ENSGACG00000014566	protein_coding
groupII	21510200	21511030	ENSGACG00000017259	protein_coding	groupIII	6657843	6658807	ENSGACG00000015012	protein_coding
groupII	21511444	21515490	ENSGACG00000017261	protein_coding	groupIII	6670461	6680264	ENSGACG00000015014	protein_coding
groupII	21518383	21521255	ENSGACG00000017264	protein_coding	groupIII	13791575	13805483	ENSGACG00000017397	protein_coding
groupIII	4995469	4996429	ENSGACG00000014534	protein_coding	groupIII	15030252	15030726	ENSGACG00000017650	protein_coding
groupIII	5907430	5907545	ENSGACG00000021673	snRNA	groupIII	15085595	15086060	ENSGACG00000017654	protein_coding
groupIII	5908387	5908501	ENSGACG00000021792	snRNA	groupIII	15790254	15791829	ENSGACG00000017710	protein_coding
groupIII	6476384	6476491	ENSGACG00000021945	snRNA	groupIII	15903924	15910984	ENSGACG00000017770	protein_coding
groupIII	7582032	7583389	ENSGACG00000015344	protein_coding	groupIII	15949085	15950812	ENSGACG00000017778	protein_coding
groupIII	11651903	11657219	ENSGACG00000016730	protein_coding	groupIII	16076417	16079556	ENSGACG00000017831	protein_coding
groupIII	15030252	15030726	ENSGACG00000017650	protein_coding	groupIII	16775134	16784646	ENSGACG00000018003	protein_coding
groupIII	15066504	15066969	ENSGACG00000017652	protein_coding	groupIV	1032371	1034255	ENSGACG00000016407	protein_coding
groupIII	15085595	15086060	ENSGACG00000017654	protein_coding	groupIV	1041544	1043491	ENSGACG00000016409	protein_coding
groupIII	15325298	15372609	ENSGACG00000017693	protein_coding	groupIV	1157978	1161567	ENSGACG00000016422	protein_coding
groupIII	15339420	15358805	ENSGACG00000017696	protein_coding	groupIV	1163192	1164964	ENSGACG00000016423	protein_coding
groupIII	15594828	15596532	ENSGACG00000017700	protein_coding	groupIV	2044919	2048363	ENSGACG00000016513	protein_coding
groupIII	15619760	15714945	ENSGACG00000017701	protein_coding	groupIV	5221494	5223424	ENSGACG00000016930	protein_coding
groupIII	15710748	15711655	ENSGACG00000017703	protein_coding	groupIV	5235526	5237184	ENSGACG00000016933	protein_coding
groupIII	16775134	16784646	ENSGACG00000018003	protein_coding	groupIV	9458591	9459397	ENSGACG00000017866	protein_coding
groupIV	1157978	1161567	ENSGACG00000016422	protein_coding	groupIV	9857056	9860968	ENSGACG00000017905	protein_coding
groupIV	2035884	2036643	ENSGACG00000016511	protein_coding	groupIV	10867466	10891677	ENSGACG00000018002	protein_coding
groupIV	2960511	2961148	ENSGACG00000016689	protein_coding	groupIV	12134598	12136513	ENSGACG00000018246	protein_coding
groupIV	2961343	2962246	ENSGACG00000016691	protein_coding	groupIV	12141624	12143011	ENSGACG00000018249	protein_coding
groupIV	7867265	7867362	ENSGACG00000022671	rRNA	groupIV	12158018	12160272	ENSGACG00000018250	protein_coding
groupIV	9946316	9949837	ENSGACG00000017913	protein_coding	groupIV	12161301	12162973	ENSGACG00000018251	protein_coding
groupIV	10011239	10014333	ENSGACG00000017914	protein_coding	groupIV	12180119	12183724	ENSGACG00000018253	protein_coding
groupIV	10714051	10716566	ENSGACG00000017995	protein_coding	groupIV	12438009	12441598	ENSGACG00000018279	protein_coding
groupIV	16793577	16794725	ENSGACG00000018755	protein_coding	groupIV	14254474	14256323	ENSGACG00000018464	protein_coding
groupIV	16797277	16798765	ENSGACG00000018756	protein_coding	groupIV	14258526	14259656	ENSGACG00000018466	protein_coding
groupIV	17428530	17429352	ENSGACG00000018760	protein_coding	groupIV	15735706	15737284	ENSGACG00000018572	protein_coding
groupIV	17674789	17678148	ENSGACG00000018769	protein_coding	groupIV	17650265	17655564	ENSGACG00000018765	protein_coding
groupIV	17695113	17703768	ENSGACG00000018770	protein_coding	groupIV	17674789	17678148	ENSGACG00000018769	protein_coding

groupIV	17968603	17984451	ENSGACG00000018816	protein_coding	groupIV	17695113	17703768	ENSGACG00000018770	protein_coding
groupIV	17974909	17977828	ENSGACG00000018817	protein_coding	groupIV	17968603	17984451	ENSGACG00000018816	protein_coding
groupIV	19844773	19845658	ENSGACG00000018957	protein_coding	groupIV	17974909	17977828	ENSGACG00000018817	protein_coding
groupIV	21059987	21065726	ENSGACG00000019053	protein_coding	groupIV	23078282	23085352	ENSGACG00000019238	protein_coding
groupIV	21110196	21118138	ENSGACG00000019056	protein_coding	groupIV	23817900	23826392	ENSGACG00000019291	protein_coding
groupIV	21134076	21142867	ENSGACG00000019061	protein_coding	groupIV	23828127	23830322	ENSGACG00000019292	protein_coding
groupIV	21160117	21174339	ENSGACG00000019063	protein_coding	groupIV	25512844	25515581	ENSGACG00000019505	protein_coding
groupIV	22128589	22135140	ENSGACG00000019173	protein_coding	groupIV	25553050	25563391	ENSGACG00000019508	protein_coding
groupIV	23095214	23095958	ENSGACG00000019239	protein_coding	groupIV	25585352	25601311	ENSGACG00000019509	protein_coding
groupIV	25474291	25479113	ENSGACG00000019490	protein_coding	groupIV	25675613	25676191	ENSGACG00000019514	pseudogene
groupIV	25482616	25490893	ENSGACG00000019494	protein_coding	groupIX	536210	537286	ENSGACG00000015947	protein_coding
groupIV	25494861	25499471	ENSGACG00000019495	protein_coding	groupIX	2327056	2339020	ENSGACG00000016293	protein_coding
groupIV	25585352	25601311	ENSGACG00000019509	protein_coding	groupIX	7914172	7917585	ENSGACG00000017439	protein_coding
groupIV	25707274	25717020	ENSGACG00000019515	protein_coding	groupIX	7917890	7920850	ENSGACG00000017443	protein_coding
groupIV	27137076	27137370	ENSGACG00000019614	protein_coding	groupIX	9738880	9747365	ENSGACG00000018037	protein_coding
groupIV	27227060	27229334	ENSGACG00000019615	protein_coding	groupIX	9749652	9756743	ENSGACG00000018047	protein_coding
groupIV	27588233	27596540	ENSGACG00000019623	protein_coding	groupIX	10193927	10196687	ENSGACG00000018165	protein_coding
groupIV	27589197	27590516	ENSGACG00000019624	protein_coding	groupIX	12707605	12709413	ENSGACG00000018504	protein_coding
groupIV	28143031	28143114	ENSGACG00000021377	miRNA	groupIX	12719447	12726901	ENSGACG00000018506	protein_coding
groupIV	28144001	28144078	ENSGACG00000021239	miRNA	groupIX	13041674	13042998	ENSGACG00000018566	protein_coding
groupIV	28144454	28144537	ENSGACG00000021356	miRNA	groupIX	13131694	13177741	ENSGACG00000018576	protein_coding
groupIV	28145175	28145252	ENSGACG00000021238	miRNA	groupIX	13755620	13759812	ENSGACG00000018666	protein_coding
groupIV	28145337	28145446	ENSGACG00000022217	miRNA	groupIX	13760494	13761558	ENSGACG00000018670	protein_coding
groupIV	28145640	28145721	ENSGACG00000021378	miRNA	groupIX	14200039	14201395	ENSGACG00000018738	protein_coding
groupIV	28146374	28146451	ENSGACG00000021237	miRNA	groupIX	14244729	14253607	ENSGACG00000018739	protein_coding
groupIV	28146826	28146909	ENSGACG00000021371	miRNA	groupIX	14766587	14767493	ENSGACG00000018877	protein_coding
groupIV	28147547	28147623	ENSGACG00000022148	miRNA	groupIX	14779973	14782136	ENSGACG00000018885	protein_coding
groupIV	28147901	28148010	ENSGACG00000022199	miRNA	groupIX	14782532	14786339	ENSGACG00000018886	protein_coding
groupIV	28148204	28148287	ENSGACG00000021370	miRNA	groupIX	14787170	14789734	ENSGACG00000018889	protein_coding
groupIV	28148923	28149000	ENSGACG00000021236	miRNA	groupIX	14837248	14840577	ENSGACG00000018899	protein_coding
groupIV	28149084	28149192	ENSGACG00000022265	miRNA	groupIX	14842577	14845125	ENSGACG00000018901	protein_coding
groupIV	28149380	28149463	ENSGACG00000021376	miRNA	groupIX	14866441	14870494	ENSGACG00000018906	protein_coding
groupIV	28150099	28150176	ENSGACG00000021235	miRNA	groupIX	15317094	15320614	ENSGACG00000019070	protein_coding
groupIV	28150551	28150634	ENSGACG00000021355	miRNA	groupIX	16751173	16753892	ENSGACG00000019356	protein_coding

groupIV	28151270	28151347	ENSGACG00000021234	miRNA	groupIX	16779202	16780079	ENSGACG00000019357	protein_coding
groupIV	28151431	28151549	ENSGACG00000021213	miRNA	groupIX	17624636	17627363	ENSGACG00000019454	protein_coding
groupIV	28151928	28152011	ENSGACG00000021369	miRNA	groupIX	18000473	18016329	ENSGACG00000019523	protein_coding
groupIV	28152647	28152724	ENSGACG00000021233	miRNA	groupIX	18613065	18613920	ENSGACG00000019632	protein_coding
groupIV	28152945	28153021	ENSGACG00000021453	miRNA	groupIX	18619888	18621460	ENSGACG00000019634	protein_coding
groupIV	28153818	28153895	ENSGACG00000021232	miRNA	groupIX	18627168	18628773	ENSGACG00000019635	protein_coding
groupIV	28154296	28154379	ENSGACG00000021368	miRNA	groupV	2349732	2354834	ENSGACG00000003113	protein_coding
groupIV	28155015	28155092	ENSGACG00000021231	miRNA	groupV	2407931	2411577	ENSGACG00000003122	protein_coding
groupIV	28155176	28155269	ENSGACG00000022142	miRNA	groupV	2433611	2439410	ENSGACG00000003139	protein_coding
groupIV	28155525	28155634	ENSGACG00000022185	miRNA	groupV	6433536	6436864	ENSGACG00000005315	protein_coding
groupIV	28156457	28156534	ENSGACG00000021230	miRNA	groupV	9159809	9160823	ENSGACG00000006864	protein_coding
groupIV	28156618	28156711	ENSGACG00000021215	miRNA	groupV	9740350	9744541	ENSGACG00000007782	protein_coding
groupIV	28156795	28156905	ENSGACG00000022184	miRNA	groupV	9747821	9756308	ENSGACG00000007789	protein_coding
groupIV	28157824	28157901	ENSGACG00000021229	miRNA	groupV	9752009	9753346	ENSGACG00000007794	protein_coding
groupIV	28157985	28158065	ENSGACG00000022303	miRNA	groupV	9897233	9898560	ENSGACG00000008003	protein_coding
groupIV	28163401	28163510	ENSGACG00000022225	miRNA	groupV	9901220	9902798	ENSGACG00000008009	protein_coding
groupIV	28163704	28163787	ENSGACG00000021367	miRNA	groupVI	1988466	1991312	ENSGACG00000002851	protein_coding
groupIV	28177107	28177190	ENSGACG00000021366	miRNA	groupVI	3639818	3643625	ENSGACG00000003705	protein_coding
groupIV	28177826	28177903	ENSGACG00000021228	miRNA	groupVI	5158325	5161804	ENSGACG00000004443	protein_coding
groupIV	28178278	28178361	ENSGACG00000021365	miRNA	groupVI	7596669	7598704	ENSGACG00000006186	protein_coding
groupIV	28179002	28179079	ENSGACG00000021227	miRNA	groupVI	7598935	7600642	ENSGACG00000006190	protein_coding
groupIV	28181263	28181346	ENSGACG00000021364	miRNA	groupVI	9254861	9257400	ENSGACG00000007928	protein_coding
groupIV	30617767	30618928	ENSGACG00000019829	protein_coding	groupVI	9257810	9259904	ENSGACG00000007939	protein_coding
groupIV	30626612	30627688	ENSGACG00000019830	protein_coding	groupVI	14576349	14579081	ENSGACG00000011333	protein_coding
groupIV	30644416	30645487	ENSGACG00000019831	protein_coding	groupVII	1863103	1864632	ENSGACG00000018970	protein_coding
groupIV	30659528	30660068	ENSGACG00000019832	protein_coding	groupVII	1865256	1865817	ENSGACG00000018971	protein_coding
groupIV	30667938	30669099	ENSGACG00000019833	protein_coding	groupVII	1870451	1873676	ENSGACG00000018972	protein_coding
groupIV	30672553	30678931	ENSGACG00000019835	protein_coding	groupVII	1946173	1947303	ENSGACG00000018977	protein_coding
groupIV	30691252	30693059	ENSGACG00000019836	protein_coding	groupVII	1948221	1952396	ENSGACG00000018978	protein_coding
groupIV	32442000	32452286	ENSGACG00000020087	protein_coding	groupVII	2209134	2211954	ENSGACG00000018984	protein_coding
groupIX	973755	1067563	ENSGACG00000016042	protein_coding	groupVII	2799482	2802249	ENSGACG00000019130	protein_coding
groupIX	1062960	1090304	ENSGACG00000016044	protein_coding	groupVII	2804572	2807645	ENSGACG00000019137	protein_coding
groupIX	1818327	1818411	ENSGACG00000022229	miRNA	groupVII	2976672	2980932	ENSGACG00000019201	protein_coding
groupIX	4859253	4872299	ENSGACG00000016687	protein_coding	groupVII	3092200	3094459	ENSGACG00000019261	protein_coding

groupIX	10461924	10467607	ENSGACG00000018208	protein_coding	groupVII	7740500	7740567	ENSGACG00000022330	snoRNA
groupIX	10617518	10620552	ENSGACG00000018226	protein_coding	groupVII	7741743	7741810	ENSGACG00000022420	snoRNA
groupIX	11993403	11993949	ENSGACG00000018412	protein_coding	groupVII	7742814	7742878	ENSGACG00000022678	snoRNA
groupIX	12698672	12700131	ENSGACG00000018501	protein_coding	groupVII	7743084	7743151	ENSGACG00000022377	snoRNA
groupIX	12786995	12790183	ENSGACG00000018518	protein_coding	groupVII	7743821	7743948	ENSGACG00000022735	snoRNA
groupIX	14623792	14644206	ENSGACG00000018842	protein_coding	groupVII	8160492	8164391	ENSGACG00000019920	protein_coding
groupIX	14652547	14675692	ENSGACG00000018848	protein_coding	groupVII	8166831	8167860	ENSGACG00000019921	protein_coding
groupIX	14762434	14763881	ENSGACG00000018874	protein_coding	groupVII	8628158	8630699	ENSGACG00000019933	protein_coding
groupIX	17101775	17107592	ENSGACG00000019395	protein_coding	groupVII	9216156	9217821	ENSGACG00000019974	protein_coding
groupIX	18356941	18358559	ENSGACG00000019581	protein_coding	groupVII	18548108	18551764	ENSGACG00000020434	protein_coding
groupIX	18363585	18365747	ENSGACG00000019582	protein_coding	groupVII	19682150	19684010	ENSGACG00000020529	protein_coding
groupIX	18541387	18543031	ENSGACG00000019627	protein_coding	groupVII	19690223	19691389	ENSGACG00000020530	protein_coding
groupIX	18556128	18557862	ENSGACG00000019628	protein_coding	groupVII	19694169	19695147	ENSGACG00000020531	protein_coding
groupIX	18576955	18578692	ENSGACG00000019631	protein_coding	groupVII	19699195	19700581	ENSGACG00000020532	protein_coding
groupIX	18613065	18613920	ENSGACG00000019632	protein_coding	groupVII	19703738	19704847	ENSGACG00000020533	protein_coding
groupIX	18619888	18621460	ENSGACG00000019634	protein_coding	groupVII	19715688	19717478	ENSGACG00000020534	protein_coding
groupIX	18627168	18628773	ENSGACG00000019635	protein_coding	groupVII	20737984	20740973	ENSGACG00000020587	protein_coding
groupIX	18643110	18644160	ENSGACG00000019636	protein_coding	groupVII	20741460	20747875	ENSGACG00000020588	protein_coding
groupIX	18754068	18756163	ENSGACG00000019670	protein_coding	groupVII	26976695	26981302	ENSGACG00000020857	protein_coding
groupIX	19379924	19380470	ENSGACG00000019773	protein_coding	groupVIII	1526334	1528158	ENSGACG00000003374	protein_coding
groupIX	19434008	19446092	ENSGACG00000019774	protein_coding	groupVIII	1528721	1530746	ENSGACG00000003379	protein_coding
groupIX	19435465	19454481	ENSGACG00000019775	protein_coding	groupVIII	1540682	1540880	ENSGACG00000003383	protein_coding
groupIX	19452539	19459616	ENSGACG00000019778	protein_coding	groupVIII	2401326	2401797	ENSGACG00000004278	protein_coding
groupIX	19470002	19473603	ENSGACG00000019779	protein_coding	groupVIII	3457300	3459158	ENSGACG00000004955	protein_coding
groupIX	19617731	19619991	ENSGACG00000019813	protein_coding	groupVIII	4797418	4801222	ENSGACG00000005415	protein_coding
groupV	298073	298954	ENSGACG00000002193	protein_coding	groupVIII	5827510	5844086	ENSGACG00000005908	protein_coding
groupV	2276176	2276290	ENSGACG00000021990	snRNA	groupVIII	5829401	5834466	ENSGACG00000005911	protein_coding
groupV	2282379	2283414	ENSGACG00000003104	protein_coding	groupVIII	6015396	6017317	ENSGACG00000006044	protein_coding
groupV	2293981	2294095	ENSGACG00000021591	snRNA	groupVIII	6015769	6016803	ENSGACG00000006048	protein_coding
groupV	2349732	2354834	ENSGACG00000003113	protein_coding	groupVIII	7382328	7385332	ENSGACG00000006820	protein_coding
groupV	2369989	2372000	ENSGACG00000003119	protein_coding	groupVIII	7386642	7387640	ENSGACG00000006825	protein_coding
groupV	2392355	2393317	ENSGACG00000003121	protein_coding	groupVIII	9799271	9799390	ENSGACG00000021753	rRNA
groupV	2407931	2411577	ENSGACG00000003122	protein_coding	groupVIII	9800110	9800229	ENSGACG00000021677	rRNA
groupV	2460111	2461901	ENSGACG00000003142	protein_coding	groupVIII	9800949	9801068	ENSGACG00000020985	rRNA

groupV	2818156	2820505	ENSGACG00000003300	protein_coding	groupVIII	9801793	9801912	ENSGACG00000021768	rRNA
groupV	2827982	2829662	ENSGACG00000003304	protein_coding	groupVIII	9802637	9802756	ENSGACG00000021730	rRNA
groupV	3780857	3782234	ENSGACG00000003536	protein_coding	groupVIII	9803476	9803595	ENSGACG00000022127	rRNA
groupV	3784132	3785207	ENSGACG00000003540	protein_coding	groupVIII	9804319	9804438	ENSGACG00000021670	rRNA
groupV	3787022	3788074	ENSGACG00000003542	protein_coding	groupVIII	9805160	9805279	ENSGACG00000021784	rRNA
groupV	5569081	5571634	ENSGACG00000004472	protein_coding	groupVIII	13018634	13018883	ENSGACG00000010319	protein_coding
groupV	6219244	6222646	ENSGACG00000005089	protein_coding	groupVIII	13877520	13879601	ENSGACG00000011122	protein_coding
groupV	9715933	9724579	ENSGACG00000007750	protein_coding	groupVIII	17428170	17433402	ENSGACG00000013513	protein_coding
groupV	9752009	9753346	ENSGACG00000007794	protein_coding	groupVIII	18848369	18849326	ENSGACG00000014343	protein_coding
groupV	9792576	9793056	ENSGACG00000007888	protein_coding	groupVIII	18852930	18860984	ENSGACG00000014346	protein_coding
groupV	10720553	10740273	ENSGACG00000008680	protein_coding	groupVIII	18977739	18979345	ENSGACG00000014402	protein_coding
groupV	10730135	10744608	ENSGACG00000008686	protein_coding	groupX	482614	492778	ENSGACG00000001913	protein_coding
groupVI	3919209	3920549	ENSGACG00000003856	protein_coding	groupX	520052	527028	ENSGACG00000001919	protein_coding
groupVI	10423422	10495268	ENSGACG00000008985	protein_coding	groupX	802045	806249	ENSGACG00000001937	protein_coding
groupVI	13967009	13980372	ENSGACG00000010952	protein_coding	groupX	1252131	1253742	ENSGACG00000001990	protein_coding
groupVI	14037234	14039561	ENSGACG00000010992	protein_coding	groupX	2022324	2030015	ENSGACG00000002382	protein_coding
groupVI	15480971	15482728	ENSGACG00000011609	protein_coding	groupX	7274594	7275863	ENSGACG00000004762	protein_coding
groupVII	100921	114439	ENSGACG00000018554	protein_coding	groupX	9151175	9152258	ENSGACG00000006431	protein_coding
groupVII	118493	120012	ENSGACG00000018555	protein_coding	groupX	9158879	9161494	ENSGACG00000006432	protein_coding
groupVII	148542	159171	ENSGACG00000018565	protein_coding	groupX	9162021	9163346	ENSGACG00000006436	protein_coding
groupVII	216241	217015	ENSGACG00000018593	protein_coding	groupX	11394451	11398811	ENSGACG00000007610	protein_coding
groupVII	218014	221452	ENSGACG00000018594	protein_coding	groupX	12695028	12695737	ENSGACG00000008252	protein_coding
groupVII	422136	423142	ENSGACG00000018645	protein_coding	groupX	12725355	12729289	ENSGACG00000008261	protein_coding
groupVII	423161	424282	ENSGACG00000018646	protein_coding	groupX	13199007	13201402	ENSGACG00000008826	protein_coding
groupVII	439558	441381	ENSGACG00000018648	protein_coding	groupX	13219610	13223472	ENSGACG00000008831	protein_coding
groupVII	443773	455609	ENSGACG00000018649	protein_coding	groupX	13226407	13227888	ENSGACG00000008840	protein_coding
groupVII	451812	460683	ENSGACG00000018651	protein_coding	groupX	13760959	13762237	ENSGACG00000009016	protein_coding
groupVII	518791	520048	ENSGACG00000018668	protein_coding	groupX	14670192	14673293	ENSGACG00000009542	protein_coding
groupVII	1064731	1078246	ENSGACG00000018781	protein_coding	groupX	14673760	14679040	ENSGACG00000009551	protein_coding
groupVII	1079750	1092181	ENSGACG00000018782	protein_coding	groupX	14747979	14771097	ENSGACG00000009653	protein_coding
groupVII	1430036	1432994	ENSGACG00000018845	protein_coding	groupX	14982273	14983437	ENSGACG00000009728	protein_coding
groupVII	1432523	1440418	ENSGACG00000018849	protein_coding	groupX	14983617	14987412	ENSGACG00000009731	protein_coding
groupVII	1437237	1440414	ENSGACG00000018850	protein_coding	groupXI	798846	806198	ENSGACG00000005131	protein_coding
groupVII	1561045	1568593	ENSGACG00000018903	protein_coding	groupXI	2407491	2415100	ENSGACG00000005871	protein_coding

groupVII	2280759	2299174	ENSGACG00000019019	protein_coding	groupXI	2519758	2521680	ENSGACG00000005975	protein_coding
groupVII	2335893	2336782	ENSGACG00000019028	protein_coding	groupXI	2527629	2528989	ENSGACG00000005990	protein_coding
groupVII	2338332	2342377	ENSGACG00000019029	protein_coding	groupXI	2857168	2860602	ENSGACG00000006401	protein_coding
groupVII	2444692	2447881	ENSGACG00000019051	protein_coding	groupXI	4310073	4313764	ENSGACG00000007445	protein_coding
groupVII	2449971	2451242	ENSGACG00000019052	protein_coding	groupXI	4320058	4324681	ENSGACG00000007447	protein_coding
groupVII	2454403	2462002	ENSGACG00000019055	protein_coding	groupXI	4378240	4381177	ENSGACG00000007449	protein_coding
groupVII	2995625	2998050	ENSGACG00000019205	protein_coding	groupXI	5217977	5219542	ENSGACG00000008371	protein_coding
groupVII	3010046	3032872	ENSGACG00000019210	protein_coding	groupXI	5224489	5227100	ENSGACG00000008375	protein_coding
groupVII	4062801	4064067	ENSGACG00000019324	protein_coding	groupXI	9203702	9204650	ENSGACG00000011117	protein_coding
groupVII	8995660	8997302	ENSGACG00000019953	protein_coding	groupXI	9650420	9654080	ENSGACG00000011265	protein_coding
groupVII	9049945	9052489	ENSGACG00000019964	protein_coding	groupXI	9675643	9679876	ENSGACG00000011268	protein_coding
groupVII	9056616	9058716	ENSGACG00000019965	protein_coding	groupXI	10229187	10233182	ENSGACG00000011425	protein_coding
groupVII	9065249	9066502	ENSGACG00000019966	protein_coding	groupXI	10238230	10239213	ENSGACG00000011428	protein_coding
groupVII	9088187	9089693	ENSGACG00000019968	protein_coding	groupXI	11030393	11032687	ENSGACG00000012078	protein_coding
groupVII	9109075	9111092	ENSGACG00000019969	protein_coding	groupXI	11537509	11539417	ENSGACG00000012329	protein_coding
groupVII	9154612	9155550	ENSGACG00000019971	protein_coding	groupXI	11545159	11547783	ENSGACG00000012332	protein_coding
groupVII	9216156	9217821	ENSGACG00000019974	protein_coding	groupXI	11553143	11558068	ENSGACG00000012338	protein_coding
groupVII	9221363	9226588	ENSGACG00000019976	protein_coding	groupXI	11557480	11558408	ENSGACG00000012343	protein_coding
groupVII	9728032	9728572	ENSGACG00000020029	protein_coding	groupXI	11562910	11565184	ENSGACG00000012344	protein_coding
groupVII	10225719	10227058	ENSGACG00000020061	protein_coding	groupXI	11566383	11567956	ENSGACG00000012347	protein_coding
groupVII	10240904	10243995	ENSGACG00000020062	protein_coding	groupXI	11802758	11803047	ENSGACG00000012554	protein_coding
groupVII	10287136	10293980	ENSGACG00000020063	protein_coding	groupXI	11812938	11820450	ENSGACG00000012556	protein_coding
groupVII	19028240	19029560	ENSGACG00000020469	protein_coding	groupXI	11826884	11830465	ENSGACG00000012558	protein_coding
groupVII	19667434	19667827	ENSGACG00000020527	protein_coding	groupXI	11838444	11838984	ENSGACG00000012564	protein_coding
groupVII	19682150	19684010	ENSGACG00000020529	protein_coding	groupXI	12320343	12321455	ENSGACG00000012766	protein_coding
groupVII	19690223	19691389	ENSGACG00000020530	protein_coding	groupXI	12326299	12327385	ENSGACG00000012767	protein_coding
groupVII	19715688	19717478	ENSGACG00000020534	protein_coding	groupXI	12330952	12351249	ENSGACG00000012769	protein_coding
groupVII	19744061	19745030	ENSGACG00000020538	protein_coding	groupXI	12357662	12360295	ENSGACG00000012776	protein_coding
groupVII	19754586	19755930	ENSGACG00000020539	protein_coding	groupXI	12365393	12366157	ENSGACG00000012779	protein_coding
groupVII	19762881	19764164	ENSGACG00000020540	protein_coding	groupXI	12369682	12371008	ENSGACG00000012781	protein_coding
groupVII	19775837	19776803	ENSGACG00000020541	protein_coding	groupXI	12374730	12380140	ENSGACG00000012783	protein_coding
groupVII	19778018	19780244	ENSGACG00000020542	protein_coding	groupXI	12398198	12399317	ENSGACG00000012790	protein_coding
groupVII	19780146	19784025	ENSGACG00000020543	protein_coding	groupXI	12405271	12405919	ENSGACG00000012792	protein_coding
groupVII	21446433	21456144	ENSGACG00000020614	protein_coding	groupXI	12426250	12426681	ENSGACG00000012794	protein_coding

groupVII	21458626	21466587	ENSGACG00000020615	protein_coding	groupXI	12434699	12435788	ENSGACG00000012797	protein_coding
groupVII	27670416	27672409	ENSGACG00000020893	protein_coding	groupXI	12439443	12461313	ENSGACG00000012799	protein_coding
groupVIII	4793464	4794694	ENSGACG00000005411	protein_coding	groupXI	12683666	12685586	ENSGACG00000013046	protein_coding
groupVIII	4797418	4801222	ENSGACG00000005415	protein_coding	groupXI	12688018	12689938	ENSGACG00000013048	protein_coding
groupVIII	4837405	4839287	ENSGACG00000005418	protein_coding	groupXI	12692690	12694610	ENSGACG00000013051	protein_coding
groupVIII	4846148	4848030	ENSGACG00000005420	protein_coding	groupXI	12694207	12699627	ENSGACG00000013052	protein_coding
groupVIII	9656461	9658641	ENSGACG00000008465	protein_coding	groupXI	12697366	12699286	ENSGACG00000013055	protein_coding
groupVIII	9816517	9816626	ENSGACG00000022597	rRNA	groupXI	14969186	14972275	ENSGACG00000014382	protein_coding
groupVIII	16520407	16526013	ENSGACG00000012935	protein_coding	groupXI	15607307	15613431	ENSGACG00000014553	protein_coding
groupVIII	16527729	16528907	ENSGACG00000012948	protein_coding	groupXI	15608501	15610308	ENSGACG00000014556	protein_coding
groupVIII	17193570	17195072	ENSGACG00000013266	protein_coding	groupXI	15616018	15617314	ENSGACG00000014557	protein_coding
groupVIII	17201147	17204765	ENSGACG00000013270	protein_coding	groupXII	7855521	7860176	ENSGACG00000006426	protein_coding
groupVIII	17206368	17210137	ENSGACG00000013272	protein_coding	groupXII	8050561	8054456	ENSGACG00000006517	protein_coding
groupVIII	18879638	18882129	ENSGACG00000014359	protein_coding	groupXII	8061270	8065049	ENSGACG00000006525	protein_coding
groupX	425778	432004	ENSGACG00000001910	protein_coding	groupXII	8067347	8071918	ENSGACG00000006529	protein_coding
groupX	482614	492778	ENSGACG00000001913	protein_coding	groupXII	8078414	8082300	ENSGACG00000006534	protein_coding
groupX	520052	527028	ENSGACG00000001919	protein_coding	groupXII	8093401	8100588	ENSGACG00000006536	protein_coding
groupX	572040	573204	ENSGACG00000001921	protein_coding	groupXII	8592346	8596394	ENSGACG00000006762	protein_coding
groupX	574900	576510	ENSGACG00000001922	protein_coding	groupXII	10099343	10100498	ENSGACG00000007860	protein_coding
groupX	577886	579878	ENSGACG00000001923	protein_coding	groupXII	10319744	10319829	ENSGACG00000022151	miRNA
groupX	581240	583157	ENSGACG00000001928	protein_coding	groupXII	10786665	10789131	ENSGACG00000008662	protein_coding
groupX	662964	665020	ENSGACG00000001932	protein_coding	groupXII	13373225	13376662	ENSGACG00000010505	protein_coding
groupX	779137	791924	ENSGACG00000001935	protein_coding	groupXII	13483911	13486062	ENSGACG00000010523	protein_coding
groupX	802045	806249	ENSGACG00000001937	protein_coding	groupXII	14228523	14229902	ENSGACG00000011017	protein_coding
groupX	891778	894040	ENSGACG00000001941	protein_coding	groupXII	18066533	18067182	ENSGACG00000013164	protein_coding
groupX	919578	921019	ENSGACG00000001944	protein_coding	groupXIII	2228514	2231244	ENSGACG00000004719	protein_coding
groupX	932539	936469	ENSGACG00000001947	protein_coding	groupXIII	3166005	3166618	ENSGACG00000005317	protein_coding
groupX	1054391	1055656	ENSGACG00000001971	protein_coding	groupXIII	3168193	3174770	ENSGACG00000005319	protein_coding
groupX	1057579	1102657	ENSGACG00000001973	protein_coding	groupXIII	3257765	3259517	ENSGACG00000005338	protein_coding
groupX	1092708	1093873	ENSGACG00000001976	protein_coding	groupXIII	4337517	4338066	ENSGACG00000005923	protein_coding
groupX	1119795	1124476	ENSGACG00000001978	protein_coding	groupXIII	12130143	12132899	ENSGACG00000011009	protein_coding
groupX	1148456	1148636	ENSGACG00000001979	protein_coding	groupXIII	12337389	12342763	ENSGACG00000011140	protein_coding
groupX	1995485	1996462	ENSGACG00000002361	protein_coding	groupXIII	12343503	12344566	ENSGACG00000011154	protein_coding
groupX	5760062	5761742	ENSGACG00000003969	protein_coding	groupXIII	13133177	13134538	ENSGACG00000011581	protein_coding

groupX	6446800	6474821	ENSGACG00000004168	protein_coding	groupXIII	13135352	13138731	ENSGACG00000011583	protein_coding
groupX	7028295	7028853	ENSGACG00000004547	protein_coding	groupXIII	14423966	14427196	ENSGACG00000012376	protein_coding
groupX	7274594	7275863	ENSGACG00000004762	protein_coding	groupXIII	14723884	14725858	ENSGACG00000012624	protein_coding
groupX	7282382	7295753	ENSGACG00000004765	protein_coding	groupXIII	16088590	16091699	ENSGACG00000013195	protein_coding
groupX	8844844	8846238	ENSGACG00000006123	protein_coding	groupXIII	16093220	16101732	ENSGACG00000013215	protein_coding
groupX	9151175	9152258	ENSGACG00000006431	protein_coding	groupXIII	16775252	16782436	ENSGACG00000013659	protein_coding
groupX	9158879	9161494	ENSGACG00000006432	protein_coding	groupXIII	17338063	17340704	ENSGACG00000013806	protein_coding
groupX	9162021	9163346	ENSGACG00000006436	protein_coding	groupXIII	17437663	17442146	ENSGACG00000013898	protein_coding
groupX	9236995	9237481	ENSGACG00000006508	protein_coding	groupXIII	18498299	18499267	ENSGACG00000014279	protein_coding
groupX	12654371	12658742	ENSGACG00000008242	protein_coding	groupXIII	18573306	18573822	ENSGACG00000014283	protein_coding
groupX	12683621	12684212	ENSGACG00000008250	protein_coding	groupXIII	18582294	18583087	ENSGACG00000014284	protein_coding
groupX	12695028	12695737	ENSGACG00000008252	protein_coding	groupXIII	19570023	19572036	ENSGACG00000014655	protein_coding
groupX	12701437	12748131	ENSGACG00000008253	protein_coding	groupXIV	72687	77968	ENSGACG00000015449	protein_coding
groupX	12725355	12729289	ENSGACG00000008261	protein_coding	groupXIV	389938	392477	ENSGACG00000015598	protein_coding
groupX	12743172	12744173	ENSGACG00000008264	protein_coding	groupXIV	6573596	6576506	ENSGACG00000016973	protein_coding
groupX	12748845	12752179	ENSGACG00000008265	protein_coding	groupXIV	6576638	6580174	ENSGACG00000016979	protein_coding
groupX	13199007	13201402	ENSGACG00000008826	protein_coding	groupXIV	6579515	6581319	ENSGACG00000016981	protein_coding
groupX	13219610	13223472	ENSGACG00000008831	protein_coding	groupXIV	6953570	6959101	ENSGACG00000017192	protein_coding
groupX	13226407	13227888	ENSGACG00000008840	protein_coding	groupXIV	7945519	7945607	ENSGACG00000022320	miRNA
groupX	13227912	13229965	ENSGACG00000008842	protein_coding	groupXIV	7945971	7950502	ENSGACG00000017484	protein_coding
groupX	14265898	14266765	ENSGACG00000009270	protein_coding	groupXIV	11265127	11270449	ENSGACG00000018106	protein_coding
groupX	14346525	14347461	ENSGACG00000009290	protein_coding	groupXIV	14959641	14966423	ENSGACG00000018641	protein_coding
groupX	14349434	14350596	ENSGACG00000009293	protein_coding	groupXV	707992	710643	ENSGACG00000004587	protein_coding
groupX	14351449	14351758	ENSGACG00000009296	protein_coding	groupXV	1524238	1525287	ENSGACG00000005289	protein_coding
groupX	14353529	14354069	ENSGACG00000009298	protein_coding	groupXV	2255583	2256584	ENSGACG00000005976	protein_coding
groupX	14355694	14356838	ENSGACG00000009300	protein_coding	groupXV	2257687	2260308	ENSGACG00000005979	protein_coding
groupX	14362209	14363366	ENSGACG00000009301	protein_coding	groupXV	2611975	2613553	ENSGACG00000006178	protein_coding
groupX	14364220	14364599	ENSGACG00000009304	protein_coding	groupXV	2614631	2617148	ENSGACG00000006181	protein_coding
groupX	14365694	14366909	ENSGACG00000009308	protein_coding	groupXV	2978714	2979601	ENSGACG00000006710	protein_coding
groupX	14373778	14374312	ENSGACG00000009312	protein_coding	groupXV	2986777	2987060	ENSGACG00000006711	protein_coding
groupX	14377101	14378262	ENSGACG00000009313	protein_coding	groupXV	7895096	7896633	ENSGACG00000009674	protein_coding
groupX	14384484	14385652	ENSGACG00000009315	protein_coding	groupXV	8490039	8494026	ENSGACG00000010083	protein_coding
groupX	14391107	14392270	ENSGACG00000009316	protein_coding	groupXV	11874054	11875197	ENSGACG00000012024	protein_coding
groupX	14393126	14395820	ENSGACG00000009320	protein_coding	groupXVI	45770	57172	ENSGACG00000001424	protein_coding

groupX	14400506	14401518	ENSGACG00000009322	protein_coding	groupXVI	83745	92475	ENSGACG00000001427	protein_coding
groupX	14402385	14402758	ENSGACG00000009324	protein_coding	groupXVI	88088	88568	ENSGACG00000001430	protein_coding
groupX	14405020	14405560	ENSGACG00000009325	protein_coding	groupXVI	97358	105500	ENSGACG00000001431	protein_coding
groupX	14407419	14408582	ENSGACG00000009327	protein_coding	groupXVI	479032	480249	ENSGACG00000001533	protein_coding
groupX	14409438	14409741	ENSGACG00000009329	protein_coding	groupXVI	495658	497657	ENSGACG00000001535	protein_coding
groupX	14411305	14412039	ENSGACG00000009331	protein_coding	groupXVI	3202294	3204354	ENSGACG00000001972	protein_coding
groupX	14562436	14563592	ENSGACG00000009470	protein_coding	groupXVI	4216513	4218833	ENSGACG00000002200	protein_coding
groupX	14569171	14569474	ENSGACG00000009472	protein_coding	groupXVI	4667417	4667523	ENSGACG00000021158	snRNA
groupX	14570331	14571493	ENSGACG00000009475	protein_coding	groupXVI	4679751	4681582	ENSGACG00000002238	protein_coding
groupX	14574132	14575341	ENSGACG00000009476	protein_coding	groupXVI	4681909	4682015	ENSGACG00000021180	snRNA
groupX	14577679	14578841	ENSGACG00000009478	protein_coding	groupXVI	4690277	4690383	ENSGACG00000022013	snRNA
groupX	14584009	14585171	ENSGACG00000009480	protein_coding	groupXVI	4703391	4713584	ENSGACG00000002240	protein_coding
groupX	14589135	14589669	ENSGACG00000009484	protein_coding	groupXVI	4733213	4733729	ENSGACG00000002251	protein_coding
groupX	14599366	14599684	ENSGACG00000009486	protein_coding	groupXVI	4753831	4754968	ENSGACG00000002252	protein_coding
groupX	14603581	14604743	ENSGACG00000009492	protein_coding	groupXVI	4759463	4761705	ENSGACG00000002254	protein_coding
groupX	14610124	14611286	ENSGACG00000009493	protein_coding	groupXVI	4784786	4787328	ENSGACG00000002255	protein_coding
groupX	14615251	14616001	ENSGACG00000009495	protein_coding	groupXVI	8173993	8178235	ENSGACG00000003624	protein_coding
groupX	14618821	14619983	ENSGACG00000009497	protein_coding	groupXVI	8398715	8399867	ENSGACG00000003669	protein_coding
groupX	14621895	14623873	ENSGACG00000009499	protein_coding	groupXVI	13801659	13803622	ENSGACG00000006943	protein_coding
groupX	14624729	14625885	ENSGACG00000009503	protein_coding	groupXVI	13808922	13810457	ENSGACG00000006947	protein_coding
groupX	14629885	14630158	ENSGACG00000009505	protein_coding	groupXVI	13855607	13860804	ENSGACG00000006963	protein_coding
groupX	14633407	14634564	ENSGACG00000009506	protein_coding	groupXVI	13901515	13901815	ENSGACG00000006974	protein_coding
groupX	14636416	14637366	ENSGACG00000009508	protein_coding	groupXVI	15500598	15501588	ENSGACG00000007859	protein_coding
groupX	14639968	14641130	ENSGACG00000009513	protein_coding	groupXVI	15511870	15512836	ENSGACG00000007864	protein_coding
groupX	14646571	14647731	ENSGACG00000009514	protein_coding	groupXVI	15514631	15515772	ENSGACG00000007868	protein_coding
groupX	14652734	14653476	ENSGACG00000009516	protein_coding	groupXVI	15950421	15951745	ENSGACG00000008139	protein_coding
groupX	14656314	14657588	ENSGACG00000009519	protein_coding	groupXVI	16368348	16369778	ENSGACG00000008301	protein_coding
groupX	14658687	14660689	ENSGACG00000009521	protein_coding	groupXVI	16460420	16461727	ENSGACG00000008305	protein_coding
groupX	15250779	15253079	ENSGACG00000009880	protein_coding	groupXVI	16467897	16469661	ENSGACG00000008316	protein_coding
groupX	15255685	15258047	ENSGACG00000009883	protein_coding	groupXVI	16483479	16485039	ENSGACG00000008324	protein_coding
groupX	15527867	15528362	ENSGACG00000010381	protein_coding	groupXVI	16908538	16919404	ENSGACG00000008521	protein_coding
groupXI	1524173	1526036	ENSGACG00000005617	protein_coding	groupXVI	16932800	16935689	ENSGACG00000008534	protein_coding
groupXI	2515095	2516105	ENSGACG00000005970	protein_coding	groupXVI	16946300	16948367	ENSGACG00000008537	protein_coding
groupXI	4310073	4313764	ENSGACG00000007445	protein_coding	groupXVI	16966116	16967188	ENSGACG00000008540	protein_coding

groupXI	4320058	4324681	ENSGACG00000007447	protein_coding	groupXVI	17028080	17029718	ENSGACG00000008551	protein_coding
groupXI	4378240	4381177	ENSGACG00000007449	protein_coding	groupXVI	17065775	17066988	ENSGACG00000008556	protein_coding
groupXI	4446709	4449166	ENSGACG00000007451	protein_coding	groupXVI	17124130	17125629	ENSGACG00000008557	protein_coding
groupXI	4496074	4496580	ENSGACG00000007452	pseudogene	groupXVI	17151884	17152878	ENSGACG00000008561	protein_coding
groupXI	4500951	4505292	ENSGACG00000007454	protein_coding	groupXVII	1834691	1835293	ENSGACG00000004312	protein_coding
groupXI	4554291	4558645	ENSGACG00000007457	protein_coding	groupXVII	1839282	1840721	ENSGACG00000004316	protein_coding
groupXI	4560382	4565290	ENSGACG00000007477	protein_coding	groupXVII	1843066	1843668	ENSGACG00000004322	protein_coding
groupXI	4571989	4573330	ENSGACG00000007511	protein_coding	groupXVII	1847556	1848158	ENSGACG00000004326	protein_coding
groupXI	5037460	5048043	ENSGACG00000008059	protein_coding	groupXVII	1850494	1851932	ENSGACG00000004327	protein_coding
groupXI	5217977	5219542	ENSGACG00000008371	protein_coding	groupXVII	1860472	1862270	ENSGACG00000004330	protein_coding
groupXI	5224489	5227100	ENSGACG00000008375	protein_coding	groupXVII	3479040	3479668	ENSGACG00000005234	protein_coding
groupXI	6475197	6478735	ENSGACG00000009259	protein_coding	groupXVII	8436150	8436272	ENSGACG00000022734	snoRNA
groupXI	8742110	8743885	ENSGACG00000010883	protein_coding	groupXVII	8437595	8437663	ENSGACG00000022710	snoRNA
groupXI	8767427	8769201	ENSGACG00000010888	protein_coding	groupXVII	8437872	8438002	ENSGACG00000022610	snoRNA
groupXI	8780064	8781863	ENSGACG00000010893	protein_coding	groupXVII	8584723	8615636	ENSGACG00000008967	protein_coding
groupXI	8822909	8826277	ENSGACG00000010896	protein_coding	groupXVII	8810501	8815093	ENSGACG00000009188	protein_coding
groupXI	8890779	8894287	ENSGACG00000010948	protein_coding	groupXVII	11175540	11184052	ENSGACG00000010910	protein_coding
groupXI	9650420	9654080	ENSGACG00000011265	protein_coding	groupXVIII	791583	793140	ENSGACG00000004270	protein_coding
groupXI	9721383	9723273	ENSGACG00000011274	protein_coding	groupXVIII	806191	807588	ENSGACG00000004274	protein_coding
groupXI	9794034	9795077	ENSGACG00000011276	protein_coding	groupXVIII	10940767	10947132	ENSGACG00000010905	protein_coding
groupXI	10220642	10222287	ENSGACG00000011417	protein_coding	groupXVIII	10947497	10948754	ENSGACG00000010912	protein_coding
groupXI	10238230	10239213	ENSGACG00000011428	protein_coding	groupXVIII	10951524	10954300	ENSGACG00000010916	protein_coding
groupXI	11545159	11547783	ENSGACG00000012332	protein_coding	groupXVIII	10952042	10952119	ENSGACG00000022615	snoRNA
groupXI	11802758	11803047	ENSGACG00000012554	protein_coding	groupXVIII	11182518	11182613	ENSGACG00000022492	miRNA
groupXI	11812938	11820450	ENSGACG00000012556	protein_coding	groupXVIII	13457003	13458614	ENSGACG00000012288	protein_coding
groupXI	11826884	11830465	ENSGACG00000012558	protein_coding	groupXX	730484	736155	ENSGACG00000003427	protein_coding
groupXI	12065872	12068920	ENSGACG00000012609	protein_coding	groupXX	1929256	1940454	ENSGACG00000003791	protein_coding
groupXI	12085946	12086903	ENSGACG00000012631	protein_coding	groupXX	2091661	2105365	ENSGACG00000003971	protein_coding
groupXI	12307268	12317710	ENSGACG00000012763	protein_coding	groupXX	3934001	3934520	ENSGACG00000005417	protein_coding
groupXI	12320343	12321455	ENSGACG00000012766	protein_coding	groupXX	3937142	3937661	ENSGACG00000005425	protein_coding
groupXI	12326299	12327385	ENSGACG00000012767	protein_coding	groupXX	3941043	3941558	ENSGACG00000005429	protein_coding
groupXI	12330952	12351249	ENSGACG00000012769	protein_coding	groupXX	6294803	6298923	ENSGACG00000006455	protein_coding
groupXI	12357662	12360295	ENSGACG00000012776	protein_coding	groupXX	8307535	8309553	ENSGACG00000007389	protein_coding
groupXI	12365393	12366157	ENSGACG00000012779	protein_coding	groupXX	13545827	13546986	ENSGACG00000012356	protein_coding

groupXI	12369682	12371008	ENSGACG00000012781	protein_coding	groupXX	13897472	13898933	ENSGACG00000012488	protein_coding
groupXI	12374730	12380140	ENSGACG00000012783	protein_coding	groupXX	13899090	13901444	ENSGACG00000012491	protein_coding
groupXI	12398198	12399317	ENSGACG00000012790	protein_coding	groupXX	14073176	14083419	ENSGACG00000012575	protein_coding
groupXI	12405271	12405919	ENSGACG00000012792	protein_coding	groupXX	14209795	14211021	ENSGACG00000012600	protein_coding
groupXI	12426250	12426681	ENSGACG00000012794	protein_coding	groupXX	14261297	14263562	ENSGACG00000012632	protein_coding
groupXI	12434699	12435788	ENSGACG00000012797	protein_coding	groupXX	14354574	14362816	ENSGACG00000012682	protein_coding
groupXI	12439443	12461313	ENSGACG00000012799	protein_coding	groupXX	14364277	14369124	ENSGACG00000012688	protein_coding
groupXI	12462274	12463350	ENSGACG00000012804	protein_coding	groupXX	15877173	15879199	ENSGACG00000013202	protein_coding
groupXI	12465740	12468183	ENSGACG00000012806	protein_coding	groupXX	15976803	15980796	ENSGACG00000013220	protein_coding
groupXI	12475199	12476534	ENSGACG00000012809	protein_coding	groupXX	18826557	18828313	ENSGACG00000014185	protein_coding
groupXI	12480535	12481571	ENSGACG00000012812	protein_coding	groupXXI	1563701	1567341	ENSGACG00000001778	protein_coding
groupXI	12683666	12685586	ENSGACG00000013046	protein_coding	groupXXI	1975032	1981586	ENSGACG00000001884	protein_coding
groupXI	12688018	12689938	ENSGACG00000013048	protein_coding	groupXXI	3413507	3415036	ENSGACG00000002237	pseudogene
groupXI	12692690	12694610	ENSGACG00000013051	protein_coding	groupXXI	3448709	3449346	ENSGACG00000002239	protein_coding
groupXI	12694207	12699627	ENSGACG00000013052	protein_coding	groupXXI	3449541	3450444	ENSGACG00000002241	protein_coding
groupXI	12697366	12699286	ENSGACG00000013055	protein_coding	groupXXI	3775350	3776625	ENSGACG00000002275	protein_coding
groupXI	12835444	12837440	ENSGACG00000013292	protein_coding	groupXXI	3782068	3790368	ENSGACG00000002276	protein_coding
groupXI	13646452	13647149	ENSGACG00000013876	protein_coding	groupXXI	3790781	3792345	ENSGACG00000002283	protein_coding
groupXI	15607307	15613431	ENSGACG00000014553	protein_coding	groupXXI	4613445	4616448	ENSGACG00000002399	protein_coding
groupXI	15608501	15610308	ENSGACG00000014556	protein_coding	groupXXI	4633728	4636042	ENSGACG00000002402	protein_coding
groupXI	15616018	15617314	ENSGACG00000014557	protein_coding	groupXXI	4653126	4655956	ENSGACG00000002404	protein_coding
groupXII	193051	219852	ENSGACG00000002604	protein_coding	groupXXI	4657259	4659904	ENSGACG00000002405	protein_coding
groupXII	890582	897690	ENSGACG00000002903	protein_coding	groupXXI	7946496	7947460	ENSGACG00000003404	protein_coding
groupXII	6444658	6446315	ENSGACG00000005562	protein_coding	groupXXI	7957299	7958968	ENSGACG00000003405	protein_coding
groupXII	8061270	8065049	ENSGACG00000006525	protein_coding	groupXXI	7959283	7960098	ENSGACG00000003407	protein_coding
groupXII	8067347	8071918	ENSGACG00000006529	protein_coding	groupXXI	7994018	7996973	ENSGACG00000003408	protein_coding
groupXII	8078414	8082300	ENSGACG00000006534	protein_coding	groupXXI	8131606	8134827	ENSGACG00000003553	protein_coding
groupXII	8093401	8100588	ENSGACG00000006536	protein_coding	scaffold_119	236539	237316	ENSGACG00000000420	protein_coding
groupXII	9995056	9998502	ENSGACG00000007655	protein_coding	scaffold_1255	1121	4319	ENSGACG00000018689	protein_coding
groupXII	10001183	10004350	ENSGACG00000007674	protein_coding	scaffold_129	135237	139395	ENSGACG00000001165	protein_coding
groupXII	12341960	12342267	ENSGACG00000022741	misc_RNA	scaffold_131	4214	13692	ENSGACG00000000304	protein_coding
groupXII	12343015	12343322	ENSGACG00000022842	misc_RNA	scaffold_131	15869	20132	ENSGACG00000000314	protein_coding
groupXII	12344167	12344474	ENSGACG00000022859	misc_RNA	scaffold_131	25852	29104	ENSGACG00000000318	protein_coding
groupXII	12345228	12345537	ENSGACG00000022817	misc_RNA	scaffold_131	32175	35453	ENSGACG00000000320	protein_coding

groupXII	16461684	16464104	ENSGACG00000012433	protein_coding	scaffold_131	38859	39828	ENSGACG00000000325	protein_coding
groupXII	16467460	16469054	ENSGACG00000012439	protein_coding	scaffold_131	45264	47851	ENSGACG00000000328	protein_coding
groupXII	16530361	16547407	ENSGACG00000012562	protein_coding	scaffold_131	52152	54217	ENSGACG00000000330	protein_coding
groupXII	16546734	16547573	ENSGACG00000012566	protein_coding	scaffold_131	56852	59742	ENSGACG00000000336	protein_coding
groupXIII	3156277	3158034	ENSGACG00000005312	protein_coding	scaffold_131	59837	100197	ENSGACG00000000343	protein_coding
groupXIII	3265049	3265662	ENSGACG00000005340	protein_coding	scaffold_131	78912	81169	ENSGACG00000000344	protein_coding
groupXIII	3287748	3291445	ENSGACG00000005345	protein_coding	scaffold_131	92495	94118	ENSGACG00000000345	protein_coding
groupXIII	4337517	4338066	ENSGACG00000005923	protein_coding	scaffold_131	96884	99355	ENSGACG00000000346	protein_coding
groupXIII	5142594	5143674	ENSGACG00000006643	protein_coding	scaffold_131	118612	120538	ENSGACG00000000348	protein_coding
groupXIII	7070566	7075041	ENSGACG00000007399	protein_coding	scaffold_135	66399	68194	ENSGACG00000015785	protein_coding
groupXIII	8978568	8989361	ENSGACG00000008971	protein_coding	scaffold_135	72729	74567	ENSGACG00000015787	protein_coding
groupXIII	16637320	16645064	ENSGACG00000013593	protein_coding	scaffold_135	85406	88235	ENSGACG00000015788	protein_coding
groupXIII	16775252	16782436	ENSGACG00000013659	protein_coding	scaffold_135	90779	92438	ENSGACG00000015789	protein_coding
groupXIII	17421767	17424812	ENSGACG00000013874	protein_coding	scaffold_137	45887	47060	ENSGACG00000017732	protein_coding
groupXIII	17427540	17430821	ENSGACG00000013879	protein_coding	scaffold_137	96946	97474	ENSGACG00000017734	protein_coding
groupXIII	18335507	18338213	ENSGACG00000014251	protein_coding	scaffold_137	101655	102467	ENSGACG00000017736	protein_coding
groupXIII	18467818	18470470	ENSGACG00000014266	protein_coding	scaffold_147	15584	16106	ENSGACG00000000669	protein_coding
groupXIII	18498299	18499267	ENSGACG00000014279	protein_coding	scaffold_147	42574	43652	ENSGACG00000000670	protein_coding
groupXIII	18539982	18541069	ENSGACG00000014282	protein_coding	scaffold_147	47785	150757	ENSGACG00000000672	protein_coding
groupXIII	18573306	18573822	ENSGACG00000014283	protein_coding	scaffold_147	63257	86855	ENSGACG00000000674	protein_coding
groupXIII	18582294	18583087	ENSGACG00000014284	protein_coding	scaffold_147	121552	190065	ENSGACG00000000676	protein_coding
groupXIII	18702444	18703421	ENSGACG00000014302	protein_coding	scaffold_147	141819	142551	ENSGACG00000000677	protein_coding
groupXIII	18748570	18752248	ENSGACG00000014305	protein_coding	scaffold_147	181547	182520	ENSGACG00000000678	protein_coding
groupXIII	19483341	19484748	ENSGACG00000014628	protein_coding	scaffold_171	10929	65821	ENSGACG00000001031	protein_coding
groupXIV	588295	588826	ENSGACG00000015649	protein_coding	scaffold_171	42527	44144	ENSGACG00000001032	protein_coding
groupXIV	3230207	3233517	ENSGACG00000016160	protein_coding	scaffold_171	55059	57157	ENSGACG00000001033	protein_coding
groupXIV	7842402	7845250	ENSGACG00000017452	protein_coding	scaffold_171	68709	75188	ENSGACG00000001034	protein_coding
groupXIV	7864834	7867803	ENSGACG00000017454	pseudogene	scaffold_171	78218	79850	ENSGACG00000001036	protein_coding
groupXIV	10842497	10843277	ENSGACG00000018068	protein_coding	scaffold_171	111132	112386	ENSGACG00000001037	protein_coding
groupXIV	13384174	13385999	ENSGACG00000018283	protein_coding	scaffold_171	119731	121255	ENSGACG00000001038	protein_coding
groupXIV	14271041	14272180	ENSGACG00000018423	protein_coding	scaffold_171	122345	134981	ENSGACG00000001040	protein_coding
groupXV	818703	822144	ENSGACG00000004683	protein_coding	scaffold_171	128655	130756	ENSGACG00000001044	protein_coding
groupXV	825196	829221	ENSGACG00000004692	protein_coding	scaffold_171	135483	139900	ENSGACG00000001045	protein_coding
groupXV	1444643	1453495	ENSGACG00000005133	protein_coding	scaffold_171	144633	158832	ENSGACG00000001047	protein_coding

groupXV	1454412	1458371	ENSGACG00000005156	protein_coding	scaffold_171	161158	163857	ENSGACG00000001051	protein_coding
groupXV	1524238	1525287	ENSGACG00000005289	protein_coding	scaffold_171	170781	172515	ENSGACG00000001053	protein_coding
groupXV	1860723	1867957	ENSGACG00000005683	protein_coding	scaffold_171	177057	179667	ENSGACG00000001054	protein_coding
groupXV	3012132	3032134	ENSGACG00000006713	protein_coding	scaffold_171	182320	184517	ENSGACG00000001057	protein_coding
groupXV	8067464	8068070	ENSGACG00000009919	protein_coding	scaffold_172	30857	31670	ENSGACG00000001756	protein_coding
groupXV	8067467	8068457	ENSGACG00000009920	protein_coding	scaffold_172	37037	37586	ENSGACG00000001757	protein_coding
groupXV	9544136	9545953	ENSGACG00000010578	protein_coding	scaffold_172	37910	39599	ENSGACG00000001759	protein_coding
groupXV	9769055	9769691	ENSGACG00000010717	protein_coding	scaffold_172	57135	72609	ENSGACG00000001764	protein_coding
groupXV	9774816	9775406	ENSGACG00000010718	protein_coding	scaffold_172	95274	103789	ENSGACG00000001770	protein_coding
groupXV	9778276	9790801	ENSGACG00000010720	protein_coding	scaffold_172	136170	136902	ENSGACG00000001772	protein_coding
groupXV	9785257	9785536	ENSGACG00000010724	protein_coding	scaffold_172	157563	158079	ENSGACG00000001774	protein_coding
groupXV	9790740	9791523	ENSGACG00000010727	protein_coding	scaffold_172	164463	166828	ENSGACG00000001775	protein_coding
groupXV	9795909	9797720	ENSGACG00000010728	protein_coding	scaffold_173	85478	86638	ENSGACG00000001444	pseudogene
groupXVI	495658	497657	ENSGACG00000001535	protein_coding	scaffold_180	1398	13644	ENSGACG00000001596	protein_coding
groupXVI	504579	510768	ENSGACG00000001536	protein_coding	scaffold_180	16522	17577	ENSGACG00000001598	protein_coding
groupXVI	513061	514874	ENSGACG00000001537	protein_coding	scaffold_180	18491	25815	ENSGACG00000001600	protein_coding
groupXVI	3895611	3897421	ENSGACG00000002127	protein_coding	scaffold_180	28649	36301	ENSGACG00000001608	protein_coding
groupXVI	4224686	4226260	ENSGACG00000002202	protein_coding	scaffold_180	53986	55637	ENSGACG00000001618	protein_coding
groupXVI	4234862	4236981	ENSGACG00000002206	protein_coding	scaffold_180	69423	106380	ENSGACG00000001624	protein_coding
groupXVI	4249199	4250141	ENSGACG00000002209	protein_coding	scaffold_180	69457	72266	ENSGACG00000001627	protein_coding
groupXVI	4252437	4253460	ENSGACG00000002211	protein_coding	scaffold_180	74609	77849	ENSGACG00000001630	protein_coding
groupXVI	4285590	4286514	ENSGACG00000002212	protein_coding	scaffold_180	90677	100224	ENSGACG00000001632	protein_coding
groupXVI	4293606	4294530	ENSGACG00000002214	protein_coding	scaffold_180	105653	110675	ENSGACG00000001639	protein_coding
groupXVI	4301584	4303257	ENSGACG00000002215	protein_coding	scaffold_185	398	115772	ENSGACG00000010493	protein_coding
groupXVI	4667417	4667523	ENSGACG00000021158	snRNA	scaffold_185	29117	109657	ENSGACG00000010499	protein_coding
groupXVI	4681909	4682015	ENSGACG00000021180	snRNA	scaffold_185	34964	145602	ENSGACG00000010501	protein_coding
groupXVI	4690277	4690383	ENSGACG00000022013	snRNA	scaffold_193	36452	37197	ENSGACG00000000571	protein_coding
groupXVI	4703391	4713584	ENSGACG00000002240	protein_coding	scaffold_193	46144	65612	ENSGACG00000000574	protein_coding
groupXVI	4733213	4733729	ENSGACG00000002251	protein_coding	scaffold_193	73529	101000	ENSGACG00000000577	protein_coding
groupXVI	4753831	4754968	ENSGACG00000002252	protein_coding	scaffold_197	16261	16993	ENSGACG00000000842	protein_coding
groupXVI	4759463	4761705	ENSGACG00000002254	protein_coding	scaffold_201	93539	104242	ENSGACG00000000902	protein_coding
groupXVI	4784786	4787328	ENSGACG00000002255	protein_coding	scaffold_224	926	1406	ENSGACG00000001249	protein_coding
groupXVI	4814441	4815533	ENSGACG00000002259	protein_coding	scaffold_224	2361	2733	ENSGACG00000001250	protein_coding
groupXVI	4855584	4856795	ENSGACG00000002261	protein_coding	scaffold_224	7485	7797	ENSGACG00000001251	protein_coding

groupXVI	8322442	8338653	ENSGACG00000003664	protein_coding	scaffold_224	10267	11827	ENSGACG00000001253	protein_coding
groupXVI	8555741	8556746	ENSGACG00000003770	protein_coding	scaffold_224	11864	12418	ENSGACG00000001256	protein_coding
groupXVI	11775069	11783061	ENSGACG00000006218	protein_coding	scaffold_224	12762	13173	ENSGACG00000001259	protein_coding
groupXVI	13813114	13814761	ENSGACG00000006948	protein_coding	scaffold_224	14139	22088	ENSGACG00000001261	protein_coding
groupXVI	13817594	13819315	ENSGACG00000006952	protein_coding	scaffold_224	15244	15363	ENSGACG00000021592	rRNA
groupXVI	13843541	13846698	ENSGACG00000006957	protein_coding	scaffold_224	15480	15599	ENSGACG00000021022	rRNA
groupXVI	13877316	13879014	ENSGACG00000006966	protein_coding	scaffold_224	24818	25130	ENSGACG00000001263	protein_coding
groupXVI	13880713	13881646	ENSGACG00000006969	protein_coding	scaffold_224	27087	27459	ENSGACG00000001265	protein_coding
groupXVI	13885491	13888462	ENSGACG00000006971	protein_coding	scaffold_224	27930	28691	ENSGACG00000001266	protein_coding
groupXVI	13896722	13897679	ENSGACG00000006973	protein_coding	scaffold_229	6996	8223	ENSGACG00000000546	protein_coding
groupXVI	13901515	13901815	ENSGACG00000006974	protein_coding	scaffold_229	28614	29289	ENSGACG00000000547	protein_coding
groupXVI	13904890	13906343	ENSGACG00000006976	protein_coding	scaffold_229	33376	47417	ENSGACG00000000548	protein_coding
groupXVI	13914788	13916661	ENSGACG00000006977	protein_coding	scaffold_229	61941	62349	ENSGACG00000000549	protein_coding
groupXVI	16368348	16369778	ENSGACG00000008301	protein_coding	scaffold_232	280	4420	ENSGACG00000001736	protein_coding
groupXVI	16648439	16649317	ENSGACG00000008457	protein_coding	scaffold_232	44158	79908	ENSGACG00000001742	protein_coding
groupXVI	16974283	16975596	ENSGACG00000008545	protein_coding	scaffold_232	167843	171745	ENSGACG00000001748	protein_coding
groupXVI	16978740	16979885	ENSGACG00000008547	protein_coding	scaffold_252	3830	5766	ENSGACG00000001319	protein_coding
groupXVI	17028080	17029718	ENSGACG00000008551	protein_coding	scaffold_252	10915	11421	ENSGACG00000001321	protein_coding
groupXVI	17057146	17058154	ENSGACG00000008554	protein_coding	scaffold_252	26780	31802	ENSGACG00000001322	protein_coding
groupXVI	17065775	17066988	ENSGACG00000008556	protein_coding	scaffold_27	2624406	2634901	ENSGACG00000000906	protein_coding
groupXVI	17124130	17125629	ENSGACG00000008557	protein_coding	scaffold_27	2637090	2638365	ENSGACG00000000911	protein_coding
groupXVI	17151884	17152878	ENSGACG00000008561	protein_coding	scaffold_27	2917816	2922016	ENSGACG00000001011	protein_coding
groupXVI	17216675	17216755	ENSGACG00000022535	miRNA	scaffold_27	4615559	4635531	ENSGACG00000001573	protein_coding
groupXVII	106907	109258	ENSGACG00000003039	protein_coding	scaffold_27	4635810	4639569	ENSGACG00000001585	protein_coding
groupXVII	612702	614501	ENSGACG00000003421	protein_coding	scaffold_290	9707	58884	ENSGACG00000000779	protein_coding
groupXVII	1834691	1835293	ENSGACG00000004312	protein_coding	scaffold_290	58570	59630	ENSGACG00000000780	protein_coding
groupXVII	1839282	1840721	ENSGACG00000004316	protein_coding	scaffold_299	12721	14758	ENSGACG00000000238	protein_coding
groupXVII	1843066	1843668	ENSGACG00000004322	protein_coding	scaffold_299	19074	23207	ENSGACG00000000240	protein_coding
groupXVII	1847556	1848158	ENSGACG00000004326	protein_coding	scaffold_299	26620	31480	ENSGACG00000000241	protein_coding
groupXVII	6049811	6053179	ENSGACG00000007307	protein_coding	scaffold_316	9386	26786	ENSGACG00000000785	protein_coding
groupXVII	6514459	6515044	ENSGACG00000007607	protein_coding	scaffold_320	2284	3790	ENSGACG00000001341	protein_coding
groupXVII	11171667	11172833	ENSGACG00000010909	protein_coding	scaffold_320	2754	19009	ENSGACG00000001342	protein_coding
groupXVII	11175540	11184052	ENSGACG00000010910	protein_coding	scaffold_320	9178	19063	ENSGACG00000001344	protein_coding
groupXVIII	5955323	5956118	ENSGACG00000007154	protein_coding	scaffold_320	23018	26275	ENSGACG00000001347	protein_coding

groupXVIII	12997842	12999531	ENSGACG00000012051	protein_coding	scaffold_320	26488	27925	ENSGACG00000001350	protein_coding
groupXVIII	13108711	13110767	ENSGACG00000012062	protein_coding	scaffold_321	40890	42189	ENSGACG00000005692	protein_coding
groupXVIII	13111961	13116856	ENSGACG00000012065	protein_coding	scaffold_327	90	8820	ENSGACG00000014758	protein_coding
groupXVIII	13185259	13186039	ENSGACG00000012073	protein_coding	scaffold_327	11415	11506	ENSGACG00000022829	miRNA
groupXVIII	13190410	13191616	ENSGACG00000012075	protein_coding	scaffold_327	19940	20969	ENSGACG00000014762	protein_coding
groupXVIII	13234806	13236773	ENSGACG00000012133	protein_coding	scaffold_330	7476	22522	ENSGACG00000001805	protein_coding
groupXVIII	15307547	15316206	ENSGACG00000013216	protein_coding	scaffold_330	23988	25609	ENSGACG00000001807	protein_coding
groupXVIII	15327716	15352321	ENSGACG00000013229	protein_coding	scaffold_332	1942	12505	ENSGACG00000001260	protein_coding
groupXVIII	15820422	15820995	ENSGACG00000013545	protein_coding	scaffold_332	23002	24274	ENSGACG00000001264	protein_coding
groupXVIII	15825270	15830242	ENSGACG00000013546	protein_coding	scaffold_332	25355	28790	ENSGACG00000001267	protein_coding
groupXX	251019	263441	ENSGACG00000003006	protein_coding	scaffold_337	21091	22328	ENSGACG00000001277	protein_coding
groupXX	263857	265269	ENSGACG00000003010	protein_coding	scaffold_346	14462	20903	ENSGACG00000000580	protein_coding
groupXX	267051	268978	ENSGACG00000003011	protein_coding	scaffold_358	21314	22955	ENSGACG00000001386	protein_coding
groupXX	271546	274521	ENSGACG00000003015	protein_coding	scaffold_360	2646	2838	ENSGACG00000021674	snRNA
groupXX	279325	280477	ENSGACG00000003030	protein_coding	scaffold_360	3114	3278	ENSGACG00000021033	snRNA
groupXX	7598428	7599090	ENSGACG00000006682	protein_coding	scaffold_360	3755	3872	ENSGACG00000022684	snRNA
groupXX	8675984	8677025	ENSGACG00000007491	protein_coding	scaffold_360	4390	4504	ENSGACG00000022773	snRNA
groupXX	10257430	10258487	ENSGACG00000008828	protein_coding	scaffold_360	4701	5211	ENSGACG00000018075	protein_coding
groupXX	13509245	13512236	ENSGACG00000012345	protein_coding	scaffold_360	11167	11313	ENSGACG00000021740	snRNA
groupXX	13513401	13517536	ENSGACG00000012348	protein_coding	scaffold_360	11631	11795	ENSGACG00000021705	snRNA
groupXX	13525609	13528280	ENSGACG00000012351	protein_coding	scaffold_360	12272	12389	ENSGACG00000022787	snRNA
groupXX	13534108	13540477	ENSGACG00000012354	protein_coding	scaffold_360	13031	13145	ENSGACG00000022784	snRNA
groupXX	13537537	13543688	ENSGACG00000012355	protein_coding	scaffold_360	41652	41791	ENSGACG00000021644	snRNA
groupXX	13545827	13546986	ENSGACG00000012356	protein_coding	scaffold_360	42630	42747	ENSGACG00000022833	snRNA
groupXX	13555738	13560232	ENSGACG00000012358	protein_coding	scaffold_360	43265	43456	ENSGACG00000021085	snRNA
groupXX	14098313	14139985	ENSGACG00000012578	protein_coding	scaffold_360	43732	43896	ENSGACG00000021999	snRNA
groupXX	14171556	14173102	ENSGACG00000012597	protein_coding	scaffold_360	44373	44490	ENSGACG00000022769	snRNA
groupXX	14209795	14211021	ENSGACG00000012600	protein_coding	scaffold_360	45008	45199	ENSGACG00000021051	snRNA
groupXXI	13472	14259	ENSGACG00000001645	protein_coding	scaffold_360	46684	46831	ENSGACG00000021941	snRNA
groupXXI	47444	48785	ENSGACG00000001646	protein_coding	scaffold_360	47114	47278	ENSGACG00000021138	snRNA
groupXXI	89382	89938	ENSGACG00000001650	protein_coding	scaffold_360	47755	47872	ENSGACG00000022863	snRNA
groupXXI	125155	125948	ENSGACG00000001651	protein_coding	scaffold_360	48390	48504	ENSGACG00000022821	snRNA
groupXXI	240731	252486	ENSGACG00000001652	protein_coding	scaffold_364	9740	17234	ENSGACG00000000857	protein_coding
groupXXI	3109191	3205745	ENSGACG00000002235	protein_coding	scaffold_37	629536	638040	ENSGACG00000000879	protein_coding

groupXXI	3413507	3415036	ENSGACG00000002237	pseudogene	scaffold_37	815922	817008	ENSGACG00000000904	protein_coding
groupXXI	3448709	3449346	ENSGACG00000002239	protein_coding	scaffold_374	2437	3372	ENSGACG00000000190	protein_coding
groupXXI	3449541	3450444	ENSGACG00000002241	protein_coding	scaffold_416	12913	15741	ENSGACG00000000537	protein_coding
groupXXI	5516656	5518336	ENSGACG00000002473	protein_coding	scaffold_434	1322	4701	ENSGACG00000001749	protein_coding
groupXXI	7946496	7947460	ENSGACG00000003404	protein_coding	scaffold_434	8775	11798	ENSGACG00000001763	protein_coding
groupXXI	11402390	11413955	ENSGACG00000005504	protein_coding	scaffold_470	45	16048	ENSGACG00000010393	protein_coding
groupXXI	11416737	11421937	ENSGACG00000005519	protein_coding	scaffold_470	9070	10422	ENSGACG00000010400	protein_coding
groupXXI	11434877	11481114	ENSGACG00000005524	protein_coding	scaffold_519	7016	8809	ENSGACG00000005704	protein_coding
groupXXI	11470555	11471458	ENSGACG00000005526	protein_coding	scaffold_521	13588	14932	ENSGACG00000015703	protein_coding
scaffold_1026	3901	4432	ENSGACG00000001967	protein_coding	scaffold_528	4782	4863	ENSGACG00000022758	miRNA
scaffold_121	2255	9077	ENSGACG00000001067	protein_coding	scaffold_528	5001	5074	ENSGACG00000022716	miRNA
scaffold_126	258852	285713	ENSGACG00000012920	protein_coding	scaffold_528	5212	5285	ENSGACG00000022866	miRNA
scaffold_131	52152	54217	ENSGACG00000000330	protein_coding	scaffold_528	6792	6946	ENSGACG00000022103	rRNA
scaffold_131	56852	59742	ENSGACG00000000336	protein_coding	scaffold_536	10135	11899	ENSGACG00000014258	protein_coding
scaffold_131	78912	81169	ENSGACG00000000344	protein_coding	scaffold_54	4287	4375	ENSGACG00000022837	miRNA
scaffold_131	96884	99355	ENSGACG00000000346	protein_coding	scaffold_54	4741	4822	ENSGACG00000022889	miRNA
scaffold_131	118612	120538	ENSGACG00000000348	protein_coding	scaffold_54	5905	5997	ENSGACG00000022144	miRNA
scaffold_137	14736	19646	ENSGACG00000017731	protein_coding	scaffold_54	8027	8181	ENSGACG00000021780	rRNA
scaffold_137	45887	47060	ENSGACG00000017732	protein_coding	scaffold_54	8591	8978	ENSGACG00000002131	protein_coding
scaffold_137	96946	97474	ENSGACG00000017734	protein_coding	scaffold_54	21188	21276	ENSGACG00000022725	miRNA
scaffold_137	101655	102467	ENSGACG00000017736	protein_coding	scaffold_54	675188	675294	ENSGACG00000021610	snRNA
scaffold_139	182184	194299	ENSGACG00000000517	protein_coding	scaffold_54	677006	677112	ENSGACG00000022053	snRNA
scaffold_159	32187	32712	ENSGACG00000010811	protein_coding	scaffold_54	678824	678930	ENSGACG00000021154	snRNA
scaffold_159	41909	43353	ENSGACG00000010812	protein_coding	scaffold_54	680641	680747	ENSGACG00000022042	snRNA
scaffold_159	47730	70137	ENSGACG00000010813	protein_coding	scaffold_54	682458	682564	ENSGACG00000022133	snRNA
scaffold_159	50899	51831	ENSGACG00000010831	protein_coding	scaffold_54	684276	684382	ENSGACG00000021157	snRNA
scaffold_173	85478	86638	ENSGACG00000001444	pseudogene	scaffold_54	712866	712972	ENSGACG00000021096	snRNA
scaffold_178	1037	1151	ENSGACG00000022688	rRNA	scaffold_54	716253	716359	ENSGACG00000021630	snRNA
scaffold_178	22787	24269	ENSGACG00000001724	protein_coding	scaffold_56	988057	989781	ENSGACG00000002148	protein_coding
scaffold_180	105653	110675	ENSGACG00000001639	protein_coding	scaffold_56	991931	1000901	ENSGACG00000002150	protein_coding
scaffold_198	54785	56486	ENSGACG00000000193	protein_coding	scaffold_56	1074909	1075028	ENSGACG00000020968	rRNA
scaffold_198	75484	76330	ENSGACG00000000194	protein_coding	scaffold_56	1075144	1075262	ENSGACG00000021717	rRNA
scaffold_198	105652	108706	ENSGACG00000000196	protein_coding	scaffold_56	1075471	1075635	ENSGACG00000021183	snRNA
scaffold_200	75101	75635	ENSGACG00000013519	protein_coding	scaffold_56	1076140	1076257	ENSGACG00000021620	snRNA

scaffold_200	86476	87692	ENSGACG00000013520	protein_coding	scaffold_56	1076754	1076868	ENSGACG00000022720	snRNA
scaffold_201	4552	84629	ENSGACG00000000899	protein_coding	scaffold_56	1117520	1124196	ENSGACG00000002171	protein_coding
scaffold_201	93539	104242	ENSGACG00000000902	protein_coding	scaffold_571	7447	8018	ENSGACG00000001394	protein_coding
scaffold_201	119656	119740	ENSGACG00000022888	snoRNA	scaffold_571	8263	8417	ENSGACG00000021870	rRNA
scaffold_224	184788	187600	ENSGACG00000001271	protein_coding	scaffold_571	10422	10514	ENSGACG00000022145	miRNA
scaffold_224	200823	201574	ENSGACG00000001272	protein_coding	scaffold_571	10533	12180	ENSGACG00000001396	protein_coding
scaffold_224	202054	202459	ENSGACG00000001273	protein_coding	scaffold_571	11372	11458	ENSGACG00000022835	miRNA
scaffold_224	205719	206031	ENSGACG00000001274	protein_coding	scaffold_571	11589	11670	ENSGACG00000022726	miRNA
scaffold_229	6996	8223	ENSGACG00000000546	protein_coding	scaffold_571	12034	12122	ENSGACG00000022840	miRNA
scaffold_229	28614	29289	ENSGACG00000000547	protein_coding	scaffold_58	45508	45667	ENSGACG00000022875	miRNA
scaffold_229	33376	47417	ENSGACG00000000548	protein_coding	scaffold_58	73155	133895	ENSGACG00000000067	protein_coding
scaffold_229	61941	62349	ENSGACG00000000549	protein_coding	scaffold_58	178703	180428	ENSGACG00000000071	protein_coding
scaffold_232	280	4420	ENSGACG00000001736	protein_coding	scaffold_58	393129	437552	ENSGACG00000000074	protein_coding
scaffold_232	44158	79908	ENSGACG00000001742	protein_coding	scaffold_58	478180	489125	ENSGACG00000000080	protein_coding
scaffold_232	167843	171745	ENSGACG00000001748	protein_coding	scaffold_58	493137	497886	ENSGACG00000000083	protein_coding
scaffold_253	38409	42330	ENSGACG00000000918	protein_coding	scaffold_58	498184	499671	ENSGACG00000000085	protein_coding
scaffold_253	52822	55474	ENSGACG00000000921	protein_coding	scaffold_58	510327	533032	ENSGACG00000000088	protein_coding
scaffold_290	9707	58884	ENSGACG00000000779	protein_coding	scaffold_58	534761	541524	ENSGACG00000000091	protein_coding
scaffold_290	58570	59630	ENSGACG00000000780	protein_coding	scaffold_58	551252	556074	ENSGACG00000000092	protein_coding
scaffold_327	90	8820	ENSGACG00000014758	protein_coding	scaffold_58	590629	595294	ENSGACG00000000093	protein_coding
scaffold_327	11415	11506	ENSGACG00000022829	miRNA	scaffold_58	597531	604158	ENSGACG00000000094	protein_coding
scaffold_327	19940	20969	ENSGACG00000014762	protein_coding	scaffold_58	624877	624956	ENSGACG00000021299	miRNA
scaffold_332	1942	12505	ENSGACG00000001260	protein_coding	scaffold_58	629373	641683	ENSGACG00000000096	protein_coding
scaffold_332	23002	24274	ENSGACG00000001264	protein_coding	scaffold_58	671488	700824	ENSGACG00000000099	protein_coding
scaffold_332	25355	28790	ENSGACG00000001267	protein_coding	scaffold_58	706702	713823	ENSGACG00000000103	protein_coding
scaffold_37	461315	477261	ENSGACG00000000864	protein_coding	scaffold_590	3989	5947	ENSGACG00000000389	protein_coding
scaffold_37	629536	638040	ENSGACG00000000879	protein_coding	scaffold_599	8866	12216	ENSGACG00000001046	protein_coding
scaffold_37	815922	817008	ENSGACG00000000904	protein_coding	scaffold_615	8204	12763	ENSGACG00000000834	protein_coding
scaffold_37	830906	832333	ENSGACG00000000907	protein_coding	scaffold_777	51	1943	ENSGACG00000000763	protein_coding
scaffold_37	845276	846902	ENSGACG00000000908	protein_coding	scaffold_797	4881	4990	ENSGACG00000022843	miRNA
scaffold_37	852504	855055	ENSGACG00000000912	protein_coding	scaffold_797	7354	8542	ENSGACG00000001335	protein_coding
scaffold_37	944486	945218	ENSGACG00000000931	protein_coding	scaffold_844	2819	2966	ENSGACG00000021101	snRNA
scaffold_37	1868617	1869815	ENSGACG00000000983	protein_coding	scaffold_844	3248	3412	ENSGACG00000021584	snRNA
scaffold_37	1893789	1895000	ENSGACG00000000984	protein_coding	scaffold_844	3854	3971	ENSGACG00000022893	snRNA

scaffold_47	647939	650522	ENSGACG00000000797	protein_coding
scaffold_48	946100	946901	ENSGACG00000015309	protein_coding
scaffold_54	515452	533705	ENSGACG00000002204	protein_coding
scaffold_54	712866	712972	ENSGACG00000021096	snRNA
scaffold_54	716253	716359	ENSGACG00000021630	snRNA
scaffold_54	721593	725829	ENSGACG00000002281	protein_coding
scaffold_54	722666	722772	ENSGACG00000021191	snRNA
scaffold_54	726050	726156	ENSGACG00000021023	snRNA
scaffold_56	988057	989781	ENSGACG00000002148	protein_coding
scaffold_56	1059526	1060737	ENSGACG00000002165	protein_coding
scaffold_56	1076140	1076257	ENSGACG00000021620	snRNA
scaffold_56	1076754	1076868	ENSGACG00000022720	snRNA
scaffold_56	1109846	1110753	ENSGACG00000002169	protein_coding
scaffold_56	1117520	1124196	ENSGACG00000002171	protein_coding
scaffold_67	618744	619392	ENSGACG00000000591	protein_coding
scaffold_678	985	2270	ENSGACG00000000408	protein_coding
scaffold_678	9847	10399	ENSGACG00000000409	protein_coding
scaffold_68	795021	803890	ENSGACG00000015224	protein_coding
scaffold_68	807895	809365	ENSGACG00000015226	protein_coding
scaffold_80	311449	330628	ENSGACG00000000469	protein_coding
scaffold_84	93840	94222	ENSGACG00000020136	protein_coding
scaffold_84	388393	389076	ENSGACG00000020146	pseudogene
scaffold_89	26629	27862	ENSGACG00000000007	protein_coding
scaffold_89	51243	53901	ENSGACG00000000008	protein_coding
scaffold_95	88544	89367	ENSGACG00000000076	protein_coding
scaffold_95	93512	94037	ENSGACG00000000077	protein_coding
scaffold_844	4570	4761	ENSGACG00000020989	snRNA
scaffold_844	5037	5201	ENSGACG00000022052	snRNA
scaffold_844	5643	5760	ENSGACG00000022824	snRNA
scaffold_844	6376	6567	ENSGACG00000022030	snRNA
scaffold_844	6842	7006	ENSGACG00000021801	snRNA
scaffold_844	7448	7565	ENSGACG00000022736	snRNA
scaffold_844	8165	8356	ENSGACG00000021182	snRNA
scaffold_844	8632	8796	ENSGACG00000021087	snRNA
scaffold_88	33712	33868	ENSGACG00000021704	rRNA
scaffold_88	49501	49587	ENSGACG00000022797	miRNA
scaffold_88	50448	50540	ENSGACG00000022146	miRNA
scaffold_88	52646	52800	ENSGACG00000022063	rRNA
scaffold_94	244763	246266	ENSGACG00000011221	protein_coding
scaffold_94	340145	340661	ENSGACG00000011222	protein_coding
scaffold_94	351657	352197	ENSGACG00000011224	protein_coding
scaffold_94	351829	387624	ENSGACG00000011226	protein_coding
scaffold_94	381419	382212	ENSGACG00000011229	protein_coding
scaffold_94	407289	409612	ENSGACG00000011232	protein_coding
scaffold_94	414835	415357	ENSGACG00000011234	protein_coding
scaffold_94	461257	473783	ENSGACG00000011237	protein_coding
scaffold_94	483063	484036	ENSGACG00000011242	protein_coding
scaffold_98	279077	279218	ENSGACG00000022040	snRNA
scaffold_98	279703	279844	ENSGACG00000021881	snRNA
scaffold_98	280329	280470	ENSGACG00000021761	snRNA
scaffold_98	280955	281096	ENSGACG00000021732	snRNA
scaffold_98	281581	281722	ENSGACG00000021993	snRNA
scaffold_98	282207	282348	ENSGACG00000021185	snRNA
scaffold_98	282833	282974	ENSGACG00000022132	snRNA
scaffold_98	283459	283600	ENSGACG00000021924	snRNA
scaffold_98	284085	284226	ENSGACG00000021194	snRNA
scaffold_98	284711	284852	ENSGACG00000022056	snRNA
scaffold_98	285337	285478	ENSGACG00000021192	snRNA
scaffold_98	285963	286102	ENSGACG00000021574	snRNA
scaffold_98	286584	286725	ENSGACG00000021127	snRNA

scaffold_98	287210	287352	ENSGACG00000021887	snRNA
scaffold_98	288489	288630	ENSGACG00000021713	snRNA
scaffold_98	289115	289256	ENSGACG00000021059	snRNA
scaffold_98	289741	289882	ENSGACG00000021931	snRNA
scaffold_98	290367	290508	ENSGACG00000020992	snRNA
scaffold_98	290995	291136	ENSGACG00000021733	snRNA
scaffold_98	291621	291762	ENSGACG00000021686	snRNA
scaffold_98	292247	292388	ENSGACG00000022004	snRNA
scaffold_98	292873	293014	ENSGACG00000022093	snRNA
scaffold_98	293499	293640	ENSGACG00000021695	snRNA
scaffold_98	295417	295558	ENSGACG00000021835	snRNA
scaffold_98	296043	296184	ENSGACG00000021163	snRNA
