# Supplementary material for: Extensive Copy-Number Variation of Young Genes across Stickleback Populations
Source: PLoS Genet. 2014 Dec 4;10(12):e1004830. doi: 10.1371/journal.pgen.1004830 (PMC4256280; doi:10.1371/journal.pgen.1004830)
Supplement: Table S12 — Number and proportion of autosomal genes across different categories with no significant BLAST protein hits in other fish from Ensembl v68. (PDF) [file pgen.1004830.s034.pdf]

Supplementary Table 12 - Number and proportion of autosomal genes across different categories with no significant BLAST protein hits in other fish from Ensembl v68

	Genes	Genes without significant hits	
		Number	Proportion
Genes	**19766**	779	0.03941111
LSG Singletons	972	618	0.635802469
LSG LSD	215	52	0.241860465
Non-LSG LSD	1585	8	0.005047319
Non-LSG Paralogs	13158	33	0.00250798
Non-LSG Singletons	3836	68	0.017726799
