# Supplementary material for: Extensive Copy-Number Variation of Young Genes across Stickleback Populations
Source: PLoS Genet. 2014 Dec 4;10(12):e1004830. doi: 10.1371/journal.pgen.1004830 (PMC4256280; doi:10.1371/journal.pgen.1004830)
Supplement: Table S13 — Primers used for validating CNVs. (PDF) [file pgen.1004830.s035.pdf]

Supplementary Table 13 - Primers used for validating CNVs

| Chrom. | Start | End | Upstream Primer (Forward) | Downstream Primer (Reverse) | Internal Primer (Reverse) | Variant Type | Validation | Validated | Pearson | pvalue | qvalue-fdr | Bonferroni | Concordant Genotypes |
|---|---|---|---|---|---|---|---|---|---|---|---|---|---|
| groupII | 11738047 | 11738144 | TGTACCATCGAGCGACGA | AGCCTTCCACATGTTCCAGT | NA | duplication | qPCR | yes | 0.888 | 4.44E-16 | 3.77E-15 | 7.55E-15 | NA of 45 |
| groupIV | 21171518 | 21171626 | CGCACATGCACTTCTCTTTC | AGGTCTGTCCTTGGTTCAGG | NA | deletion | qPCR | yes | 0.622 | 2.41E-05 | 4.10E-05 | 4.10E-04 | NA of 39 |
| groupIX | 13635509 | 13635649 | GTCGCTGGTGATTCTGTCTG | GCTCCCAGTAAAGCAGGTTC | NA | duplication | qPCR | yes | 0.981 | 0 | 0 | 0 | NA of 27 |
| groupIX | 14921708 | 14921803 | GGTTGCTGTTGGATGTAAGC | GCAGCCCAGTGTAAATATCG | NA | duplication | qPCR | yes | 0.942 | 2.15E-13 | 9.14E-13 | 3.66E-12 | NA of 27 |
| groupVI | 9255329 | 9255422 | GCAGGTAACATGCCAACTCA | GCTTGAGTGGGTTCCTCAAT | NA | duplication | qPCR | yes | 0.538 | 1.49E-03 | 1.58E-03 | 0.025 | NA of 32 |
| groupVI | 9257151 | 9257235 | TTCACACCCCAGGTAAGTCA | CCAGCTCAGTCATGGAAAAA | NA | duplication | qPCR | yes | 0.715 | 2.80E-05 | 4.33E-05 | 4.77E-04 | NA of 27 |
| groupVI | 14528182 | 14528288 | TTGACGGTCTGGATGAGTGT | GATGAGGTTTGTGAGGAGCA | NA | duplication | qPCR | yes | 0.610 | 9.36E-04 | 1.06E-03 | 0.016 | NA of 26 |
| groupVIII | 1526552 | 1526672 | CCTAAACACGGGGAGCTAAT | TCTAGAGCCGACCTTTGACC | NA | duplication | qPCR | yes | 0.615 | 1.08E-04 | 1.53E-04 | 1.83E-03 | NA of 34 |
| groupVIII | 1527610 | 1527751 | GGGTCACGTTTCCTCAAGA | AAGCCGCCAAAGGAACTAC | NA | duplication | qPCR | yes | 0.724 | 2.08E-04 | 2.72E-04 | 3.54E-03 | NA of 21 |
| groupVIII | 1540773 | 1540877 | GAACCCGCAGCAGAAAAGT | AGCTCCGTGTGTGGAAGTTA | NA | duplication | qPCR | yes | 0.832 | 7.25E-08 | 2.05E-07 | 1.23E-06 | NA of 27 |
| groupXIX | 1598074 | 1598160 | ATAACACATGGCAGCGTTTC | GCTACTGTCATCCTGCCTCA | NA | duplication | qPCR | yes | 0.985 | 8.88E-16 | 5.03E-15 | 1.51E-14 | NA of 21 |
| groupXIX | 1598141 | 1598248 | TGAGGCAGGATGACAGTAGC | CCCGTATGACAGGAGGAATC | NA | duplication | qPCR | yes | 0.961 | 1.65E-11 | 5.61E-11 | 2.81E-10 | NA of 20 |
| groupXIX | 3947152 | 3947255 | CAAGTCCCATTGACGACCT | TTGGCAGATTTGTGTGTGC | NA | duplication | qPCR | yes | 0.668 | 6.86E-04 | 8.33E-04 | 0.012 | NA of 22 |
| groupXVI | 11775836 | 11775977 | GGTTTTCATTCAGCACAGGA | CGGCCTGATTGTTTGACTAA | NA | duplication | qPCR | no | 0.445 | 0.011 | 0.011 | 0.181 | NA of 32 |
| groupXVIII | 9391 | 9470 | AGATCCACAAAGACCCTGCT | GCTGCTACAGTCGGAGACAA | NA | duplication | qPCR | yes | 0.744 | 1.31E-05 | 2.79E-05 | 2.23E-04 | NA of 26 |
| scaffold_27 | 2763228 | 2763321 | TTTCAACAGGCAGGTCACTC | AGAGCAGTCGCAACACACAT | NA | duplication | qPCR | yes | 0.856 | 1.47E-06 | 3.57E-06 | 2.50E-05 | NA of 20 |
| scaffold_95 | 89026 | 89127 | GCTCTTCAAGACCAGCTTCC | CGGAAATCCTCTTCTCTGCT | NA | duplication | qPCR | yes | 0.785 | 1.52E-05 | 2.88E-05 | 2.59E-04 | NA of 22 |
| groupV | 9798474 | 9800582 | GTGACAGAGGTGTTTCCACTCC | AGGACCCTTATCTGCATGACC | TACAACTACTCCACCCTTCAGTTCC | deletion | PCR | yes | NA | NA | NA | NA | 66 of 66 |
| groupVI | 13015885 | 13017146 | GTTGCTCATCTTCTCCTTCAGC | GCAGAAGATGCTACCAACAGG | CATGAAGACCAACCCCAAAG | deletion | PCR | yes | NA | NA | NA | NA | 54 of 65 |
| groupVI | 14524979 | 14528087 | ACGCTCCGTTTGCTCTCAC | CTTCTCTCTCAGCACATTCAGC | CCCTTCAGAGTCCAGAGTCAGTAAA | deletion | PCR | yes | NA | NA | NA | NA | 40 of 58 |
| groupVII | 9052408 | 9064505 | AAATCTCAATCTGCCCAACG | CCATCTTGAAATTGCCCCTA | ACAAAATGTGTCCGCAATGA | deletion | PCR | yes | NA | NA | NA | NA | 30 of 32 |
| groupX | 13225731 | 13231435 | CCAAAAGTAGCGACAGCACA | TGAAACCCAAAACTCCCAAA | TCGGTGGATTATGTCTGCAA | deletion | PCR | yes | NA | NA | NA | NA | 38 of 38 |
| groupXI | 12099302 | 12102118 | AGACCTAAACTTGACGTCCATCTG | TAAGGCTAGGCCAAGTTTAGGTC | GGTAGGAGCCATTTCAGGGTAT | deletion | PCR | yes | NA | NA | NA | NA | 56 of 62 |
