# Supplementary material for: Extensive Copy-Number Variation of Young Genes across Stickleback Populations
Source: PLoS Genet. 2014 Dec 4;10(12):e1004830. doi: 10.1371/journal.pgen.1004830 (PMC4256280; doi:10.1371/journal.pgen.1004830)
Supplement: Table S14 — Median dN, dS and dN/dS of genes using pairwise statistics with nine-spined stickleback orthologs. (PDF) [file pgen.1004830.s036.pdf]

Supplementary Table 14 - Median dN, dS and dN/dS of genes using pairwise statistics with nine-spined stickleback orthologs.

	**Genes analyzed**	**dN**	**dS**	**dN/dS**
**Genes**	7790	0.036	0.236	0.145
**LSG Singletons**	105	0.090	0.231	0.372
**LSG LSD**	6	0.084	0.150	0.403
**Non LSG LSDs**	201	0.049	0.198	0.215
**Non-LSG Paralogs**	5841	0.035	0.241	0.141
**Non-LSG Singletons**	1637	0.035	0.224	0.148
