# Supplementary material for: Extensive Copy-Number Variation of Young Genes across Stickleback Populations
Source: PLoS Genet. 2014 Dec 4;10(12):e1004830. doi: 10.1371/journal.pgen.1004830 (PMC4256280; doi:10.1371/journal.pgen.1004830)
Supplement: Table S15 — Genes consistent with positive selection with pairwise dN/dS >1 between three-spined stickleback and nine-spined stickleback. CDS represents protein-coding length, Length is the total gene length, and Cat is one of 5 gene categories: Non-LSG singletons, Non-LSG paralogs, Non-LSG LSD, LSG LSD and LSG singletons. (PDF) [file pgen.1004830.s037.pdf]

Supplementary Table 15 - Genes with molecular signals consistent with positive selection: with pairwise dN/dS > 1 between three-spined stickleback and nine-spined stickleback. CDS represents protein-coding length, Length is the total gene length, and Cat is one of 5 gene categories: Non-LSG singletons, Non-LSG paralogs, Non-LSG LSD, LSG LSD and LSG singletons.

**Chrom**	**Start**	**End**	**Gene ID**	**Cat**	**dN**	**dS**	**dN/dS**	**Putative Gene Name**
groupI	22269224	22284819	ENSGACG00000014580	Non-LSG Para	0.102	0.053	1.901	-
groupI	23354931	23355747	ENSGACG00000014931	LSG Sing	0.022	2E-04	109	-
groupII	9338516	9340144	ENSGACG00000015602	Non-LSG Sing	0.114	0.076	1.509	CEND1
groupII	21831392	21842872	ENSGACG00000017331	Non-LSG Para	0.039	0.013	3.048	SAMD4A
groupII	22032094	22033496	ENSGACG00000017381	Non-LSG Para	0.035	0.006	6.013	C1QTNF4_(1_of_2)
groupIII	34320	37281	ENSGACG00000012369	Non-LSG Para	0.035	0.021	1.667	TMBIM1_(1_of_2)
groupIII	5686659	5692925	ENSGACG00000014756	Non-LSG Para	0.087	0.06	1.456	F3_(2_of_2)
groupIII	8912646	8917308	ENSGACG00000015692	Non-LSG Sing	0.086	0.078	1.11	-
groupIII	13818366	13820187	ENSGACG00000017399	Non-LSG LSD	0.158	0.071	2.239	-
groupIV	5655539	5656546	ENSGACG00000017127	LSG Sing	0.067	0.06	1.127	-
groupIV	9153825	9154790	ENSGACG00000017726	LSG Sing	0.108	0.049	2.226	-
groupIV	13360789	13361899	ENSGACG00000018361	LSG Sing	0.09	0.022	4.028	-
groupV	10321126	10325727	ENSGACG00000008293	Non-LSG Para	0.046	0.014	3.162	-
groupVII	2454403	2462002	ENSGACG00000019055	Non-LSG Para	0.137	0.06	2.303	ATP6V0C_(1_of_2)
groupVII	8975874	8977495	ENSGACG00000019950	LSG Sing	0.145	0.092	1.573	-
groupVII	19279729	19280380	ENSGACG00000020498	Non-LSG Para	0.109	0.068	1.609	-
groupVII	21762040	21835045	ENSGACG00000020651	Non-LSG Para	0.284	0.274	1.036	-
groupVIII	11375615	11376881	ENSGACG00000009177	LSG Sing	0.048	0.038	1.275	NRXN2_(3_of_3)
groupVIII	18793689	18794985	ENSGACG00000014327	Non-LSG Para	0.097	0.056	1.737	-
groupIX	20186787	20196145	ENSGACG00000020011	Non-LSG Para	0.043	0.038	1.11	CHST14
groupX	7990079	7991807	ENSGACG00000005443	LSG Sing	0.051	0.027	1.897	-
groupX	9133358	9137767	ENSGACG00000006429	Non-LSG Sing	0.01	1E-04	97	TMEM196
groupX	14569171	14569474	ENSGACG00000009472	Non-LSG LSD	0.118	0.053	2.246	-
groupX	15052015	15053444	ENSGACG00000009770	LSG Sing	0.419	0.316	1.327	-
groupXI	1445790	1449722	ENSGACG00000005605	Non-LSG Para	0.042	0.015	2.779	ATP5G2

groupXI	14595152	14607919	ENSGACG00000014202	Non-LSG Para	0.137	0.104	1.316	SRCIN1_(1_of_2)
groupXI	16480036	16494230	ENSGACG00000014990	Non-LSG Sing	0.041	0.021	1.996	TMEM104
groupXII	1058851	1061564	ENSGACG00000002938	Non-LSG Para	0.115	0.053	2.182	B3GALT4
groupXIII	13894961	13895852	ENSGACG00000012150	Non-LSG Para	0.012	1E-04	99	IER5L_(2_of_2)
groupXIV	3270914	3272531	ENSGACG00000016170	LSG Sing	0.135	0.034	3.978	-
groupXVI	12810964	12837695	ENSGACG00000006588	Non-LSG Para	0.058	0.052	1.117	C19orf40_(1_of_2)
groupXVI	17276232	17296607	ENSGACG00000008632	Non-LSG Para	0.072	0.065	1.097	-
groupXVII	6299990	6308159	ENSGACG00000007453	Non-LSG Para	0.063	0.047	1.335	MAP2_(1_of_2)
groupXVII	8570784	8572274	ENSGACG00000008945	Non-LSG Sing	0.247	0.196	1.259	ITGB5
groupXVII	9139073	9175676	ENSGACG00000009348	Non-LSG Sing	0.049	0.048	1.025	ZNF362_(2_of_2)
groupXVIII	12498042	12504131	ENSGACG00000011855	Non-LSG Sing	0.012	1E-04	115	-
groupXVIII	15530697	15532931	ENSGACG00000013309	Non-LSG Sing	0.365	0.27	1.355	SLC25A26
groupXX	12327778	12330195	ENSGACG00000010681	Non-LSG Sing	0.067	0.04	1.666	-
groupXXI	7005038	7035292	ENSGACG00000002837	Non-LSG Para	0.252	0.219	1.15	SULF1
scaffold_1479	231	1508	ENSGACG00000001867	LSG Sing	0.147	0.131	1.122	-
scaffold_151	1246	2636	ENSGACG00000012693	Non-LSG Para	0.053	0.031	1.738	CACNA1H_(1_of_2)
scaffold_171	170781	172515	ENSGACG00000001053	Non-LSG LSD	0.032	0.025	1.27	-
scaffold_171	182320	184517	ENSGACG00000001057	Non-LSG LSD	0.037	0.023	1.596	-
scaffold_200	75101	75635	ENSGACG00000013519	Non-LSG LSD	0.09	0.041	2.221	-
scaffold_766	6853	9510	ENSGACG00000014743	Non-LSG LSD	0.066	0.013	5.243	-
scaffold_825	5539	6496	ENSGACG00000001812	Non-LSG LSD	0.112	0.049	2.31	-
