# Supplementary material for: Extensive Copy-Number Variation of Young Genes across Stickleback Populations
Source: PLoS Genet. 2014 Dec 4;10(12):e1004830. doi: 10.1371/journal.pgen.1004830 (PMC4256280; doi:10.1371/journal.pgen.1004830)
Supplement: Text S1 — Supporting text including methods and analyses. (PDF) [file pgen.1004830.s038.pdf]

Supporting Information

Extensive copy-number variation of young genes across stickleback genomes

Frédéric JJ Chain, Philine GD Feulner, Mahesh Panchal, Christophe Eizaguirre, Irene E Samonte, Martin Kalbe, Tobias L Lenz, Monika Stoll, Erich Bornberg-Bauer, Manfred Milinski and Thorsten BH Reusch

I. Supporting Text S1

1. Unmapped reads
2. CNV calling
3. CNV sharing across individuals and populations
4. LSGs and LSDs are young genes
5. Permutations to evaluate over- and under-representation of genes in CNVs
6. Genic CNVs
7. RNA genes
8. References

II. Supporting Figures

1. Matrix of CNV similarity between the 66 genomes
2. CNV validation
3. Frequency distribution of CNVs and sharing among individuals
4. Proportion of CNVs shared across individuals depending on gene overlap
5. Frequency distribution of CNVs depending on gene overlap
6. Average proportion of shared CNVs
7. Proportion of genes fully encompassed in CNVs
8. Frequency and sharing of CNV genes across all individuals
9. CNV genes shared across populations
10. Proportion of CNV genes that are specific to groups of individuals
11. Frequency and sharing of CNV genes across individuals within populations
12. Average proportion of shared CNV genes
13. Distribution and density of CNVs across the genome
14. CNV genes shared across individuals and segmental duplication overlap
15. Proportion of genes overlapping CNVs and segmental duplication overlap
16. V_{ST} scan across the genome in five parapatric populations
17. Overlap of BLAST hits of unmapped contigs
18. Structural properties of genes
19. CNV of immune genes
20. CNV of olfaction genes
21. CNV of group-specific gene expansions and losses
22. High CNV differences between groups

III. Supporting Tables

1. Sequencing statistics and sample information
2. CNV genotypes of bi-allelic CNVs
3. Private and fixed CNVs
4. Proportion of lineage-specific genes among CNVs
5. Lost genes

6. Unmapped genes
7. Number of CNVs, CNV genes and gene losses per individual
8. Gene ontology enrichment analysis of CNV genes
9. Gene ontology enrichment analysis of lineage-specific duplications
10. V_{ST} exon outliers
11. CNVs and CNV genes
12. Genes without BLAST hits
13. Primers used for CNV validation
14. Rates of molecular evolution (dN, dS and dN/dS)
15. Genes under positive selection

I. Supporting Text S1

1.Unmapped Reads

The detection of CNVs from mapping resequenced genomes depends on the completeness, organization and structure of the reference assembly, as well as the technological limitations of read mapping. For example, mapping approaches suffer to some extent from a reference genome ascertainment bias [1]; genomes more divergent from the reference will tend to map fewer reads. CNV deletions (including homozygous deletions, herein "gene losses") may thus be the result of a true genetic absence in the target genome or the result of sequence divergence compared to the reference genome, in which sequenced reads do not map to their respective loci due to substitutions or structural variation. To investigate the impact of sequence divergence on calling CNVs, we performed a *de novo* assembly and annotation of unmapped reads into contigs (see **Methods**). Among the 161,780 unmapped contigs, 88% had BLAT hits against the stickleback genome and 12.4% had protein sequence similarities with 9,357 annotated stickleback genes. Unmapped contigs were on average short (456bp), suggesting that the majority represents only parts of genes. We wanted to know if these unmapped genes explain gene losses, but they make up the same proportion of gene losses as expected by chance (50% of protein-coding genes in the genome as well as 50% (43/86) of the protein-coding gene losses). These results suggest that some of the gene losses (homozygous deletions) may actually be unmapped diverged sequences, but not all since half the gene losses return no similarities with the unmapped reads. We validated 6 (out of 6) homozygous deletions by PCR (see **Methods**). A minority of the unmapped contigs (803, mean size of 749bp) had significant BLASTX matches (e-value $<1x10^{-5}$) with only non-stickleback data (**Figure S17**), 87% of which hit proteins from other teleosts, and overall returning 168 annotated genes (**Table S6).** These are rather short contigs and as such could represent partial genes/duplicates or pseudogenes. Over 20% of these contigs are found in all 66 re-sequenced individuals (mean of 48 individuals), suggesting that the putative (partial) genes they correspond to are missing from the reference genome and may contribute to a slightly larger diversity of stickleback genes than are currently annotated. Some of these 803 unmapped contigs were also found to be specific to a particular population or continent. For example, 24 contigs were found in freshwater populations from both continents but not the marine population, whereas 7 were solely found in the marine population. We also found 97 contigs to be Atlantic-specific contigs, and 43 contigs not found in Atlantic freshwater populations, but most of these (36) are found in the Atlantic marine population.

2. CNV calling

The comparatively greater number of deletions called against the reference probably reflects the higher efficiency of detecting deletions rather than an indication of a shrinking genome [2,3]. In addition, because we call all deletions and duplications versus the reference, insertions are more difficult to detect and might often remain as unmapped reads either because the genes are not found in the reference or due to sequence or structural divergence from the reference. Our results from an analysis of the unmapped reads indicate that not all deletions can be explained by unmapped reads.

3. CNV sharing across individuals and populations

CNV sharing between individuals appears to occur mainly due to common ancestry (**Figure 1B, Figure S1**). Following these same analyses (that were performed using all CNVs including those that are in non-coding regions), we quantified the amount of CNV genes occurring across groups of individuals at different scales of divergence: (1) populations, (2) countries, and (3) continents. CNV genes follow the same general patterns as CNVs when it comes to sharing across individuals and populations. CNVs genes generally occur in few individuals and at low frequencies (**Figure S8,S9,S11**), and are thus mostly private although many are ancestral or recurrent; whereas 42% of CNV genes are population specific, likely due to recent mutations, 35% are shared across continents **(Figure S10)**. Like CNVs, we also found that the proportion of CNV genes shared between individuals decreases with geographic distance (**Figure S12**).

4. LSGs and LSDs are young genes

Stickleback LSGs and LSDs display some of the hallmarks of new genes. New genes often exhibit such properties as short gene lengths [4-6], narrow gene expression [7], and rapid molecular evolution [8-10]. These properties were investigated among stickleback genes. First, we found that protein-coding LSGs are on average significantly shorter (470bp) than non-LSGs (1,636bp; Mann-Whitney test, W = 2005796, $p < 0.0001$), while LSDs are intermediate in length (1,045bp, **Figure S18A)**. LSGs also have fewer exons (mean = 3.2) than non-LSGs (mean = 11.7; Mann-Whitney test, W = 19040434, $p < 0.0001$), while LSDs are intermediate in exon numbers (mean = 5.6, **Figure S18B**). Second, to test for narrow gene expression, we performed a digital expression profile analysis of ESTs from NCBI. LSGs and LSDs are expressed in significantly fewer tissue types compared to non-LSGs (Mann-Whitney test, W = 10917441, $p = 0.01867$). LSDs also have a larger proportion of genes that are absent from larval expression profiles, another indication of narrow expression profiles (LSG LSDs = 0.71 and non-LSG LSDs = 0.46 compared with LSG singletons = 0.32 and other non-LSGs = 0.34). Third, using transcriptomic sequences [11] from a sister species, the nine-spined stickleback, we calculated pairwise dN and dS (see **Methods**) to approximate genetic rates of evolution and functional constraints. This enabled the comparison between 7,075 putatively orthologous genes with length > 201bp and dS < 1, including 1,490 non-LSG singletons, 5303 non-LSG duplicates, 186 non-LSG LSDs, 6 LSG LSDs and 90 LSG singletons. Compared with non-LSGs, LSDs and LSGs have lower or equal dS (Mann-Whitney test, $p = 0.0002$ and $p = 0.3$, respectively) but up to three times higher values of dN (Mann-Whitney test, $p = 0.006$ and $p < 0.0001$, respectively) and dN/dS (Mann-Whitney test, $p < 0.0001$ for both LSD and LSGs, **Table S14**). In total, we found 46 genes with pairwise dN/dS ratios greater than one, consistent with

positive selection, consisting of 10% of LSDs and 5% of LSGs compared with only 0.5% of non-LSGs (**Table S15**). Evaluating these rates of molecular evolution between sister stickleback species required genes that originated before the speciation of nine-spined and three-spined sticklebacks over ~13 million years ago [12]. Therefore we surmise that relaxed or positive selection, which is consistent with the elevated dN and dN/dS in LSGs and LSDs, occurs in genes that have emerged several million years ago and that still persist in both stickleback species. Furthermore, all 23 genes identified by the Selectome database [13,14] as evolving under positive selection in sticklebacks are LSDs. Together, these findings support our classification and interpretation of LSGs and LSDs as young stickleback genes, which exhibit similar genetic patterns as young genes in other organisms.

5. Permutations to evaluate over- and under-representation of genes in CNVs

Random sampling of regions was performed to serve as a null expectation of gene overlap compared with the observed gene overlap of CNVs (see **Methods**). This allows to control for gene length, since lineage-specific genes are generally short (**Figure S18A**). We found that more genes were encompassed in both CNV deletions and duplications than every single random permutation ($p < 0.001$), and the same held true for the specific gene categories of LSG singletons, LSG LSDs and non-LSG LSDs. However, deletions encompassed fewer non-LSG paralogs and non-LSG singletons compared to all permutations ($p < 0.001$), and duplications did not differ significantly from random expectations for encompassing non-LSG paralogs (p = 0.765 for overrepresentation) or non-LSG singletons (p = 0.131 for overrepresentation). This indicates that regardless of gene length, CNVs are enriched with LSGs and LSDs, but not with other genes (in which deletions are actually underrepresented with non-LSGs). Similar results were found for RNA genes. Compared with permutations, there were more RNA genes in deletions (p = 0.047) and duplications (p = 0.004), but this signal was specifically due to LSG singletons and LSG LSDs (all with $p < 0.05$), rather than non-LSGs (all with $p > 0.05$).

6. Genic CNVs

Several genes have different copy-numbers between groups of populations making them candidates for adaptation. For example, some immune-related genes like *MYD88* and *MHC II* are duplicated or deleted in populations-specific patterns (**Figure S19**). The *MHC* is particularly interesting given the proposed adaptive value of *MHC II* copy-number variation in response to different pathogen communities [15]. Five olfactory receptor (*OR)* genes appear duplicated specifically in Norwegian individuals, while other *ORs* have been completely or partially deleted (**Figure S20**). Additional odorant-related genes such as a trace amine associated receptor *(TAAR)* and a *V2R* gene are duplicated in some or all of the Atlantic individuals (**Figure S20**). Other G-protein coupled receptor genes such as 4 taste receptor (*TAS1R1*) LSDs are present as a single copy in all Atlantic individuals but present in one to six copies in all Pacific individuals (**Figure S21**). We additionally found three *POGK* LSDs (related to the mammalian *POGO* transposable element with KRAB domain gene) that are lost in all Atlantic individuals or alternatively newly acquired in all Pacific individuals, whereas a piggyBac transposable element-derived ortholog (*PB*) is deleted in all Pacific individuals (**Figure S21**). An uncharacterized binding gene with a ribonuclease H-like domain has four to seven fold higher copy-numbers in Atlantic individuals (**Figure S22**). Other examples include an uncharacterized LSG (**Figure S22**) and a LSD membrane protein (*FAM159A*) that both have double the number of

copies in marine individuals compared to freshwater Atlantic populations, which in turn have double the number of copies compared to Pacific populations.

7. RNA genes

We found that CNVs affecting RNA genes have similar distributions and frequencies as those affecting protein-coding genes, suggesting an equally high turnover rate of RNA genes. Many RNA genes are associated with fragile sites [16] and commonly reside in segmental duplications as a result of lineage-specific gene family expansions, potentially imparting lineage-specific expression differences [17-24]. RNA genes may be essential or intermediary units allowing different populations to adapt to their local environments. One example of a population-specific expansion of an RNA gene is a small nuclear RNA *(U5)* that is deleted in all individuals except those from Canada (**Figure S22**).

8. References

1. Sousa V, Hey J (2013) Understanding the origin of species with genome-scale data: modelling gene flow. Nat Rev Genet 14: 404–414. doi:10.1038/nrg3446.

2. Abyzov A, Urban AE, Snyder M, Gerstein M (2011) CNVnator: An approach to discover, genotype, and characterize typical and atypical CNVs from family and population genome sequencing. Genome Res 21: 974–984. doi:10.1101/gr.114876.110.

3. Teo SM, Pawitan Y, Ku CS, Chia KS, Salim A (2012) Statistical challenges associated with detecting copy number variations with next-generation sequencing. Bioinformatics 28: 2711–2718. doi:10.1093/bioinformatics/bts535.

4. Yang L, Zou M, Fu B, He S (2013) Genome-wide identification, characterization, and expression analysis of lineage-specific genes within zebrafish. BMC Genomics 14: 1–1. doi:10.1186/1471-2164-14-65.

5. Neme R, Tautz D (2013) Phylogenetic patterns of emergence of new genes support a model of frequent de novo evolution. BMC Genomics 14: –117. doi:10.1186/1471-2164-14-117.

6. Zdobnov EM, Mering von C, Letunic I, Torrents D, Suyama M, et al. (2002) Comparative genome and proteome analysis of Anopheles gambiae and Drosophila melanogaster. Science 298: 149–159. doi:10.1126/science.1077061.

7. Donoghue MT, Keshavaiah C, Swamidatta SH, Spillane C (2011) Evolutionary origins of Brassicaceae specific genes in Arabidopsis thaliana. BMC Evolutionary Biology 11: 47. doi:10.1186/1471-2148-11-47.

8. Long M, Betrán E, Thornton K, Wang W (2003) The origin of new genes: glimpses from the young and old. Nat Rev Genet 4: 865–875. doi:10.1038/nrg1204.

9. Cain CE, Blekhman R, Marioni JC, Gilad Y (2011) Gene expression differences among primates are associated with changes in a histone epigenetic modification. Genetics 187: 1225–1234. doi:10.1534/genetics.110.126177.

10. Domazet-Lošo T, Tautz D (2003) An evolutionary analysis of orphan genes in Drosophila. Genome Res 13: 2213–2219. doi:10.1101/gr.1311003.

11. Guo B, Chain FJ, Bornberg-Bauer E, Leder EH, Merilä J (2013) Genomic divergence between nine- and three-spined sticklebacks. BMC Genomics 14: 756. doi:10.1186/1471-2164-14-756.

12. Bell MA, Stewart JD, Park PJ (2009) The World's Oldest Fossil Threespine Stickleback Fish. Copeia 2009: 256–265. doi:10.1643/CG-08-059.

13. Proux E, Studer RA, Moretti S, Robinson-Rechavi M (2009) Selectome: a database of positive selection. Nucleic Acids Res 37: D404–D407. doi:10.1093/nar/gkn768.

14. Moretti S, Laurenczy B, Gharib WH, Castella B, Kuzniar A, et al. (2013) Selectome update: quality control and computational improvements to a database of positive selection. Nucleic Acids Res. doi:10.1093/nar/gkt1065.

15. Eizaguirre C, Lenz TL (2010) Major histocompatibility complex polymorphism: dynamics and consequences of parasite-mediated local adaptation in fishes. J Fish Biology 77: 2023–2047. doi:10.1111/j.1095-8649.2010.02819.x.

16. Calin GA, Sevignani C, Dumitru CD, Hyslop T, Noch E, et al. (2004) Human microRNA genes are frequently located at fragile sites and genomic regions involved in cancers. Proc Natl Acad Sci USA 101: 2999–3004. doi:10.1073/pnas.0307323101.

17. Bentwich I, Avniel A, Karov Y, Aharonov R, Gilad S, et al. (2005) Identification of hundreds of conserved and nonconserved human microRNAs. Nature Genet 37: 766–770. doi:10.1038/ng1590.

18. Hertel J, Lindemeyer M, Missal K, Fried C, Tanzer A, et al. (2006) The expansion of the metazoan microRNA repertoire. BMC Genomics 7: 25. doi:10.1186/1471-2164-7-25.

19. Assis R, Kondrashov AS (2009) Rapid repetitive element-mediated expansion of piRNA clusters in mammalian evolution. Proc Natl Acad Sci USA 106: 7079–7082. doi:10.1073/pnas.0900523106.

20. Kaessmann H (2010) Origins, evolution, and phenotypic impact of new genes. Genome Res 20: 1313–1326. doi:10.1101/gr.101386.109.

21. Campo-Paysaa F, Sémon M, Cameron RA, Peterson KJ, Schubert M (2011) microRNA complements in deuterostomes: origin and evolution of

microRNAs. Evol Dev 13: 15–27. doi:10.1111/j.1525-142X.2010.00452.x.

22. Lu H-L, Tanguy S, Rispe C, Gauthier J-P, Walsh T, et al. (2011) Expansion of genes encoding piRNA-associated argonaute proteins in the pea aphid: diversification of expression profiles in different plastic morphs. PLoS ONE 6: e28051. doi:10.1371/journal.pone.0028051.s011.

23. Kutter C, Watt S, Stefflova K, Wilson MD, Goncalves A, et al. (2012) Rapid turnover of long noncoding RNAs and the evolution of gene expression. PLoS Genet 8: e1002841. doi:10.1371/journal.pgen.1002841.

24. Du Z-Q, Yang C-X, Rothschild MF, Ross JW (2013) Novel microRNA families expanded in the human genome. BMC Genomics 14: 98. doi:10.1186/1471-2164-14-98.
